# The Prognostic Value and Immunological Role of STEAP1 in Pan-Cancer: A Result of Data-Based Analysis

**DOI:** 10.1155/2022/8297011

**Published:** 2022-03-11

**Authors:** Chen Zhao, Kewei Xiong, Zhiqiang Ji, Fengming Liu, Xiangpan Li

**Affiliations:** ^1^Department of Oncology, Renmin Hospital of Wuhan University, Wuhan 430060, China; ^2^School of Mathematics and Statistics, Central China Normal University, Wuhan 430079, China; ^3^School of Public Health, Cheeloo College of Medicine, Shandong University, Jinan 250012, China

## Abstract

**Purpose:**

This study is aimed at systematically analyzing the expression, function, and prognostic value of six transmembrane epithelial antigen of the prostate 1 (STEAP1) in various cancers.

**Methods:**

The expressions of STEAP1 between normal and tumor tissues were analyzed using TCGA and GTEx. Clinicopathologic data was collected from GEPIA and TCGA. Prognostic analysis was conducted by Cox proportional hazard regression and Kaplan-Meier survival. DNA methylation, mutation features, and molecular subtypes of cancers were also investigated. The top-100 coexpressed genes with STEAP1 were involved in functional enrichment analysis. ESTIMATE algorithm was used to analyze the correlation between STEAP1 and immunity value. The relationships of STEAP1 and biomarkers including tumor mutational burden (TMB), microsatellite instability (MSI), and stemness score as well as chemosensitivity were also illustrated.

**Results:**

Among 33 cancers, STEAP1 was overexpressed in 19 cancers such as cervical squamous cell carcinoma and endocervical adenocarcinoma (CESC), colon adenocarcinoma, and lymphoid neoplasm diffuse large B cell lymphoma while was downregulated in 5 cancers such as adrenocortical carcinoma, breast invasive carcinoma (BRCA), and kidney chromophobe renal cell carcinoma. STEAP1 has significant prognostic relationships in multiple cancers. 15 cancers exhibited differences of DNA methylation including bladder urothelial carcinoma, BRCA, and CESC. STEAP1 expression was positively correlated to immune molecules especially in thyroid carcinoma and negatively especially in uveal melanoma. STEAP1 was associated with TMB and MSI in certain cancers. In addition, STEAP1 was connected with increased chemosensitivity of drugs such as trametinib and pimasertib.

**Conclusions:**

STEAP1 was an underlying target for prognostic prediction in different cancer types and a potential biomarker of TMB, MSI, tumor microenvironment, and chemosensitivity.

## 1. Introduction

Cancer is a global health issue of different genetic disorders and leads to a dominant cause of mortality worldwide [1, 2]. Carcinogenesis is dynamically driven by environmental selection forces, and tumor progression is triggered by regulation of gene expression such as DNA methylation, histone, and genome instability as well as alterations in transcriptome level between normal and tumor tissues [3–5].

Six transmembrane epithelial antigen of the prostate 1 (STEAP1) was firstly investigated as a potential biomarker in prostate cancer and has been identified to be overexpressed in several types of cancers [6]. STEAP1 locates at the cell membrane, has been suggested a role in intercellular communication, modulating the transport of small molecules and ions such as Na^+^, Ca^2+^, and K^+^, and releasing soluble cytokines and chemokines [7, 8]. STEAP1 also has metal reductase activity capable of reducing metal ion complexes and oxygen [9, 10].

Moreover, STEAP1 is highly expressed in multiple cancer tissues such as bladder, colon cancer, ovarian, and prostate and has the role of promoting invasion of tumor cells [10, 11]. In addition, several studies exploring the role of STEAP1 in cancer cells showed that its overexpression inhibits apoptosis and induces epithelial to mesenchymal transition, ultimately contributing to tumor progression and aggressiveness [12]. Although STEAP1 has been considered as an optimal target for T cell-based immunotherapy, with applications in a subset of cancer types nowadays [9], its expression and mutation landscape as well as prognostic value in many other cancers still remained to be investigated.

This study is aimed at systematically analyzing the expression, function, and prognostic value of STEAP1 in various cancers. The associations of STEAP1 with tumor microenvironment, immune-infiltrating, and other immune-related biomarkers in different cancer types were also investigated based on online web servers and R program.

## 2. Materials and Methods

### 2.1. Data Collection and Statistical Analysis

**Figure 1:**
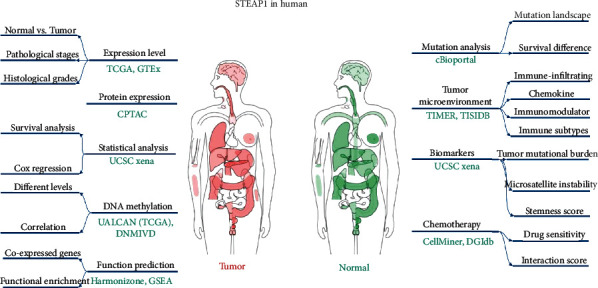
The workflow of this study and the distribution of STEAP1 expression level in human beings from GEPIA.

The workflow of this study is shown in Figure 1 with STEAP1 expression distribution in both tumor and healthy individuals. Transcriptional RNA sequencing (RNA-seq) data of 33 cancer types from The Cancer Genome Atlas (TCGA) and Genotype-Tissue Expression (GTEx) datasets from UCSC XENA [13] (https://xenabrowser.net/datapages/) were formatted in Transcripts Per Kilobase Million (TPM) according to Toil strategy [14] and transformed by log2. The 33 cancer types including in this study were as follows: adrenocortical carcinoma (ACC), bladder urothelial carcinoma (BLCA), breast invasive carcinoma (BRCA), cervical squamous cell carcinoma and endocervical adenocarcinoma (CESC), cholangiocarcinoma (CESC), colon adenocarcinoma (COAD), lymphoid neoplasm diffuse large B cell lymphoma (DLBC), esophageal carcinoma (ESCA), glioblastoma multiforme (GBM), head and neck squamous cell carcinoma (HNSC), kidney chromophobe renal cell carcinoma (KICH), kidney renal clear cell carcinoma (KIRC), kidney renal papillary cell carcinoma (KIRP), acute myeloid leukemia (LAML), brain lower grade glioma (LGG), liver hepatocellular carcinoma (LIHC), lung adenocarcinoma (LUAD), lung squamous cell carcinoma (LUSC), mesothelioma (MESO), ovarian serous cystadenocarcinoma (OV), pancreatic adenocarcinoma (PAAD), pheochromocytoma and paraganglioma (PCPG), prostate adenocarcinoma (PRAD), rectum adenocarcinoma (READ), sarcoma (SARC), skin cutaneous melanoma (SKCM), stomach adenocarcinoma (STAD), testicular germ cell tumors (TGCT), thyroid carcinoma (THCA), thymoma (THYM), uterine corpus endometrial carcinoma (UCEC), uterine carcinosarcoma (UCS), and uveal melanoma (UVM).

The differences of STEAP1 expression level between normal and tumor tissues in distinct cancer types were estimated by both nonpaired and paired statistical tests. To investigate the correlation between clinicopathologic features and STEAP1, Gene Expression Profiling Interactive Analysis [15] (GEPIA, http://gepia.cancer-pku.cn/) was used to analyze the STEAP1 expression differences among pathological stages in different cancer types. Furthermore, histological grades were also explored with clinical data downloaded from TCGA cohort (https://portal.gdc.cancer.gov/) where patients in G1 or G2 were stratified into an early-grade group whereas patients in G3 or G4 were stratified into an advanced-grade group. It should be noted that for BLCA with low and high grades, LGG with WHO G2 and G3 were included for the missing distinct histological grades.

The prognostic value of STEAP1 was performed by univariate Cox proportional hazard regression and Kaplan-Meier (KM) survival analysis with high- and low-expression groups at the optimal cutoff determined by survival package in R (version 3.6.3) for overall survival (OS), disease specific survival (DSS), and progression-free interval (PFI).

### 2.2. Biological Analysis and Tumor Microenvironment (TME)

Harmonizome [16] (https://maayanlab.cloud/Harmonizome/) is a web portal with a collection of information about 295496 attributes and 56720 genes from 114 datasets provided by 66 online resources. The top-100 coexpressed genes with STEAP1 were found in Harmonizome platform and were then validated in TCGA cohort of 33 cancer types. The genes were also included in the functional enrichment analysis regarding Gene Oncology (GO) and Kyoto Encyclopedia of Genes and Genomes (KEGG) for significant biological terms and pathway exploration in the clusterProfiler package (version 3.14.3) [17]. Patients were classified into high- and low-expression groups of STEAP1 at the median cutoff to calculate the logFoldChange (logFC) by the formula: logFC = log2 (high group/low group). Then, Gene Set Enrichment Analysis (GSEA) was performed based on the logFC value in each cancer types.

UALCAN [18] (http://ualcan.path.uab.edu/) is a resource for omics data analysis, a protein expression which was retrieved from Clinical Proteomic Tumor Analysis Consortium (CPTAC) Confirmatory/Discovery dataset. The methylation difference of STEAP1 between normal and tumor samples was evaluated. DNA methylation interactive visualization database (DNMIVD, http://119.3.41.228/dnmivd/index/) was used to investigate the methylation of STEAP1 regarding the correlation between STEAP1 expression level in Fragments Per Kilobase Million (FPKM) form and the methylation level of its promoter [19–21]. The mutation exploration was carried out by cBioPortal (https://www.cbioportal.org/), an open-access resource for interactive analysis of all-round cancer genomics datasets. The statistics of mutation frequency and corresponding mutation types in different cancer types were also examined.

TIMER database [22, 23] (https://cistrome.shinyapps.io/timer/) was utilized to analyze the immune-infiltrating of B cell, CD8+ T cell, CD4+ T cell, macrophage, neutrophil, and dentritic cell. TISIDB [24] (http://cis.hku.hk/TISIDB/index.php) is an online platform for tumor and immune system interaction, which integrates multiple heterogeneous data types including resources from PubMed database, high-throughput screening data, exome and RNA sequencing dataset of patient cohorts with immunotherapy, and well-orchestrated data from TCGA. It was adopted to systematically analyze the correlation of STEAP1 expression and molecular subtypes, TME including immune-infiltrating of lymphocytes, immunomodulators with immunoinhibitors, immunostimulators and major histocompatibility complex (MHC) molecules in humans (human leukocyte antigen, HLA), chemokine, and receptors for different cancer types. To explore the characteristics of relationships between TME and STEAP1 expression level, ESTIMATE algorithm which is aimed at computing the scores of stromal cells and immune cells in malignant tumor tissues using expression data was applied to assess the correlations of STEAP1 and immune scores by Pearson correlation coefficients.

### 2.3. Correlation of Biomarkers and STEAP1 Expression

Single-nucleotide variant data of patients was employed to calculate tumor mutational burden (TMB), and another biomarker microsatellite instability (MSI) of patients in different cancer types was taken to estimate the STEAP1 expression correlation. Moreover, RNA-based and DNA methylation-based stemness scores (RNAss and DNAss) were retrieved to analyze the correlations.

### 2.4. Drug Sensitivity and Interactions

CellMiner [25, 26] (https://discover.nci.nih.gov/cellminer/home.do) is designed for study integration and research of molecular and pharmacological data for the NCI-60 cancerous cell lines. DTP NCI-60 with average *z*-score and the corresponding RNA-seq data were downloaded. The correlations of STEAP1 expression level and drug scores were measured by Pearson correlation coefficients. In addition, the Drug Gene Interaction Database [27] (DGIdb, https://dgidb.genome.wustl.edu/search_interactions) providing connections between genes and the known or potential drug associations was used to study the interactions of top-100 coexpressed genes from Harmonizome with their target approved antineoplastics and immunotherapeutics.

## 3. Results

### 3.1. STEAP1 Expression Levels among Clinicopathological Features

**Figure 2:**
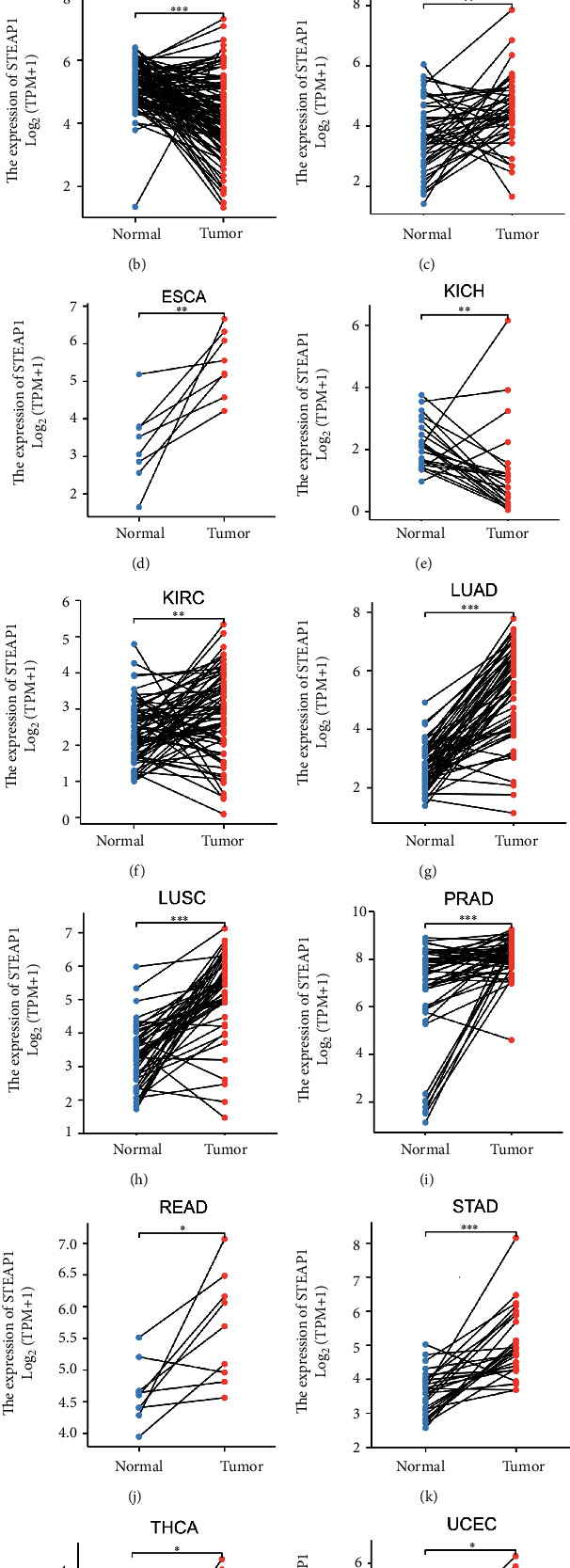
(a) The differences of STEAP1 expression level between normal and tumor tissues in 33 cancer types based on TCGA and GTEx cohorts, which were estimated by Wilcoxon rank-sum test (^∗∗∗^*p* < 0.001, ^∗∗^*p* < 0.01, and ^∗^*p* < 0.05; ns: no significance). (b–m) The difference of STEAP1 expression level between normal and tumor tissues in BRCA, ESCA, HNSC, KICH, KIRC, LUAD, LUSC, PRAD, READ, STAD, THCA, and UCEC from TCGA was estimated by paired test.

Based on TCGA and GTEx cohorts, as shown in Figure 2(a), STEAP1 was significantly upregulated in CESC, COAD, DLBC, ESCA, GBM, HNSC, KIRC, LGG, LUAD, LUSC, OV, PAAD, PRAD, READ, SKCM, STAD, TGCT, THYM, UCEC, and UCS whereas it was downregulated in ACC, BRCA, KICH, LAML, and THCA. It indicated that the expression of STEAP1 exhibited heterogeneity in urinary cancers such as renal cell carcinoma and female reproductive disorders such as breast invasive cancer, ovarian cancer, and endometrial carcinoma but consistency in digestive cancer types such as colorectal cancer and gastric cancer as well as respiratory cancers. Paired tests demonstrated STEAP1 had significant different expression on BRCA, ESCA, HNSC, KICH, KIRC, LUAD, LUSC, PRAD, READ, STAD, THCA, and UCEC between normal and tumor tissues (Figures 2(b)–2(m)).

**Figure 3:**
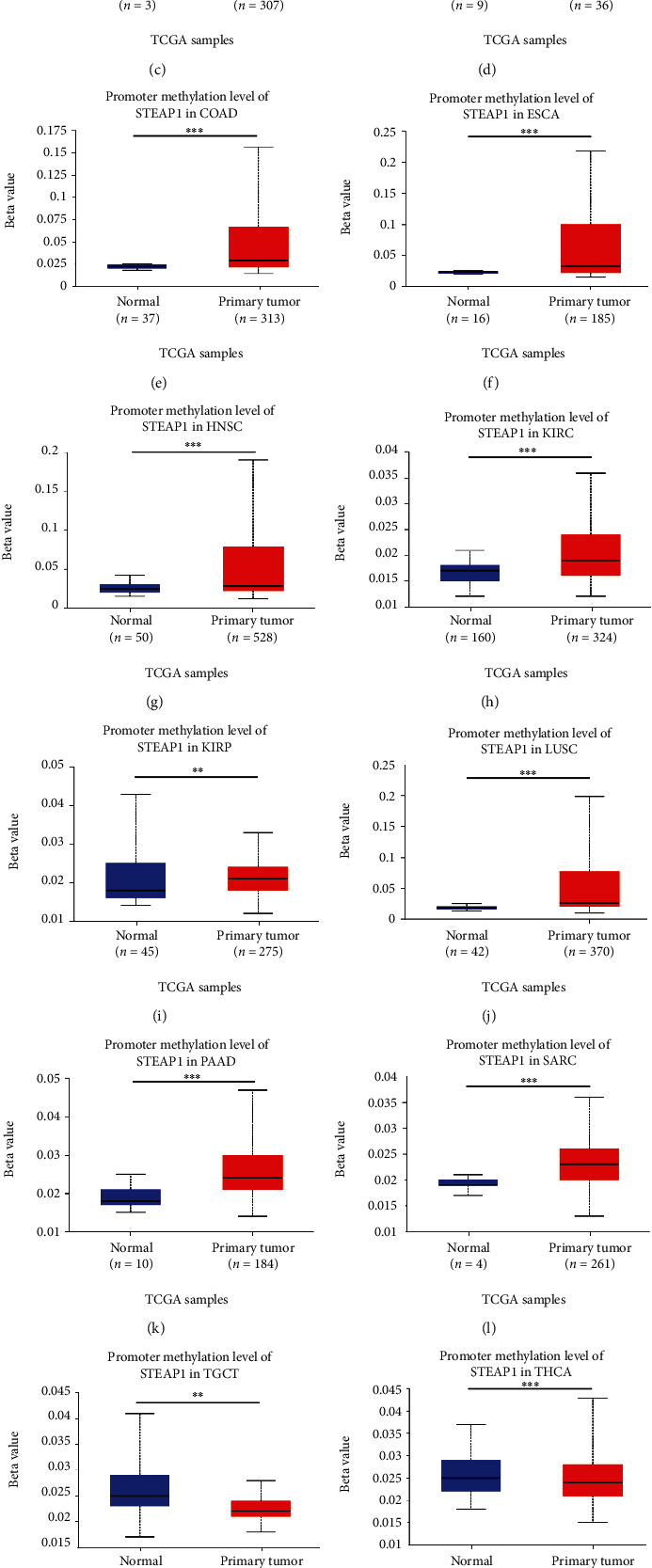
Variations of promoter methylation of STEAP1 in several cancers according to the evidence from UALCAN.

The total protein expression of STEAP1 in breast cancer, colon cancer, lung adenocarcinoma, and UCEC samples was significantly higher than that in normal samples with *p* value less than 0.001 (Figure S1a-d) whereas primary ovarian tumor samples exhibited lower STEAP1 expression level than normal samples with *p* value less than 0.01 (Figure S1e). The promoter methylation levels of STEAP1 between normal and tumor tissues exerted significant differences in 15 cancers such as BLCA, BRCA, and CESC (Figure 3). According to DNMIVD, STEAP1 expression levels were negatively correlated with methylation levels of its promoter calculated by Spearman correlation coefficients in BRCA (*r* = −0.11, Figure S2a), PRAD (*r* = −0.1, Figure S2d), SKCM (*r* = −0.14, Figure S2e), and UCEC (*r* = −0.52, Figure S2f) while having positive connections with the methylation of its promoter in COAD (*r* = 0.11, Figure S2b) and LUSC (*r* = 0.11, Figure S2c).

**Figure 4:**
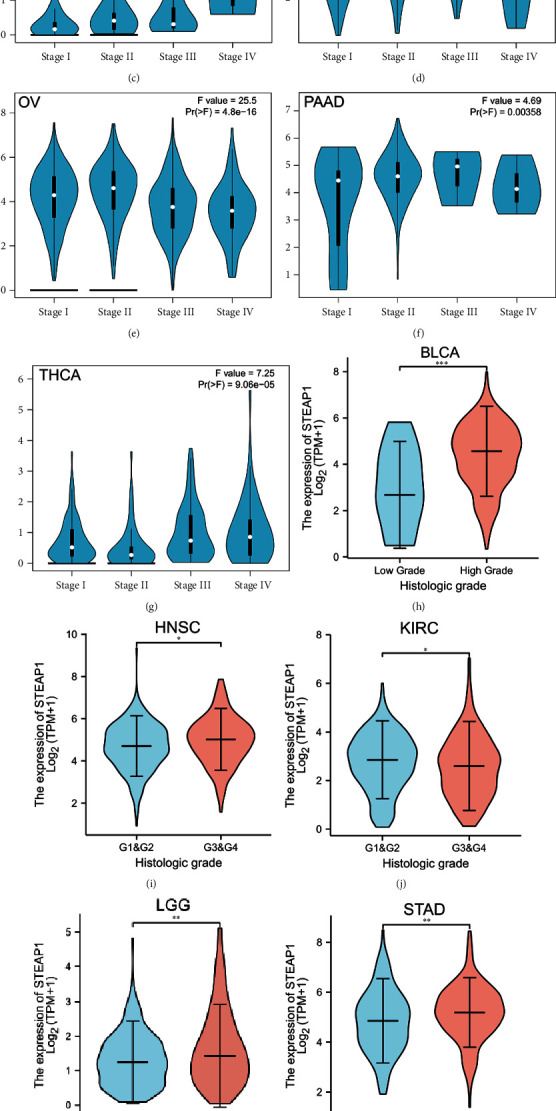
(a–g) The difference of STEAP1 expression level between distinct pathological stages in ACC, DLBC, KICH, LUAD, OV, PAAD, and THCA, from GEPIA. (h) The difference of STEAP1 expression level between low and high histological grades in BLCA. (i, j) The difference of STEAP1 expression level between early and advanced grades in HNSC and KIRC. (k) The difference of STEAP1 expression level between WHO G2 and G3 in LGG. (l, m) The difference of STEAP1 expression level between early and advanced grades in STAD and UCEC.

Kruskal-Wallis (KW) test performed on GEPIA website revealed that STEAP1 expression altered significantly with pathological stages in ACC, DLBC, KICH, LUAD, OV, PAAD, and THCA (Figures 4(a)–4(g)). In addition, patients with high grade or advanced-grade group in BLCA (Figure 4(h)), HNSC (Figure 4(i)), LGG (Figure 4(k)), and STAD (Figure 4(l)) had significant higher STEAP1 expression while individuals in high grade or advanced-grade group by our custom integration with KIRC (Figure 4(j)) and UCEC (Figure 4(m)) had opposite results.

### 3.2. Prognostic Analysis

**Figure 5:**
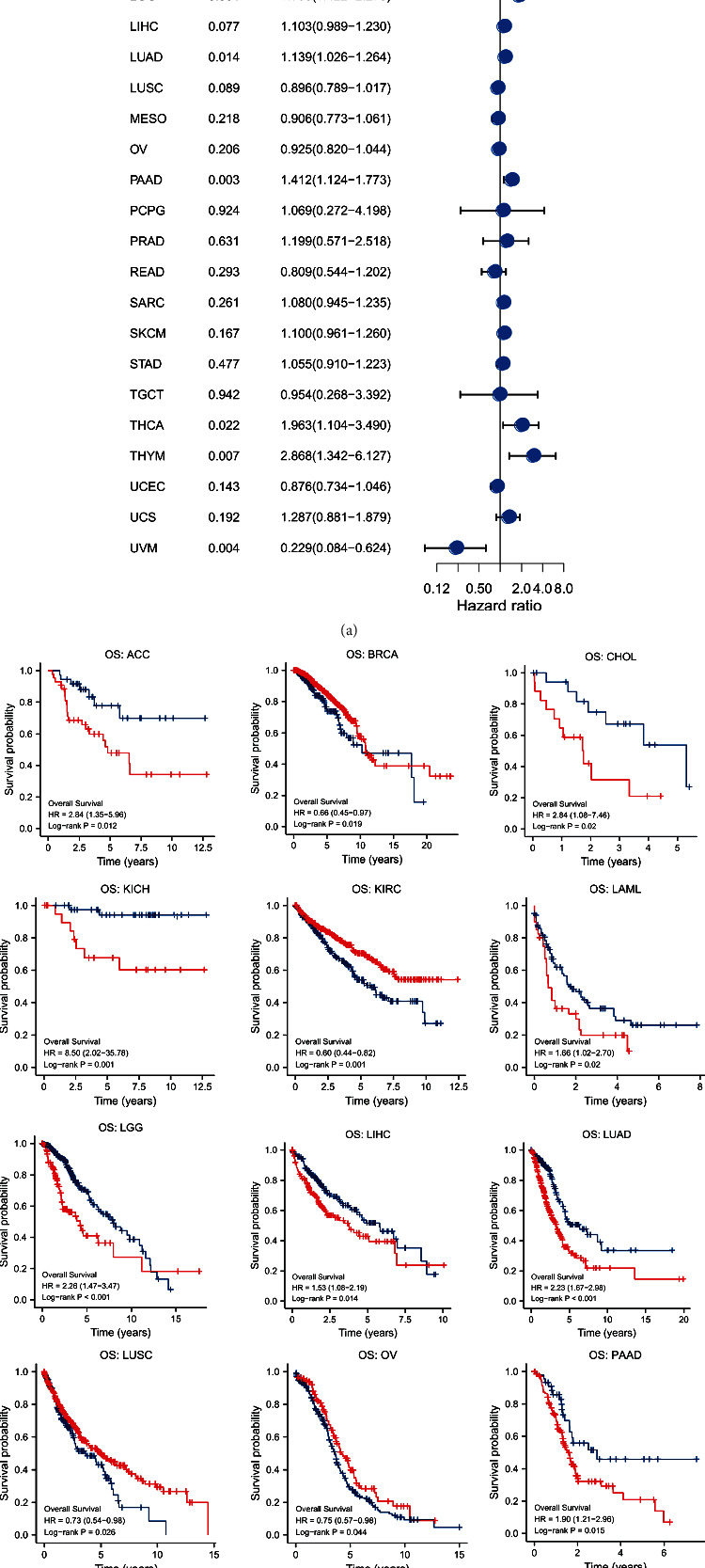
(a) A univariate Cox proportional hazard regression of OS with STEAP1 expression was illustrated by a forest plot. (b) KM survival analysis of OS between high- and low-expression groups of STEAP1.

By conducting univariate Cox proportional hazard regression, Figure 5(a) implied that STEAP1 was a risk factor in ACC (*p* < 0.001, hazard ratio (HR) = 1.669, and 95%confidence interval (CI) = 1.266‐2.199), CHOL (*p* = 0.030, HR = 1.468, and 95%CI = 1.038‐2.075), GBM (*p* = 0.011, HR = 1.222, and 95%CI = 1.047‐1.428), KICH (*p* < 0.001, HR = 3.410, and 95%CI = 1.643‐7.079), KIRP (*p* = 0.013, HR = 1.579, and 95%CI = 1.102‐2.261), LAML (*p* = 0.033, HR = 3.488, and 95%CI = 1.109‐10.975), LGG (*p* < 0.001, HR = 1.798, and 95%CI = 1.422‐2.275), LUAD (*p* = 0.014, HR = 1.139, and 95%CI = 1.026‐1.264), PAAD (*p* = 0.003, HR = 1.412, and 95%CI = 1.124‐1.773), THCA (*p* = 0.022, HR = 1.963, and 95%CI = 1.104‐3.490), and THYM (*p* = 0.007, HR = 2.868, and 95%CI = 1.342‐6.127) for OS while it played an underlying favorable factor in UVM (*p* = 0.004, HR = 0.229, and 95%CI = 0.084‐0.624).

KM survival analysis for OS revealed that patients with ACC, CHOL, KICH, LAML, LGG, LIHC, LUAD, PAAD, and THYM in the high-expression group at the optimal cutoff experienced poor prognosis whereas folks with BRCA, KIRC, LUSC, OV, UCEC, and UVM experienced better survival with high expression of STEAP1 (Figure 5(b)).

The univariate Cox regression for DSS revealed that STEAP1 was a risk factor for ACC, GBM, KICH, KIRP, LGG, LUAD, PAAD, and THCA but a favorable factor for LUSC and UVM (Figure S3a). Survival differences for DSS demonstrated that patients with higher STEAP1 expression in ACC, glioma, KICH, KIRP, LAML, LUAD, and PAAD experienced poor prognosis whereas patients in the high-expression group of STEAP1 with BRCA, ESCA, KIRC, LIHC, UCEC, and UVM had better survival outcomes (Figure S3b). In addition, a univariate Cox regression for PFI indicated that STEAP1 took a risk factor in GBM, KICH, KIRP, LGG, LUAD, and PAAD but a favorable factor in READ and UVM (Figure S4a). KM analysis for PFI demonstrated that patients in the high-expression group stratified by the optimal cutoff with ACC, glioma, KICH, KIRP, LUAD, PAAD, PRAD, and THCA had poor survival while individuals in the same group with BRCA, KIRC, READ, and UVM experienced better prognosis (Figure S4b).

### 3.3. Genomic Characteristics

**Figure 6:**
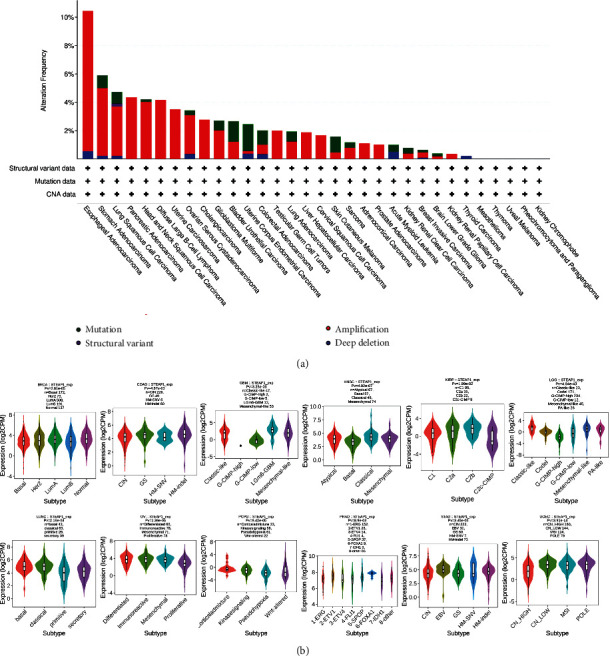
(a) The mutation frequency and corresponding mutation types of STEAP1 in different cancers. (b) The differences of STEAP1 expression levels among distinctive molecular subtypes in BRCA, COAD, GBM, HNSC, KIRP, LGG, LUSC, OV, PCPG, PRAD, STAD, and UCEC.

The mutation frequency and types were analyzed in cBioPortal. As shown in Figure 6(a), the highest frequency of STEAP1 alteration existed in esophageal adenocarcinoma with the amplification accounting for the most. In UCEC patients, mutation accounted for the most among the four alteration types including mutation, structural variant, amplification, and deep deletion. The results of molecular subtypes for different cancer types illustrated in Figure 6(b) implied that STEAP1 expression level exhibited statistically significant differences in BRCA, COAD, GBM, HNSC, KIRP, LGG, LUSC, OV, PCPG, PRAD, STAD, and UCEC.

### 3.4. Functional Enrichment Exploration

**Figure 7:**
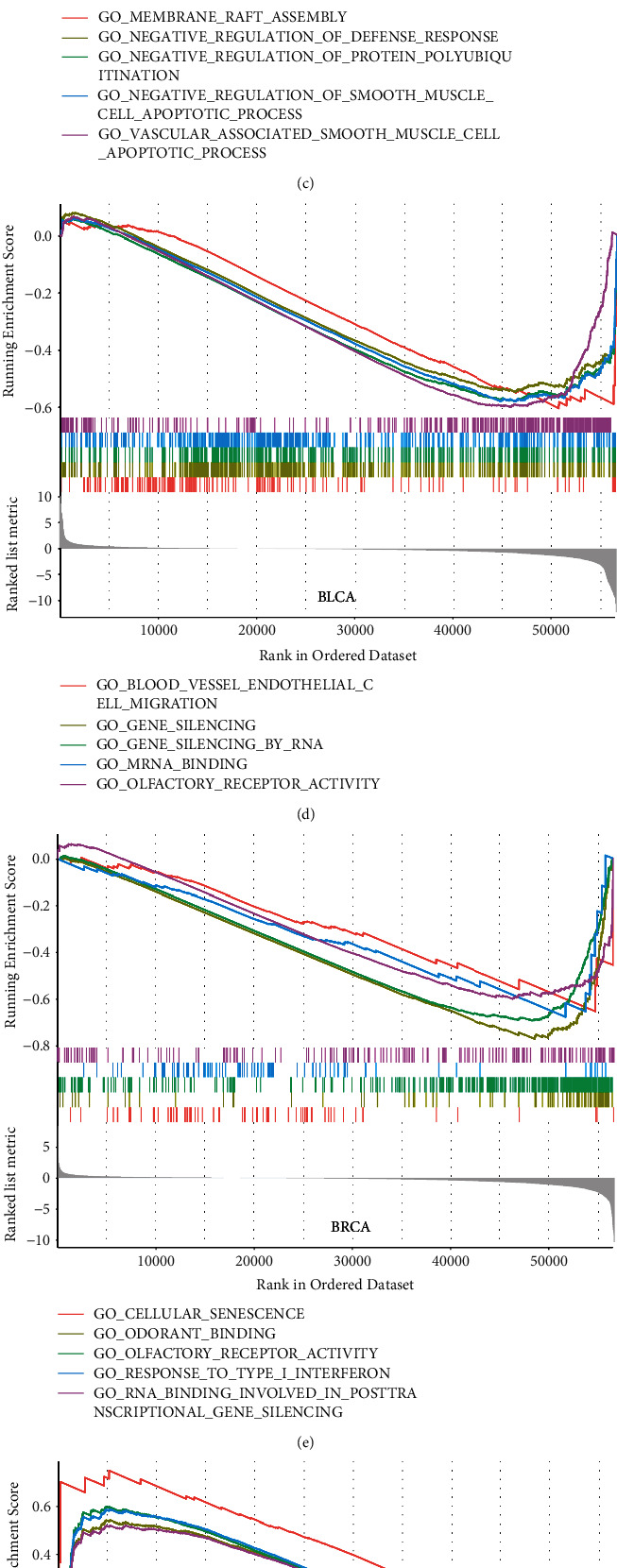
(a) Correlations of top-30 correlated genes with STEAP1 obtained from Harmonizome website in different cancer types were shown by a heatmap. (b) The network of significant signaling-related GO terms and KEGG pathways. The blue nodes represented GO terms, and the red nodes represented genes. The size demonstrated the number of enriched genes. (c–h) GSEA enrichment results.

The top-100 correlated genes found in Harmonizome (Table S1) were validated in TCGA cohort. It implied that STEAP2 was most positively correlated with STEAP1 in all cancer types as shown in a heatmap (Figure 7(a)). Then, the 100 genes were included in functional enrichment prediction containing GO and KEGG (Table S2). The significant (*p*.adjusted < 0.05) signaling-related terms and pathways represented in Figure 7(b) in network style demonstrated that the 100 genes correlated with STEAP1 were enriched in Hippo signaling, integrin-mediated signaling pathway, hepatocyte growth factor receptor signaling pathway, epidermal growth factor receptor signaling pathway, Hippo signaling pathway—multiple species, Hippo signaling pathway, and PI3K-Akt signaling pathway. The outcomes of GSEA analysis are shown in Table S3, and the most significant enriched terms of ACC, BLCA, BRCA, CESC, CHOL, and COAD are shown in Figures 7(c)–7(h).

### 3.5. Investigation to TME Related to STEAP1

**Figure 8:**
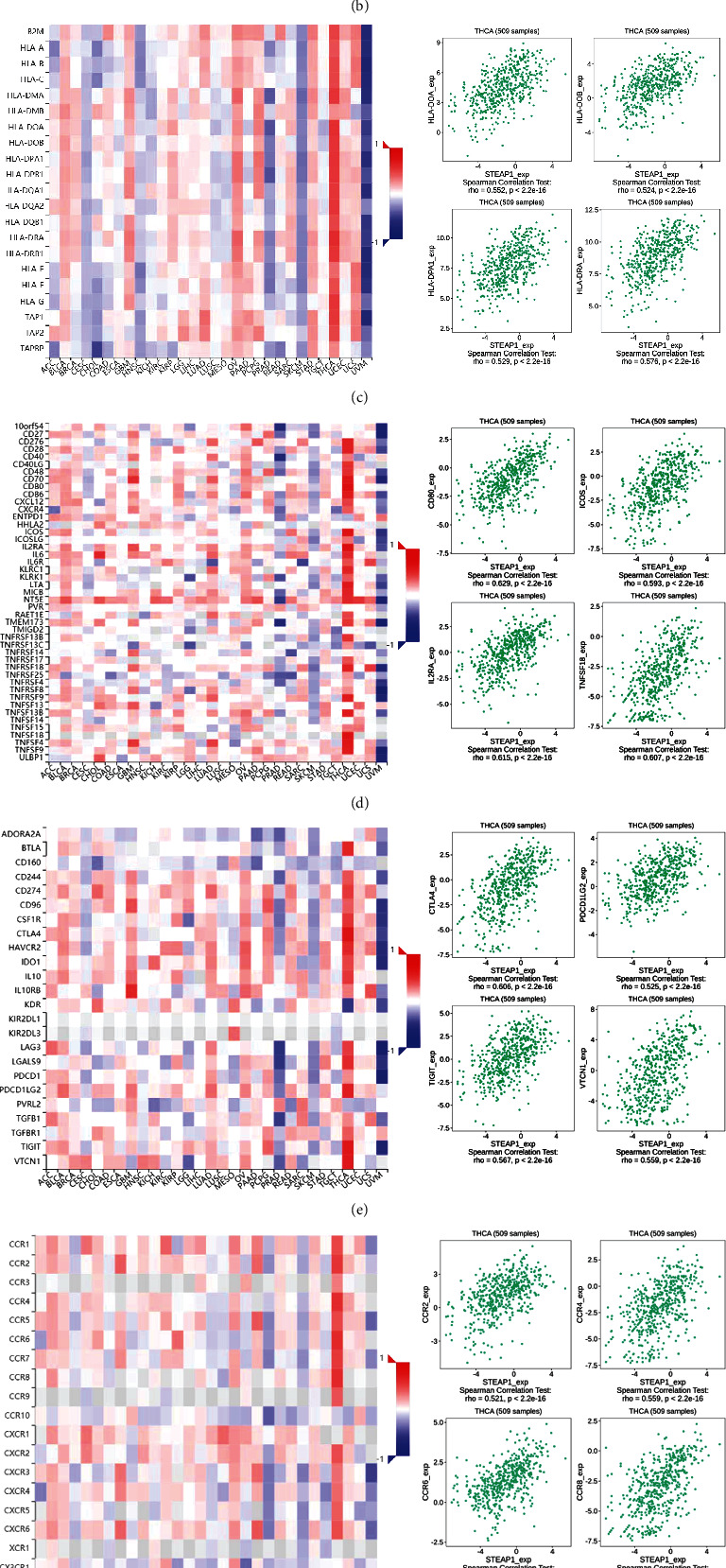
(a) The correlations of STEAP1 expression and immune-infiltrating levels of B cell, CD8^+^ T cell, CD4^+^ T cell, macrophage, neutrophil, and dentritic cell in different cancer types calculated by TIMER. (b–g) The correlations of STEAP1 expression and immune-infiltrating levels of 28 cell types, MHC molecules, immunostimulators, immunoinhibitors, receptors, and chemokines calculated by TISIDB.

According to the evidence from TIMER database (Figure 8(a)), STEAP1 expression level in THCA was positively related to the immune-infiltrating levels of all six cell types where neutrophil and dendritic cell exhibited the maximum correlation coefficients. And the immune-infiltrating of neutrophil correlated with STEAP1 expression level in UVM had the maximum positive coefficient with the value of 0.698. However, STEAP1 expression level in THYM was negatively related to the immune-infiltrating levels of CD4^+^ T cell and dendritic cell with the top-2 negative coefficients of -0.489 and -0.426, respectively. It should be also noted that STEAP1 expression in BRCA, COAD, KIRP, and PAAD was positively correlated with the most of immune-infiltrating among the six cell types. To further validate the results, TISIDB revealed that STEAP1 expression in THCA was positively related to all cell types for immune-infiltrating including Tcm CD4, Tem CD8, Th1, and Treg, and it was negatively related to infiltrating levels of all cell types in UVM (Figure 8(b)). Moreover, STEAP1 expression had the most significantly positive correlation with molecules in THCA and the most significantly negatively correlation with molecules in UVM generally of MHC molecules in human beings including HLA-DOA, HLA-DOB, HLA-DPA1, and HLA-DRA (Figure 8(c)); immune stimulators including CD80, ICOS, ILSRA, and TNFSF18 (Figure 8(d)); immune inhibitors including CTLA4, PDCD1LG2, TIGIT, and VTCN1 (Figure 8(e)); receptors including CCR2, CCR4, CCR6, and CCR8 (Figure 8(f)); and chemokines including CCL13, CCL17, CCL20, and CCL22 (Figure 8(g)).

The differences of STEAP1 expression between distinctive immune subtypes including wound healing (C1), IFN-*γ* dominant (C2), inflammatory (C3), lymphocyte depleted (C4), immunologically quiet (C5), and TGF-*β* dominant (C6) were also analyzed. It illustrated that STEAP1 had the lowest expression level in C4 of BRCA, LUAD, and STAD but highest levels in C6 of COAD, in C4 of KIRC, and PRAD. It also exhibited significant differences among immunophenotypes in CHOL, GBM, HSNC, LGG, and UVM. Moreover, LUSC, PAAD, and READ held the lowest STEAP1 expression levels in C3 (Figure S5).

**Figure 9:**
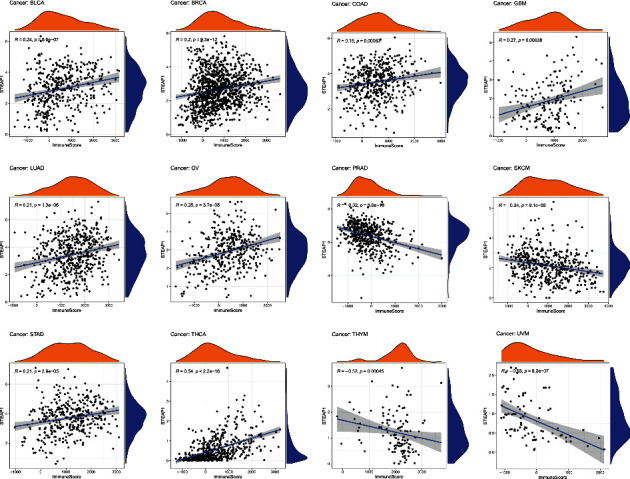
Significant correlations of STEAP1 expression and immune score were examined by ESTIMATE algorithm.

Additional approaches to exploring the relationship of TME and STEAP1 expression, immune scores and stromal scores in different cancer types were computed. Considering the threshold *p*-value of 0.001, scatter plots showed that STEAP1 expression was positively correlated with immune scores in BLCA, BRCA, COAD, GBM, LUAD, OV, STAD, THCA whereas negatively correlated with immune scores in SKCM, THYM and UVM (Figure 9). As shown in Figure S6, STEAP1 expression levels were positively correlated with stromal scores in BLCA, BRCA, DLBC, GBM, KIRP, LUAD, OV, THCA, and THYM while negatively in PRAD and UVM.

### 3.6. Tumor Biomarkers

**Figure 10:**
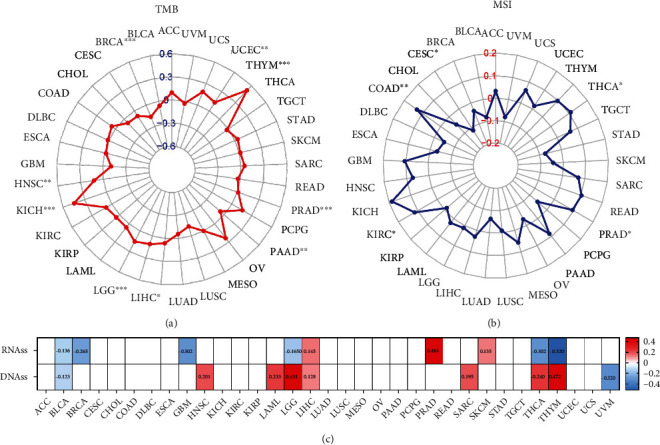
(a, b) The relationship between STEAP1 expression and TMB as well as MSI was illustrated by a radar plot. (c) Correlations between STEAP1 expression and stemness score based on RNA-seq and DNA methylation were illustrated by a heatmap.

The correlations of TMB for 33 cancer types and STEAP1 expression presented in Table S4 implied that expression levels had significantly positive correlations with HNSC, KICH, LGG, LIHC, PAAD, PRAD, THYM, and UCEC but a negative correlation with BRCA. It was apparent from Figure 10(a) that STEAP1 expression level in THYM had the maximum coefficients with *p* value < 0.001 followed by KICH. In addition, the correlations of MSI for 33 cancer types and STEAP1 expression were also calculated (Table S5).  Figure 10(b) showed by a radar plot that expression levels had significantly positive connections with COAD, KIRC, PRAD, and THCA but a negative connection with CESC. Furthermore, the correlation of stemness scores based on RNA (RN) as well as DNA methylation and STEAP1 expression level was computed. It showed that STEAP1 expression had the most positive correlation of RNAss in PRAD with the value of 0.464 of DNAss in THYM with the value of 0.472 followed by in LGG with the value of 0.431. Interestingly, DNAss of THYM held the most significantly negative correlation of RNAss with the value of -0.520.

### 3.7. Clinical Chemotherapies

**Figure 11:**
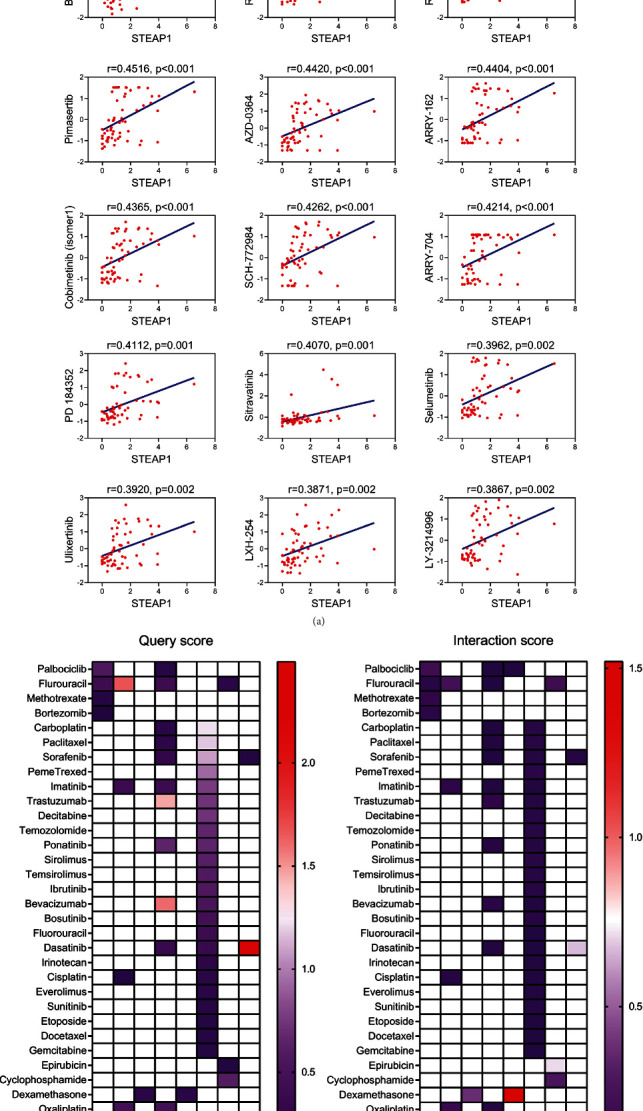
(a) The associations of STEAP1 expression and drug sensitivity based on CellMiner dataset were ordered according to *p* value from small to large (ERK inhibitor: AZD-0364 and SCH-772984; MEK inhibitor: TAK-733, PD-0325901, trametinib, RO-5162766, RO-4987655, pimasertib, ARRY-162, cobimetinib (isomer1), ARRY-704, PD184352, and selumetinib; MET inhibitor: BMS-777607; and RTK inhibitor: sitravatinib). (b, c) The query score and interaction score for approved antineoplastic and immunotherapies of coexpressed genes with STEAP1.

Drug sensitivity measured by *z*-score was assessed together with STEAP1 expression levels (Table S6). The most significantly correlated drugs are shown in Figure 11(a), indicated that STEAP1 was associated with increased sensitivity of cells to MEK, MET, ERK, RTK, and RAF inhibitors. The correlations between coexpressed genes with STEAP1 and approved antineoplastic as well as immunotherapies measured by query scores and interaction scores are shown in Figures 11(b) and 11(c), respectively.

## 4. Discussion

STEAP1 was previously validated as a promising target to discriminate adjacent and tumor samples and a useful tool for antibody therapies in different solid tumors [28, 29]. Nonetheless, there were few systematic studies of STEAP1 in pan-cancer by bioinformatics approaches. The present study was designed to determine the expression pattern, prognostic value, and potential function of STEAP1 in different cancer types systematically.

In the present study, we firstly demonstrated that STEAP1 was overexpressed at mRNA levels in CESC, COAD, DLBC, ESCA, GBM, HNSC, KIRC, LGG, LUAD, LUSC, OV, PAAD, PRAD, READ, SKCM, STAD, TGCT, THYM, UCEC, and UCS cancer tissues compared with corresponding adjacent tissues while STEAP1 was downregulated in ACC, BRCA, KICH, LAML, and THCA, indicating that STEAP1 served as a potential oncogene in most cancers. The finding was consistent with the expression differences in cancers including colon, cervix, gastric, ovary, prostate, pancreas, and testis [9, 30]. STEAP1 expression was upregulated in LUAD cells, and knockdown of STEAP1 suppressed the proliferation and invasion of LUAD epithelial cells [31]. STEAP1 exhibited higher expression levels in advanced stages of ACC, KICH, OV, and THCA. Increased STEAP1 expressions were associated with high grade of BLCA, HNSC, LGG, and STAD while high grade of KIRC and UCEC was associated with lower STEAP1 expression levels, suggested STEAP1 could be considered an underlying biomarker in certain cancers.

Another finding in prostate cancer revealed that knockdown of STEAP1 could inhibit cell growth and induce apoptosis in prostate cancer cells [32]. The further study illustrated STEAP1 can also regulate EMT via JAK2/STAT3 signaling pathway [33]. Therefore, future studies on the function of STEAP1 in cell death, proliferation, and tumor migration with wet-lab approaches need to be explored.

Survival analysis based on KM curves revealed that the upregulated expression of SETAP1 correlated to worse OS survival in ACC, CHOL, KICH, LAML, LGG, LIHC, LUAD, PAAD, and THYM but better OS survival in BRCA, KIRC, LUSC, OV, UCEC, and UVM. Together with DSS and PFI data, STEAP1 played a favorable prognostic role in BRCA and KIRC while a risk factor in ACC, KICH, LUAD, and PAAD. The results of BRCA and LUAD patients were consistent with previous studies via bioinformatics analysis [7, 31]. Furthermore, Liu et al. developed a prognostic risk model for glioblastoma multiforme based on six genes including STEAP1 and STEAP2, indicating that STEAP1 also served as an underlying predicting factor in risk stratification of cancer [34]. Interestingly, a strong positive correlation of STEAP1 and STEAP2 average expression of pan-cancer was identified in our study including glioma and other different tumor tissues. Oppositely, Zhang et al. indicated that STEAP1 was closely related to overall survival in gastric cancer patients [30], contradicting with TCGA cohort in our study, which is probably caused by racial differences: Zhang et al.'s study included only Chinese people while TCGA cohort mainly contains Caucasian.

DNA methylation is one of the epigenetic mechanisms in transcriptional regulation, and aberrant DNA methylation is a nearly universal finding in cancer [35, 36]. In the present study, promoter methylation levels of STEAP1 were upregulated in BRCA, CHOL, KIRP, and SARC while were downregulated in BLCA and THCA accorded with classical models. Furthermore, high promoter methylation of STEAP1 correlated to high STEAP1 expression levels in tumor samples of CESC, COAD, ESCA, HNSC, KIRC, LUSC, and PAAD and low methylation levels was associated with lower STEAP1 expression in TGCT and UCEC. Spainhour et al. illustrated that there was a substantial amount of positive correlation between DNA methylation and gene expression using TCGA cohort, which also revealed the effects of methylation on gene expression are largely tissue independent [37]. In linear correlation, negative relationships were examined between STEAP1 and several cancers such as BRCA, PRAD, SKCM, and UCEC but positive connections in COAD and LUSC. Consistent with a previous study, STEAP1 was downregulated in prostate cancer tissues compared with normal samples and negatively associated with promoter methylation levels integrating both vitro and silico analysis [38].

To further probe into the genomic alternations of STEAP1 in different cancers, data from TCGA were analyzed in cBioPortal. The results suggested that changes in the STEAP1 gene mainly occur in ESCA, STAD, LUSC, and PAAD. Hence, the correlation of STEAP1 expression and molecular subtypes demonstrated unity with survival analysis in this study. Among the top-100 coexpressed genes with STEAP1, STEAP2 showed the extremely positive correlation with STEAP1 in TCGA cohort. STEAP2 was also reported an overexpressed gene inhibiting apoptosis in several human cancers, especially prostate cancer [9]. Additionally, the 100 genes were involved in functional enrichment analysis. The results implied that genes were significantly enriched in Hippo signaling pathway. Hippo signaling involves in cell proliferation, tissue homeostasis, differentiation, apoptosis, and regeneration. Dysregulation of Hippo signaling, especially the hyperactivation of its downstream effectors YAP/TAZ, can lead to uncontrolled cell proliferation and malignant transformation [39–41]. PI3K-Akt, EGFR, HGFR, and integrin-mediated signaling pathway were also enriched in this study. The PI3K-Akt signaling way played a vital part in tumorigenesis and progression such as brain, breast, and endometrial [42]. A previous study confirmed STEAP2 was downregulated in breast cancer, and its upregulation inhibited tumor proliferation, invasion, and metastasis by suppressing PI3K-Akt signaling pathway *in vitro* and *in vivo* [43]. It could be assumed that STEAP1 may play a similar role in PI3K-Akt signaling pathway. Acting as EMT transcription factors, EGFR and STEAP1 were proved to be highly expressed among pulmonary neuroendocrine carcinomas and downregulated in carcinoid tumors [44], which suggested that STEAP1 may have interaction with EGFR pathway in the process of EMT. However, there are few studies about STEAP1 and HGFR or integrin-mediated signaling pathway. Further research is required to explore their interaction mechanism.

Tumorigenesis is highly associated with TME, which has limited or poorly differentiated vasculature and creating inefficiencies of nutrient and/or oxygen delivery [45]. In the modulation of TME, multiple immune cell types and cancer cell changes contribute to the specificity of the treatment regimen. Therefore, immunometabolism provides an opportunity to identify new targets to improve cancer therapies [46]. Investigation to TME in multiple cancers revealed that STEAP1 was positively correlated with the major of immune infiltration, especially in BRCA, COAD, KIRP, PAAD, and THCA but negatively correlated with tumor-immune infiltration in PRAD, THYM, and UVM. This study identified that STEAP1 had relatively strong positive correlation to immune-infiltrating of CD8^+^ T cell in READ while had relatively strong negative correlations to immune-infiltrating of CD4^+^ T cell in THYM and UVM. Schirmer et al. found that STEAP1-specific T cell receptors were useful for STEAP1-expressing cancer types in Ewing sarcoma [47]. TISIDB database in THCA samples also showed that STEAP1 had the strongest correlation with MHC molecules, immune stimulators, and immune inhibitors such as CTLA-4, as well as chemokines [48]. In addition, we investigated the expression differences between six immune subtypes, finding that STEAP1 exerted increased expression in C6 which had the worst survival and decreased expression in C3 which had the optimal prognosis in several cancers such as COAD, LUSC, and STAD, indicating that STEAP1 served as a risk factor in these tumors. By conducting coexpressed strategy, STEAP2 had an obviously strong positive correlation with STEAP1.

TMB is an underlying biomarker in multiple cancers and is measured by the total amount of somatic coding mutations [49, 50]. Previous evidence validated high TMB is sensitive to immunotherapies and contributes to better survival [51, 52]. This study demonstrated STEAP1 expression is positively related to TMB in HNSC, KICH, LGG, LIHC, PAAD, PRAD, THYM, and UCEC. MSI, defined as a phenotype of alterations in microsatellite sequence caused by deficiency in DNA mismatch repair, is associated with increasing cancer predisposition [53, 54]. It has been regarded as a primary biomarker for the treatment with immune checkpoint blockade in the recent years [55]. In the current study, STEAP1 expression was positively correlated with MSI in COAD, KIRC, PRAD, and THCA whereas exhibited negative association in CESC.

CSCs also appear to have resistance to anticancer therapies and subsequent relapse [56]. Stemness indices are conducted by Malta et al. to predict the capability of tumor invasion and risk of recurrence based on DNA methylation and gene expression levels [57]. Here, STEAP1 is related to the stemness scores calculated by RNA and DNA in cancers such as LGG, LIHC, THCA, and THYM, which may participate in tumorigenesis and metastasis. However, further studies regarding the mechanism of STEAP1 mutation and stemness indices are still required.

By retrieving data from NCI-60 cell line, our study illustrated that increased STEAP1 expression level was positively related to increased chemosensitivity for several approved drugs by Food and Drug Administration, such as TAK-733, PD-0325901, trametinib, and BMS-777607. Meanwhile, increased STEAP1 expression was also correlated with increased drug resistance for some agents such as carboplatin, S-63845, arsenic trioxide, and cisplatin. These preliminary findings suggested that STEAP1 acted as an important role in chemosensitivity or resistance in cancer cells and served as a potential target to constrain drug resistance in different cancers.

However, there are some possible limitations of this research. Firstly, the database from TCGA in the study mainly includes Caucasian patients, and the data of other ethnicities has been a relative lack of research in this area. Moreover, further experimental studies in different cancers should be performed in the near future to identify STEAP1 as a key player in several types of cancers.

## 5. Conclusion

In summary, STEAP1 was dysregulated in pan-cancer tissues, and the aberrant expression of STEAP1 was more likely associated with clinicopathological features and predicted prognosis especially in adrenocortical carcinoma, breast cancer, glioma, renal cell carcinoma, lung cancer, prostate cancer, thyroid cancer, and endometrial carcinoma. Additionally, DNA methylation, TME, TMB, MSI, and cancer stemness might contribute to STEAP1 dysregulation in cancers, and STEAP1 may be a potential therapeutic target for immunotherapy.

## Data Availability

Data used in this study can be downloaded from TCGA (https://tcga-data.nci.nih.gov/tcga/), UCSC XENA (https://xenabrowser.net/datapages/), CellMiner (https://discover.nci.nih.gov/cellminer/home.do), and Harmonizome (https://maayanlab.cloud/Harmonizome/).

## Supplementary Materials

Supplementary 1 Table S1: the top-100 correlated genes with STEAP1.

Supplementary 2 Table S2: GO and KEGG analysis.

Supplementary 3 Table S3: GSEA analysis.

Supplementary 4 Table S4: TMB for 33 cancer types and STEAP1 expression.

Supplementary 5 Table S5: MSI for 33 cancer types and STEAP1 expression.

Supplementary 6 Table S6: associations between drug sensitivity and STEAP1 expression.

Supplementary 7 Figure S1: the differences of total protein expression of STEAP1 between normal and tumor samples in BRCA, COAD, LUAD, OV, and UCEC, respectively (a-e).

Supplementary 8 Figure S2: correlations of STEAP1 expression and the methylation of its promoter in BRCA, COAD, LUSC, PRAD, SKCM, and UCEC, respectively (a–f). The red dotted lines represented the fitted lines of correlation tests.

Supplementary 9 Figure S3: (a) a univariate Cox proportional hazard regression of DSS with STEAP1 expression was illustrated by a forest plot. (b) KM survival analysis of DSS between high- and low-expression groups of STEAP1.

Supplementary 10 Figure S4: (a) a univariate Cox proportional hazard regression of PFI with STEAP1 expression was illustrated by a forest plot. (b) KM survival analysis of PFI between high- and low-expression groups of STEAP1.

Supplementary 11 Figure S5: the differences of STEAP1 expression among distinctive immunophenotypes in multiple cancers (C1: wound healing; C2: IFN-gamma dominant; C3: inflammatory; C4: lymphocyte depleted; C5: immunologically quiet; C6: TGF-*β* dominant).

Supplementary 12 Figure S6: significant correlations of STEAP1 expression and stromal score were examined by ESTIMATE algorithm.
