# Supplementary material for: The Prognostic Value and Immunological Role of STEAP1 in Pan-Cancer: A Result of Data-Based Analysis
Source: Oxid Med Cell Longev. 2022 Mar 11;2022:8297011. doi: 10.1155/2022/8297011 (PMC8933652; doi:10.1155/2022/8297011)
Supplement: Supplementary 1 — Table S1: the top-100 correlated genes with STEAP1. [file 8297011.f1.doc]

| Rank | Gene | Coefficient |
| --- | --- | --- |
| 1 | GNG12 | 0.631631 |
| 2 | STEAP2 | 0.615428 |
| 3 | TMEM54 | 0.611707 |
| 4 | PLS3 | 0.611699 |
| 5 | PPIC | 0.611201 |
| 6 | CLDN12 | 0.607597 |
| 7 | SNX7 | 0.607073 |
| 8 | RAI14 | 0.604143 |
| 9 | LRIG3 | 0.598017 |
| 10 | ERRFI1 | 0.595931 |
| 11 | YAP1 | 0.588897 |
| 12 | KDELR2 | 0.588418 |
| 13 | SDC1 | 0.58692 |
| 14 | CYR61 | 0.586736 |
| 15 | CCND1 | 0.583219 |
| 16 | RND3 | 0.578027 |
| 17 | EGFR | 0.574248 |
| 18 | NR2F2 | 0.572296 |
| 19 | FAM114A1 | 0.57057 |
| 20 | GPX8 | 0.567428 |
| 21 | BCAR1 | 0.566882 |
| 22 | PERP | 0.566824 |
| 23 | RCN1 | 0.566456 |
| 24 | TEAD1 | 0.566308 |
| 25 | RHBDF1 | 0.565254 |
| 26 | SRPX2 | 0.564056 |
| 27 | MYO1B | 0.563761 |
| 28 | UGDH | 0.563303 |
| 29 | TEAD4 | 0.560592 |
| 30 | CD276 | 0.558903 |
| 31 | LAMC1 | 0.55807 |
| 32 | SLC39A14 | 0.556829 |
| 33 | SPR | 0.555947 |
| 34 | FERMT1 | 0.553729 |
| 35 | EI24 | 0.553517 |
| 36 | MYO1C | 0.553096 |
| 37 | MET | 0.552922 |
| 38 | DDAH1 | 0.550631 |
| 39 | 10-Sep | 0.550226 |
| 40 | KDELR3 | 0.549607 |
| 41 | UAP1 | 0.54748 |
| 42 | LAMA3 | 0.547176 |
| 43 | PTPRF | 0.544628 |
| 44 | TOM1L1 | 0.544143 |
| 45 | SH3D19 | 0.543781 |
| 46 | PTPN21 | 0.542639 |
| 47 | LIMA1 | 0.54262 |
| 48 | SRPX | 0.540847 |
| 49 | EPHA2 | 0.540769 |
| 50 | S100A16 | 0.539429 |
| 51 | FAT1 | 0.539305 |
| 52 | ZNF214 | 0.537334 |
| 53 | ADAM9 | 0.536155 |
| 54 | ALDH1A3 | 0.536129 |
| 55 | CTTN | 0.535226 |
| 56 | BAIAP2L1 | 0.533597 |
| 57 | SH2D4A | 0.533352 |
| 58 | FRMD6 | 0.533196 |
| 59 | NQO1 | 0.533185 |
| 60 | ARL1 | 0.532479 |
| 61 | KIAA1217 | 0.531841 |
| 62 | EPS8 | 0.531039 |
| 63 | CALU | 0.530435 |
| 64 | LAMB1 | 0.530331 |
| 65 | P4HA2 | 0.52906 |
| 66 | PIR | 0.528472 |
| 67 | AMOTL2 | 0.527894 |
| 68 | DSTN | 0.527772 |
| 69 | FKBP9 | 0.527442 |
| 70 | SPATS2L | 0.525422 |
| 71 | MID1 | 0.525351 |
| 72 | EVA1A | 0.525267 |
| 73 | TCEAL9 | 0.525148 |
| 74 | WWTR1 | 0.525147 |
| 75 | ZDHHC9 | 0.524622 |
| 76 | RAB13 | 0.524481 |
| 77 | GNPNAT1 | 0.524368 |
| 78 | TM4SF1 | 0.522408 |
| 79 | CADPS2 | 0.52205 |
| 80 | KITLG | 0.521453 |
| 81 | PACSIN3 | 0.521379 |
| 82 | CAV1 | 0.521127 |
| 83 | FAM57A | 0.521104 |
| 84 | THAP10 | 0.521042 |
| 85 | PARVA | 0.520997 |
| 86 | EPB41L4B | 0.52044 |
| 87 | TSPAN12 | 0.520397 |
| 88 | NCKAP1 | 0.519413 |
| 89 | SDC4 | 0.518799 |
| 90 | TBX3 | 0.518797 |
| 91 | FN1 | 0.518357 |
| 92 | ASPH | 0.518346 |
| 93 | PCGF2 | 0.517845 |
| 94 | TEAD3 | 0.517459 |
| 95 | HDLBP | 0.516407 |
| 96 | AJUBA | 0.514534 |
| 97 | KIRREL | 0.513413 |
| 98 | TJP1 | 0.513248 |
| 99 | LURAP1L | 0.512343 |
| 100 | LRRC8E | 0.511932 |
