# Supplementary material for: The Prognostic Value and Immunological Role of STEAP1 in Pan-Cancer: A Result of Data-Based Analysis
Source: Oxid Med Cell Longev. 2022 Mar 11;2022:8297011. doi: 10.1155/2022/8297011 (PMC8933652; doi:10.1155/2022/8297011)
Supplement: Supplementary 2 — Table S2: GO and KEGG analysis. [file 8297011.f2.doc]

| Type | ID | Description | GeneRatio | BgRatio | pvalue | p.adjust | qvalue | Count |
| --- | --- | --- | --- | --- | --- | --- | --- | --- |
| BP | GO:0035329 | hippo signaling | 8/93 | 38/18670 | 1.2062E-11 | 2.1941E-08 | 1.8487E-08 | 8 |
| BP | GO:0007015 | actin filament organization | 14/93 | 400/18670 | 1.0922E-08 | 9.934E-06 | 8.3701E-06 | 14 |
| BP | GO:0034330 | cell junction organization | 12/93 | 290/18670 | 2.1363E-08 | 1.2953E-05 | 1.0914E-05 | 12 |
| BP | GO:0034329 | cell junction assembly | 11/93 | 241/18670 | 3.1713E-08 | 1.4422E-05 | 1.2151E-05 | 11 |
| BP | GO:0001667 | ameboidal-type cell migration | 13/93 | 461/18670 | 4.6139E-07 | 0.00015202 | 0.00012809 | 13 |
| BP | GO:0032970 | regulation of actin filament-based process | 12/93 | 388/18670 | 5.0144E-07 | 0.00015202 | 0.00012809 | 12 |
| BP | GO:0051017 | actin filament bundle assembly | 8/93 | 153/18670 | 9.5666E-07 | 0.0002486 | 0.00020946 | 8 |
| BP | GO:0061572 | actin filament bundle organization | 8/93 | 157/18670 | 1.1627E-06 | 0.00026437 | 0.00022275 | 8 |
| BP | GO:0110053 | regulation of actin filament organization | 9/93 | 261/18670 | 6.1418E-06 | 0.00124132 | 0.0010459 | 9 |
| BP | GO:0032956 | regulation of actin cytoskeleton organization | 10/93 | 343/18670 | 8.0394E-06 | 0.00146236 | 0.00123214 | 10 |
| BP | GO:0007044 | cell-substrate junction assembly | 6/93 | 97/18670 | 8.9084E-06 | 0.00147313 | 0.00124121 | 6 |
| BP | GO:1902903 | regulation of supramolecular fiber organization | 10/93 | 352/18670 | 1.0076E-05 | 0.00152738 | 0.00128692 | 10 |
| BP | GO:0008064 | regulation of actin polymerization or depolymerization | 7/93 | 179/18670 | 3.1341E-05 | 0.00421978 | 0.00355545 | 7 |
| BP | GO:0030832 | regulation of actin filament length | 7/93 | 180/18670 | 3.2478E-05 | 0.00421978 | 0.00355545 | 7 |
| BP | GO:0051044 | positive regulation of membrane protein ectodomain proteolysis | 3/93 | 15/18670 | 5.2132E-05 | 0.00596588 | 0.00502666 | 3 |
| BP | GO:1901879 | regulation of protein depolymerization | 5/93 | 83/18670 | 5.8799E-05 | 0.00596588 | 0.00502666 | 5 |
| BP | GO:0050673 | epithelial cell proliferation | 10/93 | 434/18670 | 6.0058E-05 | 0.00596588 | 0.00502666 | 10 |
| BP | GO:0010631 | epithelial cell migration | 9/93 | 351/18670 | 6.3557E-05 | 0.00596588 | 0.00502666 | 9 |
| BP | GO:0031589 | cell-substrate adhesion | 9/93 | 354/18670 | 6.7845E-05 | 0.00596588 | 0.00502666 | 9 |
| BP | GO:0090132 | epithelium migration | 9/93 | 354/18670 | 6.7845E-05 | 0.00596588 | 0.00502666 | 9 |
| BP | GO:1902905 | positive regulation of supramolecular fiber organization | 7/93 | 204/18670 | 7.1821E-05 | 0.00596588 | 0.00502666 | 7 |
| BP | GO:0090130 | tissue migration | 9/93 | 360/18670 | 7.7157E-05 | 0.00596588 | 0.00502666 | 9 |
| BP | GO:1901888 | regulation of cell junction assembly | 5/93 | 88/18670 | 7.78E-05 | 0.00596588 | 0.00502666 | 5 |
| BP | GO:0002064 | epithelial cell development | 7/93 | 207/18670 | 7.8714E-05 | 0.00596588 | 0.00502666 | 7 |
| BP | GO:0008154 | actin polymerization or depolymerization | 7/93 | 209/18670 | 8.3603E-05 | 0.00608295 | 0.0051253 | 7 |
| BP | GO:0051592 | response to calcium ion | 6/93 | 148/18670 | 9.6915E-05 | 0.0067803 | 0.00571286 | 6 |
| BP | GO:0030857 | negative regulation of epithelial cell differentiation | 4/93 | 49/18670 | 0.00010297 | 0.00693701 | 0.0058449 | 4 |
| BP | GO:0051546 | keratinocyte migration | 3/93 | 19/18670 | 0.00010943 | 0.00710908 | 0.00598988 | 3 |
| BP | GO:0050999 | regulation of nitric-oxide synthase activity | 4/93 | 51/18670 | 0.00012053 | 0.00755998 | 0.0063698 | 4 |
| BP | GO:0030856 | regulation of epithelial cell differentiation | 6/93 | 156/18670 | 0.00012944 | 0.00759501 | 0.00639931 | 6 |
| BP | GO:0045216 | cell-cell junction organization | 6/93 | 156/18670 | 0.00012944 | 0.00759501 | 0.00639931 | 6 |
| BP | GO:0051495 | positive regulation of cytoskeleton organization | 7/93 | 226/18670 | 0.00013603 | 0.00773223 | 0.00651493 | 7 |
| BP | GO:2000810 | regulation of bicellular tight junction assembly | 3/93 | 21/18670 | 0.00014912 | 0.00799842 | 0.00673921 | 3 |
| BP | GO:0031532 | actin cytoskeleton reorganization | 5/93 | 101/18670 | 0.0001495 | 0.00799842 | 0.00673921 | 5 |
| BP | GO:0070830 | bicellular tight junction assembly | 4/93 | 55/18670 | 0.00016201 | 0.00841992 | 0.00709436 | 4 |
| BP | GO:0120192 | tight junction assembly | 4/93 | 56/18670 | 0.00017381 | 0.00878231 | 0.0073997 | 4 |
| BP | GO:0009226 | nucleotide-sugar biosynthetic process | 3/93 | 23/18670 | 0.00019713 | 0.00943651 | 0.00795091 | 3 |
| BP | GO:0051043 | regulation of membrane protein ectodomain proteolysis | 3/93 | 23/18670 | 0.00019713 | 0.00943651 | 0.00795091 | 3 |
| BP | GO:0051261 | protein depolymerization | 5/93 | 109/18670 | 0.00021372 | 0.00996815 | 0.00839885 | 5 |
| BP | GO:0120193 | tight junction organization | 4/93 | 60/18670 | 0.00022728 | 0.01033571 | 0.00870854 | 4 |
| BP | GO:0043244 | regulation of protein complex disassembly | 5/93 | 113/18670 | 0.0002528 | 0.01121551 | 0.00944983 | 5 |
| BP | GO:0048568 | embryonic organ development | 9/93 | 428/18670 | 0.00028231 | 0.01205842 | 0.01016004 | 9 |
| BP | GO:0032768 | regulation of monooxygenase activity | 4/93 | 64/18670 | 0.00029168 | 0.01205842 | 0.01016004 | 4 |
| BP | GO:0043297 | apical junction assembly | 4/93 | 64/18670 | 0.00029168 | 0.01205842 | 0.01016004 | 4 |
| BP | GO:0072089 | stem cell proliferation | 5/93 | 120/18670 | 0.00033398 | 0.01350005 | 0.01137471 | 5 |
| BP | GO:0006367 | transcription initiation from RNA polymerase II promoter | 6/93 | 188/18670 | 0.00035505 | 0.01403977 | 0.01182946 | 6 |
| BP | GO:0007043 | cell-cell junction assembly | 5/93 | 124/18670 | 0.00038845 | 0.01503381 | 0.01266701 | 5 |
| BP | GO:0072091 | regulation of stem cell proliferation | 4/93 | 70/18670 | 0.00041145 | 0.01559219 | 0.01313748 | 4 |
| BP | GO:0032535 | regulation of cellular component size | 8/93 | 370/18670 | 0.0005189 | 0.01926275 | 0.01623018 | 8 |
| BP | GO:0032273 | positive regulation of protein polymerization | 5/93 | 134/18670 | 0.00055397 | 0.02007246 | 0.01691242 | 5 |
| BP | GO:0007492 | endoderm development | 4/93 | 76/18670 | 0.00056278 | 0.02007246 | 0.01691242 | 4 |
| BP | GO:1901890 | positive regulation of cell junction assembly | 3/93 | 33/18670 | 0.00058582 | 0.02011449 | 0.01694783 | 3 |
| BP | GO:0006809 | nitric oxide biosynthetic process | 4/93 | 77/18670 | 0.00059137 | 0.02011449 | 0.01694783 | 4 |
| BP | GO:0050678 | regulation of epithelial cell proliferation | 8/93 | 378/18670 | 0.00059713 | 0.02011449 | 0.01694783 | 8 |
| BP | GO:0010632 | regulation of epithelial cell migration | 7/93 | 291/18670 | 0.00062796 | 0.02043032 | 0.01721394 | 7 |
| BP | GO:0030048 | actin filament-based movement | 5/93 | 138/18670 | 0.00063321 | 0.02043032 | 0.01721394 | 5 |
| BP | GO:0003382 | epithelial cell morphogenesis | 3/93 | 34/18670 | 0.0006402 | 0.02043032 | 0.01721394 | 3 |
| BP | GO:0048545 | response to steroid hormone | 8/93 | 385/18670 | 0.00067318 | 0.02111244 | 0.01778867 | 8 |
| BP | GO:0046209 | nitric oxide metabolic process | 4/93 | 82/18670 | 0.00074999 | 0.02273723 | 0.01915767 | 4 |
| BP | GO:0097581 | lamellipodium organization | 4/93 | 82/18670 | 0.00074999 | 0.02273723 | 0.01915767 | 4 |
| BP | GO:0032271 | regulation of protein polymerization | 6/93 | 218/18670 | 0.00077488 | 0.02310667 | 0.01946895 | 6 |
| BP | GO:0042542 | response to hydrogen peroxide | 5/93 | 146/18670 | 0.00081684 | 0.02358472 | 0.01987174 | 5 |
| BP | GO:0051384 | response to glucocorticoid | 5/93 | 146/18670 | 0.00081684 | 0.02358472 | 0.01987174 | 5 |
| BP | GO:2001057 | reactive nitrogen species metabolic process | 4/93 | 85/18670 | 0.00085842 | 0.02439786 | 0.02055686 | 4 |
| BP | GO:0009225 | nucleotide-sugar metabolic process | 3/93 | 38/18670 | 0.00088963 | 0.02489603 | 0.02097661 | 3 |
| BP | GO:0007160 | cell-matrix adhesion | 6/93 | 225/18670 | 0.00091303 | 0.02516367 | 0.02120211 | 6 |
| BP | GO:0000302 | response to reactive oxygen species | 6/93 | 232/18670 | 0.00106945 | 0.02903472 | 0.02446373 | 6 |
| BP | GO:0043588 | skin development | 8/93 | 419/18670 | 0.00116181 | 0.03107836 | 0.02618564 | 8 |
| BP | GO:0006509 | membrane protein ectodomain proteolysis | 3/93 | 42/18670 | 0.00119336 | 0.03145962 | 0.02650688 | 3 |
| BP | GO:0046661 | male sex differentiation | 5/93 | 160/18670 | 0.00123016 | 0.03176296 | 0.02676246 | 5 |
| BP | GO:0046677 | response to antibiotic | 7/93 | 327/18670 | 0.00123979 | 0.03176296 | 0.02676246 | 7 |
| BP | GO:0030833 | regulation of actin filament polymerization | 5/93 | 162/18670 | 0.00129987 | 0.03223034 | 0.02715626 | 5 |
| BP | GO:0031960 | response to corticosteroid | 5/93 | 162/18670 | 0.00129987 | 0.03223034 | 0.02715626 | 5 |
| BP | GO:0006048 | UDP-N-acetylglucosamine biosynthetic process | 2/93 | 11/18670 | 0.00131118 | 0.03223034 | 0.02715626 | 2 |
| BP | GO:0048732 | gland development | 8/93 | 434/18670 | 0.00145233 | 0.03401474 | 0.02865974 | 8 |
| BP | GO:0035987 | endodermal cell differentiation | 3/93 | 45/18670 | 0.00145925 | 0.03401474 | 0.02865974 | 3 |
| BP | GO:0030838 | positive regulation of actin filament polymerization | 4/93 | 99/18670 | 0.00151365 | 0.03401474 | 0.02865974 | 4 |
| BP | GO:0006352 | DNA-templated transcription, initiation | 6/93 | 249/18670 | 0.00153511 | 0.03401474 | 0.02865974 | 6 |
| BP | GO:0033628 | regulation of cell adhesion mediated by integrin | 3/93 | 46/18670 | 0.00155546 | 0.03401474 | 0.02865974 | 3 |
| BP | GO:0060443 | mammary gland morphogenesis | 3/93 | 46/18670 | 0.00155546 | 0.03401474 | 0.02865974 | 3 |
| BP | GO:0031581 | hemidesmosome assembly | 2/93 | 12/18670 | 0.00156833 | 0.03401474 | 0.02865974 | 2 |
| BP | GO:0035437 | maintenance of protein localization in endoplasmic reticulum | 2/93 | 12/18670 | 0.00156833 | 0.03401474 | 0.02865974 | 2 |
| BP | GO:0051549 | positive regulation of keratinocyte migration | 2/93 | 12/18670 | 0.00156833 | 0.03401474 | 0.02865974 | 2 |
| BP | GO:0034446 | substrate adhesion-dependent cell spreading | 4/93 | 100/18670 | 0.00157077 | 0.03401474 | 0.02865974 | 4 |
| BP | GO:0010634 | positive regulation of epithelial cell migration | 5/93 | 171/18670 | 0.00165022 | 0.03501699 | 0.02950421 | 5 |
| BP | GO:0031952 | regulation of protein autophosphorylation | 3/93 | 47/18670 | 0.00165556 | 0.03501699 | 0.02950421 | 3 |
| BP | GO:0007229 | integrin-mediated signaling pathway | 4/93 | 103/18670 | 0.00175114 | 0.03661283 | 0.0308488 | 4 |
| BP | GO:0031953 | negative regulation of protein autophosphorylation | 2/93 | 13/18670 | 0.00184747 | 0.03733952 | 0.03146109 | 2 |
| BP | GO:0046349 | amino sugar biosynthetic process | 2/93 | 13/18670 | 0.00184747 | 0.03733952 | 0.03146109 | 2 |
| BP | GO:0072182 | regulation of nephron tubule epithelial cell differentiation | 2/93 | 13/18670 | 0.00184747 | 0.03733952 | 0.03146109 | 2 |
| BP | GO:0042136 | neurotransmitter biosynthetic process | 4/93 | 106/18670 | 0.0019454 | 0.03888663 | 0.03276464 | 4 |
| BP | GO:0001706 | endoderm formation | 3/93 | 50/18670 | 0.00197974 | 0.03914284 | 0.03298051 | 3 |
| BP | GO:0051341 | regulation of oxidoreductase activity | 4/93 | 107/18670 | 0.00201334 | 0.03925332 | 0.0330736 | 4 |
| BP | GO:0030834 | regulation of actin filament depolymerization | 3/93 | 51/18670 | 0.00209594 | 0.03925332 | 0.0330736 | 3 |
| BP | GO:0051547 | regulation of keratinocyte migration | 2/93 | 14/18670 | 0.00214841 | 0.03925332 | 0.0330736 | 2 |
| BP | GO:0060009 | Sertoli cell development | 2/93 | 14/18670 | 0.00214841 | 0.03925332 | 0.0330736 | 2 |
| BP | GO:0097202 | activation of cysteine-type endopeptidase activity | 2/93 | 14/18670 | 0.00214841 | 0.03925332 | 0.0330736 | 2 |
| BP | GO:0030041 | actin filament polymerization | 5/93 | 182/18670 | 0.00216731 | 0.03925332 | 0.0330736 | 5 |
| BP | GO:0043687 | post-translational protein modification | 7/93 | 361/18670 | 0.00217578 | 0.03925332 | 0.0330736 | 7 |
| BP | GO:0008544 | epidermis development | 8/93 | 464/18670 | 0.00220586 | 0.03925332 | 0.0330736 | 8 |
| BP | GO:0031529 | ruffle organization | 3/93 | 52/18670 | 0.0022163 | 0.03925332 | 0.0330736 | 3 |
| BP | GO:0031334 | positive regulation of protein complex assembly | 6/93 | 268/18670 | 0.00222328 | 0.03925332 | 0.0330736 | 6 |
| BP | GO:0016101 | diterpenoid metabolic process | 4/93 | 110/18670 | 0.00222698 | 0.03925332 | 0.0330736 | 4 |
| BP | GO:0045862 | positive regulation of proteolysis | 7/93 | 363/18670 | 0.00224428 | 0.03925332 | 0.0330736 | 7 |
| BP | GO:0010038 | response to metal ion | 7/93 | 364/18670 | 0.00227915 | 0.03948357 | 0.0332676 | 7 |
| BP | GO:0007369 | gastrulation | 5/93 | 185/18670 | 0.0023268 | 0.03977518 | 0.0335133 | 5 |
| BP | GO:0048593 | camera-type eye morphogenesis | 4/93 | 112/18670 | 0.00237779 | 0.03977518 | 0.0335133 | 4 |
| BP | GO:0043542 | endothelial cell migration | 6/93 | 273/18670 | 0.00243798 | 0.03977518 | 0.0335133 | 6 |
| BP | GO:0030042 | actin filament depolymerization | 3/93 | 54/18670 | 0.00246969 | 0.03977518 | 0.0335133 | 3 |
| BP | GO:0035635 | entry of bacterium into host cell | 2/93 | 15/18670 | 0.00247092 | 0.03977518 | 0.0335133 | 2 |
| BP | GO:0072160 | nephron tubule epithelial cell differentiation | 2/93 | 15/18670 | 0.00247092 | 0.03977518 | 0.0335133 | 2 |
| BP | GO:1900037 | regulation of cellular response to hypoxia | 2/93 | 15/18670 | 0.00247092 | 0.03977518 | 0.0335133 | 2 |
| BP | GO:1903543 | positive regulation of exosomal secretion | 2/93 | 15/18670 | 0.00247092 | 0.03977518 | 0.0335133 | 2 |
| BP | GO:1901654 | response to ketone | 5/93 | 193/18670 | 0.00279379 | 0.04376126 | 0.03687185 | 5 |
| BP | GO:0006047 | UDP-N-acetylglucosamine metabolic process | 2/93 | 16/18670 | 0.00281477 | 0.04376126 | 0.03687185 | 2 |
| BP | GO:0048012 | hepatocyte growth factor receptor signaling pathway | 2/93 | 16/18670 | 0.00281477 | 0.04376126 | 0.03687185 | 2 |
| BP | GO:1903541 | regulation of exosomal secretion | 2/93 | 16/18670 | 0.00281477 | 0.04376126 | 0.03687185 | 2 |
| BP | GO:0018208 | peptidyl-proline modification | 3/93 | 57/18670 | 0.00288221 | 0.04443004 | 0.03743534 | 3 |
| BP | GO:0051258 | protein polymerization | 6/93 | 283/18670 | 0.00291386 | 0.04454045 | 0.03752836 | 6 |
| BP | GO:0007173 | epidermal growth factor receptor signaling pathway | 4/93 | 119/18670 | 0.00296093 | 0.04488282 | 0.03781684 | 4 |
| BP | GO:0006721 | terpenoid metabolic process | 4/93 | 120/18670 | 0.00305152 | 0.04549764 | 0.03833486 | 4 |
| BP | GO:0022612 | gland morphogenesis | 4/93 | 120/18670 | 0.00305152 | 0.04549764 | 0.03833486 | 4 |
| BP | GO:0001704 | formation of primary germ layer | 4/93 | 121/18670 | 0.00314398 | 0.04626652 | 0.0389827 | 4 |
| BP | GO:0048562 | embryonic organ morphogenesis | 6/93 | 288/18670 | 0.0031764 | 0.04626652 | 0.0389827 | 6 |
| BP | GO:0033619 | membrane protein proteolysis | 3/93 | 59/18670 | 0.00317939 | 0.04626652 | 0.0389827 | 3 |
| BP | GO:1903409 | reactive oxygen species biosynthetic process | 4/93 | 122/18670 | 0.00323834 | 0.04675028 | 0.0393903 | 4 |
| BP | GO:0034755 | iron ion transmembrane transport | 2/93 | 19/18670 | 0.00397229 | 0.05520697 | 0.04651563 | 2 |
| BP | GO:1990182 | exosomal secretion | 2/93 | 19/18670 | 0.00397229 | 0.05520697 | 0.04651563 | 2 |
| BP | GO:2000696 | regulation of epithelial cell differentiation involved in kidney development | 2/93 | 19/18670 | 0.00397229 | 0.05520697 | 0.04651563 | 2 |
| BP | GO:0045785 | positive regulation of cell adhesion | 7/93 | 403/18670 | 0.00399908 | 0.05520697 | 0.04651563 | 7 |
| BP | GO:0030032 | lamellipodium assembly | 3/93 | 64/18670 | 0.00400248 | 0.05520697 | 0.04651563 | 3 |
| BP | GO:0007163 | establishment or maintenance of cell polarity | 5/93 | 210/18670 | 0.00400622 | 0.05520697 | 0.04651563 | 5 |
| BP | GO:1901880 | negative regulation of protein depolymerization | 3/93 | 66/18670 | 0.00436464 | 0.05716263 | 0.04816342 | 3 |
| BP | GO:0030903 | notochord development | 2/93 | 20/18670 | 0.0043994 | 0.05716263 | 0.04816342 | 2 |
| BP | GO:0033630 | positive regulation of cell adhesion mediated by integrin | 2/93 | 20/18670 | 0.0043994 | 0.05716263 | 0.04816342 | 2 |
| BP | GO:0060008 | Sertoli cell differentiation | 2/93 | 20/18670 | 0.0043994 | 0.05716263 | 0.04816342 | 2 |
| BP | GO:0072234 | metanephric nephron tubule development | 2/93 | 20/18670 | 0.0043994 | 0.05716263 | 0.04816342 | 2 |
| BP | GO:0097734 | extracellular exosome biogenesis | 2/93 | 20/18670 | 0.0043994 | 0.05716263 | 0.04816342 | 2 |
| BP | GO:0032869 | cellular response to insulin stimulus | 5/93 | 216/18670 | 0.00451233 | 0.05716263 | 0.04816342 | 5 |
| BP | GO:0030260 | entry into host cell | 4/93 | 134/18670 | 0.00452524 | 0.05716263 | 0.04816342 | 4 |
| BP | GO:0032355 | response to estradiol | 4/93 | 134/18670 | 0.00452524 | 0.05716263 | 0.04816342 | 4 |
| BP | GO:0044409 | entry into host | 4/93 | 134/18670 | 0.00452524 | 0.05716263 | 0.04816342 | 4 |
| BP | GO:0051806 | entry into cell of other organism involved in symbiotic interaction | 4/93 | 134/18670 | 0.00452524 | 0.05716263 | 0.04816342 | 4 |
| BP | GO:0051828 | entry into other organism involved in symbiotic interaction | 4/93 | 134/18670 | 0.00452524 | 0.05716263 | 0.04816342 | 4 |
| BP | GO:0043624 | cellular protein complex disassembly | 5/93 | 217/18670 | 0.00460091 | 0.0577176 | 0.04863101 | 5 |
| BP | GO:0001889 | liver development | 4/93 | 135/18670 | 0.00464592 | 0.0578831 | 0.04877046 | 4 |
| BP | GO:0033627 | cell adhesion mediated by integrin | 3/93 | 68/18670 | 0.00474606 | 0.05872846 | 0.04948273 | 3 |
| BP | GO:0008584 | male gonad development | 4/93 | 138/18670 | 0.00502093 | 0.06129574 | 0.05164584 | 4 |
| BP | GO:0061008 | hepaticobiliary system development | 4/93 | 138/18670 | 0.00502093 | 0.06129574 | 0.05164584 | 4 |
| BP | GO:0043062 | extracellular structure organization | 7/93 | 422/18670 | 0.00512987 | 0.06163428 | 0.05193109 | 7 |
| BP | GO:0006720 | isoprenoid metabolic process | 4/93 | 139/18670 | 0.00515031 | 0.06163428 | 0.05193109 | 4 |
| BP | GO:0046546 | development of primary male sexual characteristics | 4/93 | 139/18670 | 0.00515031 | 0.06163428 | 0.05193109 | 4 |
| BP | GO:0097191 | extrinsic apoptotic signaling pathway | 5/93 | 224/18670 | 0.00525601 | 0.06236554 | 0.05254722 | 5 |
| BP | GO:0072073 | kidney epithelium development | 4/93 | 140/18670 | 0.00528191 | 0.06236554 | 0.05254722 | 4 |
| BP | GO:0140112 | extracellular vesicle biogenesis | 2/93 | 22/18670 | 0.00531427 | 0.06236554 | 0.05254722 | 2 |
| BP | GO:0071375 | cellular response to peptide hormone stimulus | 6/93 | 321/18670 | 0.00537572 | 0.06268226 | 0.05281408 | 6 |
| BP | GO:0038127 | ERBB signaling pathway | 4/93 | 142/18670 | 0.00555184 | 0.06357314 | 0.05356471 | 4 |
| BP | GO:1902904 | negative regulation of supramolecular fiber organization | 4/93 | 142/18670 | 0.00555184 | 0.06357314 | 0.05356471 | 4 |
| BP | GO:0061180 | mammary gland epithelium development | 3/93 | 72/18670 | 0.00556786 | 0.06357314 | 0.05356471 | 3 |
| BP | GO:0030879 | mammary gland development | 4/93 | 143/18670 | 0.00569019 | 0.06357314 | 0.05356471 | 4 |
| BP | GO:0048608 | reproductive structure development | 7/93 | 431/18670 | 0.00574282 | 0.06357314 | 0.05356471 | 7 |
| BP | GO:0010594 | regulation of endothelial cell migration | 5/93 | 229/18670 | 0.0057627 | 0.06357314 | 0.05356471 | 5 |
| BP | GO:0072170 | metanephric tubule development | 2/93 | 23/18670 | 0.00580162 | 0.06357314 | 0.05356471 | 2 |
| BP | GO:0072215 | regulation of metanephros development | 2/93 | 23/18670 | 0.00580162 | 0.06357314 | 0.05356471 | 2 |
| BP | GO:0072243 | metanephric nephron epithelium development | 2/93 | 23/18670 | 0.00580162 | 0.06357314 | 0.05356471 | 2 |
| BP | GO:2000637 | positive regulation of gene silencing by miRNA | 2/93 | 23/18670 | 0.00580162 | 0.06357314 | 0.05356471 | 2 |
| BP | GO:0061458 | reproductive system development | 7/93 | 434/18670 | 0.00595888 | 0.06490541 | 0.05468723 | 7 |
| BP | GO:0032507 | maintenance of protein location in cell | 3/93 | 74/18670 | 0.00600877 | 0.06505922 | 0.05481683 | 3 |
| BP | GO:0032984 | protein-containing complex disassembly | 6/93 | 329/18670 | 0.00604499 | 0.06506414 | 0.05482098 | 6 |
| BP | GO:0043242 | negative regulation of protein complex disassembly | 3/93 | 75/18670 | 0.00623683 | 0.06671742 | 0.05621398 | 3 |
| BP | GO:0018126 | protein hydroxylation | 2/93 | 24/18670 | 0.00630863 | 0.06671742 | 0.05621398 | 2 |
| BP | GO:0060148 | positive regulation of posttranscriptional gene silencing | 2/93 | 24/18670 | 0.00630863 | 0.06671742 | 0.05621398 | 2 |
| BP | GO:0051494 | negative regulation of cytoskeleton organization | 4/93 | 148/18670 | 0.00641661 | 0.06746709 | 0.05684563 | 4 |
| BP | GO:0048592 | eye morphogenesis | 4/93 | 149/18670 | 0.00656893 | 0.06845156 | 0.05767511 | 4 |
| BP | GO:0060070 | canonical Wnt signaling pathway | 6/93 | 335/18670 | 0.0065855 | 0.06845156 | 0.05767511 | 6 |
| BP | GO:0008360 | regulation of cell shape | 4/93 | 150/18670 | 0.00672363 | 0.06907252 | 0.05819831 | 4 |
| BP | GO:0048754 | branching morphogenesis of an epithelial tube | 4/93 | 150/18670 | 0.00672363 | 0.06907252 | 0.05819831 | 4 |
| BP | GO:0001945 | lymph vessel development | 2/93 | 25/18670 | 0.0068351 | 0.06907252 | 0.05819831 | 2 |
| BP | GO:0060444 | branching involved in mammary gland duct morphogenesis | 2/93 | 25/18670 | 0.0068351 | 0.06907252 | 0.05819831 | 2 |
| BP | GO:0072202 | cell differentiation involved in metanephros development | 2/93 | 25/18670 | 0.0068351 | 0.06907252 | 0.05819831 | 2 |
| BP | GO:0001570 | vasculogenesis | 3/93 | 79/18670 | 0.00720052 | 0.07198229 | 0.06064999 | 3 |
| BP | GO:0042133 | neurotransmitter metabolic process | 4/93 | 153/18670 | 0.00720219 | 0.07198229 | 0.06064999 | 4 |
| BP | GO:0006979 | response to oxidative stress | 7/93 | 451/18670 | 0.00730037 | 0.07256487 | 0.06114085 | 7 |
| BP | GO:0071711 | basement membrane organization | 2/93 | 26/18670 | 0.00738084 | 0.07296599 | 0.06147883 | 2 |
| BP | GO:2001236 | regulation of extrinsic apoptotic signaling pathway | 4/93 | 155/18670 | 0.00753343 | 0.07407189 | 0.06241062 | 4 |
| BP | GO:0007045 | cell-substrate adherens junction assembly | 3/93 | 81/18670 | 0.00771361 | 0.07503236 | 0.06321988 | 3 |
| BP | GO:0048041 | focal adhesion assembly | 3/93 | 81/18670 | 0.00771361 | 0.07503236 | 0.06321988 | 3 |
| BP | GO:0072207 | metanephric epithelium development | 2/93 | 27/18670 | 0.00794563 | 0.0768782 | 0.06477512 | 2 |
| BP | GO:0006970 | response to osmotic stress | 3/93 | 83/18670 | 0.00824779 | 0.07937951 | 0.06688265 | 3 |
| BP | GO:0033598 | mammary gland epithelial cell proliferation | 2/93 | 28/18670 | 0.00852928 | 0.08165666 | 0.06880131 | 2 |
| BP | GO:0043254 | regulation of protein complex assembly | 7/93 | 467/18670 | 0.00875694 | 0.08339726 | 0.07026788 | 7 |
| BP | GO:0042058 | regulation of epidermal growth factor receptor signaling pathway | 3/93 | 86/18670 | 0.00908903 | 0.08431663 | 0.07104251 | 3 |
| BP | GO:0090596 | sensory organ morphogenesis | 5/93 | 256/18670 | 0.00910752 | 0.08431663 | 0.07104251 | 5 |
| BP | GO:0006929 | substrate-dependent cell migration | 2/93 | 29/18670 | 0.0091316 | 0.08431663 | 0.07104251 | 2 |
| BP | GO:0007263 | nitric oxide mediated signal transduction | 2/93 | 29/18670 | 0.0091316 | 0.08431663 | 0.07104251 | 2 |
| BP | GO:0061099 | negative regulation of protein tyrosine kinase activity | 2/93 | 29/18670 | 0.0091316 | 0.08431663 | 0.07104251 | 2 |
| BP | GO:1900027 | regulation of ruffle assembly | 2/93 | 29/18670 | 0.0091316 | 0.08431663 | 0.07104251 | 2 |
| BP | GO:0033673 | negative regulation of kinase activity | 5/93 | 257/18670 | 0.00925248 | 0.08500129 | 0.07161939 | 5 |
| BP | GO:0018108 | peptidyl-tyrosine phosphorylation | 6/93 | 363/18670 | 0.009582 | 0.08758621 | 0.07379736 | 6 |
| BP | GO:0045921 | positive regulation of exocytosis | 3/93 | 88/18670 | 0.00967675 | 0.08781969 | 0.07399408 | 3 |
| BP | GO:0071549 | cellular response to dexamethasone stimulus | 2/93 | 30/18670 | 0.00975238 | 0.08781969 | 0.07399408 | 2 |
| BP | GO:0097421 | liver regeneration | 2/93 | 30/18670 | 0.00975238 | 0.08781969 | 0.07399408 | 2 |
| BP | GO:0034614 | cellular response to reactive oxygen species | 4/93 | 168/18670 | 0.00993301 | 0.0887434 | 0.07477237 | 4 |
| BP | GO:0018212 | peptidyl-tyrosine modification | 6/93 | 366/18670 | 0.00995253 | 0.0887434 | 0.07477237 | 6 |
| BP | GO:0034333 | adherens junction assembly | 3/93 | 90/18670 | 0.01028619 | 0.09127111 | 0.07690213 | 3 |
| BP | GO:0035767 | endothelial cell chemotaxis | 2/93 | 31/18670 | 0.01039143 | 0.09131407 | 0.07693833 | 2 |
| BP | GO:0045737 | positive regulation of cyclin-dependent protein serine/threonine kinase activity | 2/93 | 31/18670 | 0.01039143 | 0.09131407 | 0.07693833 | 2 |
| BP | GO:0045907 | positive regulation of vasoconstriction | 2/93 | 32/18670 | 0.01104856 | 0.09662179 | 0.08141045 | 2 |
| BP | GO:1901184 | regulation of ERBB signaling pathway | 3/93 | 93/18670 | 0.01124136 | 0.09774506 | 0.08235688 | 3 |
| BP | GO:0007548 | sex differentiation | 5/93 | 270/18670 | 0.01128448 | 0.09774506 | 0.08235688 | 5 |
| BP | GO:0120032 | regulation of plasma membrane bounded cell projection assembly | 4/93 | 175/18670 | 0.01140927 | 0.09835761 | 0.082873 | 4 |
| BP | GO:0032868 | response to insulin | 5/93 | 272/18670 | 0.01162205 | 0.0996504 | 0.08396226 | 5 |
| BP | GO:0010591 | regulation of lamellipodium assembly | 2/93 | 33/18670 | 0.01172358 | 0.0996504 | 0.08396226 | 2 |
| BP | GO:0060603 | mammary gland duct morphogenesis | 2/93 | 33/18670 | 0.01172358 | 0.0996504 | 0.08396226 | 2 |
| BP | GO:0060491 | regulation of cell projection assembly | 4/93 | 177/18670 | 0.0118556 | 0.09979477 | 0.0840839 | 4 |
| BP | GO:0051651 | maintenance of location in cell | 3/93 | 95/18670 | 0.01190569 | 0.09979477 | 0.0840839 | 3 |
| BP | GO:0030900 | forebrain development | 6/93 | 381/18670 | 0.01196001 | 0.09979477 | 0.0840839 | 6 |
| BP | GO:0042176 | regulation of protein catabolic process | 6/93 | 381/18670 | 0.01196001 | 0.09979477 | 0.0840839 | 6 |
| CC | GO:0031252 | cell leading edge | 14/94 | 403/19717 | 7.0055E-09 | 4.5174E-07 | 3.609E-07 | 14 |
| CC | GO:0005925 | focal adhesion | 14/94 | 405/19717 | 7.4594E-09 | 4.5174E-07 | 3.609E-07 | 14 |
| CC | GO:0005924 | cell-substrate adherens junction | 14/94 | 408/19717 | 8.1904E-09 | 4.5174E-07 | 3.609E-07 | 14 |
| CC | GO:0030055 | cell-substrate junction | 14/94 | 412/19717 | 9.2664E-09 | 4.5174E-07 | 3.609E-07 | 14 |
| CC | GO:0030027 | lamellipodium | 10/94 | 193/19717 | 2.8347E-08 | 1.1055E-06 | 8.8322E-07 | 10 |
| CC | GO:0001726 | ruffle | 8/94 | 172/19717 | 1.6759E-06 | 5.1911E-05 | 4.1473E-05 | 8 |
| CC | GO:0005911 | cell-cell junction | 12/94 | 459/19717 | 1.8635E-06 | 5.1911E-05 | 4.1473E-05 | 12 |
| CC | GO:0070160 | tight junction | 7/94 | 128/19717 | 2.6397E-06 | 6.4343E-05 | 5.1405E-05 | 7 |
| CC | GO:0043296 | apical junction complex | 7/94 | 143/19717 | 5.5083E-06 | 0.00011935 | 9.5348E-05 | 7 |
| CC | GO:0043256 | laminin complex | 3/94 | 10/19717 | 1.2291E-05 | 0.00023967 | 0.00019148 | 3 |
| CC | GO:0005884 | actin filament | 6/94 | 111/19717 | 1.5128E-05 | 0.00026818 | 0.00021426 | 6 |
| CC | GO:0005923 | bicellular tight junction | 6/94 | 123/19717 | 2.7121E-05 | 0.00044071 | 0.0003521 | 6 |
| CC | GO:0005788 | endoplasmic reticulum lumen | 8/94 | 309/19717 | 0.00011507 | 0.00172606 | 0.00137899 | 8 |
| CC | GO:0009925 | basal plasma membrane | 3/94 | 34/19717 | 0.00056413 | 0.00785752 | 0.00627753 | 3 |
| CC | GO:0005938 | cell cortex | 7/94 | 308/19717 | 0.00068006 | 0.00884078 | 0.00706308 | 7 |
| CC | GO:0032587 | ruffle membrane | 4/94 | 94/19717 | 0.00106451 | 0.0127012 | 0.01014725 | 4 |
| CC | GO:0005604 | basement membrane | 4/94 | 95/19717 | 0.00110728 | 0.0127012 | 0.01014725 | 4 |
| CC | GO:0005903 | brush border | 4/94 | 99/19717 | 0.00129049 | 0.01363644 | 0.01089443 | 4 |
| CC | GO:0031256 | leading edge membrane | 5/94 | 170/19717 | 0.00132868 | 0.01363644 | 0.01089443 | 5 |
| CC | GO:0044420 | extracellular matrix component | 3/94 | 51/19717 | 0.00185167 | 0.01719408 | 0.0137367 | 3 |
| CC | GO:0045178 | basal part of cell | 3/94 | 51/19717 | 0.00185167 | 0.01719408 | 0.0137367 | 3 |
| CC | GO:0045177 | apical part of cell | 7/94 | 384/19717 | 0.0024119 | 0.02137817 | 0.01707945 | 7 |
| CC | GO:0016328 | lateral plasma membrane | 3/94 | 57/19717 | 0.00254862 | 0.0216079 | 0.017263 | 3 |
| CC | GO:0098858 | actin-based cell projection | 5/94 | 208/19717 | 0.00319797 | 0.02598353 | 0.02075878 | 5 |
| CC | GO:0016323 | basolateral plasma membrane | 5/94 | 217/19717 | 0.0038303 | 0.0298763 | 0.02386879 | 5 |
| CC | GO:0030663 | COPI-coated vesicle membrane | 2/94 | 20/19717 | 0.00404036 | 0.03030274 | 0.02420947 | 2 |
| CC | GO:0031258 | lamellipodium membrane | 2/94 | 22/19717 | 0.00488192 | 0.03525829 | 0.02816856 | 2 |
| CC | GO:0098862 | cluster of actin-based cell projections | 4/94 | 150/19717 | 0.00577867 | 0.0402443 | 0.03215199 | 4 |
| CC | GO:0031253 | cell projection membrane | 6/94 | 345/19717 | 0.00616207 | 0.04143459 | 0.03310294 | 6 |
| CC | GO:0030137 | COPI-coated vesicle | 2/94 | 27/19717 | 0.0073042 | 0.04747728 | 0.03793057 | 2 |
| CC | GO:0010008 | endosome membrane | 7/94 | 479/19717 | 0.00797009 | 0.04895232 | 0.03910901 | 7 |
| CC | GO:0005667 | transcription factor complex | 6/94 | 365/19717 | 0.0080332 | 0.04895232 | 0.03910901 | 6 |
| CC | GO:0002102 | podosome | 2/94 | 30/19717 | 0.00896877 | 0.05299725 | 0.04234059 | 2 |
| CC | GO:0031941 | filamentous actin | 2/94 | 32/19717 | 0.01016357 | 0.05829106 | 0.04656992 | 2 |
| CC | GO:0062023 | collagen-containing extracellular matrix | 6/94 | 406/19717 | 0.01309659 | 0.07296674 | 0.05829462 | 6 |
| CC | GO:0030175 | filopodium | 3/94 | 104/19717 | 0.01350995 | 0.07317888 | 0.0584641 | 3 |
| MF | GO:0050839 | cell adhesion molecule binding | 14/90 | 499/17697 | 2.1267E-07 | 4.4447E-05 | 3.8952E-05 | 14 |
| MF | GO:0051015 | actin filament binding | 9/90 | 198/17697 | 7.4026E-07 | 7.7357E-05 | 6.7792E-05 | 9 |
| MF | GO:0003779 | actin binding | 12/90 | 431/17697 | 1.8392E-06 | 0.00012813 | 0.00011229 | 12 |
| MF | GO:0045296 | cadherin binding | 9/90 | 331/17697 | 4.6932E-05 | 0.00245219 | 0.00214899 | 9 |
| MF | GO:0070064 | proline-rich region binding | 3/90 | 18/17697 | 9.8208E-05 | 0.00410509 | 0.00359751 | 3 |
| MF | GO:0005201 | extracellular matrix structural constituent | 6/90 | 163/17697 | 0.0001831 | 0.00637811 | 0.00558948 | 6 |
| MF | GO:0005178 | integrin binding | 5/90 | 132/17697 | 0.00056626 | 0.01690693 | 0.01481645 | 5 |
| MF | GO:0030898 | actin-dependent ATPase activity | 2/90 | 12/17697 | 0.00163306 | 0.04266381 | 0.03738858 | 2 |
| MF | GO:0004714 | transmembrane receptor protein tyrosine kinase activity | 3/90 | 62/17697 | 0.00387359 | 0.08995333 | 0.07883092 | 3 |
| MF | GO:0017124 | SH3 domain binding | 4/90 | 130/17697 | 0.00436474 | 0.09122304 | 0.07994363 | 4 |
| MF | GO:0048365 | Rac GTPase binding | 3/90 | 69/17697 | 0.00523104 | 0.09631469 | 0.08440572 | 3 |
| MF | GO:0000146 | microfilament motor activity | 2/90 | 22/17697 | 0.00553003 | 0.09631469 | 0.08440572 | 2 |
| KEGG | hsa04392 | Hippo signaling pathway - multiple species | 7/52 | 29/8076 | 4.2284E-10 | 4.9896E-08 | 3.9614E-08 | 7 |
| KEGG | hsa04510 | Focal adhesion | 10/52 | 201/8076 | 4.6401E-07 | 2.7377E-05 | 2.1735E-05 | 10 |
| KEGG | hsa04390 | Hippo signaling pathway | 8/52 | 157/8076 | 6.2141E-06 | 0.00024442 | 0.00019406 | 8 |
| KEGG | hsa04512 | ECM-receptor interaction | 6/52 | 88/8076 | 1.9196E-05 | 0.00056628 | 0.00044959 | 6 |
| KEGG | hsa05205 | Proteoglycans in cancer | 8/52 | 205/8076 | 4.333E-05 | 0.00102259 | 0.00081187 | 8 |
| KEGG | hsa04151 | PI3K-Akt signaling pathway | 10/52 | 354/8076 | 7.023E-05 | 0.00138119 | 0.00109658 | 10 |
| KEGG | hsa05100 | Bacterial invasion of epithelial cells | 5/52 | 77/8076 | 0.00012658 | 0.0021338 | 0.00169409 | 5 |
| KEGG | hsa05222 | Small cell lung cancer | 5/52 | 92/8076 | 0.00029296 | 0.00432114 | 0.0034307 | 5 |
| KEGG | hsa04530 | Tight junction | 6/52 | 169/8076 | 0.00070477 | 0.00924026 | 0.00733616 | 6 |
| KEGG | hsa04520 | Adherens junction | 4/52 | 71/8076 | 0.00108018 | 0.01274614 | 0.01011959 | 4 |
| KEGG | hsa05130 | Pathogenic Escherichia coli infection | 6/52 | 197/8076 | 0.00156179 | 0.01675372 | 0.01330134 | 6 |
| KEGG | hsa00520 | Amino sugar and nucleotide sugar metabolism | 3/52 | 48/8076 | 0.00355067 | 0.03439257 | 0.02730543 | 3 |
| KEGG | hsa05110 | Vibrio cholerae infection | 3/52 | 50/8076 | 0.00398747 | 0.03439257 | 0.02730543 | 3 |
| KEGG | hsa05146 | Amoebiasis | 4/52 | 102/8076 | 0.00408047 | 0.03439257 | 0.02730543 | 4 |
| KEGG | hsa05120 | Epithelial cell signaling in Helicobacter pylori infection | 3/52 | 70/8076 | 0.01017667 | 0.07084951 | 0.05624983 | 3 |
| KEGG | hsa04015 | Rap1 signaling pathway | 5/52 | 210/8076 | 0.01092982 | 0.07084951 | 0.05624983 | 5 |
| KEGG | hsa05218 | Melanoma | 3/52 | 72/8076 | 0.01098841 | 0.07084951 | 0.05624983 | 3 |
| KEGG | hsa05223 | Non-small cell lung cancer | 3/52 | 72/8076 | 0.01098841 | 0.07084951 | 0.05624983 | 3 |
| KEGG | hsa04115 | p53 signaling pathway | 3/52 | 73/8076 | 0.01140797 | 0.07084951 | 0.05624983 | 3 |
| KEGG | hsa05418 | Fluid shear stress and atherosclerosis | 4/52 | 139/8076 | 0.01201417 | 0.07088358 | 0.05627688 | 4 |
| KEGG | hsa04810 | Regulation of actin cytoskeleton | 5/52 | 218/8076 | 0.0127017 | 0.07137144 | 0.05666422 | 5 |
| KEGG | hsa04514 | Cell adhesion molecules | 4/52 | 149/8076 | 0.01518057 | 0.08142305 | 0.06464453 | 4 |
| KEGG | hsa04014 | Ras signaling pathway | 5/52 | 232/8076 | 0.0162565 | 0.08340293 | 0.06621642 | 5 |
| KEGG | hsa05165 | Human papillomavirus infection | 6/52 | 331/8076 | 0.01885097 | 0.09268395 | 0.07358494 | 6 |
