# Supplementary material for: The Prognostic Value and Immunological Role of STEAP1 in Pan-Cancer: A Result of Data-Based Analysis
Source: Oxid Med Cell Longev. 2022 Mar 11;2022:8297011. doi: 10.1155/2022/8297011 (PMC8933652; doi:10.1155/2022/8297011)
Supplement: Supplementary 3 — Table S3: GSEA analysis. [file 8297011.f3.doc]

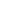

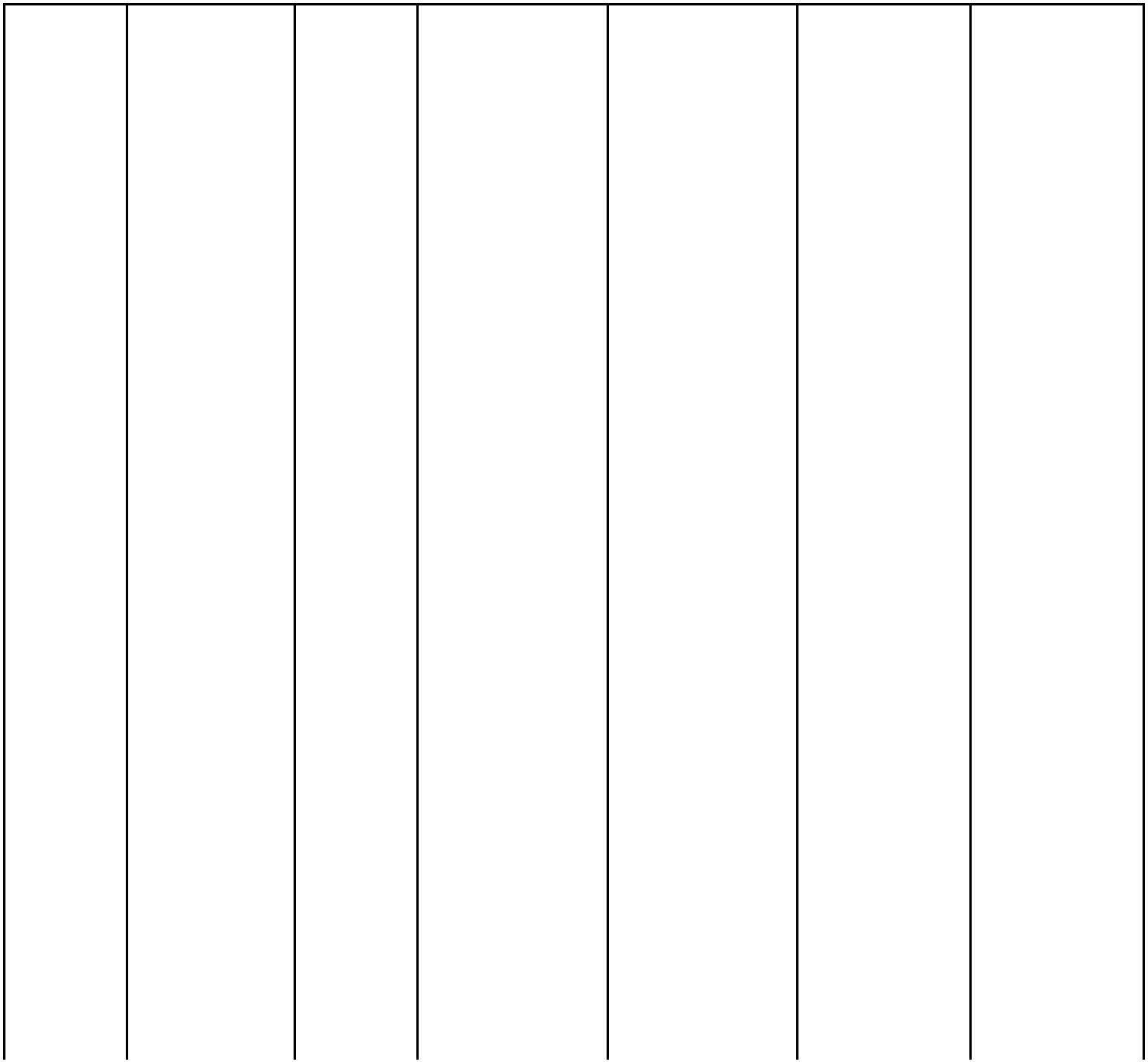
ID Description setSize enrichmentScore NES pvalue p.adjust

| GO_DIVA  LENT_INO  RGANIC_  CATION_  TRANSPO  RT |  | GO_DIVALEN  T_INORGANIC  _CATION_TR  ANSPORT 493 -0.517308677 -1.438095468 0.012987013 0.509968985 |
| --- | --- | --- |

| 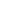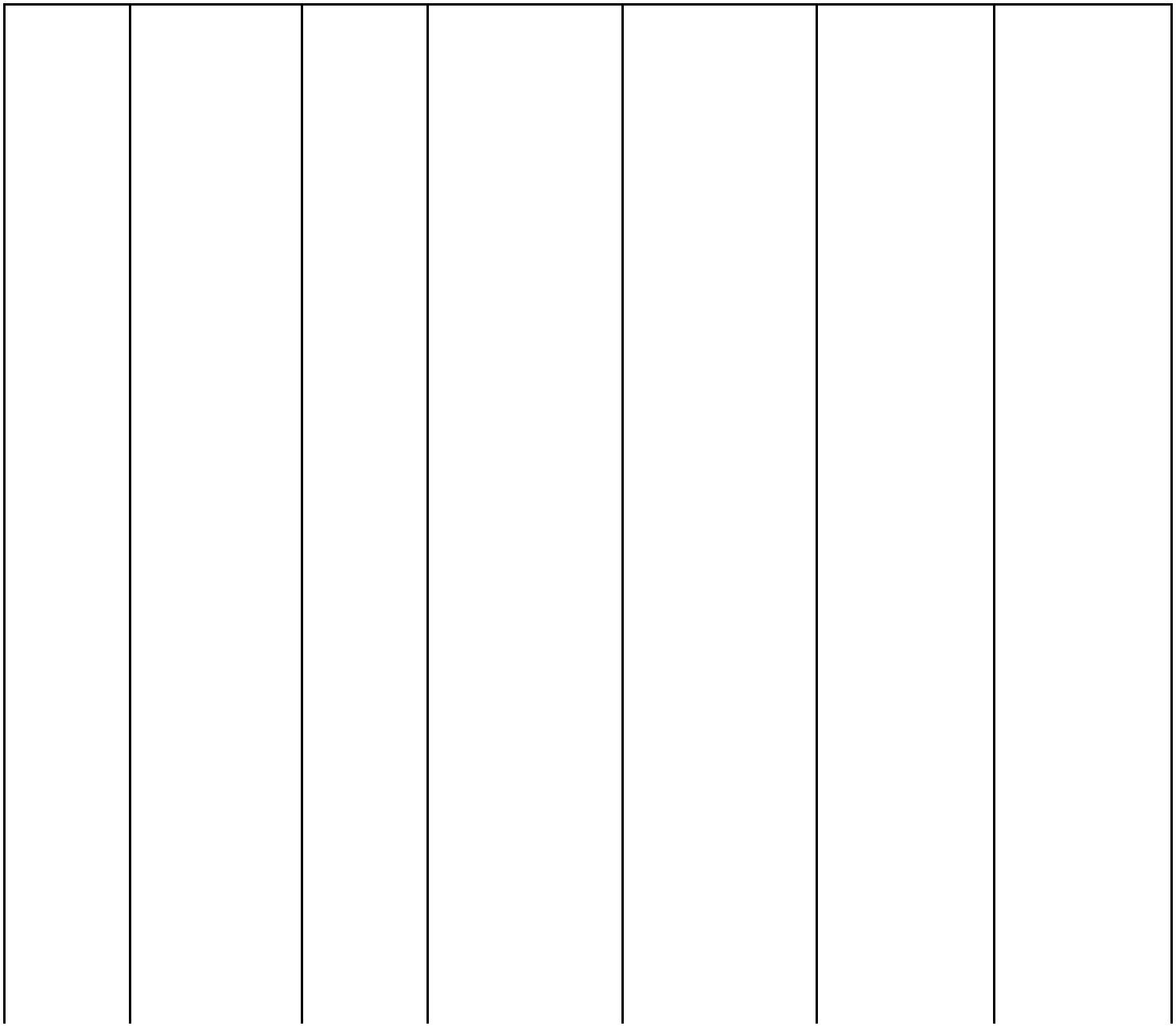GO_PASSI  VE_TRAN  SMEMBRA  NE_TRAN  SPORTER_  ACTIVITY |  | GO_PASSIVE_  TRANSMEMBR  ANE_TRANSP  ORTER_ACTIV  ITY 473 -0.523336296 -1.443872563 0.012987013 0.509968985 |
| --- | --- | --- |

| 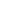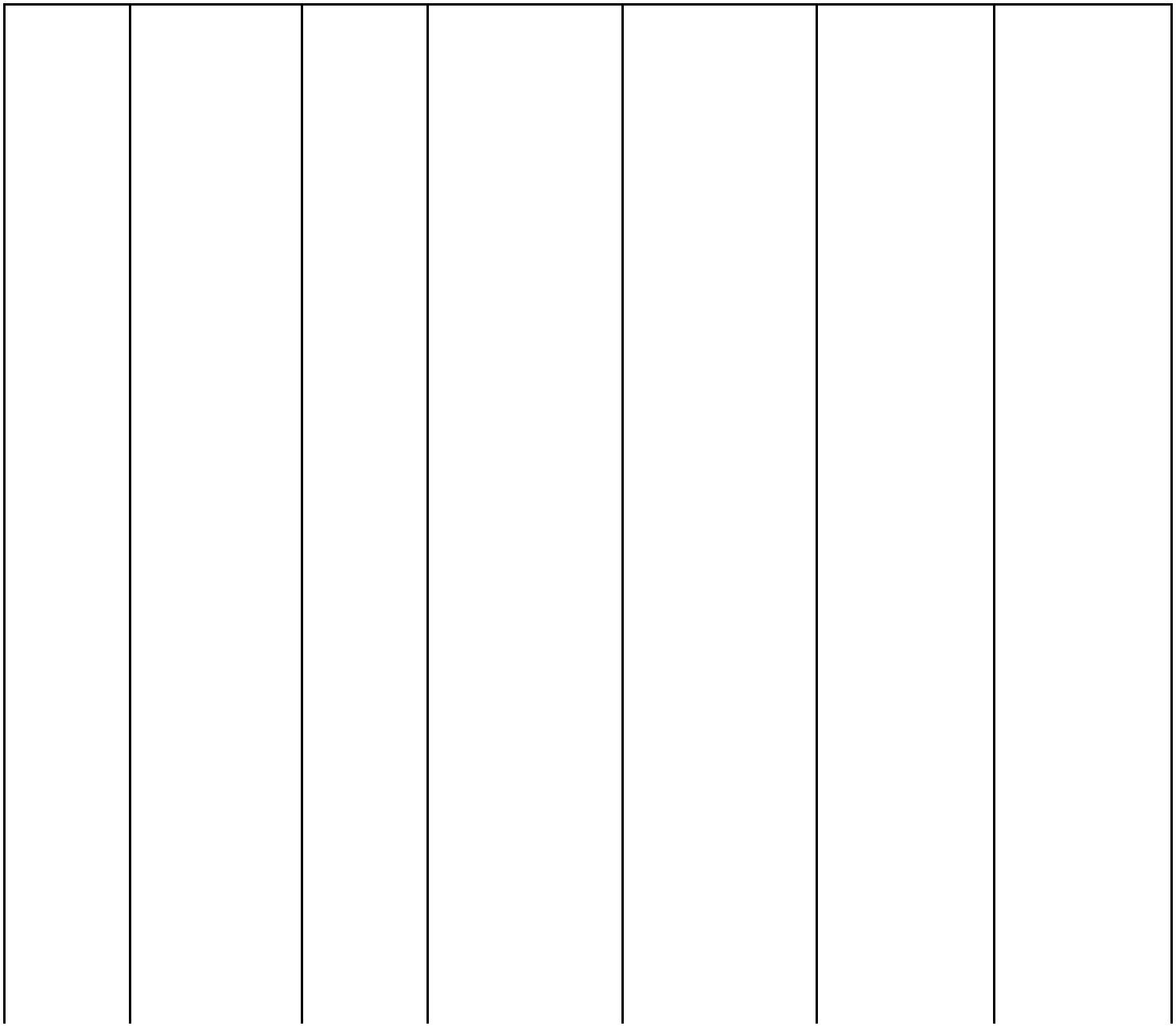GO_POSIT  IVE_REGU  LATION_  OF_SECRE  TION |  | GO_POSITIVE  _REGULATION  _OF_SECRETI  ON 430 -0.492239031 -1.357422185 0.012987013 0.509968985 |
| --- | --- | --- |

| 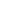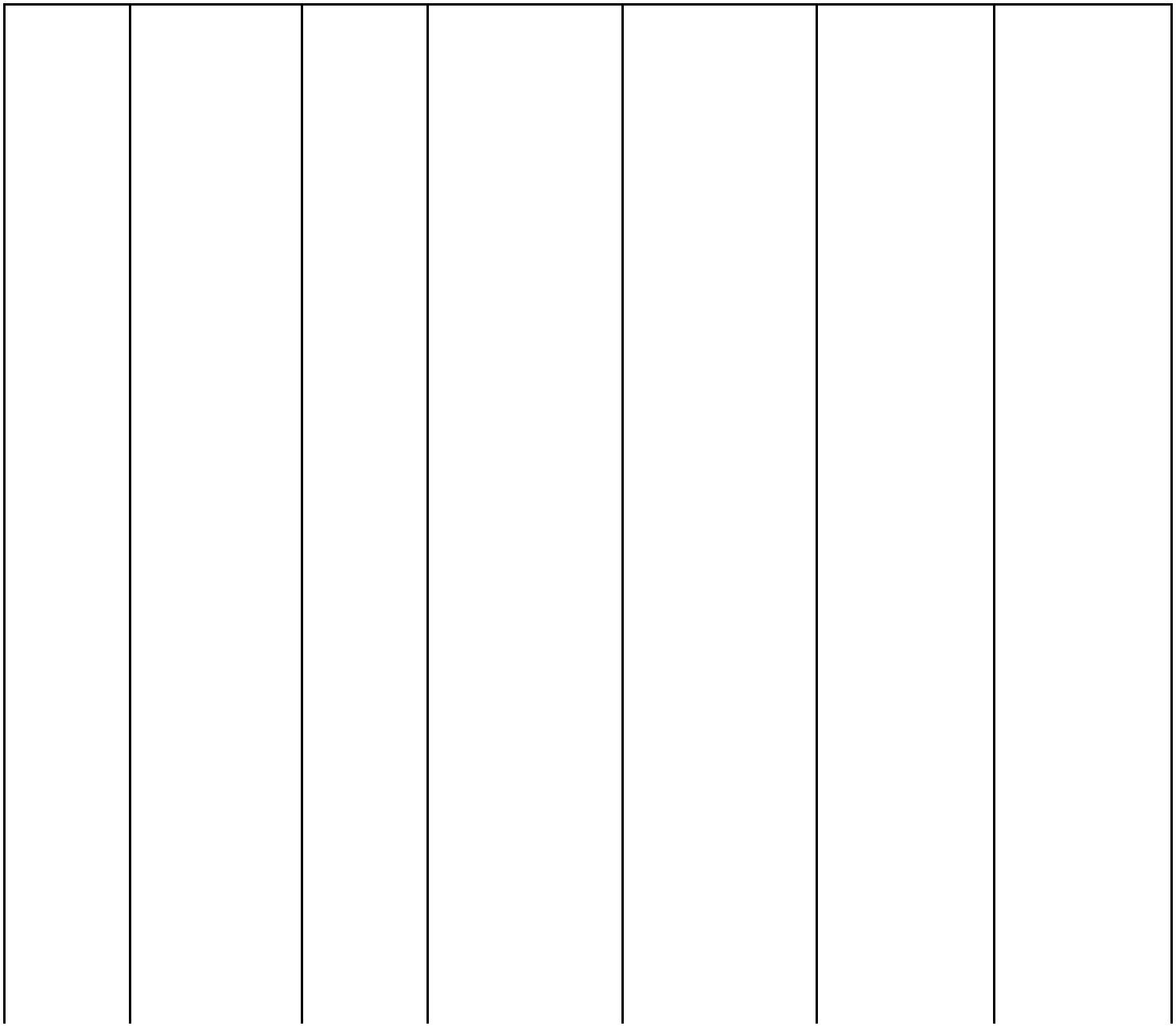GO_REGU  LATION_  OF_MEMB  RANE_PO  TENTIAL |  | GO_REGULATI  ON_OF_MEMB  RANE_POTEN  TIAL 432 -0.531260039 -1.462728469 0.012987013 0.509968985 |
| --- | --- | --- |

| 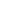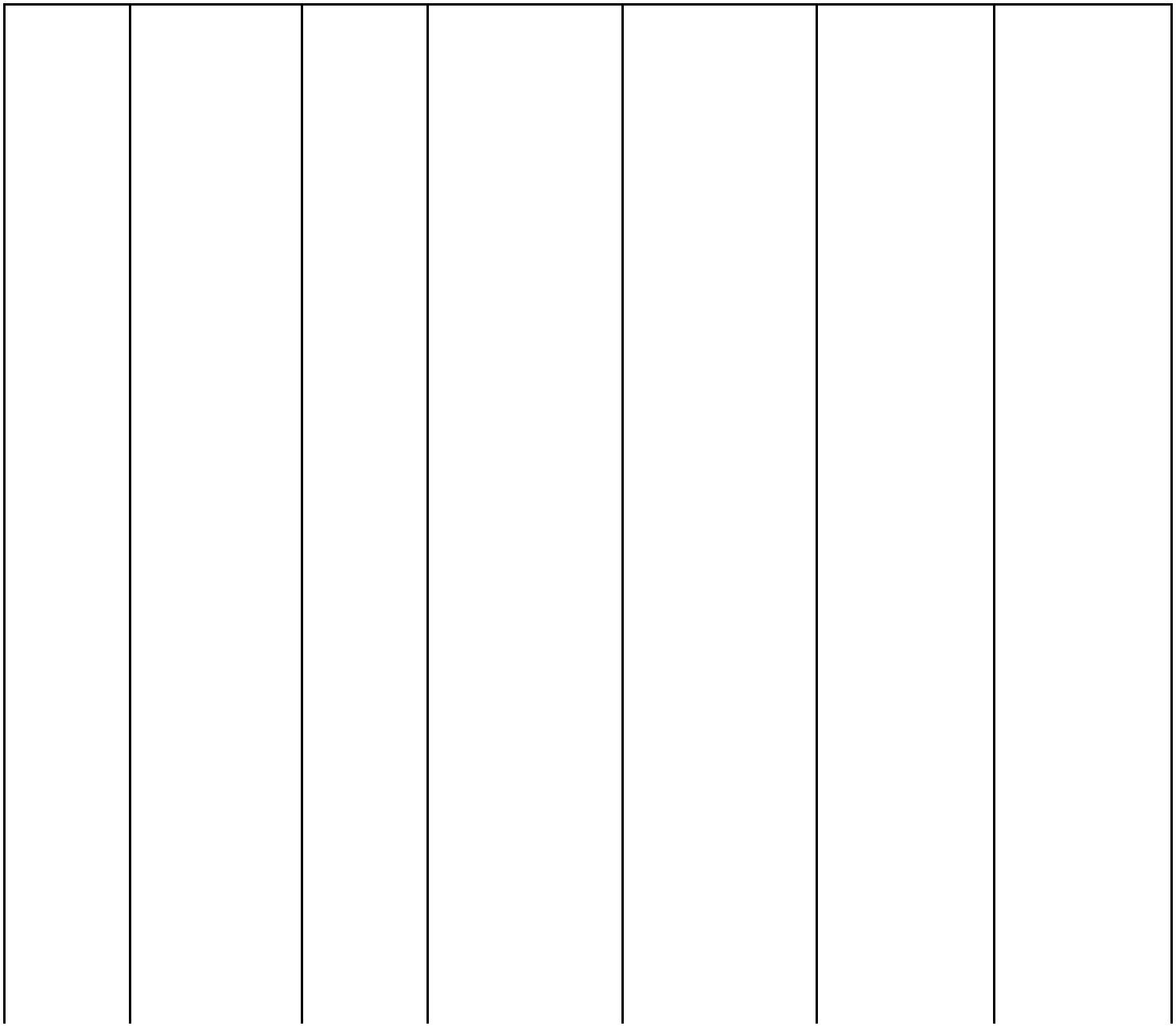GO_SYNA  PTIC_ME  MBRANE |  | GO_SYNAPTI  C_MEMBRANE 438 -0.59710691 -1.649870196 0.012987013 0.509968985 |
| --- | --- | --- |

| 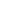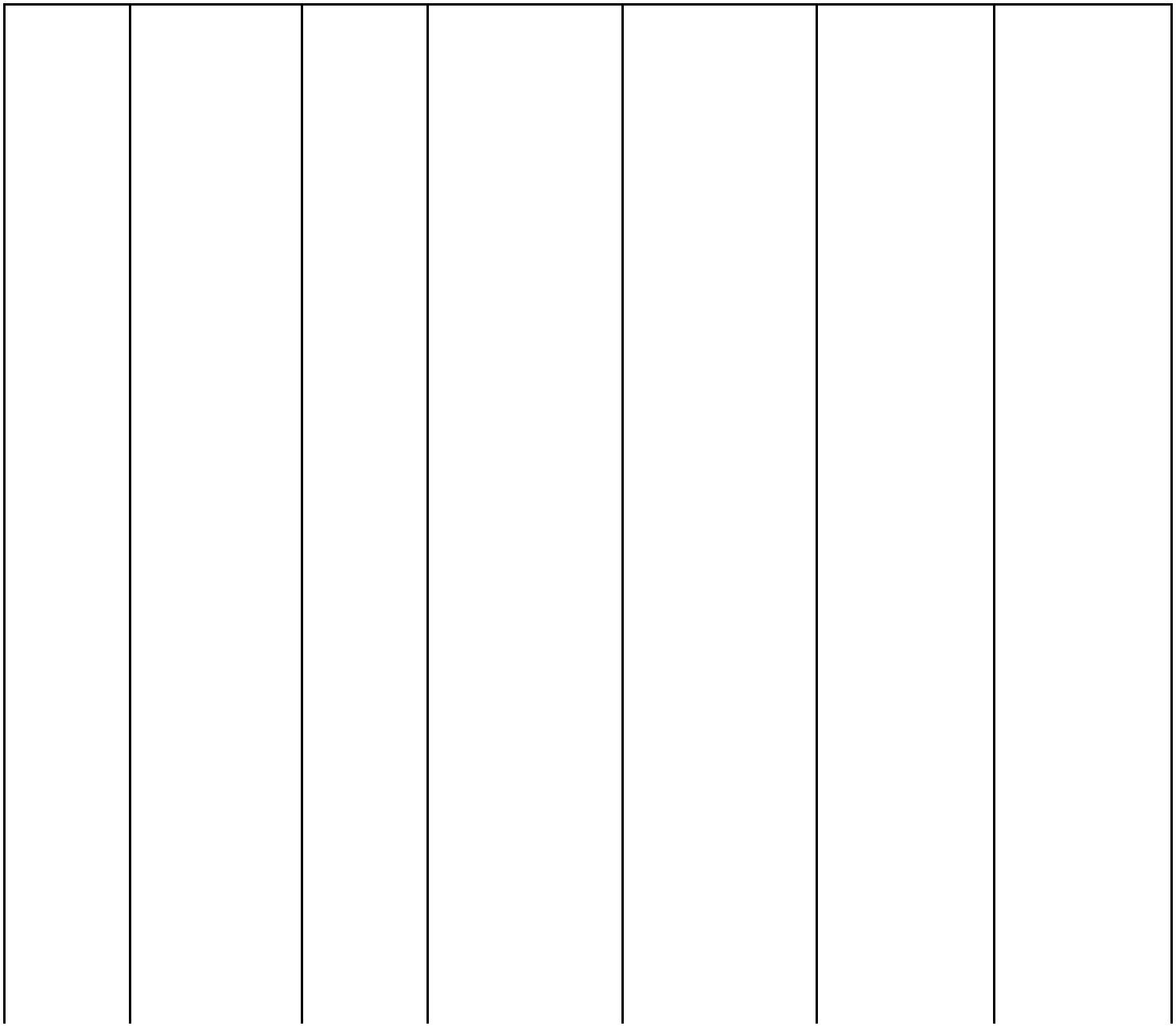GO_CELL  _GROWT  H |  | GO_CELL_GR  OWTH 477 -0.441606515 -1.215996822 0.013157895 0.509968985 |
| --- | --- | --- |

| 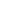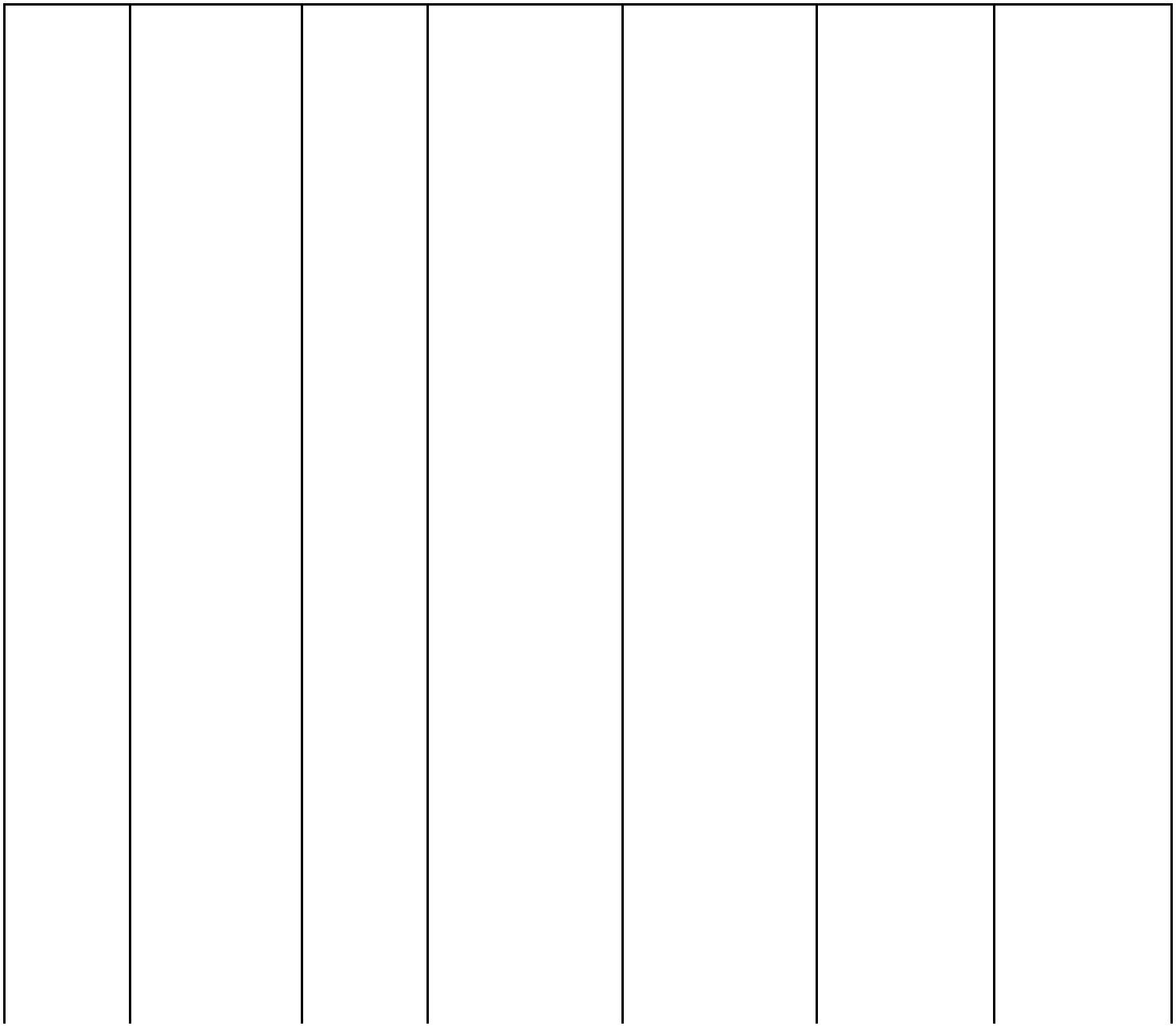GO_IMMU  NE_RESPO  NSE_REGU  LATING_C  ELL_SURF  ACE_REC  EPTOR_SI  GNALING  _PATHW  AY |  | GO_IMMUNE_  RESPONSE_RE  GULATING_CE  LL_SURFACE_  RECEPTOR_SI  GNALING_PA  THWAY 491 -0.643615253 -1.783041191 0.013157895 0.509968985 |
| --- | --- | --- |

| 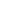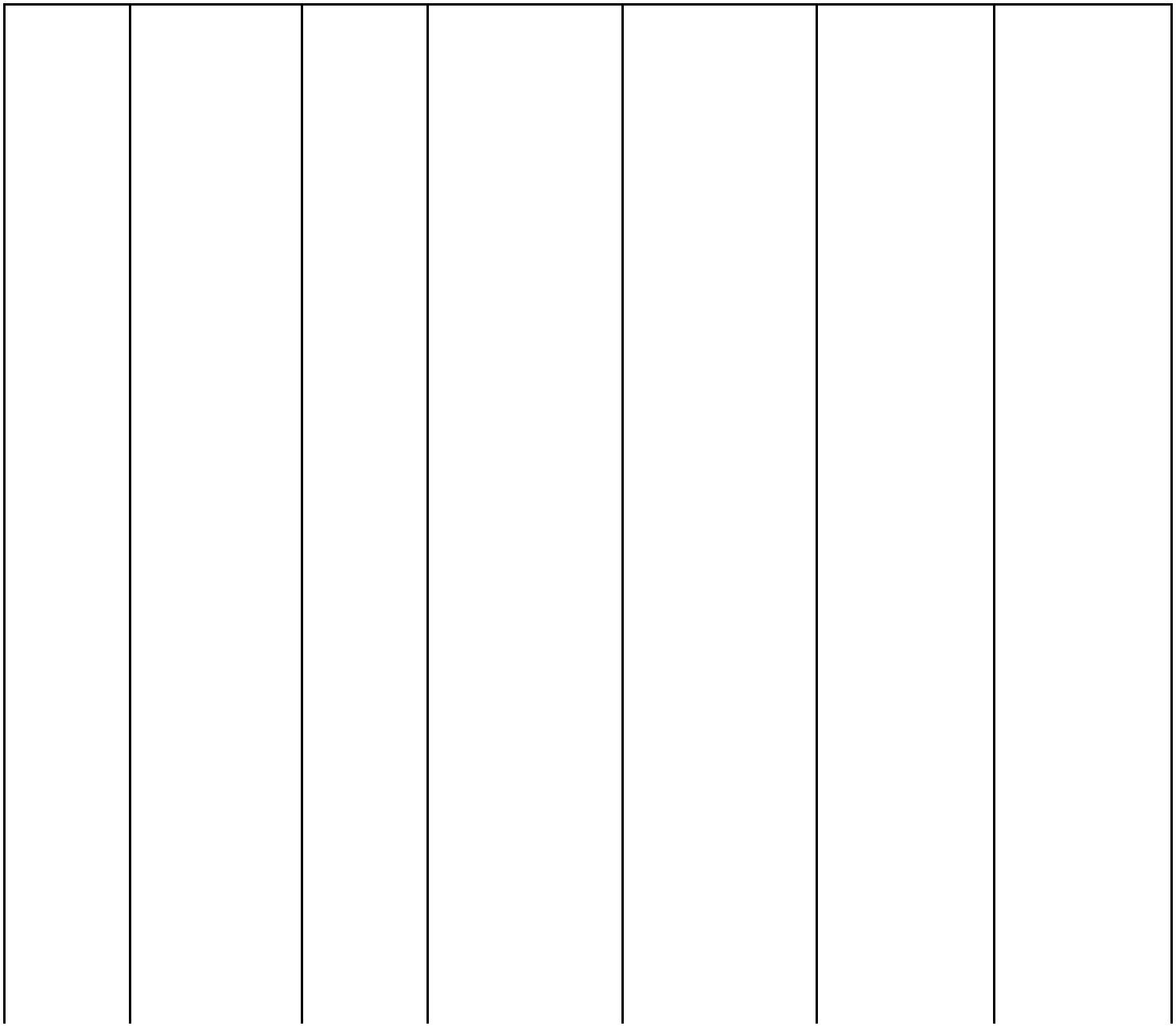GO_LEUK  OCYTE_M  IGRATION |  | GO_LEUKOCY  TE_MIGRATIO  N 497 -0.549002864 -1.525153659 0.013157895 0.509968985 |
| --- | --- | --- |

| 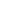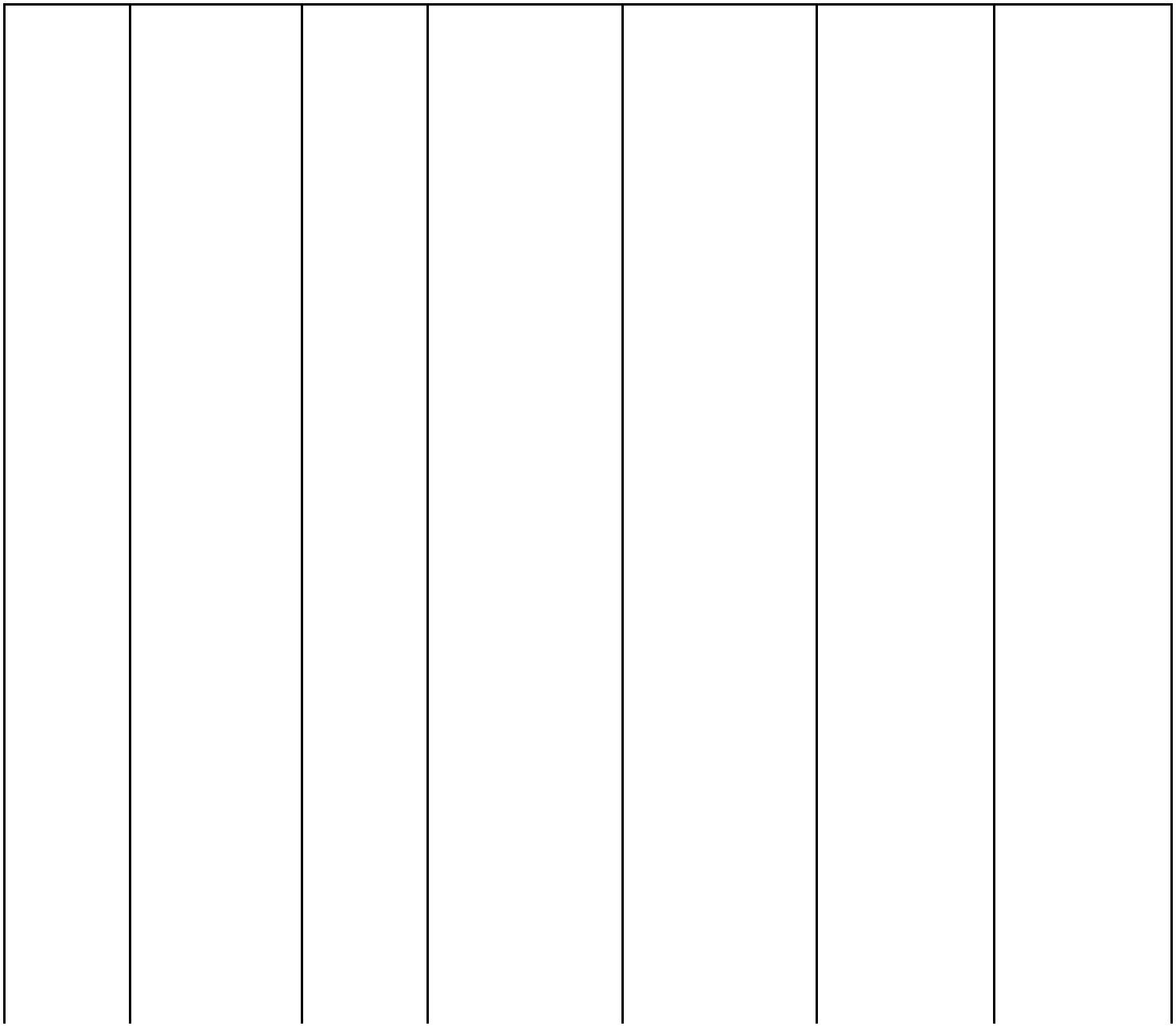GO_META  L_ION_TR  ANSMEM  BRANE_T  RANSPOR  TER_ACTI  VITY |  | GO_METAL_I  ON_TRANSME  MBRANE_TRA  NSPORTER_A  CTIVITY 442 -0.529902762 -1.461054393 0.013157895 0.509968985 |
| --- | --- | --- |

| 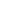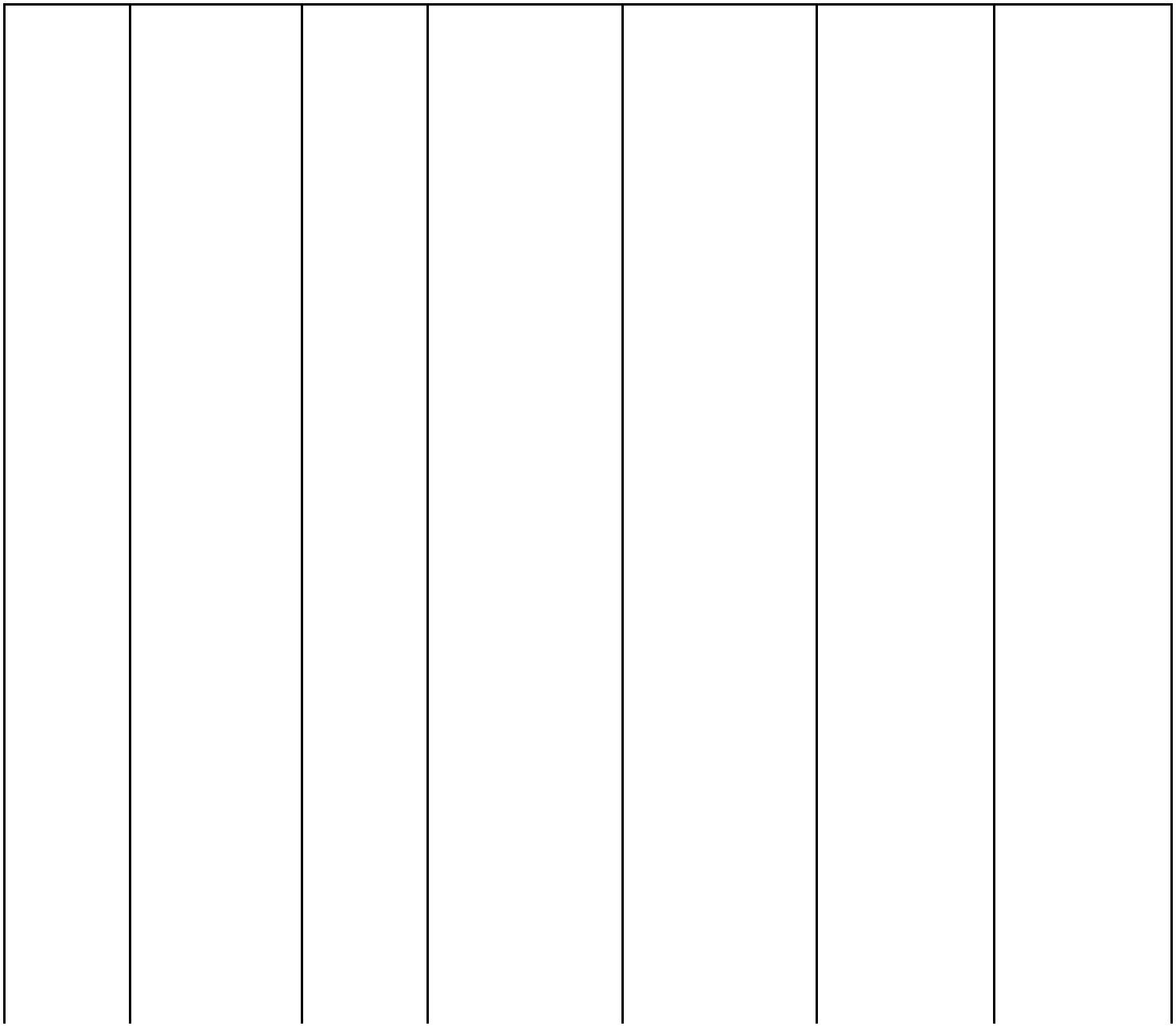GO_POSIT  IVE_REGU  LATION_  OF_ION_T  RANSPOR  T |  | GO_POSITIVE  _REGULATION  _OF_ION_TRA  NSPORT 275 -0.528227315 -1.429558227 0.013157895 0.509968985 |
| --- | --- | --- |

| 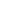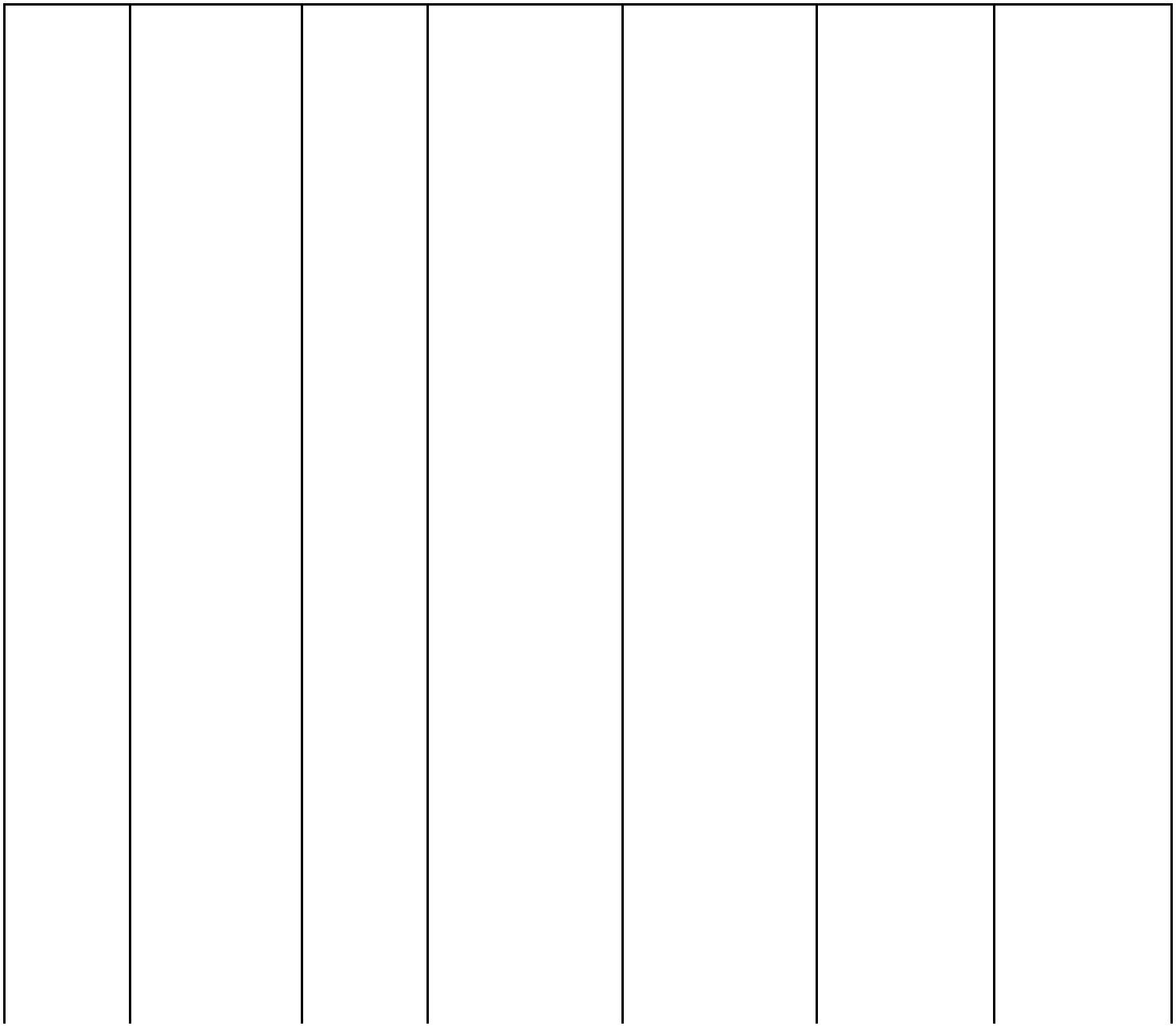GO_PROT  EIN_COM  PLEX_OLI  GOMERIZ  ATION |  | GO_PROTEIN_  COMPLEX_OL  IGOMERIZATI  ON 491 -0.443301043 -1.228100198 0.013157895 0.509968985 |
| --- | --- | --- |

| 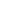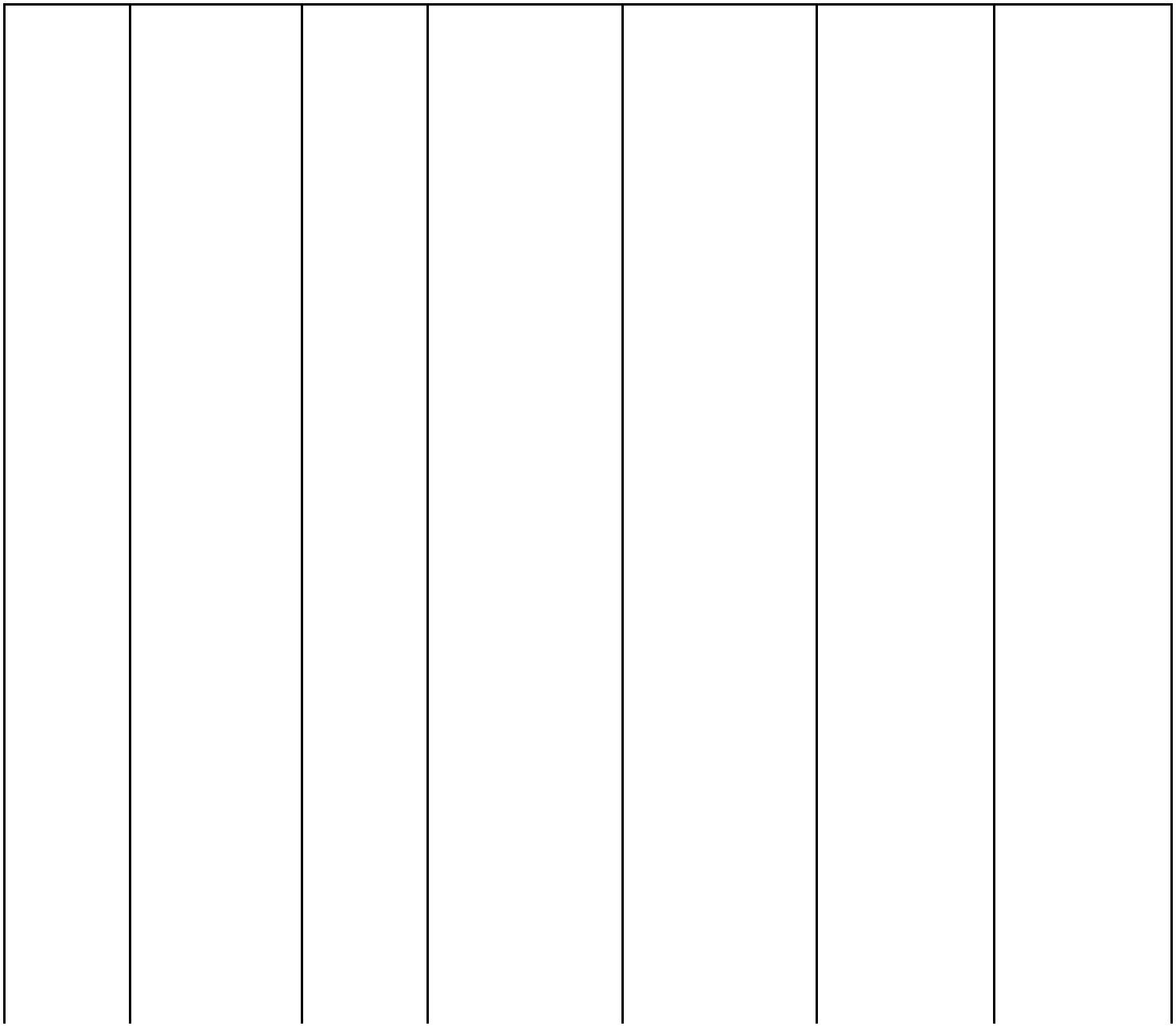GO_REGU  LATION_  OF_PEPTI  DASE_AC  TIVITY |  | GO_REGULATI  ON_OF_PEPTI  DASE_ACTIVI  TY 440 -0.448802683 -1.237643597 0.013157895 0.509968985 |
| --- | --- | --- |

| 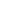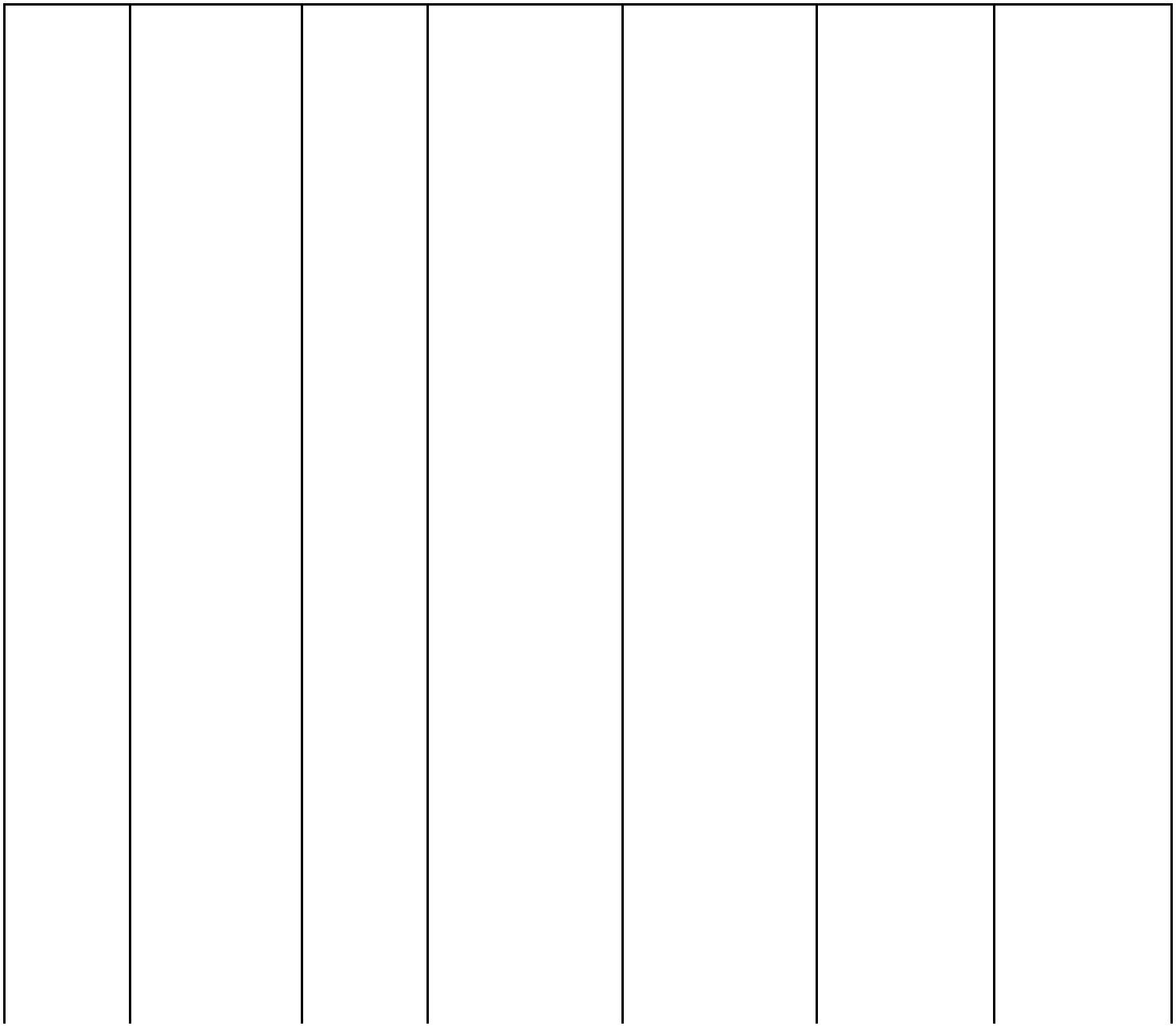GO_REGU  LATION_  OF_TRAN  S_SYNAP  TIC_SIGN  ALING |  | GO_REGULATI  ON_OF_TRAN  S_SYNAPTIC_  SIGNALING 443 -0.551781786 -1.520420067 0.013157895 0.509968985 |
| --- | --- | --- |

| 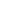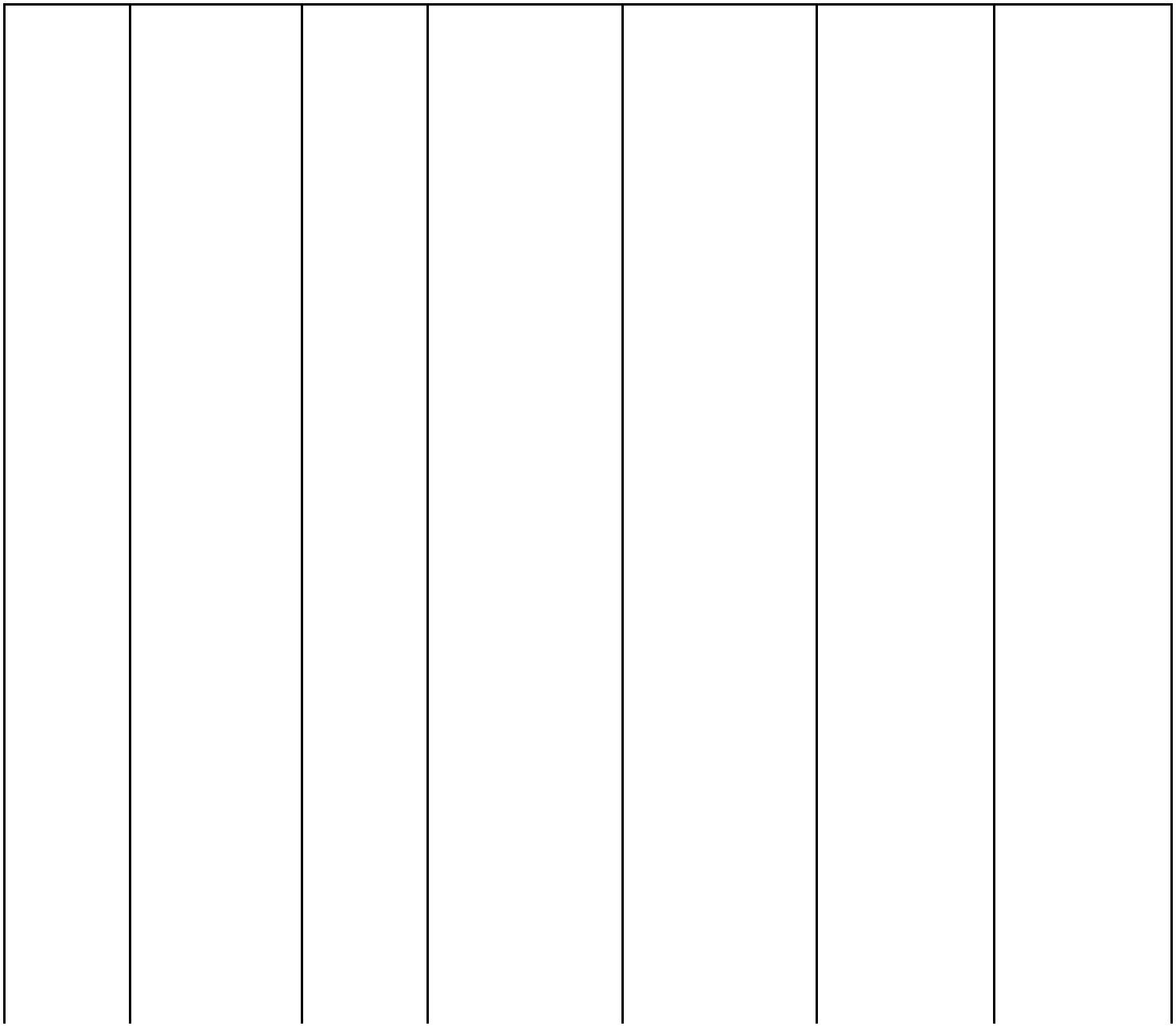GO_RESP  ONSE_TO  _RADIATI  ON |  | GO_RESPONS  E_TO_RADIAT  ION 441 -0.490414911 -1.352518544 0.013157895 0.509968985 |
| --- | --- | --- |

| 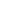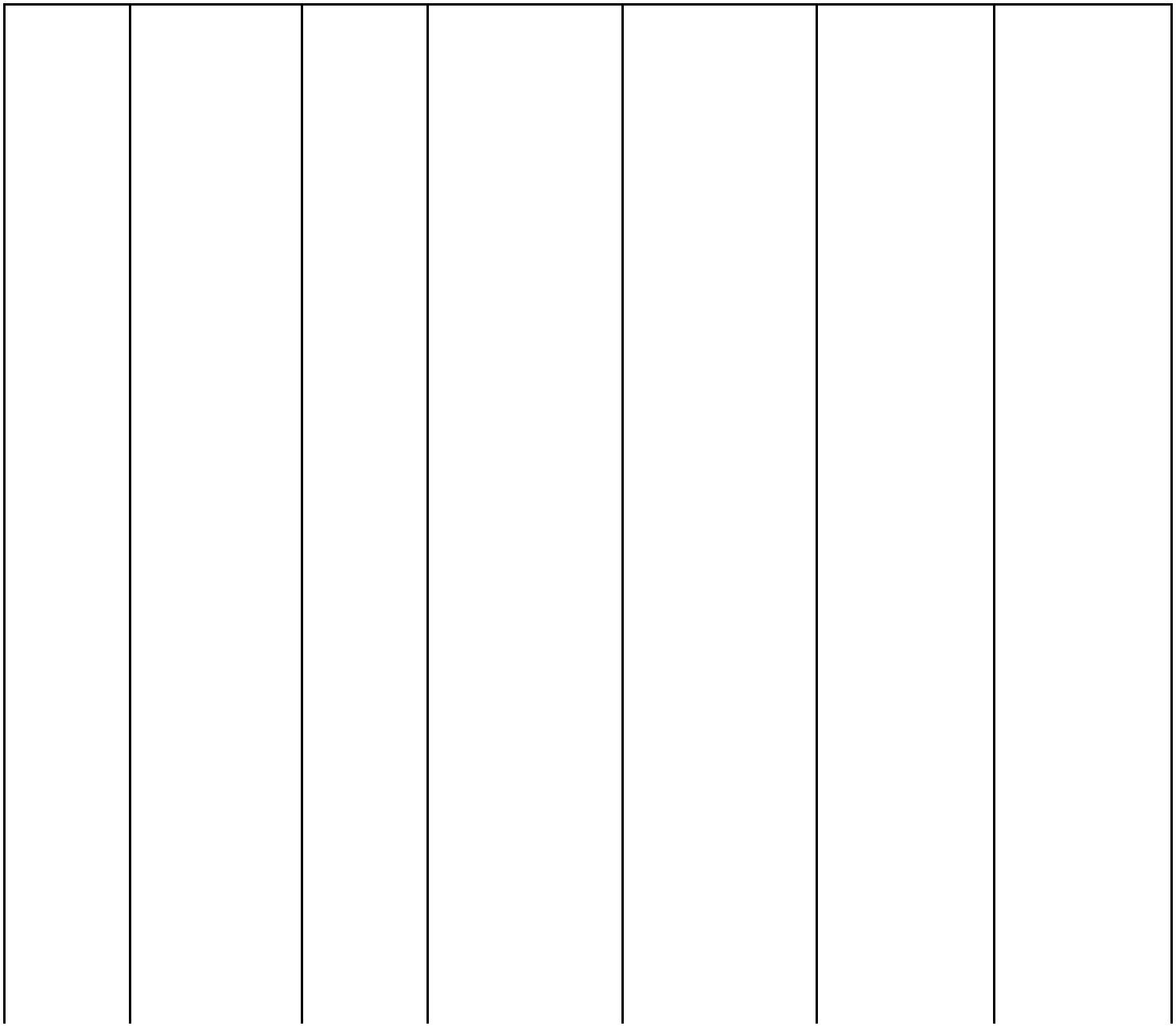GO_SECO  ND_MESS  ENGER_M  EDIATED_  SIGNALIN  G |  | GO_SECOND_  MESSENGER_  MEDIATED_SI  GNALING 440 -0.533087261 -1.470071505 0.013157895 0.509968985 |
| --- | --- | --- |

| 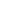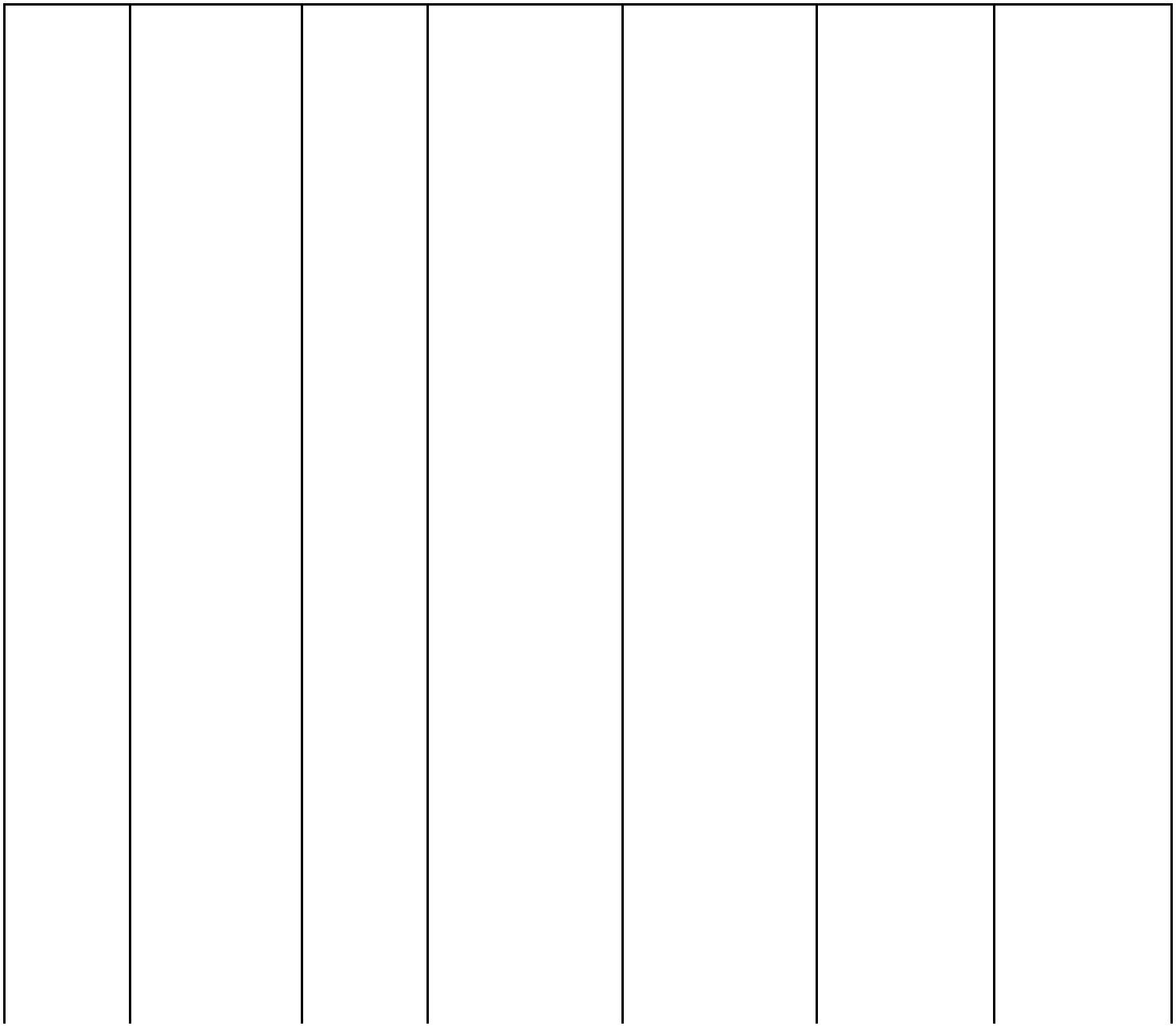GO_NEGA  TIVE_REG  ULATION_  OF_IMMU  NE_SYSTE  M_PROCE  SS |  | GO_NEGATIV  E_REGULATIO  N_OF_IMMUN  E_SYSTEM_PR  OCESS 470 -0.443834238 -1.220481221 0.013333333 0.509968985 |
| --- | --- | --- |

| 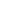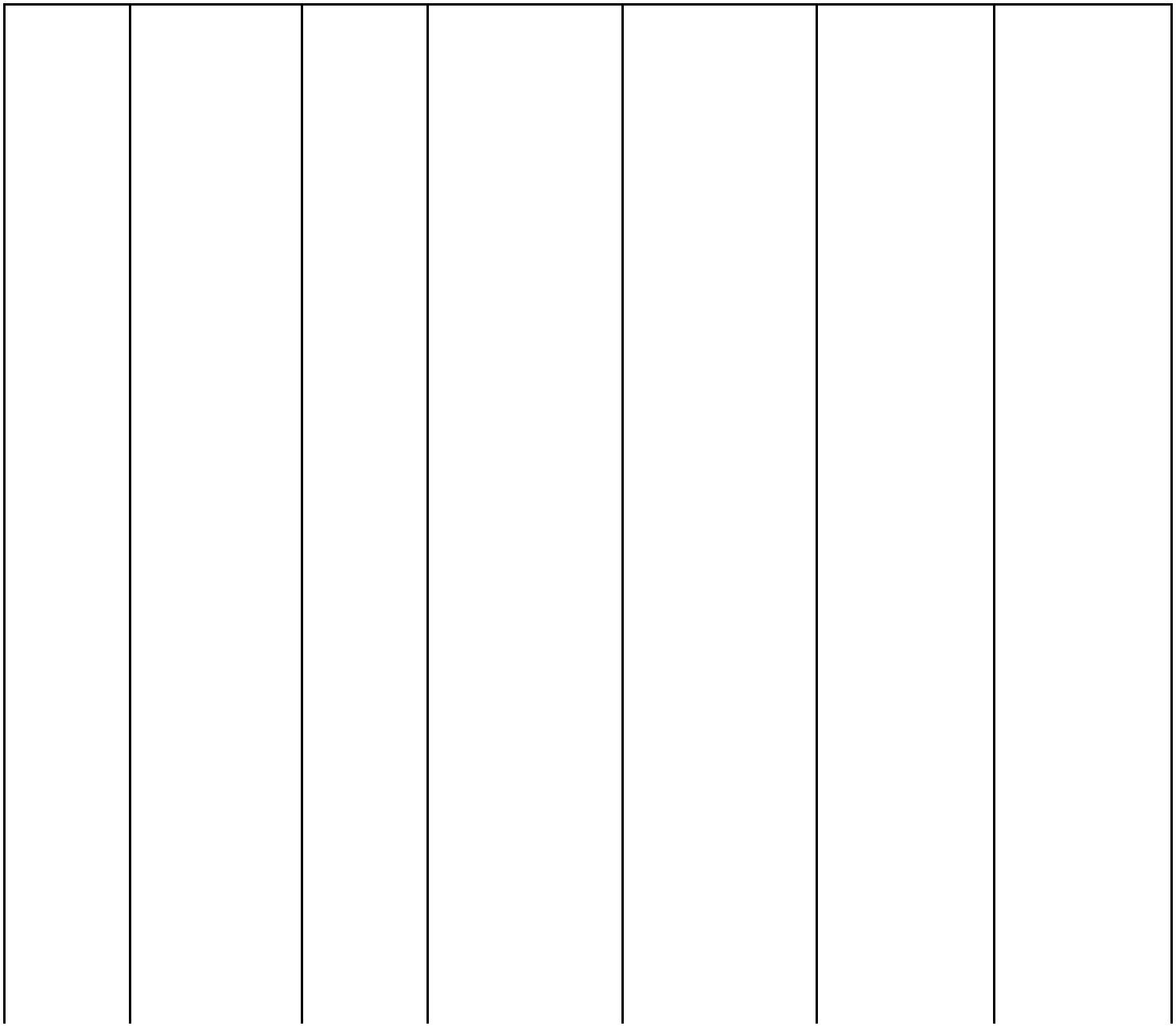GO_REGU  LATION_  OF_CELL_  CELL_AD  HESION |  | GO_REGULATI  ON_OF_CELL_  CELL_ADHESI  ON 407 -0.517043987 -1.420085242 0.013333333 0.509968985 |
| --- | --- | --- |

| 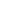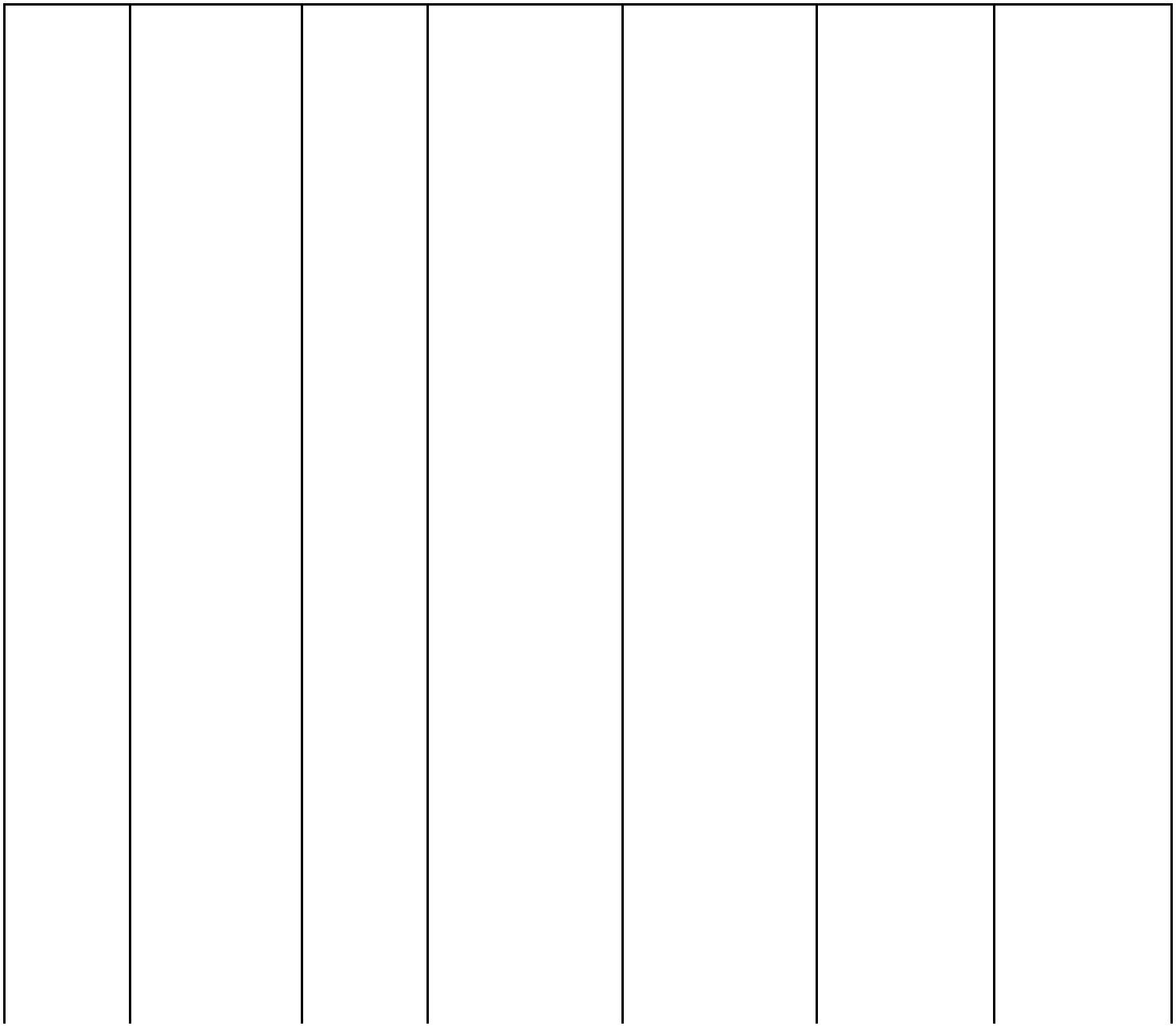GO_REGU  LATION_  OF_LEUK  OCYTE_DI  FFERENTI  ATION |  | GO_REGULATI  ON_OF_LEUK  OCYTE_DIFFE  RENTIATION 274 -0.530420752 -1.432094482 0.013333333 0.509968985 |
| --- | --- | --- |

| 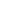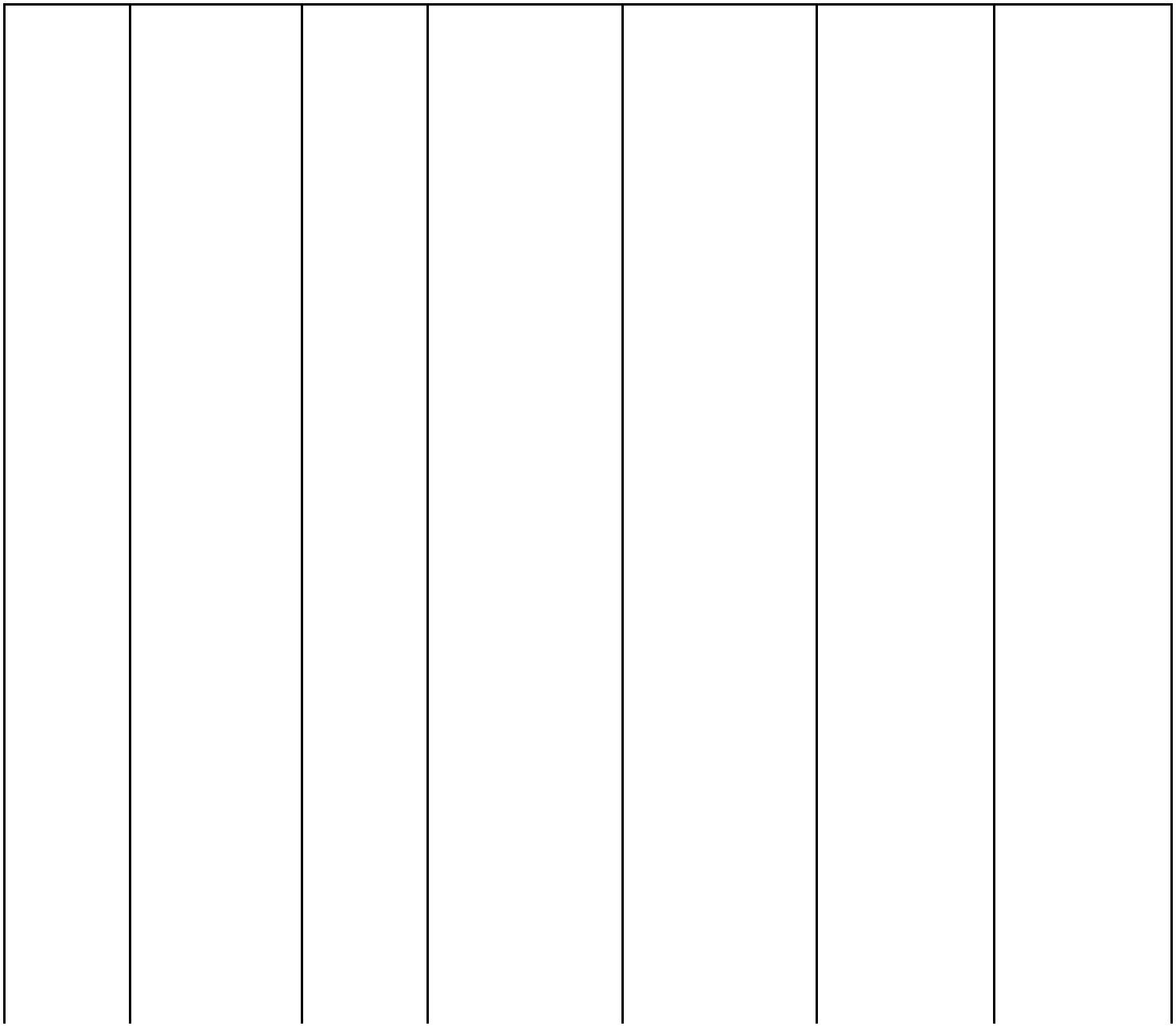GO_REGU  LATION_  OF_LYMP  HOCYTE_  ACTIVATI  ON |  | GO_REGULATI  ON_OF_LYMP  HOCYTE_ACT  IVATION 486 -0.607608117 -1.678261719 0.013333333 0.509968985 |
| --- | --- | --- |

| 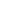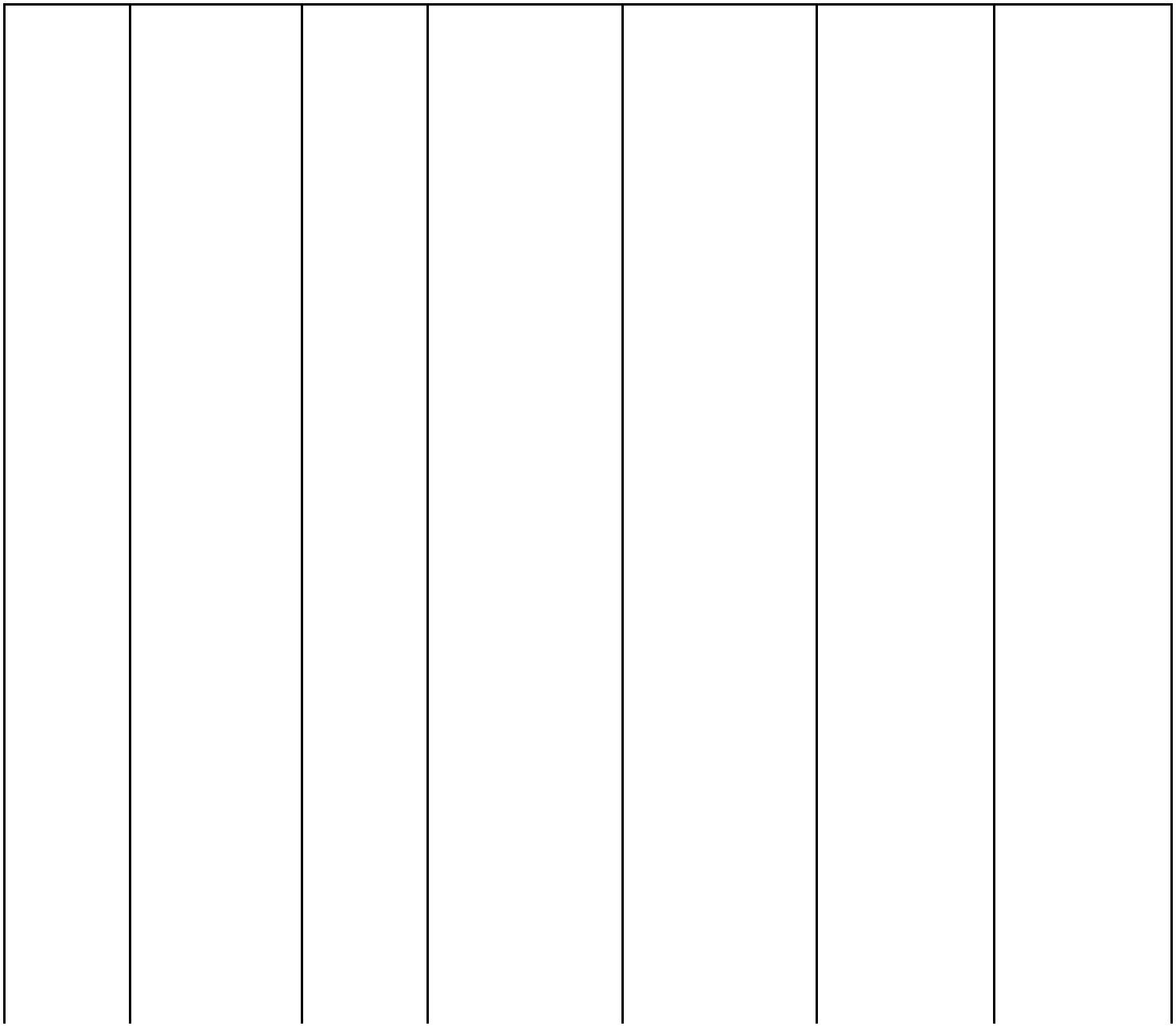GO_B_CE  LL_MEDIA  TED_IMM  UNITY |  | GO_B_CELL_  MEDIATED_IM  MUNITY 215 -0.703737661 -1.861636068 0.013513514 0.509968985 |
| --- | --- | --- |

| 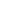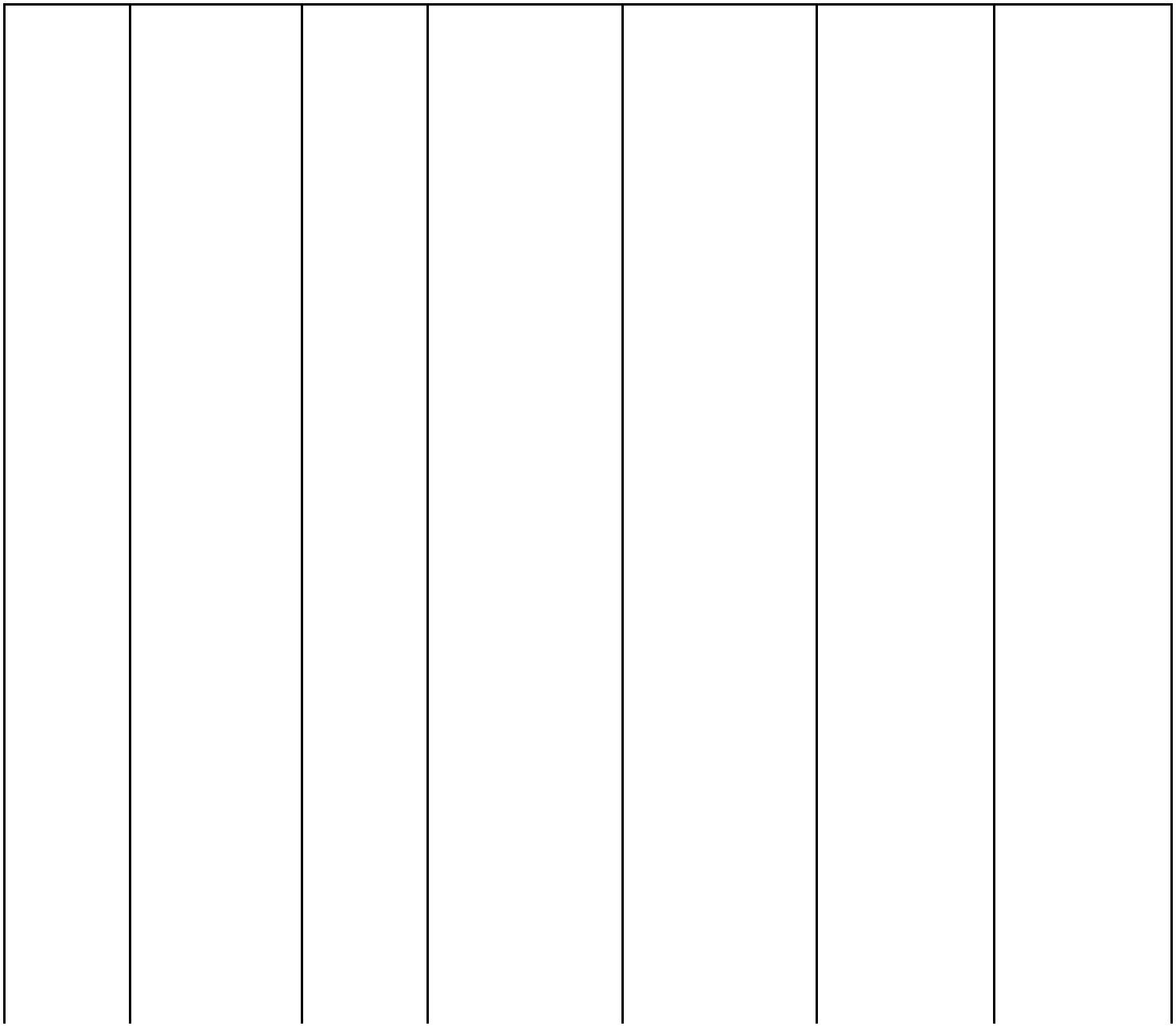GO_CALC  IUM_MEDI  ATED_SIG  NALING |  | GO_CALCIUM  _MEDIATED_S  IGNALING 218 -0.595175798 -1.573749061 0.013513514 0.509968985 |
| --- | --- | --- |

| 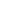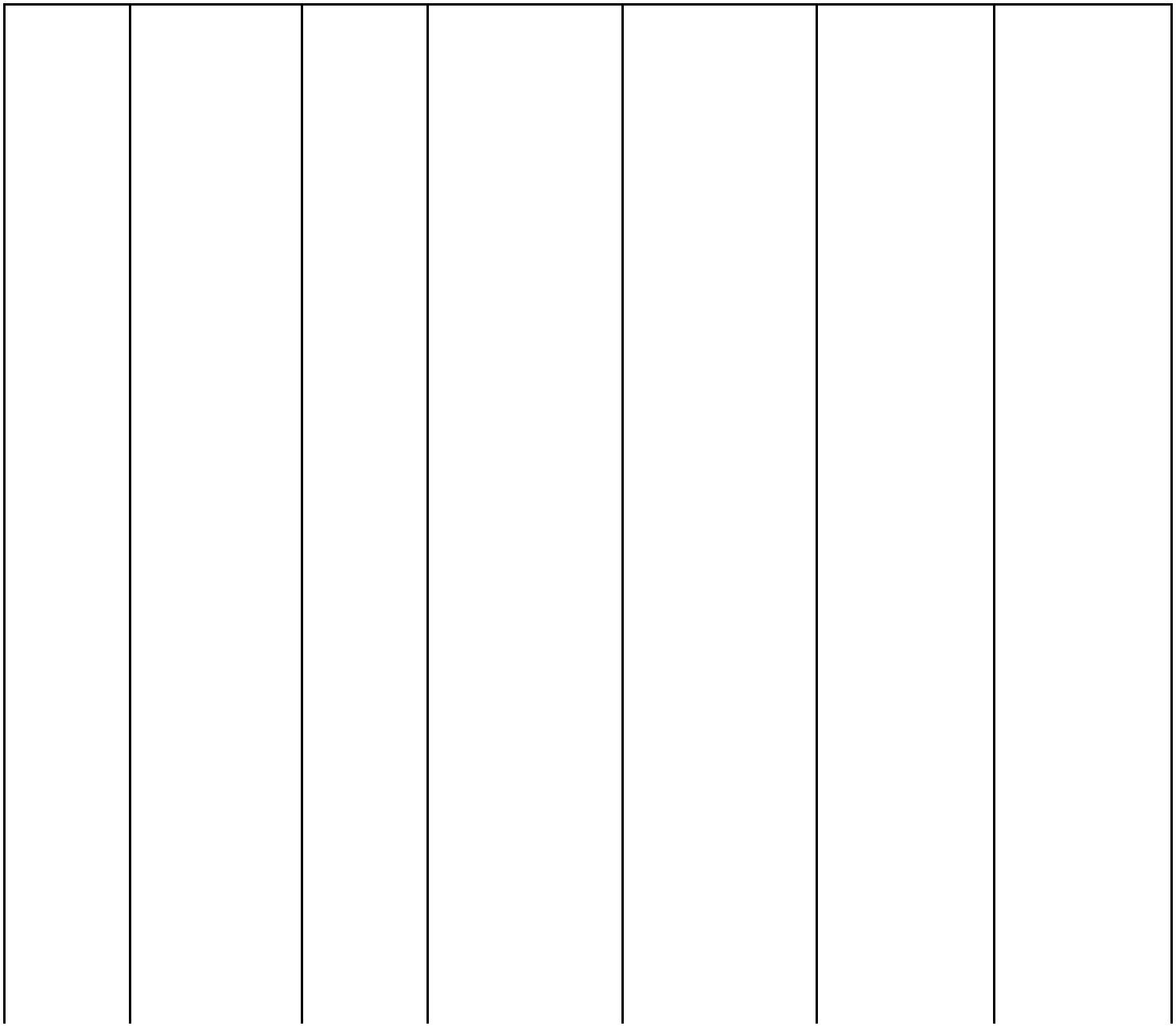GO_MON  OVALENT  _INORGA  NIC_CATI  ON_TRAN  SMEMBRA  NE_TRAN  SPORTER_  ACTIVITY |  | GO_MONOVA  LENT_INORGA  NIC_CATION_  TRANSMEMBR  ANE_TRANSP  ORTER_ACTIV  ITY 388 -0.527427983 -1.424877657 0.013513514 0.509968985 |
| --- | --- | --- |

| 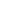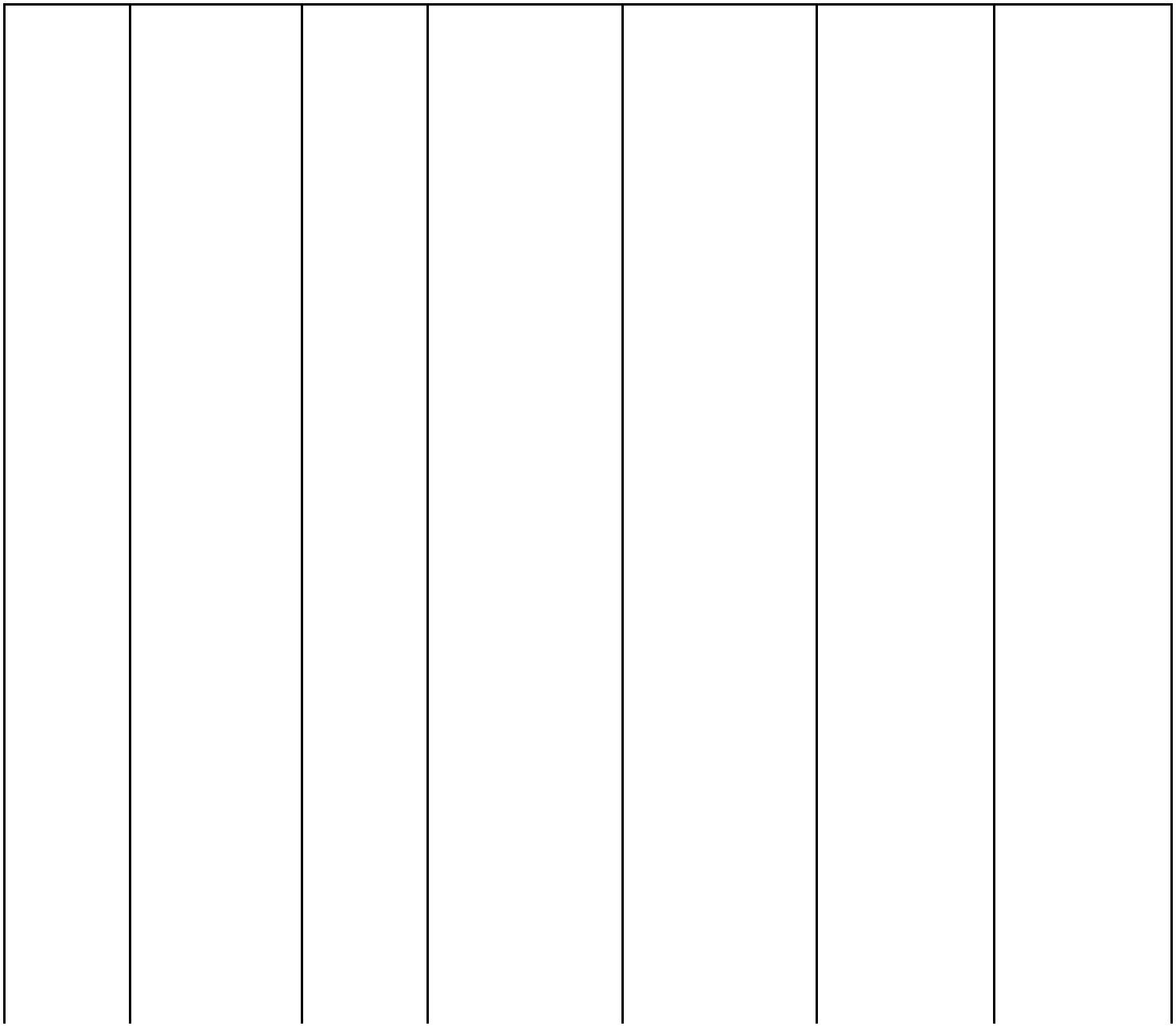GO_OSSIF  ICATION |  | GO_OSSIFICA  TION 389 -0.484716906 -1.310126189 0.013513514 0.509968985 |
| --- | --- | --- |

| 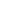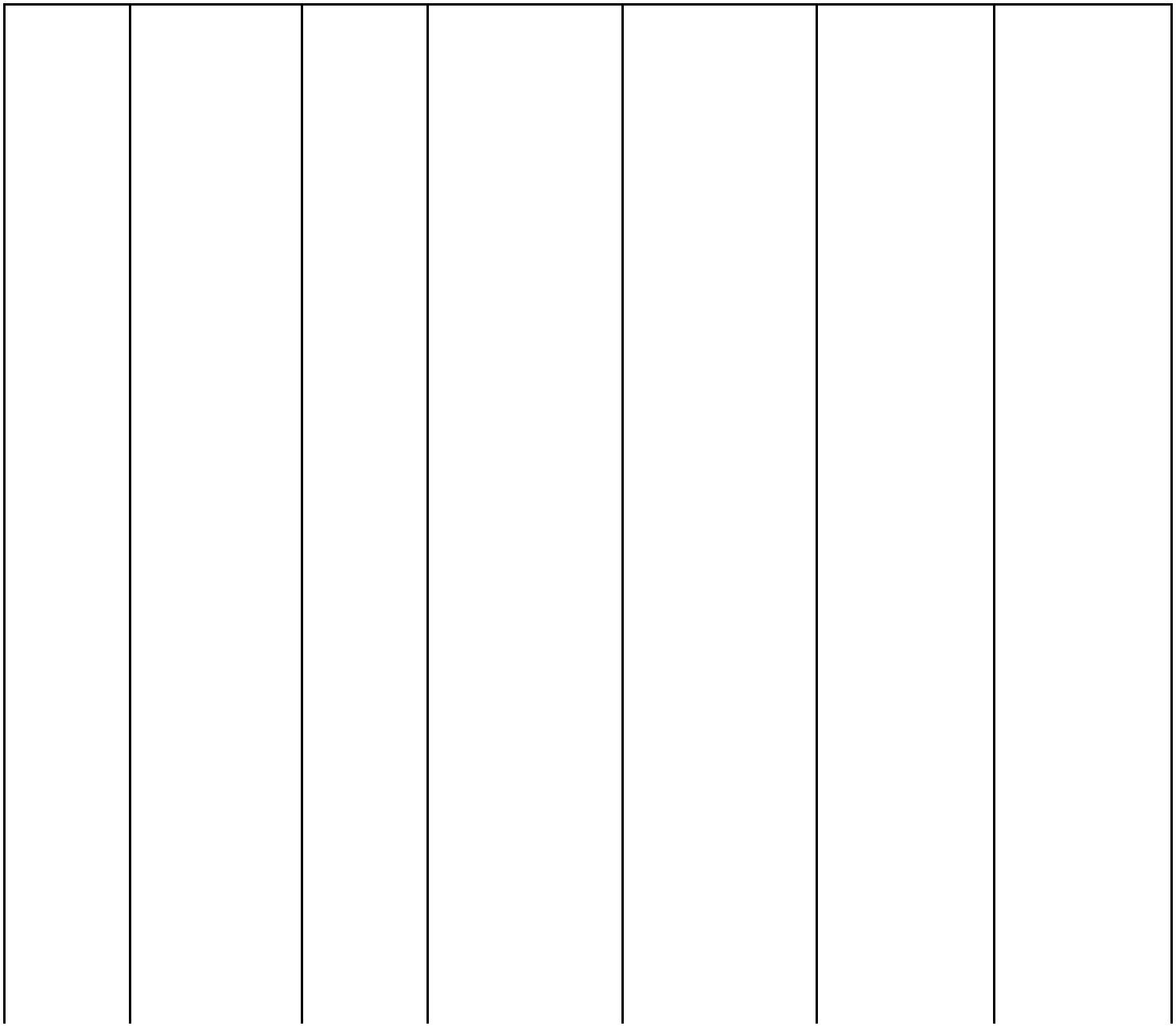GO_POSIT  IVE_REGU  LATION_  OF_CELL_  CELL_AD  HESION |  | GO_POSITIVE  _REGULATION  _OF_CELL_CE  LL_ADHESION 256 -0.590124521 -1.580951723 0.013513514 0.509968985 |
| --- | --- | --- |

| 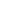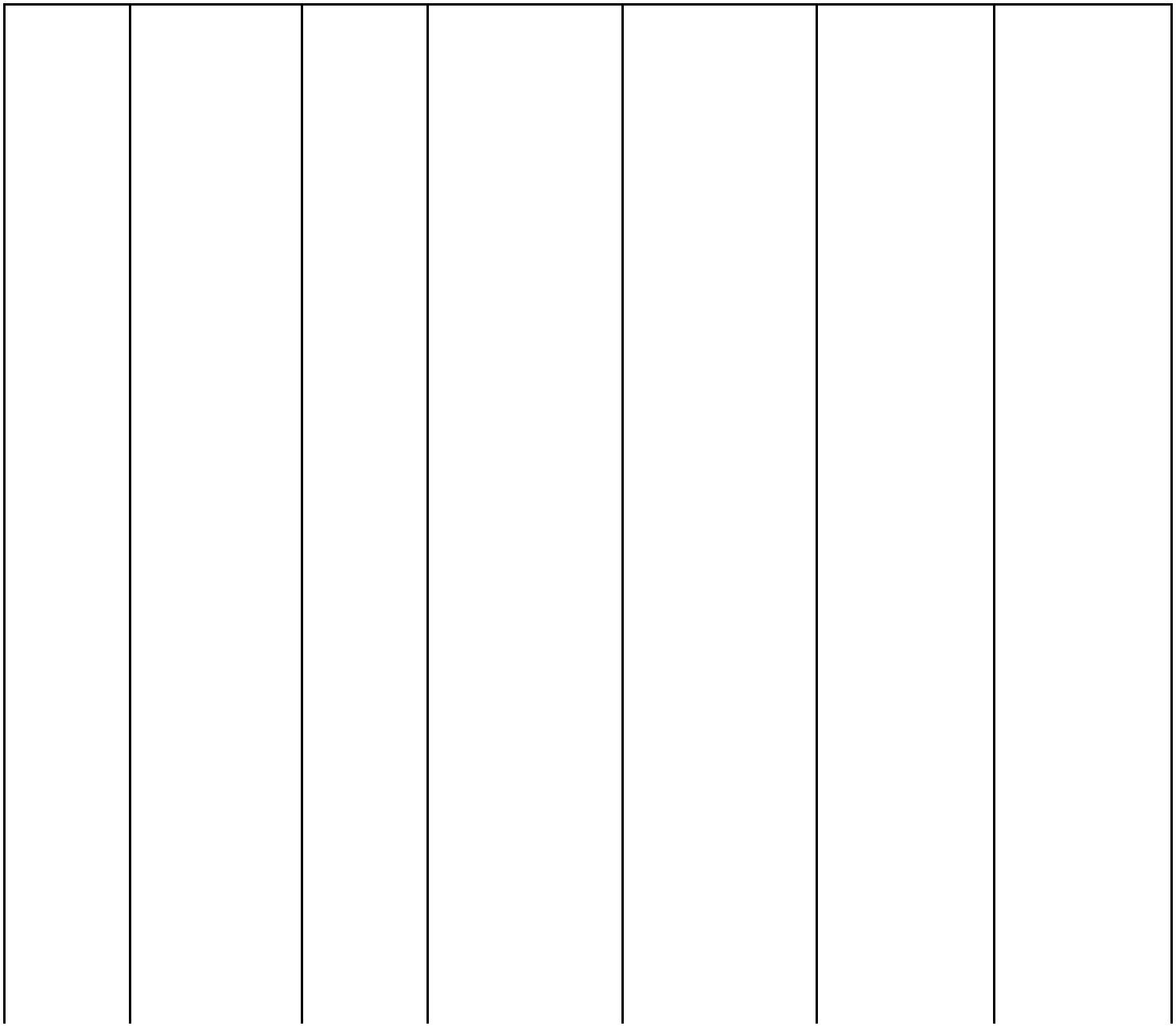GO_REGU  LATION_  OF_ANAT  OMICAL_  STRUCTU  RE_SIZE |  | GO_REGULATI  ON_OF_ANAT  OMICAL_STR  UCTURE_SIZE 499 -0.451371836 -1.248366867 0.013513514 0.509968985 |
| --- | --- | --- |

| 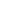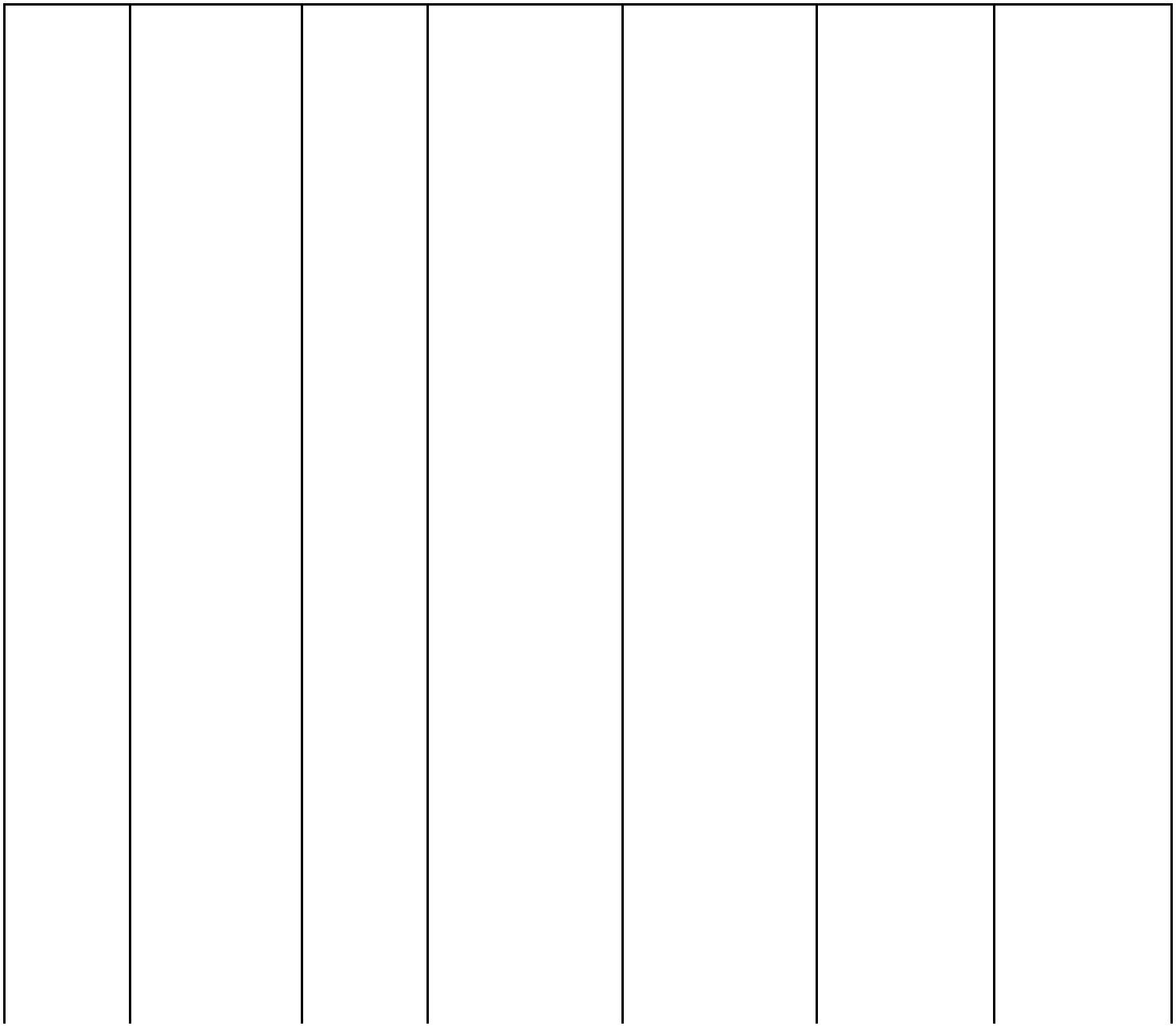GO_REGU  LATION_  OF_ION_T  RANSME  MBRANE_  TRANSPO  RT |  | GO_REGULATI  ON_OF_ION_T  RANSMEMBR  ANE_TRANSP  ORT 482 -0.568866805 -1.565013863 0.013513514 0.509968985 |
| --- | --- | --- |

| 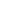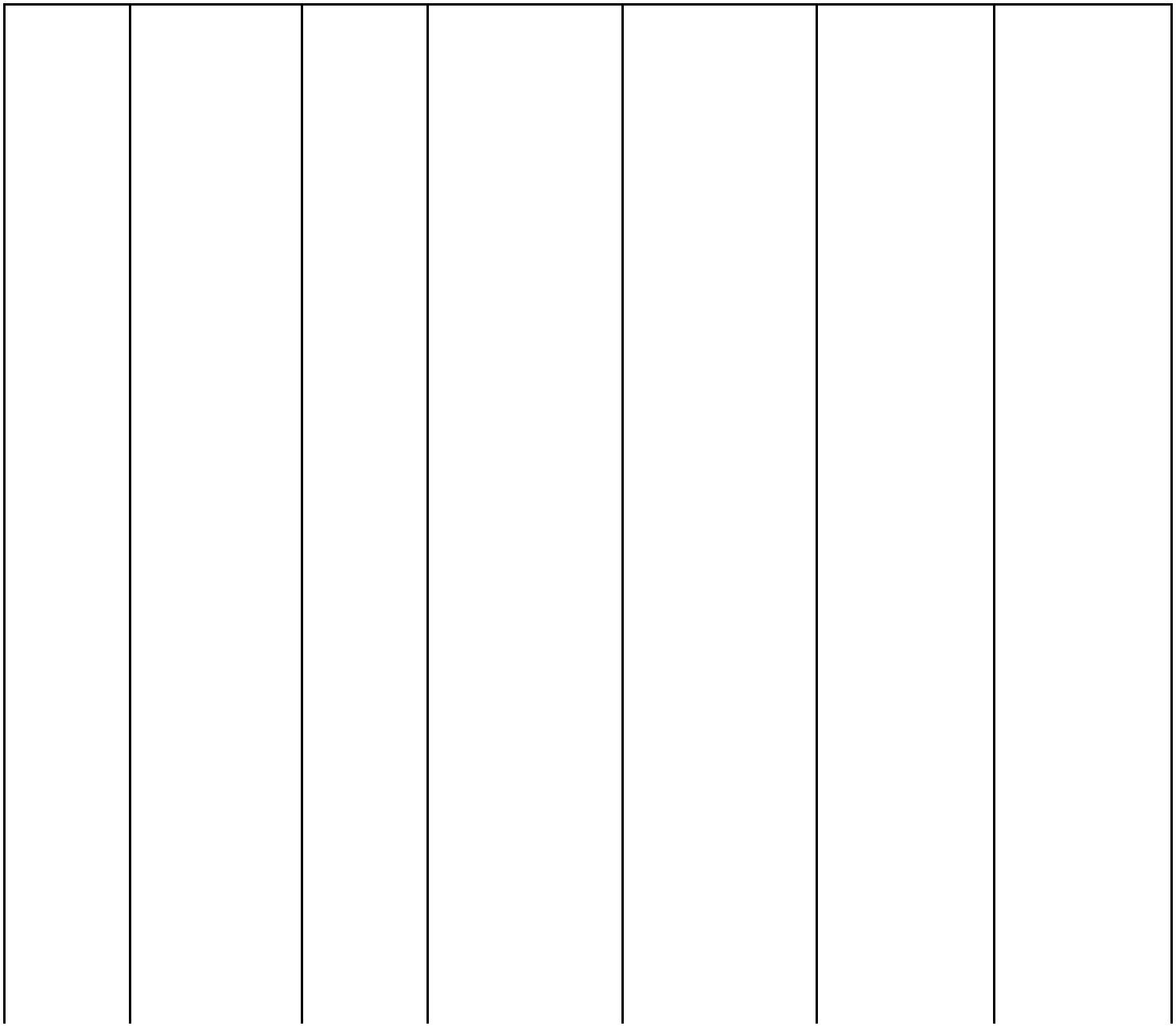GO_REGU  LATION_  OF_PEPTI  DE_SECRE  TION |  | GO_REGULATI  ON_OF_PEPTI  DE_SECRETIO  N 499 -0.507893972 -1.404691114 0.013513514 0.509968985 |
| --- | --- | --- |

| 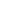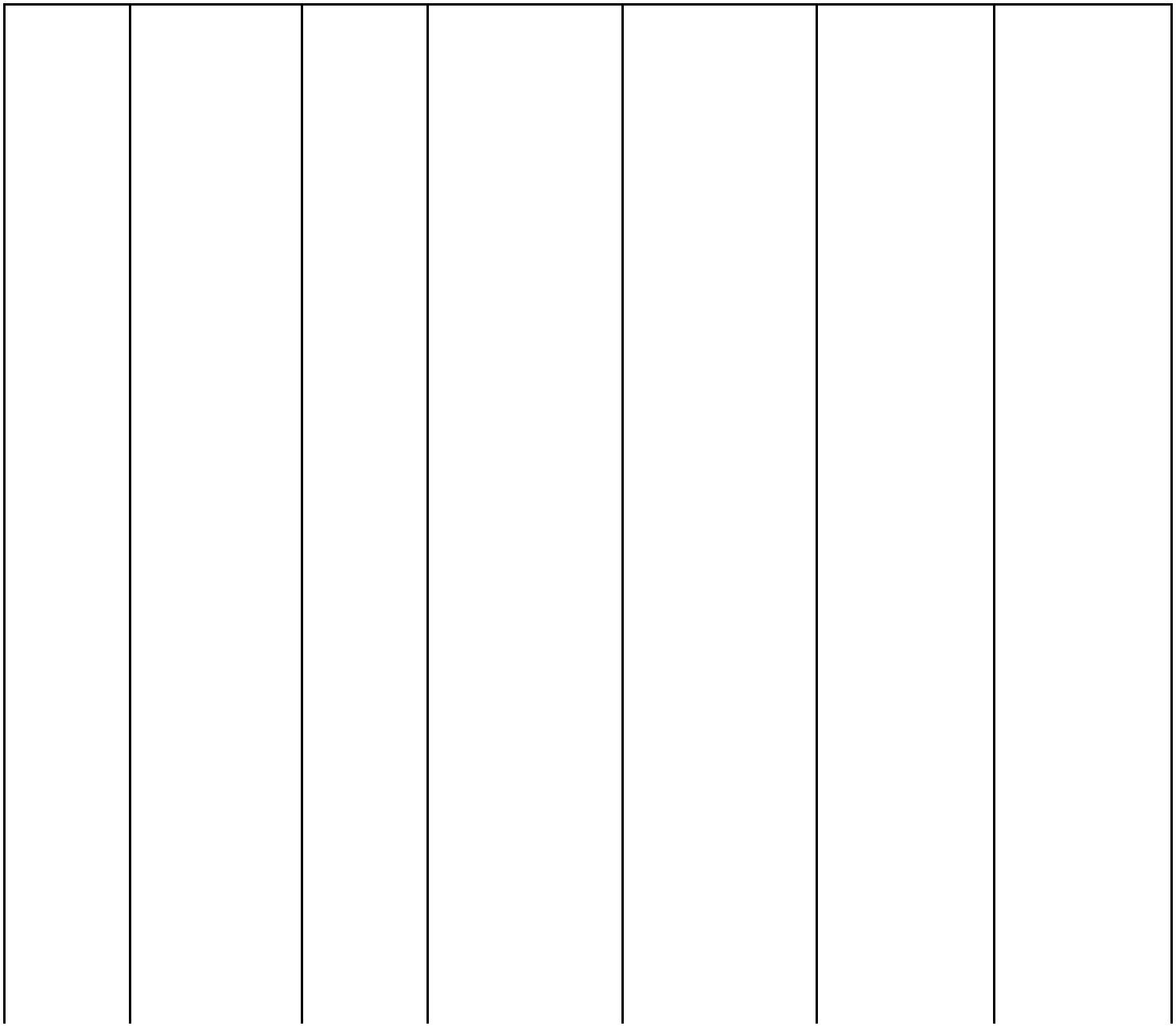GO_REGU  LATION_  OF_TRAN  SPORTER_  ACTIVITY |  | GO_REGULATI  ON_OF_TRAN  SPORTER_AC  TIVITY 286 -0.552434727 -1.492732624 0.013513514 0.509968985 |
| --- | --- | --- |

| 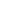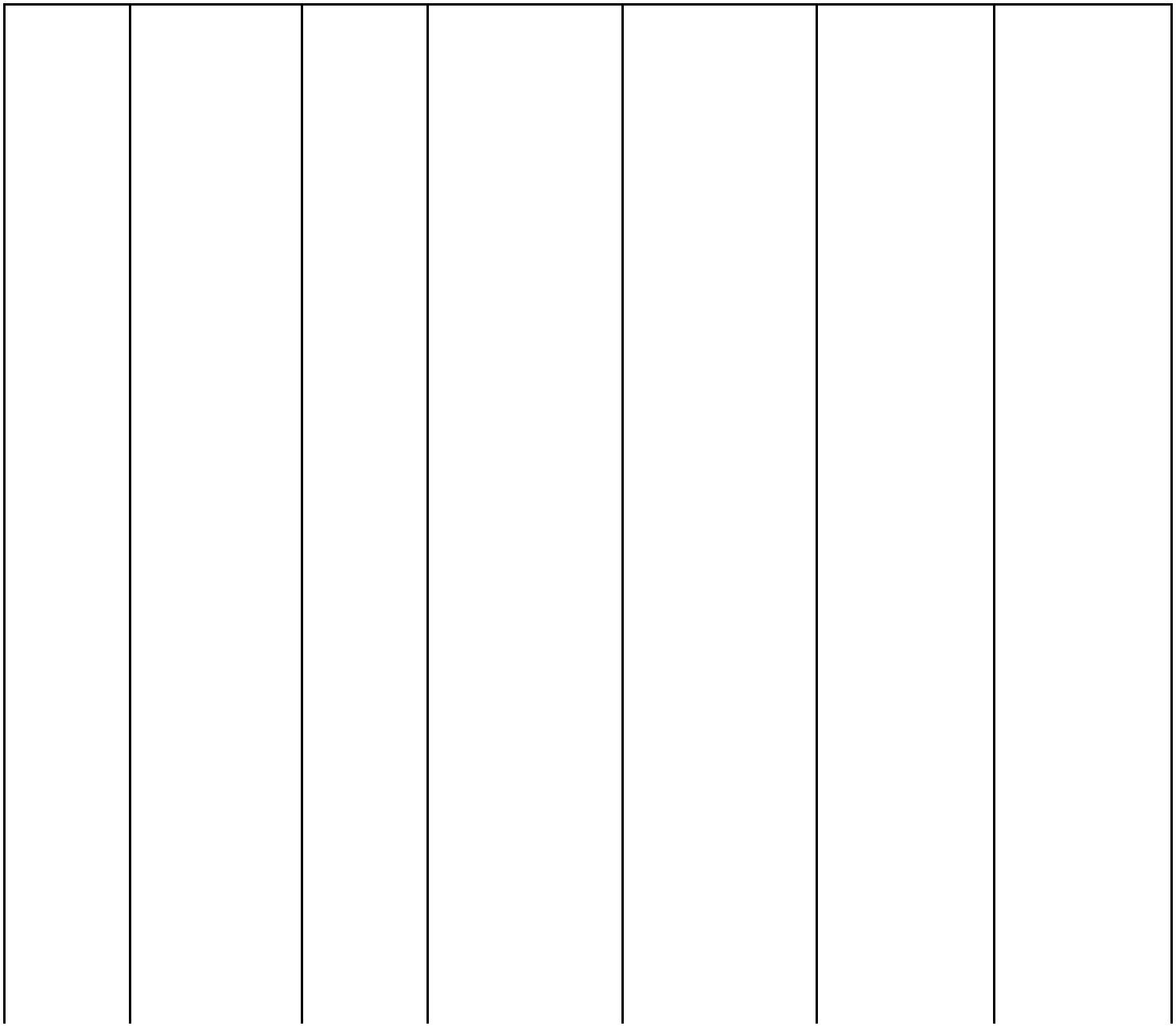GO_VESIC  LE_MEDIA  TED_TRA  NSPORT_I  N_SYNAP  SE |  | GO_VESICLE_  MEDIATED_TR  ANSPORT_IN_  SYNAPSE 214 -0.581150682 -1.538146786 0.013513514 0.509968985 |
| --- | --- | --- |

| 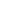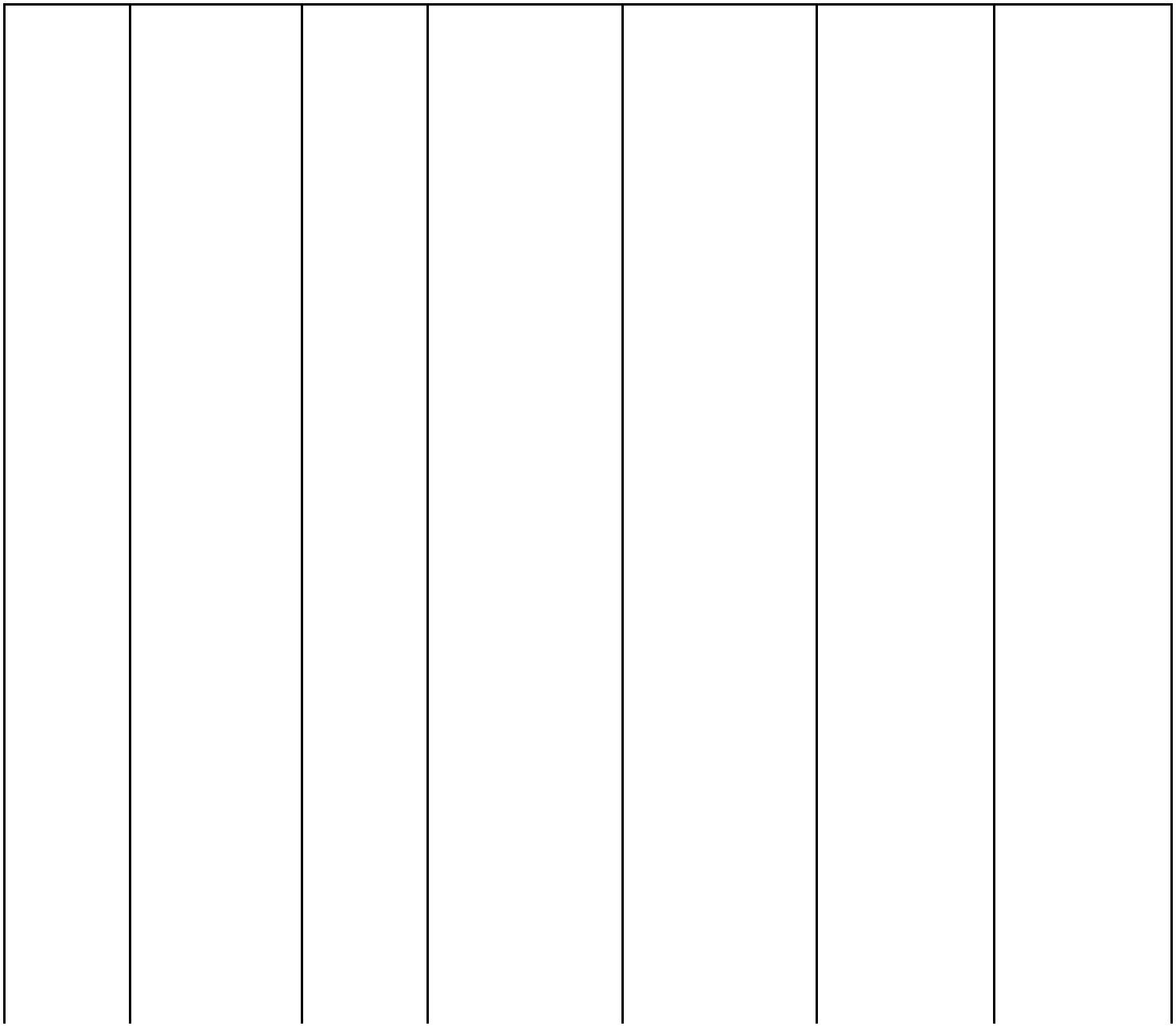GO_COAT  ED_VESIC  LE |  | GO_COATED_  VESICLE 288 -0.481443073 -1.30106197 0.01369863 0.509968985 |
| --- | --- | --- |

| 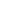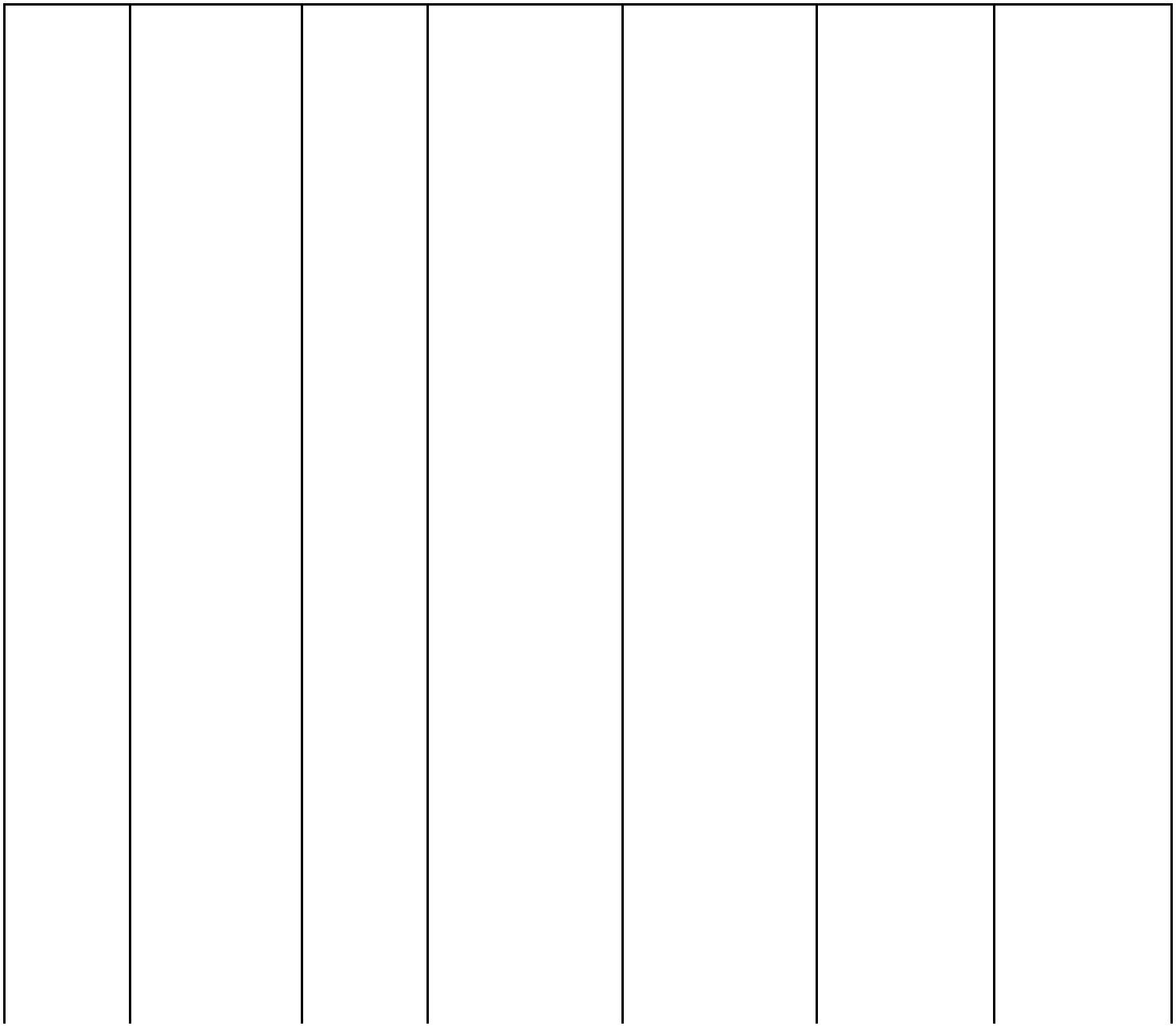GO_DIST  AL_AXON |  | GO_DISTAL_  AXON 291 -0.542828427 -1.470196594 0.01369863 0.509968985 |
| --- | --- | --- |

| 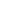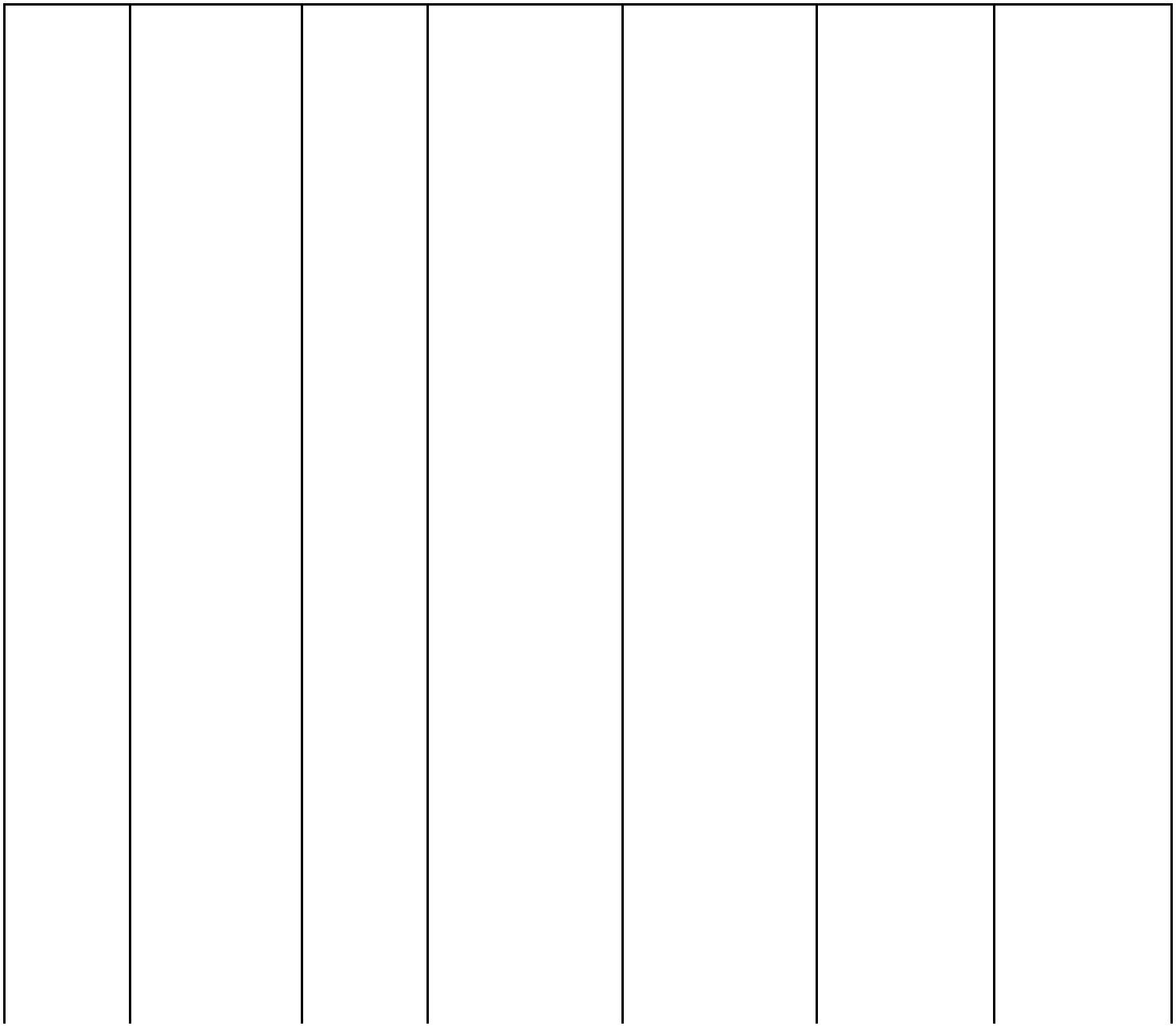GO_EXTR  ACELLUL  AR_STRU  CTURE_O  RGANIZA  TION |  | GO_EXTRACE  LLULAR_STRU  CTURE_ORGA  NIZATION 371 -0.502435208 -1.365091448 0.01369863 0.509968985 |
| --- | --- | --- |

| 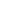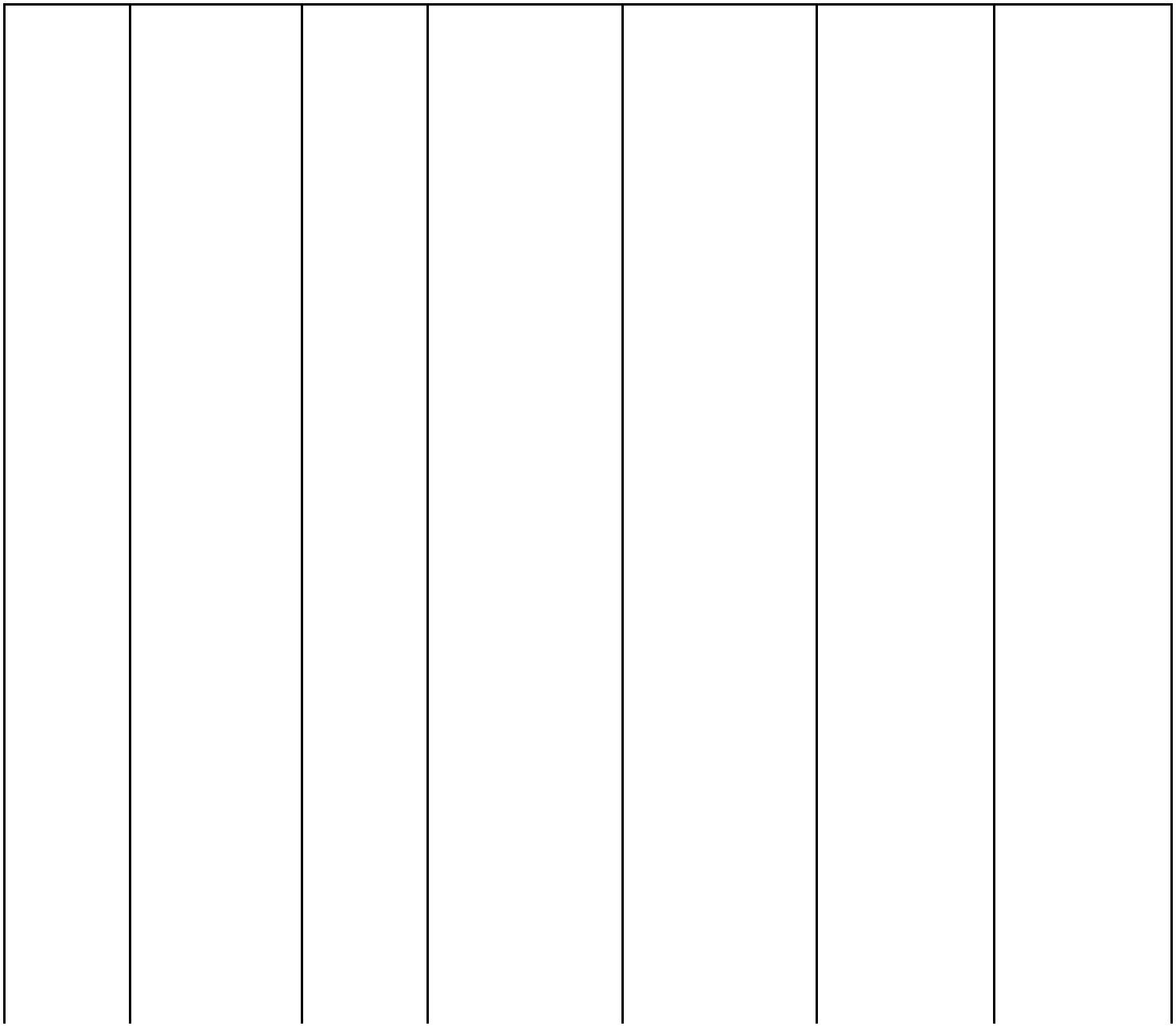GO_HEAR  T_PROCE  SS |  | GO_HEART_P  ROCESS 288 -0.526130235 -1.421825504 0.01369863 0.509968985 |
| --- | --- | --- |

| 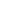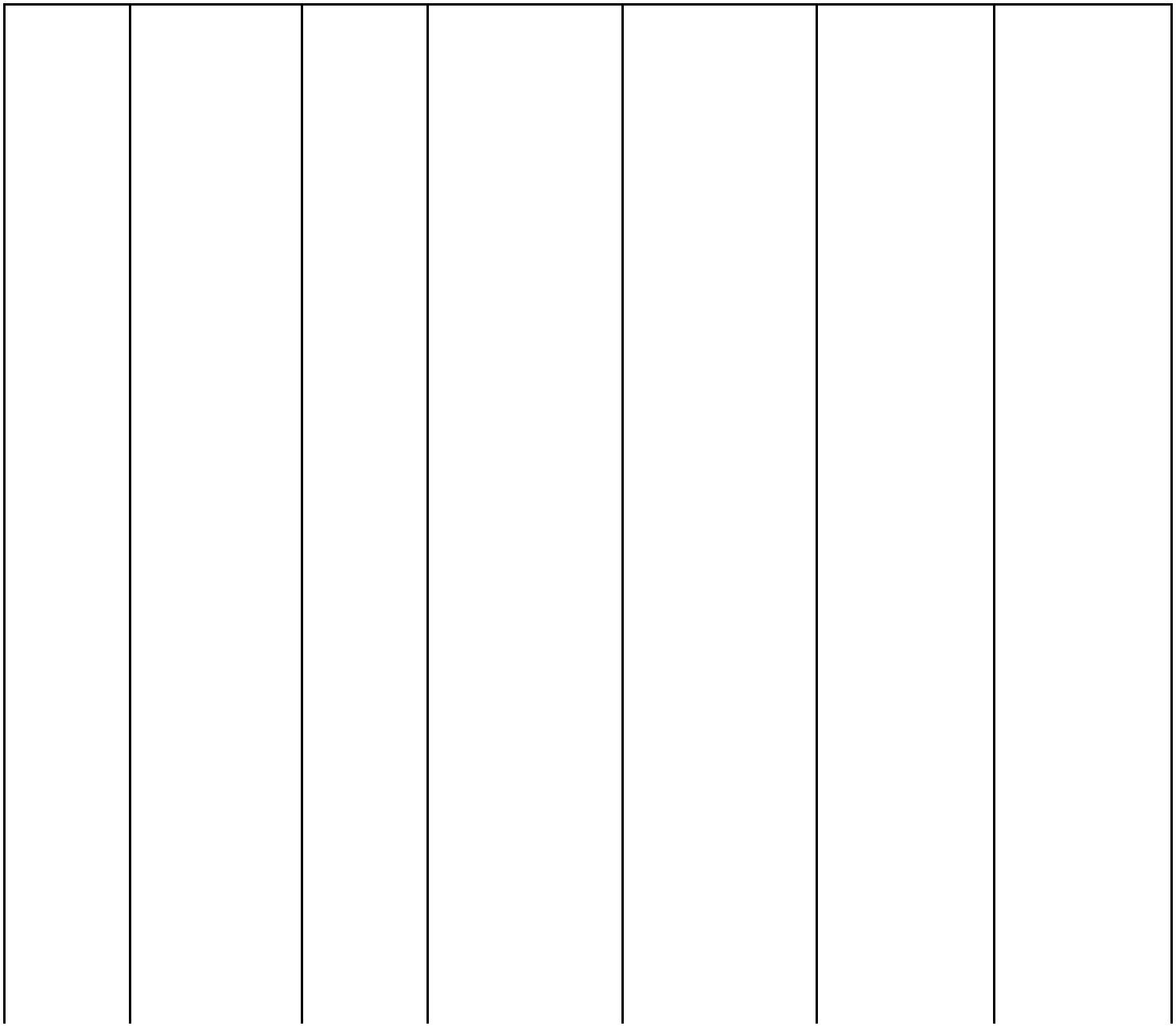GO_MUSC  LE_CELL_  PROLIFER  ATION |  | GO_MUSCLE_  CELL_PROLIFE  RATION 225 -0.518532933 -1.373247969 0.01369863 0.509968985 |
| --- | --- | --- |

| 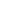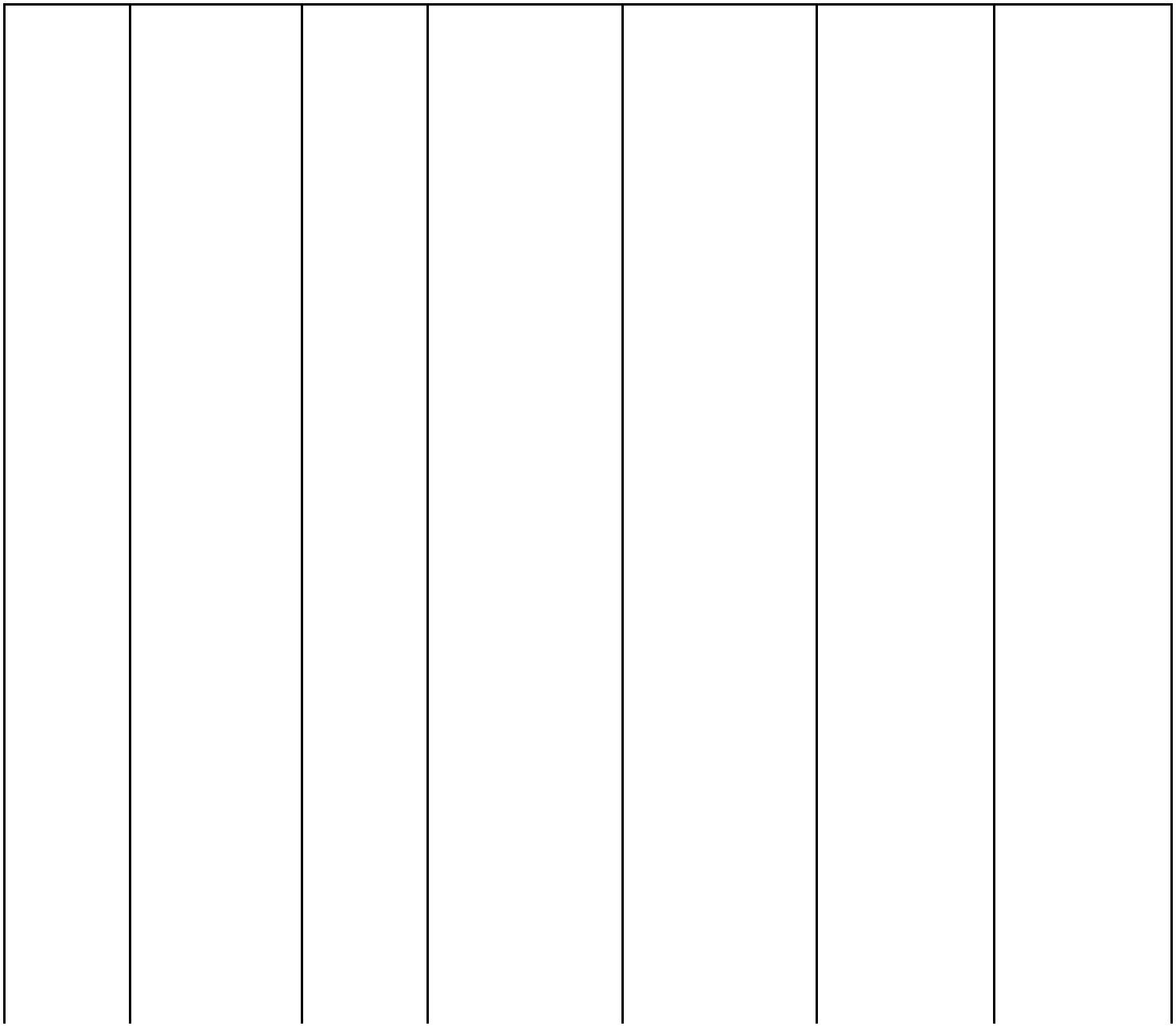GO_MUSC  LE_ORGA  N_DEVEL  OPMENT |  | GO_MUSCLE_  ORGAN_DEVE  LOPMENT 401 -0.48125134 -1.30956831 0.01369863 0.509968985 |
| --- | --- | --- |

| 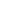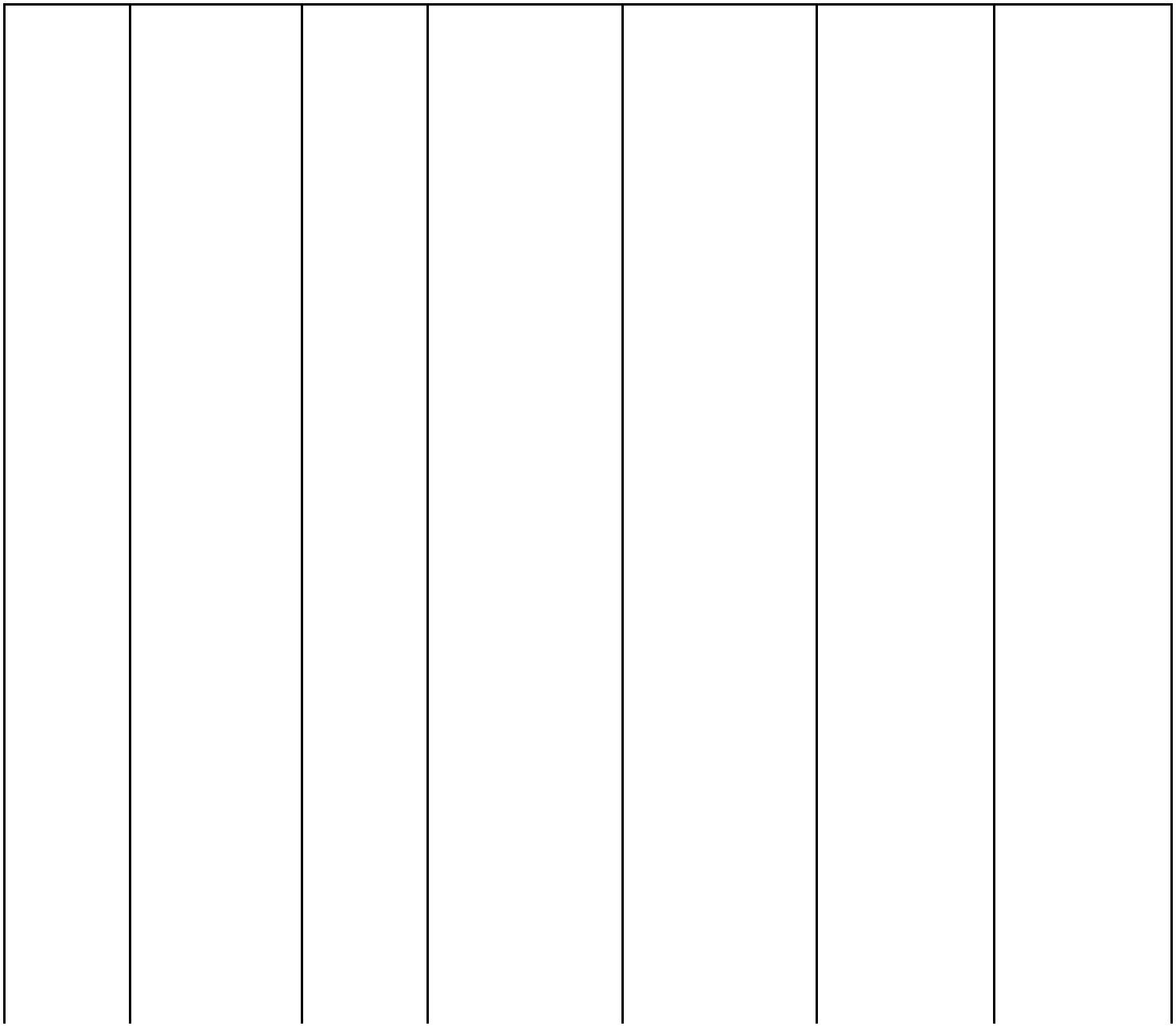GO_NEGA  TIVE_REG  ULATION_  OF_HYDR  OLASE_A  CTIVITY |  | GO_NEGATIV  E_REGULATIO  N_OF_HYDRO  LASE_ACTIVI  TY 451 -0.445197185 -1.221387483 0.01369863 0.509968985 |
| --- | --- | --- |

| 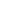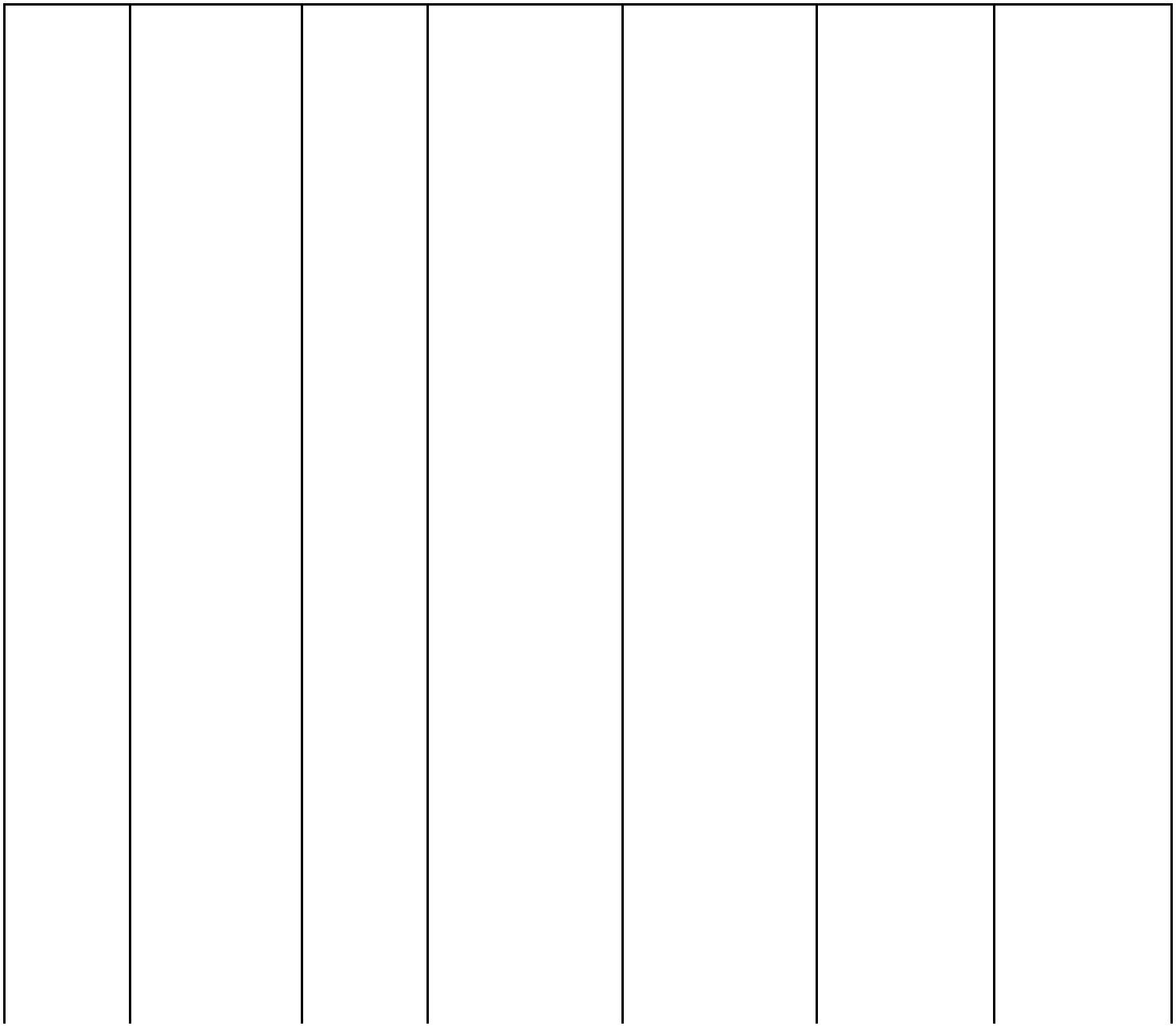GO_NEUR  ON_PROJ  ECTION_G  UIDANCE |  | GO_NEURON_  PROJECTION_  GUIDANCE 282 -0.525903153 -1.411879137 0.01369863 0.509968985 |
| --- | --- | --- |

| 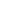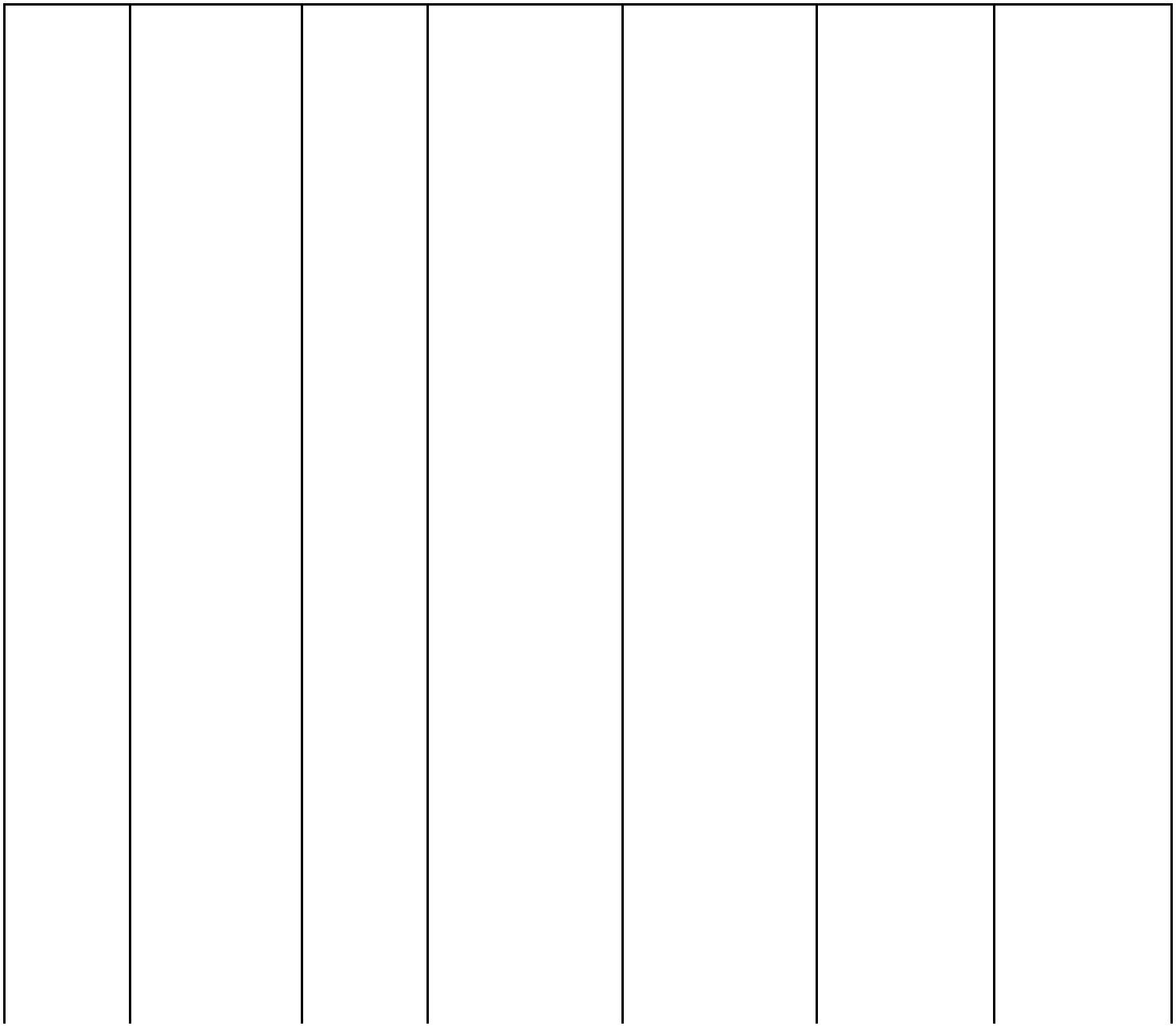GO_NEUR  OTRANSM  ITTER_TR  ANSPORT |  | GO_NEUROTR  ANSMITTER_T  RANSPORT 268 -0.563577629 -1.50877812 0.01369863 0.509968985 |
| --- | --- | --- |

| 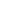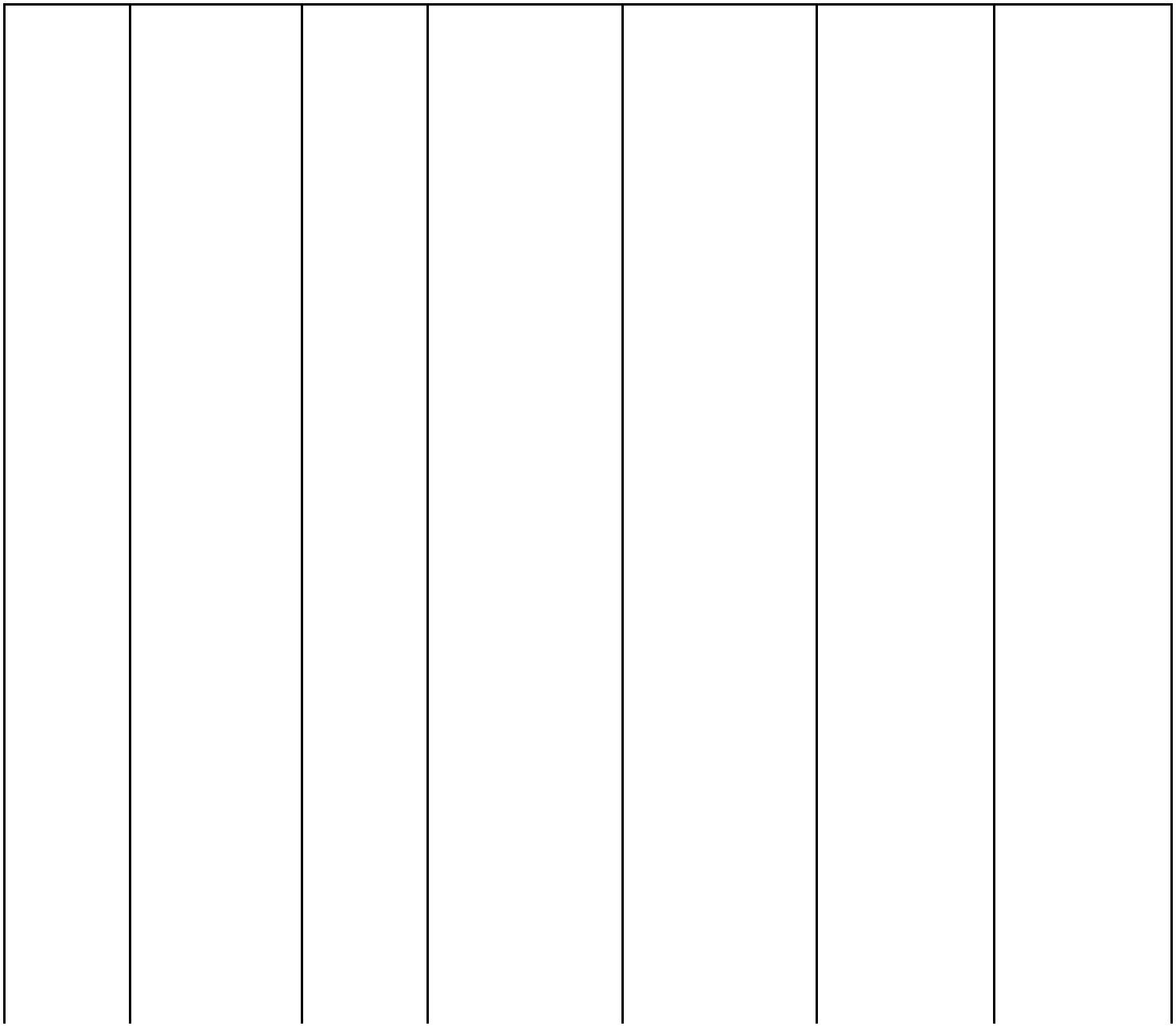GO_PLAS  MA_MEM  BRANE_SI  GNALING  _RECEPTO  R_COMPL  EX |  | GO_PLASMA_  MEMBRANE_S  IGNALING_RE  CEPTOR_COM  PLEX 293 -0.753787793 -2.039622481 0.01369863 0.509968985 |
| --- | --- | --- |

| 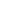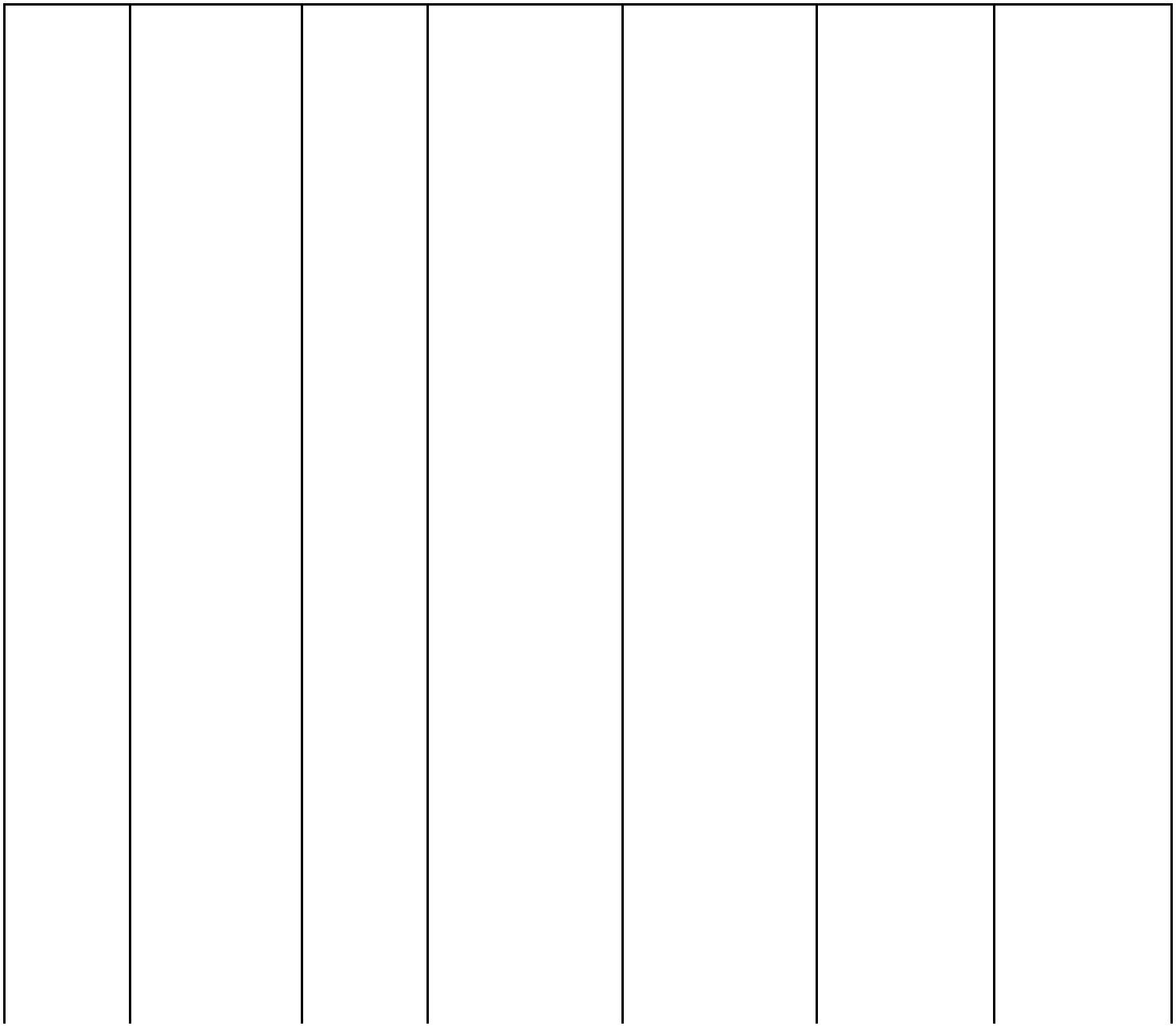GO_POSIT  IVE_REGU  LATION_  OF_CELL_  ADHESIO  N |  | GO_POSITIVE  _REGULATION  _OF_CELL_AD  HESION 398 -0.566223703 -1.536534308 0.01369863 0.509968985 |
| --- | --- | --- |

| 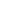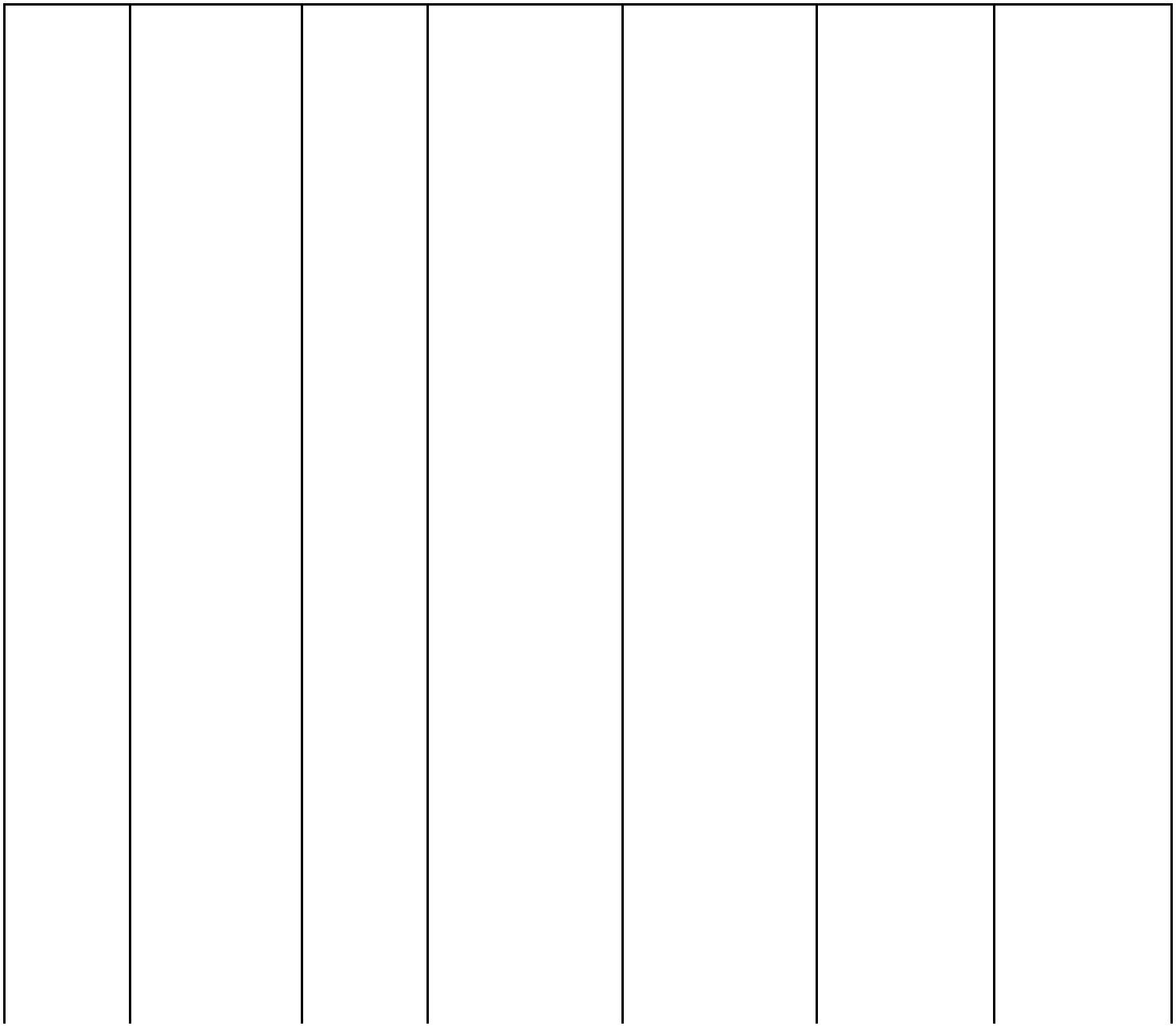GO_POSIT  IVE_REGU  LATION_  OF_CYTO  KINE_PRO  DUCTION |  | GO_POSITIVE  _REGULATION  _OF_CYTOKIN  E_PRODUCTIO  N 463 -0.5140885 -1.409346655 0.01369863 0.509968985 |
| --- | --- | --- |

| 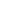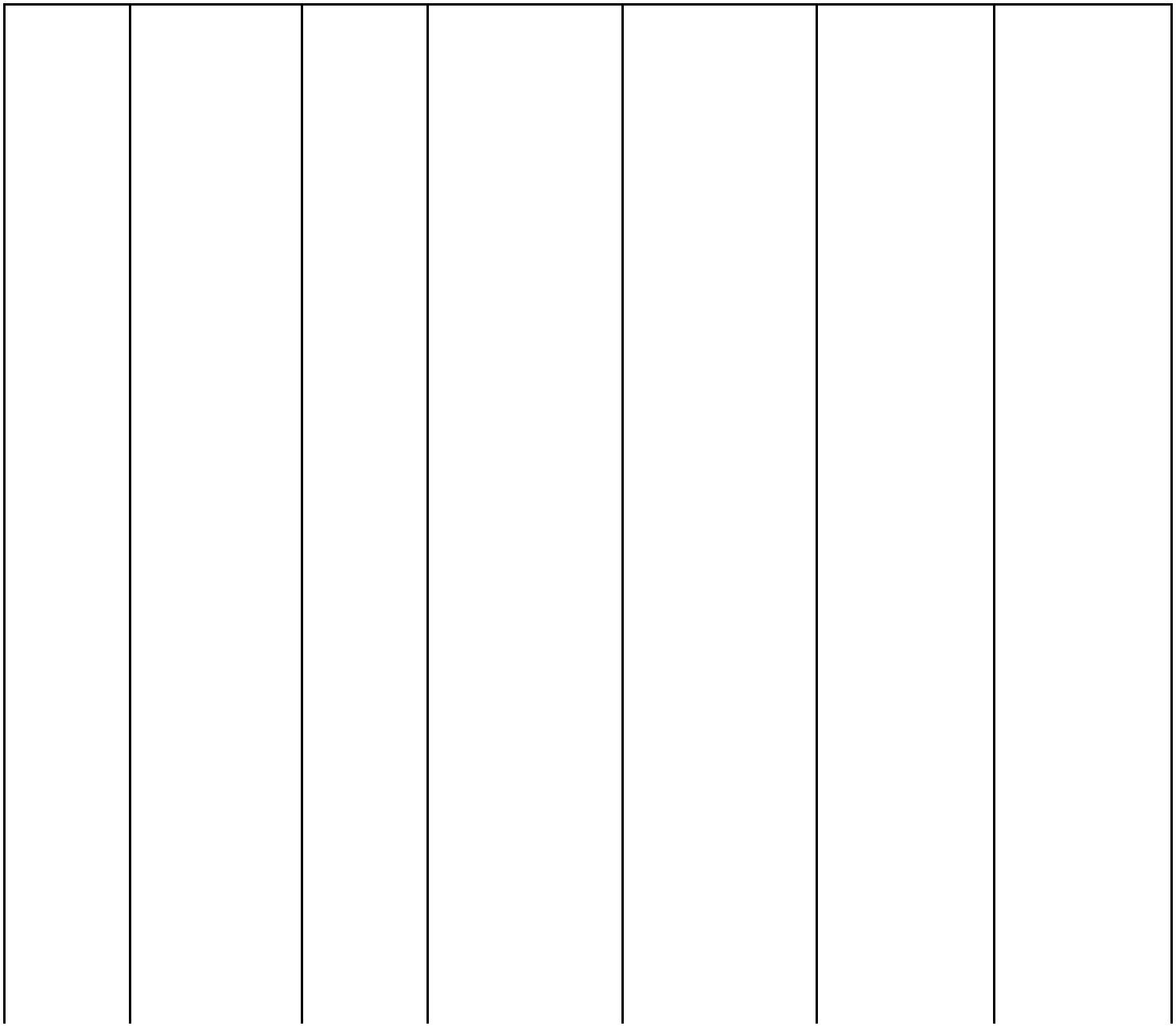GO_POSIT  IVE_REGU  LATION_  OF_ESTA  BLISHMEN  T_OF_PR  OTEIN_LO  CALIZATI  ON |  | GO_POSITIVE  _REGULATION  _OF_ESTABLI  SHMENT_OF_  PROTEIN_LOC  ALIZATION 458 -0.471331171 -1.292774747 0.01369863 0.509968985 |
| --- | --- | --- |

| 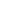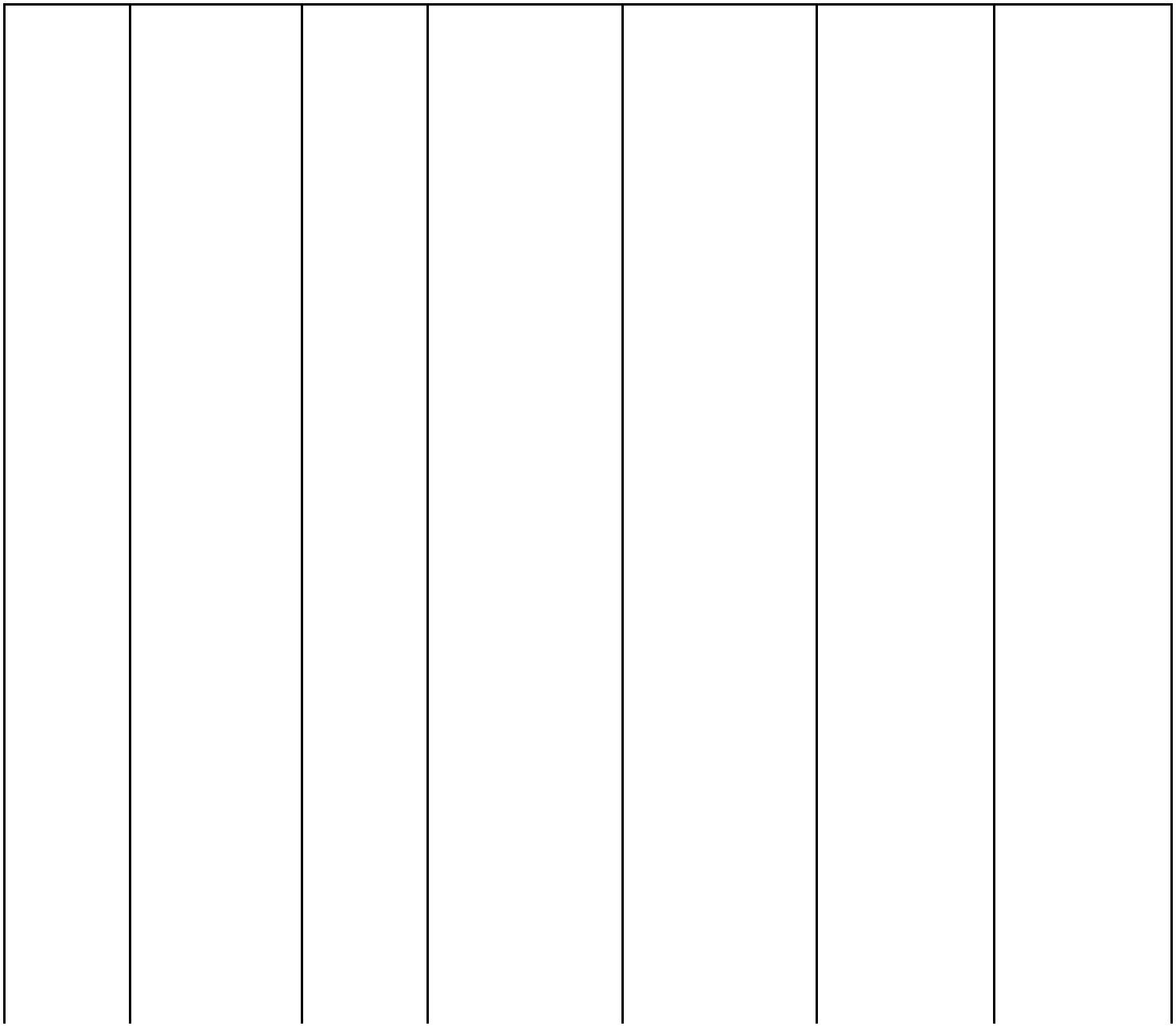GO_POSIT  IVE_REGU  LATION_  OF_IMMU  NE_EFFEC  TOR_PRO  CESS |  | GO_POSITIVE  _REGULATION  _OF_IMMUNE  _EFFECTOR_P  ROCESS 212 -0.587324229 -1.550054858 0.01369863 0.509968985 |
| --- | --- | --- |

| 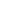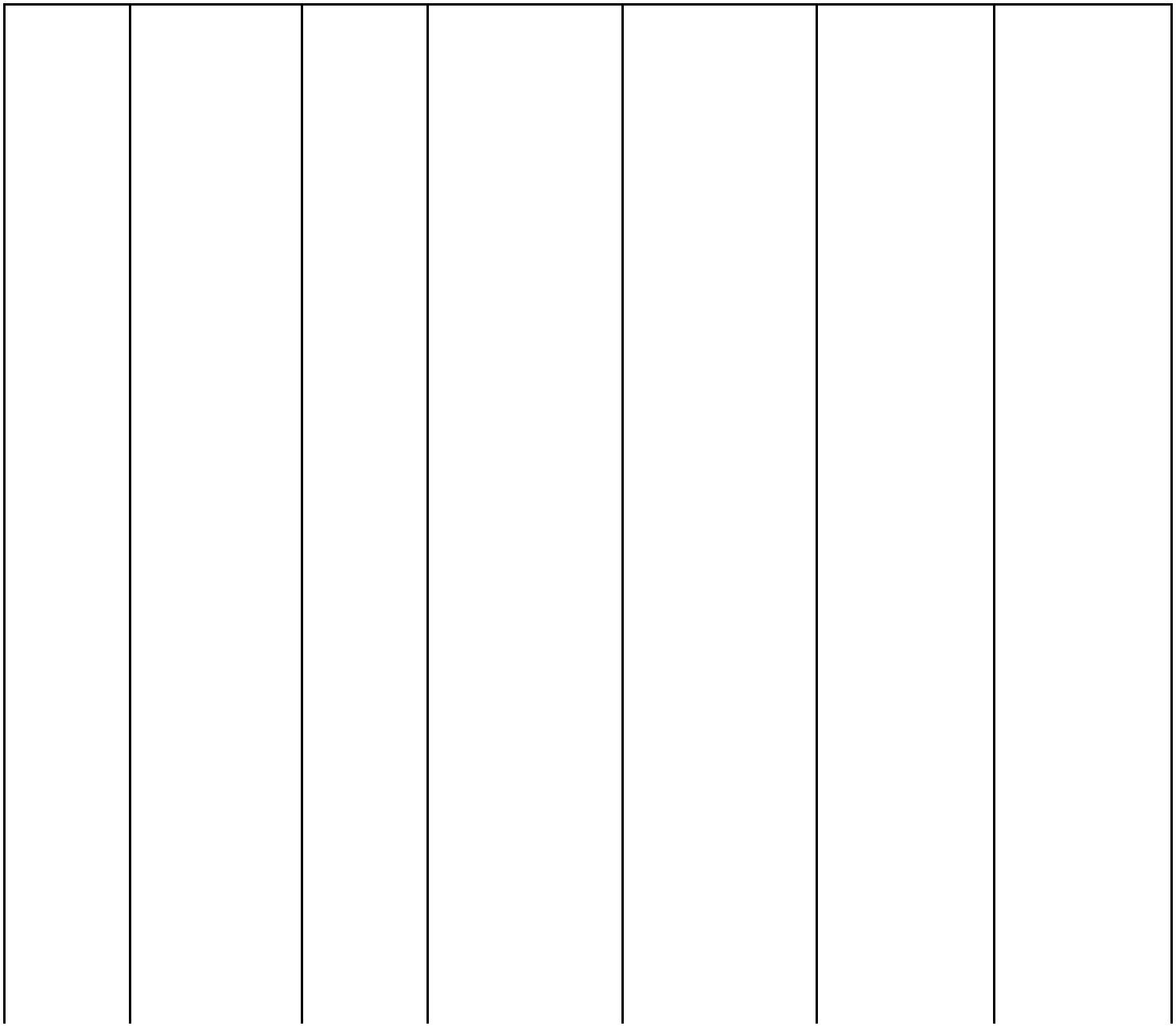GO_POSIT  IVE_REGU  LATION_  OF_LEUK  OCYTE_C  ELL_CELL  _ADHESIO  N |  | GO_POSITIVE  _REGULATION  _OF_LEUKOC  YTE_CELL_CE  LL_ADHESION 216 -0.619009038 -1.63028896 0.01369863 0.509968985 |
| --- | --- | --- |

| 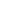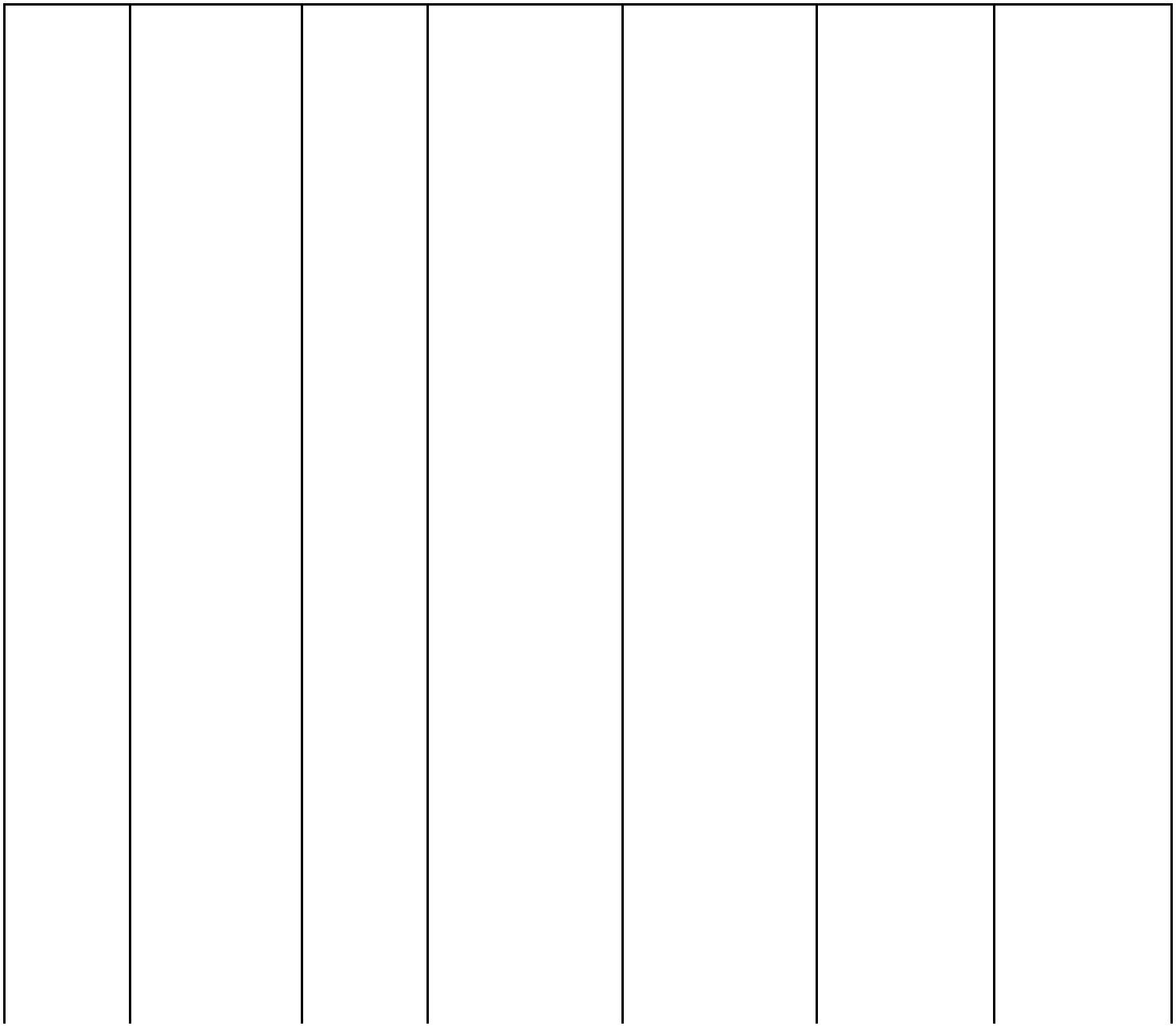GO_POSIT  IVE_REGU  LATION_  OF_PEPTI  DE_SECRE  TION |  | GO_POSITIVE  _REGULATION  _OF_PEPTIDE_  SECRETION 291 -0.509920859 -1.381069732 0.01369863 0.509968985 |
| --- | --- | --- |

| 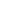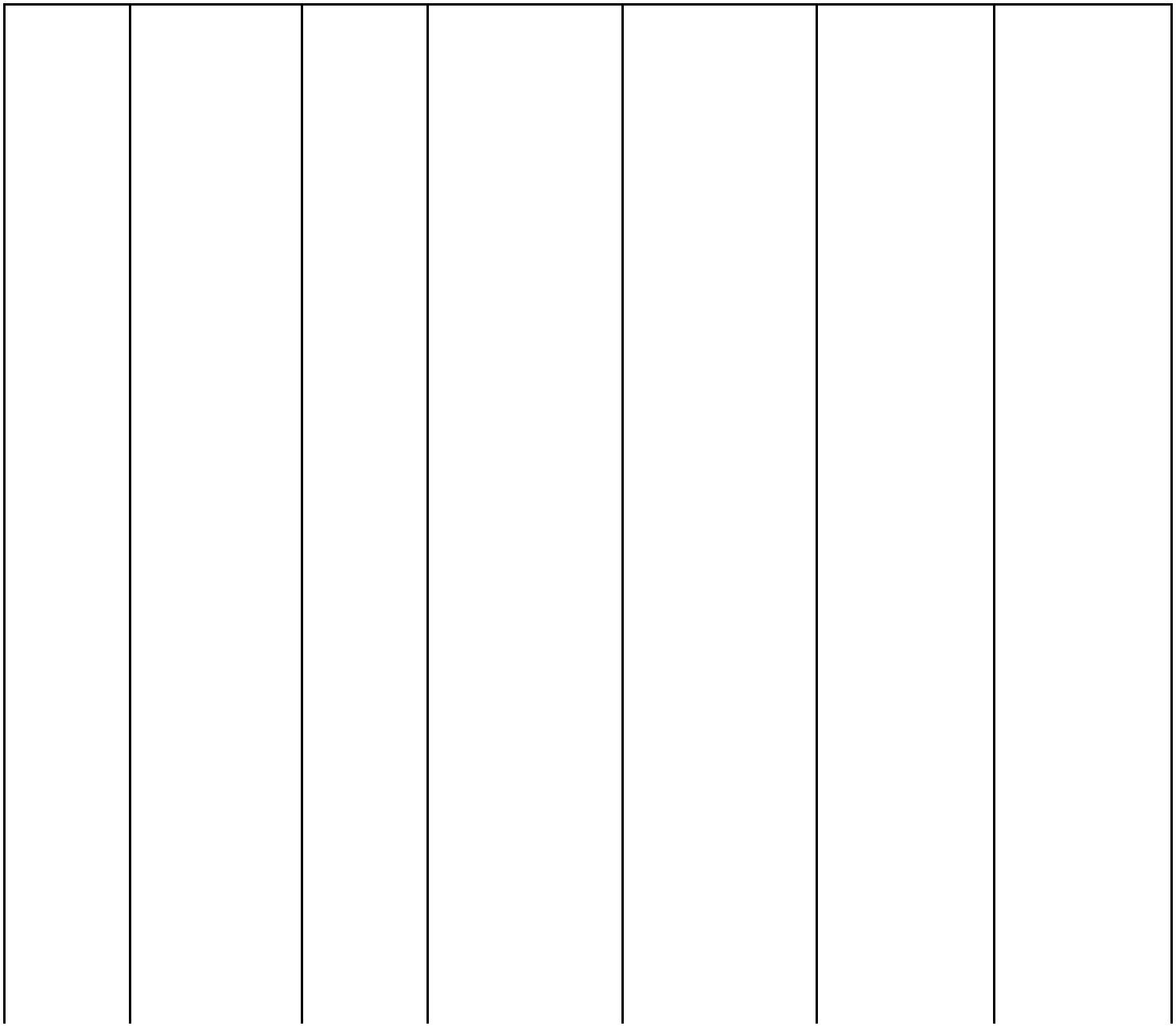GO_POSIT  IVE_REGU  LATION_  OF_RESPO  NSE_TO_  BIOTIC_S  TIMULUS |  | GO_POSITIVE  _REGULATION  _OF_RESPONS  E_TO_BIOTIC  _STIMULUS 397 -0.469470652 -1.27484683 0.01369863 0.509968985 |
| --- | --- | --- |

| 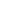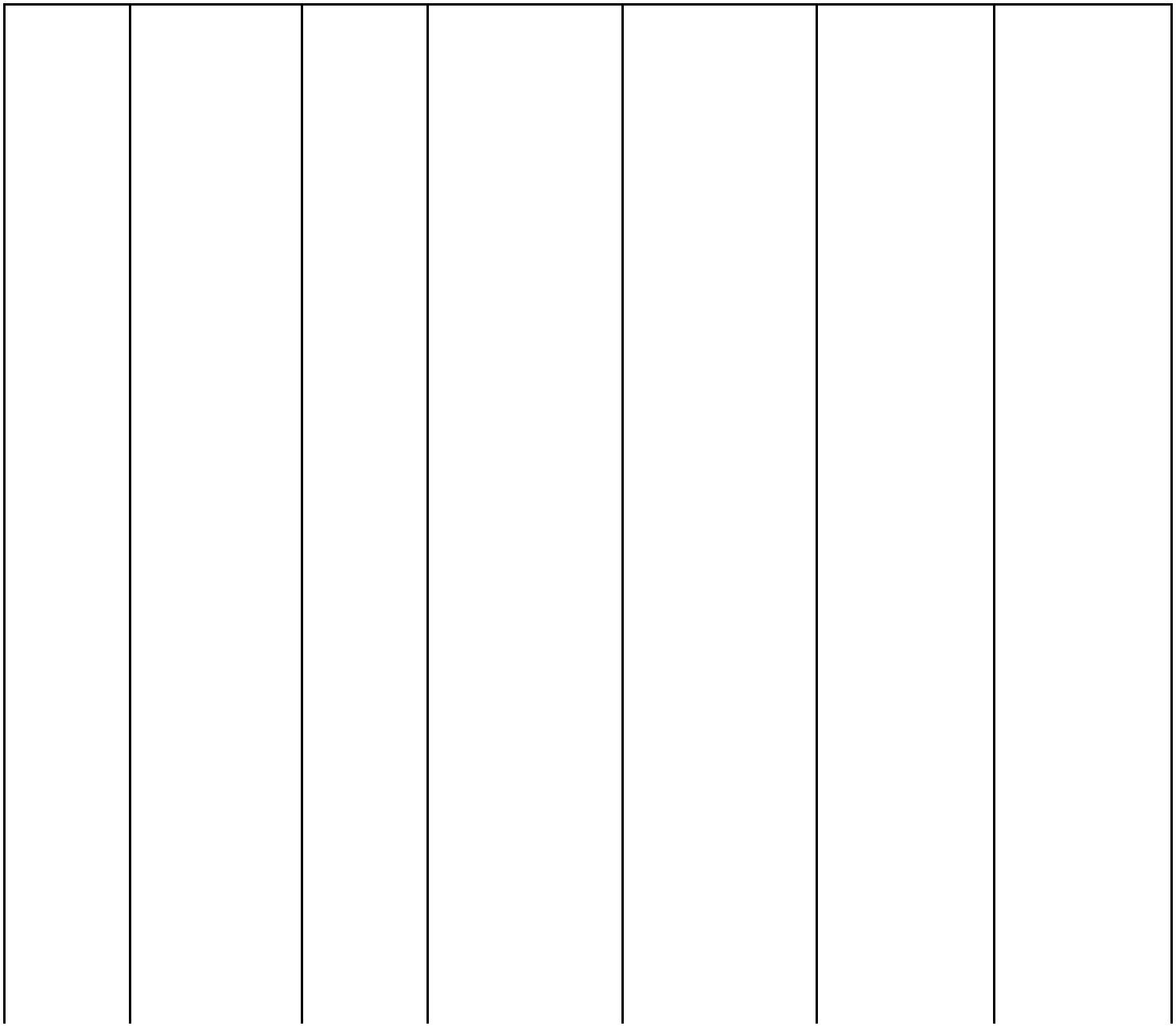GO_REGU  LATION_  OF_BLOO  D_CIRCUL  ATION |  | GO_REGULATI  ON_OF_BLOO  D_CIRCULATI  ON 291 -0.554006856 -1.500472251 0.01369863 0.509968985 |
| --- | --- | --- |

| 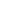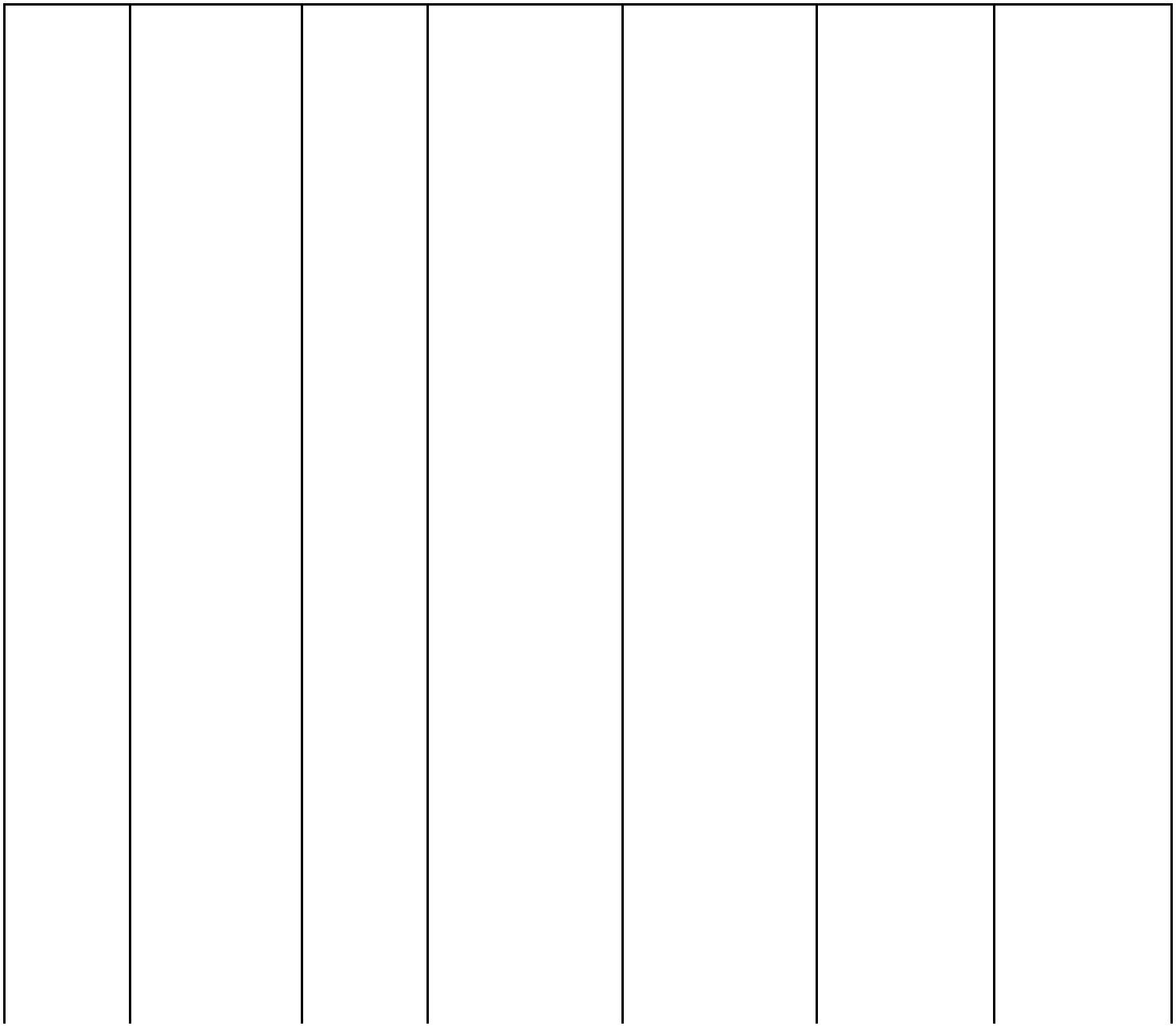GO_REGU  LATION_  OF_EXOC  YTOSIS |  | GO_REGULATI  ON_OF_EXOC  YTOSIS 216 -0.526197807 -1.385851293 0.01369863 0.509968985 |
| --- | --- | --- |

| 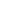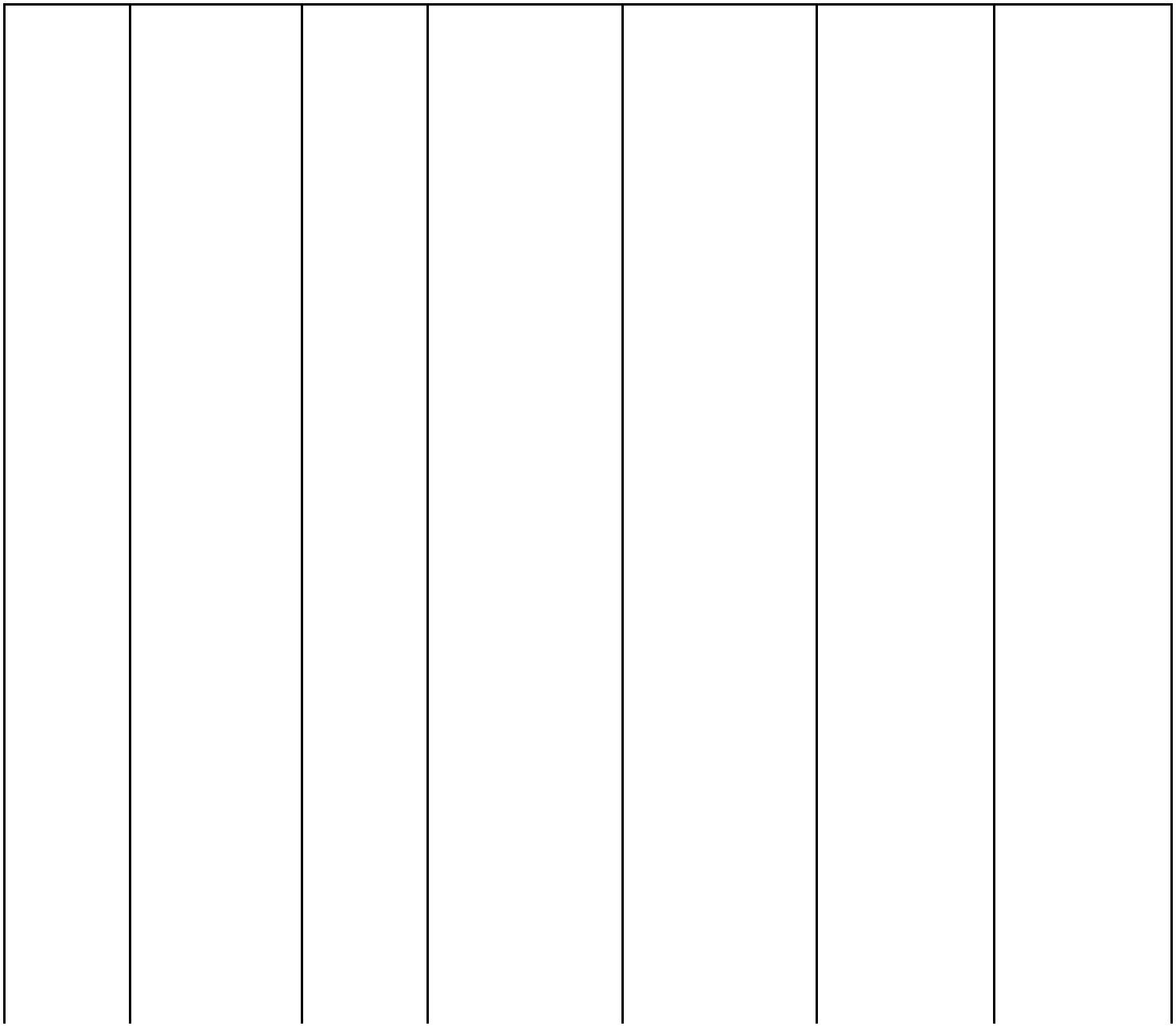GO_REGU  LATION_  OF_INFLA  MMATOR  Y_RESPO  NSE |  | GO_REGULATI  ON_OF_INFLA  MMATORY_R  ESPONSE 370 -0.504868552 -1.373221496 0.01369863 0.509968985 |
| --- | --- | --- |

| 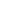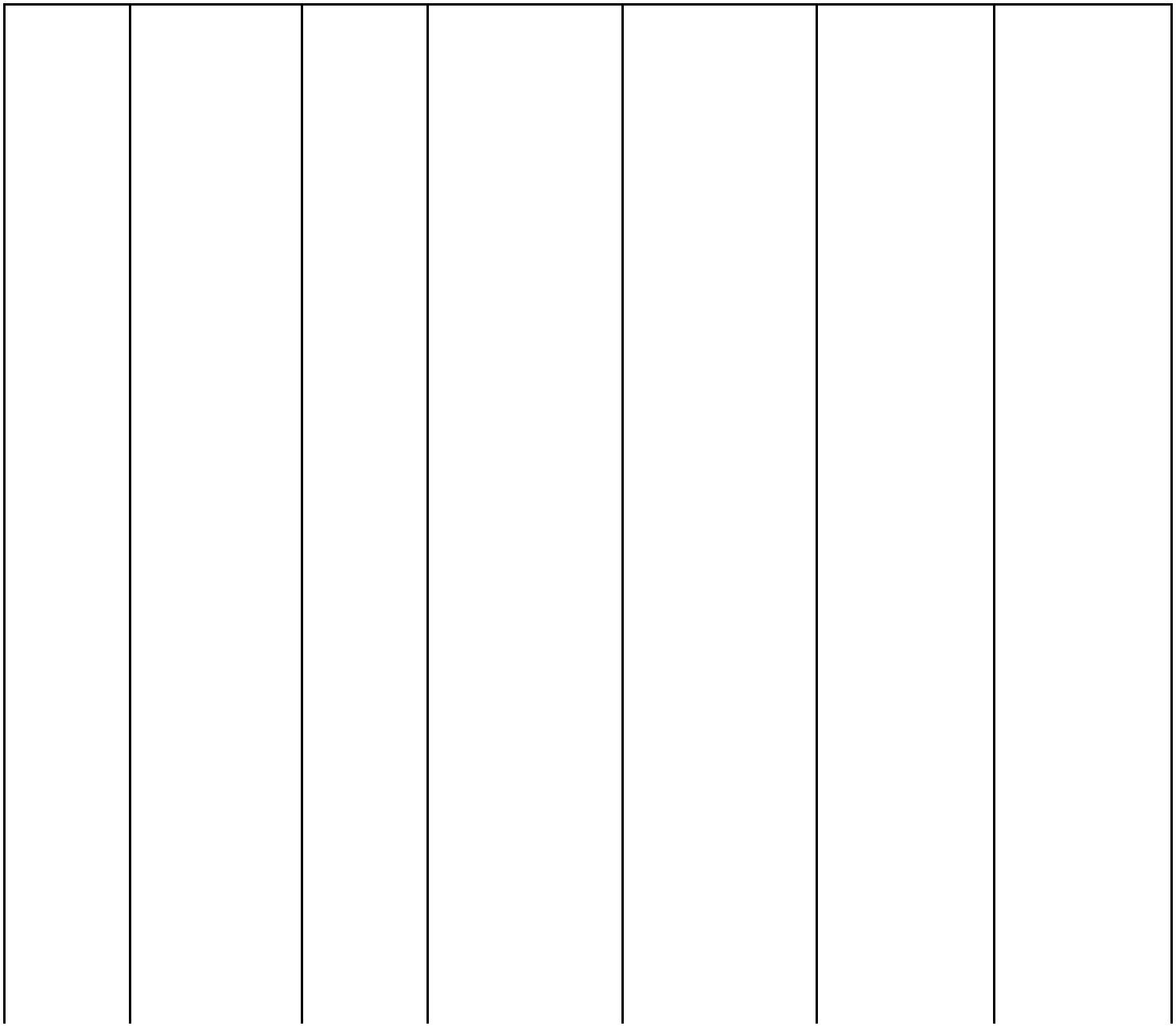GO_REGU  LATION_  OF_LEUK  OCYTE_P  ROLIFERA  TION |  | GO_REGULATI  ON_OF_LEUK  OCYTE_PROLI  FERATION 224 -0.553295147 -1.464068728 0.01369863 0.509968985 |
| --- | --- | --- |

| GO_REGU  LATION_  OF_META  L_ION_TR  ANSPORT |  | GO_REGULATI  ON_OF_META  L_ION_TRANS  PORT 394 -0.480605177 -1.300957541 0.01369863 0.509968985 |
| --- | --- | --- |

| GO_SENS  ORY_PER  CEPTION_  OF_LIGHT  _STIMULU  S |  | GO_SENSORY  _PERCEPTION  _OF_LIGHT_S  TIMULUS 221 -0.635615841 -1.682719927 0.01369863 0.509968985 |
| --- | --- | --- |

| GO_SIGN  AL_RELEA  SE |  | GO_SIGNAL_R  ELEASE 458 -0.462074498 -1.267385394 0.01369863 0.509968985 |
| --- | --- | --- |

| GO_STRI  ATED_MU  SCLE_CEL  L_DIFFERE  NTIATION |  | GO_STRIATED  _MUSCLE_CE  LL_DIFFERENT  IATION 282 -0.537893398 -1.444069049 0.01369863 0.509968985 |
| --- | --- | --- |

| GO_T_CE  LL_DIFFER  ENTIATIO  N |  | GO_T_CELL_D  IFFERENTIATI  ON 239 -0.589534646 -1.55979786 0.01369863 0.509968985 |
| --- | --- | --- |

| GO_CATI  ON_CHAN  NEL_COM  PLEX |  | GO_CATION_  CHANNEL_CO  MPLEX 219 -0.598662195 -1.575060382 0.013888889 0.509968985 |
| --- | --- | --- |

| GO_COGN  ITION |  | GO_COGNITIO  N 298 -0.526692983 -1.425521951 0.013888889 0.509968985 |
| --- | --- | --- |

| GO_EXOC  YTIC_VES  ICLE |  | GO_EXOCYTI  C_VESICLE 219 -0.517617583 -1.361834696 0.013888889 0.509968985 |
| --- | --- | --- |

| GO_EXTE  RNAL_SID  E_OF_PLA  SMA_ME  MBRANE |  | GO_EXTERNA  L_SIDE_OF_PL  ASMA_MEMB  RANE 384 -0.638608096 -1.721541194 0.013888889 0.509968985 |
| --- | --- | --- |

| GO_FC_R  ECEPTOR_  SIGNALIN  G_PATH  WAY |  | GO_FC_RECEP  TOR_SIGNALI  NG_PATHWA  Y 240 -0.658733878 -1.740985045 0.013888889 0.509968985 |
| --- | --- | --- |

| GO_MUSC  LE_CELL_  DIFFEREN  TIATION |  | GO_MUSCLE_  CELL_DIFFERE  NTIATION 369 -0.480291308 -1.305138761 0.013888889 0.509968985 |
| --- | --- | --- |

| GO_MUSC  LE_SYSTE  M_PROCE  SS |  | GO_MUSCLE_  SYSTEM_PRO  CESS 456 -0.488592095 -1.337605167 0.013888889 0.509968985 |
| --- | --- | --- |

| GO_PEPTI  DE_BINDI  NG |  | GO_PEPTIDE_  BINDING 296 -0.487490064 -1.319532433 0.013888889 0.509968985 |
| --- | --- | --- |

| GO_POSIT  IVE_REGU  LATION_  OF_CELL_  ACTIVATI  ON |  | GO_POSITIVE  _REGULATION  _OF_CELL_A  CTIVATION 386 -0.639044022 -1.721869346 0.013888889 0.509968985 |
| --- | --- | --- |

| GO_POTA  SSIUM_IO  N_TRANS  PORT |  | GO_POTASSI  UM_ION_TRA  NSPORT 241 -0.51704756 -1.372952194 0.013888889 0.509968985 |
| --- | --- | --- |

| GO_PROD  UCTION_  OF_MOLE  CULAR_M  EDIATOR_  OF_IMMU  NE_RESPO  NSE |  | GO_PRODUCT  ION_OF_MOL  ECULAR_MEDI  ATOR_OF_IM  MUNE_RESPO  NSE 298 -0.68414551 -1.851675404 0.013888889 0.509968985 |
| --- | --- | --- |

| GO_REGU  LATION_  OF_CALCI  UM_ION_  TRANSPO  RT |  | GO_REGULATI  ON_OF_CALC  IUM_ION_TRA  NSPORT 255 -0.506492977 -1.348038359 0.013888889 0.509968985 |
| --- | --- | --- |

| GO_REGU  LATION_  OF_IMMU  NE_EFFEC  TOR_PRO  CESS |  | GO_REGULATI  ON_OF_IMMU  NE_EFFECTOR  _PROCESS 454 -0.584807016 -1.601861098 0.013888889 0.509968985 |
| --- | --- | --- |

| GO_SENS  ORY_SYS  TEM_DEV  ELOPMEN  T |  | GO_SENSORY  _SYSTEM_DE  VELOPMENT 368 -0.463533655 -1.259133375 0.013888889 0.509968985 |
| --- | --- | --- |

| GO_SYNA  PSE_ORG  ANIZATIO  N |  | GO_SYNAPSE  _ORGANIZATI  ON 414 -0.514618544 -1.408197514 0.013888889 0.509968985 |
| --- | --- | --- |

| GO_ANIO  N_TRANS  MEMBRAN  E_TRANS  PORT |  | GO_ANION_T  RANSMEMBR  ANE_TRANSP  ORT 294 -0.485635593 -1.311537482 0.014084507 0.509968985 |
| --- | --- | --- |

| GO_ANTI  GEN_RECE  PTOR_ME  DIATED_S  IGNALING  _PATHW  AY |  | GO_ANTIGEN  _RECEPTOR_  MEDIATED_SI  GNALING_PA  THWAY 303 -0.65708257 -1.784293614 0.014084507 0.509968985 |
| --- | --- | --- |

| GO_B_CE  LL_ACTIV  ATION |  | GO_B_CELL_  ACTIVATION 306 -0.65036 -1.764477045 0.014084507 0.509968985 |
| --- | --- | --- |

| GO_CELL  _PROJECT  ION_MEM  BRANE |  | GO_CELL_PRO  JECTION_ME  MBRANE 326 -0.464260144 -1.257718799 0.014084507 0.509968985 |
| --- | --- | --- |

| GO_CELL  _RECOGNI  TION |  | GO_CELL_REC  OGNITION 207 -0.632791162 -1.65602119 0.014084507 0.509968985 |
| --- | --- | --- |

| GO_G_PR  OTEIN_CO  UPLED_RE  CEPTOR_  SIGNALIN  G_PATH  WAY_CO  UPLED_TO  _CYCLIC_  NUCLEOTI  DE_SECO  ND_MESS  ENGER |  | GO_G_PROTEI  N_COUPLED_R  ECEPTOR_SIG  NALING_PATH  WAY_COUPL  ED_TO_CYCLI  C_NUCLEOTID  E_SECOND_M  ESSENGER 253 -0.55186485 -1.467373817 0.014084507 0.509968985 |
| --- | --- | --- |

| GO_GATE  D_CHANN  EL_ACTIV  ITY |  | GO_GATED_C  HANNEL_ACTI  VITY 343 -0.553960809 -1.505763309 0.014084507 0.509968985 |
| --- | --- | --- |

| GO_INTRI  NSIC_CO  MPONENT  _OF_ORG  ANELLE_  MEMBRAN  E |  | GO_INTRINSIC  _COMPONENT  _OF_ORGANE  LLE_MEMBRA  NE 380 -0.472171663 -1.273218665 0.014084507 0.509968985 |
| --- | --- | --- |

| GO_LEUK  OCYTE_P  ROLIFERA  TION |  | GO_LEUKOCY  TE_PROLIFER  ATION 300 -0.533602261 -1.442372583 0.014084507 0.509968985 |
| --- | --- | --- |

| GO_RECE  PTOR_ME  DIATED_E  NDOCYTO  SIS |  | GO_RECEPTO  R_MEDIATED_  ENDOCYTOSI  S 318 -0.602028159 -1.63410678 0.014084507 0.509968985 |
| --- | --- | --- |

| GO_REGU  LATION_  OF_CYTO  SOLIC_C  ALCIUM_I  ON_CONC  ENTRATIO  N |  | GO_REGULATI  ON_OF_CYTO  SOLIC_CALCI  UM_ION_CON  CENTRATION 366 -0.586143515 -1.586176383 0.014084507 0.509968985 |
| --- | --- | --- |

| GO_REGU  LATION_  OF_NEUR  OTRANSM  ITTER_LE  VELS |  | GO_REGULATI  ON_OF_NEUR  OTRANSMITT  ER_LEVELS 350 -0.503227433 -1.361883343 0.014084507 0.509968985 |
| --- | --- | --- |

| GO_RESP  ONSE_TO  _TUMOR_  NECROSIS  _FACTOR |  | GO_RESPONS  E_TO_TUMOR  _NECROSIS_F  ACTOR 307 -0.520243537 -1.410719249 0.014084507 0.509968985 |
| --- | --- | --- |

| GO_T_CE  LL_ACTIV  ATION |  | GO_T_CELL_  ACTIVATION 467 -0.562368661 -1.534297167 0.014084507 0.509968985 |
| --- | --- | --- |

| GO_ACTI  VE_TRAN  SMEMBRA  NE_TRAN  SPORTER_  ACTIVITY |  | GO_ACTIVE_  TRANSMEMBR  ANE_TRANSP  ORTER_ACTIV  ITY 347 -0.448368543 -1.21430509 0.014285714 0.509968985 |
| --- | --- | --- |

| GO_ADAP  TIVE_IMM  UNE_RESP  ONSE_BA  SED_ON_  SOMATIC  _RECOMBI  NATION_  OF_IMMU  NE_RECEP  TORS_BUI  LT_FROM  _IMMUNO  GLOBULIN  _SUPERFA  MILY_DO  MAINS |  | GO_ADAPTIV  E_IMMUNE_RE  SPONSE_BAS  ED_ON_SOMA  TIC_RECOMBI  NATION_OF_I  MMUNE_RECE  PTORS_BUILT  _FROM_IMMU  NOGLOBULIN_  SUPERFAMILY  _DOMAINS 347 -0.645783918 -1.748960116 0.014285714 0.509968985 |
| --- | --- | --- |

GO_AGIN

G GO_AGING 315 -0.458692289 -1.242760236 0.014285714 0.509968985

| GO_AMID  E_BINDIN  G |  | GO_AMIDE_BI  NDING 356 -0.450697981 -1.21609988 0.014285714 0.509968985 |
| --- | --- | --- |

| GO_CALC  IUM_ION_  TRANSME  MBRANE_  TRANSPO  RT |  | GO_CALCIUM  _ION_TRANS  MEMBRANE_T  RANSPORT 330 -0.536428235 -1.454516134 0.014285714 0.509968985 |
| --- | --- | --- |

| GO_CELL  ULAR_RES  PONSE_T  O_ABIOTI  C_STIMUL  US |  | GO_CELLULA  R_RESPONSE_  TO_ABIOTIC_  STIMULUS 328 -0.480599168 -1.301858903 0.014285714 0.509968985 |
| --- | --- | --- |

| GO_CYTO  KINE_SEC  RETION |  | GO_CYTOKIN  E_SECRETION 242 -0.561786799 -1.488990579 0.014285714 0.509968985 |
| --- | --- | --- |

| GO_DEFE  NSE_RESP  ONSE_TO  _BACTERI  UM |  | GO_DEFENSE_  RESPONSE_TO  _BACTERIUM 324 -0.572086637 -1.5493222 0.014285714 0.509968985 |
| --- | --- | --- |

| GO_GLUT  AMATERG  IC_SYNAP  SE |  | GO_GLUTAM  ATERGIC_SYN  APSE 356 -0.576065094 -1.554372822 0.014285714 0.509968985 |
| --- | --- | --- |

| GO_HUM  ORAL_IM  MUNE_RE  SPONSE |  | GO_HUMORA  L_IMMUNE_RE  SPONSE 348 -0.634809659 -1.720564555 0.014285714 0.509968985 |
| --- | --- | --- |

| GO_LYMP  HOCYTE_  DIFFEREN  TIATION |  | GO_LYMPHOC  YTE_DIFFEREN  TIATION 353 -0.58845803 -1.584547999 0.014285714 0.509968985 |
| --- | --- | --- |

| GO_LYMP  HOCYTE_  MEDIATE  D_IMMUNI  TY |  | GO_LYMPHOC  YTE_MEDIATE  D_IMMUNITY 347 -0.665964776 -1.80361542 0.014285714 0.509968985 |
| --- | --- | --- |

| GO_MEMB  RANE_RE  GION |  | GO_MEMBRA  NE_REGION 330 -0.502803186 -1.363342379 0.014285714 0.509968985 |
| --- | --- | --- |

| GO_MUSC  LE_CONT  RACTION |  | GO_MUSCLE_  CONTRACTIO  N 357 -0.507251041 -1.367550534 0.014285714 0.509968985 |
| --- | --- | --- |

| GO_NEGA  TIVE_REG  ULATION_  OF_CELL_  MOTILITY |  | GO_NEGATIV  E_REGULATIO  N_OF_CELL_  MOTILITY 319 -0.452576516 -1.224428706 0.014285714 0.509968985 |
| --- | --- | --- |

| GO_NEUR  ON_TO_N  EURON_S  YNAPSE |  | GO_NEURON_  TO_NEURON_  SYNAPSE 363 -0.534929089 -1.444488068 0.014285714 0.509968985 |
| --- | --- | --- |

| GO_PHAG  OCYTOSI  S |  | GO_PHAGOC  YTOSIS 358 -0.664179121 -1.79176123 0.014285714 0.509968985 |
| --- | --- | --- |

| GO_REGU  LATION_  OF_SYNA  PTIC_PLA  STICITY |  | GO_REGULATI  ON_OF_SYNA  PTIC_PLASTI  CITY 187 -0.56488348 -1.451086267 0.014285714 0.509968985 |
| --- | --- | --- |

| GO_REGU  LATION_  OF_T_CEL  L_ACTIV  ATION |  | GO_REGULATI  ON_OF_T_CE  LL_ACTIVATI  ON 317 -0.566635188 -1.534128341 0.014285714 0.509968985 |
| --- | --- | --- |

| GO_RESP  ONSE_TO  _LIGHT_S  TIMULUS |  | GO_RESPONS  E_TO_LIGHT_  STIMULUS 311 -0.563333074 -1.523628033 0.014285714 0.509968985 |
| --- | --- | --- |

| GO_RESP  ONSE_TO  _MOLECU  LE_OF_BA  CTERIAL_  ORIGIN |  | GO_RESPONS  E_TO_MOLEC  ULE_OF_BAC  TERIAL_ORIGI  N 349 -0.500348695 -1.354268227 0.014285714 0.509968985 |
| --- | --- | --- |

| GO_RESP  ONSE_TO  _VIRUS |  | GO_RESPONS  E_TO_VIRUS 327 -0.478492516 -1.294309531 0.014285714 0.509968985 |
| --- | --- | --- |

| GO_T_CE  LL_PROLIF  ERATION |  | GO_T_CELL_P  ROLIFERATIO  N 186 -0.557966715 -1.436112675 0.014285714 0.509968985 |
| --- | --- | --- |

| GO_TRAN  SPORTER_  COMPLEX |  | GO_TRANSPO  RTER_COMPL  EX 330 -0.567041553 -1.53752363 0.014285714 0.509968985 |
| --- | --- | --- |

| GO_ANIO  N_TRANS  MEMBRAN  E_TRANS  PORTER_  ACTIVITY |  | GO_ANION_T  RANSMEMBR  ANE_TRANSP  ORTER_ACTIV  ITY 334 -0.459403539 -1.249048802 0.014492754 0.509968985 |
| --- | --- | --- |

| GO_ERK1  _AND_ER  K2_CASC  ADE |  | GO_ERK1_AN  D_ERK2_CAS  CADE 308 -0.505915911 -1.364102876 0.014492754 0.509968985 |
| --- | --- | --- |
| GO_LYMP  HOCYTE_  COSTIMU  LATION |  | GO_LYMPHOC  YTE_COSTIM  ULATION 57 -0.68596305 -1.569549274 0.014492754 0.509968985 |

| GO_MON  OCYTE_C  HEMOTA  XIS |  | GO_MONOCY  TE_CHEMOTA  XIS 63 -0.712971165 -1.646850394 0.014492754 0.509968985 |
| --- | --- | --- |

| GO_PEPTI  DE_RECEP  TOR_ACT  IVITY |  | GO_PEPTIDE_  RECEPTOR_A  CTIVITY 152 -0.59876515 -1.494865946 0.014492754 0.509968985 |
| --- | --- | --- |

| GO_POSIT  IVE_REGU  LATION_  OF_ERK1_  AND_ERK  2_CASCA  DE |  | GO_POSITIVE  _REGULATION  _OF_ERK1_AN  D_ERK2_CAS  CADE 206 -0.539350624 -1.403762692 0.014492754 0.509968985 |
| --- | --- | --- |

| GO_POSIT  IVE_REGU  LATION_  OF_INTER  FERON_G  AMMA_P  RODUCTI  ON |  | GO_POSITIVE  _REGULATION  _OF_INTERFER  ON_GAMMA_  PRODUCTION 63 -0.69693162 -1.609801589 0.014492754 0.509968985 |
| --- | --- | --- |

| GO_POST  SYNAPTI  C_MEMBR  ANE |  | GO_POSTSYN  APTIC_MEMB  RANE 331 -0.598107016 -1.62443513 0.014492754 0.509968985 |
| --- | --- | --- |

| GO_PROT  EIN_HOM  OOLIGOM  ERIZATIO  N |  | GO_PROTEIN_  HOMOOLIGO  MERIZATION 314 -0.474451668 -1.281071812 0.014492754 0.509968985 |
| --- | --- | --- |

| GO_REGU  LATION_  OF_SYNA  PSE_STRU  CTURE_O  R_ACTIVI  TY |  | GO_REGULATI  ON_OF_SYNA  PSE_STRUCTU  RE_OR_ACTIV  ITY 234 -0.547505819 -1.438662374 0.014492754 0.509968985 |
| --- | --- | --- |

| GO_CATI  ON_CHAN  NEL_ACTI  VITY |  | GO_CATION_  CHANNEL_AC  TIVITY 338 -0.539626261 -1.464030881 0.014705882 0.509968985 |
| --- | --- | --- |

| GO_COMP  LEMENT_  ACTIVATI  ON |  | GO_COMPLE  MENT_ACTIV  ATION 166 -0.694975086 -1.758344646 0.014705882 0.509968985 |
| --- | --- | --- |

| GO_DETE  CTION_OF  _VISIBLE_  LIGHT |  | GO_DETECTIO  N_OF_VISIBLE  _LIGHT 62 -0.700120461 -1.616605403 0.014705882 0.509968985 |
| --- | --- | --- |

| GO_FC_E  PSILON_R  ECEPTOR_  SIGNALIN  G_PATH  WAY |  | GO_FC_EPSIL  ON_RECEPTO  R_SIGNALING  _PATHWAY 168 -0.682255073 -1.723878974 0.014705882 0.509968985 |
| --- | --- | --- |

| GO_IMMU  NOGLOBU  LIN_COM  PLEX_CIR  CULATIN  G |  | GO_IMMUNO  GLOBULIN_CO  MPLEX_CIRCU  LATING 65 -0.812021197 -1.864876687 0.014705882 0.509968985 |
| --- | --- | --- |

| GO_INTRI  NSIC_CO  MPONENT  _OF_SYN  APTIC_M  EMBRANE |  | GO_INTRINSIC  _COMPONENT  _OF_SYNAPTI  C_MEMBRANE 166 -0.688523221 -1.742020891 0.014705882 0.509968985 |
| --- | --- | --- |

| GO_LEUK  OCYTE_C  ELL_CELL  _ADHESIO  N |  | GO_LEUKOCY  TE_CELL_CEL  L_ADHESION 339 -0.545386692 -1.477259208 0.014705882 0.509968985 |
| --- | --- | --- |

| GO_LOCO  MOTORY_  BEHAVIOR |  | GO_LOCOMO  TORY_BEHAVI  OR 197 -0.607039497 -1.56499244 0.014705882 0.509968985 |
| --- | --- | --- |

| GO_REGU  LATION_  OF_CATI  ON_CHAN  NEL_ACTI  VITY |  | GO_REGULATI  ON_OF_CATI  ON_CHANNEL  _ACTIVITY 183 -0.592353035 -1.518956754 0.014705882 0.509968985 |
| --- | --- | --- |

| GO_REGU  LATION_  OF_CATI  ON_TRAN  SMEMBRA  NE_TRAN  SPORT |  | GO_REGULATI  ON_OF_CATI  ON_TRANSME  MBRANE_TRA  NSPORT 342 -0.549946161 -1.487923922 0.014705882 0.509968985 |
| --- | --- | --- |

| GO_REGU  LATION_  OF_DEVEL  OPMENTA  L_GROWT  H |  | GO_REGULATI  ON_OF_DEVE  LOPMENTAL_  GROWTH 342 -0.481561289 -1.302903105 0.014705882 0.509968985 |
| --- | --- | --- |

| GO_RESP  ONSE_TO  _INTERFE  RON_GA  MMA |  | GO_RESPONS  E_TO_INTERF  ERON_GAMM  A 195 -0.581798226 -1.500302469 0.014705882 0.509968985 |
| --- | --- | --- |

| GO_LYMP  HOCYTE_  ACTIVATI  ON_INVO  LVED_IN_I  MMUNE_R  ESPONSE |  | GO_LYMPHOC  YTE_ACTIVA  TION_INVOLV  ED_IN_IMMUN  E_RESPONSE 181 -0.554301926 -1.407892472 0.014925373 0.509968985 |
| --- | --- | --- |

| GO_NATU  RAL_KILL  ER_CELL_  MEDIATE  D_IMMUNI  TY |  | GO_NATURAL  _KILLER_CELL  _MEDIATED_I  MMUNITY 64 -0.700097535 -1.608904638 0.014925373 0.509968985 |
| --- | --- | --- |
| GO_NEUR  ON_PROJ  ECTION_  MEMBRAN  E |  | GO_NEURON_  PROJECTION_  MEMBRANE 59 -0.72379842 -1.665067793 0.014925373 0.509968985 |

| GO_PHOT  OTRANSD  UCTION |  | | GO_PHOTOTR  ANSDUCTION 59 -0.714139799 -1.642848542 0.014925373 0.509968985 | |
| --- | --- | --- | --- | --- |
| GO_POSIT  IVE_REGU  LATION_  OF_ION_T  RANSME  MBRANE_  TRANSPO  RT | |  | | GO_POSITIVE  _REGULATION  _OF_ION_TRA  NSMEMBRANE  _TRANSPORT 155 -0.595373452 -1.485920349 0.014925373 0.509968985 |

| GO_REGU  LATION_  OF_B_CEL  L_ACTIV  ATION |  | GO_REGULATI  ON_OF_B_CEL  L_ACTIVATIO  N 181 -0.707963037 -1.79818215 0.014925373 0.509968985 |
| --- | --- | --- |

| GO_T_CE  LL_RECEP  TOR_SIGN  ALING_PA  THWAY |  | GO_T_CELL_R  ECEPTOR_SIG  NALING_PATH  WAY 199 -0.567027459 -1.456713899 0.014925373 0.509968985 |
| --- | --- | --- |

| GO_VOLT  AGE_GAT  ED_ION_C  HANNEL_  ACTIVITY |  | GO_VOLTAGE  _GATED_ION_  CHANNEL_AC  TIVITY 198 -0.598909685 -1.538021275 0.014925373 0.509968985 |
| --- | --- | --- |

| GO_VOLT  AGE_GAT  ED_POTA  SSIUM_C  HANNEL_  ACTIVITY |  | GO_VOLTAGE  _GATED_POT  ASSIUM_CHA  NNEL_ACTIVI  TY 86 -0.659770431 -1.571610162 0.014925373 0.509968985 |
| --- | --- | --- |

| GO_ANTI  GEN_BIND  ING |  | GO_ANTIGEN  _BINDING 149 -0.768247842 -1.899168357 0.015151515 0.509968985 |
| --- | --- | --- |

| GO_ASSO  CIATIVE_  LEARNING |  | GO_ASSOCIA  TIVE_LEARNIN  G 76 -0.65447416 -1.531812161 0.015151515 0.509968985 |
| --- | --- | --- |

| GO_B_CE  LL_RECEP  TOR_SIGN  ALING_PA  THWAY |  | GO_B_CELL_R  ECEPTOR_SIG  NALING_PATH  WAY 118 -0.755995017 -1.860288351 0.015151515 0.509968985 |
| --- | --- | --- |

| GO_CYTO  SOLIC_C  ALCIUM_I  ON_TRAN  SPORT |  | GO_CYTOSOL  IC_CALCIUM_  ION_TRANSP  ORT 180 -0.568003516 -1.436281713 0.015151515 0.509968985 |
| --- | --- | --- |

| GO_FC_R  ECEPTOR_  MEDIATE  D_STIMUL  ATORY_S  IGNALING  _PATHW  AY |  | GO_FC_RECEP  TOR_MEDIAT  ED_STIMULAT  ORY_SIGNALI  NG_PATHWA  Y 143 -0.722148598 -1.792779499 0.015151515 0.509968985 |
| --- | --- | --- |

| GO_HUM  ORAL_IM  MUNE_RE  SPONSE_  MEDIATE  D_BY_CIR  CULATIN  G_IMMUN  OGLOBULI  N |  | GO_HUMORA  L_IMMUNE_RE  SPONSE_MEDI  ATED_BY_CIR  CULATING_IM  MUNOGLOBUL  IN 143 -0.72211999 -1.792708478 0.015151515 0.509968985 |
| --- | --- | --- |

| GO_IMMU  NOGLOBU  LIN_RECE  PTOR_BIN  DING |  | GO_IMMUNO  GLOBULIN_RE  CEPTOR_BINDI  NG 69 -0.789834748 -1.817241842 0.015151515 0.509968985 |
| --- | --- | --- |

| GO_INTRI  NSIC_CO  MPONENT  _OF_POS  TSYNAPTI  C_DENSIT  Y_MEMBR  ANE |  | GO_INTRINSIC  _COMPONENT  _OF_POSTSY  NAPTIC_DENS  ITY_MEMBRA  NE 54 -0.723493025 -1.621747174 0.015151515 0.509968985 |
| --- | --- | --- |

| GO_INTRI  NSIC_CO  MPONENT  _OF_PRES  YNAPTIC_  MEMBRAN  E |  | GO_INTRINSIC  _COMPONENT  _OF_PRESYN  APTIC_MEMB  RANE 83 -0.666491478 -1.580710644 0.015151515 0.509968985 |
| --- | --- | --- |

| GO_NON_  MOTILE_C  ILIUM |  | GO_NON_MO  TILE_CILIUM 149 -0.59555299 -1.472253265 0.015151515 0.509968985 |
| --- | --- | --- |

| GO_PHOT  ORECEPT  OR_OUTE  R_SEGME  NT  GO_POSIT  IVE_REGU  LATION_  OF_CHOL  ESTEROL_  METABOL  IC_PROCE  SS |  | GO_PHOTORE  CEPTOR_OUT  ER_SEGMENT 87 -0.688754885 -1.640666613 0.015151515 0.509968985  GO_POSITIVE  _REGULATION  _OF_CHOLES  TEROL_META  BOLIC_PROCE  SS 10 -0.882513417 -1.469920214 0.015151515 0.509968985 |
| --- | --- | --- |

| GO_POSIT  IVE_REGU  LATION_  OF_INFLA  MMATOR  Y_RESPO  NSE |  | GO_POSITIVE  _REGULATION  _OF_INFLAM  MATORY_RES  PONSE 143 -0.632812304 -1.570996508 0.015151515 0.509968985 |
| --- | --- | --- |

| GO_POSIT  IVE_REGU  LATION_  OF_LEUK  OCYTE_DI  FFERENTI  ATION  GO_POSIT  IVE_REGU  LATION_  OF_OXID  ATIVE_PH  OSPHORY  LATION |  | GO_POSITIVE  _REGULATION  _OF_LEUKOC  YTE_DIFFEREN  TIATION 147 -0.599632089 -1.478633167 0.015151515 0.509968985  GO_POSITIVE  _REGULATION  _OF_OXIDATI  VE_PHOSPHO  RYLATION 10 -0.908562803 -1.513308244 0.015151515 0.509968985 |
| --- | --- | --- |

| GO_POST  SYNAPTI  C_NEURO  TRANSMI  TTER_REC  EPTOR_A  CTIVITY |  | GO_POSTSYN  APTIC_NEURO  TRANSMITTER  _RECEPTOR_A  CTIVITY 76 -0.633619603 -1.483001582 0.015151515 0.509968985 |
| --- | --- | --- |

| GO_PRES  YNAPTIC_  MEMBRAN  E  GO_REGU  LATION_  OF_ARTE  RY_MORP  HOGENESI  S |  | GO_PRESYNA  PTIC_MEMBR  ANE 157 -0.632983391 -1.576832516 0.015151515 0.509968985  GO_REGULATI  ON_OF_ARTE  RY_MORPHOG  ENESIS 10 -0.931645506 -1.551754948 0.015151515 0.509968985 |
| --- | --- | --- |

| GO_REGU  LATION_  OF_LYMP  HOCYTE_  MEDIATE  D_IMMUNI  TY | |  | | GO_REGULATI  ON_OF_LYMP  HOCYTE_MED  IATED_IMMU  NITY 147 -0.583204789 -1.438125077 0.015151515 0.509968985 |
| --- | --- | --- | --- | --- |
| GO_REGU  LATION_  OF_POST  SYNAPTI  C_MEMBR  ANE_NEU  ROTRANS  MITTER_R  ECEPTOR_  LEVELS |  | | GO_REGULATI  ON_OF_POST  SYNAPTIC_M  EMBRANE_NE  UROTRANSMI  TTER_RECEPT  OR_LEVELS 69 -0.647069322 -1.488768947 0.015151515 0.509968985 | |

| GO_VOLT  AGE_GAT  ED_CATI  ON_CHAN  NEL_ACTI  VITY |  | GO_VOLTAGE  _GATED_CAT  ION_CHANNEL  _ACTIVITY 142 -0.634133692 -1.575277774 0.015151515 0.509968985 |
| --- | --- | --- |

| GO_CARD  IAC_CON  DUCTION |  | GO_CARDIAC  _CONDUCTIO  N 144 -0.578960291 -1.424094891 0.015384615 0.509968985 |
| --- | --- | --- |

| GO_IMMU  NOGLOBU  LIN_COM  PLEX |  | GO_IMMUNO  GLOBULIN_CO  MPLEX 140 -0.796606413 -1.975220938 0.015384615 0.509968985 |
| --- | --- | --- |

| GO_IMMU  NOGLOBU  LIN_PROD  UCTION |  | GO_IMMUNO  GLOBULIN_PR  ODUCTION 202 -0.747152781 -1.919996922 0.015384615 0.509968985 |
| --- | --- | --- |

GO_LEAR

NING GO_LEARNING 145 -0.595432685 -1.462015364 0.015384615 0.509968985

| GO_MULT  ICELLULA  R_ORGAN  ISMAL_SI  GNALING |  | GO_MULTICEL  LULAR_ORGA  NISMAL_SIGN  ALING 202 -0.582073006 -1.495782936 0.015384615 0.509968985 |
| --- | --- | --- |

| GO_NEUR  ON_PROJ  ECTION_T  ERMINUS |  | GO_NEURON_  PROJECTION_  TERMINUS 140 -0.608121603 -1.507864491 0.015384615 0.509968985 |
| --- | --- | --- |

| GO_NEUR  OTRANSM  ITTER_RE  CEPTOR_  ACTIVITY |  | GO_NEUROTR  ANSMITTER_R  ECEPTOR_AC  TIVITY 116 -0.609782147 -1.498574238 0.015384615 0.509968985 |
| --- | --- | --- |

| GO_POSIT  IVE_REGU  LATION_  OF_CYTO  KINE_SEC  RETION |  | GO_POSITIVE  _REGULATION  _OF_CYTOKIN  E_SECRETION 140 -0.600987728 -1.490175731 0.015384615 0.509968985 |
| --- | --- | --- |

| GO_REGU  LATION_  OF_POST  SYNAPTI  C_MEMBR  ANE_POT  ENTIAL |  | GO_REGULATI  ON_OF_POST  SYNAPTIC_M  EMBRANE_PO  TENTIAL 144 -0.640091973 -1.574463261 0.015384615 0.509968985 |
| --- | --- | --- |

| GO_REGU  LATION_  OF_T_CEL  L_DIFFERE  NTIATION |  | | GO_REGULATI  ON_OF_T_CE  LL_DIFFERENT  IATION 139 -0.590908014 -1.465611763 0.015384615 0.509968985 | |
| --- | --- | --- | --- | --- |
| GO_SYNA  PTIC_VESI  CLE_EXO  CYTOSIS | |  | | GO_SYNAPTI  C_VESICLE_E  XOCYTOSIS 116 -0.576753439 -1.417404314 0.015384615 0.509968985 |

| GO_T_CE  LL_ACTIV  ATION_IN  VOLVED_I  N_IMMUN  E_RESPON  SE |  | GO_T_CELL_  ACTIVATION_  INVOLVED_IN  _IMMUNE_RES  PONSE 105 -0.589016183 -1.442415054 0.015384615 0.509968985 |
| --- | --- | --- |

| GO_DETE  CTION_OF  _LIGHT_S  TIMULUS |  | GO_DETECTIO  N_OF_LIGHT_  STIMULUS 74 -0.704855187 -1.636791347 0.015625 0.509968985 |
| --- | --- | --- |

| GO_DIENC  EPHALON  _DEVELOP  MENT |  | GO_DIENCEPH  ALON_DEVEL  OPMENT 74 -0.609657905 -1.415727376 0.015625 0.509968985 |
| --- | --- | --- |

| GO_EXOG  ENOUS_P  ROTEIN_BI  NDING |  | GO_EXOGENO  US_PROTEIN_  BINDING 74 -0.598498261 -1.389812821 0.015625 0.509968985 |
| --- | --- | --- |

| GO_POSIT  IVE_REGU  LATION_  OF_CALCI  UM_ION_  TRANSME  MBRANE_  TRANSPO  RT |  | GO_POSITIVE  _REGULATION  _OF_CALCIU  M_ION_TRAN  SMEMBRANE_  TRANSPORT 71 -0.635617868 -1.465773957 0.015625 0.509968985 |
| --- | --- | --- |

| GO_POSIT  IVE_REGU  LATION_  OF_LYMP  HOCYTE_  DIFFEREN  TIATION |  | | GO_POSITIVE  _REGULATION  _OF_LYMPHO  CYTE_DIFFER  ENTIATION 94 -0.649620803 -1.541060712 0.015625 0.509968985 | |
| --- | --- | --- | --- | --- |
| GO_PRES  YNAPTIC_  ACTIVE_Z  ONE | |  | | GO_PRESYNA  PTIC_ACTIVE  _ZONE 71 -0.610560215 -1.407989467 0.015625 0.509968985 |

| GO_REGU  LATION_  OF_SYNA  PTIC_TRA  NSMISSIO  N_GLUTA  MATERGI  C |  | GO_REGULATI  ON_OF_SYNA  PTIC_TRANS  MISSION_GLU  TAMATERGIC 70 -0.620981054 -1.424467416 0.015625 0.509968985 |
| --- | --- | --- |

| GO_TRAN  SMISSION  _OF_NER  VE_IMPUL  SE |  | GO_TRANSMI  SSION_OF_NE  RVE_IMPULSE 70 -0.624503565 -1.432547697 0.015625 0.509968985 |
| --- | --- | --- |

| GO_CORN  IFICATIO  N |  | GO_CORNIFIC  ATION 113 -0.586356749 -1.423905279 0.015873016 0.509968985 |
| --- | --- | --- |

| GO_CYTO  KINE_MET  ABOLIC_P  ROCESS |  | GO_CYTOKIN  E_METABOLIC  _PROCESS 123 -0.604019464 -1.473146999 0.015873016 0.509968985 |
| --- | --- | --- |

| GO_GABA  _ERGIC_S  YNAPSE |  | GO_GABA_ER  GIC_SYNAPSE 72 -0.659455329 -1.529887221 0.015873016 0.509968985 |
| --- | --- | --- |

| GO_IMMU  NE_RECEP  TOR_ACT  IVITY |  | GO_IMMUNE_  RECEPTOR_A  CTIVITY 126 -0.595293598 -1.45463627 0.015873016 0.509968985 |
| --- | --- | --- |

| GO_INTRI  NSIC_CO  MPONENT  _OF_POS  TSYNAPTI  C_MEMBR  ANE |  | GO_INTRINSIC  _COMPONENT  _OF_POSTSY  NAPTIC_MEM  BRANE 122 -0.695300343 -1.694320978 0.015873016 0.509968985 |
| --- | --- | --- |

| GO_INTRI  NSIC_CO  MPONENT  _OF_POS  TSYNAPTI  C_SPECIA  LIZATION  _MEMBRA  NE |  | GO_INTRINSIC  _COMPONENT  _OF_POSTSY  NAPTIC_SPEC  IALIZATION_  MEMBRANE 77 -0.692838589 -1.605717684 0.015873016 0.509968985 |
| --- | --- | --- |

| GO_LEUK  OCYTE_M  EDIATED_  CYTOTOX  ICITY | | |  | GO_LEUKOCY  TE_MEDIATED  _CYTOTOXICI  TY 106 -0.60548234 -1.467822408 0.015873016 0.509968985 | |
| --- | --- | --- | --- | --- | --- |
| GO_LOCO  MOTOR_R  HYTHM |  | GO_LOCOMO  TOR_RHYTHM 15 -0.882181281 -1.544278387 0.015873016 0.509968985 | | |  |

| GO_LYMP  HOCYTE_  APOPTOTI  C_PROCE  SS | |  | | GO_LYMPHOC  YTE_APOPTO  TIC_PROCESS 72 -0.611240141 -1.418031577 0.015873016 0.509968985 |
| --- | --- | --- | --- | --- |
| GO_LYMP  HOCYTE_  MIGRATIO  N |  | | GO_LYMPHOC  YTE_MIGRATI  ON 112 -0.621714522 -1.509053097 0.015873016 0.509968985 | |

| GO_MEMB  RANE_INV  AGINATIO  N |  | GO_MEMBRA  NE_INVAGINA  TION 130 -0.712757546 -1.754047098 0.015873016 0.509968985 |
| --- | --- | --- |

| GO_PHAG  OCYTOSI  S_RECOG  NITION |  | GO_PHAGOC  YTOSIS_RECO  GNITION 77 -0.777910083 -1.802878763 0.015873016 0.509968985 |
| --- | --- | --- |

| GO_PHOS  PHOLIPAS  E_C_ACTI  VATING_  G_PROTEI  N_COUPL  ED_RECEP  TOR_SIGN  ALING_PA  THWAY |  | GO_PHOSPHO  LIPASE_C_AC  TIVATING_G_  PROTEIN_COU  PLED_RECEPT  OR_SIGNALIN  G_PATHWAY 108 -0.603847964 -1.462300067 0.015873016 0.509968985 |
| --- | --- | --- |

| GO_POSIT  IVE_REGU  LATION_  OF_ADAP  TIVE_IMM  UNE_RESP  ONSE |  | GO_POSITIVE  _REGULATION  _OF_ADAPTI  VE_IMMUNE_  RESPONSE 104 -0.6100326 -1.488104856 0.015873016 0.509968985 |
| --- | --- | --- |

| GO_POSIT  IVE_REGU  LATION_  OF_B_CEL  L_ACTIV  ATION |  | GO_POSITIVE  _REGULATION  _OF_B_CELL_  ACTIVATION 137 -0.72936643 -1.807682185 0.015873016 0.509968985 |
| --- | --- | --- |

| GO_POSIT  IVE_REGU  LATION_  OF_CATI  ON_CHAN  NEL_ACTI  VITY |  | GO_POSITIVE  _REGULATION  _OF_CATION  _CHANNEL_A  CTIVITY 72 -0.634632631 -1.472300412 0.015873016 0.509968985 |
| --- | --- | --- |

| GO_POSIT  IVE_REGU  LATION_  OF_LEUK  OCYTE_M  EDIATED_  IMMUNIT  Y |  | GO_POSITIVE  _REGULATION  _OF_LEUKOC  YTE_MEDIATE  D_IMMUNITY 131 -0.632226158 -1.554748458 0.015873016 0.509968985 |
| --- | --- | --- |

| GO_POSIT  IVE_REGU  LATION_  OF_TRAN  SPORTER_  ACTIVITY  GO_POSIT  IVE_REGU  LATION_  OF_TRIGL  YCERIDE_  LIPASE_A  CTIVITY |  | GO_POSITIVE  _REGULATION  _OF_TRANSP  ORTER_ACTIV  ITY 113 -0.59082413 -1.43475384 0.015873016 0.509968985  GO_POSITIVE  _REGULATION  _OF_TRIGLYC  ERIDE_LIPASE  _ACTIVITY 12 -0.855222111 -1.507377662 0.015873016 0.509968985 |
| --- | --- | --- |

| GO_POST  SYNAPTI  C_DENSIT  Y_MEMBR  ANE |  | GO_POSTSYN  APTIC_DENSI  TY_MEMBRAN  E 82 -0.676184809 -1.585440825 0.015873016 0.509968985 |
| --- | --- | --- |

| GO_POST  SYNAPTI  C_SPECIA  LIZATION  _MEMBRA  NE |  | GO_POSTSYN  APTIC_SPECI  ALIZATION_M  EMBRANE 108 -0.663751742 -1.607365219 0.015873016 0.509968985 |
| --- | --- | --- |

| GO_REGU  LATION_  OF_NERV  OUS_SYS  TEM_PRO  CESS |  | GO_REGULATI  ON_OF_NERV  OUS_SYSTEM  _PROCESS 137 -0.605155759 -1.499834979 0.015873016 0.509968985 |
| --- | --- | --- |

| GO_REGU  LATION_  OF_NEUR  OTRANSM  ITTER_RE  CEPTOR_  ACTIVITY |  | GO_REGULATI  ON_OF_NEUR  OTRANSMITT  ER_RECEPTOR  _ACTIVITY 72 -0.629862201 -1.461233369 0.015873016 0.509968985 |
| --- | --- | --- |

| GO_REGU  LATION_  OF_STRIA  TED_MUS  CLE_CELL  _DIFFERE  NTIATION |  | GO_REGULATI  ON_OF_STRIA  TED_MUSCLE  _CELL_DIFFER  ENTIATION 112 -0.612600582 -1.486931338 0.015873016 0.509968985 |
| --- | --- | --- |

| GO_9PLU  S0_NON_  MOTILE_C  ILIUM |  | GO_9PLUS0_N  ON_MOTILE_  CILIUM 114 -0.607672228 -1.47885783 0.016129032 0.509968985 |
| --- | --- | --- |

| GO_CHEM  ICAL_SYN  APTIC_TR  ANSMISSI  ON_POST  SYNAPTI  C |  | GO_CHEMICA  L_SYNAPTIC_  TRANSMISSIO  N_POSTSYNA  PTIC 109 -0.594442763 -1.435254782 0.016129032 0.509968985 |
| --- | --- | --- |

| GO_CLAT  HRIN_CO  ATED_VE  SICLE_ME  MBRANE |  | GO_CLATHRI  N_COATED_V  ESICLE_MEMB  RANE 111 -0.588956393 -1.426125198 0.016129032 0.509968985 |
| --- | --- | --- |

| GO_DETE  CTION_OF  _ABIOTIC  _STIMULU  S |  | GO_DETECTIO  N_OF_ABIOTI  C_STIMULUS 138 -0.620691916 -1.531915904 0.016129032 0.509968985 |
| --- | --- | --- |

| GO_LEUK  OCYTE_A  POPTOTIC  _PROCES  S |  | GO_LEUKOCY  TE_APOPTOTI  C_PROCESS 107 -0.620924948 -1.502174199 0.016129032 0.509968985 |
| --- | --- | --- |

| GO_NEUR  OMUSCUL  AR_PROC  ESS |  | GO_NEUROMU  SCULAR_PRO  CESS 107 -0.673802136 -1.630097466 0.016129032 0.509968985 |
| --- | --- | --- |

| GO_NEUR  OPEPTIDE  _SIGNALI  NG_PATH  WAY |  | GO_NEUROPE  PTIDE_SIGNA  LING_PATHW  AY 103 -0.660440645 -1.604991957 0.016129032 0.509968985 |
| --- | --- | --- |

| GO_PERIK  ARYON |  | GO_PERIKARY  ON 138 -0.628071044 -1.550128165 0.016129032 0.509968985 |
| --- | --- | --- |

| GO_POSIT  IVE_REGU  LATION_  OF_LYMP  HOCYTE_  MEDIATE  D_IMMUNI  TY |  | GO_POSITIVE  _REGULATION  _OF_LYMPHO  CYTE_MEDIA  TED_IMMUNIT  Y 103 -0.672225921 -1.633632341 0.016129032 0.509968985 |
| --- | --- | --- |

| GO_REGU  LATION_  OF_ALPH  A_BETA_  T_CELL_A  CTIVATIO  N |  | GO_REGULATI  ON_OF_ALPH  A_BETA_T_C  ELL_ACTIVAT  ION 91 -0.635882776 -1.498171212 0.016129032 0.509968985 |
| --- | --- | --- |

| GO_SMO  OTH_MUS  CLE_CON  TRACTIO  N |  | GO_SMOOTH  _MUSCLE_CO  NTRACTION 109 -0.632661641 -1.527532508 0.016129032 0.509968985 |
| --- | --- | --- |

| GO_SYNA  PTIC_TRA  NSMISSIO  N_GLUTA  MATERGI  C |  | GO_SYNAPTI  C_TRANSMIS  SION_GLUTA  MATERGIC 103 -0.6486062 -1.576232085 0.016129032 0.509968985 |
| --- | --- | --- |

| GO_T_CE  LL_RECEP  TOR_COM  PLEX |  | GO_T_CELL_R  ECEPTOR_CO  MPLEX 125 -0.861465571 -2.092518884 0.016129032 0.509968985 |
| --- | --- | --- |

| GO_ALPH  A_BETA_  T_CELL_D  IFFERENTI  ATION |  | GO_ALPHA_B  ETA_T_CELL_  DIFFERENTIAT  ION 100 -0.634753936 -1.525983076 0.016393443 0.509968985 |
| --- | --- | --- |

| GO_CD4_  POSITIVE  _ALPHA_  BETA_T_  CELL_DIF  FERENTIA  TION |  | GO_CD4_POSI  TIVE_ALPHA_  BETA_T_CELL  _DIFFERENTIA  TION 73 -0.63928666 -1.47268094 0.016393443 0.509968985 |
| --- | --- | --- |

| GO_GLUT  AMATE_R  ECEPTOR_  SIGNALIN  G_PATH  WAY |  | GO_GLUTAM  ATE_RECEPTO  R_SIGNALING  _PATHWAY 100 -0.638140837 -1.53412537 0.016393443 0.509968985 |
| --- | --- | --- |

| GO_REGU  LATION_  OF_HUMO  RAL_IMM  UNE_RESP  ONSE |  | GO_REGULATI  ON_OF_HUMO  RAL_IMMUNE  _RESPONSE 133 -0.646746273 -1.586249712 0.016393443 0.509968985 |
| --- | --- | --- |

| GO_REGU  LATION_  OF_LEUK  OCYTE_M  EDIATED_  CYTOTOX  ICITY |  | GO_REGULATI  ON_OF_LEUK  OCYTE_MEDI  ATED_CYTOT  OXICITY 73 -0.627979788 -1.446634073 0.016393443 0.509968985 |
| --- | --- | --- |

| GO_INTER  FERON_G  AMMA_P  RODUCTI  ON |  | GO_INTERFER  ON_GAMMA_  PRODUCTION 110 -0.617001393 -1.483025146 0.016666667 0.511555556 |
| --- | --- | --- |

| GO_MYO  TUBE_DIF  FERENTIA  TION  GO_REGU  LATION_  OF_CELL_  GROWTH  _INVOLVE  D_IN_CAR  DIAC_MU  SCLE_CEL  L_DEVELO  PMENT | |  | GO_MYOTUBE  _DIFFERENTIA  TION 110 -0.603683604 -1.451014497 0.016666667 0.511555556  GO_REGULATI  ON_OF_CELL_  GROWTH_INV  OLVED_IN_CA  RDIAC_MUSC  LE_CELL_DEV  ELOPMENT 22 -0.77903021 -1.4395958 0.016666667 0.511555556 | |
| --- | --- | --- | --- | --- |
| GO_REGU  LATION_  OF_CARB  OHYDRAT  E_BIOSYN  THETIC_P  ROCESS |  | GO_REGULATI  ON_OF_CARB  OHYDRATE_BI  OSYNTHETIC_  PROCESS 97 0.505052814 1.324767235 0.025 0.56797434 | |  |

| GO_TRNA  _THREON  YLCARBA  MOYLADE  NOSINE_  METABOL  IC_PROCE  SS |  | GO_TRNA_TH  REONYLCARB  AMOYLADEN  OSINE_META  BOLIC_PROCE  SS 17 0.895802097 1.597816214 0.025 0.56797434 | | |  |
| --- | --- | --- | --- | --- | --- |
| GO_CYTO  SOLIC_RI  BOSOME  GO_MON  OSACCH  ARIDE_BI  OSYNTHE  TIC_PROC  ESS  GO_REGU  LATION_  OF_PROG  RAMMED_  NECROTIC  _CELL_DE  ATH  GO_CHAP  ERONIN_C  ONTAININ  G_T_COM  PLEX | | |  | GO_CYTOSOL  IC_RIBOSOME 104 0.534653785 1.422960857 0.025641026 0.56797434  GO_MONOSA  CCHARIDE_BI  OSYNTHETIC_  PROCESS 99 0.555608873 1.467242146 0.025641026 0.56797434  GO_REGULATI  ON_OF_PROG  RAMMED_NEC  ROTIC_CELL_  DEATH 24 0.766818596 1.513930318 0.025641026 0.56797434  GO_CHAPERO  NIN_CONTAIN  ING_T_COMP  LEX 11 0.846548868 1.385576261 0.026315789 0.56797434 | |

| GO_ENDO  SOME_ME  MBRANE |  | | GO_ENDOSO  ME_MEMBRA  NE 477 -0.435551795 -1.199324692 0.026315789 0.56797434 | |
| --- | --- | --- | --- | --- |
| GO_FATT  Y_ACID_  HOMEOST  ASIS  GO_POSIT  IVE_REGU  LATION_  OF_GLUC  ONEOGEN  ESIS | |  | | GO_FATTY_A  CID_HOMEOS  TASIS 16 0.799742957 1.427126667 0.026315789 0.56797434  GO_POSITIVE  _REGULATION  _OF_GLUCON  EOGENESIS 16 0.889967225 1.588130221 0.026315789 0.56797434 |

| GO_POSIT  IVE_REGU  LATION_  OF_GRO  WTH |  | GO_POSITIVE  _REGULATION  _OF_GROWTH 272 -0.485563093 -1.311380225 0.026315789 0.56797434 |
| --- | --- | --- |
| GO_POSIT  IVE_REGU  LATION_  OF_NECR  OTIC_CEL  L_DEATH  GO_REGU  LATION_  OF_BILE_  ACID_BIO  SYNTHETI  C_PROCE  SS |  | GO_POSITIVE  _REGULATION  _OF_NECROTI  C_CELL_DEAT  H 11 0.898792716 1.471085603 0.026315789 0.56797434  GO_REGULATI  ON_OF_BILE_  ACID_BIOSYN  THETIC_PROC  ESS 11 0.829168145 1.357128621 0.026315789 0.56797434 |

| GO_CARB  OHYDRAT  E_BINDIN  G |  | | GO_CARBOHY  DRATE_BINDI  NG 270 -0.487351387 -1.309262835 0.027027027 0.56797434 | |
| --- | --- | --- | --- | --- |
| GO_POSIT  IVE_REGU  LATION_  OF_GLUC  OSE_MET  ABOLIC_P  ROCESS  GO_SNRN  A_METAB  OLIC_PRO  CESS | |  | | GO_POSITIVE  _REGULATION  _OF_GLUCOS  E_METABOLIC  _PROCESS 41 0.698362622 1.555530319 0.027027027 0.56797434  GO_SNRNA_M  ETABOLIC_PR  OCESS 45 0.822784017 1.821099333 0.027027027 0.56797434 |
| GO_SNRN  A_PROCE  SSING |  | | GO_SNRNA_P  ROCESSING 36 0.834155091 1.804014795 0.027027027 0.56797434 | |

| GO_MUSC  LE_TISSU  E_DEVELO  PMENT |  | GO_MUSCLE_  TISSUE_DEVE  LOPMENT 398 -0.441538644 -1.198182398 0.02739726 0.56797434 |
| --- | --- | --- |

| GO_NEGA  TIVE_REG  ULATION_  OF_CELL_  ADHESIO  N |  | GO_NEGATIV  E_REGULATIO  N_OF_CELL_A  DHESION 284 -0.460605739 -1.235144569 0.02739726 0.56797434 |
| --- | --- | --- |

| GO_NEGA  TIVE_REG  ULATION_  OF_RESPO  NSE_TO_E  XTERNAL  _STIMULU  S |  | GO_NEGATIV  E_REGULATIO  N_OF_RESPON  SE_TO_EXTER  NAL_STIMUL  US 395 -0.442994308 -1.200151028 0.02739726 0.56797434 |
| --- | --- | --- |

| GO_REGU  LATION_  OF_CHEM  OTAXIS |  | GO_REGULATI  ON_OF_CHEM  OTAXIS 212 -0.516907023 -1.364211115 0.02739726 0.56797434 |
| --- | --- | --- |

| GO_SENS  ORY_ORG  AN_MORP  HOGENESI  S |  | GO_SENSORY  _ORGAN_MO  RPHOGENESIS 258 -0.500380872 -1.334722311 0.02739726 0.56797434 |
| --- | --- | --- |

| GO_TRAN  SPORT_V  ESICLE |  | GO_TRANSPO  RT_VESICLE 399 -0.450352169 -1.22474709 0.02739726 0.56797434 |
| --- | --- | --- |

| GO_CELL  _FATE_C  OMMITME  NT  GO_NCRN  A_3_END  _PROCES  SING |  | GO_CELL_FA  TE_COMMITM  ENT 266 -0.497586259 -1.329378452 0.027777778 0.56797434  GO_NCRNA_3  _END_PROCES  SING 48 0.802788431 1.784805628 0.027777778 0.56797434 |
| --- | --- | --- |

| GO_NEGA  TIVE_REG  ULATION_  OF_CYTO  KINE_PRO  DUCTION |  | GO_NEGATIV  E_REGULATIO  N_OF_CYTOKI  NE_PRODUCTI  ON 296 -0.453885706 -1.228572548 0.027777778 0.56797434 |
| --- | --- | --- |

| GO_POSIT  IVE_REGU  LATION_  OF_NEUR  ON_DIFFE  RENTIATI  ON |  | GO_POSITIVE  _REGULATION  _OF_NEURON  _DIFFERENTIA  TION 376 -0.438214013 -1.186656768 0.027777778 0.56797434 |
| --- | --- | --- |
| GO_REGU  LATION_  OF_BILE_  ACID_ME  TABOLIC_  PROCESS  GO_REGU  LATION_  OF_GLUC  ONEOGEN  ESIS |  | GO_REGULATI  ON_OF_BILE_  ACID_METAB  OLIC_PROCES  S 13 0.806911982 1.35940092 0.027777778 0.56797434  GO_REGULATI  ON_OF_GLUC  ONEOGENESIS 49 0.710377372 1.587162916 0.027777778 0.56797434 |

| GO_RESP  ONSE_TO  _ALCOHO  L |  | GO_RESPONS  E_TO_ALCOH  OL 240 -0.4950793 -1.308458069 0.027777778 0.56797434 |
| --- | --- | --- |

| GO_SECR  ETORY_G  RANULE_  MEMBRAN  E |  | GO_SECRETO  RY_GRANULE  _MEMBRANE 297 -0.462178036 -1.250216778 0.027777778 0.56797434 |
| --- | --- | --- |

| GO_CELL  _SUBSTR  ATE_ADH  ESION |  | GO_CELL_SUB  STRATE_ADH  ESION 343 -0.447532061 -1.216471177 0.028169014 0.56797434 |
| --- | --- | --- |

| GO_POSIT  IVE_REGU  LATION_  OF_DEVEL  OPMENTA  L_GROWT  H |  | GO_POSITIVE  _REGULATION  _OF_DEVELOP  MENTAL_GRO  WTH 186 -0.530652176 -1.365809635 0.028571429 0.56797434 |
| --- | --- | --- |

| GO_POSIT  IVE_REGU  LATION_  OF_HEMO  POIESIS |  | GO_POSITIVE  _REGULATION  _OF_HEMOPOI  ESIS 187 -0.512355149 -1.316150228 0.028571429 0.56797434 |
| --- | --- | --- |

| GO_REGU  LATION_  OF_CYST  EINE_TYP  E_ENDOPE  PTIDASE_  ACTIVITY |  | GO_REGULATI  ON_OF_CYST  EINE_TYPE_EN  DOPEPTIDASE  _ACTIVITY 235 -0.489274181 -1.289884841 0.028571429 0.56797434 |
| --- | --- | --- |

| GO_REGU  LATION_  OF_MUSC  LE_SYSTE  M_PROCE  SS |  | GO_REGULATI  ON_OF_MUSC  LE_SYSTEM_P  ROCESS 249 -0.499290345 -1.324509534 0.028571429 0.56797434 | | |  |
| --- | --- | --- | --- | --- | --- |
| GO_RNA_  3_END_PR  OCESSING | | |  | GO_RNA_3_E  ND_PROCESSI  NG 150 0.638515421 1.755533251 0.028571429 0.56797434 | |
| GO_STAR  TLE_RESP  ONSE |  | GO_STARTLE  _RESPONSE 27 -0.784876356 -1.579010854 0.028571429 0.56797434 | | |  |

| GO_COAT  ED_VESIC  LE_MEMB  RANE | | |  | GO_COATED_  VESICLE_MEM  BRANE 175 -0.526083761 -1.345564596 0.028985507 0.56797434 | |
| --- | --- | --- | --- | --- | --- |
| GO_IMMU  NOGLOBU  LIN_PROD  UCTION_I  NVOLVED  _IN_IMMU  NOGLOBU  LIN_MEDI  ATED_IM  MUNE_RE  SPONSE |  | GO_IMMUNO  GLOBULIN_PR  ODUCTION_IN  VOLVED_IN_I  MMUNOGLOB  ULIN_MEDIAT  ED_IMMUNE_  RESPONSE 57 -0.666411999 -1.524814592 0.028985507 0.56797434 | | |  |

| GO_INTER  LEUKIN_1  2_PRODU  CTION |  | | GO_INTERLEU  KIN_12_PROD  UCTION 57 -0.673661899 -1.541403061 0.028985507 0.56797434 | |
| --- | --- | --- | --- | --- |
| GO_LEADI  NG_EDGE  _MEMBRA  NE | |  | | GO_LEADING_  EDGE_MEMBR  ANE 172 -0.543172573 -1.389782192 0.028985507 0.56797434 |

| GO_NEUR  ON_SPINE |  | GO_NEURON_  SPINE 170 -0.553619085 -1.411622839 0.028985507 0.56797434 | | |  |
| --- | --- | --- | --- | --- | --- |
| GO_NEUR  OTRANSM  ITTER_BIN  DING | | |  | GO_NEUROTR  ANSMITTER_B  INDING 56 -0.629902105 -1.438046962 0.028985507 0.56797434 | |

| GO_NEUR  OTRANSM  ITTER_RE  CEPTOR_  ACTIVITY  _INVOLVE  D_IN_REG  ULATION_  OF_POST  SYNAPTI  C_MEMBR  ANE_POT  ENTIAL |  | GO_NEUROTR  ANSMITTER_R  ECEPTOR_AC  TIVITY_INVOL  VED_IN_REGU  LATION_OF_P  OSTSYNAPTI  C_MEMBRANE  _POTENTIAL 56 -0.681999087 -1.556982756 0.028985507 0.56797434 |
| --- | --- | --- |
| GO_POSIT  IVE_REGU  LATION_  OF_NATU  RAL_KILL  ER_CELL_  MEDIATE  D_IMMUNI  TY |  | GO_POSITIVE  _REGULATION  _OF_NATURA  L_KILLER_CEL  L_MEDIATED_  IMMUNITY 29 -0.799454963 -1.624713817 0.028985507 0.56797434 |

| GO_REGU  LATION_  OF_GLUT  AMATE_R  ECEPTOR_  SIGNALIN  G_PATH  WAY |  | GO_REGULATI  ON_OF_GLUT  AMATE_RECE  PTOR_SIGNAL  ING_PATHWA  Y 63 -0.658669297 -1.521421685 0.028985507 0.56797434 |
| --- | --- | --- |

| GO_REGU  LATION_  OF_LEUK  OCYTE_M  IGRATION |  | GO_REGULATI  ON_OF_LEUK  OCYTE_MIGR  ATION 194 -0.539378298 -1.390371051 0.028985507 0.56797434 |
| --- | --- | --- |

| GO_REGU  LATION_  OF_LYMP  HOCYTE_  DIFFEREN  TIATION |  | GO_REGULATI  ON_OF_LYMP  HOCYTE_DIFF  ERENTIATION 170 -0.582020651 -1.484041403 0.028985507 0.56797434 |
| --- | --- | --- |

| GO_REGU  LATION_  OF_MUSC  LE_ORGA  N_DEVEL  OPMENT |  | GO_REGULATI  ON_OF_MUSC  LE_ORGAN_D  EVELOPMENT 152 -0.569073907 -1.420739338 0.028985507 0.56797434 |
| --- | --- | --- |

| GO_REGU  LATION_  OF_OSSIF  ICATION |  | GO_REGULATI  ON_OF_OSSIF  ICATION 193 -0.50848318 -1.304817789 0.028985507 0.56797434 |
| --- | --- | --- |

| GO_REGU  LATION_  OF_PHOS  PHATASE  _ACTIVIT  Y |  | GO_REGULATI  ON_OF_PHOS  PHATASE_AC  TIVITY 175 -0.518868794 -1.327110873 0.028985507 0.56797434 |
| --- | --- | --- |

| GO_RESP  ONSE_TO  _AMMONI  UM_ION |  | GO_RESPONS  E_TO_AMMO  NIUM_ION 185 -0.543258993 -1.397273517 0.028985507 0.56797434 |
| --- | --- | --- |

| GO_VASC  ULAR_PR  OCESS_IN  _CIRCULA  TORY_SY  STEM |  | GO_VASCUL  AR_PROCESS  _IN_CIRCULA  TORY_SYSTE  M 170 -0.530542476 -1.352781898 0.028985507 0.56797434 |
| --- | --- | --- |

| GO_CELL  _KILLING |  | GO_CELL_KIL  LING 168 -0.582337035 -1.471412393 0.029411765 0.56797434 |
| --- | --- | --- |
| GO_CHEM  OKINE_BI  NDING |  | GO_CHEMOKI  NE_BINDING 32 -0.747625681 -1.498755923 0.029411765 0.56797434 |

| GO_CLAT  HRIN_CO  ATED_VE  SICLE |  | GO_CLATHRI  N_COATED_V  ESICLE 189 -0.529864841 -1.355516421 0.029411765 0.56797434 |
| --- | --- | --- |
| GO_INTER  LEUKIN_1  _SECRETI  ON |  | GO_INTERLEU  KIN_1_SECRE  TION 62 -0.657025635 -1.517097772 0.029411765 0.56797434 |

| GO_LYMP  HOCYTE_  CHEMOTA  XIS |  | GO_LYMPHOC  YTE_CHEMOT  AXIS 62 -0.611729994 -1.412508374 0.029411765 0.56797434 |
| --- | --- | --- |

| GO_NEUR  OTRANSM  ITTER_SE  CRETION |  | GO_NEUROTR  ANSMITTER_S  ECRETION 168 -0.560774644 -1.416929907 0.029411765 0.56797434 |
| --- | --- | --- |

| GO_POSIT  IVE_REGU  LATION_  OF_CELL_  GROWTH |  | GO_POSITIVE  _REGULATION  _OF_CELL_GR  OWTH 168 -0.533643283 -1.348376102 0.029411765 0.56797434 |
| --- | --- | --- |
| GO_POSIT  IVE_REGU  LATION_  OF_PHAG  OCYTOSI  S |  | GO_POSITIVE  _REGULATION  _OF_PHAGOC  YTOSIS 66 -0.658661439 -1.514746897 0.029411765 0.56797434 |

| GO_POSIT  IVE_REGU  LATION_  OF_STRIA  TED_MUS  CLE_CELL  _DIFFERE  NTIATION | |  | | GO_POSITIVE  _REGULATION  _OF_STRIATE  D_MUSCLE_C  ELL_DIFFEREN  TIATION 66 -0.637918297 -1.467043164 0.029411765 0.56797434 |
| --- | --- | --- | --- | --- |
| GO_REGU  LATION_  OF_ALPH  A_BETA_  T_CELL_D  IFFERENTI  ATION |  | | GO_REGULATI  ON_OF_ALPH  A_BETA_T_C  ELL_DIFFEREN  TIATION 62 -0.676031913 -1.560984008 0.029411765 0.56797434 | |

| GO_REGU  LATION_  OF_ANTI  GEN_RECE  PTOR_ME  DIATED_S  IGNALING  _PATHW  AY |  | GO_REGULATI  ON_OF_ANTI  GEN_RECEPTO  R_MEDIATED_  SIGNALING_P  ATHWAY 62 -0.645383882 -1.490216512 0.029411765 0.56797434 | | |  |
| --- | --- | --- | --- | --- | --- |
| GO_REGU  LATION_  OF_LIPID_  KINASE_A  CTIVITY | | |  | GO_REGULATI  ON_OF_LIPID_  KINASE_ACTI  VITY 66 -0.634971079 -1.460265342 0.029411765 0.56797434 | |

| GO_REGU  LATION_  OF_MUSC  LE_CELL_  DIFFEREN  TIATION  GO_REGU  LATION_  OF_NECR  OTIC_CEL  L_DEATH |  | GO_REGULATI  ON_OF_MUSC  LE_CELL_DIFF  ERENTIATION 171 -0.53636044 -1.368660258 0.029411765 0.56797434  GO_REGULATI  ON_OF_NECR  OTIC_CELL_D  EATH 32 0.726817502 1.481234473 0.029411765 0.56797434 |
| --- | --- | --- |

| GO_REGU  LATION_  OF_SIGN  ALING_RE  CEPTOR_  ACTIVITY |  | GO_REGULATI  ON_OF_SIGN  ALING_RECEP  TOR_ACTIVIT  Y 169 -0.558309265 -1.416009538 0.029411765 0.56797434 |
| --- | --- | --- |

| GO_REGU  LATION_  OF_SMOO  TH_MUSC  LE_CONT  RACTION |  | GO_REGULATI  ON_OF_SMO  OTH_MUSCLE  _CONTRACTI  ON 65 -0.651647449 -1.496564548 0.029411765 0.56797434 |
| --- | --- | --- |
| GO_REGU  LATORY_  T_CELL_D  IFFERENTI  ATION |  | GO_REGULAT  ORY_T_CELL_  DIFFERENTIAT  ION 32 -0.746158236 -1.495814155 0.029411765 0.56797434 |

| GO_SYNA  PSE_ASSE  MBLY |  | GO_SYNAPSE  _ASSEMBLY 178 -0.542410542 -1.377990384 0.029411765 0.56797434 |
| --- | --- | --- |

| GO_DRUG  _TRANSP  ORT |  | GO_DRUG_TR  ANSPORT 200 -0.529610301 -1.361632061 0.029850746 0.56797434 |
| --- | --- | --- |
| GO_LYMP  HOCYTE_  HOMEOST  ASIS |  | GO_LYMPHOC  YTE_HOMEOS  TASIS 61 -0.635999751 -1.463809353 0.029850746 0.56797434 |

| GO_MAIN  _AXON |  | GO_MAIN_AX  ON 67 -0.629922318 -1.449420342 0.029850746 0.56797434 |
| --- | --- | --- |
| GO_MULT  I_ORGANI  SM_BEHA  VIOR |  | GO_MULTI_O  RGANISM_BE  HAVIOR 67 -0.610734416 -1.405269922 0.029850746 0.56797434 |

| GO_POSIT  IVE_REGU  LATION_  OF_CELL_  KILLING |  | GO_POSITIVE  _REGULATION  _OF_CELL_KIL  LING 64 -0.664893038 -1.528000656 0.029850746 0.56797434 |
| --- | --- | --- |
| GO_POSIT  IVE_REGU  LATION_  OF_CYTO  KINE_BIO  SYNTHETI  C_PROCE  SS |  | GO_POSITIVE  _REGULATION  _OF_CYTOKIN  E_BIOSYNTHE  TIC_PROCESS 67 -0.657516777 -1.512913838 0.029850746 0.56797434 |

| GO_POSIT  IVE_REGU  LATION_  OF_INTER  LEUKIN_1  _PRODUC  TION |  | GO_POSITIVE  _REGULATION  _OF_INTERLE  UKIN_1_PROD  UCTION 59 -0.657721333 -1.513060235 0.029850746 0.56797434 |
| --- | --- | --- |
| GO_POSIT  IVE_T_CE  LL_SELEC  TION |  | GO_POSITIVE  _T_CELL_SEL  ECTION 34 -0.761367944 -1.531929515 0.029850746 0.56797434 |

| GO_REGU  LATION_  OF_CALCI  UM_ION_  TRANSME  MBRANE_  TRANSPO  RT | | |  | GO_REGULATI  ON_OF_CALC  IUM_ION_TRA  NSMEMBRANE  _TRANSPORT 154 -0.541893751 -1.350675355 0.029850746 0.56797434 | |
| --- | --- | --- | --- | --- | --- |
| GO_REGU  LATION_  OF_IMMU  NOGLOBU  LIN_PROD  UCTION |  | GO_REGULATI  ON_OF_IMMU  NOGLOBULIN_  PRODUCTION 64 -0.607331092 -1.395716684 0.029850746 0.56797434 | | |  |

| GO_REGU  LATION_  OF_LEUK  OCYTE_M  EDIATED_  IMMUNIT  Y |  | GO_REGULATI  ON_OF_LEUK  OCYTE_MEDI  ATED_IMMUN  ITY 198 -0.552537134 -1.418934923 0.029850746 0.56797434 |
| --- | --- | --- |

| GO_SYNC  YTIUM_F  ORMATIO  N |  | GO_SYNCYTI  UM_FORMATI  ON 59 -0.639695801 -1.471593259 0.029850746 0.56797434 | | |  |
| --- | --- | --- | --- | --- | --- |
| GO_T_CE  LL_DIFFER  ENTIATIO  N_INVOLV  ED_IN_IM  MUNE_RE  SPONSE | | |  | GO_T_CELL_D  IFFERENTIATI  ON_INVOLVE  D_IN_IMMUNE  _RESPONSE 67 -0.649579141 -1.494649728 0.029850746 0.56797434 | |

| GO_ANTI  PORTER_  ACTIVITY |  | GO_ANTIPOR  TER_ACTIVIT  Y 84 -0.589237011 -1.400287287 0.03030303 0.56797434 |
| --- | --- | --- |
| GO_CELL  _ADHESIO  N_MEDIA  TED_BY_I  NTEGRIN |  | GO_CELL_AD  HESION_MEDI  ATED_BY_INT  EGRIN 69 -0.589771986 -1.356939957 0.03030303 0.56797434 |

| GO_CELL  ULAR_DEF  ENSE_RES  PONSE |  | | GO_CELLULA  R_DEFENSE_R  ESPONSE 52 -0.691511709 -1.514055162 0.03030303 0.56797434 | |
| --- | --- | --- | --- | --- |
| GO_FICOL  IN_1_RICH  _GRANUL  E_MEMBR  ANE | |  | | GO_FICOLIN_  1_RICH_GRAN  ULE_MEMBRA  NE 60 -0.613353333 -1.399147356 0.03030303 0.56797434 |

| GO_INTER  LEUKIN_6  _SECRETI  ON |  | GO_INTERLEU  KIN_6_SECRE  TION 52 -0.638034775 -1.396968168 0.03030303 0.56797434 |
| --- | --- | --- |
| GO_MULT  ICELLULA  R_ORGAN  ISMAL_RE  SPONSE_  TO_STRE  SS |  | GO_MULTICEL  LULAR_ORGA  NISMAL_RESP  ONSE_TO_ST  RESS 76 -0.625005849 -1.462840887 0.03030303 0.56797434 |

| GO_NATU  RAL_KILL  ER_CELL_  ACTIVATI  ON |  | GO_NATURAL  _KILLER_CELL  _ACTIVATION 83 -0.603229706 -1.430673382 0.03030303 0.56797434 | | |  |
| --- | --- | --- | --- | --- | --- |
| GO_NEGA  TIVE_REG  ULATION_  OF_LEUK  OCYTE_A  POPTOTIC  _PROCES  S | | |  | GO_NEGATIV  E_REGULATIO  N_OF_LEUKO  CYTE_APOPT  OTIC_PROCES  S 49 -0.625586884 -1.34614565 0.03030303 0.56797434 | |

| GO_NEUR  OMUSCUL  AR_PROC  ESS_CON  TROLLING  _BALANC  E |  | GO_NEUROMU  SCULAR_PRO  CESS_CONTR  OLLING_BALA  NCE 52 -0.683643734 -1.496828342 0.03030303 0.56797434 | | |  |
| --- | --- | --- | --- | --- | --- |
| GO_NEUR  OTRANSM  ITTER_RE  CEPTOR_  TRANSPO  RT | | |  | GO_NEUROTR  ANSMITTER_R  ECEPTOR_TR  ANSPORT 48 -0.63779142 -1.379670105 0.03030303 0.56797434 | |
| GO_PHOT  ORECEPT  OR_INNER  _SEGMEN  T |  | GO_PHOTORE  CEPTOR_INNE  R_SEGMENT 49 -0.648945703 -1.396409449 0.03030303 0.56797434 | | |  |

| GO_POSIT  IVE_REGU  LATION_  OF_ALPH  A_BETA_  T_CELL_A  CTIVATIO  N |  | GO_POSITIVE  _REGULATION  _OF_ALPHA_  BETA_T_CELL  _ACTIVATION 60 -0.65332748 -1.490334147 0.03030303 0.56797434 |
| --- | --- | --- |
| GO_POSIT  IVE_REGU  LATION_  OF_CALCI  UM_ION_  TRANSPO  RT_INTO_  CYTOSOL |  | GO_POSITIVE  _REGULATION  _OF_CALCIU  M_ION_TRAN  SPORT_INTO_  CYTOSOL 54 -0.654105925 -1.466212388 0.03030303 0.56797434 |

| GO_PROT  EIN_LOCA  LIZATION  _TO_SYN  APSE | |  | | GO_PROTEIN_  LOCALIZATIO  N_TO_SYNAP  SE 83 -0.612541435 -1.452757908 0.03030303 0.56797434 |
| --- | --- | --- | --- | --- |
| GO_REGU  LATION_  OF_BEHA  VIOR |  | | GO_REGULATI  ON_OF_BEHA  VIOR 69 -0.590856282 -1.359434693 0.03030303 0.56797434 | |

| GO_REGU  LATION_  OF_CD4_  POSITIVE  _ALPHA_  BETA_T_  CELL_AC  TIVATION  GO_REGU  LATION_  OF_CHOL  ESTEROL_  BIOSYNTH  ETIC_PRO  CESS | | |  | GO_REGULATI  ON_OF_CD4_  POSITIVE_ALP  HA_BETA_T_  CELL_ACTIVA  TION 60 -0.657319375 -1.499440236 0.03030303 0.56797434  GO_REGULATI  ON_OF_CHOL  ESTEROL_BIO  SYNTHETIC_P  ROCESS 50 -0.665860418 -1.443665064 0.03030303 0.56797434 | |
| --- | --- | --- | --- | --- | --- |
| GO_REGU  LATION_  OF_INTER  LEUKIN_1  _SECRETI  ON |  | GO_REGULATI  ON_OF_INTER  LEUKIN_1_SE  CRETION 54 -0.668661516 -1.498839501 0.03030303 0.56797434 | | |  |

| GO_REGU  LATION_  OF_LEUK  OCYTE_A  POPTOTIC  _PROCES  S |  | GO_REGULATI  ON_OF_LEUK  OCYTE_APOP  TOTIC_PROCE  SS 85 -0.625147477 -1.47998284 0.03030303 0.56797434 |
| --- | --- | --- |
| GO_REGU  LATION_  OF_MYOT  UBE_DIFF  ERENTIAT  ION |  | GO_REGULATI  ON_OF_MYOT  UBE_DIFFEREN  TIATION 60 -0.621844442 -1.418516801 0.03030303 0.56797434 |

| GO_REGU  LATION_  OF_REGUL  ATED_SE  CRETORY  _PATHW  AY | | |  | GO_REGULATI  ON_OF_REGU  LATED_SECRE  TORY_PATH  WAY 157 -0.551596762 -1.374089308 0.03030303 0.56797434 | |
| --- | --- | --- | --- | --- | --- |
| GO_RESP  ONSE_TO  _BRONCH  ODILATO  R |  | GO_RESPONS  E_TO_BRONC  HODILATOR 52 -0.656196161 -1.436732266 0.03030303 0.56797434 | | |  |

| GO_RETIN  A_DEVEL  OPMENT_I  N_CAMER  A_TYPE_E  YE | | |  | GO_RETINA_D  EVELOPMENT  _IN_CAMERA  _TYPE_EYE 149 -0.545470153 -1.348444601 0.03030303 0.56797434 | |
| --- | --- | --- | --- | --- | --- |
| GO_SCHA  FFER_COL  LATERAL  _CA1_SY  NAPSE |  | GO_SCHAFFE  R_COLLATER  AL_CA1_SYN  APSE 84 -0.599953333 -1.425754 0.03030303 0.56797434 | | |  |

| GO_TRAN  SMITTER_  GATED_C  HANNEL_  ACTIVITY |  | GO_TRANSMI  TTER_GATED  _CHANNEL_A  CTIVITY 60 -0.625194752 -1.426159341 0.03030303 0.56797434 |
| --- | --- | --- |

| GO_BLOO  D_MICRO  PARTICLE |  | GO_BLOOD_M  ICROPARTICL  E 145 -0.558799005 -1.372065646 0.030769231 0.56797434 |
| --- | --- | --- |

| GO_CALC  IUM_ION_  TRANSME  MBRANE_I  MPORT_IN  TO_CYTO  SOL |  | GO_CALCIUM  _ION_TRANS  MEMBRANE_I  MPORT_INTO  _CYTOSOL 144 -0.572659304 -1.408596067 0.030769231 0.56797434 |
| --- | --- | --- |

| GO_CALC  IUM_ION_  TRANSME  MBRANE_  TRANSPO  RTER_AC  TIVITY |  | GO_CALCIUM  _ION_TRANS  MEMBRANE_T  RANSPORTER  _ACTIVITY 140 -0.540356896 -1.339838895 0.030769231 0.56797434 |
| --- | --- | --- |
| GO_CORE  CEPTOR_  ACTIVITY |  | GO_CORECEP  TOR_ACTIVIT  Y 45 -0.665033802 -1.412456416 0.030769231 0.56797434 |

| GO_EXCI  TATORY_  SYNAPSE |  | GO_EXCITAT  ORY_SYNAPS  E 51 -0.679805486 -1.475552139 0.030769231 0.56797434 |
| --- | --- | --- |
| GO_MHC_  PROTEIN_  BINDING |  | GO_MHC_PRO  TEIN_BINDING 41 -0.757583091 -1.595338025 0.030769231 0.56797434 |

| GO_NEUR  OTRANSM  ITTER_RE  CEPTOR_  COMPLEX |  | GO_NEUROTR  ANSMITTER_R  ECEPTOR_CO  MPLEX 53 -0.630457708 -1.394394219 0.030769231 0.56797434 |
| --- | --- | --- |
| GO_POSIT  IVE_REGU  LATION_  OF_LEUK  OCYTE_M  EDIATED_  CYTOTOX  ICITY |  | GO_POSITIVE  _REGULATION  _OF_LEUKOC  YTE_MEDIATE  D_CYTOTOXI  CITY 51 -0.683528893 -1.483633982 0.030769231 0.56797434 |

| GO_POSIT  IVE_REGU  LATION_  OF_LEUK  OCYTE_P  ROLIFERA  TION |  | GO_POSITIVE  _REGULATION  _OF_LEUKOC  YTE_PROLIFER  ATION 139 -0.57201188 -1.418744238 0.030769231 0.56797434 |
| --- | --- | --- |

| GO_POSIT  IVE_REGU  LATION_  OF_SYNA  PTIC_TRA  NSMISSIO  N |  | GO_POSITIVE  _REGULATION  _OF_SYNAPTI  C_TRANSMIS  SION 164 -0.546221751 -1.366423241 0.030769231 0.56797434 |
| --- | --- | --- |

| GO_POSIT  IVE_REGU  LATION_  OF_TRAN  SMEMBRA  NE_TRAN  SPORT |  | GO_POSITIVE  _REGULATION  _OF_TRANSM  EMBRANE_TR  ANSPORT 201 -0.528648386 -1.356700787 0.030769231 0.56797434 |
| --- | --- | --- |

| GO_POTA  SSIUM_IO  N_TRANS  MEMBRAN  E_TRANS  PORTER_  ACTIVITY | |  | | GO_POTASSI  UM_ION_TRA  NSMEMBRANE  _TRANSPORT  ER_ACTIVITY 159 -0.557008298 -1.383693815 0.030769231 0.56797434 |
| --- | --- | --- | --- | --- |
| GO_PROT  EIN_LOCA  LIZATION  _TO_POS  TSYNAPTI  C_MEMBR  ANE |  | | GO_PROTEIN_  LOCALIZATIO  N_TO_POSTS  YNAPTIC_ME  MBRANE 44 -0.648135486 -1.384716039 0.030769231 0.56797434 | |

| GO_REGU  LATION_  OF_ADAP  TIVE_IMM  UNE_RESP  ONSE |  | GO_REGULATI  ON_OF_ADAP  TIVE_IMMUNE  _RESPONSE 159 -0.537237741 -1.334580726 0.030769231 0.56797434 |
| --- | --- | --- |

| GO_REGU  LATION_  OF_NEUR  OTRANSM  ITTER_TR  ANSPORT |  | GO_REGULATI  ON_OF_NEUR  OTRANSMITT  ER_TRANSPO  RT 140 -0.572380086 -1.419241815 0.030769231 0.56797434 |
| --- | --- | --- |

| GO_REGU  LATION_  OF_TUBE_  SIZE |  | | GO_REGULATI  ON_OF_TUBE  _SIZE 140 -0.554904769 -1.37591099 0.030769231 0.56797434 | |
| --- | --- | --- | --- | --- |
| GO_SOM  ATIC_REC  OMBINATI  ON_OF_I  MMUNOG  LOBULIN_  GENE_SEG  MENTS | |  | | GO_SOMATIC  _RECOMBINA  TION_OF_IMM  UNOGLOBULIN  _GENE_SEGM  ENTS 53 -0.63270092 -1.399355569 0.030769231 0.56797434 |

| GO_SYNA  PTIC_TRA  NSMISSIO  N_GABAE  RGIC |  | GO_SYNAPTI  C_TRANSMIS  SION_GABAE  RGIC 45 -0.687010976 -1.459133444 0.030769231 0.56797434 | | |  |
| --- | --- | --- | --- | --- | --- |
| GO_TUM  OR_NECR  OSIS_FAC  TOR_RECE  PTOR_SUP  ERFAMILY  _BINDING | | |  | GO_TUMOR_N  ECROSIS_FAC  TOR_RECEPTO  R_SUPERFAMI  LY_BINDING 45 -0.673232426 -1.429869365 0.030769231 0.56797434 | |

| GO_B_CE  LL_ACTIV  ATION_IN  VOLVED_I  N_IMMUN  E_RESPON  SE |  | GO_B_CELL_  ACTIVATION_  INVOLVED_IN  _IMMUNE_RES  PONSE 74 -0.584369909 -1.35700443 0.03125 0.56797434 |
| --- | --- | --- |
| GO_CALC  IUM_CHA  NNEL_REG  ULATOR_  ACTIVITY |  | GO_CALCIUM  _CHANNEL_RE  GULATOR_AC  TIVITY 47 -0.664680517 -1.425753477 0.03125 0.56797434 |

| GO_CELL  ULAR_RES  PONSE_T  O_AMMO  NIUM_ION |  | GO_CELLULA  R_RESPONSE_  TO_AMMONI  UM_ION 135 -0.543197614 -1.347552361 0.03125 0.56797434 |
| --- | --- | --- |

| GO_EXTR  ACELLUL  AR_LIGA  ND_GATE  D_ION_CH  ANNEL_A  CTIVITY |  | | GO_EXTRACE  LLULAR_LIGA  ND_GATED_I  ON_CHANNEL  _ACTIVITY 74 -0.597385987 -1.387229934 0.03125 0.56797434 | |
| --- | --- | --- | --- | --- |
| GO_INTEG  RATOR_C  OMPLEX  GO_MEMB  RANE_DIS  RUPTION_  IN_OTHER  _ORGANI  SM | |  | | GO_INTEGRA  TOR_COMPLE  X 28 0.809797455 1.689291279 0.03125 0.56797434  GO_MEMBRA  NE_DISRUPTIO  N_IN_OTHER_  ORGANISM 11 -0.853850593 -1.458028905 0.03125 0.56797434 |

| GO_ORGA  NIC_HYDR  OXY_CO  MPOUND_  CATABOL  IC_PROCE  SS |  | GO_ORGANIC  _HYDROXY_C  OMPOUND_C  ATABOLIC_PR  OCESS 74 -0.576746092 -1.339300654 0.03125 0.56797434 |
| --- | --- | --- |
| GO_POSIT  IVE_REGU  LATION_  OF_CHEM  OTAXIS |  | GO_POSITIVE  _REGULATION  _OF_CHEMOT  AXIS 135 -0.555523146 -1.378129262 0.03125 0.56797434 |

| GO_RETIN  A_HOMEO  STASIS | | |  | GO_RETINA_H  OMEOSTASIS 74 -0.586224709 -1.361311584 0.03125 0.56797434 | |
| --- | --- | --- | --- | --- | --- |
| GO_RHYT  HMIC_BEH  AVIOR  GO_STRU  CTURAL_  CONSTIT  UENT_OF  _NUCLEA  R_PORE |  | GO_RHYTHMI  C_BEHAVIOR 47 -0.702890618 -1.507714933 0.03125 0.56797434  GO_STRUCTU  RAL_CONSTIT  UENT_OF_NU  CLEAR_PORE 28 0.711155117 1.48351681 0.03125 0.56797434 | | |  |

| GO_T_CE  LL_SELEC  TION |  | GO_T_CELL_S  ELECTION 47 -0.726376616 -1.558092887 0.03125 0.56797434 |
| --- | --- | --- |
| GO_ACTI  ON_POTE  NTIAL |  | GO_ACTION_  POTENTIAL 130 -0.547426282 -1.347178274 0.031746032 0.56797434 |

| GO_ALPH  A_BETA_  T_CELL_A  CTIVATIO  N |  | GO_ALPHA_B  ETA_T_CELL_  ACTIVATION 136 -0.589640605 -1.457220262 0.031746032 0.56797434 |
| --- | --- | --- |
| GO_ANTI  BACTERIA  L_HUMOR  AL_RESP  ONSE |  | GO_ANTIBAC  TERIAL_HUM  ORAL_RESPO  NSE 46 -0.658467781 -1.385799953 0.031746032 0.56797434 |

| GO_ANTI  MICROBIA  L_HUMOR  AL_RESP  ONSE |  | | GO_ANTIMIC  ROBIAL_HUM  ORAL_RESPO  NSE 122 -0.574769382 -1.400608863 0.031746032 0.56797434 | |
| --- | --- | --- | --- | --- |
| GO_DEND  RITE_MEM  BRANE | |  | | GO_DENDRITE  _MEMBRANE 42 -0.747392546 -1.550033028 0.031746032 0.56797434 |

| GO_EXTR  ACELLUL  AR_MATR  IX_DISAS  SEMBLY |  | GO_EXTRACE  LLULAR_MAT  RIX_DISASSE  MBLY 81 -0.61750509 -1.443016435 0.031746032 0.56797434 |
| --- | --- | --- |
| GO_INHIBI  TORY_EX  TRACELL  ULAR_LIG  AND_GAT  ED_ION_C  HANNEL_  ACTIVITY |  | GO_INHIBITOR  Y_EXTRACEL  LULAR_LIGAN  D_GATED_IO  N_CHANNEL_  ACTIVITY 15 -0.806267922 -1.411390327 0.031746032 0.56797434 |

| GO_INTRI  NSIC_CO  MPONENT  _OF_SYN  APTIC_VE  SICLE_ME  MBRANE |  | GO_INTRINSIC  _COMPONENT  _OF_SYNAPTI  C_VESICLE_M  EMBRANE 46 -0.6923039 -1.457010866 0.031746032 0.56797434 | | |  |
| --- | --- | --- | --- | --- | --- |
| GO_LIGA  ND_GATE  D_ION_CH  ANNEL_A  CTIVITY | | |  | GO_LIGAND_  GATED_ION_  CHANNEL_AC  TIVITY 137 -0.58537107 -1.450800052 0.031746032 0.56797434 | |

| GO_MYO  BLAST_F  USION |  | GO_MYOBLA  ST_FUSION 42 -0.701714187 -1.455299725 0.031746032 0.56797434 | | |  |
| --- | --- | --- | --- | --- | --- |
| GO_NEGA  TIVE_REG  ULATION_  OF_BLOO  D_PRESSU  RE | | |  | GO_NEGATIV  E_REGULATIO  N_OF_BLOOD  _PRESSURE 46 -0.65193725 -1.37205591 0.031746032 0.56797434 | |
| GO_NEUR  OPEPTIDE  _BINDING |  | GO_NEUROPE  PTIDE_BINDIN  G 24 -0.774737885 -1.462637859 0.031746032 0.56797434 | | |  |

| GO_POSIT  IVE_REGU  LATION_  OF_ALPH  A_BETA_  T_CELL_D  IFFERENTI  ATION |  | GO_POSITIVE  _REGULATION  _OF_ALPHA_  BETA_T_CELL  _DIFFERENTIA  TION 46 -0.637109376 -1.340849422 0.031746032 0.56797434 |
| --- | --- | --- |
| GO_POSIT  IVE_REGU  LATION_  OF_LEUK  OCYTE_M  IGRATION |  | GO_POSITIVE  _REGULATION  _OF_LEUKOC  YTE_MIGRATI  ON 130 -0.543141443 -1.336633582 0.031746032 0.56797434 |

| GO_POSIT  IVE_REGU  LATION_  OF_NATU  RAL_KILL  ER_CELL_  MEDIATE  D_CYTOT  OXICITY | |  | | GO_POSITIVE  _REGULATION  _OF_NATURA  L_KILLER_CEL  L_MEDIATED_  CYTOTOXICIT  Y 24 -0.804451271 -1.518734153 0.031746032 0.56797434 |
| --- | --- | --- | --- | --- |
| GO_POTA  SSIUM_C  HANNEL_  ACTIVITY |  | | GO_POTASSI  UM_CHANNEL  _ACTIVITY 123 -0.586749991 -1.43102837 0.031746032 0.56797434 | |

| GO_REGU  LATION_  OF_CARDI  AC_MUS  CLE_CELL  _DIFFERE  NTIATION | | |  | GO_REGULATI  ON_OF_CARD  IAC_MUSCLE  _CELL_DIFFER  ENTIATION 46 -0.695342839 -1.463406565 0.031746032 0.56797434 | |
| --- | --- | --- | --- | --- | --- |
| GO_REGU  LATION_  OF_CD4_  POSITIVE  _ALPHA_  BETA_T_  CELL_DIF  FERENTIA  TION |  | GO_REGULATI  ON_OF_CD4_  POSITIVE_ALP  HA_BETA_T_  CELL_DIFFERE  NTIATION 46 -0.702869847 -1.479247775 0.031746032 0.56797434 | | |  |
| GO_REGU  LATION_  OF_NATU  RAL_KILL  ER_CELL_  MEDIATE  D_IMMUNI  TY | | |  | GO_REGULATI  ON_OF_NATU  RAL_KILLER_  CELL_MEDIAT  ED_IMMUNITY 43 -0.718016816 -1.503087553 0.031746032 0.56797434 | |

| GO_REGU  LATION_  OF_SYNA  PTIC_VESI  CLE_CYC  LE | | |  | GO_REGULATI  ON_OF_SYNA  PTIC_VESICLE  _CYCLE 112 -0.573055053 -1.390944675 0.031746032 0.56797434 | | |
| --- | --- | --- | --- | --- | --- | --- |
| GO_T_HE  LPER_1_T  YPE_IMM  UNE_RESP  ONSE |  | GO_T_HELPER  _1_TYPE_IMM  UNE_RESPONS  E 42 -0.716494813 -1.485953573 0.031746032 0.56797434 | | |  | |
| GO_NCRN  A_PROCE  SSING |  | GO_NCRNA_P  ROCESSING 378 0.420314094 1.241920789 0.032258065 0.569754971 | | | |  |

| GO_POSIT  IVE_REGU  LATION_  OF_T_CEL  L_PROLIFE  RATION |  | GO_POSITIVE  _REGULATION  _OF_T_CELL_  PROLIFERATIO  N 95 -0.626568969 -1.477254106 0.032258065 0.569754971 |
| --- | --- | --- |

| GO_REGU  LATION_  OF_RELEA  SE_OF_SE  QUESTERE  D_CALCI  UM_ION_I  NTO_CYT  OSOL | | |  | | GO_REGULATI  ON_OF_RELE  ASE_OF_SEQ  UESTERED_C  ALCIUM_ION_  INTO_CYTOS  OL 78 -0.608976029 -1.410522783 0.032258065 0.569754971 | | |
| --- | --- | --- | --- | --- | --- | --- | --- |
| GO_REGU  LATION_  OF_SYNA  PTIC_VESI  CLE_EXO  CYTOSIS | | | |  | | GO_REGULATI  ON_OF_SYNA  PTIC_VESICLE  _EXOCYTOSI  S 78 -0.617836945 -1.431046618 0.032258065 0.569754971 | |
| GO_SNRN  A_3_END  _PROCES  SING |  | GO_SNRNA_3  _END_PROCES  SING 30 0.842783053 1.696435885 0.032258065 0.569754971 | | | | |  |

| GO_DEFE  NSE_RESP  ONSE_TO  _GRAM_P  OSITIVE_  BACTERIU  M |  | GO_DEFENSE_  RESPONSE_TO  _GRAM_POSI  TIVE_BACTER  IUM 101 -0.575850357 -1.387031423 0.032786885 0.576148167 |
| --- | --- | --- |

| GO_POSIT  IVE_REGU  LATION_  OF_LIPAS  E_ACTIVI  TY |  | GO_POSITIVE  _REGULATION  _OF_LIPASE_  ACTIVITY 73 -0.593866728 -1.368050151 0.032786885 0.576148167 |
| --- | --- | --- |
| GO_POSIT  IVE_REGU  LATION_  OF_T_HEL  PER_1_TY  PE_IMMU  NE_RESPO  NSE |  | GO_POSITIVE  _REGULATION  _OF_T_HELPE  R_1_TYPE_IM  MUNE_RESPO  NSE 19 -0.768261631 -1.3849634 0.033333333 0.584263959 |

| GO_OLFA  CTORY_R  ECEPTOR_  ACTIVITY |  | GO_OLFACTO  RY_RECEPTOR  _ACTIVITY 419 0.452640345 1.353805355 0.034482759 0.602880838 |
| --- | --- | --- |

| GO_SENS  ORY_PER  CEPTION_  OF_SMEL  L |  | GO_SENSORY  _PERCEPTION  _OF_SMELL 445 0.438488599 1.3018584 0.035714286 0.622835498 |
| --- | --- | --- |

| GO_CELL  _LEADING  _EDGE |  | GO_CELL_LEA  DING_EDGE 405 -0.437201941 -1.202467708 0.039473684 0.664336561 |
| --- | --- | --- |

| GO_COLL  AGEN_CO  NTAINING  _EXTRAC  ELLULAR_  MATRIX |  | GO_COLLAGE  N_CONTAININ  G_EXTRACEL  LULAR_MATR  IX 408 -0.428603459 -1.174594016 0.040540541 0.664336561 |
| --- | --- | --- |

| GO_CYTO  KINE_ACT  IVITY |  | GO_CYTOKIN  E_ACTIVITY 218 -0.497822516 -1.316329932 0.040540541 0.664336561 |
| --- | --- | --- |

| GO_LEUK  OCYTE_C  HEMOTA  XIS |  | GO_LEUKOCY  TE_CHEMOTA  XIS 221 -0.507632219 -1.343897989 0.04109589 0.664336561 |
| --- | --- | --- |

| GO_SODI  UM_ION_  TRANSPO  RT |  | GO_SODIUM_I  ON_TRANSPO  RT 221 -0.509156372 -1.347933008 0.04109589 0.664336561 |
| --- | --- | --- |

| GO_CYCL  IC_NUCLE  OTIDE_ME  DIATED_S  IGNALING |  | GO_CYCLIC_  NUCLEOTIDE_  MEDIATED_SI  GNALING 209 -0.511905381 -1.344400634 0.041666667 0.664336561 |
| --- | --- | --- |

| GO_EAR_  DEVELOP  MENT |  | GO_EAR_DEV  ELOPMENT 220 -0.507616159 -1.336321184 0.041666667 0.664336561 |
| --- | --- | --- |

| GO_PEPTI  DYL_TYR  OSINE_M  ODIFICAT  ION |  | GO_PEPTIDYL  _TYROSINE_M  ODIFICATION 368 -0.4377907 -1.189205738 0.041666667 0.664336561 |
| --- | --- | --- |

| GO_RESP  ONSE_TO  _MECHAN  ICAL_STI  MULUS |  | GO_RESPONS  E_TO_MECHA  NICAL_STIMU  LUS 211 -0.499559821 -1.312655457 0.041666667 0.664336561 |
| --- | --- | --- |

| GO_TRAN  SPORT_V  ESICLE_M  EMBRANE |  | GO_TRANSPO  RT_VESICLE_  MEMBRANE 209 -0.511555471 -1.343481675 0.041666667 0.664336561 |
| --- | --- | --- |

| GO_FORE  BRAIN_DE  VELOPME  NT |  | GO_FOREBRAI  N_DEVELOPM  ENT 380 -0.435495473 -1.174320714 0.042253521 0.664336561 |
| --- | --- | --- |

| GO_MYEL  OID_LEUK  OCYTE_M  IGRATION |  | GO_MYELOID  _LEUKOCYTE  _MIGRATION 207 -0.511657784 -1.339013852 0.042253521 0.664336561 |
| --- | --- | --- |

| GO_MUSC  LE_CELL_  DEVELOP  MENT |  | GO_MUSCLE_  CELL_DEVELO  PMENT 176 -0.511829649 -1.307217064 0.042857143 0.664336561 |
| --- | --- | --- |

| GO_NEGA  TIVE_REG  ULATION_  OF_LOCO  MOTION |  | GO_NEGATIV  E_REGULATIO  N_OF_LOCOM  OTION 365 -0.441826352 -1.193486434 0.042857143 0.664336561 |
| --- | --- | --- |

| GO_ORGA  NIC_HYDR  OXY_CO  MPOUND_  TRANSPO  RT |  | GO_ORGANIC  _HYDROXY_C  OMPOUND_TR  ANSPORT 249 -0.471124137 -1.249790664 0.042857143 0.664336561 |
| --- | --- | --- |

| GO_POSIT  IVE_REGU  LATION_  OF_PROTE  OLYSIS | | |  | GO_POSITIVE  _REGULATION  _OF_PROTEOL  YSIS 354 -0.44145434 -1.190967736 0.042857143 0.664336561 | |
| --- | --- | --- | --- | --- | --- |
| GO_AMP  A_GLUTA  MATE_RE  CEPTOR_  COMPLEX |  | GO_AMPA_G  LUTAMATE_R  ECEPTOR_CO  MPLEX 26 -0.754585228 -1.501443487 0.043478261 0.664336561 | | |  |

| GO_POSIT  IVE_REGU  LATION_  OF_NERV  OUS_SYS  TEM_PRO  CESS |  | | GO_POSITIVE  _REGULATION  _OF_NERVOU  S_SYSTEM_P  ROCESS 63 -0.598305639 -1.381991202 0.043478261 0.664336561 | |
| --- | --- | --- | --- | --- |
| GO_REGU  LATION_  OF_T_HEL  PER_1_TY  PE_IMMU  NE_RESPO  NSE  GO_SOM  ATIC_DIV  ERSIFICA  TION_OF_  IMMUNE_  RECEPTOR  S_VIA_GE  RMLINE_R  ECOMBIN  ATION_W  ITHIN_A_  SINGLE_L  OCUS | |  | | GO_REGULATI  ON_OF_T_HEL  PER_1_TYPE_I  MMUNE_RESP  ONSE 26 -0.751227772 -1.494762954 0.043478261 0.664336561  GO_SOMATIC  _DIVERSIFICA  TION_OF_IMM  UNE_RECEPTO  RS_VIA_GER  MLINE_RECO  MBINATION_  WITHIN_A_SI  NGLE_LOCUS 63 -0.59608999 -1.376873405 0.043478261 0.664336561 |

| GO_MAIN  TENANCE  _OF_LOC  ATION |  | GO_MAINTEN  ANCE_OF_LO  CATION 333 -0.446517578 -1.209710032 0.044117647 0.664336561 |
| --- | --- | --- |

| GO_REGU  LATION_  OF_MUSC  LE_CONT  RACTION |  | GO_REGULATI  ON_OF_MUSC  LE_CONTRAC  TION 167 -0.518635617 -1.312155117 0.044117647 0.664336561 |
| --- | --- | --- |

| GO_REGU  LATION_  OF_VASO  CONSTRI  CTION |  | GO_REGULATI  ON_OF_VASO  CONSTRICTIO  N 58 -0.617735669 -1.42720885 0.044117647 0.664336561 |
| --- | --- | --- |

| GO_RESP  ONSE_TO  _INTERLE  UKIN_1 |  | GO_RESPONS  E_TO_INTERL  EUKIN_1 204 -0.514779693 -1.332607945 0.044117647 0.664336561 |
| --- | --- | --- |
| GO_ACIDI  C_AMINO  _ACID_TR  ANSPORT |  | GO_ACIDIC_  AMINO_ACID  _TRANSPORT 67 -0.592574672 -1.363485241 0.044776119 0.664336561 |

| GO_AMIN  O_ACID_  BINDING |  | | GO_AMINO_A  CID_BINDING 59 -0.60021085 -1.380759791 0.044776119 0.664336561 | | | |  |
| --- | --- | --- | --- | --- | --- | --- | --- |
| GO_CARD  IOCYTE_D  IFFERENTI  ATION | |  | | | GO_CARDIOC  YTE_DIFFEREN  TIATION 154 -0.535154685 -1.333878169 0.044776119 0.664336561 | | |
| GO_FEAR  _RESPONS  E | | | |  | | GO_FEAR_RES  PONSE 40 -0.647852737 -1.351047245 0.044776119 0.664336561 | |

| GO_LIPOP  OLYSACC  HARIDE_  MEDIATE  D_SIGNAL  ING_PATH  WAY |  | | GO_LIPOPOLY  SACCHARIDE  _MEDIATED_S  IGNALING_PA  THWAY 59 -0.602982605 -1.387136096 0.044776119 0.664336561 | |
| --- | --- | --- | --- | --- |
| GO_NEGA  TIVE_REG  ULATION_  OF_LYMP  HOCYTE_  ACTIVATI  ON | |  | | GO_NEGATIV  E_REGULATIO  N_OF_LYMPH  OCYTE_ACTI  VATION 150 -0.506748217 -1.256630489 0.044776119 0.664336561 |

| GO_PHOT  ORECEPT  OR_CELL_  DIFFEREN  TIATION |  | | GO_PHOTORE  CEPTOR_CELL  _DIFFERENTIA  TION 61 -0.594887949 -1.369186925 0.044776119 0.664336561 | |
| --- | --- | --- | --- | --- |
| GO_PHOT  OTRANSD  UCTION_  VISIBLE_L  IGHT |  | | GO_PHOTOTR  ANSDUCTION  _VISIBLE_LIG  HT 34 -0.723530929 -1.455798598 0.044776119 0.664336561 | |
| GO_POSIT  IVE_REGU  LATION_  OF_DNA_  BINDING | |  | | GO_POSITIVE  _REGULATION  _OF_DNA_BIN  DING 59 -0.61184976 -1.407534613 0.044776119 0.664336561 |

| GO_POSIT  IVE_REGU  LATION_  OF_RELEA  SE_OF_SE  QUESTERE  D_CALCI  UM_ION_I  NTO_CYT  OSOL | | |  | GO_POSITIVE  _REGULATION  _OF_RELEASE  _OF_SEQUES  TERED_CALCI  UM_ION_INTO  _CYTOSOL 40 -0.665956929 -1.38880215 0.044776119 0.664336561 | |
| --- | --- | --- | --- | --- | --- |
| GO_REGU  LATION_  OF_B_CEL  L_PROLIFE  RATION | | |  | GO_REGULATI  ON_OF_B_CEL  L_PROLIFERA  TION 61 -0.605785213 -1.394267938 0.044776119 0.664336561 | |
| GO_REGU  LATION_  OF_T_HEL  PER_CELL  _DIFFERE  NTIATION |  | GO_REGULATI  ON_OF_T_HEL  PER_CELL_DIF  FERENTIATIO  N 34 -0.722214896 -1.453150641 0.044776119 0.664336561 | | |  |

| GO_SODI  UM_ION_  TRANSME  MBRANE_  TRANSPO  RTER_AC  TIVITY |  | GO_SODIUM_I  ON_TRANSME  MBRANE_TRA  NSPORTER_A  CTIVITY 155 -0.514232233 -1.283409827 0.044776119 0.664336561 |
| --- | --- | --- |
| GO_VITA  MIN_TRA  NSPORT |  | GO_VITAMIN  _TRANSPORT 40 -0.664626393 -1.386027418 0.044776119 0.664336561 |

| GO_ADUL  T_BEHAVI  OR |  | GO_ADULT_B  EHAVIOR 142 -0.535706112 -1.330769747 0.045454545 0.664336561 |
| --- | --- | --- |
| GO_AMY  LOID_BET  A_METAB  OLIC_PRO  CESS  GO_DEOX  YRIBONU  CLEOSIDE  _MONOPH  OSPHATE  _METABO  LIC_PROC  ESS |  | GO_AMYLOID  _BETA_META  BOLIC_PROCE  SS 48 -0.623662437 -1.349106296 0.045454545 0.664336561  GO_DEOXYRI  BONUCLEOSID  E_MONOPHOS  PHATE_META  BOLIC_PROCE  SS 13 -0.794681764 -1.407547727 0.045454545 0.664336561 |

| GO_ENDO  DERM_FO  RMATION |  | GO_ENDODER  M_FORMATIO  N 50 -0.606261414 -1.31444729 0.045454545 0.664336561 |
| --- | --- | --- |
| GO_EYE_  MORPHOG  ENESIS |  | GO_EYE_MOR  PHOGENESIS 149 -0.519698482 -1.284735028 0.045454545 0.664336561 |

| GO_IMMU  NOLOGIC  AL_MEM  ORY_PRO  CESS |  | GO_IMMUNOL  OGICAL_MEM  ORY_PROCES  S 13 -0.801460845 -1.419554899 0.045454545 0.664336561 | | |  |
| --- | --- | --- | --- | --- | --- |
| GO_IMPO  RT_ACRO  SS_PLAS  MA_MEM  BRANE |  | GO_IMPORT_  ACROSS_PLA  SMA_MEMBR  ANE 120 -0.545782806 -1.34129085 0.045454545 0.664336561 | | |  |
| GO_INFLA  MMATOR  Y_RESPO  NSE_TO_  ANTIGENI  C_STIMUL  US | | |  | GO_INFLAMM  ATORY_RESP  ONSE_TO_AN  TIGENIC_STIM  ULUS 50 -0.59864866 -1.297941929 0.045454545 0.664336561 | |

| GO_INTR  ASPECIES  _INTERAC  TION_BET  WEEN_OR  GANISMS |  | GO_INTRASPE  CIES_INTERA  CTION_BETW  EEN_ORGANIS  MS 52 -0.624777973 -1.367942585 0.045454545 0.664336561 | | |  |
| --- | --- | --- | --- | --- | --- |
| GO_MEMB  RANE_DEP  OLARIZA  TION | | |  | GO_MEMBRA  NE_DEPOLARI  ZATION 90 -0.602271434 -1.43460144 0.045454545 0.664336561 | |

| GO_NEGA  TIVE_REG  ULATION_  OF_TRAN  SPORTER_  ACTIVITY |  | GO_NEGATIV  E_REGULATIO  N_OF_TRANS  PORTER_ACTI  VITY 90 -0.579415021 -1.380157811 0.045454545 0.664336561 |
| --- | --- | --- |
| GO_NEGA  TIVE_REG  ULATION_  OF_VASC  ULAR_AS  SOCIATE  D_SMOOT  H_MUSCL  E_CELL_M  IGRATION |  | GO_NEGATIV  E_REGULATIO  N_OF_VASCU  LAR_ASSOCI  ATED_SMOOT  H_MUSCLE_C  ELL_MIGRATI  ON 13 -0.798335692 -1.414019599 0.045454545 0.664336561 |

| GO_NEUR  AL_RETIN  A_DEVEL  OPMENT |  | | GO_NEURAL_  RETINA_DEVE  LOPMENT 68 -0.591081894 -1.353641137 0.045454545 0.664336561 | |
| --- | --- | --- | --- | --- |
| GO_POSIT  IVE_REGU  LATION_  OF_ANIO  N_TRANS  PORT | |  | | GO_POSITIVE  _REGULATION  _OF_ANION_T  RANSPORT 50 -0.600748434 -1.302494491 0.045454545 0.664336561 |

| GO_POSIT  IVE_REGU  LATION_  OF_INTER  LEUKIN_8  _PRODUC  TION |  | | GO_POSITIVE  _REGULATION  _OF_INTERLE  UKIN_8_PROD  UCTION 49 -0.605551307 -1.303032847 0.045454545 0.664336561 | |
| --- | --- | --- | --- | --- |
| GO_POSIT  IVE_REGU  LATION_  OF_TISSU  E_REMOD  ELING |  | | GO_POSITIVE  _REGULATION  _OF_TISSUE_  REMODELING 39 -0.664069179 -1.369970812 0.045454545 0.664336561 | |
| GO_REGU  LATION_  OF_MEMB  RANE_DEP  OLARIZA  TION | |  | | GO_REGULATI  ON_OF_MEMB  RANE_DEPOL  ARIZATION 48 -0.622947911 -1.347560634 0.045454545 0.664336561 |

| GO_REGU  LATION_  OF_NEUR  ONAL_SY  NAPTIC_P  LASTICIT  Y |  | | GO_REGULATI  ON_OF_NEUR  ONAL_SYNAP  TIC_PLASTICI  TY 52 -0.603860692 -1.322144493 0.045454545 0.664336561 | | | |  |
| --- | --- | --- | --- | --- | --- | --- | --- |
| GO_REGU  LATION_  OF_T_CEL  L_RECEPT  OR_SIGN  ALING_PA  THWAY | | | |  | | GO_REGULATI  ON_OF_T_CE  LL_RECEPTOR  _SIGNALING_  PATHWAY 39 -0.658450664 -1.358379849 0.045454545 0.664336561 | |
| GO_REGU  LATION_  OF_TRAN  SCRIPTIO  N_REGUL  ATORY_R  EGION_DN  A_BINDIN  G | |  | | | GO_REGULATI  ON_OF_TRAN  SCRIPTION_RE  GULATORY_R  EGION_DNA_B  INDING 52 -0.602781671 -1.319781992 0.045454545 0.664336561 | | |

| GO_RESP  ONSE_TO  _CALCIU  M_ION |  | GO_RESPONS  E_TO_CALCIU  M_ION 147 -0.509121614 -1.255443327 0.045454545 0.664336561 |
| --- | --- | --- |

| GO_T_TU  BULE |  | GO_T_TUBUL  E 52 -0.60975456 -1.335049035 0.045454545 0.664336561 |
| --- | --- | --- |
| GO_VISU  AL_BEHA  VIOR |  | GO_VISUAL_B  EHAVIOR 52 -0.625088147 -1.368621709 0.045454545 0.664336561 |

| GO_E_BO  X_BINDIN  G |  | GO_E_BOX_BI  NDING 51 -0.613636722 -1.331929496 0.046153846 0.664336561 | | |  |
| --- | --- | --- | --- | --- | --- |
| GO_G_PR  OTEIN_CO  UPLED_NE  UROTRAN  SMITTER_  RECEPTOR  _ACTIVIT  Y | | |  | GO_G_PROTEI  N_COUPLED_  NEUROTRANS  MITTER_RECE  PTOR_ACTIVI  TY 41 -0.66521044 -1.400817313 0.046153846 0.664336561 | |

| GO_GRAN  ULOCYTE  _MIGRATI  ON |  | GO_GRANULO  CYTE_MIGRA  TION 139 -0.513372593 -1.273302941 0.046153846 0.664336561 |
| --- | --- | --- |

| GO_NEUR  ON_MIGR  ATION |  | GO_NEURON_  MIGRATION 160 -0.504774452 -1.257527779 0.046153846 0.664336561 |
| --- | --- | --- |

| GO_NEUR  OTRANSM  ITTER_TR  ANSMEM  BRANE_T  RANSPOR  TER_ACTI  VITY | |  | | GO_NEUROTR  ANSMITTER_T  RANSMEMBR  ANE_TRANSP  ORTER_ACTIV  ITY 51 -0.593067078 -1.287282044 0.046153846 0.664336561 |
| --- | --- | --- | --- | --- |
| GO_POSIT  IVE_REGU  LATION_  OF_INTER  LEUKIN_1  2_PRODU  CTION |  | | GO_POSITIVE  _REGULATION  _OF_INTERLE  UKIN_12_PRO  DUCTION 36 -0.691186572 -1.408627697 0.046153846 0.664336561 | |
| GO_POSIT  IVE_REGU  LATION_  OF_NITRI  C_OXIDE_  METABOL  IC_PROCE  SS |  | | GO_POSITIVE  _REGULATION  _OF_NITRIC_  OXIDE_META  BOLIC_PROCE  SS 44 -0.617691147 -1.319672902 0.046153846 0.664336561 | |

| GO_REGU  LATION_  OF_OSTE  OBLAST_  DIFFEREN  TIATION |  | GO_REGULATI  ON_OF_OSTE  OBLAST_DIFF  ERENTIATION 117 -0.545024412 -1.340156207 0.046153846 0.664336561 |
| --- | --- | --- |
| GO_SH3_  SH2_ADA  PTOR_AC  TIVITY |  | GO_SH3_SH2  _ADAPTOR_A  CTIVITY 53 -0.604979849 -1.338044398 0.046153846 0.664336561 |

| GO_CERE  BELLAR_C  ORTEX_D  EVELOPM  ENT |  | GO_CEREBELL  AR_CORTEX_  DEVELOPMEN  T 47 -0.62626306 -1.343347235 0.046875 0.664336561 | | |  |
| --- | --- | --- | --- | --- | --- |
| GO_CYTO  KINE_BIND  ING | | |  | GO_CYTOKIN  E_BINDING 129 -0.55318146 -1.363280701 0.046875 0.664336561 | |

| GO_REGU  LATION_  OF_BIOMI  NERALIZA  TION |  | GO_REGULATI  ON_OF_BIOMI  NERALIZATIO  N 92 -0.573681296 -1.356582499 0.046875 0.664336561 | | |  |
| --- | --- | --- | --- | --- | --- |
| GO_REGU  LATION_  OF_CARDI  AC_COND  UCTION | | |  | GO_REGULATI  ON_OF_CARD  IAC_CONDUC  TION 71 -0.574733074 -1.325369872 0.046875 0.664336561 | |

| GO_REGU  LATION_  OF_CELL_  KILLING |  | GO_REGULATI  ON_OF_CELL_  KILLING 94 -0.616602892 -1.462734087 0.046875 0.664336561 |
| --- | --- | --- |

| GO_RESP  ONSE_TO  _ESTRADI  OL |  | GO_RESPONS  E_TO_ESTRA  DIOL 135 -0.520358075 -1.29089255 0.046875 0.664336561 |
| --- | --- | --- |

| GO_ADEN  YLATE_C  YCLASE_  ACTIVATI  NG_G_PR  OTEIN_CO  UPLED_RE  CEPTOR_  SIGNALIN  G_PATH  WAY | | |  | GO_ADENYLA  TE_CYCLASE  _ACTIVATING  _G_PROTEIN_  COUPLED_REC  EPTOR_SIGNA  LING_PATHW  AY 136 -0.509388631 -1.258887921 0.047619048 0.664336561 | |
| --- | --- | --- | --- | --- | --- |
| GO_CARD  IAC_MUS  CLE_CELL  _DIFFERE  NTIATION |  | GO_CARDIAC  _MUSCLE_CE  LL_DIFFERENT  IATION 122 -0.550536616 -1.341558002 0.047619048 0.664336561 | | |  |

| GO_CELL  ULAR_RES  PONSE_T  O_LIGHT_  STIMULUS  GO_GTP_  RHO_BIND  ING |  | GO_CELLULA  R_RESPONSE_  TO_LIGHT_ST  IMULUS 127 -0.532025404 -1.299492331 0.047619048 0.664336561  GO_GTP_RHO  _BINDING 22 0.722758916 1.402149599 0.047619048 0.664336561 |
| --- | --- | --- |
| GO_NEGA  TIVE_REG  ULATION_  OF_ION_T  RANSME  MBRANE_  TRANSPO  RT |  | GO_NEGATIV  E_REGULATIO  N_OF_ION_TR  ANSMEMBRA  NE_TRANSPO  RT 104 -0.567642842 -1.384699883 0.047619048 0.664336561 |

| GO_PHEN  OTYPIC_S  WITCHIN  G |  | GO_PHENOTY  PIC_SWITCHI  NG 12 -0.820954692 -1.446979384 0.047619048 0.664336561 | | |  |
| --- | --- | --- | --- | --- | --- |
| GO_PHOT  ORECEPT  OR_CELL_  DEVELOP  MENT |  | GO_PHOTORE  CEPTOR_CELL  _DEVELOPME  NT 46 -0.622227847 -1.309530012 0.047619048 0.664336561 | | |  |
| GO_POSIT  IVE_REGU  LATION_  OF_CALCI  UM_MEDI  ATED_SIG  NALING | | |  | GO_POSITIVE  _REGULATION  _OF_CALCIU  M_MEDIATED  _SIGNALING 43 -0.623856559 -1.305973631 0.047619048 0.664336561 | |

| GO_RECE  PTOR_INT  ERNALIZA  TION |  | GO_RECEPTO  R_INTERNALIZ  ATION 112 -0.556498449 -1.350757749 0.047619048 0.664336561 |
| --- | --- | --- |
| GO_REGU  LATION_  OF_GRAN  ULOCYTE  _CHEMOT  AXIS |  | GO_REGULATI  ON_OF_GRAN  ULOCYTE_CH  EMOTAXIS 43 -0.662616219 -1.387112627 0.047619048 0.664336561 |

| GO_REGU  LATION_  OF_LEUK  OCYTE_C  HEMOTA  XIS |  | GO_REGULATI  ON_OF_LEUK  OCYTE_CHEM  OTAXIS 113 -0.541846942 -1.315817926 0.047619048 0.664336561 |
| --- | --- | --- |
| GO_U1_S  NRNP |  | GO_U1_SNRN  P 21 0.757906324 1.485107765 0.047619048 0.664336561 |

| GO_AMIN  E_TRANS  PORT |  | GO_AMINE_T  RANSPORT 97 -0.593245462 -1.410314707 0.048387097 0.664336561 |
| --- | --- | --- |

| GO_B_CE  LL_DIFFER  ENTIATIO  N |  | GO_B_CELL_D  IFFERENTIATI  ON 132 -0.535718774 -1.313781842 0.048387097 0.664336561 |
| --- | --- | --- |

| GO_B_CE  LL_PROLIF  ERATION |  | GO_B_CELL_P  ROLIFERATIO  N 95 -0.589999421 -1.391034524 0.048387097 0.664336561 |
| --- | --- | --- |

| GO_CD4_  POSITIVE  _ALPHA_  BETA_T_  CELL_AC  TIVATION  GO_CD4_  POSITIVE  _OR_CD8  _POSITIV  E_ALPHA  _BETA_T_  CELL_LIN  EAGE_CO  MMITMEN  T |  | GO_CD4_POSI  TIVE_ALPHA_  BETA_T_CELL  _ACTIVATION 91 -0.611720996 -1.441244866 0.048387097 0.664336561  GO_CD4_POSI  TIVE_OR_CD8  _POSITIVE_A  LPHA_BETA_T  _CELL_LINEA  GE_COMMITM  ENT 20 -0.756214802 -1.375582517 0.048387097 0.664336561 |
| --- | --- | --- |

| GO_COLL  AGEN_ME  TABOLIC_  PROCESS |  | GO_COLLAGE  N_METABOLI  C_PROCESS 109 -0.558756263 -1.349091364 0.048387097 0.664336561 |
| --- | --- | --- |

| GO_INTER  LEUKIN_1  _PRODUC  TION |  | GO_INTERLEU  KIN_1_PRODU  CTION 114 -0.541556597 -1.317955925 0.048387097 0.664336561 | | |  |
| --- | --- | --- | --- | --- | --- |
| GO_MOLT  ING_CYCL  E | | |  | GO_MOLTING  _CYCLE 109 -0.556845247 -1.344477305 0.048387097 0.664336561 | |

| GO_POSIT  IVE_REGU  LATION_  OF_PROD  UCTION_  OF_MOLE  CULAR_M  EDIATOR_  OF_IMMU  NE_RESPO  NSE |  | GO_POSITIVE  _REGULATION  _OF_PRODUC  TION_OF_MO  LECULAR_ME  DIATOR_OF_I  MMUNE_RESP  ONSE 97 -0.582342471 -1.384395169 0.048387097 0.664336561 |
| --- | --- | --- |

| GO_POTA  SSIUM_C  HANNEL_  COMPLEX |  | GO_POTASSI  UM_CHANNEL  _COMPLEX 96 -0.612158391 -1.45123673 0.048387097 0.664336561 |
| --- | --- | --- |

| GO_REGU  LATION_  OF_CALCI  UM_ION_  TRANSPO  RT_INTO_  CYTOSOL |  | GO_REGULATI  ON_OF_CALC  IUM_ION_TRA  NSPORT_INTO  _CYTOSOL 98 -0.611340439 -1.460958965 0.048387097 0.664336561 |
| --- | --- | --- |

| GO_REGU  LATION_  OF_DNA_  BINDING |  | GO_REGULATI  ON_OF_DNA_  BINDING 125 -0.527006141 -1.280109548 0.048387097 0.664336561 |
| --- | --- | --- |
| GO_REGU  LATION_  OF_ORGA  N_GROW  TH |  | GO_REGULATI  ON_OF_ORGA  N_GROWTH 109 -0.542923834 -1.310864692 0.048387097 0.664336561 |

| GO_REGU  LATION_  OF_PHAG  OCYTOSI  S |  | GO_REGULATI  ON_OF_PHAG  OCYTOSIS 93 -0.576760783 -1.360938328 0.048387097 0.664336561 |
| --- | --- | --- |
| GO_REGU  LATION_  OF_PHOS  PHOLIPID_  METABOL  IC_PROCE  SS |  | GO_REGULATI  ON_OF_PHOS  PHOLIPID_ME  TABOLIC_PRO  CESS 91 -0.588094877 -1.385580563 0.048387097 0.664336561 |

| GO_REGU  LATION_  OF_SYNA  PSE_ASSE  MBLY |  | GO_REGULATI  ON_OF_SYNA  PSE_ASSEMB  LY 107 -0.56667095 -1.370920081 0.048387097 0.664336561 |
| --- | --- | --- |

| GO_RESP  ONSE_TO  _CHEMOK  INE |  | GO_RESPONS  E_TO_CHEMO  KINE 95 -0.608309916 -1.434204958 0.048387097 0.664336561 |
| --- | --- | --- |

| GO_SARC  OLEMMA |  | GO_SARCOLE  MMA 134 -0.522555568 -1.290757447 0.048387097 0.664336561 |
| --- | --- | --- |

| GO_SYNA  PTIC_VESI  CLE_MEM  BRANE |  | GO_SYNAPTI  C_VESICLE_M  EMBRANE 109 -0.556108341 -1.342698078 0.048387097 0.664336561 |
| --- | --- | --- |

qvalues rank leading_edge core_enrichment

ACR2B/LGALS3/PDE2A/LYN/CA

CNB2/CALCRL/SLC30A10/TRPV4

/CACNA1C/IL16/SLC39A5/GJC2

/GJA1/CXCL12/CATSPER1/GRIN

2C/TRPM4/PDGFRB/STC1/FGF14

/SPINK1/CACNA1H/AGT/CATSP

ER3/CACNA1F/PLCL2/CNNM2/R

CVRN/ATP2C2/CACNB4/ASIC1/

SLC41A2/SLC24A4/CASQ2/GJA

4/THY1/CEMIP/SLC39A8/MT3/T

RPV2/CACNA2D1/F2R/KCNN4/P2

RX2/NTSR1/CRACR2A/CCR1/KC

NE2/CAMK2A/RGS9/PRKCB/CAC

NG1/HOMER1/STC2/HRC/PLCD4

/DYSF/MCUB/CD33/SLC30A8/T

RPM5/EPO/DRD2/TMEM37/PKD1L

3/LILRA5/BDKRB1/CD19/JPH1/C

ACNB1/REM1/GRIN2D/NOS1AP/F

2RL3/GRIN3A/ADRA2A/RGN/CC

L8/CLDN16/STRIT1/PKD2L1/CAC

NG5/APLNR/CASQ1/ATP1A2/CO

RO1A/TRPM2/BSPRY/CD84/PTPN

6/CRHR1/CCL3/CCL19/JSRP1/S

TAC/MCHR1/P2RX5/PTGS2/RRA

D/CASR/VDR/CACNA1E/SLC8A1

/LILRB2/FAM155A/CCL5/TRPM8

/CDH23/CACNA2D3/GEM/GPM6

A/TRPC6/IL13/CAV3/TMEM163 /SLC39A12/CACNA2D4/ITPR2/T

RPM3/CLIC2/HPCA/TRPV6/PLCH

tags=40%, 2/PIK3CG/CCL21/GRM6/NMUR2 list=24%, /LILRB1/SLC39A2/P2RY6/CALH

0.497710826 13358 signal=30% M1/CYSLTR1/PLCH1/HTR2A/PTP

| 0.497710826 13085 |  | tags=47%,  list=23%,  signal=36% |  | /TRPV4/SLC4A11/CACNA1C/GJ  A3/AQP10/GJC2/ANO7/GJA1/  CATSPER1/SLC26A9/GRIN2C/TM  C8/TRPM4/ATP5MGL/GABRE/CA  CNA1H/CHRNA5/CATSPER3/CLIC  6/CACNA1F/HPN/CACNB4/ASIC  1/SLC24A4/KCND3/TRPV2/SCNN  1G/ANO1/CACNA2D1/GABRG3/  KCNN4/KCNAB1/KCNJ1/KCNU1/  KCNT1/P2RX2/GRIK5/P2RX6/GA  BRB3/KCNMA1/ANO2/KCNE1/KC  NE2/GJC1/CYBB/CACNG1/KCNK  6/KCNMB4/SCN4A/KCNN3/GABR  Q/HCN2/TRPM5/TMEM37/PKD1L  3/AQP1/KCNF1/JPH1/CACNB1/  GABRA5/GRIN2D/GRIN3A/KCNQ3  /SCN1B/ANO4/KCNJ8/KCNV1/S  LC26A7/LRRC38/KCNV2/CLCNKB  /PKD2L1/CACNG5/SCN4B/AQP1  2A/NOX5/SLC17A7/CHRNA3/CL  CNKA/GJB3/KCNA2/KCNMB1/AB  CC9/BSND/GRID1/PANX2/SCN2B  /TRPM2/KCNK1/SCN9A/CLCA4/  KCNE1B/P2RX5/SCNN1B/SCNN1A  /KCNH7/CACNA1E/CLDN4/FAM1  55A/TRPM8/SLC26A8/GABRA4/  CACNA2D3/ABCC8/GPM6A/TRP  C6/GJB4/GABRD/APOL1/KCNC1  /KCNK10/CLIC3/CACNA2D4/KC  NH2/GLRB/CLIC5/ITPR2/GABRB2  /TRPM3/CLIC2/AQP4/KCNK9/H  CN4/KCNJ10/KCNJ11/GRIA1/TR  PV6/KCNK18/PRF1/NMUR2/KCNK |
| --- | --- | --- | --- | --- |

| 0.497710826 14613 |  | tags=40%,  list=26%,  signal=30% |  | 1/TREM2/FZD5/PYDC1/C1QTNF4  /ADORA2B/ZP3/SDC1/PARD6A/  FGG/CD274/CD34/BLK/NNAT/T  GFB3/PANX1/C1QTNF1/EGFR/KC  NB1/TGFB1/FGFR1/P2RX4/ITGB2  /FCER1G/CACNB2/TRPV4/RAB15  /SYTL4/GJA1/LGALS9B/CXCL12  /TRPM4/MYRIP/IL1RL1/INHBA/C  ACNA1H/FGR/SYK/AGT/UNC13D  /CSF1R/CRLF2/AIF1/LGALS9/A  CHE/UCN3/NPR1/ANO1/F2R/PLA  2R1/KCNN4/OR51E2/FRMD4A/NL  RP3/NTSR1/CCK/CYP19A1/CD17  7/DOC2B/OSM/HLA-  F/UNC13A/ORM1/ADORA1/P2RY  2/TFR2/IL1A/RPH3AL/ADAM8/I  L6/AVPR1A/CD33/SLC30A8/P2R  Y1/WNT5A/DRD2/CDK5R2/RAB2  7B/USP50/CD244/LILRA5/INHBB  /AQP1/TGFB2/KMO/TNFRSF11A  /HCAR2/CLEC4E/PTAFR/TNFSF4  /FGB/OXTR/HAVCR2/TRH/PANX  2/CASP1/SYT10/CCL3/CCL19/S  ORL1/SOX11/CASR/CHIA/FFAR2  /GPR68/VSNL1/SLC6A1/TWIST1  /TNFRSF4/IL13/FOXL2/KISS1/IL  10/CD160/GALR1/IGF1/PTGER4/  GRP/IL1B/CADPS/CADM1/PRRT2  /NOD2/IGHD/ADCY8/TLR8/ADC  YAP1/SYT1/MYB/PAEP/ILDR1/G  ATA3/CD38/SYT4/IL26/CD2/CY  P4A11/HTR2B/SYT9/TAC1/TNF/  NLRP7/ISL1/RASGRP1/PTPN22/O |
| --- | --- | --- | --- | --- |

| 0.497710826 11866 |  | tags=34%,  list=21%,  signal=27% |  | UCN3/ATP1A3/CACNA2D1/GABR  G3/KCNN4/MAPT/MIR26A2/P2R  X2/GRIK5/NTSR1/CCK/GLRX/P2  RX6/GABRB3/KCNMA1/KCNE1/K  CNE2/ADORA1/GJC1/CLDN19/S  HANK1/KCNK6/CNTNAP1/KCNMB  4/SCN4A/GABRQ/HCN2/FGF12/  DRD2/GABRA5/GRIN2D/NOS1AP/  GRIN3A/KCNQ3/SCN1B/KCNJ8/B  CO2/SCN4B/SLC26A3/SLC17A7  /CHRNA3/KCNA2/ATP1A2/GRID  1/SCN2B/RGS7BP/KCNK1/SCN9A  /NLGN4X/KCNE1B/P2RX5/GPR88  /KCNH7/CACNA1E/SLC8A1/CEL  F4/GABRA4/GABRD/CAV3/KCNC  1/KCNK10/KCNH2/GLRB/GABRB2  /RELN/GRM1/KCNK9/HCN4/KCN  J10/KCNJ11/GRIA1/SLC34A1/SE  Z6/KCNK18/KCNK13/GJA5/INSY  N2A/SCN7A/NTRK3/ADCYAP1/  ADRA1A/P2RX1/MYOC/CACNG4  /GABRA3/PLN/NETO1/GABRA2/  CHRNA4/GABRR2/KCNK3/NRXN1  /GRIK1/CUX2/OPRD1/CHRNA9/  CHRNA2/KCNJ3/GLRA2/TAFA4/  TAC1/GRIA4/HSH2D/ADRB1/RYR  2/KCNH6/GJD2/TRDN/GABRA1/  GRIN1/CNR2/GRID2/CACNA1G/G  RIN2A/TCL1A/CHRNA6/ASIC2/C  ACNA1I/GRIA3/GABRR3/BEST2/  DRD1/SCN1A/RIMS2/OPRM1/SC  N2A/GLRA3/RIMS1/GABRA6/GR  M5/GABRG2/KCND2/KCNH5/SCN |
| --- | --- | --- | --- | --- |

| 0.497710826 11604 |  | tags=40%,  list=21%,  signal=32% |  | 2/DLG2/F2R/DLGAP1/GABRG3/  CNKSR2/EFNB2/NRGN/GRIK5/P2R  X6/LRRC7/GABRB3/KCNMA1/UN  C13A/ADORA1/CAMK2A/RGS9/  SHANK1/CNTNAP1/HOMER1/SLC  6A9/GABRQ/PDLIM4/P2RY1/HCL  S1/FOSL1/DRD2/GRM2/MX2/CA  DM3/DNM3/GABRA5/GRIN2D/GR  IN3A/KCTD16/ADAM22/CACNG5  /EPHA4/NLGN4Y/CHRNA3/SYNDI  G1/KCNA2/SRCIN1/SYT11/LRRC  4/GRID1/SNCAIP/CDH2/SLC6A3  /RGS7BP/IQSEC3/NLGN4X/PCDH  17/SLITRK2/FAIM2/GSG1L/SLC8  A1/SLC6A1/LRFN2/GABRA4/GP  M6A/GABRD/ARC/SH2D5/BAAL  C/KCNC1/GRM3/CABP1/GLRB/C  DH9/SLC6A2/LHFPL4/GABRB2/G  RM1/CHRM3/SHISA9/GPC4/LRFN  5/GABBR2/GRIA1/SHISA8/CALB  2/EPHB2/PTPRO/PRRT2/ITGB3/N  TNG2/IGSF21/ADCY8/LRRTM1/I  NSYN2A/LRRC4C/NTRK3/HTR2A  /SYT1/ADRA1A/P2RX1/HTR1B/  CACNG4/GABRA3/SHISA6/PRKC  G/ADCY1/PLPPR4/NETO1/CNTN  AP4/GABRA2/CNTN2/CHRNA4/G  ABRR2/SYT6/ATP2B2/CNTN5/NR  XN1/SNAP91/GRIK1/OPRD1/CHR  NA9/CHRNA2/KCTD8/KCNJ3/KC  NA3/EPHA7/CACNG3/KCNJ4/GL  RA2/PCDH8/GRIA4/SLC1A2/ANK  1/LRRTM2/SLITRK1/CACNG7/GA |
| --- | --- | --- | --- | --- |

| 0.497710826 15532 |  | tags=31%,  list=27%,  signal=22% |  | D/IGFBP7/PTCH2/ACVRL1/ADA  M15/NLGN3/CXCL16/MACF1/RP  S6KA1/ALCAM/DRAXIN/LRP1/S  100A9/TRIM46/IQGAP1/DCLK1/  PLXNA3/FBP1/FGF13/AKAP6/NR  G3/OSGIN2/CXCR4/DCUN1D3/E  GFR/TGFB1/AUTS2/SEMA7A/SE  MA5A/RND2/PSRC1/SEMA3A/P  OU4F3/SLC44A4/CDA/FLRT3/M  UC12/MAP2/SESN2/GJA1/SFRP2  /HRG/CXCL12/SEMA3G/KIF26A  /TMC8/HAMP/CDKN1A/CDHR2/I  NHBA/OLFM1/GREM1/AGT/WT1  /NRP1/HPN/CLSTN3/MINAR1/FR  ZB/NGF/ISLR2/NRN1L/MSX1/TN  FRSF12A/BST2/MT3/NPR1/TRPV  2/MYOCD/MAPT/SEMA3F/SPOC  K1/LEF1/SGK1/KRT17/FAM107A  /UNC13A/PI16/SLIT2/FN1/CRAB  P2/AVPR1A/COBL/SH3GL2/H1-  5/HCLS1/WNT5A/RERG/CYFIP2/  EMX1/CCN3/BDKRB1/SEMA6B/T  GFB2/SYT14P1/PAPPA2/SEMA5B  /SPHK1/IGFBP1/SOX17/MAP1B/  SLIT3/SFN/ESR2/RASGRP2/FSTL  4/PLXNA4/FOXL2/CAV3/SEMA3  E/MIR199A1/SOCS2/IGF1/MIR23  A/NTN1/BARHL2/NTRK3/SYT1/  ADRA1A/PAK5/CPNE5/CD38/DC  STAMP/SYT4/DSCAM/SLIT1/SY  T2/HNF4A/EPHA7/ITGA4/CPNE6  /CACNG7/TNR/PRSS2/REG1A/A  GTR2/RIMS2/RIMS1/POU4F2/MIR |
| --- | --- | --- | --- | --- |

| 0.497710826 11714 |  | tags=42%,  list=21%,  signal=33% |  | 9B/IGLV1-47/KCNN4/IGKV4-  1/HLA-DPA1/HLA-  DRB5/CARD9/THEMIS2/IGLV6-  57/CMKLR1/LILRB4/FYB2/C5AR2  /MUC5B/PRKCB/RFTN1/IGHV3-  73/CLEC4A/MUC1/LILRA4/CLEC  4C/IGLV3-21/FPR3/HLA-  DQA2/CYFIP2/MNDA/CD19/WA  S/IGKV3-  20/FYB1/IGLC2/HCK/BTNL8/IGL  V2-  8/CLEC4E/IGHA1/MAPK10/HLA-  DQB1/ELMO1/GCSAML/IGHM/IN  PP5D/IGHA2/NFAM1/IGHV4-  59/RUNX1/IGHV5-51/IGLV2-  14/IGHG2/IGHV1-  45/IGHG1/LAT2/IGHV4-  39/IGHE/PTPN6/KIR2DL1/IGHV3-  23/IGKC/MUC3A/FOXP3/ICOSLG  /HLA-DQA1/CD79A/HLA-  DQB2/NCKAP1L/VAV1/FFAR2/LI  LRB2/IGLV2-23/IGHV3-  35/PSMB9/CD300A/IGHV4-  31/IGLV2-11/IGHV4-34/IGKV3-  15/IGLC7/IGHV3-  48/CD209/LCP2/HHLA2/IGLV3-  19/IGKV1-5/CD28/IGHV3-  11/IGHV1-69D/IGHV3-  21/KLHL6/MYO1G/CD160/IGLV1  -44/IGHV1-  3/FCGR3A/FPR1/IGHG3/IGHV4-  61/IGHV1-  18/IGLC6/NFATC2/IGHV3- |
| --- | --- | --- | --- | --- |

| 0.497710826 14175 |  | tags=52%,  list=25%,  signal=39% |  | N2/CXCR4/THBS4/DAPK2/CD34  /CD74/TGFB1/FLT1/CSF1/HMO  X1/ANGPT4/SLC16A3/P2RX4/PG  F/NBL1/ANGPT1/NKX2-  3/KIT/ITGB2/FCER1G/LGALS3/L  YN/PREX1/BST1/TRPV4/RET/S1  PR1/AIRE/IL16/ATP1B2/IGLV1-  51/COL1A1/ARTN/PROS1/FOXJ  1/CXCL12/THBD/PLA2G7/TRPM4  /ELANE/PECAM1/VPREB3/GREM1  /SYK/ANXA1/ANGPT2/CX3CR1  /ITGA6/CCL18/C3AR1/AIF1/TH  Y1/LGALS9/CCL14/HRH1/IGLV1  -  47/CHGA/ADD2/GPR183/CCL20  /IGKV4-  1/ITGA1/CYP19A1/RAC2/CD177  /IGLV6-  57/CCR1/MMP1/CMKLR1/ADOR  A1/C5AR2/CCL13/SLIT2/FN1/M  MP28/IL1A/ADAM8/IL6/THBS1/  DYSF/SCG2/IL1RN/SLC16A8/IGL  V3-  21/WNT5A/CSF3R/SELPLG/ITGA  5/SLC7A7/TNFSF18/IL27RA/CC  N3/CEACAM3/CD244/BDKRB1/E  DN2/TGFB2/CCL7/IGKV3-  20/TNFRSF11A/IGLC2/SERPINE1/  HCK/CCL8/IGLV2-  8/IGHA1/RIPK3/PTAFR/GCSAML  /IGHM/ITGAX/INPP5D/SPNS2/T  NFRSF10D/IGHA2/IGHV4-  59/DOK2/IGLV2- |
| --- | --- | --- | --- | --- |

| 0.497710826 13130 |  | tags=43%,  list=23%,  signal=33% |  | B2/KCNH3/SLC30A10/TRPV4/SL  C4A11/CACNA1C/SLC39A5/ATP  1B2/SLC9A3/CATSPER1/GRIN2C  /SLC4A5/TRPM4/CACNA1H/CA  TSPER3/SLC31A2/CACNA1F/CNN  M2/HPN/ATP2C2/SLC1A3/CACN  B4/ASIC1/SLC24A4/KCND3/SLC  39A8/TRPV2/SLC6A12/SCNN1G/  ATP1A3/CACNA2D1/KCNN4/KCN  AB1/KCNJ1/KCNU1/KCNT1/GRIK  5/SLC4A4/KCNMA1/KCNE1/KCN  E2/CACNG1/KCNK6/SLC18A1/K  CNMB4/SCN4A/SLC6A9/KCNN3/  HCN2/SLC30A8/TRPM5/SLC6A13  /TMEM37/PKD1L3/AQP1/KCNF1  /JPH1/CACNB1/SLC18A2/SLC6A  20/GRIN2D/GRIN3A/KCNQ3/SCN  1B/KCNJ8/KCNV1/CLDN16/LRRC  38/KCNV2/PKD2L1/CACNG5/SC  N4B/SLC17A7/KCNA2/KCNMB1/  ATP1A2/ABCC9/SCN2B/SLC6A3  /TRPM2/MFSD2A/KCNK1/SCN9A  /ATP12A/KCNE1B/SCNN1B/SCN  N1A/KCNH7/CACNA1E/SLC8A1/  FAM155A/SLC5A4/SLC6A1/TRP  M8/SLC34A2/CACNA2D3/ABCC8  /GPM6A/TRPC6/KCNC1/KCNK10  /SLC39A12/SLC9C2/CACNA2D4  /KCNH2/SLC6A2/SLC1A1/ITPR2  /TRPM3/KCNK9/HCN4/KCNJ10/  KCNJ11/SLC5A12/SLC9A7/TRPV  6/SLC34A1/KCNK18/KCNK13/SL  C39A2/KCNJ6/KCNT2/CALHM1/ |
| --- | --- | --- | --- | --- |

| 0.497710826 15197 |  | tags=49%,  list=27%,  signal=36% |  | BAX/CX3CL1/ARRB2/RAMP3/NL  GN3/STAC3/ATP2A1/SCN5A/A  NO6/KCNE5/WNK2/TREM2/HCRT  /PLCG2/AKAP6/EHD3/ATP1B1/S  TAC2/ABCB1/CD4/ATPSCKMT/F  ABP3/CTSS/P2RX4/ANK2/LGALS  3/CACNB2/ERFE/ABCB4/GALR2  /ATP1B2/NKX2-  5/GJC2/CXCL12/PDGFRB/STC1/  FGF14/AGT/ATP2C2/ABCA7/TH  Y1/CEMIP/TRPV2/CACNA2D1/F2  R/PLA2R1/P2RX2/NTSR1/CRACR  2A/CCK/GLRX/CCR1/KCNE1/KC  NE2/ADORA1/CAMK2A/RGS9/H  OMER1/AVPR1A/P2RY1/FGF12/D  RD2/LILRA5/BDKRB1/CD19/KMO  /TNFRSF11A/NOS1AP/F2RL3/AD  RA2A/SCN1B/RGN/LRRC38/PTAF  R/STRIT1/SCN4B/APLNR/CASQ1  /KCNMB1/TRH/CCL3/STAC/MC  HR1/P2RX5/CASR/CCL5/SLC6A1  /TRPC6/ARC/IL13/RAB3B/KCNC  1/KCNH2/RELN/SLC38A3/KCNJ1  1/IL1B/EPHB2/SLC34A1/GRM6/P  2RY6/LRRC55/ATP8A2/HAP1/TR  PV3/P2RX1/CCR2/CACNG4/CCL  4/ADCYAP1R1/SYT4/PIRT/CXCL  10/CYP4A11/CXCL11/CACNG3/  TRPC3/NOS1/XCL1/CXCL9/RYR2  /LRRC52/CRH/TRDN/GRIN1/CAL  CR/TNFSF11/DRD1/HTR3A/CAC  NG2/KCNC2/KCNA1/IFNG/NPY2R |
| --- | --- | --- | --- | --- |

| 0.497710826 13147 |  | tags=22%,  list=23%,  signal=17% |  | CDA/FCER1G/PAM/MLKL/H4C2/  KHDRBS3/AQP10/GJA1/MGST1/  RASEF/TMC8/TRPM4/PFKP/RRM2  /MAT1A/DPYSL3/ASIC1/H4C14  /KCND3/CD79B/TRIM22/CCK/P2  RX6/ANGPTL4/GCH1/SCUBE1/H  OMER1/TP73/H3C8/NLRC4/KCNF  1/HBE1/KCTD16/KCNV1/LCN2/R  IPK3/KCNV2/H4C1/PKD2L1/KCTD  4/KCNA2/OTC/PANX2/KCTD17/  TRPM2/ITLN1/CLDN1/SORL1/CD  79A/SLC6A1/ARC/ACPP/KCNC1  /GLRB/SLC1A1/TRPM3/JCHAIN/  AQP4/IKZF1/LY6G6C/CRYAA/BI  RC3/C9/SLC34A1/TDO2/PRF1/G  JA5/CALHM1/ADCY8/CLDN3/M  LC1/SYT1/KCNS2/P2RX1/FSD1/  ILDR1/PLN/CD3E/CD247/CNGB1  /CHRNA2/KCTD8/KCNA3/CD3D/  GBP1/RASGRP1/SLC1A2/KCNB2/  GRIN1/SLC22A6/PRMT8/TCL1A/  KCNG4/GBP5/AIM2/REG1A/KCN  A4/DPYS/CD3G/SEMG2/GLRA3/  MS4A1/KCND2/KCNC2/GLRA1/K  CNA1/CHRNB3/SEMG1/KCNA10 |
| --- | --- | --- | --- | --- |

| 0.497710826 12826 |  | tags=32%,  list=23%,  signal=25% |  | HRG/PROS1/SERPINA4/FETUB/SP  INK1/COL6A3/SERPINA12/ITIH1/  SYK/AGT/CR1/PZP/NGF/PI15/G  PC3/LGALS9/ANOS1/BST2/MT3  /SERPINA1/F2R/AHSG/MAPT/NL  RP3/SPOCK1/LEF1/CCK/CARD9/  HGF/TIMP4/COL4A3/PI16/BLID/  FN1/C3/CSTA/THBS1/SERPINC1  /SERPINA5/CSTL1/VSIR/KLF4/C  YFIP2/NGFR/NLRC4/USP50/MAP  K12/KHDC1L/AQP1/SERPINI2/SE  RPINE1/SERPINB8/CARD16/PI3/C  D109/BCL2L10/EPHA4/TNFSF10/  WNT9A/CST5/TNFRSF10A/ANX  A8/CASP1/TFPI2/SPOCK2/SOX2  /SORL1/VTN/SFN/TNFAIP8/PTG  S2/CASP10/CLEC7A/CLDN4/PS  MB9/MMP9/SERPINB13/FOXL2/E  CM1/CASP12/MEFV/SSPO/LCN1  /BIRC3/LAMP3/HMSD/ITIH6/CRB  2/CST7/PAX2/CLDN3/SMR3B/T  NFSF15/WFIKKN2/P2RX1/SPINK6  /SERPINB2/SERPINA3/CASP14/C  D27/TNFSF14/C4B/EPHA7/C4A/  TNF/LCK/TTBK1/NLRP7/MAGEA3  /DPEP1/SPINK5/GRIN1/SERPIND1  /WFDC5/GRIN2A/C3P1/WFDC12  /TFAP2B/AIM2/CSN2/CASP5/SP  INK4/SEMG2/ST18/FASLG/SPOC  K3/CST8/SERPINA9/CARD17/SE  RPINA7/SEMG1/CARD18/WFDC6  /SPINT4/SERPINB10/CST9/SMR3  A |
| --- | --- | --- | --- | --- |

| 0.497710826 12157 |  | tags=35%,  list=22%,  signal=28% |  | LSTN3/SLC1A3/SIPA1L1/NGF/A  SIC1/FABP5/ACHE/SYT12/ADGR  B1/HRH1/CLSTN2/F2R/TUBB2B/  MAPT/SNCG/NRGN/GRIK5/NTSR  1/FAM107A/UNC13A/ADORA1/  CAMK2A/PRKCB/SHANK1/KCNM  B4/LRP8/HOMER1/GUCY1A1/HRH  2/CA2/P2RY1/DRD2/BRSK1/GR  M2/CRHR2/DKK1/NPY5R/KMO/G  RIN2D/NPTX2/GRIN3A/NFATC4/  CACNG5/OXTR/EPHA4/NLGN4Y/  CHRNA3/ATP1A2/SYT11/LRRC4/  GRID1/MAP1B/SNCAIP/CDH2/GU  CY1B1/NLGN4X/PCDH17/PTGS2/  LILRB2/ROR2/SLC6A1/CELF4/LR  FN2/ARC/ITPKA/SLC7A11/RAB3  B/GRM3/KISS1/MAP1A/RELN/G  RM1/PLG/SHISA9/KCNJ10/IL1B/  GRIA1/SHISA8/CALB2/EPHB2/PR  RT2/GRM6/NTNG2/NTRK1/ADCY  8/LRRTM1/LRRC4C/HTR2A/ADC  YAP1/SYT1/CALB1/ADRA1A/HA  P1/P2RX1/HTR1B/NTF3/CCR2/C  ACNG4/SHISA6/PRKCG/CD38/GR  M4/ADCY1/SYT4/RASGRF1/NET  O1/CNTNAP4/CNTN2/SCTR/CHR  NA4/NRXN1/GRM8/GRIK1/CUX2  /SYT9/DRD5/TAC1/GRIA4/TNF  /SCGN/LRRTM2/CRH/CPLX4/SL  C24A2/CPLX2/DRD3/GRIN1/CNR  2/DGKB/TNR/GRID2/GRIN2A/CH  RNA6/NPTX1/GRIA3/DRD1/GRM7  /NEUROD2/RIMS2/PPFIA2/SLC8A |
| --- | --- | --- | --- | --- |

| 0.497710826 12648 |  | tags=23%,  list=22%,  signal=18% |  | SFRP2/CXCL12/THBD/HAMP/CD  KN1A/ELANE/MEIS2/CACNA1F/  ANXA1/ANGPT2/RCVRN/SLC1A3  /RPE65/MC1R/AANAT/HRH1/TS  PYL5/EYS/CCK/RHO/HMGA2/HR  H2/GNAT1/BCL3/DRD2/BRSK1/P  DE1B/PENK/RRH/GUCA1A/AQP1  /RGR/LRRN4/TNFRSF11A/NFATC  4/MAPK10/CARD16/SEMA5B/PD  E6C/ATP1A2/TULP1/RGS9BP/PPP  1R1B/AIPL1/CCL11/PTGS2/GUC  Y2D/VCAM1/GPR88/OPN1LW/N  DRG4/ELOVL4/SERPINB13/ADAM  2/SLC7A11/KCNC1/CACNA2D4/  HOXA1/GNGT2/SAG/PDE6A/GR  M6/NTRK1/IVL/ATP8A2/ABCA4  /PTPRC/PDC/GNGT1/OPN4/GAT  A3/NETO1/CNGA1/CRIP1/CXCL1  0/GRK1/CNGB1/GUCA1C/NR2E3  /TRPC3/CCR4/GRK7/SLC1A2/C  ABP4/FOXB1/DRD3/GRIN1/RP1/  GRIN2A/OPN5/ASIC2/IL12B/DRD  1/OPRM1/GJA10/SLC4A10/GUC  Y2F/GNAT2/KCNC2/OPN1MW |
| --- | --- | --- | --- | --- |

| 0.497710826 12198 |  | tags=40%,  list=22%,  signal=32% |  | 3CR1/RCAN2/PRKCA/PTGER3/A  DGRG1/CASQ2/ADGRG3/LAT/M  C1R/C10orf71/UCN3/ADGRB1/NP  R1/HRH1/CHGA/MAPT/CCL20/O  R51E2/EPHA5/P2RX2/CCR10/CC  R1/ADGRL2/SAMD14/CMKLR1/A  DCY4/PTGER1/PEX5L/RASD2/GU  CY1A1/AVPR1A/THBS1/HRC/DR  D2/DDAH1/ADGRB3/GUCY1A2/  AQP1/JPH1/EDN2/CGAS/APLP1  /GRIN2D/NOS1AP/ADRA2B/NFA  TC4/ADRA2A/RGN/GCGR/PTGFR  /PLEK/MRAP/APLNR/ADGRE3/C  ALML5/SPHK1/PTGIR/CCRL2/CA  SQ1/ATP1A2/LAT2/TRPM2/SELE  /GUCY1B1/ADRA1B/CRHR1/CCL  3/JSRP1/P2RX5/NPPC/VCAM1/  NRG1/MYOZ2/SLC8A1/TSHR/AD  GRG6/UCN2/ADM2/ADGRB2/ITP  R2/DEFB1/FPR1/FSHR/CLIC2/HP  CA/CCR9/GALR1/NFATC2/CALC  A/IGF1/PTGER4/PTHLH/CALML3  /GPR65/MAFA/NR5A2/TBC1D10  C/GPHA2/NMUR2/P2RY6/S1PR4/  ADCY8/ADCYAP1/PTPRC/CCR7/  ADRA1A/FPR2/HAP1/CCR2/CCL  4/ADCYAP1R1/CCR5/NEUROD1/  ADRB3/ADCY1/PLN/CD3E/CXCL  10/MC5R/SCTR/ADGRE1/ADGRL  3/CXCL11/ADCY2/HTR2B/CCR3  /DRD5/GBP1/NOS1/LGR5/TNF/  ADGRG4/CXCR3/ZAP70/BHLHA1  5/ADRB1/CXCL9/CCR4/CXCR6/ |
| --- | --- | --- | --- | --- |

| 0.497710826 14106 |  | tags=36%,  list=25%,  signal=28% |  | 4/CD74/BTK/TGFB3/TGFB1/TRIB  1/HMOX1/DTX1/NBL1/ANGPT1/  C1QC/MIR26B/IFI16/CLDN18/TA  L1/HLA-  G/FCER1G/LGALS3/RUNX3/LYN  /NRARP/H4C2/LGALS9B/FOXJ1/  CXCL12/TSC22D3/PELI1/JAK3/I  L1RL1/MAFB/MEIS2/TMEM176A/  INHBA/TAPBPL/TNFRSF21/GREM1  /FGR/ARG2/LOXL3/CR1/PLCL2/  IRAK3/ANXA1/LY96/HTRA1/H4C  14/THY1/LGALS9/BST2/VSIG4/  PDCD1LG2/CYP19A1/CD80/HLA  -  F/LILRB4/ADORA1/C5AR2/NLRC  3/LILRB3/PLA2G2D/SLIT2/MMP2  8/THBS1/TICAM2/LILRA4/VSIR/  DRD2/IL27RA/CCN3/MNDA/NPY  5R/TGFB2/IFNB1/MILR1/H4C1/T  NFSF4/INPP5D/PLA2G2E/LST1/H  AVCR2/RUNX1/SYT11/TNFAIP8L  2/CD86/ADTRP/CD84/CD300LF/  PTPN6/PGLYRP3/CCL3/PLA2G2A  /RNF125/FOXP3/SOX11/ALOX15  /DUSP10/GPR68/LILRB2/CD300A  /HLA-  DOA/SLAMF8/RAB7B/IL7R/APCS  /HES5/IL10/CD160/FOXF1/NLRC  5/FGL2/IFNL1/CLEC4G/LRFN5/P  TGER4/IL2RA/CCL21/TBC1D10C/  CCL25/LILRB1/SMPDL3B/IRF1/T  MEM178A/CST7/ADCYAP1/PTPR  C/CCR2/SAMSN1/CD200R1/HLA |
| --- | --- | --- | --- | --- |

| 0.497710826 14106 |  | tags=44%,  list=25%,  signal=33% |  | 274/CD74/NFKBIZ/C1QTNF1/AS  S1/TGFB1/CD4/CCM2L/DTX1/E  GR3/EPHB3/SH2B3/FERMT3/HLA  -  G/CCDC88B/ITGB2/LGALS3/RUN  X3/LYN/CDH1/TRPV4/NRARP/L  GALS9B/FOXJ1/CXCL12/RNASE1  0/PELI1/JAK3/ELANE/IGFBP2/TN  FRSF21/SYK/ARG2/LOXL3/JAG1  /SIRPB1/ANXA1/ITGA6/PLPP3/P  RKCA/AIF1/THY1/LGALS9/PTPR  U/B4GALNT2/VSIG4/TNFSF9/EFN  B2/CD1D/HLA-  DPA1/NLRP3/PDCD1LG2/PIK3R6/  LEF1/CD80/LILRB4/PLA2G2D/IGF  2/HLA-  DMB/ADAM8/IL6/IL1RN/VSIR/EP  O/WNT5A/KLF4/IL7/IFNB1/DPP4  /PTAFR/TNFSF4/FGB/PLA2G2E/  HAVCR2/RUNX1/BMP7/TNFAIP8L  2/CORO1A/CD86/IL1RL2/ADTRP  /PTPN6/CCL19/SOX2/PLA2G2A  /FOXP3/ICOSLG/MDGA1/NCKAP  1L/VAV1/VCAM1/ALOX15/SAS  H3/DUSP10/LILRB2/CCL5/CD300  A/IL7R/IL18/CD209/EBI3/HHLA2  /CD28/CD6/GLI2/IL10/CD160/P  LG/FGL2/IFNL1/CLEC4G/CD40LG  /IGF1/IL1B/TNFSF13B/IL2RA/NO  D2/CCL21/CCL25/LILRB1/IRF1/P  TPRC/CCR7/CLECL1/RASAL3/CC  R2/CARD11/IL15/MYB/CD5/GA  TA3/IL23R/CD3E/SHH/IL12RB1/ |
| --- | --- | --- | --- | --- |

| 0.497710826 14588 |  | tags=45%,  list=26%,  signal=34% |  | TMEM64/GAS6/TREM2/CASP8/T  YROBP/IL34/KITLG/TMEM176B/C  D74/BTK/NFKBIZ/TGFB1/TRIB1/  CD4/CSF1/DTX1/EGR3/C1QC/C  LDN18/TAL1/HLA-  G/RUNX3/IRF7/LYN/NRARP/FOX  J1/JAK3/MAFB/TMEM176A/INH  BA/SYK/LOXL3/CR1/ANXA1/PR  KCA/RASSF2/LGALS9/TNFSF9/N  LRP3/PIK3R6/LEF1/CCR1/CD80/  LILRB4/LILRB3/ADAM8/VSIR/CA  2/HCLS1/IL7/IFNB1/CD101/TNF  SF4/INPP5D/NFAM1/RUNX1/CD8  6/EVI2B/IL1RL2/PTPN6/PGLYRP3  /CCL3/CCL19/FOXP3/ZEB1/NCK  AP1L/SASH3/DUSP10/GPR68/LIL  RB2/ROR2/HLA-  DOA/SLAMF8/IL7R/IL18/CD28/P  RDM1/APCS/GLI2/FGL2/IFNL1/N  FATC2/OCSTAMP/IL2RA/TNFRSF  18/LILRB1/IRF1/TMEM178A/ZNF  683/POU4F1/PTPRC/IL27/CCR2/  CARD11/IL15/MYB/LIF/GATA3/I  L23R/DCSTAMP/CD2/SHH/IL12R  B1/CD27/TBX21/TNF/ZAP70/RA  SGRP1/TOX/SPINK5/IKZF3/LAG3  /TNFSF11/RHOH/TESPA1/IL12B/  PGLYRP2/GPR55/CARTPT/CTLA4  /FCRL3/IFNG/POU4F2/IL2 |
| --- | --- | --- | --- | --- |

| 0.497710826 12425 |  | tags=47%,  list=22%,  signal=37% |  | BP2/TNFRSF21/AHR/FGR/SYK/A  RG2/LOXL3/CR1/SIRPB1/ANXA1  /LAT/AIF1/THY1/LGALS9/VSIG  4/GPR183/TNFSF9/EFNB2/CD1D  /HLA-  DPA1/NLRP3/PDCD1LG2/PIK3R6/  RAC2/CD80/HLA-  F/LILRB4/PLA2G2D/IGF2/IGHV3-  73/HLA-  DMB/ADAM8/IL6/VSIR/EPO/TNF  SF18/IL27RA/MNDA/CD19/CGA  S/TNFRSF1B/IL7/IGLC2/IFNB1/I  GHA1/RIPK3/DPP4/TNFSF4/IGHM  /INPP5D/PLA2G2E/LST1/TNFSF8  /HAVCR2/IGHA2/NFAM1/LMO1/  IGHV4-59/RUNX1/IGHV5-  51/IGHG2/IGHV1-  45/TNFAIP8L2/CORO1A/CD86/I  GHG1/IL1RL2/IGHV4-  39/IGHE/PTPN6/PGLYRP3/CCL19  /IGHV3-  23/PLA2G2A/IGKC/FOXP3/ICOS  LG/SOX11/ZEB1/NCKAP1L/CLEC  7A/VAV1/VCAM1/SASH3/DUSP  10/LILRB2/CLC/IGHV3-  35/CCL5/CD300A/IGHV4-  31/HLA-DOA/IGHV4-  34/SLAMF8/IGLC7/IGHV3-  48/TNFRSF4/IL7R/IL13/IL18/CD  209/EBI3/HHLA2/CD28/PRDM1/I  GHV3-11/CD6/IGHV1-  69D/GLI2/IGHV3-  21/IL10/CD160/IGHV1- |
| --- | --- | --- | --- | --- |

| 0.497710826 10452 |  | tags=60%,  list=18%,  signal=49% |  | 73/C8G/FCER2/BCL3/IGLV3-  21/IL27RA/C1R/CD19/IGKV3-  20/IGLC2/IGLV2-  8/IGHA1/HLA-  DQB1/TNFSF4/IGHM/INPP5D/IGH  A2/C1S/IGHV4-59/IGHV5-  51/IGLV2-14/IGHG2/IGHV1-  45/IGHG1/IGHV4-  39/IGHE/PTPN6/IGHV3-  23/IGKC/FOXP3/IGLV2-  23/IGHV3-35/IGHV4-31/IGLV2-  11/IGHV4-34/IGKV3-  15/IGLC7/IGHV3-48/IGLV3-  19/IGKV1-5/CD28/IGHV3-  11/IGHV1-69D/APCS/IGHV3-  21/IL10/IGLV1-44/IGHV1-  3/IGHG3/IGHV4-61/IGHV1-  18/IGLC6/FGL2/C7/IGHV3-  30/IGHV2-70D/IGHV2-  5/CD40LG/POU2F2/IGLL5/IGLC3  /C9/IGHG4/TRDC/IGLV1-  40/IGHV1-  58/IGHD/BATF/TLR8/IGHV4-  28/IGKV1-17/IGHV1-24/IGHV3-  33/PTPRC/IGKV3D-11/IGHV2-  70/IGHV3-7/TRBC2/IGLV3-  25/IGLV7-43/IGHV3-20/IGLV3-  1/CD27/TBX21/IGHV3-  74/IGKV1-16/C4B/IGHV3-  13/C4A/IGHV4-  4/GCNT3/TNF/CRP/IGHV3-  66/XCL1/IGHV3-72/IGHV1-  69/CD70/SLA2/SUSD4/IGKV5- |
| --- | --- | --- | --- | --- |

| 0.497710826 14280 |  | tags=47%,  list=25%,  signal=35% |  | PLCG2/AKAP6/CALML6/ATP1B1  /CXCR4/CDH13/BTK/EGFR/CD4  /P2RX4/ANK2/ACKR4/BST1/CA  CNA1C/GRIN2C/TRPM4/SYK/NC  ALD/KDR/CX3CR1/RCAN2/CAS  Q2/LAT/C10orf71/MAPT/CCL20  /P2RX2/CCR10/CCR1/SAMD14/  CMKLR1/AVPR1A/HRC/JPH1/EDN  2/GRIN2D/NFATC4/RGN/PTGFR/  PLEK/CALML5/SPHK1/CCRL2/CA  SQ1/ATP1A2/LAT2/TRPM2/SELE  /CCL3/JSRP1/P2RX5/VCAM1/N  RG1/MYOZ2/SLC8A1/ADGRB2/D  EFB1/CLIC2/HPCA/CCR9/NFATC  2/IGF1/CALML3/NR5A2/TBC1D1  0C/NMUR2/P2RY6/PTPRC/CCR7/  FPR2/HAP1/CCR2/CCL4/CCR5/P  LN/CD3E/HTR2B/CCR3/GBP1/NO  S1/TNF/CXCR3/ZAP70/BHLHA15  /CCR4/CXCR6/RYR2/TRDN/SLA  2/GRIN1/SULT1A4/GRIN2A/TNFS  F11/KSR2/NEUROD2/CCR6/RIT2/  XCR1/GRM5/CXCR5/TRAT1/CCR  8 |
| --- | --- | --- | --- | --- |

| 0.497710826 13353 |  | tags=41%,  list=24%,  signal=31% |  | K4/SLC5A9/SLC2A10/SLC13A4/  KCNAB3/KCNH3/SLC4A11/ATP1B  2/SLC9A3/SLC4A5/SLC17A5/SL  C46A1/ATP5MGL/CACNA1H/HPN  /ATP2C2/SLC1A3/ASIC1/SLC24  A4/NDUFA4L2/ATP6V0A4/KCND  3/SLC15A3/SLC6A12/SCNN1G/  ATP1A3/KCNN4/KCNAB1/KCNJ1  /KCNU1/KCNT1/GRIK5/SLC2A6/  SLC4A4/KCNMA1/KCNE1/KCNE2  /KCNK6/SLC18A1/KCNMB4/SCN  4A/ATP6V1C2/SLC6A9/COX7A2  P2/KCNN3/HCN2/TRPM5/SLC6A1  3/COX7A1/AQP1/KCNF1/ATP6V  0D2/SLC18A2/SLC6A20/SLC45A  3/COX4I2/KCNQ3/SCN1B/KCNJ8  /KCNV1/LRRC38/KCNV2/PKD2L1  /SCN4B/SLC15A2/NOX5/SLC17  A7/SLC45A1/KCNA2/KCNMB1/A  TP1A2/ABCC9/SCN2B/SLC6A3/  TRPM2/MFSD2A/KCNK1/SCN9A/  ATP12A/KCNE1B/SCNN1B/SCNN1  A/KCNH7/SLC8A1/SLC5A4/SLC  6A1/SLC34A2/ABCC8/KCNC1/K  CNK10/SLC9C2/KCNH2/SLC6A2/  SLC1A1/KCNK9/HCN4/KCNJ10/K  CNJ11/SLC5A12/SLC9A7/SLC34  A1/KCNK18/SLC36A2/KCNK13/K  CNJ6/KCNT2/SLC28A3/LRRC55/  SCN7A/SLC12A3/KCNS2/OTOP2  /SLC9A2/SLC13A5/SLC4A8/KC  NQ2/KCNK3/GRIK1/SLC5A7/SLC  5A8/KCNJ3/KCNA3/KCNJ4/ABC |
| --- | --- | --- | --- | --- |

| 0.497710826 14846 |  | tags=33%,  list=26%,  signal=25% |  | CEBPA/ANO6/CCN1/SIX2/DNAJ  C13/TMEM64/CITED1/SNX10/EG  R2/IFITM5/ID1/ID3/ZHX3/TGFB3  /DHH/EGFR/TGFB1/SH3PXD2B/S  EMA7A/CSF1/CDH11/ITGA11/T  CIRG1/CEBPD/COMP/SNAI1/KL/  TWIST2/S1PR1/HEMGN/SFRP2/C  OL1A1/ASGR2/WWTR1/ISG15/T  RPM4/STC1/GREM1/FGR/JAG1/  MMP2/RASSF2/FOXC1/GPC3/IFI  TM1/ATP6V0A4/DLX5/ACHE/DH  RS3/ASPN/AHSG/LEF1/CCR1/H  GF/NOCT/CREB3L1/SCX/CHRD/I  GF2/GLI1/IL6/TNC/WNT5A/NOG  /PENK/DKK1/TGFB2/TNFRSF11A  /FGF18/FGFR2/VCAN/FOXC2/R  ORB/PHEX/BMP7/MN1/RUNX2/  WNT10B/GDF5/CCL3/SOX2/GDP  D2/TMEM119/SOX11/MRC2/PTG  S2/CASR/NPPC/ALOX15/SLC8A  1/ROR2/CHRDL2/TWIST1/BMPR1  B/CYP24A1/ECM1/GLI2/PHOSPH  O1/NPNT/CALCA/IGF1/PTGER4/  PTHLH/SLC34A1/SOST/ADGRV1  /MYOC/CLEC3A/AREG/DSPP/FG  F23/RSPO2/SHH/TAC1/MMP16/  TNF/OSR2/GDF10/NELL1/TNFSF1  1/MMP13/DMP1/STATH/IBSP/A  MELX/MYF5/MYOG/MIR138-  2/MIR208A |
| --- | --- | --- | --- | --- |

| 0.497710826 12453 |  | tags=44%,  list=22%,  signal=35% |  | RNASE10/JAK3/ELANE/IGFBP2/S  YK/SIRPB1/ANXA1/ITGA6/PLPP3  /AIF1/THY1/LGALS9/PTPRU/TN  FSF9/EFNB2/CD1D/HLA-  DPA1/NLRP3/PDCD1LG2/PIK3R6/  CD80/LILRB4/IGF2/HLA-  DMB/ADAM8/IL6/VSIR/EPO/WN  T5A/IL7/DPP4/PTAFR/TNFSF4/F  GB/HAVCR2/RUNX1/BMP7/CORO  1A/CD86/IL1RL2/PTPN6/CCL19/  SOX2/FOXP3/ICOSLG/NCKAP1L/  VAV1/VCAM1/ALOX15/SASH3/  DUSP10/LILRB2/CCL5/IL7R/IL18  /CD209/EBI3/HHLA2/CD28/CD6  /GLI2/IL10/CD160/CD40LG/IGF1  /IL1B/TNFSF13B/IL2RA/NOD2/C  CL21/LILRB1/PTPRC/CCR7/CLEC  L1/RASAL3/CCR2/CARD11/IL15  /MYB/CD5/GATA3/IL23R/CD3E  /SHH/IL12RB1/ADAM19/CD27/S  KAP1/GRAP2/TNFSF14/TNF/ITG  A4/LCK/ZAP70/XCL1/RASGRP1  /CD70/TNFSF11/RHOH/TESPA1/  IL12B/BTLA/KLRC4-  KLRK1/PDCD1/SIRPG/CXCL13/KL  RK1/ICOS/CTLA4/IL21/FGA/IFN  G/IL2 |
| --- | --- | --- | --- | --- |

| 0.497710826 15532 |  | tags=40%,  list=27%,  signal=29% |  | D3/SOD2/RILP/SLC12A8/RDX/N  KX6-  1/CDH4/APLN/ATP2B1/GPER1/S  LC12A9/BDNF/LIMA1/F2RL1/TTL  /NOS3/SEMA3D/SPTA1/ADRA1D  /SEMA4D/DEPTOR/KANK4/SPTB  N1/ICAM1/KIRREL1/BAIAP2L2/M  ACF1/RAP1GDS1/DRAXIN/ESAM  /ANO6/ACTA2/CIT/CTNNA2/V  AV2/ARFGEF1/LRP1/TRIM46/SER  PINF2/PLXNA3/ADORA2B/FGF13  /CAPG/FGG/CDC42EP2/XK/KCN  MB2/EGFR/SEMA7A/EDNRA/HM  OX1/SEMA5A/RND2/PIK3C2A/S  EMA3A/COMP/COTL1/PREX1/L  MOD2/TRPV4/RET/FMN1/MAP2/  GJA1/CXCL12/SEMA3G/TRPM4/  ECE1/CDHR2/OLFM1/AGT/ARHG  AP18/NRP1/NGF/FOXC1/ISLR2/  TNFRSF12A/MT3/NPR1/TRPV2/H  RH1/CHGA/F2R/ADD2/KCNN4/M  APT/SEMA3F/ITGA1/HTR7/KCN  MA1/GMFG/PLEKHG2/ADORA1/E  LN/SLIT2/GCH1/FN1/KCNMB4/P  2RY2/LMOD1/CRABP2/GUCY1A1  /AVPR1A/HRH2/IQGAP2/P2RY1/  HCLS1/WNT5A/GNB3/SEMA6B/  AQP1/WAS/EDN2/WNT9B/CDHR  5/ADRA2B/ADRA2A/HCK/SPTBN  5/FOXC2/PTAFR/PLEK/FGB/CAR  MIL2/OXTR/ACE/BDKRB2/KCNM  B1/ATP1A2/MAP1B/CORO1A/L  MOD3/ADRA1B/SPTB/CCL11/PT |
| --- | --- | --- | --- | --- |

| 0.497710826 12209 |  | tags=38%,  list=22%,  signal=30% |  | 1A2/CLIC6/CACNA1F/CACNB4/  CASQ2/THY1/CEMIP/KCND3/CR  HBP/CACNA2D1/DLG2/F2R/KCN  AB1/KCNJ1/KCNU1/MIR26A2/NT  SR1/CRACR2A/GLRX/LRRC7/KC  NMA1/KCNE1/KCNE2/RGS9/CYB  B/CACNG1/SHANK1/KCNK6/SCN  4A/HOMER1/THBS1/HRC/DYSF/  HCN2/TRPM5/EPO/FGF12/DRD2/  TMEM37/BDKRB1/KCNF1/CD19/J  PH1/CACNB1/REM1/NOS1AP/F2  RL3/ADRA2A/KCNQ3/SCN1B/RG  N/PM20D1/TCAF2/KCNJ8/KCNV  1/LRRC38/KCNV2/PTAFR/CLCNK  B/STRIT1/CACNG5/SCN4B/APLN  R/NOX5/MIR212/CLCNKA/CASQ  1/KCNA2/KCNMB1/ATP1A2/COR  O1A/SCN2B/FXYD7/PTPN6/CRHR  1/SCN9A/JSRP1/STAC/KCNE1B  /RRAD/GSG1L/KCNH7/CACNA1E  /SLC8A1/MMP9/TWIST1/CACN  A2D3/GEM/TRPC6/ARC/IL13/C  AV3/KCNC1/KCNK10/CLIC3/HEC  W2/CACNA2D4/KCNH2/CLIC5/R  ELN/CLIC2/HPCA/SHISA9/HCN4  /KCNJ10/KCNJ11/GRIA1/SHISA8  /EPHB2/SLC34A1/PIK3CG/HECW  1/KCNK13/KCNJ6/P2RY6/GJA5/  NEFL/CALHM1/LRRC55/SCN7A/  ANO9/KCNS2/HAP1/CCR2/CACN  G4/SHISA6/KCNQ2/PLN/RASGRF  1/NETO1/PIRT/CXCL10/CXCL11  /KCNJ3/KCNA3/CACNG3/KCNJ4 |
| --- | --- | --- | --- | --- |

| 0.497710826 12401 |  | tags=31%,  list=22%,  signal=24% |  | /FGR/SYK/ARG2/ANXA1/CSF1R  /CRLF2/PRKCA/AIF1/LGALS9/A  CHE/UCN3/CRHBP/ANO1/F2R/K  CNN4/OR51E2/EPHA5/FRMD4A/  NLRP3/DOC2B/OSM/ORM1/ADO  RA1/C5AR2/FN1/TFR2/IL1A/RP  H3AL/RSAD2/ADAM8/IL6/FFAR4  /CD33/SLC30A8/WNT5A/DRD2/  IL27RA/CCN3/USP50/CD244/LIL  RA5/INHBB/TGFB2/TNFRSF1B/HC  AR2/ADRA2A/CLEC4E/DPP4/TNF  SF4/CARD16/FGB/MIR30C1/HAV  CR2/SRCIN1/SYT11/TRH/PANX2  /ADTRP/CASP1/CCL3/CCL19/S  ORL1/FOXP3/CASR/MIR766/CHI  A/CACNA1E/FFAR2/GPR68/VSN  L1/CCL5/TWIST1/ABCC8/TNFRS  F4/IL13/KISS1/IL10/CD160/ITPR  2/CD40LG/IGF1/PTGER4/KCNJ11  /GRP/IL1B/CADM1/G6PC2/NOD2  /LILRB1/GJA5/IGHD/ADCY8/TLR  8/ADCYAP1/CCR7/IL1R2/PAEP/I  LDR1/GATA3/NEUROD1/CD38/C  D200R1/SYT4/IL26/CD2/HTR2B/  SYT9/HNF4A/BANK1/FFAR3/GBP  1/TNF/CRP/NLRP7/ISL1/RASGRP  1/PTPN22/ORM2/APOA2/CRH/D  RD3/SLC2A2/CLEC9A/TNFSF11/  TFAP2B/MMP12/TLR10/AIM2/A  GTR2/OPRM1/CRTAM/HMGB4/C  ARTPT/CCL1/KCNC2/MMP8/FGA  /IFNG/CARD17/NPY2R/SSTR5/G  HSR/IL17F/HTR2C/CARD18/NLRP |
| --- | --- | --- | --- | --- |

| 0.497710826 11549 |  | tags=36%,  list=20%,  signal=29% |  | CRHBP/CACNA2D1/DLG2/KCNAB  1/MIR26A2/NTSR1/CRACR2A/GL  RX/SGK1/LRRC7/KCNE1/KCNE2/  RGS9/SHANK1/HOMER1/HRC/DY  SF/EPO/FGF12/DRD2/JPH1/CAC  NB1/REM1/NOS1AP/ADRA2A/SC  N1B/RGN/PM20D1/TCAF2/LRRC3  8/PTAFR/STRIT1/CACNG5/SCN4  B/MIR30C1/MIR212/CASQ1/ATP  1A2/SCN2B/FXYD7/CRHR1/JSRP  1/PON1/STAC/KCNE1B/RRAD/G  SG1L/MMP9/TWIST1/GEM/TRPC  6/ARC/CAV3/KCNC1/HECW2/R  ELN/CLIC2/HPCA/SHISA9/KCNJ1  1/GRIA1/SHISA8/EPHB2/HECW1  /P2RY6/NEFL/LRRC55/ANO9/KC  NS2/HAP1/CCR2/CACNG4/SHIS  A6/PLN/RASGRF1/NETO1/PIRT/  CACNG3/NOS1/GRIA4/FXYD2/R  YR2/MRLN/LRRC52/APOA2/CRH  /CACNG7/TRDN/DRD3/GRIN1/G  RIN2A/GRIA3/OPRM1/GRM5/GRI  A2/HTR3A/CACNG2/KCNC2/KC  NA1/IFNG/OR56A5/MIR153-1 |
| --- | --- | --- | --- | --- |

| 0.497710826 11598 |  | tags=32%,  list=21%,  signal=26% |  | SYT12/POTEKP/EFNB2/GRIK5/DO  C2B/UNC13A/PRKCB/SLC18A1/S  H3GL2/P2RY1/DRD2/BRSK1/MX2  /RAB27B/DNM3/SLC18A2/GRIN3  A/MAPK10/OTOF/NLGN4Y/SLC1  7A7/SYNDIG1/SYT11/CDH2/PAC  SIN1/NLGN4X/PCDH17/GSG1L/S  TON2/TRIM9/ARC/RAB3B/AMPH  /HPCA/CADPS/PRRT2/ITGB3/HT  R2A/SYT1/ADRA1A/P2RX1/HTR  1B/SYT5/CALY/PRKCG/ADCY1/  SYT4/CHRNA4/SYT6/NRXN1/SN  AP91/SYT9/SYT2/DDC/CPLX4/  CPLX2/DRD3/CHRNA6/DRD1/RIM  S2/SLC32A1/PPFIA2/RIMS1/UNC  13C/SLC17A8/CHRM2/CHRNB3/  SLC17A6/CPLX3 |
| --- | --- | --- | --- | --- |

| 0.497710826 14640 |  | tags=27%,  list=26%,  signal=20% |  | KDELR3/LRP1/FZD5/CTSC/HLA-  DPB1/CD9/HIP1/CD74/EGFR/CD4  /HLA-  DRA/WIPI1/BTBD8/PIK3C2A/HLA  -G/HLA-  H/FZD2/AAK1/ABCB4/HLA-  DRB1/ECE1/UNC13D/NCALD/CE  MIP/SERPINA1/ASTN2/NRGN/HL  A-DPA1/HLA-DRB5/HLA-  F/SLC18A1/SH3GL2/TICAM2/TG  FA/SEC24D/WNT5A/HLA-  DQA2/RAB27B/SLC18A2/LMAN1  L/HLA-  DQB1/SLC17A7/PACSIN1/HLA-  DQA1/HLA-  DQB2/RASSF9/STON2/LRP2/ROR  2/ASTN1/IL7R/AP1M2/KIAA031  9/FOLR1/GRIA1/CLVS1/FCGR1A  /ADCY8/SYT1/CLVS2/AREG/F5  /FCGR1B/EGF/SGIP1/CLRN1/SN  AP91/SYT9/SYT2/CD3D/MALL/  CPNE6/GAD1/SLC32A1/CD3G/C  TLA4/CHRM2 |
| --- | --- | --- | --- | --- |

| 0.497710826 13511 |  | tags=33%,  list=24%,  signal=25% |  | P2RX4/SYP/ADCY10/CDHR3/TEN  M2/PREX1/TRPV4/FLRT3/AAK1/  MAP2/PCDH9/OLFM1/BASP1/DP  YSL3/NRP1/THY1/ELAVL4/UCN3  /CRHBP/TRPV2/MAPT/GRIK5/NT  SR1/CCK/KIF21B/UNC13A/MAP3  K12/ADORA1/PRKCB/SLC18A1/  COBL/DRD2/BRSK1/CDK5R2/NGF  R/FEZ1/CRHR2/PENK/SLC18A2/  EPHA4/KCNA2/SYT11/MAP1B/T  ULP1/PACSIN1/CASR/GPM6A/KI  F5C/KCNC1/PDYN/INPP5J/OTX2  /CHRM3/UNC5C/CALCA/ITGA2/  PTPRO/PRRT2/USH2A/NEFL/LRRT  M1/LAMP5/ROR1/PTPRN/ADCYA  P1/TUBB3/CALB1/HTR1B/STMN2  /PRKCG/RASGRF1/DSCAM/CNGB  1/OPRD1/STMN4/KCNA3/NOS1/  ITGA4/CABP4/CPLX4/CPLX2/GR  IN1/ZNF804A/REG1A/GAD1/NRS  N1/SLC32A1/PNOC/SLC4A10/U  NC13C/KCNC2/SLC17A8/GLRA1  /KCNA1/CHRM2/CPLX3 |
| --- | --- | --- | --- | --- |

| 0.497710826 14787 |  | tags=45%,  list=26%,  signal=33% |  | 1/MMP7/COL17A1/SPINT2/TNFR  SF1A/THSD4/LRP1/GAS6/COL8A  2/SCUBE3/PTX3/SERPINF2/HAPL  N1/KLK7/HAS3/ELF3/LAMB1/FG  G/NID1/FBLN2/COL8A1/TGFB1/  SH3PXD2B/ICAM2/CTSS/LUM/C  OL15A1/ADAM12/ITGA11/COMP  /CRISPLD2/KLKB1/COLQ/NDNF/I  TGB2/COL16A1/CDH1/MFAP4/L  AMA3/MPZL3/TTR/SFRP2/COL1  A1/NID2/ICAM5/ELANE/COL6A3  /PECAM1/MMP11/GREM1/LOXL3  /AGT/MMP2/LOXL2/WT1/KDR/  HPN/HTRA1/ITGA6/COL4A2/SUL  F1/FOXC1/ECM2/COL10A1/COL  4A1/ITGA1/HAS1/MMP1/COL4A  3/TGFBI/LOXL4/KLK5/ITGA7/EL  N/COL9A2/CREB3L1/SCX/FN1/  MMP28/SCUBE1/COL5A1/ADAM  8/IL6/THBS1/BCL3/TNC/TLL2/S  ULF2/ITGA5/ITGA10/ADAMTS14  /TGFB2/TNFRSF1B/LCP1/SMOC2  /SERPINE1/VCAN/DPP4/FOXC2/  FGB/CARMIL2/ITGAX/MYH11/C  TRB1/COL5A3/ADAMTS4/COL14  A1/ADTRP/COL9A3/SPOCK2/AD  AMTS5/VTN/PXDN/COL12A1/C  MA1/ADAMTS9/LAMB4/VCAM1  /MMP9/NCAN/ITGB8/ADAMTS2  /EGFL6/TLL1/PLG/FOXF1/NPNT  /EGFLAM/CTSG/TPSAB1/KLK4/  LAMA1/ITGA2/ITGB3/TNFRSF11B  /MMP20/ACAN/ITGAL/COL22A1 |
| --- | --- | --- | --- | --- |

| 0.497710826 13511 |  | tags=38%,  list=24%,  signal=29% |  | P2RX4/SEMA3A/ATP2A3/ANK2/  TNNI3K/CACNB2/TNNI2/CACNA1  C/ATP1B2/NKX2-  5/GJA1/WWTR1/TRPM4/STC1/  CACNA1H/AGT/CACNA1F/CACN  B4/CASQ2/KCND3/NPR1/ATP1A  3/CACNA2D1/CHGA/MIR26A2/  MYBPC3/KCNE1/KCNE2/ADORA1  /GJC1/CACNG1/TMIGD3/GCH1/  KCNK6/TNNC1/AVPR1A/HRC/MY  LK2/FOXN4/FGF12/DRD2/DES/C  ACNB1/EDN2/TGFB2/NOS1AP/C  ORIN/SCN1B/KCNJ8/CACNG5/S  CN4B/ACE/CASQ1/THRB/ATP1A  2/ABCC9/SCN2B/FXYD7/ADRA1  B/KCNK1/KCNE1B/NPPC/SLC8A1  /CELF2/CACNA2D3/CAV3/CAC  NA2D4/KCNH2/SLC1A1/ITPR2/C  LIC2/CALCA/HCN4/KCNJ11/TNN  T2/PIK3CG/GJA5/ADRA1A/CAC  NG4/PLN/ATP2B2/KCNK3/MYL4  /KCNJ3/CACNG3/KCNJ4/TAC1/  NOS1/FXYD2/ADRB1/SGCG/RYR  2/CACNA1S/CACNG6/CACNG7/  TRDN/CACNA1G/ACTC1/SCN1A  /AGTR2/TACR3/SLC8A3/KCND2  /CACNG2/CHRM2/CSRP3/MC3R  /IL2/MIR208A |
| --- | --- | --- | --- | --- |

| 0.497710826 13719 |  | tags=33%,  list=24%,  signal=25% |  | EGFR/TRIB1/FGFR1/HMOX1/ANG  PT1/SIX1/CALCRL/S1PR1/STAT  1/NKX2-  5/GJA1/ABCC4/CDKN1A/NOTCH  3/PDGFRB/ELANE/AGT/MMP2/F  OXC1/AIF1/NPR1/MYOCD/TBX5  /MIR26A2/TBX20/PDE1A/ELN/G  LI1/IL6/THBS1/TP73/KLF4/NOG  /CCN3/WNT2/NPY5R/TGFB2/FG  FR2/FOXC2/PTAFR/PTGIR/MIR27  B/PTGS2/NPPC/CCL5/NDRG4/M  MP9/MSTN/IL13/IL18/HES5/IL10  /NPR3/MIR199A1/IGF1/ITGA2/Z  FPM2/TENM4/P2RY6/GATA6/MI  R185/HTR1B/MIR503/IL15/MYB/  SHH/TNF/IL12B/IFNG/MYOG/MI  R204/MIR199B/MIR200B/MIR208  A/MIR182 |
| --- | --- | --- | --- | --- |

| 0.497710826 13835 |  | tags=30%,  list=24%,  signal=23% |  | XK/ASS1/TAGLN/TGFB1/FGFR1  /METTL21C/CCM2L/EGR3/PITX1  /HIVEP3/ITGA11/SIX1/FGF8/S1P  R1/FZD2/HAND1/NKX2-  5/GJA1/KLHL41/HEYL/HAMP/KL  HL40/COL6A3/NEURL1/CACNA1H  /GREM1/BASP1/WT1/FOXC1/M  SX1/ACHE/ADGRB1/MYOCD/TB  X5/HEG1/KCNAB1/EFNB2/P2RX2  /LEF1/MYBPC3/FZD7/SAP30/TB  X20/ITGA7/ELN/PI16/SCX/TNN  C1/GLI1/HOMER1/H1-  5/TP73/MYLK2/WNT5A/TLL2/N  OG/PAX7/MAPK12/DKK1/JPH1/  WNT2/TGFB2/ASB2/VGLL2/MYO  D1/FGFR2/FOXC2/STRA6/MYF6  /CASQ1/WNT10B/LMOD3/ACTA  1/FGF3/SOX11/NRG1/MYOZ2/L  RP2/TWIST1/MSTN/FOXL2/DLL4  /CAV3/MYH15/MIR199A1/IGF1/  CNTF/TNNT2/ZFPM2/PHOX2B/EO  MES/UNC45B/ANKRD2/MKX/SIX  4/POU4F1/SMTNL1/GATA6/LIF/  PAX5/CXCL10/SHH/MYMX/NPH  S1/SGCG/RYR2/VAX1/ISL1/MY  MK/ACTC1/MYOM3/TIFAB/ANK  RD33/NEUROG1/CSRP3/MYF5/D  CANP1/MYOG/MIR204/MIR199B/  MIR200B/MIR208A |
| --- | --- | --- | --- | --- |

| 0.497710826 12753 |  | tags=31%,  list=23%,  signal=24% |  | CTR3/PROS1/SERPINA4/SFI1/FET  UB/SPINK1/COL6A3/SERPINA12/  ITIH1/AGT/CR1/ANXA1/PZP/NG  F/PI15/GPC3/PPP1R1C/ANOS1/B  ST2/MT3/SERPINA1/DLG2/AHSG  /PLA2R1/SPOCK1/LEF1/HGF/TI  MP4/COL4A3/ANGPTL4/PI16/SLI  T2/RIMBP2/C3/CSTA/THBS1/GN  AT1/SERPINC1/SERPINA5/IQGAP  2/CSTL1/KLF4/NGFR/AQP1/TGF  B2/SERPINI2/RGN/SERPINE1/SERP  INB8/SH3RF2/CARD16/PI3/CD10  9/WNT9A/CST5/TMEM225B/AN  XA8/TFPI2/GRXCR1/SPOCK2/SO  RL1/PPP1R1B/LEPR/VTN/SFN/TN  FAIP8/PTGS2/NCKAP1L/MMP9/S  ERPINB13/SPOCD1/PPP1R1A/ECM  1/APCS/SSPO/PPP1R14C/LCN1/  BIRC3/TNNT2/SAG/LAMP3/HMS  D/ITIH6/CRB2/GZMA/CST7/PAX  2/SMR3B/ADCYAP1/BOD1L2/WF  IKKN2/PPP1R2C/SPINK6/SERPINB2  /PLN/PPP1R2P1/SERPINA3/CD27  /TNFSF14/C4B/C4A/NOS1/TNF  /NLRP7/PCDH11X/APOA2/MAGE  A3/DPEP1/SPINK5/SERPIND1/WF  DC5/C3P1/WFDC12/RHOH/TFAP  2B/SLC7A14/KLRC4-  KLRK1/CSN2/SPINK4/SPOCK3/C  ST8/KLRK1/TMEM132D/SERPINA9  /PPP1R17/CARD17/SERPINA7/CA  RD18/WFDC6/SPINT4/SERPINB10  /CST9/SMR3A/MIR182 |
| --- | --- | --- | --- | --- |

| 0.497710826 13754 |  | tags=46%,  list=24%,  signal=35% |  | SEMA3D/SPTA1/SEMA4D/TRIO/  DOK1/SPTBN1/VLDLR/OR10A4/S  H3KBP1/ALCAM/DRAXIN/GPC1/  LRP1/EGR2/PLXNA3/UNC5B/PLX  NA2/CXCR4/ROBO4/NECTIN1/SE  MA7A/SEMA5A/EPHB3/SEMA3A  /FGF8/POU4F3/RET/FLRT3/RAP1  GAP/EFNA3/CNTN6/ARTN/CXCL  12/SEMA3G/VSTM2L/NRP1/CSF  1R/PRKCA/DLX5/ANOS1/NKX2-  1/TUBB2B/SEMA3F/DOK5/EFNB2  /EPHA5/RAC2/PAX6/ZSWIM5/S  LIT2/DOK6/WNT5A/EPHA1/NOG  /EPHA3/CYFIP2/FEZ1/NFASC/G  BX1/NCAM1/SHC3/SEMA6B/SC  N1B/SPTBN5/FOXD1/SEMA5B/EP  HA4/DAB1/BMP7/DOK2/ARX/SL  IT3/SPTB/CHN1/EPHA6/NEXN/G  FRA1/TMEFF2/BMPR1B/EPHA8/KI  F5C/PLXNA4/GFRA2/GLI2/LHX9  /EMB/SEMA3E/RELN/OTX2/UNC  5A/ATOH1/UNC5C/EGFLAM/NT  N1/LAMA1/EPHB2/PTPRO/NTRK1  /GBX2/DSCAML1/TUBB3/IGSF9/  FOXG1/NRXN3/GATA3/DPYSL5/  FEZF1/LRTM1/CNTN2/DSCAM/S  LIT1/SHH/NRXN1/KIF5A/RNF165  /EPHA7/EPHA10/VAX1/ISL1/ISL  2/FEZF2/DRGX/LHX3/TNR/MYP  N/BCL11B/UNC5D/LHX1/LRTM2/  TBR1/POU4F2 |
| --- | --- | --- | --- | --- |

| 0.497710826 12166 |  | tags=36%,  list=22%,  signal=29% |  | SYK/CHRNA5/SV2A/SLC1A3/AS  IC1/SYT12/NAAA/SLC6A12/SN  CG/GRIK5/NTSR1/DOC2B/UNC13  A/CAMK2A/PRKCB/SLC18A1/KC  NMB4/SLC6A9/GABRQ/SLC6A13  /P2RY1/DRD2/BRSK1/AQP1/SLC  18A2/KMO/SLC6A20/GRIN3A/M  AOB/OTOF/SLC17A7/CHRNA3/A  TP1A2/SYT11/TRH/SNCAIP/NRX  N2/SLC6A3/SLC38A5/CHAT/TRI  M9/SLC6A1/SLC7A11/RAB3B/SL  C6A2/SLC1A1/KCNJ10/CADPS/  PRRT2/SLC1A7/SLC36A2/ITGB3/  LILRB1/HTR2A/ADCYAP1/SYT1/  ADRA1A/P2RX1/HTR1B/SYT5/PR  KCG/GRM4/ADCY1/SYT4/GABR  A2/CHRNA4/SYT6/NRXN1/SLC5  A7/SYT9/SYT2/NOS1/SLC1A2/  DDC/CRH/CPLX4/CSF2/CPLX2/  DRD3/SLC6A18/CHRNA6/SLC22A  2/TNFSF11/DRD1/GAD1/RIMS2/  SLC32A1/PPFIA2/RIMS1/SV2C/U  NC13C/SLC17A8/CHRM2/CHRNB  3/SLC17A6/HTR2C/CPLX3 |
| --- | --- | --- | --- | --- |

| 0.497710826 7553 |  | tags=50%,  list=13%,  signal=43% |  | /TRAV38-1/CD6/TRBJ2-  1/GRM1/SHISA9/NPNT/IFNL1/G  ABBR2/GRIA1/SHISA8/ITGA2/IL  2RA/TRBV11-  2/TRDC/TRAC/ITGB3/IL2RB/TRA  V17/TRAV25/ITGAL/TRBJ2-  2/CACNG4/TRAV9-  2/SHISA6/TRAV27/IL23R/TRBC2  /CD3E/TRAV41/CHRNA4/CD247  /IL12RB1/TRBV28/GRIK1/TRAV1  3-  2/TRAV6/CHRNA9/SKAP1/CHRN  A2/TRAV40/TRBV29-  1/CD3D/TRAV16/CACNG3/TRAV  5/TRBV10-3/TRAV8-  4/GRIA4/TRBV30/ITGA4/ZAP70  /TRBJ2-3/TRAV1-2/TRBV5-  4/TRBV24-1/TRBV5-6/TRBJ2-  7/CD8A/TRBV5-1/TRBV20-  1/TRBV2/CACNG7/TRAV20/TRA  V18/TRAV35/TRBV7-  9/GRIN1/TRBV4-1/TRBV3-  1/TRAV39/TRGV9/GRID2/TRAV2  9DV5/GRIN2A/TRGV3/TRBV7-  7/TRBV19/CALCR/TRAV12-  3/TRAV2/CHRNA6/TRAV36DV7/  TRBV7-4/TRAV13-1/TRAV12-  1/GRIA3/TRAV8-  6/TRAV14DV4/TRBV5-  5/TRAV21/CD8B/TRBV18/TRAV1  0/TRAV34/OLFM3/TRBV6-  5/TRAV22/TRAV8-1/TRBV10-  1/TRGC2/CD3G/TRDV1/TRAV8- |
| --- | --- | --- | --- | --- |

| 0.497710826 14186 |  | tags=45%,  list=25%,  signal=34% |  | /EMP2/CD274/CDH13/NID1/CD7  4/NFKBIZ/COL26A1/FBLN2/COL8  A1/CD4/CSF1/EGR3/ANGPT1/H  LA-  G/CCDC88B/NDNF/ITGB2/COL16  A1/RUNX3/LYN/PREX1/RET/AG  R2/FMN1/SFRP2/HRG/RELL2/CX  CL12/RNASE10/JAK3/ELANE/IGF  BP2/SYK/UNC13D/SIRPB1/ANXA  1/NRP1/KDR/ITGA6/PLPP3/PRKC  A/ADGRG1/ECM2/AIF1/VWC2/  THY1/LGALS9/PTPRU/TNFSF9/EF  NB2/CD1D/HLA-  DPA1/NLRP3/PDCD1LG2/PIK3R6/  CD80/LILRB4/CHRD/FN1/IGF2/H  LA-  DMB/ADAM8/IL6/DYSF/VSIR/EP  O/WNT5A/EPHA1/ITGA5/TNFSF  18/TGFB2/IL7/SMOC2/DPP4/FO  XC2/PTAFR/TNFSF4/FGB/HAVCR  2/RUNX1/BMP7/CORO1A/CD86/  IL1RL2/MIR27B/PTPN6/SPOCK2/  CCL19/SOX2/FOXP3/ICOSLG/NC  KAP1L/VAV1/VCAM1/ALOX15/  SASH3/DUSP10/LILRB2/CCL5/AP  BB1IP/IL7R/IL18/CD209/EBI3/HH  LA2/CD28/CD6/GLI2/EGFL6/IL1  0/CD160/FOXF1/NPNT/EDIL3/EG  FLAM/CD40LG/IGF1/IL1B/ITGA2  /TNFSF13B/IL2RA/TNFRSF18/NO  D2/CCL21/CCL25/LILRB1/CASS4  /PTPRC/CCR7/CLECL1/RASAL3/  MYOC/CCR2/CARD11/IL15/MYB |
| --- | --- | --- | --- | --- |

| 0.497710826 14601 |  | tags=43%,  list=26%,  signal=32% |  | PYDC1/CASP8/HLA-  DPB1/TYROBP/FLT4/SERPINF2/C  1QTNF4/IL1F10/ADORA2B/PLCG2  /MIR149/ZP3/MCOLN2/SERPINB7  /STING1/FAM49B/CD274/CD34/  CD74/PANX1/TGFB1/SEMA7A/C  D4/HMOX1/CCM2L/LUM/IFI16/H  LA-  G/CCDC88B/FCER1G/IRF7/TRPV4  /STAT1/TXK/LGALS9B/SPON2/  PELI1/ELANE/IL1RL1/FGR/SYK/  AGT/ANXA1/DDX60/LY96/CSF1  R/CRLF2/SULF1/C3AR1/AIF1/LG  ALS9/F2R/HEG1/HLA-  DPA1/NLRP3/IL17B/CARD9/HGF  /OSM/CD80/HLA-  F/ORM1/CEACAM20/CYBB/IL1A  /RSAD2/C3/ADAM8/IL6/THBS1  /IRF5/IL1RN/BCL3/SIGLEC16/W  NT5A/DRD2/SULF2/IL27RA/USP5  0/CD244/LILRA5/MNDA/CGAS/I  L7/NFATC4/ADRA2A/SERPINE1/  CLEC4E/PTAFR/TNFSF4/SPHK1/H  AVCR2/NFAM1/AFAP1L2/RUNX1  /TLR7/PANX2/CD86/IL1RL2/CA  SP1/CCL3/CCL19/SORL1/RNF12  5/FOXP3/PTGS2/CHIA/CLEC7A/  SCIMP/CCBE1/SASH3/FFAR2/LIL  RB2/TWIST1/RAB7B/IL13/IL18/E  BI3/HHLA2/CD28/CD6/LTB/PYHI  N1/IL10/CD160/CXCL17/IRF8/IF  NL1/CD40LG/PTGER4/IL1B/HPSE  /BIRC3/CADM1/NOD2/LILRB1/IR |
| --- | --- | --- | --- | --- |

| 0.497710826 14613 |  | tags=31%,  list=26%,  signal=23% |  | AACS/CD14/GLMN/C2CD2L/LRP  1/GAS6/TREM2/FZD5/MDM2/PY  DC1/CASP8/C1QTNF4/PARD6A/  FGG/CD274/CD34/BLK/NNAT/T  GFB3/PANX1/EGFR/KCNB1/TGFB  1/ITGB2/PLK3/PTP4A3/CEP135/  CDH1/TRPV4/SYTL4/GJA1/LGAL  S9B/ABLIM3/TRPM4/MYRIP/IL1R  L1/FGR/SYK/MICALL2/CORO2B/  CSF1R/CRLF2/AIF1/LGALS9/AC  HE/CEMIP/UCN3/ANO1/F2R/KCN  N4/OR51E2/FRMD4A/NLRP3/RAC  2/DOC2B/OSM/SLC51B/ORM1/C  AMK1/IL1A/RPH3AL/ADAM8/IL6  /RHOU/TP73/CD33/SLC30A8/HC  LS1/WNT5A/DRD2/USP50/ZIC1/  CD244/LILRA5/TGFB2/HCAR2/T  CAF2/CLEC4E/TNFSF4/FGB/HAV  CR2/TRH/PANX2/CASP1/CCL3/  CCL19/ANXA13/SORL1/SFN/PT  GS2/CASR/CHIA/FFAR2/GPR68/  VSNL1/TWIST1/TNFRSF4/IL13/M  TCL1/IL10/CD160/PRR5L/HPCA/  IGF1/PTGER4/IL1B/CADM1/NOD2  /IGHD/ADCY8/TLR8/GZMB/PAE  P/GATA3/CD38/SLC35D3/TENM  1/IL26/CD2/SHH/HTR2B/TNF/NL  RP7/ISL1/RASGRP1/PTPN22/ORM  2/CRH/CLEC9A/MMP12/AIM2/A  GTR2/CRTAM/HMGB4/CCL1/MM  P8/FGA/IFNG/IL17F/NLRP10/MIR  199B/IL2/MIR182 |
| --- | --- | --- | --- | --- |

| 0.497710826 14313 |  | tags=44%,  list=25%,  signal=33% |  | KLK7/ADORA2B/ZP3/FAM49B/C  D74/BTK/NFKBIZ/CLCF1/TGFB1/  SEMA7A/HMOX1/STXBP2/HLA-  G/ITGB2/FCER1G/LYN/HLA-  H/SPON2/FGR/SYK/UNC13D/AN  XA1/DDX60/LGALS9/CD1D/NLR  P3/RAC2/CD177/CD80/HLA-  F/KLK5/RSAD2/C3/HLA-  DMB/IL6/FCER2/WNT5A/CD244  /CD1C/PTAFR/TNFSF4/CD1E/CD  86/CD84/CD1A/SH2D1B/PGC/C  CL19/IL18RAP/FOXP3/VAV1/SCI  MP/SASH3/FFAR2/CD300A/TNF  RSF4/IL13/IL18/CD28/CD160/F  OXF1/IL1B/CADM1/NOD2/CD1B  /LILRB1/PTPRC/CCR2/MYB/GAT  A3/IL23R/IL12RB1/LBP/TBX21/N  CR3/KIR2DL4/FFAR3/TNF/XCL1/  SLAMF6/RASGRP1/MZB1/LAG3/  LTA/IL12B/KLRC4-  KLRK1/CRTAM/CLNK/SH2D1A/K  LRK1/IL21/IFNG/IL2 |
| --- | --- | --- | --- | --- |

| 0.497710826 12415 |  | tags=46%,  list=22%,  signal=36% |  | JAK3/ELANE/IGFBP2/SYK/SIRPB  1/ANXA1/AIF1/THY1/LGALS9/  TNFSF9/EFNB2/CD1D/HLA-  DPA1/NLRP3/PDCD1LG2/PIK3R6/  CD80/LILRB4/IGF2/HLA-  DMB/ADAM8/IL6/VSIR/EPO/IL7  /DPP4/PTAFR/TNFSF4/HAVCR2/  RUNX1/CORO1A/CD86/IL1RL2/P  TPN6/CCL19/FOXP3/ICOSLG/NC  KAP1L/VAV1/VCAM1/SASH3/D  USP10/LILRB2/CCL5/IL7R/IL18/  CD209/EBI3/HHLA2/CD28/CD6/  GLI2/CD160/CD40LG/IGF1/IL1B/  TNFSF13B/IL2RA/NOD2/CCL21/L  ILRB1/PTPRC/CCR7/CLECL1/RAS  AL3/CCR2/CARD11/IL15/MYB/  CD5/GATA3/IL23R/CD3E/SHH/IL  12RB1/CD27/SKAP1/GRAP2/TNF  SF14/TNF/ITGA4/LCK/ZAP70/X  CL1/RASGRP1/CD70/TNFSF11/R  HOH/TESPA1/IL12B/BTLA/KLRC4  -  KLRK1/PDCD1/SIRPG/KLRK1/ICO  S/CTLA4/IL21/IFNG/IL2 |
| --- | --- | --- | --- | --- |

| 0.497710826 12401 |  | tags=33%,  list=22%,  signal=26% |  | TRPM4/MYRIP/IL1RL1/FGR/SYK/  CSF1R/CRLF2/AIF1/LGALS9/AC  HE/UCN3/ANO1/F2R/KCNN4/OR  51E2/FRMD4A/NLRP3/DOC2B/OS  M/ORM1/ADORA1/TFR2/IL1A/R  PH3AL/ADAM8/IL6/CD33/SLC30  A8/WNT5A/DRD2/USP50/CD244  /LILRA5/TGFB2/HCAR2/CLEC4E  /TNFSF4/FGB/HAVCR2/TRH/PAN  X2/CASP1/CCL3/CCL19/SORL1  /CASR/CHIA/FFAR2/GPR68/VSN  L1/TWIST1/TNFRSF4/IL13/KISS1  /IL10/CD160/IGF1/PTGER4/GRP  /IL1B/CADM1/NOD2/IGHD/ADC  Y8/TLR8/ADCYAP1/PAEP/ILDR1  /GATA3/CD38/IL26/CD2/HTR2B  /TNF/NLRP7/ISL1/RASGRP1/PTP  N22/ORM2/CRH/CLEC9A/TNFSF1  1/MMP12/AIM2/AGTR2/CRTAM  /HMGB4/CCL1/MMP8/FGA/IFNG  /NPY2R/IL17F/NLRP10/MIR199B/  IL2/MIR182 |
| --- | --- | --- | --- | --- |

| 0.497710826 14047 |  | tags=30%,  list=25%,  signal=23% |  | FGG/STING1/CD274/PSMB10/BT  K/COLEC12/ICAM2/CTSS/PSMB  8/COCH/RELB/IFI16/HLA-  G/ITGB2/FCER1G/MUC2/IRF7/LY  N/MUC12/TXK/HRG/PELI1/SYK/  IRAK3/DDX60/LY96/PRKCA/PAR  P9/LY86/CD1D/CARD9/HLA-  F/PIK3AP1/KLK5/MUC5B/RSAD2  /RFTN1/ADAM8/CLEC4A/TICAM  2/MUC1/LILRA4/CLEC4C/SIGLEC  16/WNT5A/NLRC4/MNDA/CGAS  /IFNB1/HCK/CLEC4E/TRIL/FGB/  HAVCR2/TREML4/CD180/TLR7/C  D300LF/DMBT1/SH2D1B/PGLYRP3  /PGC/IL18RAP/RNF125/MUC3A/  CLEC7A/VAV1/SCIMP/FFAR2/PS  MB9/CCL5/CD300A/REG3G/RAB  7B/CD209/ESR1/PYHIN1/CD160/  NLRC5/BIRC3/CADM1/NOD2/SM  PDL3B/IRF1/TLR8/FPR2/CARD11  /ACOD1/LBP/NCR3/MUC6/NLRP  6/KIR2DL4/ZBP1/GFI1/SLAMF6/  RASGRP1/ICAM3/PTPN22/LAG3/  MUCL1/GBP5/IL12B/MMP12/TLR  10/MUC15/AIM2/PGLYRP2/KLRC  4-  KLRK1/CRTAM/HMGB4/CLNK/SH  2D1A/KLRK1/TNIP3/IL21/FCRL3/  FGA/NLRP10 |
| --- | --- | --- | --- | --- |

| 0.497710826 12401 |  | tags=39%,  list=22%,  signal=31% |  | ATP1B2/NKX2-  5/GJA1/TRPM4/ECE1/STC1/CA  CNA1H/AGT/CACNA1F/CACNB4  /CASQ2/KCND3/NPR1/HRH1/AT  P1A3/CACNA2D1/CHGA/F2R/OR  51E2/MIR26A2/KCNE1/KCNE2/A  DORA1/GJC1/CACNG1/TMIGD3/  GCH1/KCNK6/KCNMB4/AVPR1A/  HRH2/HRC/FOXN4/DRD2/DES/C  ACNB1/EDN2/TGFB2/NOS1AP/A  DRA2B/CORIN/ADRA2A/SCN1B/  PTAFR/CACNG5/SCN4B/FGB/OX  TR/ACE/CASQ1/THRB/BDKRB2/  ATP1A2/ABCC9/SCN2B/FXYD7/  ADRA1B/KCNK1/KCNE1B/PTGS2/  CASR/NPPC/SLC8A1/CELF2/DO  CK4/CACNA2D3/CAV3/CACNA2  D4/KCNH2/SLC1A1/ITPR2/CHRM  3/CLIC2/CALCA/HCN4/KCNJ11/  TNNT2/PIK3CG/GJA5/HTR2A/SM  TNL1/ADRA1A/P2RX1/CACNG4/  CD38/PLN/ATP2B2/KCNK3/MYL4  /KCNJ3/CACNG3/KCNJ4/TAC1/  NOS1/FXYD2/ADRB1/RYR2/CAC  NA1S/CACNG6/CACNG7/TRDN/  CACNA1G/ASIC2/AGTR2/TACR3  /SLC8A3/KCND2/CACNG2/FGA  /CHRM2/CSRP3/MC3R/IL2/MIR2  08A |
| --- | --- | --- | --- | --- |

| 0.497710826 14284 |  | tags=38%,  list=25%,  signal=28% |  | ADORA2B/CSPG5/ZP3/SDC1/FG  G/PRAM1/KCNB1/HMOX1/STXBP  2/SYP/ITGB2/FCER1G/LYN/CAC  NB2/RAB15/SYTL4/RAB26/CAC  NA1H/FGR/SYK/CHRNA5/UNC13  D/ANXA1/LGALS9/SYT12/GRIK5  /RAC2/CD177/DOC2B/HLA-  F/PRKCB/RPH3AL/P2RY1/DRD2/  CDK5R2/RAB27B/GRIN3A/ADRA2  A/PTAFR/FGB/SYT11/CD84/SY  T10/VSNL1/TRIM9/CD300A/IL13  /RAB3B/FOXF1/CADPS/TRPV6/P  RRT2/HTR2A/SYT1/ADRA1A/HA  P1/P2RX1/HTR1B/CCR2/SYT5/P  RKCG/ADCY1/SYT4/CHRNA4/SY  T6/SYT9/SYT2/RAB3C/CPLX2/  CACNA1G/CHRNA6/CACNA1I/DR  D1/RIMS2/PPFIA2/RIMS1/FGA/I  FNG/CHRM2/CHRNB3/CPLX3 |
| --- | --- | --- | --- | --- |

| 0.497710826 11178 |  | tags=32%,  list=20%,  signal=26% |  | MIR26A2/NLRP3/IL17B/SOCS3/C  YP19A1/HGF/OSM/PIK3AP1/AD  ORA1/NLRC3/C3/ADAM8/IL6/F  FAR4/WNT5A/KLF4/C2CD4A/CC  N3/LILRA5/NPY5R/TNFRSF1B/TN  FRSF11A/SERPINE1/HCK/TNFSF4  /SPHK1/ADAMTS12/SYT11/TLR7  /TNFAIP8L2/IL1RL2/NT5E/SELE/  CASP1/CCL3/PLA2G2A/TNFAIP6  /FOXP3/GGT2/CMA1/C2CD4B/S  UCNR1/PTGS2/MIR766/CLEC7A/  DUSP10/FFAR2/CCL5/MMP9/SL  AMF8/IL18/CD28/CD6/APCS/F1  2/GGT1/ESR1/CASP12/MEFV/IL  10/CXCL17/FOXF1/LRFN5/IGF1/  PTGER4/IL1B/BIRC3/ITGA2/IL2R  A/PIK3CG/NOD2/GGT3P/CCL24/  SMPDL3B/CST7/ADCYAP1/PTPRC  /CCR7/FPR2/IL1R2/CCR2/IL15/  GATA3/CD200R1/ABCD2/ACOD1  /LBP/SIGLEC10/NLRP6/FFAR3/S  TAP1/TAC1/TNF/TTBK1/XCL1/  NLRP7/ISL1/MIR3909/CNR2/TNF  SF11/LTA/GBP5/IL12B/TLR10/P  GLYRP2/IDO1/CREB3L3/CASP5/  CCL1/IL22RA2/IL21/MMP8/GHSR  /IL17F/NLRP10/MIR204/IL2/MIR  181A1/MIR138-2/MIR128-1 |
| --- | --- | --- | --- | --- |

| 0.497710826 14404 |  | tags=54%,  list=25%,  signal=41% |  | HLA-  DPB1/TYROBP/PAWR/ZP3/MAD1  L1/CD24/KITLG/CD274/CD74/B  TK/CLCF1/CD4/CSF1/HLA-  G/CCDC88B/LGALS3/LYN/BST1  /LGALS9B/FOXJ1/PELI1/JAK3/  CDKN1A/IGFBP2/TNFRSF21/AHR  /GREM1/SYK/ARG2/ANXA1/AIF  1/LGALS9/BST2/VSIG4/GPR183  /TNFSF9/CD1D/HLA-  DPA1/PDCD1LG2/RAC2/CD80/PL  A2G2D/IGF2/HLA-  DMB/IL6/VSIR/EPO/TNFSF18/M  NDA/CCL7/TNFRSF1B/IL7/RIPK3  /TNFSF4/INPP5D/PLA2G2E/LST1  /TNFSF8/HAVCR2/LMO1/CORO1  A/CD86/PTPN6/CCL19/PLA2G2A  /FOXP3/ICOSLG/SOX11/NCKAP1  L/VCAM1/SASH3/LILRB2/CLC/C  CL5/CD300A/TNFRSF4/IL13/IL18  /CD209/EBI3/HHLA2/CD28/CD6  /IL10/NFATC2/CLEC4G/OCSTA  MP/CD40LG/IGF1/IL1B/TNFSF13  B/IL2RA/LILRB1/IRF1/PTPRC/IL2  7/CLECL1/RASAL3/CCR2/CARD  11/IL15/IL23R/CD38/CD3E/SHH  /IL12RB1/SPN/TAC1/FGF10/ZAP  70/XCL1/PTPN22/MZB1/CD70/I  KZF3/IL12B/IDO1/TNFRSF13B/CT  LA4/IL21/FCRL3/IL2 |
| --- | --- | --- | --- | --- |

| 0.497710826 15225 |  | tags=44%,  list=27%,  signal=32% |  | RB2/KCNRG/RAMP3/NKAIN1/ST  AC3/ATP2A1/SCN5A/ANO6/B2  M/KCNE5/WNK2/KCNIP3/CAMK2  B/FXYD6/TREM2/HCRT/PLCG2/  AKAP6/EHD3/ATP1B1/CHD7/CX  CR4/STAC2/NKAIN4/CD4/METTL  21C/P2RX4/GPR35/GNB5/ANK2/  CRACR2B/LGALS3/LYN/CACNB2  /SLC30A10/CACNA1C/IL16/GAL  R2/ATP1B2/NKX2-  5/GJC2/GJA1/CXCL12/HAMP/P  DGFRB/STC1/FGF14/SPINK1/AG  T/SLC31A2/RCVRN/ATP2C2/CA  CNB4/CASQ2/THY1/CEMIP/TRPV  2/CACNA2D1/F2R/KCNAB1/MIR  26A2/P2RX2/NTSR1/CRACR2A/  GLRX/CCR1/KCNE1/KCNE2/ADO  RA1/CAMK2A/RGS9/CACNG1/H  OMER1/STC2/HRC/DYSF/CD33/  EPO/FGF12/DRD2/LILRA5/BDKRB  1/CD19/JPH1/CACNB1/REM1/N  OS1AP/F2RL3/ADRA2A/SCN1B/  RGN/LRRC38/STRIT1/SCN4B/APL  NR/MIR212/CASQ1/KCNMB1/AT  P1A2/CORO1A/SCN2B/FXYD7/C  D84/PTPN6/CRHR1/CCL3/JSRP1  /STAC/KCNE1B/MCHR1/P2RX5/  PTGS2/RRAD/CASR/SLC8A1/LIL  RB2/CCL5/GEM/TRPC6/IL13/CA  V3/KCNC1/HECW2/KCNH2/CLIC  2/HPCA/PIK3CG/HECW1/GRM6/  LILRB1/P2RY6/LRRC55/HTR2A/A  DCYAP1/KCNS2/HAP1/TRPV3/P2 |
| --- | --- | --- | --- | --- |

| 0.497710826 12096 |  | tags=48%,  list=21%,  signal=38% |  | CACNA1F/RCVRN/USH1G/TULP2  /RPE65/PRPH2/DHRS3/EYS/RHO  /PAX6/RDH5/TGFBI/GJC1/RGS9  /CLDN19/RP1L1/GNAT1/FSCN2/  RRH/GUCA1A/RGR/CRYGN/PDE6  H/RORB/SEMA5B/PDE6C/TACST  D2/RBP3/TULP1/RGS9BP/VSX2/  AIPL1/RDH12/LRIT3/GUCY2D/RA  X2/OPN1LW/LCTL/CDH23/DLL4  /USH1C/RAX/CACNA2D4/CABP  1/CRYGC/GLRB/CLIC5/POU6F2/  VSX1/CRYAA/NDP/KCNJ10/PDE  6A/USH2A/ZIC2/GRM6/CRYGA/  IMPG1/CRX/PAX2/ATP8A2/ABC  A4/ADGRV1/SOX14/PDC/OPN4/  RDH8/PCARE/GPR179/CNGB3/RD  3/CNGA1/IMPG2/KRT12/GABRR2  /GRK1/CNGB1/CLRN1/GRM8/EY  A4/MFRP/GUCA1C/NR2E3/GRK7  /CABP4/CPLX4/GJD2/SLC24A2/  RP1/OPN5/SLITRK6/CABP2/CNG  A3/RIMS1/CRYGD/GJA10/SLC4  A10/PPEF2/GUCY2F/PCDH15/GN  AT2/MYO3B/GLRA1/OPN1MW/C  PLX3/KERA |
| --- | --- | --- | --- | --- |

| 0.497710826 11598 |  | tags=30%,  list=21%,  signal=24% |  | CPE/TRPM4/MYRIP/INHBA/CHRN  A5/AGT/ANXA1/SV2A/PRKCA/  ASIC1/SYT12/UCN3/CRHBP/NA  AA/ANO1/SNCG/EPHA5/GRIK5/  CYP19A1/DOC2B/OSM/UNC13A  /ADORA1/CAMK2A/PRKCB/KCN  MB4/TFR2/RPH3AL/SLC6A9/IL6  /SCG5/FFAR4/IL1RN/SLC30A8/  P2RY1/DRD2/BRSK1/CCN3/INHBB  /SLC18A2/KMO/HCAR2/GRIN3A  /ADRA2A/DPP4/FGB/FOXD1/OT  OF/CHRNA3/SYT11/TRH/SNCAIP  /NRXN2/CRHR1/SOX11/CHAT/C  ASR/CACNA1E/FFAR2/GPR68/V  SNL1/CCL5/TRIM9/IL11/CCKAR  /ABCC8/FOXL2/KISS1/ITPR2/G  ALR1/KCNJ11/GRP/IL1B/CADPS  /G6PC2/PRRT2/MAFA/ADCY8/V  GF/HTR2A/PTPRN/ADCYAP1/PO  MC/SYT1/ADRA1A/P2RX1/HTR1  B/SYT5/MYB/LIF/ILDR1/GATA3  /PRKCG/NEUROD1/CD38/GRM4/  ADCY1/SYT4/CHRNA4/FGF23/S  YT6/NRXN1/SLC5A7/SYT9/SYT  2/HNF4A/FFAR3/TAC1/TNF/ISL  1/CRH/CPLX4/CPLX2/SLC2A2/  CHRNA6/SLC22A2/TNFSF11/TFA  P2B/MC4R/DRD1/GAD1/AGTR2/  RIMS2/OPRM1/SLC32A1/PPFIA2/  RIMS1/CARTPT/UNC13C/KCNC2  /FGA/IFNG/CHRM2/CHRNB3/SST  R5/GHSR/HTR2C/CPLX3 |
| --- | --- | --- | --- | --- |

| 0.497710826 12729 |  | tags=34%,  list=23%,  signal=27% |  | AKAP6/MYLK3/EHD2/SDC1/TBX  1/LDB3/NOX4/XK/TGFB1/TNNT  3/MYOF/ADAM12/SIX1/COMP/  LMOD2/PLEKHO1/NKX2-  5/KLHL41/NRAP/HAMP/KLHL40/  PDGFRB/CACNA1H/GREM1/AGT/  WT1/CASQ2/MSX1/ADGRB1/M  YOCD/TBX5/EFNB2/P2RX2/FZD7  /PI16/IGF2/CD53/HOMER1/LMO  D1/AVPR1A/DYSF/UCHL1/ADGR  B3/CCN3/DKK1/CCL8/MYOD1/A  LPK3/MYF6/MYH11/CASQ1/CDH  2/WNT10B/LMOD3/ACTA1/TME  M119/PGM5/NRG1/MYOZ2/SLC8  A1/CAV3/MIR199A1/NFATC2/S  YNPO2L/IGF1/MIR23A/TNNT2/NE  BL/ANKRD2/SIX4/ADRA1A/WFI  KKN2/GATA6/ALPK2/CXCL10/S  HH/TNFSF14/MYMX/NOS1/NPHS  1/BHLHA15/CXCL9/WNT1/MYPN  /MYMK/ACTC1/MYOM3/AGTR2  /BARX2/CSRP3/MYF5/NKX2-  6/MYOG/MIR204/MIR199B/MIR2  00B/MIR208A |
| --- | --- | --- | --- | --- |

| 0.497710826 13640 |  | tags=49%,  list=24%,  signal=37% |  | CD4/DTX1/EGR3/TCIRG1/RELB/  NKX2-3/HLA-  G/KIT/FCER1G/RUNX3/PREX1/N  RARP/AIRE/FOXJ1/JAG2/JAK3/  MAFB/SYK/LOXL3/CR1/ANXA1  /LGALS9/GPR183/TNFSF9/LFNG  /CD1D/NLRP3/PIK3R6/LEF1/FZD  7/CD80/LILRB4/PLA2G2D/RSAD2  /ADAM8/IL6/BCL3/VSIR/PAX1/  IL7/IFNB1/CLEC4E/RIPK3/TNFSF4  /TNFSF8/RUNX1/CD86/RUNX2/I  L1RL2/CCL19/DOCK2/LEPR/FOX  P3/ZEB1/NCKAP1L/VAV1/SASH3  /DUSP10/LILRB2/HLA-  DOA/IL7R/IL18/DLL4/CD28/PRD  M1/GLI2/FGL2/CCR9/IFNL1/NFA  TC2/PTGER4/IL2RA/TNFRSF18/E  OMES/IRF1/BATF/ZNF683/PTPRC  /CCR7/IL27/CCR2/LY9/CARD11  /IL15/MYB/GATA3/IL23R/CD3E  /CD2/SHH/IL12RB1/CD27/SPN/  TBX21/CD3D/LCK/ZAP70/SLAM  F6/RASGRP1/PTPN22/WNT1/CD8  A/FUT7/SPINK5/GPR18/LAG3/R  HOH/ITK/TESPA1/BCL11B/IL12B  /CCR6/CD3G/THEMIS/CTLA4/IF  NG/IL2 |
| --- | --- | --- | --- | --- |

| 0.497710826 12535 |  | tags=50%,  list=22%,  signal=39% |  | CATSPER1/GRIN2C/TRPM4/CACN  A1H/CATSPER3/CACNA1F/CACN  B4/CASQ2/VWC2/KCND3/SCNN  1G/CACNA2D1/DLG2/KCNN4/KC  NAB1/KCNJ1/GRIK5/KCNMA1/K  CNE1/KCNE2/CACNG1/SHANK1/  KCNK6/KCNMB4/SCN4A/HCN2/  MCUB/PKD1L3/KCNF1/CACNB1/  GRIN2D/NOS1AP/GRIN3A/KCNQ3  /SCN1B/KCNJ8/KCNV1/LRRC38/  KCNV2/PKD2L1/CACNG5/SCN4B  /KCNA2/KCNMB1/ABCC9/SCN2B  /KCNK1/SCN9A/CPT1C/KCNE1B  /SCNN1B/SCNN1A/CACNA1E/C  ACNA2D3/ABCC8/TRPC6/KCNC1  /CACNA2D4/KCNH2/SHISA9/HC  N4/KCNJ11/GRIA1/SHISA8/KCNJ  6/LRRC55/SCN7A/UNC80/KCNS2  /CACNG4/SHISA6/KCNQ2/CNGB  1/GRIK1/KCNJ3/KCNA3/CACNG  3/TRPC3/KCNJ4/NOS1/GRIA4/R  YR2/LRRC52/CACNA1S/KCNB2/  CACNG6/CACNG7/GRIN1/CACN  A1G/GRIN2A/KCNG4/CACNA1I/  GRIA3/CNTNAP2/KCNA4/KCNJ16  /OLFM3/SCN1A/SCN2A/VWC2L  /KCND2/SCN3A/GRIA2/TRPC7/H  TR3A/CACNG2/KCNC2/KCNA1/  KCNA10 |
| --- | --- | --- | --- | --- |

| 0.497710826 12143 |  | tags=34%,  list=21%,  signal=27% |  | AGT/FOXO6/C1QL1/CX3CR1/PA  K6/NGF/ASIC1/ABCA7/CRHBP/H  RH1/KCNAB1/MAPT/NRGN/NTSR  1/CCK/SGK1/FAM107A/RTL4/A  DORA1/SHANK1/HRH2/FOSL1/DR  D2/BRSK1/ADGRB3/ITGA5/PDE1  B/DKK1/SHC3/LRRN4/GABRA5/  NPTX2/NFATC4/MGAT3/STRA6/  OXTR/NLGN4Y/SLC17A7/ATP1A  2/SYT11/NRXN2/NLGN4X/PPP1R  1B/CCL11/PTGS2/GPR88/PTCHD  1/LILRB2/SLC6A1/NDRG4/ADAM  2/ARC/SLC7A11/KCNK10/LCE1D  /MAP1A/RELN/HOXA1/GRIA1/E  PHB2/NTRK1/ADCY8/HTR2A/EN1  /CALB1/PAK5/NRXN3/NTF3/BRI  NP1/PRKCG/ADCY1/SYT4/RASG  RF1/NETO1/CNTN2/CHRNA4/NR  XN1/CUX2/DRD5/TAC1/TNF/TT  BK1/CRH/FOXB1/SLC24A2/DRD3  /GRIN1/TNR/GRIN2A/CNTNAP2/  DRD1/JAKMIP1/NEUROD2/SCN2A  /TIFAB/SLC8A3/GRM5/TBR1/NE  UROG1/GHSR/DCANP1 |
| --- | --- | --- | --- | --- |

| 0.497710826 12813 |  | tags=30%,  list=23%,  signal=23% |  | RAB15/SYTL1/SYTL4/RAB26/SY  NGR3/SLC17A5/UNC13D/DPYSL3  /SV2A/SYTL3/NGF/CTTNBP2/S  YT12/MT3/UNC13A/RAB40AL/S  LC18A1/SLC6A9/SH3GL2/SYTL5  /DRD2/BRSK1/RAB27B/PENK/SL  C18A2/OTOF/SLC17A7/SYT11/  SNCAIP/SYT10/STON2/TRIM9/R  AB3B/TMEM163/AMPH/KCNK9/I  GF1/GRIA1/PRRT2/LAMP5/PTPRN  /SYT1/HAP1/NTF3/SYT5/SYT4  /GABRA2/SYT6/SNAP91/OPRD1  /SYT9/SYT2/RAB3C/DDC/DRD3  /GRIN1/GRIN2A/SLC32A1/SVOP  /SV2C/UNC13C/SLC17A8/SLC17  A6/OR56A5/CPLX3 |
| --- | --- | --- | --- | --- |

| 0.497710826 13273 |  | tags=57%,  list=23%,  signal=44% |  | G/F3/KIT/ITGB2/ACKR4/FCER1G  /CDH5/BTNL10/HLA-  H/S1PR1/RTN4RL1/MR1/UMODL1  /ABCC4/CXCL12/FOLR3/DNAI2/  ECE1/IL1RL1/PECAM1/ANXA1/C  UBN/CX3CR1/ITGA6/CRLF2/CD1  63/BTN3A3/THY1/CD79B/SCNN1  G/HEG1/SLC22A11/ITGA1/EPHA  5/CD1D/PDCD1LG2/CCR10/CCR1  /ULBP2/CD80/HLA-  F/ENOX1/TFR2/SCUBE1/IGHV3-  73/THBS1/SERPINA5/FCER2/CD3  3/CSF3R/TRGV5/ITGA5/PRLR/IL  27RA/CD244/NCAM1/CD1C/CD1  9/WNT2/TNFRSF11A/IGLC2/BTN  L8/STAB2/IGHA1/IGHM/FGB/EN  PEP/ACE/IGHA2/CCRL2/IGHV4-  59/IGHV5-51/IGHG2/IGHV1-  45/CD1E/CD86/IGHG1/IGHV4-  39/IGHE/CD1A/DMBT1/IGHV3-  23/IGKC/LEPR/ICOSLG/CD79A/  SCNN1B/IZUMO1R/VCAM1/GFRA  1/LRP2/SELL/RTBDN/IGHV3-  35/ANPEP/TRPM8/IGHV4-  31/IGHV4-  34/ASTN1/IGLC7/IGHV3-  48/TNFRSF4/IL7R/IL13/CD209/E  BI3/HHLA2/CD28/IGHV3-  11/CD6/CSF2RA/GFRA2/IGHV1-  69D/IGHV3-  21/ULBP1/IL2RG/IGHV1-  3/FCGR3A/IGHG3/IGHV4-  61/PLG/IGHV1- |
| --- | --- | --- | --- | --- |

| 0.497710826 10202 |  | tags=33%,  list=18%,  signal=27% |  | LILRA4/IGLV3-  21/CYFIP2/WAS/IGKV3-  20/IGLC2/HCK/IGLV2-  8/CLEC4E/MAPK10/ELMO1/IGHV  4-59/IGLV2-  14/IGHG2/IGHG1/LAT2/IGHV4-  39/IGHE/IGHV3-  23/IGKC/NCKAP1L/VAV1/IGLV2  -23/PSMB9/IGLV2-11/IGHV4-  34/IGKV3-15/IGLC7/IGHV3-  48/LCP2/IGLV3-19/IGKV1-  5/IGHV3-11/MYO1G/IGLV1-  44/FCGR3A/IGHG3/IGLC6/NFAT  C2/IGHV3-30/IGHV2-  5/IGLC3/IGHG4/FCGR1A/IGLV1-  40/IGKV1-17/IGHV3-  33/PTPRC/IGKV3D-  11/CARD11/IGHV2-70/IGHV3-  7/CD200R1/FCGR1B/IGLV3-  25/IGLV7-43/IGLV3-  1/CD247/IGKV1-  16/GRAP2/IGHV3-13/IGHV1-  69/IGKV5-2/IGKV2D-  28/IGKV2D-30/IGKV1-  12/IGHV3-  53/PIGR/ITK/IGKV3D-  20/IGKV2-29/IGKV1D-  12/IGKV2-30/CD3G/IGKV1D-  39/IGKV1-39/IGLV3-  27/IGKV1D-33/IGKV2-28 |
| --- | --- | --- | --- | --- |

| 0.497710826 12729 |  | tags=30%,  list=23%,  signal=23% |  | AKAP6/MYLK3/EHD2/SDC1/TBX  1/LDB3/NOX4/XK/TGFB1/TNNT  3/MYOF/ADAM12/SIX1/COMP/  ANK2/KIT/LMOD2/PLEKHO1/NKX  2-  5/KLHL41/NRAP/HAMP/KLHL40/  PDGFRB/CACNA1H/GREM1/AGT/  WT1/CASQ2/MSX1/ADGRB1/M  YOCD/TBX5/EFNB2/MIR26A2/P2  RX2/FZD7/PI16/CAMK1/IGF2/C  D53/HOMER1/LMOD1/AVPR1A/D  YSF/UCHL1/ADGRB3/CCN3/MAP  K12/DKK1/NFATC4/CCL8/MYOD  1/ALPK3/FGFR2/MYF6/MYH11/  CASQ1/CDH2/WNT10B/LMOD3/  ACTA1/TMEM119/PGM5/ZEB1/N  RG1/MYOZ2/SLC8A1/PRDM6/CA  V3/MIR199A1/FOXF1/NPNT/NFA  TC2/SYNPO2L/IGF1/MIR23A/TN  NT2/NEBL/ANKRD2/SIX4/ADRA1  A/WFIKKN2/GATA6/ALPK2/CXC  L10/SHH/TNFSF14/MYMX/NOS1  /FGF10/NPHS1/BHLHA15/CXCL9  /WNT1/MYPN/MYMK/ACTC1/M  YOM3/AGTR2/BARX2/CSRP3/M  YF5/NKX2-  6/MYOG/MIR204/MIR199B/MIR2  00B/MIR208A |
| --- | --- | --- | --- | --- |

| 0.497710826 13675 |  | tags=36%,  list=24%,  signal=27% |  | RX4/PIK3C2A/COMP/ANK2/KIT/  TNNI3K/TNNC2/CACNB2/CALCRL  /LMOD2/TNNI2/CACNA1C/GALR  2/NKX2-  5/GJA1/KLHL41/PPP1R12B/HAM  P/TRPM4/STC1/CACNA1H/ARG2  /AGT/PRKCA/PTGER3/SULF1/M  YBPH/CASQ2/AIF1/ACTG2/KCN  D3/MYOCD/CACNA2D1/CHGA/F  2R/ITGA1/HTR7/P2RX2/GATA5/  MYBPC3/P2RX6/KCNMA1/KCNE1  /KCNE2/TBX20/ADORA1/GJC1/  PI16/MYL10/TNNC1/SCN4A/HO  MER1/LMOD1/GUCY1A1/HRC/D  YSF/MYLK2/P2RY1/KLF4/FGF12  /DRD2/SULF2/DES/EDN2/TNFRS  F1B/NOS1AP/ADRA2B/ADRA2A/  SCN1B/KCNJ8/MYOD1/PTAFR/S  TRIT1/SCN4B/OXTR/SPHK1/MYH  11/CHRNA3/CASQ1/BDKRB2/RC  SD1/ATP1A2/SCN2B/LMOD3/AC  TA1/ADRA1B/JSRP1/STAC/KCN  E1B/PTGS2/MYOZ2/SLC8A1/DO  CK4/MSTN/CAV3/KCNH2/CHRM  3/MIR199A1/CLIC2/NPNT/CALC  A/HCN4/IGF1/MIR23A/IL1B/HTR  1D/ITGA2/TNNT2/SSTR2/PIK3CG  /NMUR2/GJA5/ANKRD2/SCN7A  /ATP8A2/HTR2A/TACR1/ADRA1  A/GATA6/P2RX1/MYOC/UTS2/  MYBPC1/IL15/CD38/PLN/MYL4/  HTR2B/KCNJ3/TRPC3/NOS1/RYR  2/MB/CACNA1S/KCNB2/TRDN/ |
| --- | --- | --- | --- | --- |

| 0.497710826 11609 |  | tags=31%,  list=21%,  signal=25% |  | ACHE/CEMIP/CRHBP/NPR1/ATP1  A3/CD1D/HLA-DPA1/HLA-  DRB5/TAP1/HLA-  F/PEX5L/AVPR1A/NPBWR1/MSR  1/GSTM1/PRLR/NGFR/PPIAL4G/  CRHR2/BDKRB1/CD1C/TMEM158  /HCRTR1/TGFB2/GCGR/PPIAL4C  /HLA-  DQB1/OXTR/ENPEP/EPHA4/CD1E  /PCSK5/SSTR1/CD1A/CRHR1/S  ORL1/LEPR/HLA-  DQA1/CMA1/MCHR1/GUCY2D/G  PR37L1/LILRB2/ANPEP/CCKAR/G  LP2R/CD209/CABP1/NPR3/GALR  1/GRIA1/ITGA2/EPHB2/SSTR2/N  OD2/CCKBR/CD1B/NMUR2/TRHD  E/FPR2/ADCYAP1R1/KPNA7/PPI  AL4H/CRIP1/SCTR/LBP/GPR149/  OPRD1/TRAV8-4/GRIA4/TRBV7-  9/GRIN1/TRGV9/TRAV29DV5/GR  IN2A/TRGV3/CALCR/GRIA3/MC4  R/HCRTR2/OPRM1/TRAV19/PTH2  R/SSTR3/GRIA2/GUCY2F/TRBV1  2-  3/MRGPRX2/SSTR5/GHSR/MC3R |
| --- | --- | --- | --- | --- |

| 0.497710826 12425 |  | tags=48%,  list=22%,  signal=38% |  | DGFRB/IL1RL1/IGFBP2/FGR/SYK/  SIRPB1/ANXA1/CRLF2/AIF1/THY  1/LGALS9/GPR183/TNFSF9/EFNB  2/CD1D/HLA-  DPA1/NLRP3/PDCD1LG2/PIK3R6/  CD177/CD80/HLA-  F/LILRB4/IGF2/IGHV3-73/HLA-  DMB/ADAM8/IL6/THBS1/VSIR/E  PO/WNT5A/LILRA5/IL7/IGLC2/I  GHA1/DPP4/PTAFR/TNFSF4/PLEK  /IGHM/INPP5D/HAVCR2/IGHA2/I  GHV4-59/RUNX1/IGHV5-  51/IGHG2/IGHV1-  45/CORO1A/CD86/IGHG1/IL1RL2  /IGHV4-  39/IGHE/PTPN6/CCL3/CCL19/IG  HV3-  23/IGKC/FOXP3/ICOSLG/NCKAP  1L/CLEC7A/VAV1/VCAM1/SAS  H3/DUSP10/LILRB2/IGHV3-  35/CCL5/IGHV4-31/IGHV4-  34/IGLC7/IGHV3-  48/TNFRSF4/IL7R/IL13/IL18/CD  209/EBI3/HHLA2/CD28/IGHV3-  11/CD6/IGHV1-  69D/GLI2/IGHV3-  21/IL10/CD160/IGHV1-  3/IGHG3/IGHV4-61/IGHV1-  18/IGLC6/NFATC2/IGHV3-  30/IGHV2-70D/IGHV2-  5/CD40LG/IGF1/IGLL5/IL1B/IGL  C3/TNFSF13B/IGHG4/IL2RA/NOD  2/TRDC/CCL21/LILRB1/IGHV1- |
| --- | --- | --- | --- | --- |

| 0.497710826 12019 |  | tags=37%,  list=21%,  signal=29% |  | HPN/SLC1A3/SLC24A4/CASQ2/  KCND3/ATP1A3/KCNN4/KCNAB1  /KCNJ1/KCNU1/KCNT1/MIR26A2  /KCNMA1/KCNE1/KCNE2/ADOR  A1/KCNK6/KCNMB4/KCNN3/HCN  2/TRPM5/DRD2/AQP1/KCNF1/N  OS1AP/ADRA2A/KCNQ3/KCNJ8/  KCNV1/LRRC38/KCNV2/PKD2L1/  MIR212/KCNA2/KCNMB1/ATP1A  2/ABCC9/KCNK1/ATP12A/KCNE  1B/KCNH7/ABCC8/CAV3/KCNC1  /KCNK10/SLC9C2/KCNH2/KCNK9  /HCN4/KCNJ10/KCNJ11/SLC9A7  /KCNK18/KCNK13/KCNJ6/GJA5  /KCNT2/LRRC55/HTR2A/ADCYA  P1/SLC12A3/KCNS2/SLC9A2/KC  NQ2/NETO1/KCNK3/KCNJ3/KCN  A3/KCNJ4/NOS1/FXYD2/LRRC52  /KCNH6/KCNB2/SLC24A2/DRD3  /KCNG4/KCNJ9/DRD1/KCNA4/K  CNJ16/KCND2/KCNH5/KCNC2/K  CNA1/OR56A5/KCNA10/MIR153  -1 |
| --- | --- | --- | --- | --- |

| 0.497710826 10132 |  | tags=44%,  list=18%,  signal=36% |  | 13/IL27RA/IGKV3-  7/CD244/CGAS/TGFB2/TNFRSF1  B/IGKV3-20/IFNB1/IGLV2-  8/HLA-  DQB1/TNFSF4/TRIL/IGLV2-  14/PGC/IGKC/IL18RAP/IGLV3-  9/FOXP3/SCIMP/IGLV4-  69/SASH3/FFAR2/IGLV2-  23/CLC/IGLV2-11/IGKV3-  15/TNFRSF4/TRAV38-  1/IL7R/IL13/IL18/IGLV5-  45/IGLV3-19/IGKV1-  5/CD28/IL10/CD160/IGLV1-  44/FGL2/IGLV8-  61/CD40LG/POU2F2/IGKV1-  9/IGLV4-  60/IL1B/TNFSF13B/NOD2/IGKV2  D-24/LILRB1/IGLV1-40/IGLV7-  46/BATF/IGKV1-17/IGLV3-  10/PTPRC/IGKV3D-11/IGLV10-  54/CCR2/CARD11/TRAV9-  2/GATA3/IGKV2-24/IGKV1-  27/IGLV3-25/IGLV7-43/IGLV9-  49/IGLV3-1/IGLV2-  18/TBX21/IGKV1-  16/TRAV40/KIR2DL4/FFAR3/IGL  V1-50/GCNT3/TNF/IGKV1-  8/XCL1/IGKV1-  6/APOA2/MZB1/IGKV1D-  43/TRAV18/CD96/IGKV5-  2/SPINK5/IGKV2D-28/IGKV2D-  30/IGKV1-37/IGKV1-  12/AICDA/TRAV2/IGKV3D- |
| --- | --- | --- | --- | --- |

| 0.497710826 14280 |  | tags=45%,  list=25%,  signal=34% |  | PLCG2/AKAP6/EHD3/ATP1B1/CH  D7/CXCR4/STAC2/CD4/METTL2  1C/P2RX4/GPR35/GNB5/ANK2/C  RACR2B/LGALS3/LYN/CACNB2/  CACNA1C/IL16/GJC2/GJA1/CX  CL12/PDGFRB/STC1/FGF14/SPIN  K1/AGT/RCVRN/ATP2C2/CACNB  4/CASQ2/THY1/CEMIP/TRPV2/  CACNA2D1/F2R/P2RX2/NTSR1/  CRACR2A/CCR1/KCNE2/CAMK2  A/RGS9/CACNG1/HOMER1/STC2  /HRC/DYSF/CD33/EPO/DRD2/LI  LRA5/BDKRB1/CD19/JPH1/CACN  B1/REM1/NOS1AP/F2RL3/ADRA2  A/RGN/STRIT1/APLNR/CASQ1/  ATP1A2/CORO1A/CD84/PTPN6/  CRHR1/CCL3/JSRP1/STAC/MCH  R1/P2RX5/PTGS2/RRAD/CASR/S  LC8A1/LILRB2/CCL5/GEM/TRPC  6/IL13/CAV3/CLIC2/HPCA/PIK3  CG/GRM6/LILRB1/P2RY6/HAP1/  TRPV3/P2RX1/CCL4/ADCYAP1R1  /EGF/PLN/CXCL10/CXCL11/OPR  D1/TRPC3/NOS1/XCL1/CXCL9/  RYR2/MRLN/CRH/TRDN/DRD3/G  RIN1/CALCR/TMC1/DRD1/FCRL3  /SEMG1/OR56A5 |
| --- | --- | --- | --- | --- |

| 0.497710826 14044 |  | tags=50%,  list=25%,  signal=38% |  | TK/PRAM1/APOBEC3F/NFKBIZ/C  LCF1/TGFB3/TGFB1/SEMA7A/H  MOX1/CPN2/STXBP2/APOBEC3G  /ANGPT1/C1QC/MIR26B/HLA-  G/ITGB2/FCER1G/C1QB/LGALS3  /LYN/HLA-H/STAT1/IGLV1-  51/PROS1/FOXJ1/SPON2/JAK3/  TRPM4/CFP/DNASE1L3/FGR/SYK  /LOXL3/UNC13D/CR1/IRAK3/AN  XA1/DDX60/HTRA1/C2/CFI/C3  AR1/PARP9/LGALS9/BST2/IGLV  1-47/VSIG4/IGKV4-  1/CD1D/NLRP3/PIK3R6/RAC2/N  CR1/CD177/IGLV6-  57/CD80/HLA-  F/KLK5/C5AR2/RSAD2/C3/HLA  -DMB/IL6/C8G/FCER2/IGLV3-  21/WNT5A/IL27RA/C1R/CD244  /CD1C/CD19/WAS/CGAS/TGFB  2/TNFRSF1B/IGKV3-  20/IGLC2/IFNB1/IGLV2-  8/RIPK3/PTAFR/TNFSF4/TRIL/H  AVCR2/C1S/CFB/IGHV4-  59/IGLV2-  14/IGHG2/CD1E/CD86/IGHG1/IG  HV4-  39/CD84/CD1A/PTPN6/SH2D1B/  PGLYRP3/PGC/CCL19/IGHV3-  23/IGKC/IL18RAP/VTN/RNF125/  FOXP3/VAV1/SCIMP/SASH3/DU  SP10/FFAR2/IGLV2-  23/CLC/CD300A/IGLV2-  11/IGHV4-34/SLAMF8/IGKV3- |
| --- | --- | --- | --- | --- |

| 0.497710826 13759 |  | tags=35%,  list=24%,  signal=26% |  | COL8A1/NECTIN1/EGFR/SH3PXD  2B/NECTIN3/PRSS56/SEMA3A/T  CIRG1/ROM1/SLC44A4/MYO7A/  CRYGB/ARHGEF15/RET/CACNA1  C/CYP1A1/SLC39A5/MAN2A1/  GRHL3/SLC4A5/PDGFRB/MEIS2/I  NHBA/JAG1/WT1/NRP1/DLX2/F  OXC1/RPE65/THY1/ACHE/PRPH2  /COL4A1/FJX1/GDF11/RHO/PA  X6/RP1L1/C3/COL5A1/GNAT1/  FOXN4/WNT5A/KLF4/FSCN2/W  NT2/TGFB2/WNT9B/CRYGN/FOX  C2/STRA6/RORB/WNT9A/PDE6C  /SLC17A7/THRB/BMP7/GDF3/NH  S/TULP1/MAB21L1/SOX2/SOX1  1/ZEB1/LCTL/CELF4/TWIST1/B  MPR1B/MAB21L2/GPM6A/FOXL2  /SLC7A11/DLL4/PRDM1/MYH15  /USH1C/RAX/HES5/PROM1/MEG  F11/CRYGC/HPCA/VSX1/NDP/PI  TX3/LAMA1/CNTF/EPHB2/PDE6  A/SDK2/GRM6/PHOX2B/CRYGA  /UNC45B/PAX2/ATP8A2/NTRK3  /POU4F1/CALB1/GNGT1/GATA3  /CRB1/NEUROD1/RD3/DSCAM/S  HH/MFRP/NEUROD4/NR2E3/FGF1  0/ADAMTS18/OSR2/VAX1/ISL1  /CABP4/RP1/BCL11B/TFAP2B/S  LITRK6/OLFM3/LHX1/FASLG/CR  YGD/GNAT2/SLC17A8/POU4F2/  MYF5/KERA |
| --- | --- | --- | --- | --- |

| 0.497710826 12029 |  | tags=34%,  list=21%,  signal=27% |  | ACNB4/SIPA1L1/CTTNBP2/ACHE  /POTEKP/ADGRB1/COL4A1/CLST  N2/DLG2/F2R/ADD2/MAPT/SNC  G/SEMA3F/CNKSR2/EFNB2/P2RX  2/GABRB3/UNC13A/SHANK1/CA  MK1/LRP8/IL1RAPL2/C3/HOMER  1/NEFH/TNC/HCLS1/WNT5A/DR  D2/ADGRB3/SPARCL1/LRRC24/N  FASC/DNM3/DKK1/CACNB1/NO  S1AP/KIRREL3/NFATC4/THBS2/S  LITRK5/OXTR/EPHA4/NLGN4Y/G  PC6/SYNDIG1/SRCIN1/LRRC4/M  AP1B/NRXN2/CDH2/DOCK10/NL  GN4X/SPOCK2/PCDH17/MDGA1/  IL10RA/SLITRK2/NRG1/TMEFF2/  LILRB2/LRFN2/GPM6A/ARC/ITPK  A/SLC7A11/ADGRB2/GLRB/IL10  /CDH9/LHFPL4/SEMA3E/GABRB2  /RELN/GPC4/LRFN5/NTN1/EPHB2  /PTPRO/SEZ6/INA/SDK2/NTNG2  /NTRK1/IGSF21/NEFL/LRRTM1/L  RRC4C/SIX4/NTRK3/POU4F1/IGS  F9/LRRN3/AMIGO2/SHISA6/PCD  HGC4/LRTM1/GABRA2/CNTN2/D  SCAM/SLIT1/PTPRD/CNTN5/NRX  N1/ADGRL3/CUX2/EPHA7/SLITR  K4/PCDH8/TNF/CAMKV/LRRTM2  /SLITRK1/GABRA1/GRIN1/DGKB  /ZNF804A/TNR/GRID2/ZNF365/  ASIC2/SLITRK6/FRMPD4/CBLN2/  DRD1/NEUROD2/PPFIA2/LRTM2/  GJA10/GRM5/GABRG2/UNC13C/  SLITRK3/CACNG2/LRRTM3/GHSR |
| --- | --- | --- | --- | --- |

| 0.497710826 13713 |  | tags=37%,  list=24%,  signal=28% |  | SLC38A9/ABCB1/SLC1A4/ACAC  B/SLC16A3/GABRR1/SLC44A4/S  LC38A6/SLC13A4/SLC26A10/SL  C4A11/ANO7/GJA1/SLC9A3/SL  C26A9/SLC4A5/SLC46A1/GABRE  /AGT/CLIC6/SLC1A3/SLC24A4/  SLC6A12/ANO1/GABRG3/NTSR1  /SLC4A4/GABRB3/ANO2/THRSP  /SLC16A11/SLC6A9/THBS1/GAB  RQ/SLC16A8/SLC7A3/SLC7A7/  GABRA5/SLC6A20/ADAMTS8/A  NO4/TCAF2/SLC26A7/PTAFR/CL  CNKB/STRA6/SLC26A3/SLC17A7  /CLCNKA/ATP1A2/BSND/SLC38  A5/CLCA4/SLC38A4/CASR/LRP  2/SLC38A8/CLDN4/SLC26A8/G  ABRA4/GABRD/APOL1/SLC7A11  /CLIC3/SLC9C2/EMB/GLRB/CLIC  5/SLC1A1/GABRB2/SLC38A3/CL  IC2/FOLR1/KCNJ10/SLC5A12/SL  C9A7/SLC34A1/SLC1A7/SLC36A  2/NMUR2/ANO9/SLC12A3/GABR  A3/SLC9A2/SLC13A5/SLC4A8/  GABRA2/BEST3/GABRR2/SLC5A8  /GLRA2/SLC1A2/SLC24A2/GAB  RA1/SLC22A6/SLC6A18/GABRR3  /BEST2/SLC32A1/GLRA3/GABR  A6/SLC4A10/GRM5/GABRG2/SL  C17A8/ANO3/GABRG1/GLRA1/S  LC17A6/SLC17A1 |
| --- | --- | --- | --- | --- |

| 0.497710826 11714 |  | tags=43%,  list=21%,  signal=34% |  | N4/HLA-DPA1/HLA-  DRB5/THEMIS2/LILRB4/FYB2/PRK  CB/RFTN1/IGHV3-73/HLA-  DQA2/MNDA/CD19/WAS/FYB1  /IGLC2/BTNL8/IGHA1/HLA-  DQB1/GCSAML/IGHM/INPP5D/IG  HA2/NFAM1/IGHV4-  59/RUNX1/IGHV5-  51/IGHG2/IGHV1-  45/IGHG1/LAT2/IGHV4-  39/IGHE/PTPN6/IGHV3-  23/IGKC/FOXP3/ICOSLG/HLA-  DQA1/CD79A/HLA-  DQB2/NCKAP1L/IGHV3-  35/PSMB9/CD300A/IGHV4-  31/IGHV4-34/IGLC7/IGHV3-  48/LCP2/HHLA2/CD28/IGHV3-  11/IGHV1-69D/IGHV3-  21/KLHL6/CD160/IGHV1-  3/IGHG3/IGHV4-61/IGHV1-  18/IGLC6/NFATC2/IGHV3-  30/IGHV2-70D/IGHV2-  5/IGLL5/IGLC3/IGHG4/TRDC/TR  AC/BTN1A1/IGHV1-  58/IGHD/IGHV4-28/IGHV1-  24/IGHV3-  33/PTPRC/CCR7/CARD11/IGHV2  -70/PAX5/GATA3/IGHV3-  7/CD38/TRBC2/CD3E/GCSAM/I  GHV3-20/CD247/IGHV3-  74/SKAP1/GRAP2/IGHV3-  13/IGHV4-  4/CD3D/STAP1/GBP1/TRAV8- |
| --- | --- | --- | --- | --- |

| 0.497710826 9827 |  | tags=41%,  list=17%,  signal=34% |  | IGLC2/IFNB1/IGHA1/TNFSF4/IGH  M/INPP5D/IGHA2/NFAM1/CDH17  /IGHV4-59/IGHV5-  51/CD180/IGHG2/IGHV1-  45/IGHG1/LAT2/IGHV4-  39/IGHE/DOCK10/PTPN6/IGHV3-  23/IGKC/FOXP3/ICOSLG/ONECU  T1/CD79A/NCKAP1L/BLNK/VCA  M1/SASH3/IFNW1/IGHV3-  35/IL11/CD300A/IGHV4-  31/IGHV4-  34/SLAMF8/IGLC7/IGHV3-  48/TNFRSF4/IL7R/IL13/IFNE/CD  28/IGHV3-11/IGHV1-  69D/IGHV3-21/IL10/IGHV1-  3/IGHG3/IGHV4-61/IGHV1-  18/IGLC6/NFATC2/IGHV3-  30/IGHV2-70D/IGHV2-  5/CD40LG/POU2F2/IGLL5/IGLC3  /TNFSF13B/IGHG4/NOD2/TRDC/  TBC1D10C/NTRK1/IGHV1-  58/IGHD/BATF/IGHV4-  28/IGHV1-24/IGHV3-  33/PTPRC/SAMSN1/CARD11/IGH  V2-70/IGHV3-  7/CD38/TRBC2/FLT3/CHRNA4/I  GHV3-20/CD27/TBX21/IGHV3-  74/BANK1/IGHV3-13/IGHV4-  4/ITGA4/IGHV3-  66/ZAP70/IGHV3-72/IGHV1-  69/MZB1/CD70/SLA2/AICDA/IK  ZF3/IGHV3-53/IGHV6-1/IGHV3-  64/CCR6/IGHV3-38/IGHV7- |
| --- | --- | --- | --- | --- |

| 0.497710826 13038 |  | tags=31%,  list=23%,  signal=24% |  | PLEKHA1/PTPRH/TRPV4/SYTL1/P  LEKHO1/SLC9A3/FGD5/SLC46A1  /CDHR2/GABRE/FGR/CUBN/SPA  TA13/ABCA7/ATP8B1/AIF1/ATP  6V0A4/THY1/DHRS3/GABRG3/M  APT/CDHR1/RHO/FAM107A/CEA  CAM20/ADORA1/JCAD/GNAT1/  PLA2G4F/DRD2/ITGA5/AQP1/GA  BRA5/LCP1/AMN/CDHR5/DPP4/  PDE6H/PKD2L1/PLEK/SLC26A3/K  CNA2/PACSIN1/HHIP/ITLN1/KCN  K1/MCHR1/SCNN1A/SCIMP/GPR  37L1/LRP2/NDRG4/GABRA4/SLC  34A2/RASGRP2/SLC7A11/FGD2/  KCNC1/PROM1/UNC5A/HPCA/SH  ISA9/FOLR1/KCNJ11/GRIA1/SHI  SA8/SLC34A1/USH2A/ITGB3/U  MOD/TBC1D10C/LAMP5/AKAP5/  ADGRV1/GABRA3/SHISA6/PSD2  /CNGA1/GABRA2/ABCC2/CNGB  1/OPRD1/DRD5/SLC1A2/ANK1/  GABRA1/DPEP1/SLC6A18/CNTN  AP2/DRD1/REG1A/OPRM1/TACR  3/GABRA6/SSTR3/GABRG2/GNA  T2/KCNC2/GABRG1/SLC17A3 |
| --- | --- | --- | --- | --- |

| 0.497710826 9401 |  | tags=46%,  list=17%,  signal=38% |  | IGLC2/IGHA1/VCAN/IGHM/EPHA  4/HAVCR2/IGHA2/IGHV4-  59/IGHV5-51/IGHG2/IGHV1-  45/IGHG1/TULP1/IGHV4-  39/IGHE/CCL19/IGHV3-  23/DOCK2/IGKC/ZP2/ZP1/IZUM  O1R/NEXN/CLEC7A/IGHV3-  35/IGHV4-31/IGHV4-  34/ADAM2/IGLC7/IGHV3-  48/CLGN/CD209/IGHV3-  11/CD6/IGHV1-69D/IGHV3-  21/EMB/NTM/IGHV1-  3/IGHG3/IGHV4-61/IGHV1-  18/IGLC6/IGHV3-30/IGHV2-  70D/IGHV2-  5/IGLL5/IGLC3/CADM1/IGHG4/E  PHB2/TRDC/CCL21/PRF1/IGHV1  -58/IGHD/DSCAML1/IGHV4-  28/IGHV1-24/IGHV3-  33/CCR7/IGSF9/ZPBP/FOXG1/C  D5/IGHV2-70/PAEP/IGHV3-  7/TRBC2/CNTN2/DSCAM/IGHV3  -20/OPCML/NCR3/IGHV3-  74/IGHV3-13/IGHV4-4/IGHV3-  66/FCN2/IGHV3-72/IGHV1-  69/FEZF2/MYPN/IGHV3-  53/IGHV6-1/IGHV3-  64/CNTNAP2/CRTAM/IGHV3-  38/IGHV7-81/IGHV3-  43/TRBC1/ADAM30/ZPBP2/IGHV  3-16 |
| --- | --- | --- | --- | --- |

| 0.497710826 11908 |  | tags=47%,  list=21%,  signal=37% |  | PRKCA/VIPR1/PTGER3/ADGRG1/  ADGRG3/MC1R/UCN3/ADGRB1/H  RH1/CHGA/HTR7/CCR1/ADGRL2  /OR11H7/ADORA1/ADCY4/PTGE  R1/HRH2/GNAT1/P2RY1/GNA15  /HRH4/DRD2/ADGRB3/GRM2/CR  HR2/APLP1/GPR176/ADRA2B/AD  RA2A/GCGR/PTGFR/HTR4/MRAP  /ADGRE3/PTGIR/SSTR1/ADRA1B  /CRHR1/RGS1/PSAPL1/MCHR1/  CASR/GPR37L1/INSL3/TSHR/AD  GRG6/GLP2R/UCN2/ADM2/ADGR  B2/GRM3/FPR1/NPR3/CHRM3/FS  HR/GALR1/CALCA/PTGER4/PTHL  H/GPR65/HTR1D/SSTR2/GRM6/G  PHA2/S1PR4/ADCY8/HTR2A/AD  CYAP1/AKAP5/NPY/ADRA1A/FP  R2/HTR1B/GRM4/ADRB3/ADCY1  /PLN/CXCL10/MC5R/ADGRE1/G  RM8/ADGRL3/CXCL11/ADCY2/O  PRD1/HTR2B/FFAR3/CCR3/DRD5  /NOS1/LGR5/ADGRG4/ADRB1/C  XCL9/ADGRG5/DRD3/CNR2/HTR  1F/CALCR/MC4R/DRD1/GRM7/A  GTR2/OPRM1/HTR5A/RIT2/XCR1  /SSTR3/CALCB/GNAT2/CHRM2/  HTR1E/NPY2R/SSTR5/OR5T2/OR  56A5/HTR2C/MC3R |
| --- | --- | --- | --- | --- |

| 0.497710826 12535 |  | tags=50%,  list=22%,  signal=39% |  | /GABRE/CACNA1H/CHRNA5/CA  TSPER3/CLIC6/CACNA1F/HPN/C  ACNB4/ASIC1/KCND3/SCNN1G/  ANO1/CACNA2D1/GABRG3/KCN  N4/KCNAB1/KCNJ1/KCNU1/KCN  T1/P2RX2/GRIK5/P2RX6/GABRB3  /KCNMA1/ANO2/KCNE1/KCNE2  /CYBB/CACNG1/KCNK6/KCNMB4  /SCN4A/KCNN3/GABRQ/HCN2/  TRPM5/TMEM37/AQP1/KCNF1/J  PH1/CACNB1/GABRA5/GRIN2D/  GRIN3A/KCNQ3/SCN1B/ANO4/K  CNJ8/KCNV1/LRRC38/KCNV2/CL  CNKB/PKD2L1/CACNG5/SCN4B/  SLC17A7/CHRNA3/CLCNKA/KCN  A2/KCNMB1/GRID1/SCN2B/TRP  M2/KCNK1/SCN9A/CLCA4/KCNE  1B/P2RX5/SCNN1B/SCNN1A/KC  NH7/CACNA1E/FAM155A/GABR  A4/CACNA2D3/GABRD/KCNC1/  KCNK10/CLIC3/CACNA2D4/KCNH  2/GLRB/CLIC5/ITPR2/GABRB2/T  RPM3/CLIC2/KCNK9/HCN4/KCNJ  10/KCNJ11/GRIA1/KCNK18/NMU  R2/KCNK13/KCNJ6/KCNT2/CALH  M1/LRRC55/SCN7A/ANO9/KCNS  2/P2RX1/HTR1B/CACNG4/GABR  A3/KCNQ2/CNGB3/CNGA1/GAB  RA2/CHRNA4/GABRR2/KCNK3/C  NGB1/GRIK1/CHRNA9/CHRNA2/K  CNJ3/KCNA3/CACNG3/KCNJ4/G  LRA2/GRIA4/RYR2/LRRC52/KCN  H6/CACNA1S/KCNB2/CACNG6/ |
| --- | --- | --- | --- | --- |

| 0.497710826 19343 |  | tags=47%,  list=34%,  signal=31% |  | CHCHD10/SLC25A17/VAMP5/EM  C6/UQCC3/SYVN1/TEX261/PGA  P3/ZDHHC20/SYNGR1/AUP1/SLC  35A4/MFF/ESYT1/RCE1/NPC1/S  PG7/PIGT/TOMM40/EMC1/PREB/  MUL1/COA1/TMEM11/TRAM1/E  MC7/TMCO1/TIMM17B/PISD/EX  T1/FAR2/SLC37A1/SLC35A2/YI  F1B/VAMP1/TAPBP/ELOVL1/ZMP  STE24/DOLK/PIGU/DERL2/RAB35  /CYP2E1/SLC35B1/DPM2/TIMM1  7A/LBR/RER1/STIM1/DOLPP1/FI  CD/MTLN/YIF1A/SYN1/SPPL2A/  WFS1/SELENOS/DPAGT1/SLC37  A3/ABCB10/GPAA1/CLSTN1/SL  C66A1/TBL2/SLC37A2/RHBDD1/  PKD2/DHCR7/CNR1/EMC2/ETFDH  /SLC30A3/MICOS10/RHBDD2/SL  C8B1/EMC9/HLA-  E/PCSK7/CHST12/CLN3/SAMD8  /SLC6A17/ABCB6/FITM1/HACD1  /BNIP1/TMEM70/HM13/DERL1/B  2M/MCU/HLA-  DPB1/STING1/CD74/DGAT2/SLC  38A9/TAP2/DHRS9/HACD4/P2RX  4/SYP/HLA-DRA/COQ2/HLA-  G/MBOAT4/SLC22A17/MAJIN/H  LA-H/HLA-  DRB1/ELOVL7/SFXN1/RAB26/VO  PP1/ECE1/CHST4/SV2A/CHST2/  PCSK2/B4GALNT2/MGARP/SGMS  1/LFNG/HLA-  DPA1/P2RX2/HLA- |
| --- | --- | --- | --- | --- |

| 0.497710826 14812 |  | tags=51%,  list=26%,  signal=38% |  | GLMN/TREM2/HLA-  DPB1/TYROBP/PAWR/IL34/ZP3/  MAD1L1/CD24/KITLG/CD274/PS  MB10/CD74/BTK/CLCF1/GAPT/  CD4/CSF1/TCIRG1/HLA-  G/KIT/CCDC88B/LGALS3/LYN/B  ST1/LGALS9B/FOXJ1/PELI1/JAK  3/CDKN1A/IGFBP2/TNFRSF21/A  HR/GREM1/SYK/ARG2/PLCL2/A  NXA1/CSF1R/AIF1/LGALS9/BST  2/VSIG4/GPR183/TNFSF9/CD1D  /HLA-  DPA1/PDCD1LG2/LEF1/RAC2/CD  80/PLA2G2D/IGF2/HLA-  DMB/IL6/VSIR/EPO/TNFSF18/M  NDA/CD19/CCL7/TNFRSF1B/IL7  /IFNB1/RIPK3/TNFSF4/INPP5D/PL  A2G2E/LST1/ACE/TNFSF8/HAV  CR2/LMO1/CD180/CORO1A/CD8  6/PTPN6/CCL19/DOCK2/PLA2G2  A/FOXP3/ICOSLG/SOX11/CD79  A/NCKAP1L/VCAM1/SASH3/LIL  RB2/IFNW1/CLC/CCL5/CD300A  /TNFRSF4/IL7R/IL13/IL18/CD20  9/IFNE/EBI3/HHLA2/CD28/CD6/  IL10/NPR3/NFATC2/CLEC4G/OC  STAMP/CD40LG/IGF1/IL1B/TNFS  F13B/IL2RA/PIK3CG/LILRB1/IRF1  /PTPRC/IL27/CLECL1/RASAL3/C  CR2/CARD11/IL15/IL23R/CD38/  FLT3/CD3E/SHH/IL12RB1/SPN/T  NFSF14/TAC1/FGF10/ZAP70/XC  L1/PTPN22/MZB1/CD70/IKZF3/T |
| --- | --- | --- | --- | --- |

| 0.497710826 10498 |  | tags=35%,  list=19%,  signal=28% |  | 21/DRD2/APOC2/ARHGAP27/MX  2/SFRP4/DNM3/DKK1/BMP2KL/I  GKV3-  20/AMN/SCGB3A2/IGLC2/SERPI  NE1/IGLV2-  8/STAB2/IGHA1/IGHA2/IGHV4-  59/RAB31/IGLV2-  14/SYT11/IGHV4-  39/SELE/MIR27B/CCL19/IGHV3-  23/SORL1/IGKC/VTN/GSG1L/LR  P2/IGLV2-23/IGLV2-11/IGHV4-  34/IGKV3-15/IGLC7/IGHV3-  48/ARC/APOL1/IGLV3-  19/CAV3/IGKV1-5/IGHV3-  11/IGLV1-  44/MIR199A1/HPCA/IGLC6/JCH  AIN/IGHV3-30/FOLR1/IGHV2-  5/GRIA1/IGLC3/SAG/CCL21/ITG  B3/FCGR1A/LILRB1/IGLV1-  40/LRRTM1/LRP1B/IGKV1-  17/IGHV3-33/IGKV3D-  11/FPR2/HTR1B/NTF3/CACNG4/  CALY/IGHV2-70/IGHV3-  7/FCGR1B/EGF/SH3GL3/SGIP1/I  GLV3-25/IGLV7-  43/CNTN2/IGLV3-  1/SNAP91/IGKV1-  16/HTR2B/IGHV3-  13/CACNG3/ITGA4/LRRTM2/IGH  V1-69/HPR/DRD3/IGKV5-  2/IGKV2D-28/IGKV2D-  30/IGKV1-12/CLEC9A/IGHV3-  53/IGKV3D-20/IGKV2- |
| --- | --- | --- | --- | --- |

| 0.497710826 11149 |  | tags=40%,  list=20%,  signal=32% |  | 1/GPR6/CMKLR1/ADORA1/C5AR  2/PTGER1/AVPR1A/HRC/PLCD4/  P2RY1/GNA15/EPO/FPR3/WNT5  A/HRH4/DRD2/BDKRB1/CD19/JP  H1/HCRTR1/EDN2/GRIN2D/P2RY8  /F2RL3/CD52/PTGFR/OXTR/APL  NR/PTGIR/CCRL2/CASQ1/BDKRB  2/ATP1A2/CORO1A/TRPM2/ADR  A1B/PTPN6/CCL3/CCL19/F2RL2  /JSRP1/MCHR1/P2RX5/SLC8A1/  FAM155A/CDH23/TRPC6/IL13/C  AV3/ESR1/KISS1/ITPR2/GRM1/F  PR1/MIR199A1/CLIC2/CCR9/GA  LR1/GPR20/CALCA/PTGER4/GRI  A1/GPR65/TRPV6/CALB2/PTGDR  /PDE6A/PLCH2/PIK3CG/CCKBR/  CCL21/NMUR2/P2RY6/S1PR4/AD  CY8/TMEM178A/CYSLTR1/PLCH  1/HTR2A/ADCYAP1/TACR1/CAL  B1/PTPRC/CCR7/ADRA1A/FPR2/  HAP1/P2RX1/HTR1B/CCR2/ADC  YAP1R1/CCR5/CD38/PLN/CXCL1  0/ATP2B2/KCNK3/CNGB1/CXCL  11/CHRNA9/HTR2B/CCR3/TRPC3  /TAC1/NOS1/CXCR3/LCK/XCL1  /CXCL9/CCR4/SCGN/CXCR6/RY  R2/TRDN/DRD3/GRIN1/GPR18/G  RIN2A/CALCR/DRD1/HCRTR2/CC  R6/GPR174/SLC8A3/FASLG/GPR  55/MS4A1/XCR1/GRM5/P2RY10  /CXCL13/TRPC7/CALCB/CXCR5  /GNAT2/CCL1/CCR8/NPY2R/OR  56A5/HTR2C/GPR32P1/IL2 |
| --- | --- | --- | --- | --- |

| 0.497710826 12157 |  | tags=33%,  list=22%,  signal=26% |  | CHRNA5/ARG2/AGT/SV2A/CX3  CR1/SLC1A3/ASIC1/AIF1/ACHE  /SYT12/NAAA/SNCG/GRIK5/NT  SR1/DOC2B/BHMT/UNC13A/CA  MK2A/PRKCB/GCH1/SLC18A1/S  DS/KCNMB4/NQO1/PRIMA1/SLC  6A9/TDH/P2RY1/KLF4/DRD2/DD  AH1/BRSK1/DPYD/PDE1B/MOXD  1/SLC18A2/KMO/NOS1AP/GRIN  3A/RGN/MAOB/OTOF/MIR212/S  LC17A7/CHRNA3/ATP1A2/SYT1  1/TRH/SNCAIP/NRXN2/GLYAT/  SLC6A3/CHAT/PTGS2/CLEC7A/  AGXT2/TRIM9/SLC6A1/SLC7A11  /RAB3B/IL10/SLC6A2/SLC1A1/  MIR199A1/KCNJ10/IL1B/CADPS/  PRRT2/SLC1A7/ITGB3/HTR2A/S  YT1/ADRA1A/P2RX1/HTR1B/SY  T5/GLDC/PRKCG/GRM4/ADCY1/  SYT4/GABRA2/CHRNA4/SYT6/N  RXN1/SLC5A7/SYT9/SYT2/NOS  1/TNF/SLC1A2/DDC/CPLX4/CPL  X2/DRD3/HDC/GRIN2A/CHRNA6  /SLC22A2/DRD1/GAD1/DPYS/A  GTR2/RIMS2/SLC32A1/PPFIA2/K  LRC4-  KLRK1/RIMS1/UNC13C/KLRK1/M  MP8/IFNG/CHRM2/CHRNB3/CPLX  3/MOXD2P/MIR99B |
| --- | --- | --- | --- | --- |

| 0.497710826 12275 |  | tags=30%,  list=22%,  signal=23% |  | EDAR/CHI3L1/TNFRSF21/SYK/C  CL18/TNFRSF12A/CRHBP/CCL14  /SGMS1/CCL20/PCK1/TNFSF9/  CCL13/GCH1/THBS1/CLIP3/TNFS  F18/CCL7/TNFRSF1B/TNFRSF11A  /CCL8/MYOD1/LCN2/TNFSF4/C  ARD16/TNFSF8/SPHK1/ADAMTS1  2/SELE/TNFRSF19/CASP1/CCL3  /CCL19/CLDN1/CCL23/CCL11/G  GT2/PTGS2/GPD1/MIR766/VCA  M1/TDGF1/PSMB9/CCL5/TNFRSF  4/GGT1/CCL3L3/LTB/CCL16/NP  NT/OCSTAMP/CALCA/CD40LG/  KCNJ11/BIRC3/TNFSF13B/TNFRS  F18/CCL21/GGT3P/CCL25/GBP3  /TNFRSF11B/GBP2/CCL24/TNFSF  15/CCL26/CCL4/GATA3/DCSTA  MP/ABCC2/ACOD1/CD27/TNFSF  14/GBP1/TNF/XCL1/CCL22/XCL  2/TNFRSF8/CD70/UBD/TCL1A/T  NFSF11/LTA/AIM2/ST18/TNFRS  F13B/CCL1/TNFRSF17/TNFRSF9/  MYOG |
| --- | --- | --- | --- | --- |

| 0.497710826 13330 |  | tags=44%,  list=24%,  signal=34% |  | G/KIT/CCDC88B/FCER1G/LGALS  3/RUNX3/LYN/PREX1/NRARP/AI  RE/LGALS9B/FOXJ1/JAG2/PELI1  /JAK3/MAFB/IGFBP2/TNFRSF21  /SYK/ARG2/LOXL3/CR1/SIRPB1  /ANXA1/LAT/AIF1/THY1/LGAL  S9/VSIG4/GPR183/TNFSF9/LFNG  /EFNB2/CD1D/HLA-  DPA1/NLRP3/PDCD1LG2/PIK3R6/  LEF1/RAC2/FZD7/CD80/LILRB4/  NLRC3/PLA2G2D/IGF2/RSAD2/H  LA-  DMB/ADAM8/IL6/CLEC4A/BCL3  /VSIR/EPO/PAX1/PRLR/TNFSF18  /CD1C/WAS/CGAS/TNFRSF1B/I  L7/LCP1/IFNB1/CLEC4E/RIPK3/D  PP4/TNFSF4/PLA2G2E/TNFSF8/H  AVCR2/LMO1/RUNX1/TNFAIP8L2  /CORO1A/CD86/RUNX2/IL1RL2/  JAML/PTPN6/CCL19/DOCK2/PL  A2G2A/LEPR/FOXP3/ICOSLG/ZE  B1/NCKAP1L/CLEC7A/VAV1/VC  AM1/SASH3/DUSP10/LILRB2/IFN  W1/CLC/CCL5/APBB1IP/CD300A  /HLA-  DOA/TNFRSF4/IL7R/IL18/CD209  /IFNE/EBI3/DLL4/HHLA2/CD28/  PRDM1/CD6/GLI2/IL10/CD160/F  GL2/CCR9/IFNL1/NFATC2/CLEC  4G/CD40LG/IGF1/PTGER4/IL1B/  TNFSF13B/IL2RA/TNFRSF18/PIK3  CG/NOD2/CCL21/EOMES/LILRB1  /IRF1/BATF/ZNF683/ITGAL/PTP |
| --- | --- | --- | --- | --- |

| 0.497710826 13668 |  | tags=35%,  list=24%,  signal=27% |  | /SLC16A3/SLC22A7/ATP2A3/A  BCC6/ATP6V0E2/TCIRG1/SLC5A  9/SLCO2A1/SLC2A10/SLC13A4/  SLC26A10/SLC4A11/ABCB4/SLC  47A2/ATP1B2/ABCC4/SLC9A3/  SLC26A9/SLC4A5/SLC17A5/ATP  2C2/SLC1A3/ABCA7/SLC24A4/  SLC22A4/ATP6V0A4/SLC16A13/  SLC47A1/ABCA6/SLC15A3/SLC6  A12/ATP1A3/SLC22A11/SLC2A6  /TAP1/SLC4A4/SLC16A9/SLCO2  B1/SLC18A1/SLC16A11/ATP6V1  C2/SLC6A9/SLC16A14/SLC16A8  /SLC6A13/ABCB9/SLC29A4/SLC  16A6/ATP6V0D2/SLC18A2/SLC6  A20/SLC45A3/SLC26A7/SLC26A  3/SLC15A2/SLC17A7/CDH17/SL  C45A1/ATP1A2/ABCC9/SLC6A3  /MFSD2A/SLC38A4/SLCO3A1/A  TP12A/SLC8A1/ABCC12/SLC5A4  /SLC6A1/SLC26A8/SLC34A2/A  BCC8/SLC7A11/SLCO5A1/SLC9C  2/SLCO1A2/SLC6A2/SLC1A1/SL  C38A3/SLC5A12/SLC9A7/ABCC  3/SLC34A1/SLC1A7/SLC36A2/S  LC28A3/ABCA4/SLC22A8/SLC25  A31/SLC12A3/ABCG4/SLC9A2/  SLC13A5/SLC4A8/SLC35D3/ABC  C2/ABCD2/ATP2B2/SLC5A7/SL  C5A8/ABCB11/FXYD2/SLC1A2/  SLC24A2/SLC22A6/SLC6A18/AB  CA13/SLC32A1/SLC8A3/SLC13A  2/SLC4A10/SLC17A8/SLC17A6/ |
| --- | --- | --- | --- | --- |

| 0.497710826 10462 |  | tags=49%,  list=19%,  signal=40% |  | 73/IL6/C8G/FCER2/BCL3/IGLV3  -  21/IL27RA/C1R/CD1C/CD19/W  AS/TNFRSF1B/IGKV3-  20/IGLC2/IFNB1/IGLV2-  8/IGHA1/RIPK3/HLA-  DQB1/TNFSF4/IGHM/INPP5D/GZ  MM/HAVCR2/IGHA2/C1S/IGHV4  -59/IGHV5-51/IGLV2-  14/IGHG2/IGHV1-  45/CD1E/IGHG1/IGHV4-  39/IGHE/CD1A/PTPN6/CCL19/IG  HV3-  23/IGKC/IL18RAP/FOXP3/IL18BP  /SASH3/IGLV2-23/CLC/IGHV3-  35/IGHV4-31/IGLV2-11/IGHV4-  34/IGKV3-15/IGLC7/IGHV3-  48/IL7R/IL18/EBI3/IGLV3-  19/IGKV1-5/CD28/IGHV3-  11/IGHV1-69D/APCS/IGHV3-  21/KLHL6/IL10/MYO1G/IGLV1-  44/IGHV1-3/IGHG3/IGHV4-  61/IGHV1-  18/IGLC6/FGL2/CLEC4G/C7/IGH  V3-30/IGHV2-70D/IGHV2-  5/CD40LG/POU2F2/IGLL5/IL1B/I  GLC3/C9/TNFSF13B/IGHG4/TRD  C/PRF1/CD1B/LILRB1/IGLV1-  40/IGHV1-  58/IGHD/BATF/TLR8/IGHV4-  28/IGKV1-17/IGHV1-24/IGHV3-  33/PTPRC/IL27/IGKV3D-  11/CCR2/LY9/IGHV2- |
| --- | --- | --- | --- | --- |

| 0.497710826 15858 |  | tags=30%,  list=28%,  signal=22% |  | ATP2B1/TP63/NUAK1/GLRX2/PP  P3CA/CDKN2A/CD68/WNT16/ID  E/TBX3/ICAM1/CX3CL1/STAT3  /B2M/PRKDC/ALDH3A1/MMP7/L  RP1/CTSC/PAWR/GJB2/TFCP2L1  /ENO3/NOX4/RAD54L/C1QA/TG  FB3/ASS1/ECRG4/COMP/HLA-  G/ITGB2/KL/SLC30A10/CYP1A1  /HAMP/CDKN1A/PDGFRB/IGFBP2  /ARG2/AGT/LOXL2/COL4A2/PL  A2R1/PCK1/HMGA2/VASH1/KCN  E2/NQO1/P2RY1/EPO/PENK/DKK  1/CGAS/NPY5R/KMO/TNFRSF1B  /RGN/SERPINE1/INPP5D/IGFBP1/  KCNMB1/SLC6A3/BCL2A1/CLDN  1/CCL11/PTGS2/VCAM1/TWIST  1/TRPC6/MAGEA2/IL10/CALCA  /PITX3/NTRK1/PAX2/ATP8A2/H  TR2A/ADRA1A/FOXG1/IL15/PA  X5/KRT16/RNF165/KIR2DL4/SLC  1A2/WNT1/DDC/TACR3/SLC32  A1/GRM5/NPY2R/KRT83/KRT33B  /MIR543 |
| --- | --- | --- | --- | --- |

| 0.497710826 13536 |  | tags=32%,  list=24%,  signal=24% |  | ACACB/HLA-  DRA/FOLH1/LVRN/HLA-  G/ITGB2/GUCY2C/CALCRL/HLA  -H/GALR2/HLA-  DRB1/MGST1/FOLR3/ERAP2/ECE  1/SLC46A1/INHBA/VIPR1/PPIAL4  D/ACHE/CEMIP/CRHBP/NPR1/AT  P1A3/CD1D/HLA-DPA1/HLA-  DRB5/TAP1/HLA-  F/PEX5L/AVPR1A/NPBWR1/MSR  1/CLIP3/GSTM1/PRLR/NGFR/PPI  AL4G/CRHR2/BDKRB1/CD1C/TM  EM158/HCRTR1/TGFB2/GCGR/PPI  AL4C/HLA-  DQB1/OXTR/ENPEP/EPHA4/CD1E  /PCSK5/SSTR1/CD300LF/CD1A/  CRHR1/SORL1/LEPR/HLA-  DQA1/CMA1/MCHR1/GUCY2D/G  PR37L1/LILRB2/ANPEP/CCKAR/G  LP2R/CD209/CABP1/NPR3/GALR  1/FOLR1/GRIA1/ITGA2/EPHB2/S  STR2/NOD2/CCKBR/CD1B/NMUR  2/TRHDE/FPR2/ADCYAP1R1/KPN  A7/PPIAL4H/CRIP1/SCTR/LBP/G  PR149/OPRD1/TRAV8-  4/GRIA4/TRBV7-  9/GRIN1/TRGV9/TRAV29DV5/GR  IN2A/TRGV3/CALCR/GRIA3/MC4  R/HCRTR2/OPRM1/TRAV19/PTH2  R/SSTR3/GRIA2/GUCY2F/TRBV1  2-  3/MRGPRX2/SSTR5/GHSR/MC3R |
| --- | --- | --- | --- | --- |

| 0.497710826 14185 |  | tags=43%,  list=25%,  signal=32% |  | OLN2/RASA3/PANX1/STAC2/ME  TTL21C/P2RX4/GPR35/ATP2A3/  GNB5/ANK2/PDE2A/LYN/CACNB  2/TRPV4/CACNA1C/GJC2/CATS  PER1/GRIN2C/TRPM4/FGF14/CA  CNA1H/CATSPER3/CACNA1F/PL  CL2/ATP2C2/CACNB4/ASIC1/SL  C24A4/CASQ2/THY1/CEMIP/TRP  V2/CACNA2D1/F2R/NTSR1/CRA  CR2A/KCNE2/RGS9/CACNG1/HR  C/PLCD4/DYSF/MCUB/TRPM5/D  RD2/TMEM37/PKD1L3/BDKRB1/C  D19/JPH1/CACNB1/REM1/GRIN2  D/NOS1AP/F2RL3/GRIN3A/ADRA  2A/RGN/STRIT1/PKD2L1/CACNG  5/APLNR/CASQ1/ATP1A2/CORO  1A/TRPM2/PTPN6/CRHR1/CCL3/  CCL19/JSRP1/STAC/RRAD/CAC  NA1E/SLC8A1/FAM155A/TRPM8  /CACNA2D3/GEM/GPM6A/TRPC  6/IL13/CAV3/CACNA2D4/ITPR2  /TRPM3/CLIC2/HPCA/TRPV6/PL  CH2/PIK3CG/CCL21/P2RY6/CAL  HM1/PLCH1/HTR2A/PTPRC/CCR7  /HAP1/TRPV3/CACNG4/CCR5/P  LN/CXCL10/ATP2B2/CXCL11/CH  RNA9/HTR2B/CACNG3/TRPC3/N  OS1/LCK/XCL1/BHLHA15/CXCL  9/RYR2/MRLN/CACNA1S/CACN  G6/CACNG7/TRDN/SLC24A2/DR  D3/GRIN1/CACNA1G/GRIN2A/C  ALCR/TMC1/CACNA1I/DRD1/OP  RM1/SLC8A3/FASLG/XCR1/TRP |
| --- | --- | --- | --- | --- |

| 0.497710826 13719 |  | tags=25%,  list=24%,  signal=19% |  | EGFR/PPEF1/OPN3/KCNK4/RELB/  IFI16/FAS/PDE2A/TRPV4/MFAP4  /AQP10/GJA1/SFRP2/COL1A1/  HAMP/CDKN1A/AGT/ASIC1/RPE  65/MC1R/TSPYL5/PCK1/RHO/H  MGA2/SCX/AVPR1A/GNAT1/DY  SF/EPO/PKD1L3/RRH/GUCA1A/  AQP1/RGR/NFATC4/MAPK10/PT  AFR/PKD2L1/CARD16/BDKRB2/R  CSD1/ATP1A2/TNFRSF10A/TLR7  /CASP1/AIPL1/PTGS2/GUCY2D/  GPR88/GPR68/OPN1LW/IL13/GR  M1/SLC38A3/HPCA/PTGER4/IL1  B/ITGA2/SAG/PDE6A/KCNK18/I  RF1/TLR8/GNGT1/OPN4/GATA3  /CNGA1/CRIP1/GRK1/CNGB1/G  UCA1C/TNFSF14/GRK7/TNFRSF8  /RP1/OPN5/NEUROD2/SCN2A/C  ASP5/GUCY2F/OPN1MW/MYOG |
| --- | --- | --- | --- | --- |

| 0.497710826 12430 |  | tags=38%,  list=22%,  signal=30% |  | ISG15/IL1RL1/CHI3L1/TNFRSF21/  FGR/SYK/ARG2/AGT/ANXA1/C  SF1R/CRLF2/AIF1/LGALS9/F2R/  NLRP3/OSM/ORM1/C5AR2/FN1/  IL1A/FFAR4/DYSF/WNT5A/DRD  2/IL27RA/NLRC4/USP50/CD244/  LILRA5/TNFRSF1B/CLEC4E/CARD  16/HAVCR2/NOX5/SYT11/PANX  2/CASP1/CCL3/CCL19/SORL1/F  OXP3/MIR766/CHIA/FFAR2/TWI  ST1/TNFRSF4/LCP2/IL10/CD160  /PTGER4/IL1B/CADM1/NOD2/LIL  RB1/IGHD/TLR8/CCR7/CLECL1/I  L1R2/CARD11/PAEP/GATA3/CD  200R1/IL26/CD2/HTR2B/BANK1/  GBP1/TNF/CRP/NLRP7/RASGRP1  /PTPN22/ORM2/APOA2/CLEC9A  /GBP5/MMP12/TLR10/AIM2/AG  TR2/CRTAM/HMGB4/CCL1/MMP  8/IFNG/CARD17/GHSR/IL17F/C  ARD18/NLRP10/MIR204/MIR182 |
| --- | --- | --- | --- | --- |

| 0.497710826 10395 |  | tags=45%,  list=18%,  signal=37% |  | 73/IL6/CEBPE/LYG1/BCL3/SIGLE  C16/H2BS1/IL27RA/NLRC4/SPAG  11A/HTN3/IGKV3-  20/HTN1/IGLC2/SERPINE1/STAB  2/CLEC4E/IGHA1/LCN2/PRG2/IG  HM/FGB/TNFSF8/HAVCR2/IGHA2  /H2BC10/IGHV4-59/IGHV5-  51/LYZL2/IGHG2/SYT11/IGHV1-  45/IGHG1/IGHV4-  39/IGHE/GNLY/DMBT1/PGLYRP3  /PGC/ADAMTS5/IGHV3-  23/PLA2G2A/IGKC/DEFB123/PRB  3/IGHV3-35/IGHV4-31/IGHV4-  34/SLAMF8/REG3G/KRT6A/IGLC  7/IGHV3-48/IFNE/IGHV3-  11/IGHV1-69D/IGHV3-  21/IL10/CD160/SIGLEC11/DEFB1  /IGHV1-3/IRF8/IGHG3/IGHV4-  61/GBP4/IGHV1-  18/IGLC6/JCHAIN/CTSG/IGHV3  -30/IGHV2-70D/IGHV2-  5/IGLL5/IGLC3/IGHG4/NOD2/TR  DC/C10orf99/IGHV1-  58/GBP2/IGHD/LYZL1/VGF/PLA  C8/IGHV4-28/IGHV1-  24/IGHV3-33/FPR2/IGHV2-  70/DCD/DEFB119/IL23R/IGHV3-  7/TRBC2/IL22RA1/GBP7/IGHV3-  20/LBP/SPN/IGHV3-74/IGHV3-  13/IGHV4-4/TNF/CRP/IGHV3-  66/FCN2/IGHV3-72/IGHV1-  69/GALP/SPINK5/WFDC12/DEFB  134/GBP6/AICDA/S100A7/IGHV |
| --- | --- | --- | --- | --- |

| 0.497710826 11652 |  | tags=33%,  list=21%,  signal=26% |  | CTTNBP2/ADAM23/CLSTN2/DLG  2/SEMA3F/CNKSR2/EFNB2/NRGN  /GRIK5/P2RX6/UNC13A/RGS9/S  HANK1/CAMK1/IL1RAPL2/HOMER  1/GUCY1A1/SH3GL2/P2RY1/WN  T5A/DRD2/SPARCL1/DNM3/NOS  1AP/NPTX2/GRIN3A/ADAM22/M  APK10/CACNG5/EPHA4/NLGN4Y  /GPC6/LRRC4/GRID1/CORO1A/R  GS7BP/NLGN4X/PPP1R1B/CPT1C  /PCDH17/SLITRK2/GSG1L/NRG1  /GPM6A/GABRD/ARC/GRM3/GR  M1/CHRM3/PLG/HPCA/SHISA9/  GPC4/LRFN5/GRIA1/CADPS/EPH  B2/PTPRO/PRRT2/ITGB3/NTNG2/  ADCY8/LRRTM1/ACAN/LRRC4C/  NTRK3/HTR2A/SYT1/CALB1/AD  RA1A/P2RX1/CACNG4/CALY/SH  ISA6/PSD2/ADCY1/PLPPR4/SH3  GL3/SYT4/NETO1/SYT6/PTPRD/  ATP2B2/NRXN1/ADGRL3/KCNA3  /EPHA7/CACNG3/SLITRK4/PCDH  8/CAMKV/SLC1A2/LRRTM2/SLIT  RK1/CACNG7/CPLX2/DRD3/GRIN  1/DGKB/TNR/GRID2/GRIN2A/AC  TC1/NPTX1/FRMPD4/CBLN2/DRD  1/ADGRA1/PPFIA2/SCN2A/KCN  D2/HTR3A/CACNG2/LRRTM3/KC  NA1/CHRM2/GHSR |
| --- | --- | --- | --- | --- |

| 0.497710826 10813 |  | tags=53%,  list=19%,  signal=43% |  | N3/C3/IGHV3-  73/IL6/C8G/FCER2/BCL3/IGLV3  -  21/H2BS1/C1R/SPAG11A/CD19/  HTN3/IGKV3-  20/IL7/HTN1/IGLC2/PRSS3/IFNB  1/IGLV2-8/IGHA1/LCN2/HLA-  DQB1/IGHM/PI3/FGB/SPNS2/IGH  A2/C1S/CFB/H2BC10/IGHV4-  59/IGHV5-51/IGLV2-  14/IGHG2/IGHV1-  45/IGHG1/IGHV4-  39/IGHE/ITLN1/GNLY/PTPN6/DM  BT1/PGLYRP3/PGC/IGHV3-  23/PLA2G2A/IGKC/VTN/BLNK/I  GLV2-23/IFNW1/IGHV3-  35/IGHV4-31/IGLV2-11/IGHV4-  34/IGKV3-  15/REG3G/KRT6A/IGLC7/IGHV3  -48/IFNE/EBI3/IGLV3-  19/IGKV1-5/CD28/IGHV3-  11/IGHV1-69D/APCS/IGHV3-  21/DEFB1/IGLV1-44/IGHV1-  3/IGHG3/IGHV4-61/IGHV1-  18/IGLC6/JCHAIN/C7/CTSG/IGH  V3-30/IGHV2-70D/IGHV2-  5/POU2F2/IGLL5/IL1B/IGLC3/C9  /IGHG4/CR1L/MNX1/TRDC/IGLV  1-40/IGHV1-58/IGHD/IGHV4-  28/IGKV1-17/IGHV1-24/IGHV3-  33/PTPRC/CCR7/IGKV3D-  11/CCR2/IGHV2-  70/DCD/PAX5/GATA3/IGHV3- |
| --- | --- | --- | --- | --- |

| 0.497710826 13330 |  | tags=44%,  list=24%,  signal=34% |  | G/KIT/FCER1G/RUNX3/PREX1/N  RARP/AIRE/FOXJ1/JAG2/SPI1/J  AK3/MAFB/INHBA/SYK/LOXL3/  CR1/PLCL2/ANXA1/TPD52/ADG  RG3/LGALS9/CD79B/GPR183/TN  FSF9/LFNG/CD1D/NLRP3/PIK3R6  /LEF1/FZD7/CD80/LILRB4/DOCK  11/PLA2G2D/RSAD2/ADAM8/IL6  /BCL3/VSIR/PAX1/MFNG/CD19  /IL7/IFNB1/CLEC4E/RIPK3/TNFS  F4/INPP5D/TNFSF8/NFAM1/CDH  17/RUNX1/CD86/RUNX2/IL1RL2  /DOCK10/PTPN6/PGLYRP3/CCL1  9/DOCK2/LEPR/FOXP3/ONECUT1  /CD79A/ZEB1/NCKAP1L/BLNK/V  AV1/VCAM1/SASH3/DUSP10/LI  LRB2/IFNW1/IL11/HLA-  DOA/SLAMF8/IL7R/IL18/IFNE/D  LL4/CD28/PRDM1/GLI2/IL10/FG  L2/CCR9/IFNL1/NFATC2/IKZF1/  CD40LG/POU2F2/PTGER4/IL2RA/  TNFRSF18/EOMES/NTRK1/IRF1/B  ATF/ZNF683/PTPRC/CCR7/IL27/  CCR2/LY9/CARD11/IL15/MYB/G  ATA3/IL23R/FLT3/CD3E/CD2/S  HH/IL12RB1/CD27/SPN/TBX21/C  D3D/ITGA4/LCK/ZAP70/SLAMF6  /RASGRP1/PTPN22/WNT1/CD8A  /FUT7/TOX/SPINK5/GPR18/AIC  DA/IKZF3/LAG3/RHOH/ITK/TESP  A1/LY6D/BCL11B/IL12B/PGLYRP  2/CCR6/CD3G/MS4A1/THEMIS/  CTLA4/CR2/FCRL3/IFNG/IFNA10 |
| --- | --- | --- | --- | --- |

| 0.497710826 10462 |  | tags=52%,  list=19%,  signal=43% |  | 73/IL6/C8G/FCER2/BCL3/IGLV3  -  21/IL27RA/C1R/CD1C/CD19/W  AS/TNFRSF1B/IGKV3-  20/IGLC2/IFNB1/IGLV2-  8/IGHA1/RIPK3/HLA-  DQB1/TNFSF4/IGHM/INPP5D/GZ  MM/HAVCR2/IGHA2/C1S/IGHV4  -59/IGHV5-51/IGLV2-  14/IGHG2/IGHV1-  45/CD1E/CORO1A/IGHG1/IGHV4  -  39/IGHE/CD1A/PTPN6/SH2D1B/I  GHV3-  23/SLAMF7/IGKC/IL18RAP/FOXP  3/VAV1/SASH3/IGLV2-  23/CLC/IGHV3-35/IGHV4-  31/IGLV2-11/IGHV4-34/IGKV3-  15/IGLC7/IGHV3-  48/IL7R/IL18/IGLV3-19/IGKV1-  5/CD28/IGHV3-11/IGHV1-  69D/APCS/IGHV3-  21/ULBP1/IL10/MYO1G/CD160/I  GLV1-44/IGHV1-  3/IGHG3/IGHV4-61/IGHV1-  18/IGLC6/FGL2/CLEC4G/C7/IGH  V3-30/IGHV2-70D/IGHV2-  5/CD40LG/POU2F2/IGLL5/IL1B/I  GLC3/C9/CADM1/IGHG4/TRDC/  PRF1/CD1B/LILRB1/IGLV1-  40/KIR3DL1/IGHV1-  58/IGHD/BATF/TLR8/IGHV4-  28/IGKV1-17/IGHV1-24/IGHV3- |
| --- | --- | --- | --- | --- |

| 0.497710826 14601 |  | tags=36%,  list=26%,  signal=27% |  | CD14/TNFRSF1A/GPC1/CASP8/I  QGAP1/UNC5B/AKAP6/EHD2/AT  P1B1/CD24/PRKAR2B/EMP2/CDH  13/BTK/EGFR/SLC38A9/CD4/HM  OX1/MYOF/ANGPT1/ANK2/FAS  /ITGB2/LYN/CDH1/RET/S1PR1/  ABCB4/RTN4RL1/GJA1/CAVIN3/  PECAM1/KDR/ADGRG1/SULF1/L  AT/THY1/BST2/F2R/ADD2/MAP  T/ITGA1/NTSR1/CD177/KCNMA  1/KCNE1/NPHS2/FYB2/LRP8/RFT  N1/DYSF/CLIP3/SELPLG/SYNJ2/  CD19/TNFRSF1B/NOS1AP/HCK/D  PP4/INPP5D/CHRNA3/ATP1A2/L  AT2/ADTRP/CDH2/SLC6A3/SELE  /ITLN1/ADRA1B/CD1A/CD79A/  PTGS2/FAIM2/TDGF1/TRPM8/CD  48/ARC/LCP2/CAV3/BAALC/LY  6K/SLC6A2/UNC5A/STOML3/HP  SE/BIRC3/SLC34A1/ADCY8/HTR  2A/MLC1/AKAP5/PTPRC/ADRA1  A/P2RX1/ADCYAP1R1/CARD11/  ADCY1/CD2/SHH/ADCY2/OPRD1  /SKAP1/KCNA3/NOS1/TNF/LCK  /ZAP70/MALL/CD8A/MAL2/SL  C22A6/TNR/OPRM1/RIT2/FASLG  /MS4A1/KCND2/FAM170B/GHSR |
| --- | --- | --- | --- | --- |

| 0.497710826 13675 |  | tags=38%,  list=24%,  signal=29% |  | 3C2A/COMP/ANK2/KIT/TNNI3K/  TNNC2/CACNB2/CALCRL/LMOD2  /TNNI2/CACNA1C/GALR2/NKX2  -  5/GJA1/KLHL41/PPP1R12B/TRPM  4/STC1/CACNA1H/ARG2/AGT/  PTGER3/SULF1/MYBPH/CASQ2/  ACTG2/KCND3/MYOCD/CACNA2  D1/CHGA/F2R/ITGA1/HTR7/P2R  X2/MYBPC3/P2RX6/KCNMA1/KC  NE1/KCNE2/TBX20/ADORA1/GJ  C1/MYL10/TNNC1/SCN4A/HOME  R1/LMOD1/GUCY1A1/HRC/DYSF  /MYLK2/FGF12/DRD2/SULF2/DE  S/EDN2/NOS1AP/ADRA2B/ADRA  2A/SCN1B/KCNJ8/PTAFR/STRIT  1/SCN4B/OXTR/SPHK1/MYH11/  CHRNA3/CASQ1/BDKRB2/RCSD1  /ATP1A2/SCN2B/LMOD3/ACTA1  /ADRA1B/JSRP1/STAC/KCNE1B  /PTGS2/SLC8A1/DOCK4/CAV3/  KCNH2/CHRM3/CLIC2/NPNT/CAL  CA/HCN4/HTR1D/ITGA2/TNNT2  /SSTR2/PIK3CG/NMUR2/GJA5/  ANKRD2/SCN7A/ATP8A2/HTR2A  /TACR1/ADRA1A/P2RX1/UTS2/  MYBPC1/CD38/PLN/MYL4/HTR2B  /KCNJ3/NOS1/RYR2/MB/CACN  A1S/KCNB2/TRDN/CACNA1G/A  CTC1/MYOM3/DRD1/SCN1A/TA  CR3/TIFAB/SLC8A3/NEUROG1/G  LRA1/KCNA1/CHRM2/NPY2R/GH  SR/CSRP3/DCANP1/MIR153-1 |
| --- | --- | --- | --- | --- |

| 0.497710826 13703 |  | tags=31%,  list=24%,  signal=24% |  | TGFB1/TRIB1/HMOX1/ANGPT4/N  BL1/SRGAP2/SEMA3A/CLDN5/C  DH1/GPR173/TIE1/DACH1/GJA1  /SFRP2/HRG/CXCL12/SRGAP1/S  TC1/GREM1/DPYSL3/JAG1/ANG  PT2/CX3CR1/ADGRG1/SULF1/MI  R2355/IFITM1/AIF1/THY1/PTPRU  /BST2/NKX2-  1/ADGRB1/MYOCD/TBX5/MIR26  A2/HAS1/CYP19A1/VASH1/AD  ORA1/C5AR2/SLIT2/TMIGD3/CH  RD/MMP28/THBS1/BMERB1/SRG  AP2C/KLF4/DRD2/EPHA1/NOG/I  L27RA/CCN3/WAS/RGN/SERPINE  1/MIR212/TACSTD2/SRGAP2B/A  DTRP/ADAMTS9/SLURP1/MIR193  A/NRG1/DUSP10/TMEFF2/NDRG4  /CD300A/SLAMF8/DLL4/PTPRR/  MARVELD3/PTGER4/CCL21/CCL2  5/MIR503/GATA3/CD200R1/GCS  AM/SHH/KRT16/STAP1/FRMD5/  DPEP1/GPR18/AGTR2/PTPRT/KLR  C4-  KLRK1/SEMG2/SPOCK3/CXCL13/  KLRK1/SEMG1/MIR204/MIR129-  2/MIR138-2/MIR218-  1/MIR200B/MIR182 |
| --- | --- | --- | --- | --- |

| 0.497710826 11951 |  | tags=34%,  list=21%,  signal=27% |  | SLC16A3/P2RX4/SRGAP2/DLG4/  CTNND2/LYN/MX1/CACNA1C/D  NAJC6/MAP2/GRIN2C/NEURL1/T  NIK/CLSTN3/SIPA1L1/FABP5/SY  T12/MT3/ADGRB1/CLSTN2/DLG  2/DLGAP1/ADD2/CNKSR2/EFNB2  /NRGN/GRIK5/NTSR1/SPOCK1/G  NG3/P2RX6/LRRC7/SAMD14/AD  ORA1/CAMK2A/RGS9/SHANK1/  CAMK1/LRP8/HOMER1/NEFH/SL  C6A9/SH3GL2/P2RY1/HCLS1/DR  D2/MX2/USP50/DNM3/PENK/GRI  N2D/GRIN3A/ADAM22/CACNG5/  EPHA4/DAB1/NLGN4Y/CHRNA3/  SYNDIG1/SRCIN1/SYT11/LRRC4/  GRID1/MAP1B/CDH2/RGS7BP/IQ  SEC3/NLGN4X/GSG1L/LRFN2/AR  C/SH2D5/BAALC/GRM3/CABP1/  GRM1/CHRM3/SHISA9/LRFN5/GR  IA1/SHISA8/PTPRO/PRRT2/ADC  Y8/INSYN2A/LRRC4C/AKAP5/SY  T1/CALB1/CACNG4/SHISA6/PRK  CG/ADCY1/PLPPR4/SH3GL3/NET  O1/ATP2B2/GRIK1/OPRD1/SYT9  /EPHA7/CACNG3/NOS1/GRIA4/  LRRTM2/SLITRK1/CACNG7/DRD3  /GRIN1/GRID2/GRIN2A/GRIA3/F  RMPD4/GRM7/ADGRA1/GRM5/K  CND2/GRIA2/SLITRK3/CACNG2/  LRRTM3/CHRM2 |
| --- | --- | --- | --- | --- |

| 0.497710826 12470 |  | tags=49%,  list=22%,  signal=38% |  | PECAM1/FGR/SYK/UNC13D/SIRP  B1/ANXA1/C2/ABCA7/AIF1/AD  GRB1/PLD4/IGLV1-  47/AHSG/IGKV4-  1/RAC2/IGLV6-  57/NCF4/CD93/ADORA1/ARHGA  P25/FCN3/C3/IGHV3-  73/THBS1/MSR1/DYSF/TICAM2  /CEBPE/IGLV3-  21/CYFIP2/XKR5/WAS/IGKV3-  20/IGLC2/HCK/IGLV2-  8/IGHA1/ELMO1/IGHM/SPHK1/I  GHA2/CEACAM4/IGHV4-  59/RAB31/IGHV5-51/IGLV2-  14/IGHG2/SYT11/IGHV1-  45/CORO1A/IGHG1/TULP1/IGHV  4-39/IGHE/CD300LF/IGHV3-  23/DOCK2/IGKC/LEPR/NCKAP1L  /CLEC7A/VAV1/ALOX15/IGLV2  -23/IGHV3-  35/CAMK1D/CD300A/IGHV4-  31/IGLV2-11/IGHV4-34/IGKV3-  15/RAB7B/IGLC7/IGHV3-  48/IGLV3-19/IGKV1-5/IGHV3-  11/IGHV1-69D/IGHV3-  21/MYO1G/IL2RG/IGLV1-  44/IGHV1-  3/IRF8/FCGR3A/IGHG3/IGHV4-  61/IGHV1-18/IGLC6/IGHV3-  30/IGHV2-70D/IGHV2-  5/IGLL5/BIN2/IL1B/IGLC3/ITGA2  /IGHG4/IL15RA/TRDC/ITGB3/FC  GR1A/IGLV1-40/IL2RB/IGHV1- |
| --- | --- | --- | --- | --- |

| 0.497710826 12508 |  | tags=35%,  list=22%,  signal=28% |  | GRIN2C/FGF14/NEURL1/AGT/CX  3CR1/SIPA1L1/SYT12/ADGRB1/  HRH1/MAPT/NRGN/FAM107A/UN  C13A/ADORA1/CAMK2A/SHANK  1/HRH2/DRD2/BRSK1/CRHR2/GRI  N2D/NFATC4/EPHA4/MAP1B/PT  GS2/LILRB2/ARC/ITPKA/MAP1A  /RELN/SHISA9/KCNJ10/GRIA1/S  HISA8/CALB2/EPHB2/PRRT2/AD  CY8/LRRTM1/CALB1/SHISA6/CD  38/ADCY1/SYT4/RASGRF1/NET  O1/CNTN2/SCTR/DRD5/SCGN/L  RRTM2/CRH/SLC24A2/CPLX2/GR  IN1/TNR/GRID2/GRIN2A/DRD1/N  EUROD2/RIMS2/SLC8A3/RIMS1/  SLC4A10/GRM5/UNC13C |
| --- | --- | --- | --- | --- |

| 0.497710826 12216 |  | tags=44%,  list=22%,  signal=34% |  | YK/ARG2/LOXL3/CR1/SIRPB1/A  NXA1/LAT/AIF1/THY1/LGALS9  /VSIG4/TNFSF9/EFNB2/CD1D/HL  A-  DPA1/NLRP3/PDCD1LG2/PIK3R6/  RAC2/CD80/LILRB4/PLA2G2D/IG  F2/HLA-  DMB/ADAM8/IL6/VSIR/EPO/TNF  SF18/CGAS/TNFRSF1B/IL7/IFNB1  /RIPK3/DPP4/TNFSF4/PLA2G2E/  TNFSF8/HAVCR2/LMO1/RUNX1/  TNFAIP8L2/CORO1A/CD86/IL1RL  2/PTPN6/CCL19/PLA2G2A/FOXP  3/ICOSLG/ZEB1/NCKAP1L/VAV1  /VCAM1/SASH3/DUSP10/LILRB2  /CLC/CCL5/CD300A/HLA-  DOA/IL7R/IL18/CD209/EBI3/HHL  A2/CD28/PRDM1/CD6/GLI2/IL10  /CD160/FGL2/IFNL1/NFATC2/C  LEC4G/CD40LG/IGF1/IL1B/TNFSF  13B/IL2RA/TNFRSF18/NOD2/CCL  21/LILRB1/IRF1/ZNF683/PTPRC/  CCR7/IL27/CLECL1/RASAL3/CC  R2/CARD11/IL15/MYB/CD5/GA  TA3/IL23R/CD3E/CD2/SHH/IL12  RB1/CD27/SPN/TBX21/GRAP2/T  NFSF14/LCK/ZAP70/XCL1/RASG  RP1/SIT1/PTPN22/CD70/SPINK5/  LAG3/TNFSF11/RHOH/TESPA1/IL  12B/IDO1/BTLA/KLRC4-  KLRK1/PDCD1/LAX1/SIRPG/TIGI  T/KLRK1/ICOS/CTLA4/IL21/IFN  G/IL2 |
| --- | --- | --- | --- | --- |

| 0.497710826 12392 |  | tags=26%,  list=22%,  signal=21% |  | CDKN1A/ELANE/MEIS2/CACNA1  F/RCVRN/SLC1A3/RPE65/MC1R/  AANAT/HRH1/EYS/CCK/RHO/HR  H2/GNAT1/BCL3/DRD2/BRSK1/P  DE1B/RRH/GUCA1A/AQP1/RGR/  LRRN4/NFATC4/MAPK10/CARD1  6/SEMA5B/PDE6C/ATP1A2/TULP  1/RGS9BP/PPP1R1B/AIPL1/PTGS2  /GUCY2D/GPR88/OPN1LW/NDRG  4/ELOVL4/SERPINB13/ADAM2/S  LC7A11/KCNC1/CACNA2D4/HOX  A1/GNGT2/SAG/PDE6A/GRM6/I  VL/ATP8A2/ABCA4/PDC/GNGT1  /OPN4/NETO1/CNGA1/CRIP1/GR  K1/CNGB1/GUCA1C/NR2E3/TRP  C3/GRK7/SLC1A2/CABP4/FOXB  1/DRD3/GRIN1/RP1/GRIN2A/OPN  5/ASIC2/IL12B/DRD1/GJA10/SL  C4A10/GUCY2F/GNAT2/KCNC2/  OPN1MW |
| --- | --- | --- | --- | --- |

| 0.497710826 12676 |  | tags=34%,  list=22%,  signal=27% |  | GJA1/MGST1/THBD/SPON2/HAM  P/PELI1/ELANE/CYP1A2/IRAK3/  CX3CR1/LY96/PRKCA/FMO1/LG  ALS9/LY86/F2R/SGMS1/PCK1/N  LRP3/PDCD1LG2/CARD9/TIMP4/  CD80/CMPK2/NOCT/PTGER1/GC  H1/IL1A/IL6/IRF5/TICAM2/CEBP  E/IL1RN/EPO/WNT5A/PENK/BDK  RB1/KMO/TNFRSF1B/TNFRSF11A  /SERPINE1/HCK/PTGFR/MAOB/F  GFR2/LCN2/PTAFR/TNFSF4/CAR  D16/PTGIR/HAVCR2/CD180/CD8  6/SELE/CASP1/CCL3/CLDN1/GG  T2/IL10RA/PTGS2/MIR766/VCA  M1/SCIMP/VDR/DUSP10/LILRB2/  CCL5/CSF2RB/IL13/IL18/CD6/G  GT1/IL10/IRF8/CTSG/PTGER4/IL  1B/NOD2/GGT3P/LILRB1/CCR7/  CCR5/IL23R/PALM3/CXCL10/AB  CC2/ACOD1/LBP/CXCL11/C4B/  STAP1/TAC1/IL37/NOS1/FGF10  /TNF/NLRP7/GFI1/CXCL9/PTPN2  2/CSF2/NUGGC/CD96/CNR2/AI  CDA/S100A7/LTA/IL12B/IDO1/  OPRM1/KLRC4-  KLRK1/IL36G/FASLG/CXCL13/KL  RK1/TNIP3/GPR31/CARD17/GHSR  /DEFA4 |
| --- | --- | --- | --- | --- |

| 0.497710826 13673 |  | tags=30%,  list=24%,  signal=23% |  | IFNAR2/PIM2/APOBEC3G/APOBE  C3D/MIR26B/IFI16/OAS1/IRF7/  MX1/STAT1/CYP1A1/SERINC5/  CXCL12/SPON2/ISG15/OAS2/FG  R/UNC13D/IRAK3/DDX60/IFI44L  /HTRA1/IFITM1/PARP9/LGALS9/  BST2/VWCE/TRIM22/NLRP3/CAR  D9/HMGA2/RNASE6/FCN3/RSAD  2/IL6/IRF5/BCL3/OASL/FOSL1/  MX2/PENK/CGAS/IRF9/RNASE2/  IFNB1/CCL8/LCN2/SLFN11/TNFS  F4/MIR30C1/ABCC9/TLR7/DMBT  1/CCL19/IFI27/RNF125/FOXP3/  CCL11/APOBEC3H/IFNW1/CCL5/  IFNE/BATF3/NLRC5/FGL2/IFNL1/  IL1B/BIRC3/IL2RA/PRF1/GBP3/LI  LRB1/IRF1/TLR8/ISG20/PTPRC/IL  27/CCL4/IL15/GATA3/IL23R/BT  BD17/CXCL10/IL12RB1/TBX21/G  BP1/TNF/XCL1/CXCL9/CCL22/  AICDA/IL12B/MMP12/AIM2/IFN  G/CHRM2/IFNA10/IFNA21 |
| --- | --- | --- | --- | --- |

| 0.497710826 12660 |  | tags=54%,  list=22%,  signal=42% |  | LRRC32/PDE5A/IL20RB/BTN3A1/  GLMN/HLA-  DPB1/PAWR/ZP3/MAD1L1/CD24  /CD274/PSMB10/CD4/HLA-  G/CCDC88B/LGALS3/LGALS9B/F  OXJ1/PELI1/JAK3/IGFBP2/TNFRS  F21/SYK/ARG2/ANXA1/AIF1/L  GALS9/VSIG4/TNFSF9/CD1D/HL  A-  DPA1/PDCD1LG2/RAC2/CD80/PL  A2G2D/IGF2/HLA-  DMB/IL6/VSIR/EPO/TNFSF18/TN  FRSF1B/RIPK3/TNFSF4/PLA2G2E/  TNFSF8/HAVCR2/LMO1/CORO1A  /CD86/PTPN6/CCL19/DOCK2/PL  A2G2A/FOXP3/ICOSLG/NCKAP1L  /VCAM1/SASH3/LILRB2/CLC/C  CL5/TNFRSF4/IL18/CD209/EBI3/  HHLA2/CD28/CD6/IL10/CLEC4G  /CD40LG/IGF1/IL1B/TNFSF13B/I  L2RA/PIK3CG/LILRB1/IRF1/PTPR  C/IL27/CLECL1/RASAL3/CCR2/  CARD11/IL15/IL23R/CD3E/SHH/I  L12RB1/SPN/TNFSF14/ZAP70/X  CL1/CD70/IL12B/IDO1/CTLA4/I  L21/IL2 |
| --- | --- | --- | --- | --- |

| 0.497710826 12243 |  | tags=46%,  list=22%,  signal=37% |  | SPER3/CLIC6/CACNA1F/CACNB4  /CASQ2/VWC2/KCND3/SCNN1G  /ANO1/ATP1A3/CACNA2D1/DL  G2/GABRG3/KCNN4/KCNAB1/KC  NJ1/GRIK5/LRRC7/GABRB3/KCN  MA1/ANO2/KCNE1/KCNE2/CAC  NG1/SHANK1/KCNK6/KCNMB4/S  CN4A/GABRQ/HCN2/MCUB/PKD  1L3/KCNF1/CACNB1/GABRA5/G  RIN2D/NOS1AP/GRIN3A/KCNQ3/  SCN1B/KCNJ8/KCNV1/LRRC38/K  CNV2/CLCNKB/PKD2L1/CACNG5  /SCN4B/CHRNA3/CLCNKA/KCNA  2/KCNMB1/ATP1A2/ABCC9/SCN  2B/KCNK1/SCN9A/CPT1C/ATP1  2A/KCNE1B/SCNN1B/SCNN1A/C  ACNA1E/CLDN4/GABRA4/CACN  A2D3/ABCC8/TRPC6/GABRD/KC  NC1/CLIC3/CACNA2D4/KCNH2/  GLRB/CLIC5/GABRB2/CLIC2/SHI  SA9/HCN4/KCNJ11/GRIA1/SHIS  A8/KCNJ6/LRRC55/SCN7A/UNC  80/KCNS2/CACNG4/GABRA3/SH  ISA6/KCNQ2/PLN/CNGB3/GABR  A2/CHRNA4/BEST3/GABRR2/CN  GB1/GRIK1/CHRNA9/CHRNA2/KC  NJ3/KCNA3/CACNG3/TRPC3/KC  NJ4/GLRA2/NOS1/GRIA4/FXYD2  /RYR2/LRRC52/CACNA1S/KCNB  2/CACNG6/CACNG7/GABRA1/G  RIN1/GRID2/CACNA1G/GRIN2A/  CHRNA6/KCNG4/CACNA1I/GRIA3  /GABRR3/CNTNAP2/BEST2/KCN |
| --- | --- | --- | --- | --- |

| 0.497710826 12688 |  | tags=38%,  list=22%,  signal=30% |  | 38A6/SLCO2A1/SLC13A4/SLC26  A10/SLC4A11/SFXN1/ANO7/GJ  A1/SLC9A3/SLC26A9/SLC4A5/  SLC17A5/SLC46A1/GABRE/CLIC  6/SLC1A3/SLC24A4/SLC16A13/  SLC6A12/ANO1/GABRG3/SLC22  A11/SLC2A6/SLC4A4/GABRB3/  ANO2/SLC51B/SLC16A9/SLCO2B  1/SLC18A1/SLC16A11/SLC6A9/  GABRQ/SLC16A14/SLC16A8/SLC  6A13/SLC7A3/SLC7A7/SFXN5/S  LC16A6/SLC18A2/GABRA5/SLC6  A20/ADAMTS8/ANO4/SLC26A7  /CLCNKB/SLC26A3/SLC17A7/CL  CNKA/BSND/SLC6A3/MFSD2A/S  LC38A5/CLCA4/SLC38A4/SLCO3  A1/SLC38A8/CLDN4/RTBDN/SLC  6A1/SLC26A8/GABRA4/SLC34A  2/GABRD/APOL1/SLC7A11/SLC  O5A1/CLIC3/SLC9C2/GLRB/CLIC  5/SLCO1A2/SLC6A2/SLC1A1/G  ABRB2/SLC38A3/CLIC2/SLC5A1  2/SLC9A7/ABCC3/SLC34A1/SL  C1A7/SLC36A2/NMUR2/ANO9/S  LC22A8/SLC25A31/SLC12A3/SL  C52A1/GABRA3/SLC9A2/SLC13  A5/SLC27A2/SLC4A8/AKR1C4/  GABRA2/ABCC2/BEST3/GABRR2  /SLC2A14/SLC5A8/GLRA2/ABC  B11/SLC1A2/SLC24A2/GABRA1  /SLC22A6/SLC2A2/SLC6A18/G  ABRR3/BEST2/SLC32A1/GLRA3/  SLC13A2/GABRA6/SLC4A10/GA |
| --- | --- | --- | --- | --- |

| 0.497710826 14762 | |  | | tags=44%,  list=26%,  signal=32% | |  | | CCN1/WNK2/CCL17/ATF3/LRP1  /GAS6/TREM2/FLT4/SERPINF2/  CTSH/FGG/BMPER/NOX4/CD74/  EGFR/TGFB1/SEMA7A/CD4/PLA  2G5/GLIPR2/ANGPT1/FGF8/LYN  /SLC30A10/TRPV4/CAVIN3/PDG  FRB/NECAB2/CHI3L1/SYK/AGT/  NRP1/KDR/CSF1R/PRKCA/ABCA7  /CCL18/LGALS9/CCL14/MT3/F2  R/TNFAIP8L3/GPR183/CCL20/CC  R1/MAP3K12/C5AR2/CCL13/FN1  /THPO/FFAR4/P2RY1/EPO/KLF4  /DRD2/HCRTR1/NPY5R/CCL7/TN  FRSF11A/CCL8/FGF18/FGFR2/S  YT14P1/FGB/OXTR/HAVCR2/MIR  27B/PTPN6/CCL3/CCL19/OR2AT  4/PLA2G2A/CCL23/CCL11/CAS  R/SCIMP/ALOX15/DUSP10/CCL5  /NDRG4/PTPRR/CCL3L3/CCL16/  FSHR/NPNT/IGF1/PTGER4/MIR23  A/IL1B/EPHB2/NOD2/CCL21/TB  C1D10C/CCL25/NTRK1/P2RY6/C  CL24/HTR2A/ADCYAP1/PTPRC/  CCR7/ADRA1A/FPR2/MIR185/CC  L26/CCL4/MIR503/LIF/EGF/IL26  /FGF23/CHRNA9/HTR2B/EPHA7/  NLRP6/GBP1/FGF10/TNF/XCL1/  CCL22/RASGRP1/XCL2/PTPN22/  SULT1A4/S100A7/CALCR/TNFSF  11/SLAMF1/OPRM1/HMGB4/GPR  55/CCL1/FGA/HTR2C |
| --- | --- | --- | --- | --- | --- | --- | --- | --- |
| 0.497710826 9166 |  | | tags=51%,  list=16%,  signal=43% | |  | | EFNB2/PDCD1LG2/CD80/DPP4/T  NFSF4/CD86/PTPN6/CCL19/ICOS  LG/VAV1/HHLA2/CD28/CD160/  CD40LG/TNFSF13B/CCL21/CCR7  /CARD11/CD5/CD3E/GRAP2/TN  FSF14/LCK/BTLA/KLRC4-  KLRK1/PDCD1/KLRK1/ICOS/CTL  A4 | |

| 0.497710826 13684 |  | tags=73%,  list=24%,  signal=55% |  | FLT1/NBL1/LGALS3/LYN/CXCL1  2/PLA2G7/GREM1/ANXA1/CCL1  8/AIF1/CCL14/CCL20/CCR1/CC  L13/SLIT2/IL6/CCN3/CCL7/TNF  RSF11A/SERPINE1/CCL8/CCL3/C  CL19/CCL23/CCL11/CCL5/SLA  MF8/CCL3L3/CXCL17/CCL16/C  ALCA/PTPRO/CCL21/CCL25/CC  L24/FPR2/CCL26/CCR2/CCL4/C  XCL10/XCL1/CCL22/XCL2/S100  A7/TNFSF11/CCL1 |
| --- | --- | --- | --- | --- |

| 0.497710826 12002 |  | tags=57%,  list=21%,  signal=45% |  | CX3CR1/VIPR1/RXFP4/MC1R/PR  OKR2/NPR1/F2R/NTSR1/CCR10/  CCR1/CMKLR1/AVPR1A/NPBWR1  /FPR3/CRHR2/BDKRB1/HCRTR1/  NPY5R/F2RL3/GCGR/OXTR/APLN  R/CCRL2/KISS1R/BDKRB2/SSTR1  /NPFFR1/CRHR1/F2RL2/MCHR2/  SORCS1/MCHR1/GUCY2D/GPR37  L1/TSHR/CCKAR/GLP2R/FPR1/N  PR3/FSHR/CCR9/GALR1/GPR84/  SSTR2/CCKBR/NMUR2/RXFP3/N  MBR/CYSLTR1/GPR83/TACR1/C  CR7/FPR2/CCR2/ADCYAP1R1/C  CR5/MC5R/SCTR/OPRD1/NPFFR2  /NLRP6/CCR3/LGR5/CXCR3/CCR  4/CXCR6/UTS2R/CALCR/MC4R/  HCRTR2/AGTR2/OPRM1/TACR3/  CCR6/PRLHR/CYSLTR2/XCR1/PT  H2R/SSTR3/GUCY2F/CXCR5/CC  R8/NPY2R/SSTR5/GPR32P1/MC3  R |
| --- | --- | --- | --- | --- |

| 0.497710826 14544 |  | tags=51%,  list=26%,  signal=38% |  | LRP1/GAS6/TREM2/FLT4/SERPIN  F2/FGG/BMPER/NOX4/CD74/EG  FR/TGFB1/SEMA7A/CD4/PLA2G  5/GLIPR2/ANGPT1/FGF8/SLC30A  10/TRPV4/CAVIN3/PDGFRB/NEC  AB2/CHI3L1/NRP1/KDR/CSF1R/P  RKCA/ABCA7/CCL18/LGALS9/C  CL14/MT3/F2R/TNFAIP8L3/GPR1  83/CCL20/CCR1/MAP3K12/C5A  R2/CCL13/THPO/FFAR4/P2RY1/  EPO/DRD2/HCRTR1/NPY5R/CCL7  /TNFRSF11A/CCL8/FGF18/FGFR2  /SYT14P1/FGB/HAVCR2/MIR27B  /CCL3/CCL19/OR2AT4/PLA2G2  A/CCL23/CCL11/CASR/SCIMP/  ALOX15/CCL5/NDRG4/CCL3L3/  CCL16/FSHR/NPNT/MIR23A/NOD  2/CCL21/CCL25/NTRK1/P2RY6/  CCL24/HTR2A/ADCYAP1/PTPRC  /CCR7/ADRA1A/FPR2/CCL26/C  CL4/IL26/FGF23/HTR2B/FGF10/  TNF/XCL1/CCL22/RASGRP1/XCL  2/PTPN22/S100A7/CALCR/TNFS  F11/SLAMF1/OPRM1/HMGB4/GP  R55/CCL1/FGA/HTR2C |
| --- | --- | --- | --- | --- |

| 0.497710826 10152 |  | tags=52%,  list=18%,  signal=43% |  | BCL3/WNT5A/IL27RA/CD244/TN  FSF4/HAVCR2/TLR7/CLEC7A/SA  SH3/IL18/EBI3/CD160/IRF8/IFNL  1/IL1B/TLR8/IL27/CCR2/IL23R/  CD3E/CD2/IL12RB1/TNF/SLAMF6  /ISL1/RASGRP1/PTPN22/LTA/IL  12B/KLRC4-  KLRK1/KLRK1/IL21/IL2 |
| --- | --- | --- | --- | --- |

| 0.497710826 12278 |  | tags=45%,  list=22%,  signal=36% |  | LSTN3/SIPA1L1/FABP5/KCND3/  ADGRB1/CLSTN2/DLG2/F2R/DLG  AP1/GABRG3/CNKSR2/EFNB2/NR  GN/GRIK5/P2RX6/LRRC7/GABRB  3/KCNMA1/ADORA1/CAMK2A/R  GS9/SHANK1/HOMER1/SLC6A9/  GABRQ/PDLIM4/P2RY1/HCLS1/D  RD2/GRM2/MX2/DNM3/GABRA5  /GRIN2D/GRIN3A/KCTD16/ADA  M22/CACNG5/EPHA4/NLGN4Y/C  HRNA3/SYNDIG1/SRCIN1/LRRC4/  GRID1/CDH2/SLC6A3/RGS7BP/IQ  SEC3/NLGN4X/PCDH17/SLITRK2/  FAIM2/GSG1L/SLC8A1/SLC6A1/  LRFN2/GABRA4/GABRD/ARC/SH  2D5/BAALC/KCNC1/GRM3/CABP  1/GLRB/CDH9/LHFPL4/GABRB2/  GRM1/CHRM3/SHISA9/LRFN5/G  ABBR2/GRIA1/SHISA8/EPHB2/PT  PRO/PRRT2/ITGB3/IGSF21/ADCY  8/LRRTM1/INSYN2A/LRRC4C/NT  RK3/HTR2A/ADRA1A/P2RX1/CA  CNG4/GABRA3/SHISA6/ADCY1/  PLPPR4/NETO1/GABRA2/CNTN2/  CHRNA4/GABRR2/ATP2B2/GRIK1  /OPRD1/CHRNA9/CHRNA2/KCTD  8/KCNA3/EPHA7/CACNG3/KCNJ  4/GLRA2/PCDH8/GRIA4/ANK1/L  RRTM2/SLITRK1/CACNG7/GABRA  1/DRD3/GRIN1/GRID2/GRIN2A/C  HRNA6/GRIA3/GABRR3/DRD1/GR  M7/OPRM1/HTR5A/SLC8A3/GLR  A3/LRRTM4/GABRA6/GRM5/GA |
| --- | --- | --- | --- | --- |

| 0.497710826 9821 |  | tags=19%,  list=17%,  signal=16% |  | NLRC4/KCNF1/KCTD16/KCNV1/L  CN2/RIPK3/KCNV2/PKD2L1/KCT  D4/KCNA2/OTC/KCTD17/TRPM2  /ITLN1/CLDN1/CD79A/SLC6A1/  ARC/ACPP/KCNC1/SLC1A1/AQP  4/LY6G6C/CRYAA/C9/SLC34A1  /TDO2/PRF1/CALHM1/ADCY8/C  LDN3/SYT1/KCNS2/P2RX1/FSD1  /ILDR1/PLN/CD3E/CD247/KCTD8  /KCNA3/CD3D/GBP1/SLC1A2/K  CNB2/SLC22A6/PRMT8/TCL1A/  KCNG4/GBP5/REG1A/KCNA4/DP  YS/CD3G/GLRA3/KCND2/KCNC2  /GLRA1/KCNA1/KCNA10 |
| --- | --- | --- | --- | --- |

| 0.497710826 10528 |  | tags=33%,  list=19%,  signal=27% |  | CAMK1/LRP8/IL1RAPL2/HOMER1  /HCLS1/WNT5A/DRD2/ADGRB3/  MX2/LRRC24/DNM3/DKK1/NFAT  C4/THBS2/SLITRK5/OXTR/EPHA4  /SLC17A7/GPC6/SYNDIG1/SRCI  N1/CDH2/MDGA1/IL10RA/SLITR  K2/LILRB2/LRFN2/GPM6A/ARC/I  TPKA/SLC7A11/ADGRB2/IL10/L  HFPL4/RELN/GPC4/LRFN5/NTN1/  EPHB2/PTPRO/NTNG2/NTRK1/LRR  TM1/SIX4/NTRK3/IGSF9/LRRN3/  AMIGO2/SHISA6/LRTM1/SLIT1/P  TPRD/NRXN1/ADGRL3/CUX2/EPH  A7/SLITRK4/PCDH8/TNF/CAMKV  /LRRTM2/SLITRK1/GRIN1/DGKB/  ZNF804A/GRID2/ASIC2/SLITRK6  /FRMPD4/CBLN2/NEUROD2/LRTM  2/SLITRK3/SLC17A8/LRRTM3/SL  C17A6/GHSR |
| --- | --- | --- | --- | --- |

| 0.497710826 12256 |  | tags=44%,  list=22%,  signal=34% |  | ATSPER3/CACNA1F/HPN/CACNB  4/ASIC1/SLC24A4/KCND3/TRPV  2/SCNN1G/ANO1/CACNA2D1/K  CNN4/KCNAB1/KCNJ1/KCNU1/K  CNT1/P2RX2/GRIK5/P2RX6/KCN  MA1/KCNE1/KCNE2/CACNG1/KC  NK6/KCNMB4/SCN4A/KCNN3/HC  N2/TRPM5/TMEM37/PKD1L3/AQ  P1/KCNF1/JPH1/CACNB1/GRIN2  D/GRIN3A/KCNQ3/SCN1B/KCNJ8  /KCNV1/LRRC38/KCNV2/PKD2L1  /CACNG5/SCN4B/NOX5/CHRNA  3/KCNA2/KCNMB1/ABCC9/SCN2  B/TRPM2/KCNK1/SCN9A/KCNE1  B/P2RX5/SCNN1B/SCNN1A/KCN  H7/CACNA1E/FAM155A/TRPM8/  CACNA2D3/ABCC8/GPM6A/TRP  C6/KCNC1/KCNK10/CACNA2D4/  KCNH2/ITPR2/TRPM3/KCNK9/HC  N4/KCNJ10/KCNJ11/GRIA1/TRP  V6/KCNK18/KCNK13/KCNJ6/KCN  T2/CALHM1/LRRC55/SCN7A/UN  C80/KCNS2/OTOP2/TRPV3/P2RX  1/HTR1B/CACNG4/KCNQ2/CNGB  3/CNGA1/CHRNA4/KCNK3/CNGB  1/GRIK1/CHRNA9/CHRNA2/KCNJ  3/KCNA3/CACNG3/TRPC3/KCNJ  4/GRIA4/RYR2/LRRC52/KCNH6/  CACNA1S/KCNB2/CACNG6/CAC  NG7/SLC24A2/GRIN1/CACNA1G  /GRIN2A/CHRNA6/ASIC2/KCNG4  /TMC1/CACNA1I/GRIA3/OTOP3  /KCNJ9/KCNA4/KCNJ16/SCN1A |
| --- | --- | --- | --- | --- |

| 0.497710826 10525 |  | tags=65%,  list=19%,  signal=53% |  | 73/C8G/IGLV3-  21/C1R/CD19/IGKV3-  20/IGLC2/IGLV2-  8/IGHA1/IGHM/IGHA2/C1S/CFB  /IGHV4-59/IGHV5-51/IGLV2-  14/IGHG2/IGHV1-  45/IGHG1/IGHV4-  39/IGHE/IGHV3-  23/IGKC/VTN/IGLV2-  23/IGHV3-35/IGHV4-31/IGLV2-  11/IGHV4-34/IGKV3-  15/IGLC7/IGHV3-48/IGLV3-  19/IGKV1-5/IGHV3-11/IGHV1-  69D/APCS/IGHV3-21/IGLV1-  44/IGHV1-3/IGHG3/IGHV4-  61/IGHV1-18/IGLC6/C7/IGHV3-  30/IGHV2-70D/IGHV2-  5/IGLL5/IL1B/IGLC3/C9/IGHG4/  CR1L/TRDC/IGLV1-40/IGHV1-  58/IGHD/IGHV4-28/IGKV1-  17/IGHV1-24/IGHV3-  33/IGKV3D-11/IGHV2-  70/IGHV3-7/TRBC2/IGLV3-  25/IGLV7-43/IGHV3-20/IGLV3-  1/IGHV3-74/IGKV1-  16/C4B/IGHV3-13/C4A/IGHV4-  4/CRP/IGHV3-66/FCN2/IGHV3-  72/IGHV1-69/SUSD4/IGKV5-  2/IGKV2D-28/IGKV2D-  30/IGKV1-12/IGHV3-  53/IGKV3D-20/IGHV6-  1/IGKV2-29/IGHV3-  64/IGKV1D-12/IGKV2- |
| --- | --- | --- | --- | --- |

| 0.497710826 12096 |  | tags=58%,  list=21%,  signal=46% |  | CACNA1F/RPE65/EYS/RHO/GNA  T1/RRH/GUCA1A/RGR/SEMA5B/  PDE6C/TULP1/RGS9BP/AIPL1/GU  CY2D/GPR88/OPN1LW/ELOVL4/  CACNA2D4/SAG/PDE6A/GRM6/  ATP8A2/ABCA4/GNGT1/OPN4/C  NGA1/GRK1/CNGB1/GUCA1C/GR  K7/RP1/OPN5/GJA10/GUCY2F/  GNAT2/OPN1MW |
| --- | --- | --- | --- | --- |

| 0.497710826 9494 |  | tags=35%,  list=17%,  signal=29% |  | IGKV3-20/IGLC2/IGLV2-  8/MAPK10/IGHV4-59/IGLV2-  14/LAT2/IGHV4-  39/IGHE/IGHV3-  23/IGKC/VAV1/IGLV2-  23/PSMB9/IGLV2-11/IGHV4-  34/IGKV3-15/IGLC7/IGHV3-  48/LCP2/IGLV3-19/IGKV1-  5/IGHV3-11/IGLV1-  44/IGLC6/NFATC2/IGHV3-  30/IGHV2-5/IGLC3/IGLV1-  40/IGKV1-17/IGHV3-  33/IGKV3D-11/CARD11/IGHV2-  70/IGHV3-7/IGLV3-25/IGLV7-  43/IGLV3-1/IGKV1-  16/GRAP2/IGHV3-13/IGHV1-  69/IGKV5-2/IGKV2D-  28/IGKV2D-30/IGKV1-  12/IGHV3-53/ITK/IGKV3D-  20/IGKV2-29/IGKV1D-  12/IGKV2-30/IGKV1D-  39/IGKV1-39/IGLV3-  27/IGKV1D-33/IGKV2-28 |
| --- | --- | --- | --- | --- |

| 0.497710826 9494 |  | tags=89%,  list=17%,  signal=74% |  | IGKV3-  20/IGLC2/IGHA1/IGHM/IGHA2/I  GHV4-59/IGHV5-  51/IGHG2/IGHV1-  45/IGHG1/IGHV4-  39/IGHE/IGHV3-  23/IGKC/IGHV3-35/IGHV4-  31/IGHV4-34/IGLC7/IGHV3-  48/IGHV3-11/IGHV1-  69D/IGHV3-21/IGHV1-  3/IGHG3/IGHV4-61/IGHV1-  18/IGLC6/JCHAIN/IGHV3-  30/IGHV2-70D/IGHV2-  5/IGLL5/IGLC3/IGHG4/TRDC/IGH  V1-58/IGHD/IGHV4-28/IGHV1-  24/IGHV3-33/IGHV2-70/IGHV3-  7/TRBC2/IGHV3-20/IGHV3-  74/IGHV3-13/IGHV4-4/IGHV3-  66/IGHV3-72/IGHV1-69/IGHV3-  53/IGHV6-1/IGHV3-64/IGHV3-  38/IGHV7-81/IGHV3-  43/TRBC1/IGHV3-16 |
| --- | --- | --- | --- | --- |

| 0.497710826 11604 |  | tags=59%,  list=21%,  signal=47% |  | KCND3/ADAM23/CLSTN2/GABRG  3/EFNB2/GRIK5/P2RX6/ADORA1  /SLC6A9/P2RY1/DRD2/GRM2/C  ADM3/GABRA5/ADAM22/CACN  G5/EPHA4/KCNA2/SYT11/LRRC4  /GRID1/CDH2/SLC6A3/RGS7BP/  PCDH17/SLITRK2/GSG1L/SLC8A1  /SLC6A1/LRFN2/GABRA4/GPM6  A/GABRD/KCNC1/GRM3/CDH9/  GABRB2/CHRM3/SHISA9/GPC4/L  RFN5/GRIA1/EPHB2/PTPRO/PRRT  2/NTNG2/ADCY8/LRRTM1/LRRC  4C/NTRK3/HTR2A/ADRA1A/P2R  X1/HTR1B/CACNG4/GABRA3/SH  ISA6/ADCY1/PLPPR4/NETO1/CN  TN2/CHRNA4/SYT6/CNTN5/NRX  N1/OPRD1/CHRNA9/KCNJ3/KCN  A3/EPHA7/CACNG3/PCDH8/SLC  1A2/LRRTM2/SLITRK1/CACNG7/  GABRA1/DRD3/GRIN1/GRID2/GRI  N2A/CHRNA6/KCNJ9/DRD1/OPR  M1/HTR5A/SCN2A/SLC8A3/KCN  D2/HTR3A/SLITRK3/CACNG2/GL  RA1/LRRTM3/KCNA1/CHRM2/GH  SR/CPLX3 |
| --- | --- | --- | --- | --- |

| 0.497710826 13298 |  | tags=44%,  list=24%,  signal=34% |  | G/CCDC88B/ITGB2/LGALS3/RUN  X3/LYN/NRARP/LGALS9B/FOXJ1  /CXCL12/PELI1/JAK3/ELANE/PE  CAM1/IGFBP2/TNFRSF21/SYK/A  RG2/LOXL3/SIRPB1/ANXA1/CX3  CR1/AIF1/THY1/LGALS9/VSIG4  /ADD2/TNFSF9/EFNB2/CD1D/HL  A-  DPA1/NLRP3/PDCD1LG2/PIK3R6/  RAC2/CD177/CD80/LILRB4/PLA2  G2D/IGF2/HLA-  DMB/ADAM8/IL6/VSIR/EPO/KLF  4/SELPLG/ITGA5/IL7/IFNB1/DPP  4/PTAFR/TNFSF4/PLA2G2E/HAV  CR2/RUNX1/BMP7/TNFAIP8L2/C  ORO1A/CD86/IL1RL2/ADTRP/NT  5E/SELE/PTPN6/CCL19/PLA2G2A  /FOXP3/ICOSLG/NCKAP1L/VAV  1/VCAM1/SASH3/DUSP10/SELL  /LILRB2/CCL5/CD300A/IL7R/IL1  8/CD209/EBI3/HHLA2/CD28/CD  6/GLI2/IL10/CD160/FGL2/IFNL1  /CLEC4G/CD40LG/IGF1/IL1B/TN  FSF13B/IL2RA/NOD2/CCL21/UM  OD/CCL25/LILRB1/IRF1/ITGAL/P  TPRC/CCR7/CLECL1/RASAL3/CC  R2/CARD11/IL15/MYB/CD5/GA  TA3/IL23R/CD3E/SHH/IL12RB1/  CD27/SPN/TBX21/SKAP1/GRAP2  /TNFSF14/TNF/ITGA4/LCK/ZAP  70/XCL1/RASGRP1/PTPN22/CD7  0/LAG3/TNFSF11/RHOH/TESPA1  /IL12B/IDO1/BTLA/KLRC4- |
| --- | --- | --- | --- | --- |

| 0.497710826 10445 |  | tags=35%,  list=18%,  signal=28% |  | RASD2/PREX2/DMRT3/UCHL1/FG  F12/DRD2/ZIC1/GBX1/PDE1B/PE  NK/SLC18A2/GRIN2D/ADAM22/  NOVA1/DPP4/EPHA4/DAB1/CHR  NA3/ATP1A2/TRH/SLC6A3/PPP1  R1B/SLURP1/GPR88/NRG1/CDH23  /ASTN1/GLRB/KLHL1/GRM1/CE  ND1/KCNJ10/SEZ6/GRM6/ADCY  8/LRRTM1/EN1/CALB1/PAK5/C  NTN2/DSCAM/CHRNA4/ADGRL3  /OPRD1/BSX/FEZF2/CRH/DMBX  1/DRD3/GRIN1/TNR/NEGR1/OTO  G/SLITRK6/DRD1/IDO1/GAD1/S  CN1A/OPRM1/SLC4A10/GRM5/K  CND2/GLRA1/NPY2R/GHSR/HTR2  C/MC3R/NMS |
| --- | --- | --- | --- | --- |

| 0.497710826 11866 |  | tags=43%,  list=21%,  signal=34% |  | CACNB4/CASQ2/CRHBP/CACNA  2D1/DLG2/KCNAB1/MIR26A2/NT  SR1/CRACR2A/LRRC7/KCNE1/KC  NE2/RGS9/SHANK1/HOMER1/HR  C/DYSF/EPO/FGF12/DRD2/JPH1  /CACNB1/REM1/NOS1AP/SCN1B  /LRRC38/CACNG5/MIR212/CAS  Q1/CRHR1/JSRP1/STAC/KCNE1B  /RRAD/GSG1L/MMP9/GEM/ARC  /KCNC1/RELN/CLIC2/HPCA/SHI  SA9/KCNJ11/GRIA1/SHISA8/EPH  B2/P2RY6/NEFL/LRRC55/KCNS2/  HAP1/CCR2/CACNG4/SHISA6/PL  N/RASGRF1/NETO1/PIRT/CACNG  3/NOS1/GRIA4/LRRC52/CRH/CA  CNG7/TRDN/DRD3/GRIN1/GRIN2  A/GRIA3/OPRM1/GRIA2/CACNG  2/KCNC2/KCNA1/IFNG/OR56A5  /MIR153-1 |
| --- | --- | --- | --- | --- |

| 0.497710826 13610 |  | tags=39%,  list=24%,  signal=30% |  | ATPSCKMT/METTL21C/CTSS/GP  R35/DLG4/TCIRG1/GNB5/ANK2/  LYN/CACNB2/CACNA1C/GALR2  /ATP1B2/GJC2/HAMP/FGF14/A  GT/SLC31A2/CACNB4/CASQ2/T  HY1/CEMIP/CRHBP/CACNA2D1/  DLG2/F2R/KCNAB1/MIR26A2/NT  SR1/CRACR2A/GLRX/LRRC7/KC  NE1/KCNE2/RGS9/CACNG1/SHA  NK1/HOMER1/HRC/DYSF/EPO/F  GF12/DRD2/BDKRB1/CD19/JPH1  /CACNB1/REM1/NOS1AP/F2RL3  /ADRA2A/SCN1B/RGN/PM20D1/  LRRC38/STRIT1/CACNG5/SCN4B  /APLNR/NOX5/MIR212/CASQ1/  KCNMB1/ATP1A2/CORO1A/SCN2  B/FXYD7/PTPN6/CRHR1/JSRP1/  STAC/KCNE1B/RRAD/GSG1L/SL  C8A1/MMP9/TWIST1/GEM/ARC  /IL13/CAV3/KCNC1/HECW2/KC  NH2/RELN/CLIC2/HPCA/SHISA9/  KCNJ11/GRIA1/SHISA8/EPHB2/PI  K3CG/HECW1/P2RY6/NEFL/LRRC  55/KCNS2/HAP1/CCR2/CACNG4  /SHISA6/PLN/RASGRF1/NETO1/  PIRT/CXCL10/CXCL11/CACNG3/  NOS1/GRIA4/FXYD2/XCL1/CXC  L9/RYR2/MRLN/LRRC52/CRH/CA  CNG7/TRDN/DRD3/GRIN1/GRIN2  A/CALCR/TMC1/GRIA3/DRD1/O  PRM1/GRIA2/CACNG2/KCNC2/K  CNA1/IFNG/OR56A5/MIR153-1 |
| --- | --- | --- | --- | --- |

| 0.497710826 15460 |  | tags=37%,  list=27%,  signal=27% |  | SEMA3D/GH1/SEMA4D/POR/HEY  2/PLCB1/STK3/MBD5/STAT3/M  ACF1/DRAXIN/DLL1/PRKDC/LRP  1/TRIM46/GDF15/PLXNA3/FGF1  3/AKAP6/ZP3/CHD7/CXCR4/DI  O3/BCL2L11/SH3PXD2B/FGFR1/S  EMA7A/CSF1/SEMA5A/ACACB/  RND2/SEMA3A/SIX1/FGF8/COL  Q/AGR2/MAP2/NKX2-  5/GJA1/SFRP2/CXCL12/SEMA3  G/HAMP/CDKN1A/OLFM1/BASP1  /WT1/NRP1/NGF/FOXC1/ISLR2  /TNFRSF12A/MT3/PHLDA2/TRPV  2/TBX5/MAPT/SEMA3F/UNC13A  /TBX20/PI16/FN1/IGF2/GLI1/CR  ABP2/STC2/TP73/NRK/HCLS1/  WNT5A/DRD2/TLL2/NOG/FOXS1  /SEMA6B/WNT2/IL7/MYOD1/FG  FR2/FOXC2/SYT14P1/GJD4/POU  3F2/ARX/MAP1B/SLC6A3/GDF5  /PRLH/NPPC/SASH3/DUSP10/MS  TN/FSTL4/PLXNA4/CAV3/SEMA  3E/MIR199A1/SOCS2/IGF1/NTN1  /ZFPM2/BARHL2/SIX4/ATP8A2/  NTRK3/PLAC8/SYT1/GATA6/CP  NE5/SYT4/DSCAM/SYT2/EPHA7  /ADRB1/CPNE6/CACNG7/DRD3/  TNR/REG1A/RIMS2/RIMS1/POU4  F2/GHSR/MIR204/MIR199B/MIR2  00B/MIR208A |
| --- | --- | --- | --- | --- |

| 0.497710826 15206 |  | tags=50%,  list=27%,  signal=37% |  | ICAM1/CX3CL1/CD58/CXCL16/  TLR3/B2M/SHFL/CCL17/CAMK2B  /CITED1/HLA-  DPB1/CDC42EP2/ASS1/STXBP2/  HLA-DRA/HLA-  G/OAS1/MRC1/IRF7/HLA-  H/STAT1/HLA-  DRB1/TXK/OAS2/CCL18/IFITM1  /PARP9/AIF1/LGALS9/BST2/CC  L14/TRIM22/CCL20/HLA-  DPA1/SOCS3/HLA-DRB5/HLA-  F/CAMK2A/CCL13/GCH1/IRF5/I  FI30/SLC30A8/OASL/WNT5A/H  LA-  DQA2/NCAM1/WAS/CCL7/IRF9  /HCK/CCL8/PTAFR/HLA-  DQB1/CASP1/CCL3/CCL19/CLD  N1/CCL23/HLA-  DQA1/CCL11/HLA-  DQB2/VCAM1/TDGF1/CCL5/RAB  7B/CCL3L3/MEFV/IRF8/CCL16/  GBP4/NLRC5/AQP4/CCL21/FCGR  1A/CCL25/GBP3/CIITA/GBP2/C  CL24/IRF1/CCL26/CCL4/IL23R/F  CGR1B/ACOD1/GBP7/IL12RB1/G  BP1/XCL1/CCL22/XCL2/UBD/GB  P6/GBP5/IL12B/CCL1/IFNG |
| --- | --- | --- | --- | --- |

| 0.497710826 12148 |  | tags=37%,  list=21%,  signal=29% |  | LOXL3/UNC13D/PLCL2/ANXA1/  LGALS9/GPR183/LFNG/NLRP3/LE  F1/CD80/HLA-F/DOCK11/HLA-  DMB/IL6/BCL3/TNFSF18/IL27RA  /MFNG/CD244/CD1C/CD19/LCP  1/IFNB1/CLEC4E/TNFSF4/HAVCR  2/CDH17/CD180/CORO1A/CD86  /DOCK10/PGLYRP3/CCL19/FOXP  3/IFNW1/APBB1IP/IL18/IFNE/CD  28/IL10/FGL2/IFNL1/CD40LG/PT  GER4/EOMES/LILRB1/BATF/ZNF6  83/ITGAL/PTPRC/IL27/LY9/MYB  /GATA3/IL23R/IL12RB1/SPN/TB  X21/SLAMF6/AICDA/IL12B/PGL  YRP2/CCR6/IFNG/IFNA10/IFNA2  1/IL2 |
| --- | --- | --- | --- | --- |

| 0.497710826 8879 |  | tags=52%,  list=16%,  signal=44% |  | PIK3R6/NCR1/ULBP2/HLA-  F/HAVCR2/CORO1A/PTPN6/SH2  D1B/SLAMF7/IL18RAP/VAV1/IL1  8/ULBP1/CD160/CADM1/LILRB1  /KIR3DL1/KLRC2/GZMB/KLRD1/  NCR3/KIR2DL4/SLAMF6/RASGRP  1/CD96/LAG3/IL12B/KLRC4-  KLRK1/CRTAM/CLNK/SH2D1A/K  LRK1/IL21 |
| --- | --- | --- | --- | --- |
| 0.497710826 7476 |  | tags=46%,  list=13%,  signal=40% |  | GABRA4/KCNC1/UNC5A/HPCA/  SHISA9/KCNJ11/GRIA1/SHISA8/  USH2A/LAMP5/AKAP5/ADGRV1/  GABRA3/SHISA6/GABRA2/OPRD1  /SLC1A2/ANK1/GABRA1/CNTNA  P2/REG1A/OPRM1/TACR3/GABR  A6/GABRG2/KCNC2/GABRG1 |

| 0.497710826 9679 |  | | tags=51%,  list=17%,  signal=42% | |  | | GNAT1/RRH/GUCA1A/RGR/PDE6  C/AIPL1/GUCY2D/GPR88/OPN1L  W/GNGT2/SAG/PDE6A/ABCA4/  PDC/GNGT1/OPN4/CNGA1/GRK1  /CNGB1/GUCA1C/NR2E3/TRPC3  /GRK7/CABP4/RP1/OPN5/ASIC2  /GUCY2F/GNAT2/OPN1MW | |  |
| --- | --- | --- | --- | --- | --- | --- | --- | --- | --- |
| 0.497710826 15197 | |  | | tags=50%,  list=27%,  signal=36% | |  | | BAX/CX3CL1/RAMP3/NLGN3/ST  AC3/ATP2A1/ANO6/KCNE5/WN  K2/TREM2/PLCG2/AKAP6/EHD3/  ATP1B1/STAC2/ABCB1/ATPSCK  MT/CTSS/ANK2/CACNB2/GALR2  /ATP1B2/GJC2/FGF14/AGT/TH  Y1/CEMIP/CACNA2D1/F2R/NTSR  1/CRACR2A/GLRX/KCNE1/KCNE  2/RGS9/BDKRB1/CD19/NOS1AP/  F2RL3/RGN/LRRC38/PTAFR/APLN  R/CASQ1/KCNMB1/STAC/TRPC6  /ARC/IL13/KCNC1/KCNH2/RELN  /KCNJ11/EPHB2/SLC34A1/P2RY6  /LRRC55/HAP1/CCR2/CACNG4/  PIRT/CXCL10/CXCL11/CACNG3/  NOS1/XCL1/CXCL9/RYR2/LRRC5  2/TRDN/CALCR/DRD1/HTR3A/C  ACNG2/KCNC2/KCNA1/IFNG | |

| 0.497710826 9827 |  | tags=51%,  list=17%,  signal=42% |  | IL27RA/MNDA/CD19/IL7/IGLC2/  IGHA1/TNFSF4/IGHM/INPP5D/IGH  A2/NFAM1/IGHV4-59/IGHV5-  51/IGHG2/IGHV1-  45/IGHG1/IGHV4-  39/IGHE/PTPN6/IGHV3-  23/IGKC/FOXP3/NCKAP1L/SASH  3/IGHV3-35/CD300A/IGHV4-  31/IGHV4-  34/SLAMF8/IGLC7/IGHV3-  48/TNFRSF4/IL13/CD28/IGHV3-  11/IGHV1-69D/IGHV3-  21/IL10/IGHV1-  3/IGHG3/IGHV4-61/IGHV1-  18/IGLC6/NFATC2/IGHV3-  30/IGHV2-70D/IGHV2-  5/IGLL5/IGLC3/TNFSF13B/IGHG4  /NOD2/TRDC/TBC1D10C/IGHV1  -58/IGHD/IGHV4-28/IGHV1-  24/IGHV3-  33/PTPRC/SAMSN1/CARD11/IGH  V2-70/IGHV3-  7/CD38/TRBC2/IGHV3-  20/CD27/TBX21/IGHV3-  74/BANK1/IGHV3-13/IGHV4-  4/IGHV3-66/IGHV3-72/IGHV1-  69/MZB1/SLA2/IKZF3/IGHV3-  53/IGHV6-1/IGHV3-64/IGHV3-  38/IGHV7-81/IGHV3-  43/TRBC1/TNFRSF13B/CTLA4/IL  21/FCRL3/IGHV3-16/IL2 |
| --- | --- | --- | --- | --- |

| 0.497710826 14404 |  | tags=35%,  list=25%,  signal=26% |  | HLA-  DPB1/PAWR/PLCG2/PSMB10/PR  AM1/NFKBIZ/CD4/HLA-  DRA/PSMB8/LGALS3/BTNL10/HL  A-  DRB1/TXK/TNFRSF21/CACNA1F/  LAT/BTN3A3/THY1/KCNN4/HLA  -DPA1/HLA-  DRB5/THEMIS2/LILRB4/FYB2/RFT  N1/HLA-  DQA2/WAS/FYB1/BTNL8/HLA-  DQB1/INPP5D/PTPN6/FOXP3/ICO  SLG/HLA-DQA1/HLA-  DQB2/PSMB9/CD300A/LCP2/HHL  A2/CD28/CD160/TRAC/BTN1A1  /PTPRC/CCR7/CARD11/GATA3/  CD3E/CD247/SKAP1/GRAP2/CD3  D/GBP1/TRAV8-  4/LCK/ZAP70/PTPN22/UBASH3A  /TRBV7-  9/TRAV29DV5/ITK/TESPA1/CD3  G/TRBC1/TRAV19/THEMIS/CTLA  4/TRAT1/TRBV12-3 |
| --- | --- | --- | --- | --- |

| 0.497710826 12209 |  | tags=49%,  list=22%,  signal=39% |  | CACNA1H/CATSPER3/CLIC6/CA  CNA1F/CACNB4/KCND3/ANO1/  CACNA2D1/KCNAB1/KCNJ1/KCN  U1/KCNT1/KCNMA1/KCNE1/KCN  E2/CYBB/CACNG1/KCNK6/SCN4  A/HCN2/TRPM5/TMEM37/KCNF1  /CACNB1/GRIN2D/GRIN3A/KCNQ  3/SCN1B/KCNJ8/KCNV1/LRRC38  /KCNV2/CLCNKB/CACNG5/SCN4  B/CLCNKA/KCNA2/SCN2B/KCNK  1/SCN9A/KCNE1B/KCNH7/CACN  A1E/CACNA2D3/KCNC1/KCNK10  /CLIC3/CACNA2D4/KCNH2/CLIC  5/CLIC2/KCNK9/HCN4/KCNJ10/  KCNJ11/KCNK18/KCNK13/KCNJ6  /KCNT2/CALHM1/LRRC55/SCN7  A/KCNS2/HTR1B/CACNG4/KCNQ  2/KCNK3/KCNJ3/KCNA3/CACNG  3/KCNJ4/LRRC52/KCNH6/CACN  A1S/KCNB2/CACNG6/CACNG7/  GRIN1/CACNA1G/GRIN2A/KCNG4  /TMC1/CACNA1I/KCNJ9/KCNA4  /KCNJ16/SCN1A/OPRM1/SCN2A  /KCND2/KCNH5/SCN3A/CACNG2  /KCNC2/KCNA1/KCNA10/SLC17  A3 |
| --- | --- | --- | --- | --- |

| 0.497710826 11604 |  | tags=55%,  list=21%,  signal=43% |  | KCND3/KCNAB1/KCNJ1/KCNT1/  KCNMA1/KCNE1/KCNE2/KCNK6/  HCN2/KCNF1/KCNQ3/KCNJ8/KC  NV1/LRRC38/KCNV2/KCNA2/KC  NK1/KCNE1B/KCNH7/KCNC1/KC  NH2/KCNK9/HCN4/KCNJ10/KCNJ  11/KCNK18/KCNJ6/KCNT2/LRRC  55/KCNS2/KCNQ2/KCNK3/KCNJ  3/KCNA3/KCNJ4/LRRC52/KCNH6  /KCNB2/KCNG4/KCNJ9/KCNA4/  KCNJ16/KCND2/KCNH5/KCNC2/  KCNA1/KCNA10 |
| --- | --- | --- | --- | --- |

| 0.497710826 11254 |  | tags=79%,  list=20%,  signal=63% |  | DPA1/HLA-DRB5/IGLV6-  57/TAP1/HLA-  F/LILRB4/FCN3/IGHV3-  73/IGLV3-21/CD1C/IGKV3-  20/IGLC2/IGLV2-  8/IGHA1/HLA-  DQB1/IGHM/IGHA2/IGHV4-  59/IGHV5-51/IGLV2-  14/IGHG2/IGHV1-  45/CD1E/IGHG1/IGHV4-  39/IGHE/CD1A/IGHV3-  23/IGKC/HLA-  DQA1/LILRA1/IGLV2-23/IGHV3-  35/CD48/IGHV4-31/IGLV2-  11/IGHV4-34/IGKV3-  15/IGLC7/IGHV3-  48/IL7R/CD209/IGLV3-  19/IGKV1-5/IGHV3-11/IGHV1-  69D/IGHV3-21/IGLV1-  44/IGHV1-3/IGHG3/IGHV4-  61/PLG/IGHV1-  18/IGLC6/JCHAIN/IGHV3-  30/IGHV2-70D/IGHV2-  5/IGLL5/IGLC3/IGHG4/TRDC/CD  1B/IGLV1-40/IGHV1-  58/IGHD/IGHV4-28/IGKV1-  17/IGHV1-24/IGHV3-  33/IGKV3D-11/KLRC2/IGHV2-  70/KLRD1/IGHV3-  7/TRBC2/IGLV3-25/IGLV7-  43/IGHV3-20/IGLV3-1/IGHV3-  74/IGKV1-16/IGHV3-13/IGHV4-  4/TRAV8-4/ITGA4/IGHV3- |
| --- | --- | --- | --- | --- |

| 0.497710826 12288 |  | tags=43%,  list=22%,  signal=34% |  | MEIS2/AGT/ASIC1/HRH1/NRGN/  CCK/SHANK1/HRH2/DRD2/BRSK1  /PDE1B/LRRN4/GABRA5/NPTX2/  ATP1A2/PPP1R1B/NDRG4/ADAM  2/SLC7A11/MAP1A/RELN/NETO  1/DRD5/TAC1/CRH/FOXB1/DRD  3/GRIN1/TNR/GRIN2A/DRD1/NE  UROD2/TBR1 |
| --- | --- | --- | --- | --- |

| 0.497710826 9697 |  | tags=67%,  list=17%,  signal=56% |  | MNDA/CD19/IGLC2/IGHA1/GCS  AML/IGHM/IGHA2/NFAM1/IGHV  4-59/RUNX1/IGHV5-  51/IGHG2/IGHV1-  45/IGHG1/LAT2/IGHV4-  39/IGHE/PTPN6/IGHV3-  23/IGKC/CD79A/NCKAP1L/IGHV  3-35/CD300A/IGHV4-  31/IGHV4-34/IGLC7/IGHV3-  48/IGHV3-11/IGHV1-  69D/IGHV3-21/KLHL6/IGHV1-  3/IGHG3/IGHV4-61/IGHV1-  18/IGLC6/NFATC2/IGHV3-  30/IGHV2-70D/IGHV2-  5/IGLL5/IGLC3/IGHG4/TRDC/IGH  V1-58/IGHD/IGHV4-28/IGHV1-  24/IGHV3-33/PTPRC/IGHV2-  70/PAX5/IGHV3-  7/CD38/TRBC2/GCSAM/IGHV3-  20/IGHV3-74/IGHV3-13/IGHV4-  4/STAP1/LCK/IGHV3-  66/IGHV3-72/PTPN22/IGHV1-  69/IGHV3-53/ITK/IGHV6-  1/IGHV3-64/IGHV3-38/IGHV7-  81/IGHV3-  43/TRBC1/MS4A1/CTLA4/FCRL3  /IGHV3-16 |
| --- | --- | --- | --- | --- |

| 0.497710826 11741 |  | tags=36%,  list=21%,  signal=29% |  | CASQ2/THY1/CEMIP/CACNA2D1  /F2R/P2RX2/NTSR1/RGS9/HRC/  PLCD4/MCUB/EPO/DRD2/BDKRB1  /CD19/JPH1/GRIN2D/F2RL3/APL  NR/CASQ1/ATP1A2/CORO1A/T  RPM2/PTPN6/CCL3/CCL19/JSRP  1/P2RX5/SLC8A1/FAM155A/IL1  3/ITPR2/CLIC2/TRPV6/PLCH2/C  CL21/P2RY6/PLCH1/HTR2A/PTPR  C/CCR7/ADRA1A/HAP1/ADCYA  P1R1/CCR5/PLN/CXCL10/CXCL1  1/HTR2B/TRPC3/NOS1/LCK/XCL  1/CXCL9/RYR2/TRDN/GRIN1/GR  IN2A/CALCR/DRD1/SLC8A3/FAS  LG/MS4A1/XCR1/HTR2C |
| --- | --- | --- | --- | --- |

| 0.497710826 10132 |  | tags=45%,  list=18%,  signal=37% |  | IGLV3-21/CYFIP2/WAS/IGKV3-  20/IGLC2/HCK/IGLV2-  8/ELMO1/IGHV4-59/IGLV2-  14/IGHG2/IGHG1/IGHV4-  39/IGHV3-  23/IGKC/NCKAP1L/VAV1/IGLV2  -23/IGLV2-11/IGHV4-  34/IGKV3-15/IGLC7/IGHV3-  48/IGLV3-19/IGKV1-5/IGHV3-  11/MYO1G/IGLV1-  44/FCGR3A/IGHG3/IGLC6/IGHV3  -30/IGHV2-  5/IGLC3/IGHG4/FCGR1A/IGLV1-  40/IGKV1-17/IGHV3-  33/PTPRC/IGKV3D-11/IGHV2-  70/IGHV3-7/IGLV3-25/IGLV7-  43/IGLV3-1/CD247/IGKV1-  16/IGHV3-13/IGHV1-69/IGKV5-  2/IGKV2D-28/IGKV2D-  30/IGKV1-12/IGHV3-  53/IGKV3D-20/IGKV2-  29/IGKV1D-12/IGKV2-  30/CD3G/IGKV1D-39/IGKV1-  39/IGLV3-27/IGKV1D-  33/IGKV2-28 |
| --- | --- | --- | --- | --- |

| 0.497710826 10452 |  | tags=75%,  list=18%,  signal=61% |  | 73/C8G/FCER2/BCL3/IGLV3-  21/C1R/IGKV3-  20/IGLC2/IGLV2-  8/IGHA1/HLA-  DQB1/IGHM/IGHA2/C1S/IGHV4-  59/IGHV5-51/IGLV2-  14/IGHG2/IGHV1-  45/IGHG1/IGHV4-  39/IGHE/PTPN6/IGHV3-  23/IGKC/IGLV2-23/IGHV3-  35/IGHV4-31/IGLV2-11/IGHV4-  34/IGKV3-15/IGLC7/IGHV3-  48/IGLV3-19/IGKV1-5/IGHV3-  11/IGHV1-69D/APCS/IGHV3-  21/IGLV1-44/IGHV1-  3/IGHG3/IGHV4-61/IGHV1-  18/IGLC6/C7/IGHV3-30/IGHV2-  70D/IGHV2-  5/IGLL5/IGLC3/C9/IGHG4/TRDC  /IGLV1-40/IGHV1-  58/IGHD/IGHV4-28/IGKV1-  17/IGHV1-24/IGHV3-  33/PTPRC/IGKV3D-11/IGHV2-  70/IGHV3-7/TRBC2/IGLV3-  25/IGLV7-43/IGHV3-20/IGLV3-  1/IGHV3-74/IGKV1-  16/C4B/IGHV3-13/C4A/IGHV4-  4/TNF/CRP/IGHV3-66/IGHV3-  72/IGHV1-69/SUSD4/IGKV5-  2/IGKV2D-28/IGKV2D-  30/IGKV1-12/IGHV3-  53/IGKV3D-20/IGHV6-  1/LTA/IGKV2-29/IGHV3- |
| --- | --- | --- | --- | --- |

| 0.497710826 9401 |  | tags=83%,  list=17%,  signal=69% |  | IGLC2/IGHA1/IGHM/IGHA2/IGHV  4-59/IGHV5-51/IGHG2/IGHV1-  45/IGHG1/IGHV4-  39/IGHE/IGHV3-  23/IGKC/IGHV3-35/IGHV4-  31/IGHV4-34/IGLC7/IGHV3-  48/IGHV3-11/IGHV1-  69D/IGHV3-21/IGHV1-  3/IGHG3/IGHV4-61/IGHV1-  18/IGLC6/JCHAIN/IGHV3-  30/IGHV2-70D/IGHV2-  5/IGLL5/IGLC3/IGHG4/TRDC/IGH  V1-58/IGHD/IGHV4-28/IGHV1-  24/IGHV3-33/IGHV2-70/IGHV3-  7/TRBC2/IGHV3-20/IGHV3-  74/IGHV3-13/IGHV4-4/IGHV3-  66/IGHV3-72/IGHV1-69/IGHV3-  53/IGHV6-1/IGHV3-64/IGHV3-  38/IGHV7-81/IGHV3-  43/TRBC1/IGHV3-16 |
| --- | --- | --- | --- | --- |

| 0.497710826 9242 |  | tags=56%,  list=16%,  signal=47% |  | ADAM22/LRRC4/GRID1/RGS7BP/  GSG1L/LRFN2/CHRM3/SHISA9/L  RFN5/GRIA1/PTPRO/LRRC4C/CA  CNG4/SHISA6/ADCY1/PLPPR4/N  ETO1/OPRD1/EPHA7/CACNG3/LR  RTM2/SLITRK1/CACNG7/DRD3/G  RIN1/GRID2/GRIN2A/SLITRK3/CA  CNG2/LRRTM3 |
| --- | --- | --- | --- | --- |

| 0.497710826 11224 |  | tags=57%,  list=20%,  signal=45% |  | EFNB2/GRIK5/ADORA1/SLC6A9/  P2RY1/DRD2/CADM3/GABRA5/E  PHA4/KCNA2/SYT11/CDH2/SLC  6A3/RGS7BP/PCDH17/SLC6A1/G  PM6A/KCNC1/GRM3/CDH9/CHR  M3/GPC4/EPHB2/PRRT2/NTNG2/  ADCY8/HTR2A/ADRA1A/P2RX1/  HTR1B/CHRNA4/CNTN5/NRXN1/  OPRD1/KCNJ3/KCNA3/SLC1A2/  CHRNA6/KCNJ9/DRD1/OPRM1/S  CN2A/HTR3A/GLRA1/KCNA1/CH  RM2/CPLX3 |
| --- | --- | --- | --- | --- |

| 0.497710826 12096 |  | tags=42%,  list=21%,  signal=33% |  | CACNA1F/RCVRN/USH1G/PRPH2  /DHRS3/CDHR1/EYS/RHO/ANO2  /RP1L1/GNAT1/DRD2/GLIS2/RRH  /GUCA1A/RGR/PTGS1/MAK/KIF  17/SPTBN5/PDE6H/PKD2L1/MAP  1B/TULP1/GRXCR1/RGS9BP/MCH  R1/GUCY2D/OPN1LW/USH1C/PR  OM1/SAG/PDE6A/USH2A/GPR83  /ATP8A2/ABCA4/PDC/GNGT1/  OPN4/PCARE/CNGB3/RD3/CNGA  1/GRK1/CNGB1/GUCA1C/DRD5/  CERKL/GRK7/RP1/OPN5/DRD1/S  STR3/PPEF2/GUCY2F/PCDH15/G  NAT2/OPN1MW/KNCN/NPY2R/C  ETN1 |
| --- | --- | --- | --- | --- |

| 0.497710826 13478 |  | tags=59%,  list=24%,  signal=45% |  | OPN3/ROM1/NXNL1/MYO7A/MY  RIP/CACNA1F/RCVRN/PRPH2/DH  RS3/CDHR1/EYS/RHO/RP1L1/GN  AT1/RRH/GUCA1A/RGR/PTGS1/  MAK/SPTBN5/PDE6H/MAP1B/TU  LP1/RGS9BP/GUCY2D/OPN1LW/  USH1C/PROM1/SAG/PDE6A/ATP  8A2/ABCA4/PDC/GNGT1/OPN4/  PCARE/CNGB3/RD3/CNGA1/GRK  1/CNGB1/GUCA1C/CERKL/GRK7  /RP1/OPN5/PPEF2/GUCY2F/PCD  H15/GNAT2/OPN1MW | |
| --- | --- | --- | --- | --- | --- |
| 0.497710826 258 |  | tags=20%,  list=0%,  signal=20% POR/MIR182 | | |  |

| 0.497710826 10452 |  | tags=36%,  list=18%,  signal=29% |  | C3/ADAM8/IL6/WNT5A/C2CD4  A/LILRA5/NPY5R/TNFRSF11A/SE  RPINE1/TNFSF4/TLR7/CCL3/PLA  2G2A/C2CD4B/SUCNR1/PTGS2/  CLEC7A/FFAR2/IL18/CD28/CD6  /F12/PTGER4/IL1B/ITGA2/PIK3C  G/NOD2/CCL24/CCR7/CCR2/IL1  5/LBP/FFAR3/STAP1/TAC1/TNF  /TTBK1/TNFSF11/LTA/GBP5/IL1  2B/TLR10/IDO1/CREB3L3/CCL1/  IL21/MMP8/IL17F/NLRP10/IL2/M  IR128-1 |
| --- | --- | --- | --- | --- |

| 0.497710826 14588 | |  | | tags=51%,  list=26%,  signal=38% |  | TMEM64/GAS6/TREM2/CASP8/T  YROBP/IL34/KITLG/CD74/BTK/N  FKBIZ/TGFB1/TRIB1/CD4/CSF1/  EGR3/HLA-  G/RUNX3/SYK/ANXA1/PRKCA/  LGALS9/TNFSF9/NLRP3/PIK3R6/  LEF1/CCR1/CD80/LILRB4/ADAM  8/VSIR/CA2/HCLS1/IL7/CD101/  TNFSF4/INPP5D/RUNX1/CD86/EV  I2B/IL1RL2/CCL19/FOXP3/NCKA  P1L/SASH3/DUSP10/GPR68/LILR  B2/ROR2/IL7R/IL18/GLI2/OCST  AMP/IL2RA/POU4F1/PTPRC/IL15  /MYB/LIF/GATA3/IL23R/DCSTA  MP/SHH/IL12RB1/CD27/TNF/ZA  P70/RASGRP1/TOX/TNFSF11/RH  OH/TESPA1/IL12B/IFNG/POU4F2  /IL2 | |
| --- | --- | --- | --- | --- | --- | --- | --- |
| 0.497710826 1205 |  | | tags=20%,  list=2%,  signal=20% PGK2/MYOG | | | |  |

| 0.497710826 10033 |  | tags=55%,  list=18%,  signal=46% |  | HRH4/DRD2/GABRA5/GRIN2D/GR  IN3A/CHRNA3/GRID1/GABRA4/G  ABRD/GLRB/GABRB2/GRM1/CHR  M3/GRIA1/GABRA3/GABRA2/CH  RNA4/GABRR2/GRIK1/CHRNA9/C  HRNA2/DRD5/GLRA2/GRIA4/ADR  B1/GABRA1/DRD3/GRIN1/GRID2  /GRIN2A/CHRNA6/GRIA3/DRD1/  GABRA6/GRM5/GABRG2/GRIA2/  GABRG1/GLRA1/CHRM2/CHRNB3  /OR56A5 |
| --- | --- | --- | --- | --- |

| 0.497710826 11224 |  | tags=43%,  list=20%,  signal=34% |  | EFNB2/GRIK5/UNC13A/ADORA1/  CNTNAP1/SLC6A9/P2RY1/FOSL1  /DRD2/GRM2/CADM3/GABRA5/  KCTD16/EPHA4/KCNA2/SYT11/S  NCAIP/CDH2/SLC6A3/RGS7BP/P  CDH17/SLC6A1/GPM6A/KCNC1/  GRM3/CDH9/SLC6A2/CHRM3/GP  C4/EPHB2/PRRT2/NTNG2/IGSF21  /ADCY8/HTR2A/SYT1/ADRA1A  /P2RX1/HTR1B/CNTNAP4/CHRNA  4/CNTN5/NRXN1/SNAP91/GRIK1  /OPRD1/KCTD8/KCNJ3/KCNA3/  PCDH8/SLC1A2/GRIN2A/CHRNA6  /IL31RA/KCNJ9/DRD1/RIMS2/OP  RM1/SCN2A/RIMS1/UNC13C/HT  R3A/KCNC2/GLRA1/KCNA1/CHR  M2/CPLX3 | |
| --- | --- | --- | --- | --- | --- |
| 0.497710826 204 |  | tags=20%,  list=0%,  signal=20% EFNB2/MIR153-1 | | |  |

| 0.497710826 11187 |  | tags=40%,  list=20%,  signal=32% |  | CD1D/NLRP3/PIK3R6/NCR1/HLA  -  F/RSAD2/C3/IL6/FCER2/IL27RA  /CD1C/WAS/TNFRSF1B/IFNB1/R  IPK3/TNFSF4/HAVCR2/CD1E/CD  1A/PTPN6/SH2D1B/IL18RAP/FOX  P3/VAV1/SASH3/CLC/IL7R/IL18  /CD28/IL10/CD160/CLEC4G/IL1  B/CADM1/CD1B/LILRB1/PTPRC/  CCR2/GATA3/IL23R/IL12RB1/TB  X21/NCR3/KIR2DL4/TNF/XCL1/  SLAMF6/RASGRP1/CD96/LAG3/  LTA/IL12B/KLRC4-  KLRK1/CRTAM/CLNK/SH2D1A/K  LRK1/IL21/IL2 |
| --- | --- | --- | --- | --- |
| 0.497710826 11399 |  | tags=33%,  list=20%,  signal=27% |  | DLG2/EFNB2/LRRC7/MX2/DNM3  /ADAM22/MAPK10/GPC6/CPT1C  /GSG1L/ARC/HPCA/GPC4/ITGB3  /CACNG4/CALY/SHISA6/FRRS1L  /CACNG3/CACNG7/DRD3/CACN  G2/GHSR |

| 0.497710826 13078 |  | tags=56%,  list=23%,  signal=43% |  | KCNAB3/CACNB2/KCNH3/CACN  A1C/CATSPER1/GRIN2C/CACNA  1H/CATSPER3/CACNA1F/CACNB  4/KCND3/CACNA2D1/KCNAB1/K  CNJ1/KCNT1/KCNMA1/KCNE1/K  CNE2/CACNG1/KCNK6/HCN2/KC  NF1/CACNB1/GRIN2D/GRIN3A/K  CNQ3/KCNJ8/KCNV1/LRRC38/K  CNV2/CACNG5/KCNA2/KCNK1/  KCNE1B/KCNH7/CACNA1E/CACN  A2D3/KCNC1/CACNA2D4/KCNH2  /KCNK9/HCN4/KCNJ10/KCNJ11/  KCNK18/KCNJ6/KCNT2/CALHM1  /LRRC55/KCNS2/HTR1B/CACNG4  /KCNQ2/KCNK3/KCNJ3/KCNA3/  CACNG3/KCNJ4/LRRC52/KCNH6  /CACNA1S/KCNB2/CACNG6/CA  CNG7/GRIN1/CACNA1G/GRIN2A  /KCNG4/TMC1/CACNA1I/KCNJ9  /KCNA4/KCNJ16/OPRM1/KCND2  /KCNH5/CACNG2/KCNC2/KCNA  1/KCNA10 |
| --- | --- | --- | --- | --- |

| 0.497710826 13353 |  | tags=44%,  list=24%,  signal=33% |  | ATP2A3/ANK2/TNNI3K/CACNB2  /CACNA1C/ATP1B2/NKX2-  5/GJA1/TRPM4/AGT/CACNA1F  /CACNB4/CASQ2/KCND3/NPR1/  ATP1A3/CACNA2D1/KCNE1/KCN  E2/GJC1/CACNG1/KCNK6/HRC/  CACNB1/CORIN/SCN1B/CACNG5  /SCN4B/CASQ1/ATP1A2/ABCC9  /SCN2B/FXYD7/KCNK1/KCNE1B  /NPPC/SLC8A1/CACNA2D3/CAC  NA2D4/KCNH2/ITPR2/HCN4/KCN  J11/GJA5/CACNG4/PLN/ATP2B2  /KCNK3/KCNJ3/CACNG3/KCNJ4  /NOS1/FXYD2/RYR2/CACNA1S/  CACNG6/CACNG7/TRDN/CACNA  1G/SLC8A3/KCND2/CACNG2/MI  R208A |
| --- | --- | --- | --- | --- |

| 0.497710826 9494 |  | tags=84%,  list=17%,  signal=70% |  | 8/IGHA1/IGHM/IGHA2/IGHV4-  59/IGHV5-51/IGLV2-  14/IGHG2/IGHV1-  45/IGHG1/IGHV4-  39/IGHE/IGHV3-  23/IGKC/IGLV3-  9/CD79A/IGLV4-69/IGLV2-  23/IGHV3-35/IGHV4-31/IGLV2-  11/IGHV4-34/IGKV3-  15/IGLC7/IGHV3-48/IGLV5-  45/IGLV3-19/IGKV1-5/IGHV3-  11/IGHV1-69D/IGHV3-  21/IGLV1-44/IGHV1-  3/IGHG3/IGHV4-61/IGHV1-  18/IGLC6/JCHAIN/IGLV8-  61/IGHV3-30/IGHV2-  70D/IGHV2-5/IGLL5/IGKV1-  9/IGLV4-  60/IGLC3/IGHG4/TRDC/IGLV1-  40/IGHV1-58/IGHD/IGLV7-  46/IGHV4-28/IGKV1-17/IGHV1-  24/IGLV3-10/IGHV3-  33/IGKV3D-11/IGLV10-  54/IGHV2-70/IGHV3-  7/TRBC2/IGKV2-24/IGKV1-  27/IGLV3-25/IGLV7-43/IGLV9-  49/IGHV3-20/IGLV3-1/IGLV2-  18/IGHV3-74/IGKV1-16/IGHV3-  13/IGHV4-4/IGHV3-66/IGKV1-  8/IGHV3-72/IGHV1-69/IGKV1-  6/IGKV1D-43/IGKV5-  2/IGKV2D-28/IGKV2D-  30/IGKV1-12/IGHV3- |
| --- | --- | --- | --- | --- |

| 0.497710826 8176 |  | tags=47%,  list=14%,  signal=40% |  | 69/SASH3/IGLV2-23/IGLV2-  11/IGKV3-  15/TNFRSF4/TRAV38-  1/IL7R/IL13/IGLV5-45/IGLV3-  19/IGKV1-5/CD28/IL10/IGLV1-  44/FGL2/IGLV8-  61/CD40LG/POU2F2/IGKV1-  9/IGLV4-60/TNFSF13B/IGKV2D-  24/IGLV1-40/IGLV7-  46/BATF/IGKV1-17/IGLV3-  10/PTPRC/IGKV3D-11/IGLV10-  54/CARD11/TRAV9-2/IGKV2-  24/IGKV1-27/IGLV3-25/IGLV7-  43/IGLV9-49/IGLV3-1/IGLV2-  18/TBX21/IGKV1-  16/TRAV40/IGLV1-  50/GCNT3/TNF/IGKV1-  8/XCL1/IGKV1-  6/MZB1/IGKV1D-  43/TRAV18/IGKV5-2/IGKV2D-  28/IGKV2D-30/IGKV1-  37/IGKV1-  12/AICDA/TRAV2/IGKV3D-  20/IGKV2-  29/TRAV14DV4/IGKV1D-  12/IGKV2-30/IGKV3D-  7/IGKV1D-  17/CCR6/TRDV1/IGKV1D-  8/IGKV3D-15/IGLV3-  12/TRAV19/IGKV2D-  29/LAX1/IGKV1D-39/IGKV1-  39/IGLV3-27/IGLV5-48/IGKV1-  13/IGKV1D-33/IGLV1- |
| --- | --- | --- | --- | --- |

| 0.497710826 12288 |  | tags=40%,  list=22%,  signal=31% |  | MEIS2/AGT/C1QL1/PAK6/ASIC1  /HRH1/NRGN/NTSR1/CCK/SHAN  K1/HRH2/FOSL1/DRD2/BRSK1/A  DGRB3/PDE1B/DKK1/LRRN4/GAB  RA5/NPTX2/STRA6/NLGN4Y/AT  P1A2/SYT11/NRXN2/NLGN4X/PP  P1R1B/PTGS2/GPR88/SLC6A1/N  DRG4/ADAM2/ARC/SLC7A11/M  AP1A/RELN/EPHB2/EN1/PAK5/N  RXN3/NETO1/CNTN2/NRXN1/DR  D5/TAC1/CRH/FOXB1/SLC24A2  /DRD3/GRIN1/TNR/GRIN2A/CNT  NAP2/DRD1/NEUROD2/SLC8A3/  GRM5/TBR1 |
| --- | --- | --- | --- | --- |

| 0.497710826 13358 |  | tags=47%,  list=24%,  signal=36% |  | GPR35/ATP2A3/ANK2/TNNI3K/C  ACNB2/S1PR1/CACNA1C/ATP1B  2/NKX2-  5/GJA1/TRPM4/CACNA1H/AGT  /CACNA1F/CACNB4/CASQ2/KC  ND3/NPR1/ATP1A3/CACNA2D1/  KCNE1/KCNE2/TYMP/GJC1/CAC  NG1/CLDN19/KCNK6/CNTNAP1/  KCNMB4/SCN4A/AVPR1A/HRC/D  MRT3/FGF12/NFASC/CACNB1/C  ORIN/SCN1B/CACNG5/SCN4B/C  ASQ1/KCNA2/ATP1A2/ABCC9/  SCN2B/FXYD7/KCNK1/SCN9A/K  CNE1B/NPPC/GPR88/SLC8A1/CA  CNA2D3/CACNA2D4/KCNH2/ITPR  2/HCN4/KCNJ11/ITGA2/GJA5/S  CN7A/NTRK3/P2RX1/CACNG4/P  LN/ATP2B2/KCNK3/KCNJ3/CAC  NG3/DRD5/KCNJ4/NOS1/FXYD2  /RYR2/CACNA1S/CACNG6/CAC  NG7/TRDN/TNR/CACNA1G/CAC  NA1I/CNTNAP2/DRD1/SCN1A/S  CN2A/SLC8A3/CARTPT/KCND2/  SCN3A/CACNG2/GLRA1/KCNA1  /GHSR/MIR208A |
| --- | --- | --- | --- | --- |

| 0.497710826 11558 |  | tags=41%,  list=20%,  signal=32% |  | UCN3/CRHBP/GRIK5/NTSR1/CCK  /UNC13A/ADORA1/PRKCB/GCH1  /SLC18A1/UCHL1/DRD2/CRHR2/  PENK/SLC18A2/EPHA4/KCNA2/S  YT11/TULP1/PACSIN1/CASR/KC  NC1/PDYN/CHRM3/CALCA/ITGA  2/PRRT2/USH2A/SCRG1/ROR1/P  TPRN/ADCYAP1/SYT1/CALB1/H  TR1B/PRKCG/SYT4/CNGB1/ATC  AY/OPRD1/KCNA3/NOS1/CABP4  /CPLX4/CPLX2/GRIN1/GAD1/SL  C32A1/PNOC/SLC4A10/UNC13C  /KCNC2/SLC17A8/GLRA1/KCNA  1/CHRM2/CPLX3 |
| --- | --- | --- | --- | --- |

| 0.497710826 7476 |  | tags=55%,  list=13%,  signal=48% |  | HRH1/GABRG3/HTR7/GRIK5/GAB  RB3/OR11H7/ADORA1/HRH2/HRH  4/DRD2/GABRA5/GRIN2D/GRIN3  A/HTR4/CHRNA3/GRID1/GABRA  4/GABRD/GLRB/GABRB2/GRM1/  CHRM3/GABBR2/GRIA1/HTR1D/H  TR2A/HTR1B/GABRA3/GABRA2/  CHRNA4/GABRR2/GRIK1/CHRNA9  /HTR2B/CHRNA2/DRD5/GLRA2/  GRIA4/ADRB1/GABRA1/DRD3/GR  IN1/GRID2/GRIN2A/HTR1F/CHRN  A6/GRIA3/DRD1/OPRM1/HTR5A  /GLRA3/GABRA6/GRM5/GABRG2  /GRIA2/HTR3A/GABRG1/GLRA1  /CHRM2/CHRNB3/HTR1E/OR5T2/  OR56A5/HTR2C |
| --- | --- | --- | --- | --- |

| 0.497710826 12355 |  | tags=41%,  list=22%,  signal=32% |  | IL1RL1/FGR/SYK/CSF1R/CRLF2/  AIF1/LGALS9/F2R/NLRP3/OSM/  ORM1/IL1A/WNT5A/DRD2/USP5  0/CD244/LILRA5/CLEC4E/HAVC  R2/PANX2/CASP1/CCL3/CCL19  /SORL1/CHIA/FFAR2/TWIST1/IL  10/CD160/PTGER4/IL1B/CADM1  /NOD2/IGHD/TLR8/PAEP/GATA3  /IL26/CD2/HTR2B/TNF/NLRP7/R  ASGRP1/PTPN22/ORM2/CLEC9A  /MMP12/AIM2/AGTR2/CRTAM/  HMGB4/CCL1/MMP8/IFNG/IL17F  /NLRP10/MIR182 |
| --- | --- | --- | --- | --- |

| 0.497710826 11361 |  | tags=44%,  list=20%,  signal=35% |  | GABRG3/P2RX2/GRIK5/NTSR1/P2  RX6/GABRB3/ADORA1/SHANK1/  DRD2/GABRA5/GRIN2D/GRIN3A/  SLC17A7/CHRNA3/GRID1/RGS7B  P/NLGN4X/P2RX5/CELF4/GABRA  4/GABRD/GLRB/GABRB2/RELN/G  RM1/GRIA1/SEZ6/INSYN2A/ADC  YAP1/P2RX1/GABRA3/NETO1/G  ABRA2/CHRNA4/GABRR2/NRXN1  /GRIK1/CUX2/CHRNA9/CHRNA2  /GLRA2/GRIA4/ADRB1/GABRA1  /GRIN1/GRID2/GRIN2A/CHRNA6/  GRIA3/RIMS2/OPRM1/GLRA3/RI  MS1/GABRA6/GRM5/GABRG2/K  CND2/GRIA2/GABRG1/GLRA1/K  CNA1/CHRNB3/NPY2R |
| --- | --- | --- | --- | --- |

| 0.497710826 12577 | |  | | tags=49%,  list=22%,  signal=38% | |  | | DTX1/EGR3/HLA-  G/RUNX3/NRARP/FOXJ1/JAK3/  SYK/LOXL3/CR1/ANXA1/LGALS  9/TNFSF9/NLRP3/PIK3R6/CD80/  LILRB4/ADAM8/VSIR/IL7/IFNB1/  TNFSF4/RUNX1/CD86/IL1RL2/CC  L19/FOXP3/ZEB1/NCKAP1L/SAS  H3/DUSP10/LILRB2/HLA-  DOA/IL7R/IL18/CD28/PRDM1/GL  I2/FGL2/IFNL1/NFATC2/IL2RA/T  NFRSF18/IRF1/ZNF683/PTPRC/IL  27/CCR2/CARD11/IL15/MYB/G  ATA3/IL23R/CD2/SHH/IL12RB1/  CD27/TBX21/ZAP70/RASGRP1/S  PINK5/LAG3/RHOH/TESPA1/IL12  B/CTLA4/IFNG/IL2 |
| --- | --- | --- | --- | --- | --- | --- | --- | --- |
| 0.497710826 6410 |  | | tags=22%,  list=11%,  signal=20% | |  | | CADPS/PRRT2/HTR2A/SYT1/AD  RA1A/P2RX1/HTR1B/SYT5/PRKC  G/ADCY1/SYT4/CHRNA4/SYT6/  SYT9/SYT2/CPLX4/CPLX2/CHRN  A6/DRD1/RIMS2/PPFIA2/RIMS1/  UNC13C/CHRM2/CHRNB3/CPLX3 | |

| 0.497710826 12415 |  | tags=44%,  list=22%,  signal=34% |  | JAK3/LOXL3/ANXA1/LGALS9/G  PR183/NLRP3/LEF1/CD80/HLA-  DMB/IL6/BCL3/TNFSF18/CD1C/  LCP1/IFNB1/CLEC4E/TNFSF4/HA  VCR2/CD86/CCL19/FOXP3/IFNW  1/APBB1IP/IL18/IFNE/FGL2/IFNL  1/PTGER4/EOMES/LILRB1/BATF/  ITGAL/IL27/LY9/MYB/GATA3/I  L23R/IL12RB1/SPN/TBX21/SLAM  F6/IL12B/IFNG/IFNA10/IFNA21/I  L2 |
| --- | --- | --- | --- | --- |

| 0.497710826 12096 |  | tags=58%,  list=21%,  signal=46% |  | CACNA1F/RCVRN/RPE65/EYS/RH  O/GNAT1/RRH/GUCA1A/RGR/SE  MA5B/PDE6C/TULP1/RGS9BP/AI  PL1/GUCY2D/GPR88/OPN1LW/EL  OVL4/CACNA2D4/GNGT2/SAG/  PDE6A/GRM6/ATP8A2/ABCA4/P  DC/GNGT1/OPN4/CNGA1/GRK1/  CNGB1/GUCA1C/NR2E3/TRPC3/  GRK7/CABP4/RP1/OPN5/ASIC2/  GJA10/GUCY2F/GNAT2/OPN1M  W |
| --- | --- | --- | --- | --- |

| 0.497710826 13600 |  | tags=55%,  list=24%,  signal=42% |  | SEMA5A/PITX1/SEMA3A/FGF8/  CDH1/OTP/TBX19/NRP1/MSX1/  NKX2-  1/PAX6/TAL2/GLI1/WNT5A/DR  D2/NOG/NR0B1/POU3F2/ARX/S  LC6A3/SOX2/PTCHD1/KCNC1/R  AX/GLI2/OLIG2/SOX3/GBX2/PO  U4F1/ADCYAP1/HAP1/SHH/FGF1  0/ISL1/WNT1/CRH/FOXB1/LHX3  /CNTNAP2/KCNC2/NKX2-6 |
| --- | --- | --- | --- | --- |

| 0.497710826 14055 |  | tags=42%,  list=25%,  signal=32% |  | CXCR4/NECTIN1/EGFR/CD4/CDH  R3/MRC1/CR1/EFNB2/CD80/CLD  N6/SELPLG/ITGA5/NCAM1/DPP4  /CD86/CLDN1/ANPEP/TNFRSF4/  CD209/CLDN9/CLEC4G/ITGA2/IT  GB3/HTR2A/NECTIN4/SLC52A1/  CCR5/GPR15/SLAMF1/HAVCR1/  CR2 |
| --- | --- | --- | --- | --- |

| 0.497710826 16889 |  | tags=65%,  list=30%,  signal=45% |  | PKD2/CAPN3/CACNB3/SLC9A1/  S100A1/GPER1/F2/BAX/CX3CL1  /RAMP3/STAC3/ATP2A1/PLCG2  /AKAP6/EHD3/ATP1B1/STAC2/  ANK2/CACNB2/GJC2/FGF14/TH  Y1/CEMIP/CACNA2D1/F2R/NTSR  1/CRACR2A/KCNE2/BDKRB1/CD  19/F2RL3/RGN/APLNR/CASQ1/S  TAC/IL13/P2RY6/HAP1/CXCL10  /CXCL11/XCL1/CXCL9/RYR2/T  RDN/CALCR/DRD1 |
| --- | --- | --- | --- | --- |

| 0.497710826 12166 |  | tags=45%,  list=22%,  signal=35% |  | SYK/ANXA1/LGALS9/TNFSF9/N  LRP3/PIK3R6/CD80/LILRB4/ADA  M8/VSIR/IL7/TNFSF4/INPP5D/RU  NX1/CD86/IL1RL2/CCL19/FOXP3  /NCKAP1L/SASH3/DUSP10/LILRB  2/IL7R/IL18/GLI2/IL2RA/PTPRC/  IL15/MYB/GATA3/IL23R/SHH/IL  12RB1/CD27/ZAP70/RASGRP1/T  OX/RHOH/TESPA1/IL12B/IFNG/I  L2 | |
| --- | --- | --- | --- | --- | --- |
| 0.497710826 10854 |  | tags=31%,  list=19%,  signal=25% |  | UNC13A/ADORA1/CNTNAP1/P2R  Y1/BRSK1/SLC17A7/SYT11/CDH  2/GUCY1B1/GPM6A/NTNG2/AD  CY8/P2RX1/NRXN1/GRM7/GAD1  /RIMS2/SLC32A1/PPFIA2/RIMS1  /UNC13C/CPLX3 |  |

| 0.497710826 10854 |  | tags=50%,  list=19%,  signal=40% |  | UNC13A/ADORA1/SHANK1/HOM  ER1/DRD2/GRM2/DKK1/KMO/OX  TR/ATP1A2/CDH2/PTGS2/ROR2  /GRM3/RELN/GRM1/GRM6/NTRK  1/HTR2A/ADCYAP1/SYT1/HTR1  B/CCR2/GRM4/NRXN1/GRM8/GR  IK1/TNF/DRD3/TNR/DRD1/GRM7  /GRM5/NPY2R/OR56A5 |
| --- | --- | --- | --- | --- |

| 0.497710826 10837 |  | tags=49%,  list=19%,  signal=39% |  | TYMP/CLDN19/CNTNAP1/KCNMB  4/SCN4A/AVPR1A/DMRT3/FGF1  2/NFASC/SCN1B/KCNA2/SCN9A  /GPR88/ITGA2/SCN7A/NTRK3/P  2RX1/CACNG4/CACNG3/DRD5/T  NR/CACNA1G/CACNA1I/CNTNAP  2/DRD1/SCN1A/SCN2A/CARTPT  /KCND2/SCN3A/CACNG2/GLRA1  /KCNA1/GHSR |
| --- | --- | --- | --- | --- |

| 0.497710826 11030 |  | tags=42%,  list=20%,  signal=34% |  | AC021066.1/SPRR2D/KRT17/ST1  4/KLK5/CSTA/SPRR2A/KRT80/S  PRR1A/TCHH/PI3/KRT13/DSG4/  SPRR1B/CELA2A/SPRR3/KRT36/K  RT6A/KLK12/DSC3/KRT5/IVL/K  RT6B/LCE1A/KRT79/TGM5/SPIN  K6/KRT3/KRT12/DSC1/CASP14/  KRT16/SPINK5/KRT24/SPRR2G/K  RT75/KRT71/KRT6C/DSG1/KRT4  0/LIPM/KRT74/KRT37/KRT85/KR  T26/KRT83/KRT84/KRT33B |
| --- | --- | --- | --- | --- |

| 0.497710826 11178 |  | tags=41%,  list=20%,  signal=33% |  | MIR26A2/CARD9/CD80/CYBB/IL  1A/IL6/THBS1/CEBPE/BCL3/WN  T5A/KLF4/INHBB/PTAFR/INPP5D  /TLR7/CD86/PCSK5/FOXP3/CM  A1/MIR766/CLEC7A/IL18/EBI3/  CD28/LTB/IL10/IL1B/LILRB1/IRF  1/TLR8/IGF2BP3/PTPRC/TNFSF15  /IL27/CCR2/CARD11/GATA3/C  D3E/LBP/SPN/TNF/APOA2/TNFR  SF8/LAG3/IL12B/IL21/IFNG/GHS  R/IL17F/MIR204/MIR181A1 |
| --- | --- | --- | --- | --- |

| 0.497710826 12046 |  | tags=56%,  list=21%,  signal=44% |  | SV2A/CLSTN3/KCND3/GABRG3/  GABRB3/GUCY1A1/DRD2/GABRA  5/NLGN4Y/IQSEC3/NLGN4X/PCD  H17/MDGA1/SLITRK2/NRG1/SLC  6A1/GABRA4/GABRD/GLRB/GAB  RB2/LRFN5/PTPRO/LRRTM1/ACA  N/CALB1/ADRA1A/GABRA3/GA  BRR2/ATP2B2/CNTN5/NRXN1/LR  RTM2/SLITRK1/GABRA1/DRD3/D  RD1/SLC32A1/GABRG2/KCND2/  SLITRK3 |
| --- | --- | --- | --- | --- |

| 0.497710826 13673 |  | tags=63%,  list=24%,  signal=48% |  | IFNAR2/CD4/HLA-  DRA/GPR35/F3/ACKR4/FCER1G/  MR1/IL17REL/GPR75/IL1RL1/CR1  /CX3CR1/CRLF2/C3AR1/HLA-  DPA1/CCR10/CCR1/KRT17/CMK  LR1/IL20RA/C5AR2/IL1RAPL2/HL  A-  DQA2/CSF3R/PRLR/IL27RA/HLA  -  DQB1/CCRL2/IL1RL2/IL18RAP/LE  PR/HLA-DQA1/HLA-  DQB2/IL10RA/GFRA1/LILRB2/HL  A-  DOA/CSF2RB/IL7R/EBI3/CSF2RA  /GFRA2/CD160/IL2RG/CCR9/IL2  RA/IL15RA/LILRB1/IL2RB/KIR3DL  1/IL9R/CCR7/IL1R2/CCR2/CCR5  /IL23R/CD200R1/FCGR1B/IL22RA  1/FLT3/HLA-  DOB/IL12RB1/KIR2DL4/CCR3/CX  CR3/CCR4/CXCR6/PIGR/IL12B/I  L31RA/KLRC4-  KLRK1/CCR6/IL21R/XCR1/CXCR  5/KLRK1/IL22RA2/CR2/CCR8 |
| --- | --- | --- | --- | --- |

| 0.497710826 11604 |  | tags=62%,  list=21%,  signal=50% |  | KCND3/CLSTN2/EFNB2/GRIK5/P2  RX6/ADORA1/SLC6A9/DRD2/GR  M2/GABRA5/ADAM22/CACNG5/  EPHA4/LRRC4/GRID1/CDH2/SLC6  A3/RGS7BP/PCDH17/SLITRK2/GS  G1L/SLC8A1/SLC6A1/LRFN2/GA  BRA4/GABRD/KCNC1/GRM3/CDH  9/GABRB2/CHRM3/SHISA9/LRFN  5/GRIA1/EPHB2/PTPRO/LRRTM1/  LRRC4C/NTRK3/HTR2A/ADRA1A  /P2RX1/CACNG4/GABRA3/SHIS  A6/ADCY1/PLPPR4/NETO1/CNTN  2/OPRD1/CHRNA9/KCNA3/EPHA  7/CACNG3/PCDH8/LRRTM2/SLIT  RK1/CACNG7/GABRA1/DRD3/GR  IN1/GRID2/GRIN2A/CHRNA6/DRD  1/OPRM1/HTR5A/SLC8A3/KCND  2/HTR3A/SLITRK3/CACNG2/GLR  A1/LRRTM3/KCNA1/CHRM2 |
| --- | --- | --- | --- | --- |

| 0.497710826 9501 |  | tags=53%,  list=17%,  signal=44% |  | GABRA5/ADAM22/LRRC4/GRID1  /CDH2/RGS7BP/GSG1L/LRFN2/G  ABRA4/GABRB2/CHRM3/SHISA9  /LRFN5/GRIA1/PTPRO/LRRTM1/L  RRC4C/CACNG4/GABRA3/SHISA  6/ADCY1/PLPPR4/NETO1/OPRD1  /CHRNA9/EPHA7/CACNG3/LRRT  M2/SLITRK1/CACNG7/GABRA1/  DRD3/GRIN1/GRID2/GRIN2A/HTR  5A/KCND2/SLITRK3/CACNG2/GL  RA1/LRRTM3 |
| --- | --- | --- | --- | --- |

| 0.497710826 12358 | |  | | tags=44%,  list=22%,  signal=35% |  | ELANE/DNASE1L3/UNC13D/LGAL  S9/CD1D/PIK3R6/NCR1/ULBP2/H  LA-  F/CD1C/RIPK3/GZMM/HAVCR2/  CD1E/CORO1A/CD1A/PTPN6/SL  AMF7/IL18RAP/VAV1/IL7R/IL18  /ULBP1/CD160/CTSG/CADM1/P  RF1/CD1B/LILRB1/KIR3DL1/PTPR  C/GZMB/IL23R/IL12RB1/NCR3/K  IR2DL4/STAP1/XCL1/SLAMF6/R  ASGRP1/LAG3/IL12B/KLRC4-  KLRK1/CRTAM/SH2D1A/KLRK1/I  L21 | |
| --- | --- | --- | --- | --- | --- | --- | --- |
| 0.497710826 3340 |  | | tags=20%,  list=6%,  signal=19% KCND2/MC3R/NMS | | | |  |

| 0.497710826 13999 |  | tags=40%,  list=25%,  signal=30% |  | CD274/CD74/BTK/BCL2L11/LGA  LS3/LYN/TSC22D3/JAK3/TNFRS  F21/ARG2/LGALS9/GIMAP8/AD  AM8/WNT5A/RIPK3/CLC/CCL5/  IL7R/IL10/IL2RA/LGALS13/CD27  /HSH2D/IDO1/CD3G/FASLG/PDC  D1/LGALS14/IL2 |
| --- | --- | --- | --- | --- |
| 0.497710826 12960 |  | tags=54%,  list=23%,  signal=41% |  | RET/S1PR1/AIRE/ARTN/CXCL12  /CCL18/AIF1/CCL14/GPR183/C  CL20/CCL13/ADAM8/WNT5A/IL  27RA/CCL7/CCL8/RIPK3/GCSAM  L/SPNS2/ADTRP/CCL3/CCL19/C  CL23/CCL11/CCL5/ECM1/CCL3L  3/MYO1G/CCL16/PIK3CG/C10orf  99/CCL21/CCL25/CCL24/ITGAL  /CCR7/CCL26/CCR2/CCL4/GAT  A3/CD200R1/GCSAM/CXCL10/S  PN/CXCL11/TBX21/TNFSF14/ITG  A4/CXCR3/ZAP70/XCL1/GPR15  /CCL22/XCL2/S100A7/KLRC4-  KLRK1/CCR6/CXCL13/CCL1/KLR  K1 |

| 0.497710826 10583 |  | tags=57%,  list=19%,  signal=46% |  | ARHGAP25/C3/IGHV3-  73/THBS1/MSR1/XKR5/IGLC2/IG  HA1/ELMO1/IGHM/IGHA2/IGHV4  -59/RAB31/IGHV5-  51/IGHG2/SYT11/IGHV1-  45/IGHG1/IGHV4-  39/IGHE/IGHV3-  23/IGKC/NCKAP1L/ALOX15/IGH  V3-35/CD300A/IGHV4-  31/IGHV4-34/IGLC7/IGHV3-  48/IGHV3-11/IGHV1-  69D/IGHV3-21/IGHV1-  3/IGHG3/IGHV4-61/IGHV1-  18/IGLC6/IGHV3-30/IGHV2-  70D/IGHV2-  5/IGLL5/BIN2/IGLC3/ITGA2/IGH  G4/TRDC/FCGR1A/IGHV1-  58/IGHD/IGHV4-28/IGHV1-  24/IGHV3-33/XKR4/IGHV2-  70/IGHV3-7/TRBC2/IGHV3-  20/IGHV3-74/IGHV3-13/IGHV4-  4/STAP1/IGHV3-66/IGHV3-  72/IGHV1-69/IGHV3-  53/XKR7/IGHV6-1/IGHV3-  64/IGHV3-38/IGHV7-81/IGHV3-  43/TRBC1/IGHV3-16 |
| --- | --- | --- | --- | --- |

| 0.497710826 9401 |  | tags=77%,  list=17%,  signal=64% |  | IGLC2/IGHA1/IGHM/IGHA2/IGHV  4-59/IGHV5-51/IGHG2/IGHV1-  45/IGHG1/TULP1/IGHV4-  39/IGHE/IGHV3-  23/IGKC/CLEC7A/IGHV3-  35/IGHV4-31/IGHV4-  34/IGLC7/IGHV3-48/IGHV3-  11/IGHV1-69D/IGHV3-  21/IGHV1-3/IGHG3/IGHV4-  61/IGHV1-18/IGLC6/IGHV3-  30/IGHV2-70D/IGHV2-  5/IGLL5/IGLC3/IGHG4/TRDC/IGH  V1-58/IGHD/IGHV4-28/IGHV1-  24/IGHV3-33/IGHV2-70/IGHV3-  7/TRBC2/IGHV3-20/IGHV3-  74/IGHV3-13/IGHV4-4/IGHV3-  66/FCN2/IGHV3-72/IGHV1-  69/IGHV3-53/IGHV6-1/IGHV3-  64/IGHV3-38/IGHV7-81/IGHV3-  43/TRBC1/IGHV3-16 |
| --- | --- | --- | --- | --- |

| 0.497710826 12143 |  | tags=55%,  list=21%,  signal=43% |  | AGT/CX3CR1/PTGER3/C3AR1/H  RH1/ANO1/CHGA/F2R/CMKLR1/  C5AR2/P2RY2/HOMER1/TRHR/P2  RY1/GNA15/FPR3/DRD2/P2RY8/  F2RL3/ADRA2A/ADRA1B/F2RL2/  CASR/GNG13/CCKAR/ESR1/KISS  1/GRM1/FPR1/NPR3/GPR20/CAL  CA/GRP/GPR65/CCKBR/NMUR2/  P2RY6/NMBR/HTR2A/TACR1/AD  RA1A/FPR2/OPRD1/HTR2B/DRD5  /DRD3/GPR18/DRD1/HCRTR2/OP  RM1/GPR174/GPR55/GRM5/P2RY  10/CHRM2/OR56A5/HTR2C/GPR3  2P1/MC3R |
| --- | --- | --- | --- | --- |

| 0.497710826 14175 |  | tags=44%,  list=25%,  signal=33% |  | ZP3/FAM49B/CD274/BTK/NFKBIZ  /CLCF1/TGFB1/CD4/HLA-  G/FCER1G/HLA-  H/ANXA1/CD1D/NLRP3/CD80/H  LA-  F/RSAD2/C3/IL6/FCER2/IL27RA  /CD1C/TNFSF4/CD1E/CD1A/CC  L19/FOXP3/SASH3/IL18/CD28/I  L1B/TNFSF13B/CD1B/PTPRC/CCR  2/GATA3/IL23R/IL12RB1/TBX21  /SKAP1/TNF/XCL1/LTA/IL12B/  NLRP10/IL2 |
| --- | --- | --- | --- | --- |

| 0.497710826 9488 |  | tags=55%,  list=17%,  signal=46% |  | IL7/IGLC2/IGHA1/TNFSF4/IGHM  /INPP5D/IGHA2/IGHV4-  59/IGHV5-51/IGHG2/IGHV1-  45/IGHG1/IGHV4-  39/IGHE/IGHV3-  23/IGKC/NCKAP1L/SASH3/IGHV  3-35/IGHV4-31/IGHV4-  34/IGLC7/IGHV3-  48/TNFRSF4/IL13/CD28/IGHV3-  11/IGHV1-69D/IGHV3-  21/IGHV1-3/IGHG3/IGHV4-  61/IGHV1-  18/IGLC6/NFATC2/IGHV3-  30/IGHV2-70D/IGHV2-  5/IGLL5/IGLC3/TNFSF13B/IGHG4  /NOD2/TRDC/IGHV1-  58/IGHD/IGHV4-28/IGHV1-  24/IGHV3-  33/PTPRC/CARD11/IGHV2-  70/IGHV3-  7/CD38/TRBC2/IGHV3-  20/CD27/TBX21/IGHV3-  74/IGHV3-13/IGHV4-4/IGHV3-  66/IGHV3-72/IGHV1-69/IGHV3-  53/IGHV6-1/IGHV3-64/IGHV3-  38/IGHV7-81/IGHV3-  43/TRBC1/IL21/FCRL3/IGHV3-  16/IL2 |
| --- | --- | --- | --- | --- |

| 0.497710826 14507 |  | tags=56%,  list=26%,  signal=41% |  | NLGN3/STAC3/TREM2/PLCG2/A  KAP6/EHD3/STAC2/ATPSCKMT/  CTSS/ANK2/CACNB2/GALR2/FG  F14/CACNA2D1/NTSR1/CRACR2  A/KCNE2/RGS9/NOS1AP/LRRC38  /CASQ1/STAC/ARC/KCNC1/RE  LN/KCNJ11/EPHB2/P2RY6/LRRC5  5/HAP1/CCR2/CACNG4/PIRT/C  ACNG3/LRRC52/TRDN/CACNG2/  KCNC2/KCNA1/IFNG |
| --- | --- | --- | --- | --- |

| 0.497710826 11187 |  | tags=37%,  list=20%,  signal=29% |  | CD1D/NLRP3/CD177/HLA-  F/RSAD2/C3/IL6/FCER2/CD1C/  PTAFR/TNFSF4/CD1E/CD1A/SH2  D1B/IL18RAP/FOXP3/VAV1/SCI  MP/SASH3/IL13/IL18/CD28/CD1  60/IL1B/CADM1/NOD2/CD1B/PT  PRC/GATA3/IL23R/IL12RB1/TBX  21/NCR3/KIR2DL4/TNF/XCL1/SL  AMF6/RASGRP1/LAG3/LTA/IL12  B/KLRC4-  KLRK1/CRTAM/CLNK/SH2D1A/K  LRK1/IL21/IL2 |
| --- | --- | --- | --- | --- |

| 0.497710826 15068 | |  | | tags=48%,  list=27%,  signal=35% |  | NLGN3/STAC3/ATP2A1/WNK2/T  REM2/PLCG2/AKAP6/EHD3/ATP1  B1/STAC2/ABCB1/ATPSCKMT/C  TSS/ANK2/CACNB2/GALR2/ATP  1B2/SYNGR3/FGF14/CACNA2D1  /NTSR1/CRACR2A/GLRX/SGK1/  KCNE2/RGS9/NOS1AP/RGN/LRRC  38/PTAFR/CASQ1/PON1/STAC/  TRPC6/ARC/KCNC1/RELN/KCNJ1  1/EPHB2/P2RY6/LRRC55/HAP1/  CCR2/CACNG4/PIRT/CACNG3/R  YR2/LRRC52/TRDN/HTR3A/CAC  NG2/KCNC2/KCNA1/IFNG | |
| --- | --- | --- | --- | --- | --- | --- | --- |
| 0.497710826 2835 |  | | tags=25%,  list=5%,  signal=24% APOC2/PNLIP/MIR182 | | | |  |

| 0.497710826 11434 |  | tags=56%,  list=20%,  signal=45% |  | CLSTN2/DLG2/CNKSR2/EFNB2/G  RIK5/RGS9/GRIN2D/GRIN3A/ADA  M22/LRRC4/GRID1/RGS7BP/GSG  1L/LRFN2/ARC/GRM1/CHRM3/S  HISA9/LRFN5/GRIA1/PTPRO/LRR  C4C/CACNG4/SHISA6/ADCY1/P  LPPR4/NETO1/ATP2B2/GRIK1/OP  RD1/EPHA7/CACNG3/GRIA4/LRR  TM2/SLITRK1/CACNG7/DRD3/GR  IN1/GRID2/GRIN2A/GRIA3/GRM5  /GRIA2/SLITRK3/CACNG2/LRRT  M3 |
| --- | --- | --- | --- | --- |

| 0.497710826 11604 |  | tags=55%,  list=21%,  signal=43% |  | KCND3/CLSTN2/DLG2/CNKSR2/E  FNB2/GRIK5/P2RX6/RGS9/GABR  A5/GRIN2D/GRIN3A/ADAM22/LR  RC4/GRID1/CDH2/RGS7BP/GSG1  L/LRFN2/GABRA4/ARC/GABRB2  /GRM1/CHRM3/SHISA9/LRFN5/  GRIA1/PTPRO/LRRTM1/LRRC4C/  CACNG4/GABRA3/SHISA6/ADCY  1/PLPPR4/NETO1/ATP2B2/GRIK1  /OPRD1/CHRNA9/EPHA7/CACNG  3/GRIA4/LRRTM2/SLITRK1/CAC  NG7/GABRA1/DRD3/GRIN1/GRID  2/GRIN2A/GRIA3/HTR5A/GRM5/  KCND2/GRIA2/SLITRK3/CACNG2  /GLRA1/LRRTM3 |
| --- | --- | --- | --- | --- |

| 0.497710826 12233 |  | tags=41%,  list=22%,  signal=32% |  | MGLL/TNFRSF21/AGT/FABP5/F2  R/NCMAP/NTSR1/CCK/HGF/TY  MP/ADORA1/ZFHX2/SHANK1/HO  MER1/AVPR1A/FGF12/CCN3/TN  FRSF1B/GRIN2D/NPTX2/PTAFR/C  ACNG5/ITGAX/CCL3/NLGN4X/C  ELF4/ACPP/IL10/RELN/GRM1/ZN  F488/SHISA9/ITGA2/NMUR2/TE  NM4/CST7/SMR3B/CACNG4/SHI  SA6/NETO1/NRXN1/CUX2/OPRD  1/TAFA4/GRIN1/TNR/NPTX1/RI  MS2/OPRM1/RIMS1/CARTPT/GL  RA1/NPY2R/GHSR/HTR2C/SMR3  A |
| --- | --- | --- | --- | --- |

| 0.497710826 11549 |  | tags=49%,  list=20%,  signal=39% |  | CRHBP/DLG2/LRRC7/RGS9/SHAN  K1/HOMER1/LYPD6B/NPTX2/CA  CNG5/SLURP1/GSG1L/ARC/RELN  /SHISA9/GRIA1/SHISA8/EPHB2/  NEFL/CCR2/CACNG4/SHISA6/RA  SGRF1/NETO1/CACNG3/GRIA4/  CRH/CACNG7/GRIN1/GRIN2A/NP  TX1/GRIA3/OPRM1/GRIA2/CAC  NG2/IFNG |
| --- | --- | --- | --- | --- |

| 0.497710826 11630 |  | tags=28%,  list=21%,  signal=22% |  | MSX1/ADGRB1/MYOCD/EFNB2/F  ZD7/PI16/CD53/CCN3/DKK1/CC  L8/MYOD1/MYF6/LMOD3/TMEM  119/NRG1/CAV3/MIR199A1/NFA  TC2/IGF1/ANKRD2/CXCL10/SHH  /TNFSF14/BHLHA15/CXCL9/MYF  5/MYOG/MIR204/MIR199B/MIR2  00B/MIR208A |
| --- | --- | --- | --- | --- |

| 0.497710826 12096 |  | tags=45%,  list=21%,  signal=35% |  | MYRIP/CACNA1F/RCVRN/USH1G  /PRPH2/DHRS3/CDHR1/EYS/RHO  /RP1L1/GNAT1/RRH/GUCA1A/R  GR/PTGS1/MAK/KIF17/SPTBN5/  PDE6H/MAP1B/TULP1/RGS9BP/G  UCY2D/OPN1LW/USH1C/PROM1  /SAG/PDE6A/USH2A/ATP8A2/A  BCA4/PDC/GNGT1/OPN4/PCARE  /CNGB3/RD3/CNGA1/GRK1/CNG  B1/GUCA1C/CERKL/GRK7/RP1/  OPN5/PPEF2/GUCY2F/PCDH15/G  NAT2/OPN1MW/CETN1 |
| --- | --- | --- | --- | --- |

| 0.497710826 11149 |  | tags=34%,  list=20%,  signal=27% |  | P2RX2/GRIK5/NTSR1/P2RX6/GA  BRB3/ADORA1/SHANK1/DRD2/G  RIN2D/SLC17A7/CHRNA3/NLGN4  X/P2RX5/CELF4/GLRB/RELN/SEZ  6/INSYN2A/P2RX1/NETO1/CHRN  A4/NRXN1/CUX2/CHRNA9/CHRN  A2/GLRA2/GRIN1/GRID2/GRIN2A  /CHRNA6/RIMS2/OPRM1/GLRA3  /RIMS1/GLRA1/CHRNB3/NPY2R |
| --- | --- | --- | --- | --- |

| 0.497710826 14502 |  | tags=41%,  list=26%,  signal=31% |  | FZD5/HLA-  DPB1/CD9/HIP1/CD74/EGFR/CD4  /HLA-DRA/BTBD8/FZD2/HLA-  DRB1/NCALD/CEMIP/NRGN/HLA  -DPA1/HLA-  DRB5/SLC18A1/SH3GL2/TGFA/  WNT5A/HLA-  DQA2/SLC18A2/HLA-  DQB1/SLC17A7/HLA-  DQA1/HLA-  DQB2/RASSF9/LRP2/ROR2/IL7R/  AP1M2/KIAA0319/FCGR1A/ADC  Y8/SYT1/AREG/FCGR1B/EGF/S  GIP1/SYT9/SYT2/CD3D/GAD1/S  LC32A1/CD3G/CHRM2 |
| --- | --- | --- | --- | --- |

| 0.497710826 9749 |  | tags=50%,  list=17%,  signal=41% |  | CACNA1F/HPN/RCVRN/RPE65/A  NO1/EYS/NTSR1/RHO/ADORA1/  GNAT1/PKD1L3/RRH/GUCA1A/R  GR/PRDM12/PKD2L1/SEMA5B/PD  E6C/TULP1/RGS9BP/AIPL1/GUCY  2D/GPR88/OPN1LW/TRPM8/ELO  VL4/CAV3/CACNA2D4/TRPM3/  CALCA/GNGT2/ITGA2/SAG/PDE  6A/GRM6/NTRK1/ATP8A2/ABCA  4/HTR2A/TACR1/ADGRV1/PDC/  GNGT1/OPN4/CNGA1/GRK1/CNG  B1/CHRNA9/NPFFR2/GUCA1C/NR  2E3/TRPC3/TAC1/GRK7/CABP4/  DRGX/RP1/OPN5/ASIC2/TMC1/  SCN1A/GJA10/GUCY2F/GNAT2/  ANO3/KCNA1/LHFPL5/OPN1MW  /CSRP3 |
| --- | --- | --- | --- | --- |

| 0.497710826 14093 |  | tags=42%,  list=25%,  signal=32% |  | KITLG/CD274/CD74/BTK/BCL2L1  1/FCER1G/LGALS3/IRF7/LYN/C  XCL12/TSC22D3/JAK3/TNFRSF2  1/ARG2/ANXA1/LGALS9/GIMAP  8/ADAM8/IL6/HCLS1/WNT5A/H  CAR2/RIPK3/CCL19/CLC/CCL5/I  L7R/SLC7A11/IL10/IL2RA/NOD2  /CCL21/LILRB1/CCR7/CCR5/LG  ALS13/CD27/HSH2D/IDO1/CD3G  /FASLG/PDCD1/LGALS14/GHSR  /IL2 |
| --- | --- | --- | --- | --- |

| 0.497710826 10575 |  | tags=40%,  list=19%,  signal=33% |  | GCH1/SHANK1/CNTNAP1/UCHL1  /FGF12/DRD2/FOXS1/GBX1/PEN  K/GRIN2D/GRIN3A/STRA6/NPAS  1/SLC6A3/SLURP1/GPR88/CDH2  3/USH1C/GLRB/HOXA1/PRRT2/  NEFL/ATP8A2/POU4F1/NRXN1/C  LRN1/CSMD1/DRD3/GRIN1/TNR/  GRID2/GRIN2A/SLITRK6/DRD1/S  CN1A/TIFAB/PCDH15/NEUROG1/  GLRA1/KCNA1/POU4F2/DCANP1  /HMX3 |
| --- | --- | --- | --- | --- |

| 0.497710826 10645 |  | tags=50%,  list=19%,  signal=41% |  | NXPH4/TAC3/NPBWR1/SCG5/PE  NK/HCRTR1/NPY5R/KISS1R/SSTR  1/NPFFR1/NXPH3/MCHR2/SORCS  1/MCHR1/PDYN/GLRB/GALR1/C  ALCA/GRP/GPR84/SSTR2/NMUR  2/RXFP3/CYSLTR1/GPR83/ADCY  AP1/POMC/NPY/ECEL1/TENM1/  GPR149/OPRD1/NPFFR2/GLRA2/T  AC1/NXPH2/GALP/UTS2R/PPY/  HCRTR2/OPRM1/PRLHR/GLRA3/P  NOC/CYSLTR2/SSTR3/CARTPT/  GLRA1/PMCH/NPY2R/SSTR5/NM  S |
| --- | --- | --- | --- | --- |

| 0.497710826 13133 |  | tags=42%,  list=23%,  signal=32% |  | CTNND2/PAM/CACNA1C/RTN4RL  1/GJC2/NEURL1/OLFM1/CACNA  1F/RCVRN/AIF1/ELAVL4/PCSK2  /CRHBP/KCNAB1/ASTN2/ITGA1  /GRIK5/NTSR1/CCK/DRD2/NGFR  /PENK/SCN1B/MAPK10/EPHA4/K  CNA2/MAP1B/TRPM2/KCNK1/SL  C2A3/LRIT3/ASTN1/RBFOX3/HP  CA/PTPRN/ADCYAP1/CCR2/CPN  E5/SLC5A7/CPNE6/RGS8/KCNB2  /CRH/NGB/CNR2/CNTNAP2/OPR  M1/CNGA3/HTR5A/SLC8A3/GLR  A3/SLC4A10/KCND2/KCNC2/SL  C17A8/NEUROG1/GLRA1/KCNA1 |
| --- | --- | --- | --- | --- |

| 0.497710826 11187 |  | tags=42%,  list=20%,  signal=34% |  | CD1D/NLRP3/HLA-  F/RSAD2/C3/IL6/FCER2/CD1C/  TNFSF4/CD1E/CD1A/SH2D1B/IL1  8RAP/FOXP3/VAV1/SASH3/IL18  /CD28/CD160/IL1B/CADM1/CD1  B/PTPRC/GATA3/IL23R/IL12RB1  /TBX21/NCR3/KIR2DL4/TNF/XC  L1/SLAMF6/RASGRP1/LAG3/LT  A/IL12B/KLRC4-  KLRK1/CRTAM/CLNK/SH2D1A/K  LRK1/IL21/IL2 |
| --- | --- | --- | --- | --- |

| 0.497710826 12660 |  | tags=46%,  list=22%,  signal=36% |  | LGALS9B/JAK3/SYK/ARG2/LOX  L3/ANXA1/LGALS9/NLRP3/CD80  /VSIR/TNFSF4/RUNX1/CD86/CC  L19/FOXP3/NCKAP1L/SASH3/CD  300A/IL18/EBI3/CD28/PRDM1/C  D160/LILRB1/IRF1/ZNF683/PTPR  C/IL27/RASAL3/CCR2/MYB/GA  TA3/IL23R/CD3E/SHH/IL12RB1/  TBX21/ZAP70/XCL1/IL12B/IFNG  /IL2 |
| --- | --- | --- | --- | --- |

| 0.497710826 12143 |  | tags=45%,  list=21%,  signal=35% |  | AGT/PTGER3/SULF1/MYOCD/F2  R/HTR7/P2RX2/KCNMA1/ADORA  1/GUCY1A1/DRD2/SULF2/EDN2/  ADRA2B/ADRA2A/PTAFR/OXTR/  SPHK1/MYH11/CHRNA3/BDKRB2/  ATP1A2/ADRA1B/PTGS2/SLC8A  1/DOCK4/CHRM3/NPNT/CALCA  /HTR1D/ITGA2/SSTR2/NMUR2/H  TR2A/TACR1/ADRA1A/P2RX1/C  D38/HTR2B/KCNB2/DRD1/TACR3  /TIFAB/NEUROG1/CHRM2/NPY2R  /GHSR/DCANP1/MIR153-1 |
| --- | --- | --- | --- | --- |

| 0.497710826 11148 |  | tags=50%,  list=20%,  signal=41% |  | GRIK5/UNC13A/ADORA1/SHANK  1/HOMER1/DRD2/GRM2/DKK1/S  HC3/KMO/GRIN2D/GRIN3A/OXTR  /SLC17A7/ATP1A2/GRID1/CDH2  /PTGS2/ROR2/GRM3/RELN/GRM  1/GRIA1/GRM6/NTRK1/HTR2A/  ADCYAP1/SYT1/P2RX1/HTR1B/  CCR2/GRM4/NRXN1/GRM8/GRIK  1/GRIA4/TNF/DRD3/GRIN1/TNR  /GRID2/GRIN2A/GRIA3/DRD1/GR  M7/GRM5/GRIA2/UNC13C/SLC1  7A8/SLC17A6/NPY2R/OR56A5 |
| --- | --- | --- | --- | --- |

| 0.497710826 6238 |  | tags=82%,  list=11%,  signal=73% |  | 2/TRDC/TRAC/TRAV17/TRAV25  /TRBJ2-2/TRAV9-  2/TRAV27/TRBC2/CD3E/TRAV41  /CD247/TRBV28/TRAV13-  2/TRAV6/SKAP1/TRAV40/TRBV2  9-  1/CD3D/TRAV16/TRAV5/TRBV1  0-3/TRAV8-  4/TRBV30/ZAP70/TRBJ2-  3/TRAV1-2/TRBV5-4/TRBV24-  1/TRBV5-6/TRBJ2-  7/CD8A/TRBV5-1/TRBV20-  1/TRBV2/TRAV20/TRAV18/TRA  V35/TRBV7-9/TRBV4-  1/TRBV3-  1/TRAV39/TRGV9/TRAV29DV5/  TRGV3/TRBV7-  7/TRBV19/TRAV12-  3/TRAV2/TRAV36DV7/TRBV7-  4/TRAV13-1/TRAV12-  1/TRAV8-  6/TRAV14DV4/TRBV5-  5/TRAV21/CD8B/TRBV18/TRAV1  0/TRAV34/TRBV6-  5/TRAV22/TRAV8-1/TRBV10-  1/TRGC2/CD3G/TRDV1/TRAV8-  3/TRAV26-  1/TRBV9/TRBC1/TRBV12-  4/TRAV19/TRAV3/TRBV6-  1/TRBV7-6/TRBV4-  2/TRAV23DV6/TRGV4/TRAV24/  TRBV13/TRBV6-  6/TRAJ3/TRBV12- |
| --- | --- | --- | --- | --- |

| 0.497710826 12415 |  | tags=47%,  list=22%,  signal=37% |  | JAK3/SYK/LOXL3/ANXA1/LGAL  S9/GPR183/NLRP3/LEF1/CD80/P  LA2G2D/RSAD2/IL6/BCL3/PAX1  /TNFSF4/TNFSF8/RUNX1/CD86/  CCL19/FOXP3/NCKAP1L/SASH3/  IL18/PRDM1/PTGER4/EOMES/IRF  1/BATF/ZNF683/IL27/LY9/MYB  /GATA3/IL23R/SHH/IL12RB1/SP  N/TBX21/ZAP70/SLAMF6/FUT7  /GPR18/ITK/BCL11B/IL12B/IFNG  /IL2 |
| --- | --- | --- | --- | --- |

| 0.497710826 13310 |  | tags=53%,  list=24%,  signal=41% |  | RELB/NKX2-  3/RUNX3/JAK3/LOXL3/ANXA1/  LGALS9/GPR183/NLRP3/LEF1/CD  80/PLA2G2D/RSAD2/IL6/BCL3/P  AX1/TNFSF4/RUNX1/CD86/CCL  19/FOXP3/NCKAP1L/SASH3/IL1  8/PTGER4/BATF/IL27/LY9/MYB  /GATA3/IL23R/IL12RB1/SPN/TB  X21/SLAMF6/FUT7/IL12B/IFNG/  IL2 |
| --- | --- | --- | --- | --- |

| 0.497710826 11549 |  | tags=49%,  list=20%,  signal=39% |  | CRHBP/DLG2/GRIK5/LRRC7/UNC1  3A/RGS9/SHANK1/HOMER1/GRM  2/GRIN2D/GRIN3A/CACNG5/GRI  D1/SSTR1/GSG1L/ARC/GRM3/R  ELN/GRM1/SHISA9/GRIA1/SHISA  8/EPHB2/GRM6/NEFL/CCR2/CA  CNG4/SHISA6/GRM4/FRRS1L/RA  SGRF1/NETO1/GRM8/GRIK1/CAC  NG3/GRIA4/CRH/CACNG7/GRIN1  /SULT1A4/GRID2/GRIN2A/GRIA3  /GRM7/OPRM1/GRM5/GRIA2/C  ACNG2/IFNG |
| --- | --- | --- | --- | --- |

| 0.497710826 11867 |  | tags=67%,  list=21%,  signal=53% |  | C2/CFI/C3AR1/IGLV1-  47/VSIG4/IGKV4-1/IGLV6-  57/KLK5/C5AR2/C3/C8G/FCER2  /IGLV3-21/C1R/CD19/IGKV3-  20/IGLC2/IGLV2-  8/SPNS2/C1S/CFB/IGHV4-  59/IGLV2-  14/IGHG2/IGHG1/IGHV4-  39/PTPN6/PGC/IGHV3-  23/IGKC/VTN/IGLV2-23/IGLV2-  11/IGHV4-34/IGKV3-  15/IGLC7/IGHV3-48/IGLV3-  19/IGKV1-5/IGHV3-11/IGLV1-  44/IGHG3/IGLC6/C7/IGHV3-  30/IGHV2-  5/IL1B/IGLC3/C9/IGHG4/CR1L/I  GLV1-40/IGKV1-17/IGHV3-  33/PTPRC/CCR7/IGKV3D-  11/IGHV2-70/IGHV3-7/IGLV3-  25/IGLV7-43/ACOD1/IGLV3-  1/IGKV1-16/C4B/IGHV3-  13/C4A/TNF/IGHV1-  69/SUSD4/IGKV5-  2/SPINK5/IGKV2D-28/IGKV2D-  30/IGKV1-12/IGHV3-  53/IGKV3D-20/LTA/IGKV2-  29/IGKV1D-12/IGKV2-  30/IGKV1D-39/IGKV1-  39/CXCL13/IGLV3-  27/CD5L/IGKV1D-  33/CR2/IGKV2-28 |
| --- | --- | --- | --- | --- |

| 0.497710826 11624 |  | tags=45%,  list=21%,  signal=36% |  | LGALS9/CD1D/PIK3R6/NCR1/HL  A-  F/CD1C/RIPK3/HAVCR2/CD1E/C  D1A/IL18RAP/VAV1/IL7R/CD160  /CADM1/CD1B/LILRB1/PTPRC/IL  23R/IL12RB1/NCR3/KIR2DL4/STA  P1/XCL1/SLAMF6/RASGRP1/LA  G3/IL12B/KLRC4-  KLRK1/CRTAM/SH2D1A/KLRK1/I  L21 |
| --- | --- | --- | --- | --- |

| 0.499259259 10152 |  | tags=44%,  list=18%,  signal=36% |  | BCL3/VSIR/WNT5A/IL27RA/CD2  44/RIPK3/TNFSF4/HAVCR2/TLR7  /PGLYRP3/IL18RAP/FOXP3/CLEC  7A/SASH3/IL18/EBI3/IL10/CD16  0/IRF8/IFNL1/IL1B/EOMES/LILRB  1/TLR8/CCR7/IL27/CCR2/GATA  3/IL23R/CD3E/CD2/IL12RB1/SPN  /TNF/XCL1/SLAMF6/ISL1/RASG  RP1/PTPN22/CD96/ITK/LTA/IL1  2B/PGLYRP2/KLRC4-  KLRK1/KLRK1/IL21/IL2 |
| --- | --- | --- | --- | --- |

| 0.499259259 14413 | |  | | tags=43%,  list=26%,  signal=32% | |  | | GDF15/CD9/EHD2/TBX1/XK/MY  OF/ADAM12/SIX1/PLEKHO1/NK  X2-  5/KLHL41/KLHL40/CACNA1H/AD  GRB1/MYOCD/P2RX2/CD53/HOM  ER1/AVPR1A/ADGRB3/CCN3/CC  L8/MYOD1/MYF6/WNT10B/LMO  D3/ACTA1/TMEM119/CAV3/NF  ATC2/IGF1/ANKRD2/SIX4/CXCL  10/SHH/TNFSF14/MYMX/NOS1/  NPHS1/BHLHA15/CXCL9/WNT1/  MYMK/BARX2/MYF5/MYOG/MIR  200B | | |
| --- | --- | --- | --- | --- | --- | --- | --- | --- | --- | --- |
| 0.499259259 7199 |  | | tags=23%,  list=13%,  signal=20% | |  | | CAV3/MIR199A1/IGF1/MIR199B/  MIR208A | | |  |
| 0.554321901 8892 |  | | tags=15%,  list=16%,  signal=13% | |  | | MIR107/MIR103A1/GCG/MAS1/I  NS/PPARGC1A/HAS2/POU1F1/C  1QTNF12/ENPP1/PTH1R/SIK1/MS  T1/LHCGR/PPP1R3B | |  | |

| 0.554321901 2577 |  | | tags=18%,  list=5%,  signal=17% CTAG1A/CTAG1B/CTAG2 | | | | | |  | |
| --- | --- | --- | --- | --- | --- | --- | --- | --- | --- | --- |
| 0.554321901 21775 | |  | | tags=84%,  list=39%,  signal=52% |  | PPARGC1A/RPL3L/RPL39P5/HBA1  /RPS10P5/RPL10L/EIF2A/EIF2D/  RPLP0P6/RPL35A/RPL14/RPL15/R  PL24/RPSA/RPL32/RSL24D1/RPL  39L/RPS7/RPS3A/RPS10/RPL29/  RPL36A/RPL9/RPS18/RPS15A/RP  L17/NUFIP1/RPL39/RPL5/RPL10A  /RPL31/RPS17/RPL38/RPS6/RPS2  9/RPL34/RPS24/RPL26/RPS12/RP  S3/RPL7L1/RPL22/RPS27A/RPL12  /RPL21/EIF2AK4/RPS23/RPS4X/  RPLP1/RPS8/RPS27/RPL3/RPS19/  RPL23/RPL37/RPL4/RPS25/RPL6/  RPL18/RPL11/RPL18A/RPS9/RPL3  7A/DDX3X/RPS5/RPL27A/RPL10  /RPS21/RPS13/RPL7A/RPL35/RP  S2/RPL13A/RPS28/RPL13/RPS14  /RPS15/RPLP0/RPS4Y2/RPLP2/RP  L36AL/RPS16/RPS11/RPL41/RAC  K1/RPL36/RPL27 | | | | |
| 0.554321901 3597 |  | | tags=6%,  list=6%,  signal=6% | |  | MIR107/MIR103A1/GCG/INS/FBP  2/PPARGC1A | | | |  |
| 0.554321901 513 |  | | tags=8%,  list=1%,  signal=8% MIR107/MIR103A1 | | | | |  | | |
| 0.554321901 4120 |  | | tags=18%,  list=7%,  signal=17% CCT8L2/CCT8L1P | | | |  | | | |

| 0.554321901 19134 | |  | | tags=42%,  list=34%,  signal=28% | |  | | 5F/BACE1/AP2S1/CLCN3/VPS33  A/AP1G2/TM9SF2/SNX21/PLIN3  /NPC1/PLEKHB2/CHMP2A/SORT1  /STAM/ANTXR1/RAP2A/PSENEN  /AP5S1/GPR135/ARHGAP1/SNX8  /SNF8/ANTXR2/TFRC/GRB14/LA  MTOR2/RAB11FIP3/RAB8A/SCA  MP4/ANKFY1/INPP4A/VPS28/AP  2A1/RAB35/ABCA2/GOSR2/LAP  TM4B/EHD1/GRIPAP1/BAIAP3/SP  PL2A/ATP6V0D1/IRAK1/PSEN1/T  PCN1/YIPF2/CLCN4/PLEKHM2/C  ORO1C/STEAP4/PLEKHM1/IKBKE  /TBK1/PCSK9/AP2A2/STARD3/  ZNRF2/RNF144A/SLC29A3/TMEM  175/SLC30A3/ATP9A/SLC39A4/  CHMP4A/TICAM1/RILP/IFITM3/I  RAK2/HLA-  E/SLC9B2/ATP6V0B/FIG4/MCOL  N1/PLEKHF2/SNX25/SLC11A2/C  D68/DTX3L/ABCB6/WLS/PRAF2  /SNX16/MARCHF3/TMEM165/TL  R3/KCNH1/B2M/DNAJC13/CD14  /SLC9A9/FZD5/PLEKHF1/SNX10  /PARM1/HLA-  DPB1/LDLRAD4/EHD3/EHD2/MCO  LN2/VPS53/ZFYVE28/RAB23/CD  274/PMEPA1/DIO3/EGFR/SLC38  A9/HLA-  DRA/WIPI1/ATP6V0E2/TCIRG1/  MARCHF1/HLA-  G/MRC1/IRF7/RET/HLA-  H/RAB15/HLA-DRB1/HLA- | | |
| --- | --- | --- | --- | --- | --- | --- | --- | --- | --- | --- |
| 0.554321901 3247 |  | | tags=19%,  list=6%,  signal=18% MIR132/INS/NR1H4 | | | | | |  | |
| 0.554321901 3597 |  | | tags=25%,  list=6%,  signal=23% | |  | | MIR107/MIR103A1/GCG/PPARGC  1A | | |  |

| 0.554321901 14185 | |  | | tags=31%,  list=25%,  signal=24% |  | AKAP6/ZP3/TFCP2L1/CHD7/CXC  R4/DIO3/EGFR/SH3PXD2B/FGFR1  /SEMA7A/CSF1/SEMA5A/ACAC  B/RND2/FGF8/SLC44A4/AGR2/S  FRP2/CXCL12/HAMP/BASP1/WT  1/NRP1/HPN/NGF/ISLR2/TNFRSF  12A/TRPV2/TBX5/MAPT/LEF1/K  RT17/UNC13A/TBX20/FN1/IGF2  /GLI1/RFTN1/CRABP2/AVPR1A/  H1-  5/HCLS1/DRD2/FOXS1/WNT2/T  GFB2/IL7/GH2/MYOD1/FGFR2/S  YT14P1/SPHK1/IGFBP1/POU3F2/  ARX/MAP1B/SLC6A3/CSHL1/SF  N/SASH3/MIR199A1/IGF1/NTN1  /ZFPM2/ATP8A2/NTRK3/SYT1/  GATA6/CPNE5/CD38/SYT4/DSC  AM/SYT2/CPNE6/CACNG7/PRSS  2/REG1A/RIMS2/RIMS1/POU4F2  /GHSR/MIR204/MIR199B/IL2/MI  R208A | | |
| --- | --- | --- | --- | --- | --- | --- | --- | --- |
| 0.554321901 513 |  | | tags=18%,  list=1%,  signal=18% MIR107/MIR103A1 | | | |  | |
| 0.554321901 8066 |  | | tags=55%,  list=14%,  signal=47% | |  | MIR33A/NR1H4/MALRD1/FGF19/  STAR/CYP7A1 | |  |

| 0.554321901 14198 | |  | | tags=45%,  list=25%,  signal=34% | |  | | SLC2A5/ZP3/CLEC2B/GALNT5/C  LEC14A/CD34/PPP1R3F/COLEC12  /NECTIN1/ASGR1/GALNT14/GAL  NT7/HLA-  DRA/GALNT10/P4HTM/LGALS3/  MRC1/PAM/HLA-  DRB1/MAN2A1/LGALS9B/ASGR2  /CLEC18B/CALR3/STBD1/TINAG  L1/PFKP/CHI3L1/GALNT15/LOXL  2/SBSPON/CD72/MANBA/LGALS  9/PLA2R1/SIGLEC1/P4HA2/ADG  RL2/CD93/PRG4/GALNT16/PPP1R  3G/FCN3/CLEC4A/FCER2/CLEC4  C/CD33/SIGLEC16/SIGLEC7/SUS  D2/LGALS2/LAYN/CRYBG1/PKD  1L3/NPTX2/LMAN1L/P3H2/CLEC  4E/VCAN/PRG2/HKDC1/CLEC19  A/SIGLEC9/GALNT18/SELE/ITLN  1/SIGLEC8/ZG16B/VTN/SLC2A3  /MRC2/CLEC7A/CLEC18C/SIGLE  C5/SELL/GALNT9/CLC/NCAN/R  EG3G/CRYBG2/CD209/SIGLEC14  /HK3/APCS/CLEC17A/SIGLEC11  /CLEC4G/SIGLEC12/CLEC1B/AC  AN/GAL3ST3/CLECL1/KLRC2/CL  EC3A/MGAM/CHI3L2/LGALS13/  CD69/KLRD1/CNTN2/GALNT13/  ADGRL3/LGALS7B/SIGLEC10/C4B  /FCN2/CLEC12A/GALNT17/CLEC  9A/CLEC2L/REG1A/SIGLEC6/KLR  C4-  KLRK1/KLRC3/LGALS14/KLRC1/  KLRK1/REG1B/MBL2/REG3A/IL2 |
| --- | --- | --- | --- | --- | --- | --- | --- | --- |
| 0.554321901 3597 |  | | tags=12%,  list=6%,  signal=11% | |  | | MIR107/MIR103A1/GCG/INS/PPA  RGC1A | |
| 0.554321901 2249 |  | | tags=9%,  list=4%,  signal=9% | |  | | CT45A10/CT45A1/CT45A3/CT45  A5 | |
| 0.554321901 2249 |  | | tags=11%,  list=4%,  signal=11% | |  | | CT45A10/CT45A1/CT45A3/CT45  A5 | |

| 0.554321901 12718 |  | tags=27%,  list=23%,  signal=21% |  | NKX2-  5/GJA1/KLHL41/HEYL/NRAP/HA  MP/KLHL40/PDGFRB/NEURL1/GRE  M1/AGT/WT1/FOXC1/MYOCD/  TBX5/HEG1/KCNAB1/EFNB2/P2R  X2/GATA5/LEF1/MYBPC3/FZD7  /SAP30/TBX20/GJC1/ELN/PI16  /SCX/TNNC1/GLI1/HOMER1/TP7  3/MYLK2/WNT5A/TLL2/NOG/P  AX7/DKK1/WNT2/TGFB2/ASB2/  VGLL2/MYOD1/ALPK3/FGFR2/FO  XC2/STRA6/MYF6/MYH11/CAS  Q1/BMP7/WNT10B/LMOD3/ACT  A1/FGF3/SOX11/PGM5/ADAMT  S9/NRG1/MYOZ2/SLC8A1/LRP2  /NDRG4/TWIST1/MSTN/FOXL2/  DLL4/CAV3/MYH15/MIR199A1/I  GF1/MIR23A/TNNT2/ZFPM2/PHO  X2B/EOMES/TENM4/GJA5/NEBL  /ANKRD2/SIX4/POU4F1/ADRA1  A/GATA6/ALPK2/PAX5/PLN/SH  H/NPHS1/SGCG/RYR2/VAX1/ISL  1/ACTC1/MYOM3/AGTR2/ANKR  D33/CSRP3/MYF5/NKX2-  6/MYOG/MIR204/MIR199B/MIR2  00B/MIR208A |
| --- | --- | --- | --- | --- |

| 0.554321901 15644 |  | tags=45%,  list=28%,  signal=33% |  | RND1/LGALS1/JAM2/GLI3/SEM  A4D/BMP2/ZC3H12A/ACVRL1/C  X3CL1/ADAM15/LPXN/LRRC32/  PDE5A/IL20RB/SPINT2/NEXMIF/G  LMN/LRP1/PAWR/CD9/PLXNA3/  MAD1L1/PLXNA2/FGG/CD274/C  DH13/PLET1/CD74/C1QTNF1/AS  S1/TGFB1/SEMA5A/CCM2L/DTX  1/ANGPT1/SH2B3/HLA-  G/LGALS3/RUNX3/CDH1/TRPV4  /NRARP/LGALS9B/COL1A1/HRG  /FOXJ1/CXCL12/PELI1/JAK3/T  NFRSF21/ARG2/LOXL3/JAG1/A  NXA1/ANGPT2/CORO2B/LGALS9  /B4GALNT2/VSIG4/SPOCK1/PDC  D1LG2/FZD7/FAM107A/CD80/T  GFBI/ARHGDIG/PLA2G2D/THBS1/  MUC1/IL1RN/VSIR/TNC/KLF4/IF  NB1/SERPINE1/ADAM22/TNFSF4  /PLA2G2E/DAB1/HAVCR2/RUNX  1/TACSTD2/DACT2/TNFAIP8L2/  CD86/ADTRP/ARHGAP6/PTPN6/P  LA2G2A/FOXP3/LILRB2/CD300A  /PLXNA4/IL10/SEMA3E/PLG/FG  L2/IFNL1/CLEC4G/IL2RA/PTPRO  /CCL21/CCL25/LILRB1/IRF1/PTP  RC/MYOC/MIR503/ADAMDEC1/  DSCAM/SHH/SPN/TBX21/GBP1/  ADAMTS18/XCL1/PTPN22/WNT1  /TNR/LAG3/MMP12/IDO1/LAX1  /TIGIT/CTLA4/IL2/MIR138-2 |
| --- | --- | --- | --- | --- |

| 0.554321901 13685 |  | tags=33%,  list=24%,  signal=26% |  | TRIB1/SEMA7A/PLAUR/SEMA5A  /NBL1/SEMA3A/MIR26B/SH2B3/  IFI16/KLKB1/HLA-  G/CDH5/CALCRL/RTN4RL1/GJA1  /HRG/PROS1/THBD/SEMA3G/CD  KN1A/PLAU/ELANE/GREM1/ARG  2/CR1/IRAK3/NRP1/ANGPT2/HT  RA1/AIF1/LGALS9/VSIG4/SEMA  3F/MIR26A2/NLRP3/SOCS3/CCK  /CYP19A1/HGF/HLA-  F/ADORA1/C5AR2/NLRC3/SLIT2  /MMP28/THBS1/FFAR4/WNT5A  /KLF4/DRD2/CCN3/SEMA6B/NP  Y5R/TNFRSF1B/GRIN3A/SERPINE1  /CARD16/FGB/CD109/EPHA4/H  AVCR2/SYT11/TNFAIP8L2/ADTR  P/SLC6A3/NT5E/TNFAIP6/VTN/  RNF125/FOXP3/MIR766/DUSP10/  SLAMF8/APCS/F12/MEFV/IL10/  SIGLEC11/CXCL17/SEMA3E/PLG  /FOXF1/NLRC5/FGL2/LRFN5/IGF  1/PTGER4/IL2RA/PTPRO/LILRB1/  SMPDL3B/CST7/ADCYAP1/PTPRC  /FPR2/IL1R2/GATA3/SERPINB2/  CD200R1/ABCD2/ACOD1/NRXN1  /SIGLEC10/NLRP6/KIR2DL4/STAP  1/ADAMTS18/NLRP7/ISL1/MIR39  09/CD96/DRD3/SPINK5/GRIN1/C  NR2/GPR18/TNR/GRID2/IL12B/M  MP12/DRD1/KLRC4-  KLRK1/CARTPT/CXCL13/KLRK1/I  L22RA2/FGA/GHSR/MIR204/IL2/  MIR181A1/MIR138-2 |
| --- | --- | --- | --- | --- |

| 0.554321901 14937 |  | tags=51%,  list=26%,  signal=38% |  | LPAR1/CXCL14/ANO6/S100A14  /GAS6/TREM2/RARRES2/PLXNA  3/F7/CXCR4/THBS4/DAPK2/CD  H13/CD74/TGFB1/FGFR1/SEMA7  A/CSF1/SEMA5A/P2RX4/PGF/N  BL1/SEMA3A/F3/LYN/BST1/TRP  V4/S1PR1/IL16/ARTN/HRG/CXC  L12/SEMA3G/PLA2G7/PDGFRB/E  LANE/GREM1/NRP1/KDR/ANGPT2  /C3AR1/AIF1/LGALS9/TUBB2B/  GPR183/SEMA3F/EFNB2/CYP19A  1/RAC2/CCR1/CMKLR1/C5AR2/  ZSWIM5/SLIT2/MMP28/IL6/THB  S1/DYSF/SCG2/WNT5A/CCN3/  SEMA6B/EDN2/CCL7/SMOC2/SE  RPINE1/FGF18/CCL3/CCL19/SUC  NR1/CASR/NCKAP1L/CCL5/CAM  K1D/MSTN/SLAMF8/PLXNA4/CX  CL17/SEMA3E/ITGA2/PTPRO/CC  L21/NTRK3/CCR7/FPR2/NTF3/C  CL26/CCR2/CCL4/CXCL10/DSC  AM/LBP/TNFSF14/STAP1/FGF10  /XCL1/CCR4/XCL2/FEZF2/GPR1  8/S100A7/KLRC4-  KLRK1/CCR6/TBR1/CXCL13/CCL  1/KLRK1/POU4F2 |
| --- | --- | --- | --- | --- |

| 0.554321901 13759 |  | tags=37%,  list=24%,  signal=28% |  | COL8A1/NECTIN1/NECTIN3/FGFR  1/SIX1/FGF8/POU4F3/ROM1/SL  C44A4/MYO7A/CRYGB/FZD2/M  AN2A1/GRHL3/MAFB/JAG1/HPN  /USH1G/FRZB/RPE65/THY1/MSX  1/DLX5/FJX1/GDF11/PAX6/CO  L5A1/GNAT1/FOXN4/WNT5A/N  OG/FSCN2/ZIC1/WNT2/FGFR2/  STRA6/RORB/WNT9A/PDE6C/TH  RB/HOXC13/BMP7/PRRX1/TULP1  /SOX11/NKX3-  2/ZEB1/LCTL/ROR2/TWIST1/FO  XL2/PRDM1/USH1C/GLI2/PROM1  /MEGF11/HOXA1/ATOH1/VSX1  /PITX3/NTN1/CNTF/EPHB2/SDK2  /GBX2/PAX2/SIX4/ATP8A2/CA  LB1/FOXG1/GNGT1/GATA3/CRB  1/DSCAM/CLRN1/CHRNA9/MFRP  /NR2E3/FGF10/OSR2/WNT1/CA  BP4/RP1/TFAP2B/SLITRK6/OLFM  3/LHX1/TIFAB/FASLG/GNAT2/  MYO3B/NEUROG1/LHFPL5/DCANP  1/HMX3 |
| --- | --- | --- | --- | --- |

| 0.554321901 13510 |  | tags=27%,  list=24%,  signal=21% |  | SYP/HLA-  DRA/BTBD8/DLG4/CRISPLD2/HL  A-G/PAM/HLA-  H/RAB15/SYTL1/SYTL4/HLA-  DRB1/RAB26/SYNGR3/CPE/SLC1  7A5/TMEM184A/MYRIP/GALNT1  5/UNC13D/DPYSL3/NCALD/SV2  A/SYTL3/SCG3/NGF/CTTNBP2/  PCSK2/SYT12/MT3/CHGA/NRGN  /HLA-DPA1/HLA-DRB5/HLA-  F/UNC13A/RAB40AL/SLC18A1/R  PH3AL/SLC6A9/SH3GL2/TGFA/S  YTL5/SEC24D/SLC30A8/HLA-  DQA2/DRD2/BRSK1/RAB27B/PEN  K/SLC18A2/HCK/PRG2/HLA-  DQB1/OTOF/SLC17A7/SYT11/S  NCAIP/SYT10/SORL1/HLA-  DQA1/HLA-  DQB2/RASSF9/STON2/TRIM9/RA  B3B/TMEM163/AMPH/CPA3/KCN  K9/FOLR1/IGF1/GRIA1/PCSK1/P  RRT2/LAMP5/VGF/PTPRN/SYT1/  HAP1/NTF3/SYT5/AREG/SYT4/  GABRA2/SYT6/CLRN1/SNAP91/  OPRD1/SYT9/SYT2/RAB3C/SCG  N/DDC/DRD3/GRIN1/GRIN2A/NP  TX1/NRSN1/SLC32A1/SVOP/SV  2C/UNC13C/SLC17A8/SLC17A6  /OR56A5/CPLX3 |
| --- | --- | --- | --- | --- |

| 0.554321901 12718 | |  | | tags=38%,  list=23%,  signal=29% | |  | | NKX2-  5/SFRP2/PRDM14/JAG2/TBX19/  NOTCH3/TBXT/LOXL3/TBX15/J  AG1/WT1/NRP1/DLX2/NKX2-  1/TBX5/GCM1/TBX10/FZD7/PA  X6/WNT10A/TBX20/IL6/DMRT3  /FOXN4/WNT5A/KLF4/PAX7/G  BX1/DKK1/WNT2/WNT9B/IL7/E  VX1/GSX2/MYOD1/FGFR2/FOXC  2/OLIG1/MYF6/WNT9A/SOX17/  POU3F2/GDF3/ARX/PRRX1/RUNX  2/WNT10B/SOX2/FOXP3/EBF2/  ONECUT1/NRG1/CHD5/NKX2-  2/ROR2/SLAMF8/DLL4/PRDM1/  GLI2/HES5/POU6F2/OLIG2/ATOH  1/MNX1/BARHL2/EOMES/TENM4  /BATF/DSCAML1/PAX2/NTRK3/  POU4F1/GATA6/FOXG1/LY9/GA  TA3/IL23R/NEUROD1/SHH/IL12R  B1/SPN/TBX21/NEUROD4/FGF10  /TLX3/SLAMF6/ISL1/ISL2/WNT  1/FEZF2/LHX3/BCL11B/IL12B/T  BR1/NEUROG1/MYT1L/OLIG3/M  YF5/MYOG/MIR208A |
| --- | --- | --- | --- | --- | --- | --- | --- | --- |
| 0.554321901 2249 |  | | tags=8%,  list=4%,  signal=8% | |  | | CT45A10/CT45A1/CT45A3/CT45  A5 | |

| 0.554321901 12430 |  | tags=33%,  list=22%,  signal=26% |  | LGALS9B/FOXJ1/ISG15/JAK3/EL  ANE/IL1RL1/INHBA/TNFRSF21/A  RG2/IRAK3/ANXA1/CX3CR1/MC  1R/LGALS9/BST2/VSIG4/MIR26A  2/NLRP3/PDCD1LG2/LEF1/HGF/  CMKLR1/HLA-  F/ORM1/C5AR2/NLRC3/FN1/IL6  /THBS1/CLEC4A/FFAR4/LILRA4  /CD33/BCL3/VSIR/KLF4/IL27RA  /LILRA5/INHBB/TGFB2/IFNB1/TN  FSF4/CARD16/INPP5D/HAVCR2/  SYT11/CD84/PTPN6/PGLYRP3/R  NF125/FOXP3/MIR766/NCKAP1L  /TWIST1/TNFRSF4/IL13/MEFV/I  L10/NLRC5/IFNL1/IGF1/PTGER4/  LILRB1/TLR8/POMC/PTPRC/IL1R2  /GATA6/GATA3/IL23R/CD200R1  /ABCD2/ACOD1/LBP/TBX21/BA  NK1/GBP1/TNF/XCL1/NLRP7/PT  PN22/APOA2/CD96/GPR18/LAG3  /SLAMF1/IL12B/PGLYRP2/IDO1/  TIGIT/MMP8/IFNG/CARD17/GHS  R/CARD18/MIR204/MIR181A1 |
| --- | --- | --- | --- | --- |

| 0.554321901 12365 | |  | | tags=28%,  list=22%,  signal=22% | |  | | NKX2-  5/HEYL/CXCL12/TCF4/NEURL1/  AGT/DPYSL3/FOXO6/NRP1/ITGA  6/DLX2/NGF/ISLR2/VWC2/TNFR  SF12A/TRPV2/ALK/TUBB2B/MAP  T/GDF6/HGF/LRRC7/UNC13A/SL  IT2/SHANK1/FN1/CAMK1/LRP8/  CRABP2/COBL/HCLS1/EPO/WNT  5A/EPHA3/NGFR/FEZ1/DKK1/PC  P4/ADRA2B/SCN1B/SYT14P1/EP  HA4/DAB1/BMP7/MAP1B/PACSI  N1/GDF5/SOX11/ZEB1/NKX2-  2/ROR2/CAMK1D/NDRG4/TRPC6  /PLXNA4/ITPKA/GLI2/RELN/AT  OH1/SOCS2/TRIM67/NTN1/CNTF  /LIN28A/PHOX2B/NTRK1/NEFL/R  OR1/ATP8A2/NTRK3/ADCYAP1/  SYT1/BRINP3/NEUROG3/FOXG1/  CPNE5/BRINP1/STMN2/NEUROD1  /FEZF1/SH3GL3/SYT4/DSCAM/P  TPRD/CUX2/BRINP2/SYT2/PLPPR  5/CPNE6/FEZF2/SLITRK1/CACNG  7/ZNF804A/NEGR1/REG1A/NEUR  OD2/RIMS2/RIT2/VWC2L/RIMS1  /MMD2/NEUROG1/POU4F2/IL2 | |
| --- | --- | --- | --- | --- | --- | --- | --- | --- | --- |
| 0.554321901 8066 |  | | tags=46%,  list=14%,  signal=40% | |  | | MIR33A/NR1H4/MALRD1/FGF19/  STAR/CYP7A1 | |  |
| 0.554321901 3597 |  | | tags=10%,  list=6%,  signal=10% | |  | | MIR107/MIR103A1/GCG/INS/PPA  RGC1A | | |

| 0.554321901 10685 |  | tags=26%,  list=19%,  signal=21% |  | CYBB/SLIT2/LRP8/NQO1/AVPR1  A/TNC/KLF4/FOSL1/DRD2/PENK  /MAP4K1/GRIN3A/PTGFR/MAOB  /FGFR2/TNFSF4/KCNMB1/HSD3B  2/TRH/SLIT3/SLC6A3/CCL3/CC  L19/CLDN1/NPPC/VCAM1/MSTN  /IL13/ITPR2/PTGER4/PTGDR/CC  L21/P2RY6/NEFL/ADCY8/CA3/N  TRK3/CLDN3/MLC1/ADCYAP1/A  DH7/CCR7/MIR185/HTR1B/ADCY  AP1R1/CCR5/GATA3/ADCY1/CD  27/ADCY2/GLRA2/CRH/DRD3/G  RIN1/GRIN2A/ACTC1/OPRM1/HT  R3A/KCNC2/GLRA1/OR56A5/IL2  /MIR182 |
| --- | --- | --- | --- | --- |

| 0.554321901 14612 |  | tags=39%,  list=26%,  signal=29% |  | DNAJC13/CD14/IQGAP1/TYROBP  /CD9/CHRNB4/FCAR/SLC2A5/S  LCO4C1/STING1/PLAUR/STXBP2  /RND2/TCIRG1/ITGB2/FCER1G/L  GALS3/BST1/PAM/HLA-  H/SYTL4/RAB26/MGST1/ABCC4  /CPE/STBD1/SPACA3/TMEM184  A/PLAU/PECAM1/CR1/SIRPB1/  MANBA/SCG3/ADGRG3/FABP5/  C3AR1/BST2/FCGR3B/GLIPR1/PK  P1/CD177/CD93/CYBB/LILRB3/  ATP8B3/CD53/RPH3AL/ENPP4/A  DAM8/RAB44/TICAM2/SERPINA5  /CLEC4C/CD33/SLC30A8/IQGAP  2/RAB27B/CEACAM3/NFASC/M  OXD1/TNFRSF1B/LAIR1/PTAFR/I  TGAX/CD109/ADGRE3/NFAM1/R  AB31/RHOF/SIGLEC9/TRPM2/DM  BT1/SLC2A3/NCKAP1L/SIGLEC5  /SELL/ATP8B4/LILRB2/ANPEP/C  D300A/SIGLEC14/ACPP/ITPR2/F  PR1/LRMP/GPR84/LAMP3/ITGB3  /TBC1D10C/ITGAL/MS4A3/SYT1  /PTPRC/FPR2/ZPBP/P2RX1/SYT5  /MGAM/SLC27A2/CD38/SYT4/  DSC1/MMP25/FRMPD3/SYT9/SY  T2/CLEC12A/PIGR/ABCA13/DSG  1/FAM170B/SERPINB10/LY6G6F/  MOXD2P |
| --- | --- | --- | --- | --- |

| 0.554321901 15164 |  | tags=36%,  list=27%,  signal=27% |  | CX3CL1/PDPN/ADAM15/MACF1  /THBS3/PEAK1/COL3A1/CCN1/  COL17A1/NEXMIF/LRP1/GAS6/I  QGAP1/ID1/CSPG5/LAMB1/FGG  /EMP2/CDH13/NID1/CD34/PLET  1/BCL2L11/TECTA/COL26A1/FB  LN2/COL8A1/CSF1/EPDR1/EPHB3  /SRGAP2/ANGPT1/ITGA11/FERM  T3/NDNF/ITGB2/COL16A1/PREX  1/BST1/AGR2/FMN1/COL1A1/H  RG/RELL2/NID2/PLAU/PECAM1/  GREM1/UNC13D/JAG1/NRP1/KD  R/ANGPT2/MICALL2/ITGA6/COR  O2B/ECM2/VWC2/THY1/TNFRSF  12A/ITGA1/SIGLEC1/SPOCK1/R  AC2/FZD7/FAM107A/ITGA7/FN  1/THBS1/EPHA1/ITGA5/EPHA3/I  TGA10/SMOC2/SERPINE1/FGB/T  ACSTD2/ADAMTS12/COL5A3/SR  CIN1/CORO1A/ARHGAP6/SPOCK  2/VTN/ONECUT1/ADAMTS9/LA  MB4/VCAM1/ALOX15/PARVG/E  GFL6/MYO1G/SEMA3E/PLG/FOX  F1/NPNT/EDIL3/EGFLAM/HPSE/I  TGA2/PTPRO/CCL21/ITGB3/CCL  25/ITGAL/CASS4/CCR7/LAMB3  /MYOC/MIR503/CD3E/SKAP1/G  BP1/ITGA4/WNT1/CD96/MMP12  /MSLNL/PPFIA2/DMP1/FGA/NPY  2R/MYF5/DEFB118 |
| --- | --- | --- | --- | --- |

| 0.554321901 14185 |  | tags=35%,  list=25%,  signal=27% |  | AKAP6/ZP3/CHD7/CXCR4/DIO3  /SH3PXD2B/FGFR1/SEMA7A/CSF  1/SEMA5A/ACACB/RND2/FGF8/  AGR2/CXCL12/HAMP/BASP1/W  T1/NRP1/NGF/ISLR2/TNFRSF12A  /TRPV2/TBX5/MAPT/UNC13A/T  BX20/FN1/IGF2/GLI1/CRABP2/H  CLS1/DRD2/FOXS1/WNT2/IL7/  MYOD1/FGFR2/SYT14P1/POU3F2  /ARX/MAP1B/SLC6A3/SASH3/  MIR199A1/IGF1/NTN1/ZFPM2/A  TP8A2/NTRK3/SYT1/GATA6/CP  NE5/SYT4/DSCAM/SYT2/CPNE6  /CACNG7/REG1A/RIMS2/RIMS1  /POU4F2/GHSR/MIR204/MIR199B  /MIR208A |
| --- | --- | --- | --- | --- |

| 0.554321901 14588 |  | tags=45%,  list=26%,  signal=33% |  | TMEM64/GAS6/TREM2/CASP8/T  YROBP/IL34/KITLG/CD74/BTK/N  FKBIZ/TGFB1/TRIB1/CD4/CSF1/  EGR3/TAL1/HLA-  G/RUNX3/STAT1/ISG15/INHBA/  SYK/JAG1/ANXA1/PRKCA/FOX  C1/LGALS9/TNFSF9/NLRP3/PIK3  R6/LEF1/CCR1/CD80/LILRB4/TH  PO/ADAM8/VSIR/CA2/HCLS1/IL  7/CD101/TNFSF4/INPP5D/RUNX1  /CD86/EVI2B/IL1RL2/CCL19/FO  XP3/NCKAP1L/RHEX/SASH3/DU  SP10/GPR68/LILRB2/ROR2/RAB7  B/IL7R/IL18/GLI2/OCSTAMP/IL2  RA/POU4F1/PTPRC/IL15/MYB/LI  F/GATA3/IL23R/DCSTAMP/SHH  /IL12RB1/CD27/TNF/ZAP70/RAS  GRP1/TOX/TNFSF11/RHOH/TESP  A1/IL12B/IFNG/POU4F2/IL2 |
| --- | --- | --- | --- | --- |

| 0.554321901 11826 |  | tags=40%,  list=21%,  signal=31% |  | KHDC1/LGMN/PRR7/GPER1/TP63  /JAK2/FABP1/S100A8/DAPK1/N  AIP/POR/BAX/TBC1D10A/ARRB2  /MICAL1/RPS6KA1/CCN1/IFI6/G  AS6/S100A9/MDM2/CASP8/HIP  1/DHCR24/CTSH/BCL2L11/PLAU  R/IFI16/F3/FAS/SFRP2/SYK/NG  F/LGALS9/MT3/F2R/MAPT/NLR  P3/LEF1/CCK/CARD9/HGF/COL4  A3/BLID/THBS1/KLF4/CYFIP2/N  GFR/NLRC4/USP50/KHDC1L/AQP  1/CARD16/BCL2L10/TNFSF10/W  NT9A/TNFRSF10A/CASP1/SOX2  /SFN/TNFAIP8/PTGS2/CASP10/  CLEC7A/PSMB9/MMP9/FOXL2/C  ASP12/MEFV/BIRC3/LAMP3/CST  7/PAX2/TNFSF15/P2RX1/CASP1  4/CD27/TNFSF14/EPHA7/TNF/L  CK/TTBK1/MAGEA3/DPEP1/GRIN  1/GRIN2A/TFAP2B/AIM2/CSN2/  CASP5/ST18/FASLG/CARD18 |
| --- | --- | --- | --- | --- |

| 0.554321901 13238 | |  | | tags=34%,  list=23%,  signal=26% | |  | | ANK2/KIT/TNNI3K/TNNC2/CALC  RL/TNNI2/CACNA1C/NKX2-  5/PPP1R12B/HAMP/TRPM4/STC1  /AGT/PRKCA/MYBPH/CASQ2/A  IF1/MYOCD/CHGA/F2R/GATA5/  MYBPC3/KCNMA1/ADORA1/PI16  /TNNC1/SCN4A/GUCY1A1/HRC  /MYLK2/KLF4/EDN2/TNFRSF1B/  NOS1AP/ADRA2B/ADRA2A/PTAF  R/STRIT1/OXTR/SPHK1/CHRNA3  /CASQ1/ATP1A2/ADRA1B/PTGS  2/SLC8A1/DOCK4/MSTN/CAV3  /CHRM3/MIR199A1/CLIC2/NPNT  /CALCA/HCN4/IGF1/ITGA2/TNN  T2/SSTR2/PIK3CG/NMUR2/GJA5  /TACR1/ADRA1A/P2RX1/PLN/T  RPC3/NOS1/RYR2/KCNB2/MYMK  /TACR3/TIFAB/SLC8A3/NEUROG  1/KCNA1/CHRM2/NPY2R/GHSR/  DCANP1/MYOG/MIR199B/MIR208  A/MIR153-1 |
| --- | --- | --- | --- | --- | --- | --- | --- | --- |
| 0.554321901 2249 |  | | tags=3%,  list=4%,  signal=3% | |  | | CT45A10/CT45A1/CT45A3/CT45  A5 | |
| 0.554321901 9982 |  | | tags=63%,  list=18%,  signal=52% | |  | | DRD2/PENK/GRIN2D/GRIN3A/NPA  S1/SLC6A3/GLRB/NRXN1/CSMD  1/DRD3/GRIN1/GRID2/GRIN2A/S  LITRK6/DRD1/GLRA1/KCNA1 | |

| 0.554321901 14806 | |  | | tags=31%,  list=26%,  signal=23% | |  | | B2M/KDELR3/FZD5/HLA-  DPB1/CD9/HIP1/CD74/EGFR/CD4  /HLA-DRA/BTBD8/HLA-  G/HLA-H/FZD2/HLA-  DRB1/NCALD/CEMIP/NRGN/HLA  -DPA1/HLA-DRB5/HLA-  F/SLC18A1/SH3GL2/TGFA/SEC2  4D/WNT5A/HLA-  DQA2/SLC18A2/HLA-  DQB1/SLC17A7/HLA-  DQA1/HLA-  DQB2/RASSF9/LRP2/ROR2/IL7R/  AP1M2/KIAA0319/FOLR1/GRIA1  /FCGR1A/ADCY8/SYT1/AREG/F  CGR1B/EGF/SGIP1/SYT9/SYT2/  CD3D/GAD1/SLC32A1/CD3G/CH  RM2 |
| --- | --- | --- | --- | --- | --- | --- | --- | --- |
| 0.554321901 9827 |  | | tags=30%,  list=17%,  signal=25% | |  | | IL27RA/HLA-  DQB1/TNFSF4/FOXP3/CD28/IL10  /FGL2/CD40LG/POU2F2/BATF/P  TPRC/TBX21/GCNT3/XCL1/AICD  A/CCR6/IL2 | |

| 0.554321901 12415 |  | tags=49%,  list=22%,  signal=38% |  | JAK3/SYK/IRAK3/LGALS9/CMKL  R1/THBS1/IRF5/LILRA5/TNFSF4/  CCL19/CLEC7A/SCIMP/MEFV/LT  B/IL10/IRF8/CD40LG/LILRB1/IRF  1/TLR8/CCR7/IL23R/ISL1/SLAM  F1/IL12B/IDO1/TIGIT/IFNG |
| --- | --- | --- | --- | --- |
| 0.554321901 13038 |  | tags=33%,  list=23%,  signal=26% |  | PLEKHA1/TRPV4/PLEKHO1/FGD5/  GABRE/FGR/SPATA13/ABCA7/A  IF1/THY1/GABRG3/MAPT/FAM1  07A/ADORA1/JCAD/PLA2G4F/I  TGA5/GABRA5/LCP1/DPP4/PLEK  /KCNA2/PACSIN1/SCIMP/GABR  A4/RASGRP2/FGD2/KCNC1/MYO  1G/AMPH/UNC5A/HPCA/SHISA9  /KCNJ11/GRIA1/SHISA8/USH2A  /ITGB3/LAMP5/AKAP5/ADGRV1  /GABRA3/SHISA6/PSD2/GABRA  2/OPRD1/SLC1A2/ANK1/GABRA  1/CNTNAP2/REG1A/OPRM1/TAC  R3/GABRA6/GABRG2/KCNC2/GA  BRG1 |

| 0.554321901 11865 | |  | | tags=32%,  list=21%,  signal=26% | |  | | SIPA1L1/CTTNBP2/MT3/ADGRB1  /MAPT/NRGN/NTSR1/P2RX6/AD  ORA1/CAMK2A/SHANK1/HOMER  1/PDLIM4/DRD2/EPHA1/MX2/NG  FR/USP50/DNM3/EPHA4/SYNDIG  1/ATP1A2/SYT11/LRRC4/MAP1B  /DOCK10/PPP1R1B/SLC8A1/GPM  6A/ARC/ITPKA/GRM3/HPCA/GR  IA1/PTPRO/PRRT2/SEZ6/AKAP5  /CALB1/CD3E/OPRD1/NOS1/GRI  A4/GRIN1/ZNF804A/GRID2/GRIN  2A/ASIC2/FRMPD4/DRD1/KCNA  4/OPRM1/SLC8A3/KCND2/GRIA2 |
| --- | --- | --- | --- | --- | --- | --- | --- | --- |
| 0.554321901 8794 |  | | tags=45%,  list=16%,  signal=38% | |  | | GRIN3A/CHRNA3/CRHR1/SLC6A1  /GLRB/CHRM3/HTR2A/HTR1B/GL  DC/CHRNA4/SLC5A7/HTR2B/CH  RNA2/GLRA2/CRP/GRIN1/HTR1F  /CHRNA6/HTR5A/GLRA3/HTR3A  /GLRA1/CHRNB3/HTR1E/HTR2C | |

| 0.554321901 9501 |  | tags=57%,  list=17%,  signal=48% |  | GABRA5/GRIN2D/GRIN3A/CHRNA  3/GRID1/GABRA4/GABRD/GLRB/  GABRB2/GRIA1/GABRA3/GABRA  2/CHRNA4/GABRR2/GRIK1/CHRN  A9/CHRNA2/GLRA2/GRIA4/ADR  B1/GABRA1/GRIN1/GRID2/GRIN2  A/CHRNA6/GRIA3/GABRA6/GAB  RG2/GRIA2/GABRG1/GLRA1/CHR  NB3 |
| --- | --- | --- | --- | --- |
| 0.554321901 8313 |  | tags=59%,  list=15%,  signal=50% |  | SH2D1B/IL18RAP/VAV1/CD160/  CADM1/NCR3/KIR2DL4/SLAMF6/  RASGRP1/LAG3/IL12B/KLRC4-  KLRK1/CRTAM/CLNK/SH2D1A/K  LRK1/IL21 |

| 0.554321901 11549 |  | tags=51%,  list=20%,  signal=40% |  | CRHBP/DLG2/LRRC7/UNC13A/RG  S9/SHANK1/CACNG5/GSG1L/AR  C/RELN/SHISA9/GRIA1/SHISA8/  EPHB2/NEFL/CCR2/CACNG4/SHI  SA6/FRRS1L/RASGRF1/NETO1/C  ACNG3/GRIA4/CRH/CACNG7/GR  IN1/GRIN2A/GRIA3/OPRM1/GRIA  2/CACNG2/IFNG |
| --- | --- | --- | --- | --- |

| 0.554321901 15206 |  | tags=53%,  list=27%,  signal=39% |  | ICAM1/PLCB1/CX3CL1/PADI2/C  XCL14/ANO6/S100A14/GAS6/T  REM2/RARRES2/CD9/F7/ZP3/KIT  LG/THBS4/DAPK2/CD74/CSF1/H  MOX1/P2RX4/PGF/NBL1/LGALS3  /LYN/BST1/TRPV4/AIRE/CXCL1  2/PLA2G7/ELANE/GREM1/ANXA  1/CX3CR1/C3AR1/AIF1/THY1/L  GALS9/CCL20/CYP19A1/RAC2/  CCR1/CMKLR1/ADORA1/C5AR2/  SLIT2/MMP28/ADAM8/IL6/THBS  1/DYSF/WNT5A/TNFSF18/IL27R  A/CCN3/BDKRB1/EDN2/CCL7/SE  RPINE1/RIPK3/PTAFR/GCSAML/A  DTRP/CCL3/CCL19/NCKAP1L/CC  L5/CAMK1D/CD300A/MSTN/SL  AMF8/ECM1/CXCL17/PTGER4/IT  GA2/TNFRSF18/C10orf99/CCL21  /CCL25/CCL24/CCR7/FPR2/CCR  2/CCL4/CD200R1/GCSAM/CXCL  10/LBP/SPN/TNFSF14/STAP1/T  NF/ITGA4/CXCR3/XCL1/XCL2/  GPR18/S100A7/KLRC4-  KLRK1/CCR6/CXCL13/CCL1/KLR  K1 |
| --- | --- | --- | --- | --- |

| 0.554321901 12577 |  | tags=43%,  list=22%,  signal=33% |  | FOXJ1/JAK3/INHBA/SYK/LOXL3  /CR1/ANXA1/LGALS9/TNFSF9/  NLRP3/PIK3R6/CD80/LILRB4/AD  AM8/VSIR/IL7/IFNB1/TNFSF4/IN  PP5D/NFAM1/RUNX1/CD86/IL1R  L2/PTPN6/PGLYRP3/CCL19/FOXP  3/ZEB1/NCKAP1L/SASH3/DUSP1  0/LILRB2/HLA-  DOA/SLAMF8/IL7R/IL18/CD28/P  RDM1/GLI2/FGL2/IFNL1/NFATC2  /IL2RA/TNFRSF18/IRF1/ZNF683/  PTPRC/IL27/CCR2/CARD11/IL15  /MYB/GATA3/IL23R/CD2/SHH/I  L12RB1/CD27/TBX21/ZAP70/RA  SGRP1/TOX/SPINK5/IKZF3/LAG3  /RHOH/TESPA1/IL12B/PGLYRP2/  CTLA4/FCRL3/IFNG/IL2 |
| --- | --- | --- | --- | --- |

| 0.554321901 11473 |  | tags=25%,  list=20%,  signal=20% |  | MYOCD/TBX5/EFNB2/LEF1/FZD7  /TBX20/PI16/GLI1/TP73/TLL2/  NOG/DKK1/WNT2/MYOD1/FGFR  2/MYF6/WNT10B/LMOD3/FGF3/  NRG1/TWIST1/MSTN/CAV3/MIR  199A1/IGF1/ZFPM2/SIX4/GATA  6/SHH/TIFAB/NEUROG1/MYF5/D  CANP1/MYOG/MIR204/MIR199B/  MIR200B/MIR208A |
| --- | --- | --- | --- | --- |

| 0.554321901 14846 |  | tags=45%,  list=26%,  signal=33% |  | HDAC4/HAND2/UCMA/FAM20C/  LTF/BMP6/CEBPB/LIMD1/ATP2B1  /TP63/BMP2K/PTN/OMD/GLI3/S  EMA4D/BMP2/CEBPA/ANO6/CC  N1/SIX2/TMEM64/CITED1/EGR2  /IFITM5/ID1/ID3/ZHX3/TGFB3/  TGFB1/CSF1/TCIRG1/CEBPD/CO  MP/KL/TWIST2/S1PR1/HEMGN/  SFRP2/ISG15/TRPM4/GREM1/JA  G1/RASSF2/IFITM1/DHRS3/AHS  G/CCR1/HGF/NOCT/CHRD/GLI1  /IL6/WNT5A/NOG/DKK1/TGFB2  /FGFR2/RORB/BMP7/RUNX2/WN  T10B/CCL3/GDPD2/TMEM119/S  OX11/NPPC/SLC8A1/TWIST1/B  MPR1B/ECM1/PHOSPHO1/NPNT/  CALCA/IGF1/PTGER4/SOST/AD  GRV1/AREG/FGF23/TAC1/TNF/  OSR2/GDF10/NELL1/STATH/MIR  138-2/MIR208A |
| --- | --- | --- | --- | --- |

| 0.554321901 12596 |  | tags=23%,  list=22%,  signal=18% |  | PHACTR3/SFI1/PDGFRB/RCAN2/P  PP1R1C/DLG2/ITGA1/RIMBP2/CD  33/DRD2/TGFB2/RGN/SH3RF2/RI  PK3/PLEK/TMEM225B/GRXCR1/P  PP1R1B/NCKAP1L/CD300A/SPOC  D1/PPP1R1A/PPP1R14C/NPNT/IT  GA2/SAG/PPP2R2C/PTPRC/BOD1  L2/PPP1R2C/PPP1R2P1/TNF/PPP2  R2B/PCDH11X/AGTR2/SLC7A14/  TMEM132D/FCRL3/IFNG/PPP1R17 |
| --- | --- | --- | --- | --- |

| 0.554321901 11667 |  | tags=34%,  list=21%,  signal=27% |  | AIF1/CRHBP/HRH1/ALK/HTR7/G  ABRB3/OR11H7/RGS9/PTGER1/L  Y6H/HOMER1/HRH2/GNA15/HRH  4/DRD2/PENK/HTR4/PTAFR/OXT  R/LYPD1/CHRNA3/SLC6A3/PPP1  R1B/SLC6A1/GABRB2/CHRM3/PI  TX3/HTR1D/MTRNR2L5/SLC34A1  /ADCY8/HTR2A/HTR1B/CACNG4  /CALY/GLDC/PRKCG/CHRNA4/  ADGRL3/HTR2B/DRD5/TAC1/NS  G2/RGS8/CRH/GABRA1/DRD3/G  RIN1/SULT1A4/HTR1F/DRD1/OPR  M1/TACR3/HTR5A/GABRG2/HTR  3A/KCNC2/CHRM2/HTR1E/OR5T  2/OR56A5/HTR2C |
| --- | --- | --- | --- | --- |

| 0.554321901 13731 |  | tags=48%,  list=24%,  signal=37% |  | SERPINF2/ADORA2B/MYLK3/FGG  /KCNMB2/EGFR/TGFB1/EDNRA/  HMOX1/PIK3C2A/ANGPT1/COMP  /CDH5/PDE2A/PTP4A3/TRPV4/G  JA1/TRPM4/ECE1/AGT/FOXC1/  NPR1/HRH1/CHGA/F2R/ITGA1/H  TR7/KCNMA1/ADORA1/SLIT2/G  CH1/KCNMB4/P2RY2/GUCY1A1/  AVPR1A/HRH2/P2RY1/DDAH1/C  2CD4A/EDN2/ADRA2B/ADRA2A/  FOXC2/PTAFR/FGB/OXTR/ACE/  BDKRB2/KCNMB1/ATP1A2/ADRA  1B/C2CD4B/PTGS2/CASR/NPPC/  SLC8A1/DOCK4/CHRM3/CALCA  /MIR23A/HTR1D/GJA5/HTR2A/  ADCYAP1/SMTNL1/ADRA1A/P2R  X1/HTR1B/UTS2/CD38/ADRB3/H  TR2B/DRD5/NOS1/CRP/ADRB1/U  TS2R/ASIC2/DRD1/AGTR2/FGA/  MIR153-1 |
| --- | --- | --- | --- | --- |

| 0.554321901 11187 | |  | | tags=40%,  list=20%,  signal=32% | |  | | CD1D/PIK3R6/NCR1/LYZ/ULBP2/  HLA-  F/CCL13/C3/FCER2/CD1C/HTN3  /HTN1/RIPK3/GZMM/HAVCR2/C  D1E/CORO1A/CD1A/GNLY/PTPN  6/PGLYRP3/SLAMF7/IL18RAP/CL  EC7A/VAV1/REG3G/KRT6A/IL7R  /IL13/IL18/APOL1/ULBP1/CD160  /CTSG/C9/CADM1/CR1L/PRF1/  CD1B/LILRB1/KIR3DL1/PTPRC/GZ  MB/DCD/IL23R/IL12RB1/NCR3/K  IR2DL4/STAP1/XCL1/SLAMF6/R  ASGRP1/LAG3/IL12B/PGLYRP2/K  LRC4-  KLRK1/CRTAM/SH2D1A/KLRK1/  CD5L/IL21/IFNG/MBL2/SEMG1/  DEFB118/DEFA4/DEFB128 | |
| --- | --- | --- | --- | --- | --- | --- | --- | --- | --- |
| 0.554321901 6671 |  | | tags=47%,  list=12%,  signal=41% | |  | | CCR9/ITGB3/CCR7/ACKR1/CCR2  /CCR5/CCR3/ITGA4/CXCR3/CC  R4/CXCR6/CCR6/XCR1/CXCR5/  CCR8 | |  |

| 0.554321901 14544 |  | tags=34%,  list=26%,  signal=26% |  | LRP1/FZD5/HLA-  DPB1/CD9/HIP1/CD74/EGFR/CD4  /HLA-  DRA/WIPI1/BTBD8/PIK3C2A/FZD  2/AAK1/ABCB4/HLA-  DRB1/ECE1/UNC13D/NCALD/CE  MIP/ASTN2/NRGN/HLA-  DPA1/HLA-  DRB5/SLC18A1/SH3GL2/TGFA/  WNT5A/HLA-  DQA2/RAB27B/SLC18A2/HLA-  DQB1/SLC17A7/HLA-  DQA1/HLA-  DQB2/RASSF9/STON2/LRP2/ROR  2/ASTN1/IL7R/AP1M2/KIAA031  9/FOLR1/CLVS1/FCGR1A/ADCY  8/SYT1/CLVS2/AREG/FCGR1B/E  GF/SGIP1/CLRN1/SNAP91/SYT9  /SYT2/CD3D/MALL/CPNE6/GAD  1/SLC32A1/CD3G/CTLA4/CHRM  2 |
| --- | --- | --- | --- | --- |
| 0.554321901 11624 |  | tags=42%,  list=21%,  signal=33% |  | ARG2/LGALS9/NLRP3/ORM1/WN  T5A/NLRC4/USP50/LILRA5/CAR  D16/PANX2/CASP1/CCL3/CCL1  9/NOD2/IGHD/TLR8/CCR7/IL1R2  /NLRP7/ORM2/GBP5/AIM2/HMG  B4/CARD17/CARD18/NLRP10 |

| 0.554321901 11761 |  | tags=58%,  list=21%,  signal=46% |  | CCL18/CCL14/GPR183/CCL20/C  CL13/ADAM8/WNT5A/CCL7/CC  L8/CCL3/CCL19/CCL23/CCL11/  CCL5/CCL3L3/CCL16/PIK3CG/C  10orf99/CCL21/CCL25/CCL24/C  CL26/CCR2/CCL4/CXCL10/CXC  L11/TNFSF14/CXCR3/XCL1/CCL  22/XCL2/S100A7/KLRC4-  KLRK1/CXCL13/CCL1/KLRK1 |
| --- | --- | --- | --- | --- |

| 0.554321901 12157 |  | tags=35%,  list=22%,  signal=27% |  | CHRNA5/SV2A/ASIC1/SYT12/N  AAA/SNCG/GRIK5/DOC2B/UNC1  3A/CAMK2A/PRKCB/KCNMB4/SL  C6A9/P2RY1/DRD2/BRSK1/SLC1  8A2/KMO/GRIN3A/OTOF/CHRNA  3/SYT11/SNCAIP/NRXN2/CHAT  /TRIM9/CADPS/PRRT2/HTR2A/S  YT1/ADRA1A/P2RX1/HTR1B/SY  T5/PRKCG/GRM4/ADCY1/SYT4/  CHRNA4/SYT6/NRXN1/SLC5A7/  SYT9/SYT2/CPLX4/CPLX2/CHRN  A6/SLC22A2/DRD1/GAD1/RIMS2  /SLC32A1/PPFIA2/RIMS1/UNC13  C/CHRM2/CHRNB3/CPLX3 |
| --- | --- | --- | --- | --- |

| 0.554321901 13719 | |  | | tags=29%,  list=24%,  signal=22% | |  | | EGFR/SEMA7A/SEMA5A/RND2/S  LC44A4/SFRP2/CXCL12/HAMP/  NRP1/HPN/NGF/ISLR2/TNFRSF12  A/TRPV2/MAPT/LEF1/KRT17/UN  C13A/FN1/CRABP2/AVPR1A/H1  -  5/HCLS1/TGFB2/SYT14P1/SPHK1  /IGFBP1/MAP1B/SFN/MIR199A1  /IGF1/NTN1/NTRK3/SYT1/CPNE  5/CD38/SYT4/DSCAM/SYT2/CP  NE6/CACNG7/PRSS2/REG1A/RIM  S2/RIMS1/POU4F2/IL2/MIR208A |
| --- | --- | --- | --- | --- | --- | --- | --- | --- |
| 0.554321901 8770 |  | | tags=44%,  list=16%,  signal=37% | |  | | SPACA3/SIRPB1/C2/ABCA7/AH  SG/C3/RAB31/TULP1/CD300LF/  DOCK2/NCKAP1L/CLEC7A/CAMK  1D/IL2RG/IL1B/ITGA2/IL15RA/IL  2RB/PTPRC/FPR2/IL15/C4B/C4A  /STAP1/TNF/APOA2/SIRPG/IFN  G/MBL2 | |

| 0.554321901 9377 |  | | tags=30%,  list=17%,  signal=25% | |  | | ADGRB1/MYOCD/EFNB2/CD53/C  CL8/MYOD1/MYF6/LMOD3/NRG  1/CAV3/MIR199A1/NFATC2/IGF  1/SHH/TNFSF14/CXCL9/MYF5/  MYOG/MIR204/MIR208A | |
| --- | --- | --- | --- | --- | --- | --- | --- | --- |
| 0.554321901 12415 | |  | | tags=45%,  list=22%,  signal=35% | |  | | JAK3/SYK/LOXL3/ANXA1/LGAL  S9/NLRP3/CD80/TNFSF4/RUNX1  /CD86/CCL19/FOXP3/NCKAP1L  /SASH3/IL18/PRDM1/ZNF683/IL  27/MYB/GATA3/IL23R/SHH/IL1  2RB1/TBX21/ZAP70/IL12B/IFNG  /IL2 |

| 0.554321901 13108 |  | tags=44%,  list=23%,  signal=33% |  | LGALS3/LYN/CACNA1F/PLCL2/  THY1/KCNN4/LILRB4/PRKCB/CD1  9/GCSAML/NFAM1/RUNX1/PTPN  6/CD300A/CD160/PTPRC/CCR7/  PAX5/GCSAM/STAP1/GBP1/LCK  /PTPN22/UBASH3A/TESPA1/TRA  T1/FCRL3 |
| --- | --- | --- | --- | --- |
| 0.554321901 13703 |  | tags=39%,  list=24%,  signal=30% |  | TGFB1/FLT1/KIT/LYN/PDGFRB/F  GR/TNFAIP8L3/SOCS3/PIK3R6/K  LF4/CD19/EEF1A2/PIK3R5/PIK3R  3/AGAP2/CCL19/EPHA8/SOCS2  /NOD2/CCKBR/CCL21/CCR7/FPR  2/FLT3/SLA2/MIR138-2 |

| 0.554321901 11630 |  | tags=23%,  list=21%,  signal=18% |  | MSX1/ADGRB1/MYOCD/EFNB2/  MIR26A2/FZD7/PI16/CAMK1/IGF  2/CD53/CCN3/MAPK12/DKK1/C  CL8/MYOD1/FGFR2/MYF6/CDH2  /LMOD3/TMEM119/ZEB1/NRG1/  PRDM6/CAV3/MIR199A1/NFATC  2/IGF1/ANKRD2/CXCL10/SHH/T  NFSF14/BHLHA15/CXCL9/MYF5/  MYOG/MIR204/MIR199B/MIR200B  /MIR208A | |
| --- | --- | --- | --- | --- | --- |
| 0.554321901 513 |  | tags=6%,  list=1%,  signal=6% MIR107/MIR103A1 | | |  |

| 0.554321901 12388 |  | tags=46%,  list=22%,  signal=36% |  | PLAU/NEURL1/GREM1/TAFA1/D  KK3/NRP1/MTRNR2L9/CRHBP/PHL  DA2/DLG2/MTRNR2L2/LRRC7/M  TRNR2L3/ADORA1/RGS9/LY6H/S  HANK1/HOMER1/TGFA/LYPD6B/  P2RY1/NOG/DKK1/GPRC5A/NPT  X2/ADRA2B/ADRA2A/SERPINE1/  CACNG5/MTRNR2L6/DKK2/LYPD1  /MTRNR2L11/MTRNR2L1/CGA/M  TRNR2L10/NCF1/IL18BP/SLURP1/  MTRNR2L13/GSG1L/ESR2/ARC/C  AV3/IL10/RELN/SHISA9/GRIA1/  SHISA8/MTRNR2L5/EPHB2/NEFL/  ADH7/WFIKKN2/CCR2/CACNG4/  SHISA6/GREM2/EGF/RASGRF1/N  ETO1/MTRNR2L7/CACNG3/TAFA  4/GRIA4/CRH/CACNG7/GRIN1/G  RIN2A/CALCR/NPTX1/GRIA3/AG  TR2/OPRM1/HMGB4/GRIA2/CAC  NG2/IFNG |
| --- | --- | --- | --- | --- |

| 0.554321901 11473 |  | tags=46%,  list=20%,  signal=37% |  | MYOCD/F2R/KCNMA1/ADORA1/  GUCY1A1/EDN2/ADRA2B/ADRA2  A/PTAFR/OXTR/SPHK1/CHRNA3  /ATP1A2/ADRA1B/PTGS2/DOCK  4/CHRM3/NPNT/CALCA/ITGA2/  NMUR2/TACR1/ADRA1A/P2RX1/  KCNB2/TACR3/CHRM2/NPY2R/G  HSR/MIR153-1 |
| --- | --- | --- | --- | --- |
| 0.554321901 12124 |  | tags=62%,  list=21%,  signal=49% |  | CR1/LGALS9/LILRB4/PLA2G2D/V  SIR/TNFSF4/RUNX1/FOXP3/DUSP  10/LILRB2/CD28/NFATC2/IL2RA  /TNFRSF18/IRF1/FUT7/LAG3/CT  LA4/IFNG/IL2 |

| 0.554321901 11609 |  | tags=42%,  list=21%,  signal=34% |  | ACHE/ADGRB1/CLSTN2/ADD2/E  FNB2/GABRB3/SHANK1/IL1RAPL2  /WNT5A/DRD2/ADGRB3/LRRC24  /DNM3/DKK1/KIRREL3/THBS2/SL  ITRK5/OXTR/NLGN4Y/GPC6/SYN  DIG1/LRRC4/MAP1B/NRXN2/CDH  2/NLGN4X/SPOCK2/PCDH17/MD  GA1/SLITRK2/NRG1/GPM6A/AD  GRB2/CDH9/LHFPL4/GABRB2/GP  C4/LRFN5/NTN1/EPHB2/SDK2/N  TNG2/NTRK1/LRRTM1/SIX4/NTR  K3/POU4F1/LRRN3/AMIGO2/LRT  M1/GABRA2/DSCAM/SLIT1/PTP  RD/CNTN5/NRXN1/ADGRL3/CUX  2/EPHA7/SLITRK4/LRRTM2/SLITR  K1/GABRA1/GRIN1/GRID2/ASIC2  /SLITRK6/CBLN2/DRD1/LRTM2/  GJA10/GABRG2/SLITRK3/LRRTM  3/GHSR |
| --- | --- | --- | --- | --- |

| 0.554321901 12688 |  | tags=38%,  list=22%,  signal=30% |  | SFXN1/GJA1/CXCL12/FOLR3/SL  C46A1/INHBA/AGT/CUBN/SLC1  A3/ATP8B1/SLC47A1/SYT12/SN  CG/NTSR1/SLC6A9/CA2/SLC7A  3/DRD2/AQP1/SLC18A2/HBE1/S  LC6A20/AMN/HBD/STRA6/SLC1  7A7/CHRNA3/KCNA2/ATP1A2/S  YT11/SLC6A3/SYT10/SLC38A5/  LRP2/SLC35F3/RTBDN/SLC7A11/  RAB3B/SLC6A2/SLC1A1/SLC38A  3/GALR1/FOLR1/KCNJ10/SLC1A  7/SLC36A2/CALHM1/HTR2A/SL  C25A31/SYT1/HTR1B/SYT5/SLC  52A1/SLC13A5/SYT4/ABCC2/C  HRNA4/SYT6/IPCEF1/SLC5A8/S  YT9/SYT2/MB/SLC1A2/TCN1/C  RH/NGB/DRD3/CHRNA6/SLC22A  2/DRD1/SLC32A1/SLC17A8/SLC  17A6/NPY2R/GHSR/OR56A5 |
| --- | --- | --- | --- | --- |
| 0.554321901 13957 |  | tags=43%,  list=25%,  signal=32% |  | CD74/BCL2L11/GAPT/TCIRG1/L  YN/TSC22D3/JAK3/CACNA1F/L  AT/LGALS9/DOCK11/RIPK3/SPN  S2/LMO1/CORO1A/DOCK10/FO  XP3/NCKAP1L/TNFSF13B/IL2RA/  TNFSF14/SIT1/GPR174/TNFRSF13  B/TNFRSF17/IL2 |

| 0.554321901 13348 |  | tags=57%,  list=24%,  signal=43% |  | DLG4/ANK2/MAP2/GJC2/ERMN/  THY1/UCN3/CRHBP/DLG2/KCNA  B1/MAPT/NCMAP/SPOCK1/CCK  /LRRC7/ADORA1/CNTNAP1/NFA  SC/TNFRSF1B/KCNQ3/SCN1B/KC  NA2/MAP1B/KCNC1/MAP1A/KC  NJ11/MYOC/KCNQ2/CNTN2/SLC  1A2/ANK1/CRH/CNTNAP2/SCN1  A/CNGA3/SCN2A/KCNC2/KCNA  1 |
| --- | --- | --- | --- | --- |
| 0.554321901 13607 |  | tags=42%,  list=24%,  signal=32% |  | TBX1/GNB1L/DLG4/DACH1/CX3  CR1/CRHBP/SHANK1/AVPR1A/PE  NK/KIRREL3/OXTR/NLGN4Y/GRID  1/NRXN2/NLGN4X/PTCHD1/GRP  /POU4F1/EN1/GNG8/NRXN3/BRI  NP1/GCNT4/NRXN1/DRD3/GRIN1  /CNTNAP2/GAD1 |

| 0.554321901 8595 |  | | tags=44%,  list=15%,  signal=37% | |  | | CD1E/CD1A/PGLYRP3/IL18RAP/  CLEC7A/VAV1/CD160/CADM1/P  RF1/CD1B/PTPRC/IL23R/IL12RB1  /NCR3/STAP1/XCL1/SLAMF6/R  ASGRP1/LAG3/IL12B/PGLYRP2/K  LRC4-  KLRK1/CRTAM/SH2D1A/KLRK1/  CD5L/IL21/IFNG | |
| --- | --- | --- | --- | --- | --- | --- | --- | --- |
| 0.554321901 10901 | |  | | tags=45%,  list=19%,  signal=36% | |  | | CD80/CYBB/IL1A/IL6/THBS1/BC  L3/WNT5A/PTAFR/TLR7/CD86/  CLEC7A/EBI3/CD28/LTB/IL1B/IR  F1/TLR8/PTPRC/IL27/CCR2/CAR  D11/CD3E/LBP/SPN/TNF/APOA2  /TNFRSF8/IL12B/IL21/IFNG |

| 0.554321901 11624 | |  | | tags=41%,  list=21%,  signal=32% | |  | | LGALS9/NLRP3/ORM1/WNT5A/U  SP50/LILRA5/MNDA/HAVCR2/PA  NX2/CASP1/CCL3/CCL19/CLEC7  A/PYHIN1/NOD2/IGHD/TLR8/NL  RP7/ISL1/ORM2/AIM2/HMGB4/I  FNG/NLRP10 | |
| --- | --- | --- | --- | --- | --- | --- | --- | --- | --- |
| 0.554321901 8188 |  | | tags=53%,  list=14%,  signal=45% | |  | | DOCK2/FOXP3/BATF/PTPRC/LY9  /IL23R/CD3E/SHH/IL12RB1/SPN  /TBX21/CD3D/ZAP70/SLAMF6/  BCL11B/IL12B/CD3G/THEMIS | |  |

| 0.554321901 14280 |  | tags=46%,  list=25%,  signal=35% |  | PLCG2/AKAP6/EHD3/ATP1B1/CH  D7/STAC2/METTL21C/GPR35/G  NB5/ANK2/LYN/CACNB2/CACN  A1C/GJC2/FGF14/CACNB4/CAS  Q2/THY1/CEMIP/CACNA2D1/F2  R/NTSR1/CRACR2A/KCNE2/RGS  9/CACNG1/HRC/DYSF/DRD2/BD  KRB1/CD19/JPH1/CACNB1/REM1  /NOS1AP/F2RL3/ADRA2A/RGN/  STRIT1/APLNR/CASQ1/ATP1A2/  CORO1A/PTPN6/CRHR1/JSRP1/S  TAC/RRAD/SLC8A1/GEM/IL13/  CAV3/CLIC2/HPCA/PIK3CG/P2R  Y6/HAP1/PLN/CXCL10/CXCL11  /NOS1/XCL1/CXCL9/RYR2/MRL  N/TRDN/DRD3/CALCR/TMC1/DR  D1/OR56A5 |
| --- | --- | --- | --- | --- |
| 0.554321901 10383 |  | tags=28%,  list=18%,  signal=23% |  | IL6/IL27RA/CGAS/TNFSF4/FOXP  3/SASH3/TNFRSF4/IL13/CD28/I  L10/CD40LG/PTPRC/TBX21/TNF  /XCL1/MZB1/FCRL3/IL2 |

| 0.554321901 14284 |  | tags=46%,  list=25%,  signal=35% |  | ADORA2B/ZP3/FAM49B/BTK/PR  AM1/CLCF1/TGFB1/HMOX1/STX  BP2/HLA-  G/ITGB2/FCER1G/LYN/HLA-  H/FOXJ1/JAK3/TRPM4/DNASE1L  3/FGR/SYK/UNC13D/CR1/LGAL  S9/BST2/CD1D/NLRP3/PIK3R6/R  AC2/NCR1/CD177/HLA-  F/RSAD2/C3/IL6/FCER2/IL27RA  /CD1C/WAS/TNFRSF1B/IFNB1/R  IPK3/PTAFR/TNFSF4/HAVCR2/C  D1E/CD84/CD1A/PTPN6/SH2D1B  /IL18RAP/FOXP3/VAV1/SCIMP/  SASH3/CLC/CD300A/IL7R/IL13/  IL18/CD28/IL10/CD160/FOXF1/  CLEC4G/IL1B/CADM1/NOD2/CD  1B/LILRB1/PTPRC/CCR2/GATA3  /IL23R/IL12RB1/TBX21/NCR3/KI  R2DL4/TNF/XCL1/SLAMF6/RAS  GRP1/CD96/LAG3/LTA/IL12B/K  LRC4-  KLRK1/CRTAM/CLNK/SH2D1A/K  LRK1/IL21/IL2 |
| --- | --- | --- | --- | --- |

| 0.554321901 14507 |  | tags=54%,  list=26%,  signal=40% |  | TREM2/GDF15/TYROBP/CD9/EHD  2/MYOF/ADAM12/PLEKHO1/CA  CNA1H/ADGRB1/GCM1/CAMK1/  CD53/ADGRB3/CCL8/MYOD1/CD  109/MYF6/CAV3/NFATC2/OCST  AMP/DCSTAMP/CXCL10/TNFSF1  4/MYMX/NOS1/NPHS1/CXCL9/  WNT1/MYMK/MYF5/MYOG |
| --- | --- | --- | --- | --- |
| 0.554321901 12415 |  | tags=49%,  list=22%,  signal=38% |  | JAK3/LOXL3/ANXA1/LGALS9/G  PR183/NLRP3/LEF1/CD80/IL6/BC  L3/CLEC4E/TNFSF4/CD86/CCL19  /FOXP3/IL18/FGL2/IFNL1/PTGER  4/EOMES/BATF/IL27/LY9/MYB/  GATA3/IL23R/IL12RB1/SPN/TBX  21/SLAMF6/IL12B/IFNG/IL2 |

| 0.554321901 13007 |  | tags=32%,  list=23%,  signal=25% |  | SLC26A10/SLC4A11/SLC47A2/S  LC9A3/SLC26A9/SLC4A5/SLC24  A4/SLC22A4/SLC47A1/ATP1A3  /SLC26A7/SLC26A3/SLC8A1/SL  C26A8/SLC7A11/SLC9C2/SLC38  A3/SLC9A7/SLC25A31/SLC9A2  /SLC4A8/SLC35D3/SLC24A2/SL  C22A6/SLC8A3/SLC4A10/SLC9B  1P1 |  |
| --- | --- | --- | --- | --- | --- |
| 0.554321901 13410 |  | tags=48%,  list=24%,  signal=37% |  | ITGA11/FERMT3/ITGB2/COL16A1  /LYN/RET/SFRP2/HRG/PLAU/SY  K/FYB2/MUC1/ITGA5/CCN3/TG  FB2/SERPINE1/DPP4/FOXC2/PTP  N6/VTN/NCKAP1L/CCL5/ITGB8/  EPHA8/NPNT/ITGA2/PIK3CG/CCL  21/ITGB3/CD3E/SKAP1/ITGA4/  CXCL13 | |

| 0.554321901 12002 |  | tags=71%,  list=21%,  signal=56% |  | FCMR/TYROBP/ADORA2B/RAB23  /TCIRG1/HLA-  G/NCF2/CX3CR1/LY96/LSP1/NC  R1/FOSL1/MNDA/IL1RL2/NCF1/  GNLY/LILRB2/CD300C/CCR9/UM  OD/PRF1/CCR2/KLRC2/CCR5/KI  R3DL2/LBP/SPN/KIR2DL4/CCR3/  CXCL9/ITK/CCR6/KLRC3/SH2D1  A/NCR2/CD5L/TRAT1 |
| --- | --- | --- | --- | --- |
| 0.554321901 13330 |  | tags=47%,  list=24%,  signal=36% |  | TCIRG1/ITGB2/FCER1G/LGALS3/  STBD1/CR1/PKP1/CD93/ENPP4/  ADAM8/CLEC4C/NFASC/ITGAX/  ADGRE3/TRPM2/SLC2A3/NCKAP  1L/SIGLEC5/LILRB2/CD300A/SIG  LEC14/FPR1/TBC1D10C/FPR2/M  GAM/DSC1/DSG1/SERPINB10 |

| 0.554321901 11667 |  | tags=35%,  list=21%,  signal=27% |  | AIF1/LGALS9/F2R/C5AR2/IL27R  A/LILRA5/SYT11/TWIST1/IL1B/  TLR8/PAEP/CD200R1/BANK1/TN  F/PTPN22/IL17F/NLRP10/MIR204 |  |
| --- | --- | --- | --- | --- | --- |
| 0.554321901 11817 |  | tags=45%,  list=21%,  signal=35% |  | ASIC1/GJA4/P2RX2/CCK/GCH1  /THBS1/PENK/GABRA5/DPP4/LY  PD1/LPAR5/SCN9A/SLC6A2/REL  N/KIAA0319/CALCA/NTRK1/NM  UR2/ADCYAP1/TACR1/ADCYAP1  R1/BRINP1/PRKCG/PIRT/TAC1/N  OS1/ADRB1/CRH/DRD1/NEUROD  2/IDO1/MORC1/NPY2R/HTR2C | |

| 0.554321901 9783 |  | tags=35%,  list=17%,  signal=29% |  | CD244/IFNB1/HAVCR2/CORO1A/  PGLYRP3/SLAMF7/IFNW1/IL18/I  FNE/PRDM1/ULBP1/ZNF683/PTPR  C/IL15/IL23R/CD2/NCR3/RASGR  P1/PTPN22/TOX/IL12B/PGLYRP2  /KLRC4-  KLRK1/IL21R/CLNK/KLRK1/IFNA1  0/IFNA21/IL2 |
| --- | --- | --- | --- | --- |
| 0.554321901 8261 |  | tags=41%,  list=15%,  signal=35% |  | FCER1G/IRF7/CXCL12/TSC22D3/  JAK3/ARG2/HCLS1/CCL19/CCL5  /IL7R/NOD2/CCL21/LILRB1/CCR  7/CCR5/CD27/HSH2D/IDO1/GHS  R/IL2 |

| 0.554321901 7813 |  | | tags=46%,  list=14%,  signal=40% | |  | | DLG4/POU4F3/MYO7A/IGDCC3/  GRIN2C/USH1G/SLC1A3/SHANK1  /CNTNAP1/FOXS1/GPR88/CDH23  /USH1C/NEFL/POU4F1/NRXN1/C  LRN1/TNR/TIFAB/PCDH15/NEURO  G1/POU4F2/DCANP1/HMX3 | |
| --- | --- | --- | --- | --- | --- | --- | --- | --- |
| 0.554321901 11224 | |  | | tags=35%,  list=20%,  signal=28% | |  | | EFNB2/LRRC7/MX2/DNM3/KIF17  /MAPK10/GSG1L/ARC/KIF5C/HP  CA/ITGB3/CACNG4/CALY/KIF5  A/CACNG3/DRD3/CACNG2 |
| 0.554321901 12006 | |  | | tags=47%,  list=21%,  signal=37% | |  | | RCVRN/USH1G/RHO/GNAT1/DNM  3/MAK/TULP1/AIPL1/RDH12/US  H1C/SAG/USH2A/ADGRV1/PDC  /GNGT1/CRB1/PCARE/RD3/NOS  1/CERKL/RP1/PPEF2/GNAT2 |

| 0.554321901 12166 |  | tags=47%,  list=22%,  signal=37% |  | SYK/ANXA1/LGALS9/NLRP3/CD  80/TNFSF4/RUNX1/CD86/CCL19  /FOXP3/NCKAP1L/SASH3/IL18/  EBI3/CD28/CD160/PTPRC/RASAL  3/CCR2/MYB/IL23R/CD3E/SHH/  IL12RB1/ZAP70/XCL1/IL12B/IFN  G | |
| --- | --- | --- | --- | --- | --- |
| 0.554321901 11660 |  | tags=44%,  list=21%,  signal=35% |  | THY1/CEMIP/F2R/P2RX2/NTSR1  /BDKRB1/CD19/F2RL3/APLNR/C  ASQ1/P2RX5/IL13/P2RY6/HAP1  /ADCYAP1R1/CXCL10/CXCL11/  TRPC3/XCL1/CXCL9/TRDN/GRIN  1/CALCR/DRD1 |  |

| 0.554321901 11399 | |  | | tags=36%,  list=20%,  signal=29% | |  | | CLSTN3/DLG2/MAPT/LRRC7/SH  ANK1/WNT5A/RAB27B/KIF17/A  DAM22/MAPK10/GPC6/NRXN2/K  IF5C/MAP1A/RELN/GPC4/LRRTM  1/CACNG4/SHISA6/NETO1/NRX  N1/KIF5A/CACNG3/NPHS1/CAC  NG7/GRIN1/GRIN2A/ASIC2/CAC  NG2/GHSR |
| --- | --- | --- | --- | --- | --- | --- | --- | --- |
| 0.554321901 9982 |  | | tags=36%,  list=18%,  signal=30% | |  | | DRD2/PENK/STRA6/KCNA2/TRH  /CCL3/LEPR/RELN/HOXA1/HTR1  D/INSL5/HTR1B/SGIP1/CNTNAP4  /NRXN1/HTR2B/CRH/CSF2/DRD3  /MC4R/HCRTR2/TACR3/NPY2R/  GHSR/MC3R | |

| 0.554321901 13087 |  | tags=48%,  list=23%,  signal=37% |  | RUNX3/LGALS9B/JAK3/ARG2/L  OXL3/ANXA1/LGALS9/NLRP3/C  D80/VSIR/TNFSF4/RUNX1/CD86  /CCL19/FOXP3/NCKAP1L/SASH3  /IL18/CD160/IL27/MYB/GATA3  /IL23R/IL12RB1/TBX21/XCL1/IL  12B/IFNG/IL2 | |
| --- | --- | --- | --- | --- | --- |
| 0.554321901 536 |  | tags=6%,  list=1%,  signal=6% MIR185/MIR548P/MIR182 | | |  |
| 0.554321901 12150 |  | tags=44%,  list=21%,  signal=35% |  | ARG2/LGALS9/NLRP3/ORM1/WN  T5A/USP50/LILRA5/CARD16/PA  NX2/CASP1/CCL3/CCL19/NOD2  /IGHD/TLR8/CCR7/IL1R2/NLRP7  /ORM2/AIM2/HMGB4/CARD17/  CARD18/NLRP10 | |

| 0.554321901 14093 |  | tags=46%,  list=25%,  signal=34% |  | KITLG/CD274/CD74/BTK/FCER1G  /LGALS3/IRF7/LYN/CXCL12/TS  C22D3/JAK3/ARG2/ANXA1/LGA  LS9/GIMAP8/ADAM8/HCLS1/W  NT5A/HCAR2/RIPK3/CCL19/CCL  5/IL7R/SLC7A11/IL10/NOD2/CC  L21/LILRB1/CCR7/CCR5/LGALS1  3/CD27/HSH2D/IDO1/CD3G/PDC  D1/LGALS14/GHSR/IL2 |
| --- | --- | --- | --- | --- |
| 0.554321901 11502 |  | tags=32%,  list=20%,  signal=25% |  | ADGRB1/MYOCD/CD53/CCN3/C  CL8/MYOD1/MYF6/LMOD3/TME  M119/CAV3/NFATC2/ANKRD2/C  XCL10/TNFSF14/BHLHA15/CXCL  9/MYF5/MYOG/MIR200B |

| 0.554321901 14284 |  | tags=43%,  list=25%,  signal=32% |  | ADORA2B/CSPG5/ZP3/PRAM1/K  CNB1/HMOX1/STXBP2/SYP/ITGB  2/FCER1G/LYN/CACNB2/RAB15  /CACNA1H/FGR/SYK/CHRNA5/  UNC13D/LGALS9/SYT12/GRIK5/  RAC2/CD177/DOC2B/HLA-  F/PRKCB/RPH3AL/P2RY1/DRD2/  CDK5R2/GRIN3A/ADRA2A/PTAFR  /SYT11/CD84/SYT10/TRIM9/CD  300A/IL13/FOXF1/TRPV6/PRRT2  /HTR2A/SYT1/ADRA1A/P2RX1/  HTR1B/CCR2/SYT5/PRKCG/ADC  Y1/SYT4/CHRNA4/SYT6/SYT9/  SYT2/CPLX2/CACNA1G/CHRNA6  /CACNA1I/DRD1/RIMS2/PPFIA2  /RIMS1/CHRM2/CHRNB3/CPLX3 | |
| --- | --- | --- | --- | --- | --- |
| 0.554321901 11817 |  | tags=38%,  list=21%,  signal=30% |  | ASIC1/MT3/RGS9/DRD2/PDE1B/  AQP1/SLC18A2/OXTR/GUCY1B1  /CCL19/PPP1R1B/CCR7/DRD5/D  RD3/DPEP1/GRIN1/CNR2/GRIN2A  /DRD1/KCNC2 |  |

| 0.554321901 14110 |  | tags=40%,  list=25%,  signal=30% |  | CHD7/DIO3/NECTIN1/NECTIN3/T  CIRG1/ROM1/ARHGEF15/RET/M  AN2A1/SLC4A5/PDGFRB/NRP1/D  LX2/RPE65/THY1/ACHE/PRPH2/  COL4A1/FJX1/RHO/PAX6/RP1L1  /GNAT1/FOXN4/TGFB2/RORB/P  DE6C/SLC17A7/THRB/TULP1/CE  LF4/BMPR1B/GPM6A/DLL4/USH1  C/PROM1/MEGF11/HPCA/VSX1/  NDP/LAMA1/PDE6A/SDK2/GRM6  /PAX2/ATP8A2/CALB1/NEUROD  1/RD3/DSCAM/MFRP/NEUROD4/  NR2E3/CABP4/RP1/TFAP2B/LHX  1/GNAT2/SLC17A8/POU4F2 |
| --- | --- | --- | --- | --- |
| 0.554321901 11343 |  | tags=33%,  list=20%,  signal=27% |  | TUBB2B/EFNB2/SHANK1/NEFH/S  H3GL2/EPHA4/SYT11/LRRC4/GS  G1L/LRFN2/GRM1/PLG/INA/NTN  G2/NEFL/ADCY8/LRRC4C/ADCY  1/NRXN1/EPHA7/CACNG3/ADRB  1/LRRTM2/DGKB/TNR/GRM5/CA  CNG2/GHSR |

| 0.554321901 9501 |  | tags=55%,  list=17%,  signal=46% |  | GABRA5/GRIN2D/GRIN3A/CHRNA  3/GRID1/GABRA4/GABRD/GLRB/  GABRB2/GRIA1/GABRA3/GABRA  2/CHRNA4/GABRR2/GRIK1/CHRN  A9/CHRNA2/GLRA2/GRIA4/GAB  RA1/GRIN1/GRID2/GRIN2A/CHRN  A6/GRIA3/GLRA3/GABRA6/GAB  RG2/GRIA2/HTR3A/GABRG1/GLR  A1/CHRNB3 |
| --- | --- | --- | --- | --- |

| 0.554321901 12616 |  | tags=50%,  list=22%,  signal=39% |  | HRG/PROS1/POTEE/ITIH1/AGT/P  ZP/ACTG2/IGLV1-  47/AHSG/IGKV4-  1/ORM1/ANGPTL4/FN1/FCN3/C  3/SERPINC1/C8G/IGLV3-  21/POTEF/C1R/HBE1/IGKV3-  20/IGLC2/IGHA1/HBD/IGHM/FGB  /IGHA2/C1S/CFB/IGHG2/IGHG1  /ACTA1/IGHV3-  23/PON1/IGKC/VTN/IGKV3-  15/APOL1/IGKV1-  5/APCS/IGHG3/PLG/JCHAIN/IGL  C3/C9/IGHG4/IGHD/IGKV1-  17/IGKV3D-11/MIR503/IGHV3-  7/IGLV3-  25/SERPINA3/GC/C4B/IGHV3-  13/C4A/FCN2/ORM2/HPR/APOA  2/IGKV2D-28/ACTC1/IGKV1D-  12/IGKV2-30/IGKV1-  39/CD5L/IGKV1D-  33/FGA/PRSS1/AFM |
| --- | --- | --- | --- | --- |

| 0.554321901 11741 |  | tags=39%,  list=21%,  signal=31% |  | CASQ2/THY1/CEMIP/CACNA2D1  /F2R/NTSR1/HRC/PLCD4/DRD2/  BDKRB1/CD19/JPH1/GRIN2D/F2R  L3/APLNR/CASQ1/ATP1A2/COR  O1A/TRPM2/PTPN6/CCL3/CCL19  /JSRP1/SLC8A1/FAM155A/IL13  /ITPR2/CLIC2/TRPV6/PLCH2/CC  L21/P2RY6/PLCH1/HTR2A/PTPRC  /CCR7/HAP1/CCR5/PLN/CXCL1  0/CXCL11/HTR2B/NOS1/LCK/X  CL1/CXCL9/RYR2/TRDN/GRIN1/  GRIN2A/CALCR/DRD1/SLC8A3/F  ASLG/XCR1/HTR2C |
| --- | --- | --- | --- | --- |

| 0.554321901 13085 |  | tags=45%,  list=23%,  signal=35% |  | MCOLN2/RASA3/PANX1/P2RX4/  ATP2A3/PDE2A/CACNB2/TRPV4  /CACNA1C/CATSPER1/GRIN2C/  TRPM4/CACNA1H/CATSPER3/CA  CNA1F/ATP2C2/CACNB4/SLC24  A4/TRPV2/CACNA2D1/CACNG1  /TMEM37/PKD1L3/JPH1/CACNB1  /GRIN2D/GRIN3A/PKD2L1/CACN  G5/TRPM2/CACNA1E/SLC8A1/F  AM155A/TRPM8/CACNA2D3/GP  M6A/TRPC6/CACNA2D4/ITPR2/  TRPM3/TRPV6/CALHM1/TRPV3/  HTR1B/CACNG4/ATP2B2/CHRNA  9/CACNG3/TRPC3/RYR2/CACNA  1S/CACNG6/CACNG7/SLC24A2/  GRIN1/CACNA1G/GRIN2A/TMC1  /CACNA1I/OPRM1/SLC8A3/TRP  C7/CACNG2 |
| --- | --- | --- | --- | --- |
| 0.554321901 12026 |  | tags=58%,  list=21%,  signal=46% |  | CXCR4/IGSF1/NECTIN1/CD4/NRP  1/LY96/CD80/LILRA4/NGFR/GP  C6/CD86/ZP2/ROR2/CD28/GPC4  /ITGB3/ROR1/CCR5/CD8B2/ITG  A4/GPR15/CXCR6/CD8A/CD8B/  CCR8/FCRL1 |

| 0.554321901 10999 | |  | | tags=59%,  list=19%,  signal=47% | |  | | P2RX6/SHANK1/SLC6A9/CADM3  /FGFR2/NLGN4Y/SLC17A7/SYND  IG1/SYT11/LRRC4/NLGN4X/GRIA  1/CALB2/ADCY8/LRRTM1/AKAP  5/SYT1/SHISA6/NETO1/KCNJ3/  CACNG3/LRRTM2/GRIN1/GRID2/  GRIA3/KCNJ9/GRIA2/UNC13C/S  LC17A8/SLC17A6 | |
| --- | --- | --- | --- | --- | --- | --- | --- | --- | --- |
| 0.554321901 7857 |  | | tags=49%,  list=14%,  signal=42% | |  | | CLEC7A/LILRB2/LILRB1/KIR2DS4  /FCRL6/KLRD1/KIR3DL2/CD8B2/  TRAV8-4/CD8A/TRBV7-  9/TRGV9/TRAV29DV5/TRGV3/L  AG3/CD8B/KLRC4-  KLRK1/TRAV19/KLRK1/TRBV12-  3 | |  |

| 0.554321901 11662 | |  | | tags=51%,  list=21%,  signal=40% | |  | | VWC2/DLG2/GRIK5/LRRC7/SHA  NK1/GRIN2D/GRIN3A/CACNG5/C  PT1C/SHISA9/GRIA1/SHISA8/CA  CNG4/SHISA6/GRIK1/CACNG3/G  RIA4/CACNG7/GRIN1/GRID2/GRI  N2A/GRIA3/OLFM3/VWC2L/GRI  A2/HTR3A/CACNG2 |
| --- | --- | --- | --- | --- | --- | --- | --- | --- |
| 0.554321901 8595 |  | | tags=43%,  list=15%,  signal=37% | |  | | CD1E/CD1A/IL18RAP/VAV1/CD1  60/CADM1/CD1B/PTPRC/IL23R/I  L12RB1/NCR3/STAP1/XCL1/SLA  MF6/RASGRP1/LAG3/IL12B/KLR  C4-  KLRK1/CRTAM/SH2D1A/KLRK1/I  L21 | |

| 0.554321901 14175 |  | tags=56%,  list=25%,  signal=42% |  | ZP3/CD24/KITLG/CD274/CD74/  CLCF1/CD4/CSF1/CCDC88B/LYN  /BST1/PELI1/JAK3/CDKN1A/IGF  BP2/SYK/ANXA1/AIF1/LGALS9/  BST2/GPR183/TNFSF9/CD1D/HL  A-  DPA1/PDCD1LG2/CD80/IGF2/HL  A-  DMB/IL6/EPO/IL7/TNFSF4/HAVC  R2/CORO1A/CD86/CCL19/FOXP  3/ICOSLG/NCKAP1L/VCAM1/SA  SH3/LILRB2/CCL5/TNFRSF4/IL13  /IL18/CD209/EBI3/HHLA2/CD28  /CD6/NFATC2/OCSTAMP/CD40L  G/IGF1/IL1B/TNFSF13B/IL2RA/P  TPRC/CLECL1/RASAL3/CCR2/C  ARD11/IL15/IL23R/CD38/CD3E/  SHH/IL12RB1/TAC1/FGF10/ZAP7  0/XCL1/CD70/IL12B/IL21/FCRL3  /IL2 |
| --- | --- | --- | --- | --- |

| 0.554321901 12002 |  | tags=35%,  list=21%,  signal=28% |  | GRIN2C/CA7/CX3CR1/CLSTN3/S  LC1A3/SYT12/CLSTN2/NRGN/NT  SR1/FAM107A/UNC13A/ADORA1  /SHANK1/CA2/DRD2/CRHR2/KM  O/GRIN2D/NFATC4/OXTR/EPHA4  /PTGS2/LILRB2/ROR2/ARC/RAB  3B/KISS1/RELN/CALB2/EPHB2/P  RRT2/NTRK1/ADCY8/LRRTM1/A  DCYAP1/SYT1/CALB1/ADRA1A/  HAP1/CCR2/ADCY1/NETO1/NRX  N1/CUX2/SYT9/TAC1/TNF/SCG  N/LRRTM2/CRH/SLC24A2/GRIN1  /TNR/GRIN2A/DRD1/RIMS2/SLC  8A3/RIMS1 |
| --- | --- | --- | --- | --- |

| 0.554321901 15197 |  | tags=43%,  list=27%,  signal=31% |  | BAX/CX3CL1/RAMP3/NLGN3/ST  AC3/ATP2A1/ANO6/KCNE5/WN  K2/TREM2/PLCG2/AKAP6/EHD3/  ATP1B1/STAC2/ABCB1/ATPSCK  MT/CTSS/ANK2/CACNB2/ERFE/  GALR2/ATP1B2/GJC2/FGF14/AG  T/GPC3/THY1/CEMIP/CACNA2D  1/F2R/NTSR1/CRACR2A/GLRX/  KCNE1/KCNE2/RGS9/C3/CLIP3/  CA2/BDKRB1/CD19/NOS1AP/F2R  L3/RGN/LRRC38/PTAFR/APLNR/  CASQ1/KCNMB1/ITLN1/STAC/T  RPC6/ARC/IL13/KCNC1/KCNH2/  RELN/IGF1/KCNJ11/EPHB2/SLC3  4A1/P2RY6/LRRC55/HAP1/CCR2  /CACNG4/PIRT/CXCL10/CXCL11  /CACNG3/NOS1/XCL1/CXCL9/R  YR2/SLC1A2/LRRC52/TRDN/CAL  CR/DRD1/HTR3A/CACNG2/KCNC  2/KCNA1/IFNG/POU4F2 |
| --- | --- | --- | --- | --- |

| 0.554321901 11767 | |  | | tags=45%,  list=21%,  signal=35% | |  | | SLC24A4/KCND3/ATP1A3/KCNN  4/KCNAB1/KCNJ1/KCNU1/KCNT  1/GRIK5/KCNMA1/KCNE1/KCNE2  /KCNK6/KCNMB4/KCNN3/HCN2/  TRPM5/AQP1/KCNF1/KCNQ3/KC  NJ8/KCNV1/LRRC38/KCNV2/PKD  2L1/KCNA2/KCNMB1/ATP1A2/A  BCC9/KCNK1/ATP12A/KCNE1B/  KCNH7/ABCC8/KCNC1/KCNK10/  SLC9C2/KCNH2/KCNK9/HCN4/K  CNJ10/KCNJ11/SLC9A7/KCNK18  /KCNK13/KCNJ6/KCNT2/LRRC55  /SLC12A3/KCNS2/SLC9A2/KCN  Q2/KCNK3/GRIK1/KCNJ3/KCNA3  /KCNJ4/FXYD2/LRRC52/KCNH6/  KCNB2/SLC24A2/KCNG4/KCNJ9/  KCNA4/KCNJ16/KCND2/KCNH5/  KCNC2/KCNA1/KCNA10 | |
| --- | --- | --- | --- | --- | --- | --- | --- | --- | --- |
| 0.554321901 9242 |  | | tags=32%,  list=16%,  signal=27% | |  | | DLG2/LRRC7/ADAM22/MAPK10/  GPC6/GPC4/CACNG4/SHISA6/C  ACNG3/CACNG7/GRIN1/GRIN2A  /CACNG2/GHSR | |  |

| 0.554321901 14175 |  | tags=45%,  list=25%,  signal=34% |  | ZP3/FAM49B/CD274/BTK/NFKBIZ  /CLCF1/TGFB1/CD4/HLA-  G/FCER1G/IRF7/HLA-  H/FOXJ1/JAK3/TRPM4/IL1RL1/L  OXL3/CR1/ANXA1/CD1D/NLRP3  /CD80/HLA-  F/RSAD2/C3/IL6/FCER2/IL27RA  /CD1C/WAS/TNFRSF1B/IFNB1/R  IPK3/TNFSF4/HAVCR2/CD1E/CD  1A/PTPN6/CCL19/FOXP3/ALOX  15/SASH3/DUSP10/CLC/CD48/I  L7R/IL18/CD28/IL10/CD160/CLE  C4G/IL1B/TNFSF13B/CD1B/LILRB  1/IRF1/PTPRC/IL27/CCR2/SAMS  N1/GATA3/IL23R/IL12RB1/TBX2  1/SKAP1/TNF/XCL1/LTA/IL12B  /NLRP10/IL2 |
| --- | --- | --- | --- | --- |

| 0.554321901 12157 |  | tags=36%,  list=22%,  signal=29% |  | CHRNA5/SV2A/ASIC1/SYT12/S  NCG/GRIK5/NTSR1/UNC13A/CA  MK2A/PRKCB/KCNMB4/P2RY1/D  RD2/KMO/GRIN3A/MAOB/CHRN  A3/ATP1A2/SYT11/TRH/SNCAIP  /SLC6A1/RAB3B/PRRT2/ITGB3/L  ILRB1/HTR2A/SYT1/ADRA1A/P2  RX1/HTR1B/PRKCG/ADCY1/SYT  4/CHRNA4/SYT9/NOS1/CRH/CP  LX4/CPLX2/DRD3/CHRNA6/TNFS  F11/DRD1/RIMS2/PPFIA2/RIMS1  /CHRM2/CHRNB3/HTR2C/CPLX3 |
| --- | --- | --- | --- | --- |

| 0.554321901 13731 | |  | | tags=49%,  list=24%,  signal=37% | |  | | KCNMB2/EGFR/EDNRA/HMOX1/P  IK3C2A/COMP/GJA1/TRPM4/EC  E1/AGT/FOXC1/NPR1/HRH1/CH  GA/F2R/ITGA1/HTR7/KCNMA1/  ADORA1/GCH1/KCNMB4/P2RY2/  GUCY1A1/AVPR1A/HRH2/P2RY1  /EDN2/WNT9B/ADRA2B/ADRA2  A/FOXC2/PTAFR/FGB/OXTR/AC  E/BDKRB2/KCNMB1/ATP1A2/AD  RA1B/PTGS2/CASR/NPPC/SLC8  A1/DOCK4/CHRM3/CALCA/HTR  1D/GJA5/HTR2A/ADCYAP1/SMT  NL1/ADRA1A/P2RX1/HTR1B/UTS  2/CD38/ADRB3/HTR2B/DRD5/NO  S1/CRP/ADRB1/UTS2R/ASIC2/D  RD1/AGTR2/FGA/MIR153-1 | |
| --- | --- | --- | --- | --- | --- | --- | --- | --- | --- |
| 0.554321901 9827 |  | | tags=23%,  list=17%,  signal=19% | |  | | IL27RA/TNFSF4/FOXP3/CD28/IL  10/CD40LG/BATF/PTPRC/TBX21  /AICDA/CCR6/IL2 | |  |

| 0.554321901 12418 |  | tags=58%,  list=22%,  signal=45% |  | CA7/GABRE/PLCL2/CLSTN3/GA  BRG3/ADORA1/CA2/DRD2/NPY5  R/GABRA5/OXTR/SLC6A1/GABR  A4/GABRB2/ADRA1A/HAP1/HTR  1B/GABRA3/CNTNAP4/GABRA2/  TAC1/GABRA1/CNR2/GABRA6/  GABRG2/GABRG1 |
| --- | --- | --- | --- | --- |
| 0.554321901 11826 |  | tags=38%,  list=21%,  signal=30% |  | STAT1/NGF/TNFSF9/TNFSF18/T  NFSF4/TNFSF8/TNFSF10/LTB/CD  40LG/TNFSF13B/TNFSF15/TNFSF  14/TNF/CD70/TNFSF11/LTA/FA  SLG |

| 0.554321901 11293 |  | tags=38%,  list=20%,  signal=30% |  | PLCG2/CLCF1/GAPT/TGFB1/NKX  2-  3/PLCL2/GPR183/LFNG/DOCK11  /BCL3/IL27RA/MFNG/CD19/IFNB  1/TNFSF4/CDH17/CD180/DOCK1  0/FOXP3/CD28/IL10/CD40LG/B  ATF/PTPRC/TBX21/AICDA/CCR6  /IL2 |
| --- | --- | --- | --- | --- |
| 0.554321901 11040 |  | tags=47%,  list=20%,  signal=38% |  | SGK1/PRKCB/CACNG1/MCUB/GR  M2/REM1/NRXN2/RRAD/GEM/C  AV3/GRM3/NPY/CACNG4/PLN/  NRXN1/HPCAL4/CABP4/CACNG6  /CACNG7/CABP2/GRM7/NPY2R |

| 0.554321901 11667 |  | tags=36%,  list=21%,  signal=28% |  | AIF1/CRHBP/HRH1/ALK/HTR7/G  ABRB3/OR11H7/RGS9/PTGER1/L  Y6H/HRH2/GNA15/HRH4/DRD2/H  TR4/PTAFR/LYPD1/CHRNA3/GAB  RB2/CHRM3/HTR1D/MTRNR2L5/S  LC34A1/ADCY8/HTR2A/HTR1B/  CALY/CHRNA4/HTR2B/DRD5/NS  G2/RGS8/CRH/GABRA1/DRD3/S  ULT1A4/HTR1F/DRD1/OPRM1/HT  R5A/GABRG2/HTR3A/KCNC2/CH  RM2/HTR1E/OR5T2/OR56A5/HTR  2C |
| --- | --- | --- | --- | --- |

| 0.554321901 11361 | |  | | tags=58%,  list=20%,  signal=46% | |  | | GABRG3/P2RX2/GRIK5/P2RX6/G  ABRB3/GABRQ/GABRA5/GRIN2D  /GRIN3A/SLC17A7/CHRNA3/GRI  D1/P2RX5/GABRA4/GABRD/GLR  B/GABRB2/GRIA1/P2RX1/GABRA  3/GABRA2/CHRNA4/GABRR2/GRI  K1/CHRNA9/CHRNA2/GLRA2/GRI  A4/GABRA1/GRIN1/GRID2/GRIN2  A/CHRNA6/GRIA3/GABRR3/GLR  A3/GABRA6/GABRG2/GRIA2/HT  R3A/GABRG1/GLRA1/CHRNB3 | |
| --- | --- | --- | --- | --- | --- | --- | --- | --- | --- |
| 0.554321901 2249 |  | | tags=14%,  list=4%,  signal=14% | |  | | CT45A10/CT45A1/CT45A3/CT45  A5 | | |
| 0.554321901 3894 |  | | tags=27%,  list=7%,  signal=25% RNASE7/DEFB118/DEFA4 | | | | | |  |

| 0.554321901 11516 |  | tags=34%,  list=20%,  signal=27% |  | MT3/SULT1A2/NTSR1/SULT1A1/  SULT1E1/CYP4F12/SULT1B1/SYN  J2/MOXD1/MAOB/ADH4/SLC6A  3/MIOX/CYP24A1/INPP5J/SULT2  A1/ALDH3B2/ADH7/AKR1B10/FG  F23/CYP4A11/SULT1A4/HAO1/  GK2/MOXD2P |
| --- | --- | --- | --- | --- |
| 0.554321901 14937 |  | tags=57%,  list=26%,  signal=42% |  | LPAR1/CXCL14/ANO6/S100A14  /GAS6/TREM2/RARRES2/F7/CX  CR4/THBS4/DAPK2/CDH13/CD74  /TGFB1/FGFR1/CSF1/SEMA5A/P  2RX4/PGF/F3/TRPV4/S1PR1/IL1  6/ARTN/CXCL12/PLA2G7/PDGFR  B/NRP1/KDR/C3AR1/AIF1/LGAL  S9/TUBB2B/RAC2/CCR1/CMKLR1  /SLIT2/IL6/THBS1/DYSF/SCG2/  WNT5A/EDN2/CCL7/SMOC2/SE  RPINE1/FGF18/CCL3/CCL19/SUC  NR1/CASR/NCKAP1L/CCL5/CAM  K1D/MSTN/CXCL17/ITGA2/CCL  21/NTRK3/CCR7/FPR2/NTF3/CC  L26/CCR2/CCL4/CXCL10/DSCA  M/LBP/TNFSF14/FGF10/XCL1/C  CR4/XCL2/S100A7/CCR6/CXCL  13/CCL1 |

| 0.554321901 13512 | |  | | tags=49%,  list=24%,  signal=37% | |  | | ARMS2/ALB/NXNL1/POTEE/USH  1G/RPE65/CDHR1/RHO/LYZ/RP1  L1/POTEI/POTEF/MAK/IGHA1/IG  HA2/TULP1/ZG16B/IGKC/AIPL1/  RDH12/CDH23/USH1C/PROM1/IG  HG3/LCN1/JCHAIN/USH2A/POTE  J/ABCA4/ADGRV1/ESRRB/CNGB  1/CLRN1/RP1/PIGR/PCDH15 | |
| --- | --- | --- | --- | --- | --- | --- | --- | --- | --- |
| 0.554321901 4222 |  | | tags=17%,  list=7%,  signal=16% | |  | | CRH/CSF2/DRD3/HCRTR2/KCND2  /NPY2R/MC3R/NMS | | |
| 0.554321901 7341 |  | | tags=14%,  list=13%,  signal=12% | |  | | POM121L12/NPAP1/POM121L2/N  UP62CL | |  |

| 0.554321901 8188 |  | | tags=49%,  list=14%,  signal=42% | |  | | DOCK2/FOXP3/CD28/BATF/PTPR  C/CCR7/LY9/CARD11/IL15/GAT  A3/IL23R/CD3E/SHH/IL12RB1/SP  N/TBX21/CD3D/ZAP70/SLAMF6  /BCL11B/IL12B/CD3G/THEMIS | |
| --- | --- | --- | --- | --- | --- | --- | --- | --- |
| 0.554321901 14368 | |  | | tags=47%,  list=25%,  signal=35% | |  | | CHRNB4/FGF13/AKAP6/ATP1B1/  KCNMB2/KCNB1/P2RX4/GPR35/  ANK2/CACNB2/CACNA1C/GJA1  /TRPM4/CACNA1H/KCND3/CAC  NA2D1/NTSR1/KCNE1/KCNE2/GJ  C1/CLDN19/CNTNAP1/KCNMB4/  SCN4A/HCN2/FGF12/NOS1AP/S  CN1B/KCNJ8/SCN4B/KCNA2/AT  P1A2/SCN2B/SCN9A/KCNE1B/GP  R88/SLC8A1/CAV3/KCNH2/HCN  4/GJA5/SCN7A/NTRK3/ADRA1A  /P2RX1/CHRNA4/KCNJ3/TAC1/  RYR2/GJD2/CNR2/CACNA1G/CA  CNA1I/DRD1/SCN1A/SCN2A/KC  ND2/SCN3A/GLRA1/KCNA1/MIR  208A |

| 0.554321901 13330 |  | tags=49%,  list=24%,  signal=38% |  | TCIRG1/RELB/NKX2-  3/RUNX3/LGALS9B/JAK3/SYK/  ARG2/LOXL3/ANXA1/LGALS9/G  PR183/NLRP3/LEF1/CD80/PLA2G  2D/RSAD2/IL6/CLEC4A/BCL3/V  SIR/PAX1/TNFSF4/TNFSF8/RUNX  1/CD86/CCL19/DOCK2/FOXP3/  NCKAP1L/SASH3/CD300A/IL18/  EBI3/CD28/PRDM1/CD160/PTGER  4/EOMES/LILRB1/IRF1/BATF/ZN  F683/PTPRC/IL27/RASAL3/CCR2  /LY9/IL15/MYB/GATA3/IL23R/  CD3E/SHH/IL12RB1/SPN/TBX21/  ZAP70/XCL1/SLAMF6/FUT7/GPR  18/ITK/BCL11B/IL12B/IFNG/IL2 | |
| --- | --- | --- | --- | --- | --- |
| 0.554321901 10813 |  | tags=43%,  list=19%,  signal=35% |  | RNASE6/KLK5/H2BS1/IGKV3-  20/IGHA1/IGHM/FGB/IGHA2/H2  BC10/DMBT1/PGC/DEFB1/JCHAI  N/CTSG/SPINK5/RNASE7/SEMG2  /FGA/SEMG1/DEFA4 |  |

| 0.554321901 11015 | |  | | tags=42%,  list=19%,  signal=34% | |  | | LYZ/RNASE6/KLK5/CCL13/BCL3  /H2BS1/SPAG11A/HTN3/IGKV3-  20/HTN1/PRSS3/IGHA1/LCN2/IG  HM/PI3/FGB/IGHA2/H2BC10/ITL  N1/GNLY/DMBT1/PGLYRP3/PGC  /PLA2G2A/REG3G/KRT6A/DEFB1  /JCHAIN/CTSG/DCD/CXCL10/A  COD1/CXCL11/CXCL9/GALP/SPI  NK5/PRSS2/S100A7/RNASE7/RE  G1A/PGLYRP2/BPIFA2/SEMG2/C  XCL13/FGA/REG1B/REG3A/SEM  G1/DEFB118/DEFA4/CST9 |
| --- | --- | --- | --- | --- | --- | --- | --- | --- |
| 0.554321901 7476 |  | | tags=48%,  list=13%,  signal=41% | |  | | GABRA4/KCNC1/HPCA/SHISA9/  GRIA1/SHISA8/LAMP5/AKAP5/G  ABRA3/SHISA6/GABRA2/OPRD1  /GABRA1/REG1A/OPRM1/TACR3  /GABRA6/GABRG2/KCNC2/GABR  G1 | |

| 0.554321901 15160 | |  | | tags=54%,  list=27%,  signal=40% | |  | | PDPN/ADAM15/MMP19/MMP7/L  RP1/SCUBE3/KLK7/TGFB1/SH3PX  D2B/CTSS/KLKB1/ELANE/MMP11  /MMP2/HPN/HTRA1/MMP1/KLK5  /SCUBE1/ADAM8/IL6/TLL2/LCP  1/DPP4/CARMIL2/CTRB1/ADAM  TS4/ADAMTS5/CMA1/MMP9/TL  L1/PLG/CTSG/TPSAB1/KLK4/M  MP20/TMPRSS6/MMP16/PRSS2/  CTRB2/MMP12/MMP13/MMP8/PR  SS1 |
| --- | --- | --- | --- | --- | --- | --- | --- | --- |
| 0.554321901 7476 |  | | tags=87%,  list=13%,  signal=75% | |  | | GABRA5/GABRA4/GLRB/GABRB2  /GABRA3/GABRA2/GLRA2/GABR  A1/GLRA3/GABRA6/GABRG2/G  ABRG1/GLRA1 | |

| 0.554321901 10399 |  | tags=37%,  list=18%,  signal=30% |  | SLC6A9/RAB27B/SLC17A7/SYT1  1/RAB3B/TMEM163/SYT1/SYT4  /GABRA2/OPRD1/SYT9/RAB3C/  SLC32A1/SV2C/SLC17A8/SLC17  A6/CPLX3 |  |
| --- | --- | --- | --- | --- | --- |
| 0.554321901 11458 |  | tags=53%,  list=20%,  signal=43% |  | SCNN1G/GABRG3/KCNJ1/P2RX2  /GRIK5/P2RX6/GABRB3/KCNE2/  KCNK6/GABRQ/HCN2/AQP1/JPH  1/GABRA5/GRIN2D/GRIN3A/KCN  J8/SLC17A7/CHRNA3/GRID1/TR  PM2/KCNK1/P2RX5/SCNN1B/SC  NN1A/GABRA4/GABRD/KCNH2/G  LRB/ITPR2/GABRB2/HCN4/KCNJ1  0/KCNJ11/GRIA1/KCNJ6/P2RX1  /GABRA3/CNGB3/CNGA1/GABR  A2/CHRNA4/GABRR2/CNGB1/GRI  K1/CHRNA9/CHRNA2/KCNJ3/KC  NJ4/GLRA2/GRIA4/RYR2/GABRA  1/GRIN1/GRID2/GRIN2A/CHRNA6  /ASIC2/GRIA3/GABRR3/KCNJ9/  KCNJ16/CNGA3/GLRA3/GABRA6  /GABRG2/GRIA2/HTR3A/GABRG  1/GLRA1/CHRNB3/ASIC5/KCNA1  0 | |

| 0.554321901 14413 | |  | | tags=60%,  list=26%,  signal=44% | |  | | GDF15/CD9/EHD2/MYOF/ADAM  12/PLEKHO1/CACNA1H/ADGRB1  /CD53/ADGRB3/CCL8/MYOD1/  MYF6/CAV3/NFATC2/CXCL10/T  NFSF14/MYMX/NOS1/NPHS1/CX  CL9/WNT1/MYMK/MYF5/MYOG | |
| --- | --- | --- | --- | --- | --- | --- | --- | --- | --- |
| 0.554321901 11125 | |  | | tags=43%,  list=20%,  signal=35% | |  | | NTSR1/ADORA1/GCH1/KCNK6/D  RD2/BDKRB1/PTAFR/GPR37L1/A  DM2/CALCA/GJA5/ADRA1A/AD  RB3/FFAR3/DRD5/NOS1/ADRB1/  CRH/DRD3/AGTR2 | |
| 0.554321901 8439 |  | | tags=58%,  list=15%,  signal=50% | |  | | SSTR1/MCHR1/GALR1/SSTR2/N  MUR2/ADCYAP1R1/GPR149/OPR  D1/MC4R/OPRM1/SSTR3/MRGPR  X2/SSTR5/MC3R | |  |

| 0.554321901 12166 |  | tags=43%,  list=22%,  signal=34% |  | SYK/ANXA1/LGALS9/NLRP3/CD  80/TNFSF4/RUNX1/CD86/CCL19  /FOXP3/NCKAP1L/SASH3/IL18/  MYB/IL23R/SHH/IL12RB1/ZAP70  /IL12B/IFNG |  |
| --- | --- | --- | --- | --- | --- |
| 0.554321901 15206 |  | tags=53%,  list=27%,  signal=39% |  | ICAM1/CX3CL1/CXCL14/ANO6/  S100A14/GAS6/TREM2/RARRES2  /F7/ZP3/KITLG/THBS4/DAPK2/  CD74/CSF1/P2RX4/PGF/LGALS3  /TRPV4/CXCL12/PLA2G7/ELANE  /CX3CR1/C3AR1/AIF1/THY1/LG  ALS9/CCL20/RAC2/CCR1/CMKL  R1/ADAM8/IL6/THBS1/DYSF/W  NT5A/TNFSF18/BDKRB1/EDN2/C  CL7/SERPINE1/PTAFR/CCL3/CCL  19/NCKAP1L/CCL5/CAMK1D/MS  TN/CXCL17/ITGA2/TNFRSF18/C  CL21/CCL24/CCR7/FPR2/CCR2/  CCL4/CXCL10/LBP/SPN/TNFSF1  4/TNF/ITGA4/XCL1/XCL2/S100  A7/CCR6/CXCL13/CCL1 | |

| 0.554321901 8169 |  | | tags=58%,  list=14%,  signal=50% | |  | | IL18RAP/VAV1/CD160/CADM1/  NCR3/SLAMF6/RASGRP1/LAG3/I  L12B/KLRC4-  KLRK1/CRTAM/SH2D1A/KLRK1/I  L21 | |  |
| --- | --- | --- | --- | --- | --- | --- | --- | --- | --- |
| 0.554321901 11326 | |  | | tags=50%,  list=20%,  signal=40% | |  | | KCND3/KCNN4/KCNAB1/KCNJ1/  KCNU1/KCNT1/GRIK5/KCNMA1/  KCNE1/KCNE2/KCNK6/KCNMB4/  KCNN3/HCN2/TRPM5/AQP1/KCN  F1/KCNQ3/KCNJ8/KCNV1/LRRC3  8/KCNV2/PKD2L1/KCNA2/KCNM  B1/ABCC9/KCNK1/KCNE1B/KCN  H7/ABCC8/KCNC1/KCNK10/KCN  H2/KCNK9/HCN4/KCNJ10/KCNJ1  1/KCNK18/KCNK13/KCNJ6/KCNT  2/LRRC55/KCNS2/KCNQ2/KCNK3  /GRIK1/KCNJ3/KCNA3/KCNJ4/L  RRC52/KCNH6/KCNB2/KCNG4/K  CNJ9/KCNA4/KCNJ16/KCND2/K  CNH5/KCNC2/KCNA1/KCNA10 | |

| 0.554321901 856 |  | tags=33%,  list=2%,  signal=32% |  | HAMP/GREM1/MYOCD/EFNB2/FZ  D7/PI16/DKK1/NRG1/CAV3/MIR  199A1/IGF1/MIR204/MIR199B/MI  R200B/MIR208A | |
| --- | --- | --- | --- | --- | --- |
| 0.554321901 12415 |  | tags=50%,  list=22%,  signal=39% |  | JAK3/LOXL3/ANXA1/LGALS9/N  LRP3/CD80/TNFSF4/RUNX1/CD8  6/CCL19/FOXP3/NCKAP1L/SASH  3/IL18/IL27/MYB/GATA3/IL23R  /IL12RB1/TBX21/IL12B/IFNG/IL2 | |
| 0.554321901 11624 |  | tags=56%,  list=21%,  signal=44% |  | LGALS9/PIK3R6/NCR1/HLA-  F/HAVCR2/SH2D1B/IL18RAP/VA  V1/CD160/CADM1/LILRB1/NCR3  /KIR2DL4/SLAMF6/RASGRP1/CD  96/LAG3/IL12B/KLRC4-  KLRK1/CRTAM/CLNK/SH2D1A/K  LRK1/IL21 |  |

| 0.554321901 10063 | |  | | tags=28%,  list=18%,  signal=23% | |  | | P2RY1/DRD2/BRSK1/DNM3/GRIN  3A/SLC17A7/SYT11/CDH2/PCD  H17/RAB3B/PRRT2/HTR2A/SYT1  /ADRA1A/P2RX1/HTR1B/PRKCG  /ADCY1/SYT4/CHRNA4/NRXN1/  SYT9/CPLX2/CHRNA6/DRD1/RIM  S2/PPFIA2/RIMS1/CHRM2/CHRNB  3/CPLX3 |
| --- | --- | --- | --- | --- | --- | --- | --- | --- |
| 0.554321901 12415 | |  | | tags=55%,  list=22%,  signal=43% | |  | | JAK3/IL1RL1/ANXA1/LEF1/CD80  /BCL3/IL27RA/TNFSF4/HAVCR2  /CCL19/IL18RAP/IL18BP/IL18/E  BI3/IL1B/IL27/CCR2/IL23R/IL12R  B1/SPN/XCL1/IL12B/NLRP10 |
| 0.556059731 2249 |  | | tags=1%,  list=4%,  signal=1% | |  | | CT45A10/CT45A1/CT45A3/CT45  A5 | |

| 0.556059731 12415 |  | tags=57%,  list=22%,  signal=44% |  | JAK3/IGFBP2/SYK/ANXA1/AIF1  /LGALS9/TNFSF9/CD1D/HLA-  DPA1/PDCD1LG2/CD80/IGF2/HL  A-  DMB/IL6/EPO/TNFSF4/HAVCR2/  CORO1A/CD86/CCL19/FOXP3/IC  OSLG/NCKAP1L/VCAM1/SASH3/  LILRB2/CCL5/IL18/CD209/EBI3/  HHLA2/CD28/CD6/CD40LG/IGF1  /IL1B/TNFSF13B/IL2RA/PTPRC/C  LECL1/RASAL3/CCR2/CARD11/I  L15/IL23R/CD3E/SHH/IL12RB1/Z  AP70/XCL1/CD70/IL12B/IL21/IL  2 |
| --- | --- | --- | --- | --- |

| 0.556059731 14185 | |  | | tags=46%,  list=25%,  signal=35% | |  | | AKAP6/CHD7/METTL21C/ANK2/  LYN/CACNA1C/CASQ2/THY1/C  EMIP/F2R/NTSR1/HRC/BDKRB1/  CD19/JPH1/F2RL3/APLNR/CASQ  1/ATP1A2/CORO1A/PTPN6/JSRP  1/SLC8A1/IL13/CLIC2/P2RY6/H  AP1/PLN/CXCL10/CXCL11/NOS  1/XCL1/CXCL9/RYR2/TRDN/DR  D1 |
| --- | --- | --- | --- | --- | --- | --- | --- | --- |
| 0.556059731 6299 |  | | tags=26%,  list=11%,  signal=23% | |  | | PRRT2/HTR2A/SYT1/ADRA1A/P2  RX1/HTR1B/PRKCG/ADCY1/SYT  4/CHRNA4/SYT9/CPLX2/CHRNA  6/DRD1/RIMS2/PPFIA2/RIMS1/C  HRM2/CHRNB3/CPLX3 | |
| 0.556059731 2249 |  | | tags=13%,  list=4%,  signal=13% | |  | | CT45A10/CT45A1/CT45A3/CT45  A5 | |

| 0.562299253 11421 |  | tags=42%,  list=20%,  signal=33% |  | CHGA/NLRP3/MPEG1/CARD9/LY  Z/RNASE6/IL6/LYG1/H2BS1/IL2  7RA/STAB2/TNFSF8/HAVCR2/H2  BC10/LYZL2/DMBT1/PGLYRP3/P  LA2G2A/REG3G/KRT6A/DEFB1/G  BP4/CTSG/C10orf99/GBP2/LYZL  1/GBP7/LBP/TNF/CRP/FCN2/GB  P6/RNASE7/LTA/PGLYRP2/KLRC  4-  KLRK1/KLRK1/MBL2/LALBA/DEF  A4/DEFB128/LYZL4 |
| --- | --- | --- | --- | --- |

| 0.562299253 13719 |  | tags=44%,  list=24%,  signal=33% |  | EGFR/FLT1/FGFR1/EDNRA/PLA2  G5/KIT/TXK/PDGFRB/AGT/AVPR  1A/GNA15/APOC2/FGFR2/PTAF  R/SELE/ARHGAP6/CCL5/ESR1/H  PCA/NMUR2/P2RY6/S1PR4/NTRK  3/HTR2A/ADRA1A/NTF3/ADCY  AP1R1/HTR2B/ITK/GPR55/PNLIP/  MIR182 | |
| --- | --- | --- | --- | --- | --- |
| 0.570219966 12054 |  | tags=63%,  list=21%,  signal=50% |  | ANXA1/CD80/IL27RA/CCL19/IL  18/IL1B/CCR2/IL23R/IL12RB1/X  CL1/IL12B/NLRP10 |  |

| 0.58838935 3699 |  | tags=15%,  list=7%,  signal=14% |  | OR13G1/OR5M8/OR1E1/OR11G2/  OR2Z1/OR1J2/OR8H1/OR10A5/O  R52K2/OR11L1/OR5B2/OR9Q2/O  R2A4/OR51I1/OR6V1/OR10D3/O  R6S1/OR12D2/OR4D1/OR9A1P/O  R13C5/OR2AJ1/OR6C65/OR2J3/  OR4D11/OR1D2/OR13C2/OR14C3  6/OR4F6/OR2M7/OR10D4P/OR6C  1/OR51B6/OR13F1/OR10P1/OR4  C5/OR52K1/OR9I1/OR2D3/OR2W  1/OR6K6/OR2B2/OR1N2/OR2L3/  OR6F1/OR2B8P/OR1C1/OR2L2/O  R2A25/OR52I1/OR5B12/OR2A14  /OR2T8/OR2A2/OR6A2/OR10A2  /OR11A1/OR1P1/OR5G3/OR10J3  /OR2A5 |
| --- | --- | --- | --- | --- |

| 0.607864358 3699 |  | tags=14%,  list=7%,  signal=13% |  | OR13G1/OR5M8/OR1E1/OR11G2/  OR2Z1/OR1J2/OR8H1/OR10A5/O  R52K2/OR11L1/OR5B2/OR9Q2/O  R2A4/OR51I1/OR6V1/OR10D3/O  R6S1/OR12D2/OR4D1/OR9A1P/O  R13C5/OR2AJ1/OR6C65/OR2J3/  OR4D11/OR1D2/OR13C2/OR14C3  6/OR4F6/OR2M7/OR10D4P/OR6C  1/OR51B6/OR13F1/OR10P1/OR4  C5/OR52K1/OR9I1/OR2D3/OR2W  1/OR6K6/OR2B2/OR1N2/CNGA2  /OR2L3/OR6F1/OR2B8P/OR1C1/  OR2L2/OR2A25/OR52I1/OR5B12/  OR2A14/OR2T8/OR2A2/OR6A2/  OR10A2/OR11A1/OR1P1/OR5G3/  OR10J3/OR2A5 |
| --- | --- | --- | --- | --- |

| 0.648367857 14942 |  | tags=29%,  list=26%,  signal=22% |  | MPP2/KIF18A/TIAM2/ACTA2/SY  NE2/CTNNA2/AC004151.1/IQGA  P1/SHISA7/CAPG/ASAP3/KITLG  /PARD6A/CXCR4/PDE4A/WIPF1  /PLEKHG5/KCNB1/TLN2/SRGAP2  /LDB2/PLEKHA1/CDH1/TRPV4/P  TPN13/AAK1/PLEKHO1/KLHL41/  ABLIM3/FGD5/GABRE/FGR/DPYS  L3/SPATA13/ABCA7/AIF1/THY1  /FGD3/ACTG2/TNFRSF12A/GAB  RG3/FRMD4B/MAPT/RAC2/CD17  7/RAB3IP/FAM107A/ADORA1/J  CAD/COBL/PLA2G4F/DYSF/PDLI  M4/IQGAP2/HCLS1/ITGA5/LAYN  /GABRA5/LCP1/DPP4/PLEK/CAR  MIL2/KCNA2/SRCIN1/NHS/CORO  1A/CDH2/PACSIN1/BSPRY/ACT  A1/GDPD2/ABI3/SCIMP/APBB1IP  /GABRA4/RASGRP2/FGD2/KCNC  1/INPP5J/MEFV/MYO1G/PSTPIP1  /AMPH/UNC5A/HPCA/SHISA9/U  NC5C/KCNJ11/GRIA1/SHISA8/P  TPRO/USH2A/ITGB3/LAMP5/AK  AP5/TUBB3/ADGRV1/SAMSN1/G  ABRA3/SHISA6/STMN2/PSD2/G  ABRA2/CLRN1/OPRD1/SKAP1/SL  C1A2/ANK1/GABRA1/ACTC1/C  NTNAP2/REG1A/OPRM1/TACR3/  GABRA6/GABRG2/KCNC2/GABRG  1 |
| --- | --- | --- | --- | --- |

| 0.648367857 14792 |  | tags=52%,  list=26%,  signal=39% |  | C3B/LGALS1/LAMA5/F2/GH1/S  PARC/TGM2/FBN1/IGFBP7/COL2  0A1/ICAM1/ANGPTL2/THBS3/W  NT2B/COL9A1/COL3A1/CCN1/C  OL18A1/COL17A1/GPC1/THSD4  /CTSC/COL8A2/S100A9/RARRE  S2/GDF15/MMRN2/SERPINF2/F7  /HAPLN1/CTSH/SSC5D/ZP3/CLE  C14A/LAMB1/FGG/COL21A1/TH  BS4/CDH13/NID1/C1QA/TGFB3/  TECTA/COL26A1/FBLN2/COL8A1  /TGFB1/SEMA7A/CTSS/LUM/C  OL15A1/COCH/C1QC/COMP/F3  /COLQ/COL16A1/C1QB/MUC2/  LGALS3/MFAP4/LAMA3/PCOLCE  /SFRP2/COL1A1/HRG/RELL2/CX  CL12/NID2/CFP/ELANE/TINAGL1  /COL6A3/GREM1/ITIH1/AGT/M  MP2/ANXA1/LOXL2/PZP/SBSPO  N/HTRA1/EMILIN3/ITGA6/COL4  A2/SULF1/ANGPTL6/GPC3/ECM  2/COL10A1/VWC2/LGALS9/AC  HE/COL4A1/LRRC15/SERPINA1/  ASPN/AHSG/SPON1/EYS/COL6A  5/FREM3/COL4A3/TGFBI/TPSB2  /PRG4/ORM1/ADAM11/ELN/COL  9A2/ENTPD2/FN1/MMP28/COL5  A1/MXRA5/THBS1/SERPINC1/SE  RPINA5/TNC/WNT5A/SPARCL1/  S100A4/CCN3/NCAM1/WNT2/T  GFB2/APLP1/IL7/SMOC2/SERPIN  E1/PODNL1/ADAMTS8/LMAN1L/  THBS2/SERPINB8/P3H2/FGFR2/V |
| --- | --- | --- | --- | --- |

| 0.648367857 11266 |  | tags=45%,  list=20%,  signal=36% |  | CCL20/TNFSF9/IL17B/GDF11/GD  F6/OSM/CCL13/THPO/IL1A/IL6  /SCG2/IL1RN/EPO/WNT5A/TNF  SF18/INHBB/WNT2/TGFB2/CCL7  /IL7/IFNB1/CCL8/TNFSF4/TNFSF  8/TNFSF10/BMP7/GDF3/GDF5/C  CL3/CCL19/CCL23/CCL11/SLUR  P1/NRG1/IFNW1/CCL5/IL11/MS  TN/LEFTY2/IL13/IL18/IFNE/EBI3  /IL32/CCL3L3/LTB/IL10/CCL16  /SECTM1/IFNL1/NDP/CD40LG/IL  1B/TNFSF13B/CNTF/C10orf99/C  CL21/CCL25/TNFRSF11B/CCL24/  TNFSF15/IL27/CCL26/CCL4/IL15  /AREG/LIF/GREM2/CXCL10/IL26  /CXCL11/TNFSF14/IL37/TNF/X  CL1/CXCL9/CCL22/XCL2/WNT1  /IL19/CSF2/CD70/GDF10/TNFSF  11/LTA/IL12B/IL36G/FASLG/CX  CL13/CCL1/IL21/IFNG/IL17F/IFN  A10/BMP15/IL22/IFNA21/IL2 |
| --- | --- | --- | --- | --- |

| 0.648367857 15208 |  | tags=57%,  list=27%,  signal=42% |  | CXCL1/CX3CL1/PADI2/CXCL16/  CXCL14/ANO6/HSD3B7/CCL17/  S100A14/GAS6/S100A9/RARRES  2/F7/IL1F10/CXCR4/THBS4/DAP  K2/CD74/FLT1/CSF1/PGF/NBL1  /KIT/ITGB2/FCER1G/LGALS3/LY  N/PREX1/BST1/TRPV4/S1PR1/IL  16/CXCL12/PLA2G7/TRPM4/GRE  M1/SYK/ANXA1/CCL18/C3AR1  /AIF1/LGALS9/CCL14/HRH1/CH  GA/GPR183/CCL20/ITGA1/CYP1  9A1/RAC2/CCR1/CMKLR1/C5AR  2/CCL13/SLIT2/MMP28/IL1A/A  DAM8/IL6/THBS1/DYSF/SCG2/I  L1RN/WNT5A/CSF3R/CCN3/EDN  2/TGFB2/CCL7/TNFRSF11A/SERP  INE1/CCL8/CORO1A/TRPM2/JA  ML/CCL3/CCL19/CCL23/CCL11  /NCKAP1L/VAV1/FFAR2/CCL5/  CAMK1D/MSTN/SLAMF8/CCL3L3  /IL10/CXCL17/CCL16/CALCA/I  L1B/PTPRO/PIK3CG/C10orf99/CC  L21/CCL25/CCL24/CCR7/FPR2/  CCL26/CCR2/CCL4/CCR5/CXCL  10/LBP/CXCL11/TNFSF14/STAP  1/TAFA4/IL37/CXCR3/XCL1/C  XCL9/CCL22/XCL2/CNR2/GPR18  /S100A7/TNFSF11/KLRC4-  KLRK1/CCR6/IL36G/CXCL13/CX  CR5/CCL1/KLRK1 |
| --- | --- | --- | --- | --- |

| 0.648367857 13257 |  | tags=36%,  list=23%,  signal=28% |  | SLC5A9/SLC38A6/SLC13A4/SLC  4A11/ATP1B2/NKX2-  5/SLC9A3/SLC4A5/TRPM4/CAC  NA1H/CATSPER3/ASIC1/SLC24A  4/SLC22A4/SCNN1G/ATP1A3/G  LRX/SGK1/SLC4A4/SCN4A/HCN  2/TRPM5/FGF12/DRD2/SCN1B/P  KD2L1/SCN4B/SLC17A7/ATP1A2  /SCN2B/FXYD7/TRPM2/KCNK1/  SCN9A/SLC38A4/ATP12A/SCNN  1B/SCNN1A/SLC8A1/SLC38A8/  SLC5A4/SLC34A2/CAV3/SLC9C  2/HECW2/SLC38A3/HCN4/SLC5  A12/SLC9A7/SLC34A1/HECW1/  SLC28A3/SCN7A/SLC12A3/SLC9  A2/SLC13A5/SLC4A8/NETO1/SL  C5A7/SLC5A8/NOS1/ABCB11/F  XYD2/SLC24A2/DRD3/CACNA1G  /ASIC2/CACNA1I/SCN1A/SCN2  A/SLC8A3/SLC13A2/SLC4A10/  SCN3A/SLC17A8/SLC17A6/SLC9  B1P1/SLC17A1/ASIC5/SLC17A3 |
| --- | --- | --- | --- | --- |

| 0.648367857 11908 |  | tags=41%,  list=21%,  signal=33% |  | PRKCA/PTGER3/ADGRG1/ADGRG  3/MC1R/UCN3/ADGRB1/NPR1/C  HGA/OR51E2/EPHA5/ADGRL2/A  DCY4/PTGER1/PEX5L/RASD2/TH  BS1/DRD2/ADGRB3/AQP1/CGAS  /APLP1/ADRA2B/ADRA2A/GCGR  /PTGFR/MRAP/APLNR/ADGRE3/  PTGIR/GUCY1B1/ADRA1B/CRHR1  /NPPC/TSHR/ADGRG6/UCN2/AD  M2/ADGRB2/DEFB1/FSHR/GALR1  /CALCA/PTGER4/PTHLH/GPR65/  GPHA2/S1PR4/ADCY8/ADCYAP1  /ADRA1A/ADCYAP1R1/ADRB3/  ADCY1/PLN/CXCL10/MC5R/SCT  R/ADGRE1/ADGRL3/CXCL11/AD  CY2/HTR2B/DRD5/NOS1/LGR5/  ADGRG4/ADRB1/CXCL9/ADGRG5  /CRH/DRD3/CALCR/MC4R/DRD1  /RIMS2/OPRM1/HTR5A/RIT2/GU  CY2F/CALCB/KCNC2/NPY2R/OR  56A5/HTR2C/MC3R |
| --- | --- | --- | --- | --- |

| 0.648367857 13377 |  | tags=37%,  list=24%,  signal=29% |  | SIX1/FGF8/POU4F3/CEBPD/SLC4  4A4/MYO7A/C1QB/FZD2/GRHL3  /JAG2/ECE1/PDGFRB/MAFB/JA  G1/HPN/USH1G/FRZB/ATP8B1/M  SX1/DLX5/MYCL/GABRB3/PVAL  B/WNT5A/NOG/ZIC1/TGFB2/GA  BRA5/FGFR2/STRA6/EPHA4/PRR  X1/GRXCR1/SOX2/NKX3-  2/ZEB1/ROR2/TWIST1/CDH23/U  SH1C/GLI2/HES5/GABRB2/HPCA  /HOXA1/ATOH1/NTN1/EPHB2/U  SH2A/PHOX2B/GBX2/PAX2/ROR  1/SIX4/ATP8A2/NTRK3/CALB1/  ADGRV1/FOXG1/GATA3/NEUROD  1/PLPPR4/SHH/KCNK3/ESRRB/CL  RN1/CHRNA9/FGF10/LGR5/OSR2  /WNT1/LHX3/TMC1/SLITRK6/TI  FAB/PCDH15/MYO3B/SLC17A8/  NEUROG1/LHFPL5/DCANP1/HMX3 |
| --- | --- | --- | --- | --- |

| 0.648367857 14104 |  | tags=38%,  list=25%,  signal=29% |  | CD24/KITLG/ZFYVE28/THBS4/N  OX4/CD74/BLK/BTK/CLCF1/EGF  R/TGFB1/FLT1/DOCK3/FGFR1/C  D4/ANGPT4/EPHB3/ANGPT1/FGF  8/SH2B3/DLG4/TAL1/KIT/ITGB2  /LYN/BST1/HIPK4/RET/TIE1/TX  K/SFRP2/HRG/JAK3/PDGFRB/SPI  NK1/NEURL1/PECAM1/GREM1/F  GR/SYK/AGT/NRP1/KDR/CSF1R  /PARP9/THY1/ALK/EPHA5/SOC  S3/CCK/HGF/OSM/CD80/ADOR  A1/LRP8/IGF2/IL6/TGFA/HCLS1  /EPO/EPHA1/ITGA5/EPHA3/PRLR  /TNFSF18/IL27RA/LILRA5/GPRC5  A/IL7/ADRA2A/MATK/HCK/GH2  /FGFR2/EPHA4/ACE/AFAP1L2/S  RCIN1/NCF1/PTPN6/CSHL1/VTN  /DOK7/EPHA6/NRG1/TDGF1/CC  L5/ROR2/IL11/CD300A/EPHA8/I  L13/IL18/HES5/RELN/SLA/IFNL1  /IGF1/CNTF/EPHB2/TNFRSF18/IT  GB3/FCGR1A/NTRK1/CASS4/RO  R1/NTRK3/HTR2A/PTPRC/ADRA1  A/NTF3/SAMSN1/IL15/AREG/LI  F/IL23R/EGF/FLT3/CD3E/BANK1  /EPHA7/STAP1/FGF10/TNF/EPH  A10/LCK/TTBK1/ZAP70/ISL1/C  SF2/STYK1/ITK/IL12B/IL31RA/G  RM5/IL22RA2/IL21/IFNG/IL2 |
| --- | --- | --- | --- | --- |

| 0.648367857 13329 |  | tags=34%,  list=24%,  signal=26% |  | KCNK4/KIT/FAS/PDE2A/TRPV4/  PPL/STAT1/GJA1/COL1A1/CXC  L12/MEIS2/IGFBP2/CHI3L1/AGT  /HPN/ANGPT2/SLC1A3/SCX/TH  BS1/TNC/P2RY1/FOSL1/DRD2/P  KD1L3/BDKRB1/AQP1/STRA6/PK  D2L1/ATP1A2/TNFRSF10A/TLR7  /MAP1B/NRXN2/GDF5/ACTA1/  CASP1/PTGS2/SLC8A1/IL13/CA  V3/KCNC1/SCEL/KIAA0319/PTG  ER4/IL1B/ITGA2/NTRK1/IRF1/TL  R8/ATP8A2/HTR2A/SOST/ADGR  V1/CXCL10/NRXN1/CHRNA9/TN  FSF14/RYR2/TNFRSF8/ASIC2/TM  C1/SLITRK6/CNTNAP2/SCN1A/C  ASP5/TIFAB/ANO3/NEUROG1/KC  NA1/LHFPL5/CSRP3/DCANP1 |
| --- | --- | --- | --- | --- |

| 0.648367857 13510 |  | tags=33%,  list=24%,  signal=26% |  | SYP/HLA-DRA/HLA-  G/PAM/HLA-H/SYTL4/HLA-  DRB1/RAB26/SYNGR3/CPE/SLC1  7A5/TMEM184A/NCALD/SV2A/  SCG3/SYT12/CHGA/NRGN/HLA-  DPA1/HLA-DRB5/HLA-  F/UNC13A/SLC18A1/RPH3AL/SL  C6A9/SH3GL2/TGFA/SEC24D/SL  C30A8/HLA-  DQA2/DRD2/RAB27B/SLC18A2/  HLA-  DQB1/OTOF/SLC17A7/SYT11/S  YT10/SORL1/HLA-DQA1/HLA-  DQB2/RASSF9/RAB3B/TMEM163  /AMPH/FOLR1/GRIA1/PRRT2/LA  MP5/PTPRN/SYT1/SYT5/AREG/  SYT4/GABRA2/SYT6/OPRD1/SY  T9/SYT2/RAB3C/SCGN/DRD3/S  LC32A1/SVOP/SV2C/UNC13C/S  LC17A8/SLC17A6/OR56A5/CPLX  3 |
| --- | --- | --- | --- | --- |

| 0.648367857 14956 |  | tags=37%,  list=26%,  signal=27% |  | SCN5A/LPAR1/DRAXIN/EMX2/F  ABP7/B2M/WNT2B/COL3A1/SY  NE2/LRP1/TYROBP/DCLK1/PLXN  A3/FGF13/NRG3/WDR62/CHD7/  STIL/LAMB1/CXCR4/EGFR/FGFR  1/SEMA7A/SEMA5A/PITX1/EPH  B3/SRGAP2/SEMA3A/FGF8/CDH  1/RTN4RL1/CXCL12/OTP/TBX19  /NOTCH3/INHBA/TACC2/NRP1/  CSF1R/DLX2/ADGRG1/MSX1/DL  X5/NKX2-  1/ALK/EPHA5/NRGN/LEF1/PAX6  /TAL2/SLIT2/CHRD/LRP8/GLI1/  AVPR1A/BMERB1/SRGAP2C/WNT  5A/DRD2/CDK5R2/NOG/EMX1/F  EZ1/DKK1/AQP1/NR0B1/KIRREL3  /GSX2/SLITRK5/FGFR2/SALL3/  OXTR/DAB1/POU3F2/HSD3B2/A  RX/CDH2/SLC6A3/MFSD2A/BCL  2A1/SSTR1/SOX2/NRG1/CHD5/  PTCHD1/SLC8A1/LRP2/CCKAR/P  LXNA4/SLC7A11/KCNC1/RAX/G  LI2/HES5/RELN/OTX2/OLIG2/AT  OH1/SOX3/EPHB2/SSTR2/EOMES  /NEFL/GBX2/POU4F1/ADCYAP1  /NPY/HAP1/NEUROG3/FOXG1/P  AX5/NEUROD1/FEZF1/CNTN2/SL  IT1/SHH/UNCX/FGF10/ISL1/SLC  1A2/WNT1/FEZF2/CRH/FOXB1/  GRIN1/LHX3/TNR/BCL11B/CNTN  AP2/DRD1/SLC32A1/HTR5A/LHX  1/SLC8A3/SLC4A10/SSTR3/TBR  1/KCNC2/KCNA1/NKX2-6 |
| --- | --- | --- | --- | --- |

| 0.648367857 14684 |  | tags=53%,  list=26%,  signal=39% |  | CCL17/S100A14/TREM2/S100A9  /RARRES2/CD9/IL1F10/MCOLN2  /THBS4/DAPK2/CD74/FLT1/CSF  1/P2RX4/PGF/NBL1/KIT/ITGB2/  FCER1G/LGALS3/LYN/PREX1/BS  T1/TRPV4/CXCL12/PLA2G7/PEC  AM1/GREM1/SYK/ANXA1/CX3C  R1/CCL18/C3AR1/AIF1/CCL14/  HRH1/CHGA/CCL20/ITGA1/CYP1  9A1/RAC2/CD177/CCR1/CMKLR  1/C5AR2/CCL13/SLIT2/MMP28/  IL1A/ADAM8/IL6/THBS1/DYSF/  SCG2/IL1RN/CSF3R/CCN3/EDN2  /TGFB2/CCL7/TNFRSF11A/SERPI  NE1/CCL8/JAML/CCL3/CCL19/  CCL23/CCL11/NCKAP1L/VAV1/  CCL5/ROR2/CAMK1D/CD300A/  MSTN/SLAMF8/CCL3L3/CXCL17  /CCL16/CALCA/PTGER4/IL1B/P  TPRO/PIK3CG/CCL21/UMOD/CCL  25/CCL24/CCR7/FPR2/CCL26/C  CR2/CCL4/CD200R1/CXCL10/LB  P/CXCL11/STAP1/TAFA4/IL37/  XCL1/CXCL9/CCL22/XCL2/S100  A7/TNFSF11/IL36G/CXCL13/CC  L1 |
| --- | --- | --- | --- | --- |

| 0.648367857 14185 |  | tags=34%,  list=25%,  signal=25% |  | AKAP6/MYLK3/SDC1/LDB3/XK/  TNNT3/MYOF/SIX1/COMP/ANK2  /LMOD2/NKX2-  5/KLHL41/NRAP/HAMP/KLHL40/  PDGFRB/AGT/CASQ2/P2RX2/PI1  6/HOMER1/LMOD1/DYSF/UCHL1  /NFATC4/MYOD1/ALPK3/MYF6  /MYH11/CASQ1/WNT10B/LMOD  3/ACTA1/PGM5/MYOZ2/SLC8A  1/CAV3/MIR199A1/NFATC2/SY  NPO2L/IGF1/MIR23A/TNNT2/NEB  L/SIX4/ADRA1A/WFIKKN2/ALPK  2/MYPN/ACTC1/MYOM3/AGTR2  /CSRP3/MYF5/NKX2-  6/MYOG/MIR199B/MIR208A |
| --- | --- | --- | --- | --- |

| 0.648367857 13703 |  | tags=30%,  list=24%,  signal=23% |  | TGFB1/TRIB1/SEMA7A/HMOX1/  ANGPT4/SEMA5A/NBL1/SRGAP2  /SEMA3A/CLDN5/CDH1/GPR173  /TIE1/DACH1/GJA1/SFRP2/HRG  /CXCL12/SEMA3G/SRGAP1/ELA  NE/STC1/GREM1/DPYSL3/JAG1  /NRP1/ANGPT2/CX3CR1/ADGRG  1/SULF1/MIR2355/IFITM1/AIF1/  THY1/PTPRU/BST2/NKX2-  1/ADGRB1/MYOCD/TBX5/SEMA  3F/MIR26A2/HAS1/CYP19A1/V  ASH1/ADORA1/C5AR2/SLIT2/T  MIGD3/CHRD/MMP28/THBS1/BM  ERB1/SRGAP2C/WNT5A/KLF4/D  RD2/EPHA1/NOG/IL27RA/CCN3/  SEMA6B/WAS/RGN/SERPINE1/MI  R212/TACSTD2/SRGAP2B/ADTRP  /ADAMTS9/SLURP1/MIR193A/N  RG1/DUSP10/TMEFF2/NDRG4/CD  300A/SLAMF8/DLL4/PTPRR/SEM  A3E/MARVELD3/PTGER4/PTPRO/  CCL21/CCL25/MIR503/GATA3/C  D200R1/GCSAM/SHH/KRT16/ST  AP1/FRMD5/DPEP1/GPR18/AGTR  2/PTPRT/KLRC4-  KLRK1/SEMG2/SPOCK3/CXCL13/  KLRK1/SEMG1/GHSR/MIR204/MI  R129-2/MIR138-2/MIR218-  1/MIR200B/MIR182 |
| --- | --- | --- | --- | --- |

| 0.648367857 10600 |  | tags=29%,  list=19%,  signal=24% |  | SLCO2B1/SLC18A1/CES1/MSR1/  SLC16A8/P2RY1/DRD2/APOC2/  AQP1/SLC18A2/ADRA2B/ADRA2  A/LIPG/MAOB/STRA6/OXTR/KC  NA2/SYT11/SLC6A3/MIR27B/CR  HR1/SYT10/PON1/LIPC/RAB3B/E  MB/SLCO1A2/SLC6A2/GALR1/S  LC5A12/ABCC3/ITGB3/LILRB1/H  TR2A/POMC/SYT1/P2RX1/HTR1  B/ABCG4/SYT5/MYB/EGF/AQP3  /SYT4/AKR1C4/ABCC2/CHRNA4  /SYT6/SHH/SLC5A8/SYT9/SYT  2/FFAR3/TAC1/NOS1/ABCB11/  APOA2/CRH/DRD3/CHRNA6/DRD  1/AGTR2/MIR302A/CARTPT/NPY  2R/PNLIP/GHSR/OR56A5/SLCO1B  1/SCP2D1/MIR128-1/MIR301B |
| --- | --- | --- | --- | --- |

| 0.648367857 16699 | |  | | tags=33%,  list=30%,  signal=23% | |  | | RHBDD1/DAP/CAPN3/DDRGK1/R  NF139/CASP4/RNF144A/GBA/M  BP/RNF19A/PLK1/NRDC/PSME2/  KHDC1/LGMN/PRR7/GPER1/JAK2  /UBE2V2/S100A8/DAPK1/CLEC3  B/ADAM9/RNF19B/PSME4/ZC3H  12A/BAX/TBC1D10A/STAT3/CE  BPA/CCN1/NKD2/TREM2/CTSC/  S100A9/MDM2/CASP8/HIP1/CTS  H/BCL2L11/TRIB1/IFI16/KLKB1/F  3/FAS/LYN/PLK3/PCOLCE/EFNA  3/SFRP2/KLHL40/SYK/CR1/HPN  /NGF/ECSCR/LGALS9/F2R/SPON  1/MAPT/NLRP3/CCK/CARD9/CO  L4A3/KCNE2/BLID/FN1/ADAM8/  VSIR/CYFIP2/NGFR/NLRC4/USP5  0/MAPK12/KHDC1L/TNFRSF1B/A  DRA2A/RGN/SH3RF2/BCL2L10/E  PHA4/TNFSF10/CBFA2T3/TNFRSF  10A/CASP1/BAG2/CASP10/CLE  C7A/CCBE1/CLDN4/FOXL2/CAV  3/F12/CASP12/MEFV/PYHIN1/T  RIM67/IL1B/CLDN3/TNFSF15/P2R  X1/EGF/CNTN2/CASP14/TNF/L  CK/TTBK1/GRIN1/GRIN2A/AIM2  /CASP5/SEMG2/ST18/FASLG/IF  NG/SEMG1 | |
| --- | --- | --- | --- | --- | --- | --- | --- | --- | --- |
| 0.648367857 9094 |  | | tags=58%,  list=16%,  signal=48% | |  | | CACNG5/CPT1C/SHISA9/GRIA1/  SHISA8/CACNG4/SHISA6/CACN  G3/GRIA4/CACNG7/GRIA3/OLF  M3/VWC2L/GRIA2/CACNG2 | |  |

| 0.648367857 11198 | |  | | tags=33%,  list=20%,  signal=27% | |  | | NCMAP/NTSR1/CCK/HGF/SHANK  1/TNFRSF1B/PTAFR/ITGAX/RELN  /ZNF488/ITGA2/TENM4/CST7/N  ETO1/NRXN1/CUX2/GRIN1/TNR/  RIMS2/RIMS1/CARTPT | | |
| --- | --- | --- | --- | --- | --- | --- | --- | --- | --- | --- |
| 0.648367857 12415 | |  | | tags=65%,  list=22%,  signal=51% | |  | | JAK3/IL1RL1/ANXA1/CD80/IL27  RA/TNFSF4/HAVCR2/CCL19/IL1  8/IL1B/IL27/CCR2/IL23R/IL12RB  1/XCL1/IL12B/NLRP10 | |  |
| 0.648367857 9827 |  | | tags=21%,  list=17%,  signal=17% | |  | | IL27RA/TNFSF4/FOXP3/CD28/IL  10/CD40LG/BATF/PTPRC/TBX21  /AICDA/BCL11B/CCR6/IL2 | |  | |

| 0.648367857 10571 |  | tags=20%,  list=19%,  signal=16% |  | SHANK1/C3/IL6/MSR1/HRC/PLC  D4/DYSF/SLC30A8/BCL3/DRD2/  BDKRB1/CD19/JPH1/SLC18A2/F2  RL3/LCN2/SMIM22/APLNR/SLC1  7A7/CASQ1/ATP1A2/CORO1A/  TRPM2/PTPN6/CCL3/CCL19/SOR  L1/JSRP1/SLC8A1/FFAR2/IL13/I  L10/ITPR2/CLIC2/IL1B/PLCH2/C  CL21/ITGB3/P2RY6/PLCH1/HTR2  A/PTPRC/CCR7/HAP1/CCR5/PLN  /CXCL10/B4GALNT1/CXCL11/H  TR2B/NOS1/TNF/CRP/LCK/XCL1  /CXCL9/RYR2/TRDN/S100A7/D  RD1/RIT2/FASLG/VSTM2A/XCR1  /FTHL17/HTR2C/SUN5 |
| --- | --- | --- | --- | --- |

| 0.648367857 13238 |  | tags=39%,  list=23%,  signal=30% |  | ANK2/KIT/TNNI3K/TNNC2/CALC  RL/TNNI2/CACNA1C/NKX2-  5/PPP1R12B/TRPM4/STC1/MYBP  H/CASQ2/MYOCD/CHGA/F2R/M  YBPC3/KCNMA1/ADORA1/TNNC  1/SCN4A/GUCY1A1/HRC/MYLK2  /EDN2/NOS1AP/ADRA2B/ADRA2  A/PTAFR/STRIT1/OXTR/SPHK1/  CHRNA3/CASQ1/ATP1A2/ADRA  1B/PTGS2/SLC8A1/DOCK4/CAV  3/CHRM3/CLIC2/NPNT/CALCA/  HCN4/ITGA2/TNNT2/SSTR2/PIK3  CG/NMUR2/GJA5/TACR1/ADRA  1A/P2RX1/PLN/NOS1/RYR2/KCN  B2/TACR3/SLC8A3/KCNA1/CHR  M2/NPY2R/GHSR/MIR153-1 |
| --- | --- | --- | --- | --- |

| 0.648367857 14047 |  | tags=59%,  list=25%,  signal=44% |  | FGG/KCNMB2/EGFR/GJA1/TRPM  4/ECE1/AGT/HRH1/F2R/KCNMB4  /AVPR1A/HRH2/EDN2/ADRA2B/  ADRA2A/PTAFR/FGB/OXTR/ACE  /BDKRB2/ATP1A2/ADRA1B/PTG  S2/CASR/DOCK4/CHRM3/GJA5/  HTR2A/SMTNL1/ADRA1A/P2RX1  /CD38/ASIC2/FGA |
| --- | --- | --- | --- | --- |

| 0.648367857 12425 |  | tags=27%,  list=22%,  signal=21% |  | PELI1/CHI3L1/IRAK3/ANXA1/PR  KCA/CCL18/LGALS9/CCL14/CC  L20/PCK1/CCL13/IL1A/IL6/IL1R  N/SLC30A8/EPO/KMO/CCL7/TN  FRSF11A/CCL8/LCN2/FGB/ADA  MTS12/IL1RL2/SELE/CCL3/CCL1  9/CCL23/CCL11/MIR766/PSMB9  /CCL5/CCL3L3/CCL16/IL1B/NO  D2/CCL21/CCL25/GBP3/GBP2/C  CL24/IL1R2/CCL26/CCL4/CD38/  ABCC2/ACOD1/GBP1/XCL1/NLR  P7/CCL22/XCL2/ST18/CCL1/MI  R204 |
| --- | --- | --- | --- | --- |
| 0.648367857 11125 |  | tags=34%,  list=20%,  signal=28% |  | NTSR1/CCK/ADORA1/AVPR1A/G  RM2/NPY5R/KMO/SLC17A7/ATP  1A2/TRH/SLC6A1/SLC7A11/SLC  1A1/KCNJ10/SLC1A7/SYT1/SYT  4/SLC1A2/GRM7/PPFIA2/RIMS1  /SLC17A8/SLC17A6 |

| 0.648367857 9973 |  | | tags=29%,  list=18%,  signal=24% | |  | | DDAH1/GRIN3A/OTC/CASR/GLR  B/SLC1A1/TDO2/GLDC/GLRA2/  NOS1/DDC/GRIN1/GRM7/GAD1/  DPYS/GLRA3/GLRA1 | |  |
| --- | --- | --- | --- | --- | --- | --- | --- | --- | --- |
| 0.648367857 12509 | |  | | tags=29%,  list=22%,  signal=22% | |  | | NRAP/HAMP/PDGFRB/GREM1/TB  XT/AGT/JAG1/WT1/MYOCD/T  BX5/EFNB2/FZD7/PI16/DKK1/TG  FB2/ALPK3/MYH11/SOX17/BMP  7/VCAM1/NRG1/SLC8A1/TWIST  1/CAV3/MIR199A1/FOLR1/IGF1  /MIR23A/EOMES/TENM4/NEBL/  ADRA1A/GATA6/ALPK2/ISL1/A  CTC1/MYOM3/AGTR2/CSRP3/N  KX2-  6/MIR204/MIR199B/MIR200B/MIR  208A | |
| 0.648367857 9718 |  | | tags=32%,  list=17%,  signal=27% | |  | | PENK/GABRA5/DPP4/LYPD1/ADC  YAP1/BRINP1/ADRB1/CRH/DRD1  /NEUROD2/MORC1/NPY2R/HTR2  C | | |

| 0.648367857 16554 |  | tags=47%,  list=29%,  signal=34% |  | MTDH/LTF/BMP6/TICAM1/IRAK2  /NOS3/CX3CL1/CD14/TGFB1/T  RIB1/LYN/LY96/PRKCA/LY86/H  CK/PTAFR/CARD16/CD180/CCL3  /SCIMP/CCL5/IL18/CD6/IL1B/A  COD1/LBP/TNF/PTPN22 |
| --- | --- | --- | --- | --- |
| 0.648367857 14812 |  | tags=50%,  list=26%,  signal=37% |  | LRRC32/PDE5A/IL20RB/GLMN/TY  ROBP/PAWR/MAD1L1/CD274/CD  74/BTK/DTX1/HLA-  G/LGALS3/RUNX3/LYN/NRARP/  LGALS9B/FOXJ1/PELI1/JAK3/IN  HBA/TNFRSF21/FGR/ARG2/LOXL  3/ANXA1/LGALS9/VSIG4/PDCD  1LG2/CD80/HLA-  F/PLA2G2D/VSIR/MNDA/IFNB1/  TNFSF4/INPP5D/PLA2G2E/LST1/  HAVCR2/RUNX1/TNFAIP8L2/CD8  6/PTPN6/PGLYRP3/PLA2G2A/FO  XP3/SOX11/LILRB2/CD300A/IL1  0/FGL2/IFNL1/CLEC4G/IL2RA/T  BC1D10C/LILRB1/IRF1/SAMSN1/  SHH/SPN/TBX21/BANK1/XCL1/P  TPN22/SLA2/LAG3/PGLYRP2/ID  O1/CLNK/TNFRSF13B/LAX1/TIGI  T/CTLA4/IL2 |

| 0.648367857 11891 |  | tags=46%,  list=21%,  signal=36% |  | DLX2/RPE65/THY1/CDHR1/PAX6  /RP1L1/GNAT1/FSCN2/RORB/PD  E6C/THRB/TULP1/PRDM1/USH1C  /PROM1/CNTF/SDK2/GNGT1/CR  B1/PCARE/DSCAM/CNGB1/MFRP  /NR2E3/CABP4/RP1/OLFM3/GN  AT2 |  | |
| --- | --- | --- | --- | --- | --- | --- |
| 0.648367857 11042 |  | tags=50%,  list=20%,  signal=40% |  | RHO/GNAT1/GUCA1A/PDE6C/AI  PL1/GUCY2D/SAG/PDE6A/ABCA  4/GNGT1/CNGA1/GRK1/CNGB1/  GUCA1C/GRK7/RP1/GUCY2F | |  |
| 0.648367857 11826 |  | tags=32%,  list=21%,  signal=25% |  | NGF/FOXC1/MYOCD/HMGA2/PA  X6/KLF4/MMP9/TWIST1/PYHIN1  /IGF1/ITGA2/POU4F1/GATA3/N  EUROD1/EGF/ISL1/MMP8/IFNG/P  OU4F2 | | |

| 0.648367857 11660 |  | tags=45%,  list=21%,  signal=36% |  | THY1/CEMIP/F2R/NTSR1/BDKRB1  /CD19/F2RL3/APLNR/CASQ1/IL  13/P2RY6/HAP1/CXCL10/CXCL1  1/XCL1/CXCL9/TRDN/DRD1 |
| --- | --- | --- | --- | --- |
| 0.648367857 14395 |  | tags=54%,  list=25%,  signal=40% |  | TYROBP/PAWR/CD74/BTK/CLCF  1/LYN/BST1/PELI1/CDKN1A/TN  FRSF21/AHR/GPR183/MNDA/IL7  /INPP5D/NCKAP1L/SASH3/CD30  0A/TNFRSF4/IL13/IL10/NFATC2  /TNFSF13B/PTPRC/CARD11/CD3  8/MZB1/IKZF3/TNFRSF13B/CTLA  4/IL21/FCRL3/IL2 |
| 0.648367857 12415 |  | tags=50%,  list=22%,  signal=39% |  | JAK3/LOXL3/ANXA1/NLRP3/CD  80/TNFSF4/CD86/CCL19/FOXP3  /IL18/IL27/MYB/IL23R/IL12RB1/  TBX21/IL12B/IL2 |

| 0.648367857 11889 |  | tags=43%,  list=21%,  signal=34% |  | SLC1A3/ASIC1/SLC24A4/SLC6A  12/SCNN1G/ATP1A3/GRIK5/SLC  4A4/SLC18A1/SCN4A/SLC6A9/  HCN2/TRPM5/SLC6A13/SLC18A2  /SLC6A20/SCN1B/PKD2L1/SCN4  B/SLC17A7/ATP1A2/SCN2B/SLC  6A3/TRPM2/MFSD2A/KCNK1/SC  N9A/ATP12A/SCNN1B/SCNN1A/  SLC8A1/SLC5A4/SLC6A1/SLC34  A2/SLC9C2/SLC6A2/SLC1A1/H  CN4/SLC5A12/SLC9A7/SLC34A1  /SLC28A3/SCN7A/SLC12A3/SL  C9A2/SLC13A5/SLC4A8/GRIK1/  SLC5A7/SLC5A8/ABCB11/FXYD2  /SLC1A2/SLC24A2/CACNA1G/S  LC6A18/ASIC2/CACNA1I/SCN1A  /SCN2A/SLC8A3/SLC13A2/SLC  4A10/SCN3A/ASIC5/SLC17A3 | |
| --- | --- | --- | --- | --- | --- |
| 0.648367857 12567 |  | tags=42%,  list=22%,  signal=33% |  | FOLR3/SLC46A1/CUBN/SLC2A6/  AMN/STRA6/LRP2/SLC35F3/RTB  DN/FOLR1/SLC52A1/SLC2A14/G  C/TCN1/SLC2A2/AFM/C2orf83 |  |

| 0.648367857 11549 | |  | | tags=42%,  list=20%,  signal=34% | |  | | CRHBP/ALK/SNCG/PBX3/NTSR1/  ZFHX2/SHANK1/HOMER1/PREX2/  DMRT3/UCHL1/FGF12/DRD2/ZIC  1/GBX1/GRIN2D/SLITRK5/ADAM  22/EPHA4/DAB1/CHRNA3/ATP1  A2/TRH/NRXN2/NLGN4X/PPP1R1  B/PCDH17/ADAM2/SLC7A11/GL  RB/KLHL1/ASIP/CEND1/KCNJ10/  SEZ6/HTR2A/NPY/EN1/NRXN3/P  AX5/CNTN2/CHRNA4/NRXN1/OP  RD1/SLC1A2/SLITRK1/DMBX1/D  RD3/GRIN1/OTOG/SLITRK6/CNT  NAP2/DRD1/SCN1A/OPRM1/GAB  RG2/CARTPT/GLRA1/GHSR/OR56  A5 | | |
| --- | --- | --- | --- | --- | --- | --- | --- | --- | --- | --- |
| 0.648367857 11769 | |  | | tags=27%,  list=21%,  signal=21% | |  | | ABCA7/SPON1/UNC13A/MGAT3  /EPHA4/ACE/SORL1/IGF1/NAT8  B/HAP1/TNF/LRRTM3/IFNG | |  |
| 0.648367857 2916 |  | | tags=54%,  list=5%,  signal=51% | |  | | CDADC1/NT5M/GUK1/TYMS/SH  MT1/TK1/AL353753.1 | |  | |

| 0.648367857 14097 |  | tags=52%,  list=25%,  signal=39% |  | LAMB1/COL8A1/ITGB2/LAMA3/I  NHBA/MMP2/COL4A2/HMGA2/T  BX20/ITGA7/FN1/COL5A1/NOG  /ITGA5/DKK1/SOX17/SOX2/VT  N/COL12A1/MMP9/EOMES/LAM  B3/GATA6/ITGA4/LHX1/MMP8 |
| --- | --- | --- | --- | --- |
| 0.648367857 13922 |  | tags=38%,  list=25%,  signal=28% |  | DIO3/COL8A1/NECTIN1/NECTIN3  /ROM1/MYO7A/CRYGB/MAN2A  1/JAG1/RPE65/THY1/FJX1/GDF  11/PAX6/COL5A1/GNAT1/FOXN  4/WNT5A/FSCN2/WNT2/STRA6  /RORB/WNT9A/PDE6C/THRB/BM  P7/TULP1/SOX11/ZEB1/LCTL/T  WIST1/FOXL2/PRDM1/USH1C/PR  OM1/MEGF11/VSX1/PITX3/CNT  F/EPHB2/SDK2/PAX2/ATP8A2/C  ALB1/GNGT1/CRB1/DSCAM/MFR  P/NR2E3/CABP4/RP1/TFAP2B/O  LFM3/LHX1/FASLG/GNAT2 |

| 0.648367857 9103 |  | | tags=54%,  list=16%,  signal=45% | |  | | TNFSF4/CD160/FGL2/IFNL1/IL23  R/IL12RB1/IL12B | |  |
| --- | --- | --- | --- | --- | --- | --- | --- | --- | --- |
| 0.648367857 12770 | |  | | tags=42%,  list=23%,  signal=32% | |  | | SLC39A5/ATP1B2/SLC9A3/TRPM  4/SLC46A1/AGT/SLC1A3/SLC39  A8/ATP1A3/CACNA2D1/KCNJ1/  NTSR1/KCNE2/THBS1/HCN2/SLC  7A3/KCNJ8/ATP1A2/ABCC9/TR  PM2/ATP12A/SLC8A1/LRP2/FA  M155A/SLC7A11/SLC39A12/SLC  9C2/KCNH2/SLC1A1/KCNK9/FOL  R1/HCN4/KCNJ10/KCNJ11/SLC9  A7/TRPV6/SLC34A1/KCNJ6/SLC  12A3/SLC9A2/KCNJ3/KCNJ4/FX  YD2/SLC1A2/CALCR/KCNJ9/KC  NJ16/CNGA3/SLC8A3/IFNG | |
| 0.648367857 7183 |  | | tags=36%,  list=13%,  signal=31% | |  | | C3/SERPINC1/NPY5R/CD28/CD6  /IL10/IL2RA/CYSLTR1/ADCYAP1  /CCR7/GATA3/NLRP6/TNF/RAS  GRP1/LTA/IL12B/IL31RA/OPRM1 | | |

| 0.648367857 10571 |  | tags=35%,  list=19%,  signal=28% |  | SHANK1/AVPR1A/OXTR/NLGN4Y  /GRID1/NRXN2/NLGN4X/PTCHD1  /GRP/EN1/GNG8/NRXN3/BRINP1  /NRXN1/DRD3/GRIN1/CNTNAP2/  GAD1 |
| --- | --- | --- | --- | --- |
| 0.648367857 13511 |  | tags=44%,  list=24%,  signal=34% |  | P2RX4/ANK2/CACNB2/CACNA1C  /TRPM4/CACNA1H/KDR/CACNB4  /CACNA2D1/MIR26A2/NTSR1/C  CK/SCN4A/HCN2/FGF12/SCN1B  /SCN4B/ATP1A2/SCN2B/SCN9A  /CACNA1E/SLC8A1/CAV3/KCN  H2/HCN4/GJA5/SCN7A/MYOC/  CACNG4/CHRNA4/HSH2D/CACN  A1G/CHRNA6/CACNA1I/BEST2/  SCN1A/SCN2A/SCN3A/CACNG2  /MIR208A |

| 0.648367857 11290 |  | tags=38%,  list=20%,  signal=30% |  | CASQ2/KCNAB1/MIR26A2/KCNE  1/KCNE2/DYSF/EPO/FGF12/DRD  2/REM1/ADRA2A/TCAF2/MIR30  C1/MIR212/ATP1A2/CRHR1/KCN  E1B/RRAD/MMP9/TWIST1/GEM/  CAV3/HECW2/CLIC2/EPHB2/HEC  W1/ANO9/PLN/MRLN/APOA2/T  RDN/DRD3/OR56A5/MIR153-1 | |
| --- | --- | --- | --- | --- | --- |
| 0.648367857 697 |  | tags=23%,  list=1%,  signal=23% MIR503/MIR218-1/MIR182 | | |  |

| 0.648367857 11891 |  | tags=43%,  list=21%,  signal=34% |  | DLX2/RPE65/THY1/FJX1/FOXN4  /TGFB2/RORB/PDE6C/SLC17A7/  THRB/GPM6A/DLL4/USH1C/PRO  M1/MEGF11/VSX1/SDK2/ATP8A  2/CALB1/NEUROD1/DSCAM/NEU  ROD4/CABP4/RP1/TFAP2B/LHX1  /GNAT2/SLC17A8/POU4F2 |
| --- | --- | --- | --- | --- |
| 0.648367857 13668 |  | tags=46%,  list=24%,  signal=35% |  | ABCB1/FABP3/P2RX4/ERFE/ABC  B4/AGT/ABCA7/PLA2R1/NTSR1  /CCK/AVPR1A/KMO/TNFRSF11A  /PTAFR/TRH/SLC6A1/SLC38A3/  IL1B/SLC34A1/ATP8A2/SYT4/C  YP4A11/TNFSF11 |

| 0.648367857 12358 |  | tags=39%,  list=22%,  signal=30% |  | ELANE/SYK/LGALS9/F2R/WNT5  A/CD244/SERPINE1/AFAP1L2/TL  R7/CLEC7A/FFAR2/IL1B/NOD2/  TLR8/CD2/LBP/TNF/APOA2/NLR  P10 | |
| --- | --- | --- | --- | --- | --- |
| 0.648367857 12616 |  | tags=41%,  list=22%,  signal=32% |  | HRG/SYK/PRKCA/ADAM8/CA2/  TNFRSF11A/VDR/IL18/MIR199A1  /IL15/DCSTAMP/TNFSF11/IL12B  /MC4R/IL21/IL2 |  |
| 0.648367857 12401 |  | tags=31%,  list=22%,  signal=24% |  | TRPM4/KDR/MIR26A2/NTSR1/CC  K/FGF12/SCN1B/SCN2B/CAV3/  HCN4/GJA5/MYOC/HSH2D/CAC  NA1G/MIR208A | |

| 0.648367857 14583 |  | tags=46%,  list=26%,  signal=34% |  | CAMK2B/SHISA7/EGR2/SYP/DLG  4/KIT/NEURL1/AGT/UNC13A/C  AMK2A/DRD2/ARC/SHISA9/KCN  J10/SHISA8/EPHB2/SHISA6/SYT  4/RASGRF1/NETO1/CNTN2/GRIN  1/SLC4A10/GRM5 | |
| --- | --- | --- | --- | --- | --- |
| 0.648367857 12096 |  | tags=38%,  list=21%,  signal=30% |  | LGALS3/CACNA1F/THY1/KCNN4  /LILRB4/PTPN6/CD300A/CD160/  CCR7/GBP1/LCK/PTPN22/UBASH  3A/TESPA1/TRAT1 | |
| 0.648367857 11813 |  | tags=31%,  list=21%,  signal=24% |  | FOXC1/MSX1/HMGA2/PAX6/KL  F4/ZBTB7C/SOX11/TWIST1/IGF  1/POU4F1/LIF/GATA3/NEUROD1  /ANKRD33/IFNG/POU4F2 |  |

| 0.648367857 11741 |  | tags=31%,  list=21%,  signal=24% |  | CASQ2/SYT12/AANAT/CRHBP/K  CNMA1/KCNMB4/HOMER1/THBS1  /WNT5A/PENK/GUCA1A/FGB/C  PNE4/KCNMB1/SYT11/TRPM2/S  YT10/SUCNR1/CASR/ALOX15/S  LC6A1/RASGRP2/LCE1D/HPCA/T  RPV6/TNNT2/ADCY8/SYT1/ADG  RV1/CPNE5/SYT5/ADCY1/AQP3  /SYT4/SYT6/SYT9/SYT2/TRPC3  /RYR2/CPNE6/DPEP1/NEUROD2/  CACNG2/FGA/AMELX |
| --- | --- | --- | --- | --- |

| 0.648367857 14185 |  | tags=56%,  list=25%,  signal=42% |  | STAC3/SCN5A/AKAP6/TGFB3/A  NK2/CACNB2/CACNA1C/STBD1/  CACNA2D1/CACNG1/DYSF/NOS  1AP/AHNAK2/SCN1B/CASQ1/A  TP1A2/STAC/SLC8A1/CAV3/KC  NJ11/ADRA1A/CACNG4/KCNJ3/  NOS1/CACNA1S/CACNG6/CACN  G7/SCN1A/SCN2A |
| --- | --- | --- | --- | --- |
| 0.648367857 12288 |  | tags=38%,  list=22%,  signal=30% |  | MEIS2/HRH1/CCK/HRH2/DRD2/P  DE1B/LRRN4/ATP1A2/PPP1R1B/N  DRG4/ADAM2/SLC7A11/HOXA1  /NETO1/SLC1A2/FOXB1/DRD3/  GRIN1/GRIN2A/DRD1 |

| 0.648367857 9256 |  | tags=27%,  list=16%,  signal=23% |  | MYOD1/MYF6/ZEB1/TWIST1/AS  CL2/ARNTL2/GATA3/NEUROD1/  NEUROG2/SCRT2/NEUROD2/NEUR  OG1/MYF5/MYOG |
| --- | --- | --- | --- | --- |
| 0.648367857 6826 |  | tags=51%,  list=12%,  signal=45% |  | HRH1/HTR7/OR11H7/HRH2/HRH4  /HTR4/GRM1/CHRM3/GABBR2/H  TR1D/HTR2A/HTR1B/HTR2B/ADR  B1/HTR1F/OPRM1/HTR5A/CHRM  2/HTR1E/OR5T2/HTR2C |

| 0.648367857 14684 |  | tags=57%,  list=26%,  signal=42% |  | CCL17/S100A14/S100A9/RARRE  S2/IL1F10/MCOLN2/THBS4/DAP  K2/CD74/CSF1/ITGB2/FCER1G/L  GALS3/PREX1/BST1/TRPV4/PEC  AM1/SYK/ANXA1/CCL18/C3AR  1/CCL14/HRH1/CCL20/ITGA1/R  AC2/CD177/CMKLR1/C5AR2/CC  L13/SLIT2/IL1A/ADAM8/THBS1  /DYSF/SCG2/IL1RN/CSF3R/EDN  2/TGFB2/CCL7/CCL8/JAML/CC  L3/CCL19/CCL23/CCL11/NCKAP  1L/VAV1/CCL5/CAMK1D/CD300  A/MSTN/SLAMF8/CCL3L3/CXCL  17/CCL16/PTGER4/IL1B/PIK3CG  /CCL21/UMOD/CCL25/CCL24/C  CR7/CCL26/CCL4/CXCL10/LBP/  CXCL11/IL37/XCL1/CXCL9/CCL  22/XCL2/S100A7/IL36G/CXCL1  3/CCL1 |
| --- | --- | --- | --- | --- |

| 0.648367857 15197 |  | tags=44%,  list=27%,  signal=32% |  | BAX/CX3CL1/STAT3/COL3A1/C  TNNA2/NEXMIF/CAMK2B/GAS6/  TRIM46/DCLK1/FGF13/NRG3/CX  CR4/AUTS2/FGFR1/SRGAP2/SEM  A3A/NDNF/GPR173/CXCL12/LRP  12/NRP1/ADGRG1/NKX2-  1/TUBB2B/ULK4/SPOCK1/CCK/P  AX6/TBX20/CAMK2A/SRGAP2C  /DRD2/CDK5R2/KIRREL3/DAB1/  ARX/MAP1B/MDGA1/NRG1/TWI  ST1/CCKAR/ASTN1/GPM6A/REL  N/SCRT1/ATOH1/KIAA0319/NTN  1/PHOX2B/BARHL2/BARHL1/NTR  K3/SOX14/FOXG1/GATA3/FEZF  1/CNTN2/ADGRL3/NEUROD4/TL  X3/CCR4/VAX1/FEZF2/DRGX/S  CRT2/UNC5D/DRD1/LHX1/OLIG3 |
| --- | --- | --- | --- | --- |

| 0.648367857 10562 |  | tags=49%,  list=19%,  signal=40% |  | SLC1A3/SLC6A12/SLC18A1/SLC  6A9/GABRQ/SLC6A13/AQP1/SL  C18A2/SLC6A20/SLC17A7/SLC6  A3/SLC38A5/SLC6A1/SLC7A11  /SLC6A2/SLC1A1/SLC1A7/SLC3  6A2/SLC5A7/SLC1A2/SLC6A18  /SLC32A1/SLC17A8/SLC17A6/C  PLX3 |
| --- | --- | --- | --- | --- |
| 0.648367857 12166 |  | tags=47%,  list=22%,  signal=37% |  | SYK/LGALS9/IRF5/TNFSF4/CCL1  9/CLEC7A/SCIMP/LTB/IRF8/CD4  0LG/IRF1/CCR7/IL23R/ISL1/IL12  B/IDO1/IFNG |
| 0.648367857 10027 |  | tags=32%,  list=18%,  signal=26% |  | KLF4/DDAH1/NOS1AP/PTGS2/CL  EC7A/AGXT2/IL1B/TNF/AGTR2/  KLRC4-  KLRK1/KLRK1/MMP8/IFNG/MIR99  B |

| 0.648367857 16660 | |  | | tags=47%,  list=29%,  signal=33% | |  | | HDAC4/HAND2/UCMA/FAM20C/  LTF/BMP6/CEBPB/LIMD1/TP63/G  LI3/SEMA4D/BMP2/CEBPA/CCN1  /TMEM64/CITED1/ID1/ID3/ZHX3  /TCIRG1/CEBPD/TWIST2/HEMGN  /SFRP2/TRPM4/GREM1/JAG1/R  ASSF2/IFITM1/HGF/NOCT/CHRD  /GLI1/IL6/NOG/FGFR2/RORB/B  MP7/RUNX2/WNT10B/GDPD2/T  MEM119/SOX11/NPPC/TWIST1/  BMPR1B/NPNT/IGF1/AREG/FGF23  /TNF/GDF10/NELL1/MIR138-  2/MIR208A |
| --- | --- | --- | --- | --- | --- | --- | --- | --- |
| 0.648367857 8401 |  | | tags=40%,  list=15%,  signal=34% | |  | | SH2D3C/SH2B3/GRAP/LAT/SH2D  2A/CNTNAP1/ARHGAP6/CHN1/B  LNK/CD28/SLA/SOCS2/CHN2/S  KAP1/GRAP2/STAP1/HSH2D/SLA  2/CLNK/SH2D1A/FCRL2 | |

| 0.648367857 7881 |  | | tags=21%,  list=14%,  signal=18% | |  | | FAIM2/GLI2/KLHL1/CEND1/SEZ6  /GRID2/ZNF365/NEUROD2/AGTR  2/LHX1 | |  |
| --- | --- | --- | --- | --- | --- | --- | --- | --- | --- |
| 0.648367857 12358 | |  | | tags=46%,  list=22%,  signal=36% | |  | | ELANE/IL1RL1/GREM1/NRP1/CX3  CR1/CSF1R/CRLF2/CCR10/CCR1  /IL20RA/CD93/CHRD/THBS1/IL1  RN/CSF3R/NOG/PRLR/IL27RA/T  NFRSF1B/TNFRSF11A/CD109/CC  RL2/GDF5/LEPR/PXDN/IL10RA/IL  18BP/EBI3/CSF2RA/IL2RG/CCR9  /IL2RA/ITGB3/IL2RB/CCR7/WFI  KKN2/IL1R2/ACKR1/CCR2/CCR5  /IL23R/GREM2/IL22RA1/IL12RB1  /CCR3/GBP1/ITGA4/CXCR3/NLR  P7/CCR4/CXCR6/IL12B/IL31RA/  CCR6/XCR1/CXCR5/IL22RA2/CC  R8/IL17F | |

| 0.648367857 16569 |  | tags=52%,  list=29%,  signal=37% |  | FAM20C/LTF/BMP6/CEBPB/ATP2  B1/BMP2K/PTN/OMD/NOS3/BMP  2/HEY2/HEY1/ODAPH/ANO6/CC  N1/GAS6/IFITM5/TGFB3/TGFB1/  COMP/KL/S1PR1/ISG15/TRPM4/  GREM1/ASPN/AHSG/CCR1/ENA  M/BMP7/WNT10B/CCL3/TMEM1  19/SLC8A1/TWIST1/BMPR1B/EC  M1/AMTN/PHOSPHO1/MMP20/A  DGRV1/FGF23/OSR2/NELL1/DMP  1/STATH/AMELX/MIR208A |
| --- | --- | --- | --- | --- |
| 0.648367857 13353 |  | tags=39%,  list=24%,  signal=30% |  | ATP2A3/ANK2/TNNI3K/ATP1B2/  NKX2-  5/TRPM4/AGT/CASQ2/NPR1/AT  P1A3/HRC/CORIN/CASQ1/ATP1  A2/ABCC9/FXYD7/NPPC/SLC8A  1/ITPR2/KCNJ11/GJA5/ATP2B2/  NOS1/FXYD2/RYR2/TRDN/SLC8  A3/MIR208A |

| 0.648367857 11187 |  | tags=45%,  list=20%,  signal=36% |  | CD1D/PIK3R6/NCR1/HLA-  F/FCER2/CD1C/RIPK3/HAVCR2/  CD1E/CD1A/PGLYRP3/IL18RAP/  CLEC7A/VAV1/KRT6A/IL7R/IL13  /CD160/CADM1/CR1L/PRF1/CD  1B/LILRB1/PTPRC/IL23R/IL12RB1  /NCR3/KIR2DL4/STAP1/XCL1/S  LAMF6/RASGRP1/LAG3/IL12B/P  GLYRP2/KLRC4-  KLRK1/CRTAM/SH2D1A/KLRK1/  CD5L/IL21/IFNG |
| --- | --- | --- | --- | --- |

| 0.648367857 15541 |  | tags=43%,  list=27%,  signal=31% |  | ARNT2/EZH2/GH1/CCDC62/STA  T3/RAMP3/GGT5/KIF18A/CASP8  /GJB2/F7/BCL2L11/DHH/ASS1/  EGFR/TGFB1/CD4/PAM/GJA1/C  OL1A1/PDGFRB/IGFBP2/CYP1A2  /AGT/ANXA1/CRHBP/RGS9/NQ  O1/PENK/PTGFR/MYOD1/OXTR/  BMP7/MAP1B/SSTR1/GGT2/PTG  S2/SLC6A1/GGT1/ESR1/IL10/S  OCS2/KCNJ11/ITGA2/SLC34A1/  SSTR2/GGT3P/POU4F1/ADCYAP1  R1/AREG/CD38/FGF10/TACR3/H  TR5A/SSTR3/POU4F2/GHSR/MY  OG |
| --- | --- | --- | --- | --- |

| 0.648367857 11908 | |  | | tags=47%,  list=21%,  signal=37% | |  | | PRKCA/PTGER3/ADGRG1/ADGRG  3/MC1R/UCN3/ADGRB1/CHGA/  ADGRL2/ADCY4/PTGER1/DRD2/  ADGRB3/APLP1/ADRA2B/ADRA2  A/GCGR/PTGFR/MRAP/ADGRE3/  PTGIR/ADRA1B/CRHR1/TSHR/AD  GRG6/UCN2/ADM2/ADGRB2/FSH  R/GALR1/CALCA/PTGER4/PTHLH  /GPR65/GPHA2/S1PR4/ADCY8/  ADCYAP1/ADRA1A/ADRB3/ADC  Y1/PLN/CXCL10/MC5R/ADGRE1  /ADGRL3/CXCL11/ADCY2/DRD5  /NOS1/LGR5/ADGRG4/ADRB1/C  XCL9/ADGRG5/DRD3/CALCR/M  C4R/DRD1/OPRM1/RIT2/CALCB/  OR56A5/MC3R |
| --- | --- | --- | --- | --- | --- | --- | --- | --- |
| 0.648367857 7796 |  | | tags=16%,  list=14%,  signal=13% | |  | | NRG1/SLC8A1/CAV3/MIR199A1  /IGF1/MIR23A/NEBL/ADRA1A/G  ATA6/ALPK2/ACTC1/MYOM3/A  GTR2/CSRP3/NKX2-  6/MIR204/MIR199B/MIR200B/MIR  208A | |

| 0.648367857 10341  0.648367857 1786 |  | GNAT1/RRH/GUCA1A/AQP1/RGR  /NFATC4/CARD16/AIPL1/PTGS2  /GUCY2D/GPR88/OPN1LW/SAG  tags=20%, /PDE6A/GNGT1/OPN4/CNGA1/C  list=18%, RIP1/GRK1/CNGB1/GUCA1C/GRK  signal=17% 7/RP1/OPN5/GUCY2F/OPN1MW  tags=5%,  list=3%,  signal=4% EXOC1L | | |
| --- | --- | --- | --- | --- |
| 0.648367857 11290 |  | tags=34%,  list=20%,  signal=27% |  | CASQ2/KCNAB1/MIR26A2/NTSR  1/KCNE1/KCNE2/THBS1/DYSF/E  PO/FGF12/DRD2/REM1/ADRA2A  /TCAF2/MIR212/ATP1A2/CRHR1  /KCNE1B/RRAD/MMP9/TWIST1/  GEM/CAV3/HECW2/KCNH2/CLIC  2/EPHB2/HECW1/ANO9/PLN/MR  LN/TRDN/DRD3/OR56A5/MIR153  -1 |

| 0.648367857 837 |  | | tags=25%,  list=1%,  signal=25% MYOCD/MIR199B/MIR182 | | | |  |
| --- | --- | --- | --- | --- | --- | --- | --- |
| 0.648367857 11801 | |  | | tags=50%,  list=21%,  signal=40% |  | RPE65/THY1/CDHR1/PAX6/RP1L  1/GNAT1/FSCN2/RORB/PDE6C/T  HRB/TULP1/PRDM1/USH1C/GNGT  1/CRB1/PCARE/CNGB1/MFRP/NR  2E3/CABP4/RP1/OLFM3/GNAT2 | |
| 0.648367857 8290 |  | | tags=49%,  list=15%,  signal=42% | |  | TREM2/AKAP6/CDH13/CD4/P2R  X4/SYK/C10orf71/P2RX2/CCL3/  P2RX5/NRG1/IGF1/P2RY6/HAP1/  CCL4/CD3E/TNF/ZAP70/TRDN/N  EUROD2/TRAT1 | |

| 0.648367857 16083 |  | tags=47%,  list=28%,  signal=34% |  | WDR54/GRK4/APLN/ANKRD13B/  CEACAM1/RAMP1/ARRB2/RAMP  3/ARR3/LRP1/CD9/PLCG2/RSPO  1/ANGPT1/DLG4/ITGB2/FCER1G  /CALCRL/MX1/NECAB2/GREM1/  SYK/ACHE/EFNB2/SH3GL2/DRD2  /MX2/SFRP4/DNM3/DKK1/RAB3  1/SELE/GSG1L/ARC/CAV3/MIR1  99A1/HPCA/GRIA1/SAG/ITGB3/  LILRB1/LRRTM1/HTR1B/NTF3/CA  CNG4/CALY/EGF/CNTN2/HTR2B  /CACNG3/LRRTM2/DRD3/CACN  G2 |
| --- | --- | --- | --- | --- |
| 0.648367857 14628 |  | tags=60%,  list=26%,  signal=45% |  | S100A14/RARRES2/THBS4/DAPK  2/CD74/CSF1/BST1/TRPV4/C3A  R1/RAC2/CMKLR1/C5AR2/SLIT2  /THBS1/DYSF/CCL19/NCKAP1L/  CCL5/CAMK1D/MSTN/CXCL17/  CCL21/CCR7/LBP/XCL1/S100A7 |

| 0.648367857 14921  0.648367857 2186 |  | CXCL14/ANO6/S100A14/GAS6/  RARRES2/F7/THBS4/DAPK2/CD7  4/CSF1/PGF/NBL1/LYN/BST1/T  RPV4/CXCL12/PLA2G7/GREM1/  C3AR1/AIF1/LGALS9/CYP19A1/  RAC2/CCR1/CMKLR1/C5AR2/SLI  T2/MMP28/IL6/THBS1/DYSF/W  NT5A/CCN3/EDN2/CCL7/SERPIN  E1/CCL3/CCL19/NCKAP1L/CCL5  /CAMK1D/MSTN/SLAMF8/CXCL  17/CCL21/CCR7/FPR2/CCR2/CC  L4/CXCL10/LBP/TNFSF14/STAP1  /XCL1/XCL2/GPR18/S100A7/KL  tags=55%, RC4-  list=26%, KLRK1/CCR6/CXCL13/CCL1/KLR  signal=40% K1  tags=5%,  list=4%,  signal=5% RBMXL3 |
| --- | --- | --- |

| 0.648367857 12143 |  | tags=51%,  list=21%,  signal=40% |  | AGT/SV2A/SYT12/CHGA/SNCG  /NTSR1/CCK/ADORA1/SLC18A1  /AVPR1A/P2RY1/DRD2/SLC18A2  /NPY5R/KMO/ADRA2B/ADRA2A  /OXTR/CHRNA3/KCNA2/ATP1A2  /SYT11/TRH/SYT10/SLC6A1/R  AB3B/SLC38A3/SLC22A16/HTR2  A/SYT1/HTR1B/SYT5/SYT4/CH  RNA4/SYT6/SYT9/SYT2/FFAR3  /DDC/CRH/DRD3/CHRNA6/DRD1  /GRM7/AGTR2/CARTPT/NPY2R/  GHSR/OR56A5 |
| --- | --- | --- | --- | --- |

| 0.648367857 12415 |  | tags=34%,  list=22%,  signal=27% |  | JAK3/INHBA/SYK/PLCL2/TPD52  /ADGRG3/CD79B/GPR183/LFNG/  DOCK11/BCL3/MFNG/CD19/IL7/  IFNB1/INPP5D/NFAM1/CDH17/DO  CK10/PTPN6/ONECUT1/CD79A/N  CKAP1L/BLNK/VCAM1/IFNW1/IL  11/SLAMF8/IFNE/IL10/CD40LG/  POU2F2/NTRK1/PTPRC/CARD11/  FLT3/CD27/ITGA4/AICDA/IKZF3  /MS4A1/CR2/FCRL3/IFNA10/IFN  A21 |
| --- | --- | --- | --- | --- |

| 0.648367857 9697 |  | tags=36%,  list=17%,  signal=30% |  | MNDA/CD19/IL7/IFNB1/INPP5D/  CD180/CD79A/NCKAP1L/SASH3  /IFNW1/CD300A/TNFRSF4/IL7R/  IL13/IFNE/IL10/NFATC2/CD40LG  /TNFSF13B/PTPRC/CARD11/CD3  8/MZB1/CD70/IKZF3/TNFRSF13B  /MS4A1/CTLA4/CR2/IL21/FCRL  3/IFNA10/IFNA21/IL2 |
| --- | --- | --- | --- | --- |

| 0.648367857 13330 | |  | | tags=49%,  list=24%,  signal=38% | |  | | TCIRG1/RELB/NKX2-  3/RUNX3/LGALS9B/JAK3/ARG2  /LOXL3/ANXA1/LGALS9/GPR18  3/NLRP3/LEF1/CD80/PLA2G2D/R  SAD2/IL6/BCL3/VSIR/PAX1/TNF  SF4/RUNX1/CD86/CCL19/FOXP3  /NCKAP1L/SASH3/IL18/CD160/  PTGER4/BATF/IL27/LY9/MYB/G  ATA3/IL23R/IL12RB1/SPN/TBX2  1/XCL1/SLAMF6/FUT7/IL12B/IF  NG/IL2 | |
| --- | --- | --- | --- | --- | --- | --- | --- | --- | --- |
| 0.648367857 8108 |  | | tags=60%,  list=14%,  signal=51% | |  | | LOXL3/IL6/FOXP3/BATF/LY9/IL  23R/SHH/IL12RB1/SPN/TBX21/S  LAMF6/IL12B | |  |

| 0.648367857 15667 |  | tags=46%,  list=28%,  signal=33% |  | MMP24/P3H1/F2/CYGB/ARRB2/  ADAM15/MMP19/P3H3/MMP7/S  ERPINF2/MIR149/ID1/SERPINB7/T  GFB3/TGFB1/CTSS/COL15A1/MF  AP4/COL1A1/PDGFRB/MMP11/M  MP2/F2R/MMP1/CREB3L1/SCX/  MMP28/COL5A1/IL6/VSIR/ADA  MTS14/P3H2/MRC2/NPPC/MMP9  /ADAMTS2/ITGA2/CIITA/MMP2  0/MYB/MMP25/TMPRSS6/MMP1  6/KLK6/PRSS2/MMP12/MMP13/  MMP8/AMELX/MIR218-1 |
| --- | --- | --- | --- | --- |

| 0.648367857 10174 |  | tags=34%,  list=18%,  signal=28% |  | CD33/WNT5A/NLRC4/USP50/LIL  RA5/MNDA/CARD16/HAVCR2/P  ANX2/CASP1/CCL3/CCL19/CM  A1/SUCNR1/MIR766/CLEC7A/ME  FV/PYHIN1/IL10/IGF1/IL1B/NOD  2/IGHD/TLR8/CCR7/IL1R2/NLRP  7/ISL1/ORM2/GBP5/AIM2/HMGB  4/IFNG/CARD17/GHSR/CARD18/  NLRP10/MIR204/MIR181A1 | |
| --- | --- | --- | --- | --- | --- |
| 0.648367857 11283 |  | tags=26%,  list=20%,  signal=21% |  | DNASE1L2/KRT17/WNT10A/FOX  E1/WNT5A/DKK1/TGFB2/FGFR2  /CD109/HOXC13/WNT10B/TNFR  SF19/DSG4/PTGS2/FST/HPSE/T  RPV3/SOX21/SHH/KRT16/FGF10  /LGR5/TNF/SPINK5/KRT71/KRT8  3/KRT84/KRT33B |  |

| 0.648367857 11161 |  | tags=39%,  list=20%,  signal=31% |  | FZD5/KLK7/CD74/CLCF1/TGFB1  /SEMA7A/HLA-  G/FCER1G/SPON2/NLRP3/HLA-  F/KLK5/RSAD2/IL6/WNT5A/CD  244/TNFSF4/PGC/SCIMP/SASH3  /FFAR2/TNFRSF4/IL13/IL18/CD2  8/CD160/IL1B/NOD2/LILRB1/PTP  RC/GATA3/TBX21/KIR2DL4/FFA  R3/XCL1/MZB1/CLNK/IL2 |
| --- | --- | --- | --- | --- |

| 0.648367857 11604 |  | tags=49%,  list=21%,  signal=39% |  | KCND3/DLG2/KCNN4/KCNAB1/K  CNJ1/GRIK5/KCNMA1/KCNE1/KC  NE2/KCNK6/KCNMB4/HCN2/KCN  F1/NOS1AP/KCNQ3/KCNJ8/KCN  V1/LRRC38/KCNV2/KCNA2/KCN  MB1/ABCC9/KCNK1/KCNE1B/AB  CC8/KCNC1/KCNH2/HCN4/KCNJ  11/KCNJ6/LRRC55/KCNS2/KCNQ  2/GRIK1/KCNJ3/KCNA3/KCNJ4/  LRRC52/KCNB2/KCNG4/CNTNAP2  /KCNA4/KCNJ16/KCND2/KCNC2  /KCNA1/KCNA10 |
| --- | --- | --- | --- | --- |

| 0.648367857 14185 |  | tags=46%,  list=25%,  signal=34% |  | AKAP6/CHD7/CD4/METTL21C/P  2RX4/ANK2/LYN/CACNA1C/CA  SQ2/THY1/CEMIP/F2R/P2RX2/N  TSR1/HRC/EPO/BDKRB1/CD19/J  PH1/F2RL3/APLNR/CASQ1/ATP1  A2/CORO1A/PTPN6/JSRP1/P2RX  5/SLC8A1/IL13/CLIC2/P2RY6/H  AP1/ADCYAP1R1/PLN/CXCL10/  CXCL11/TRPC3/NOS1/XCL1/CX  CL9/RYR2/TRDN/GRIN1/CALCR/  DRD1 |
| --- | --- | --- | --- | --- |

| 0.648367857 14190 |  | tags=30%,  list=25%,  signal=22% |  | ID1/ID3/MDFI/TGFB1/PLAUR/HM  OX1/IFI16/HAND1/NGF/FOXC1/  MSX1/MYOCD/LEF1/HMGA2/PA  X6/BCL3/KLF4/PAX7/ZBTB7C/S  OX11/MMP9/TWIST1/PYHIN1/IG  F1/ITGA2/GZMA/POU4F1/WFIKK  N2/LIF/GATA3/NEUROD1/EGF/I  SL1/ANKRD33/MMP8/IFNG/POU4  F2 | |
| --- | --- | --- | --- | --- | --- |
| 0.648367857 12718 |  | tags=28%,  list=23%,  signal=22% |  | BCL2L11/FGFR1/ACACB/FGF8/N  KX2-  5/GJA1/HAMP/BASP1/WT1/FO  XC1/TBX5/TBX20/PI16/GLI1/TP  73/NOG/WNT2/IL7/FGFR2/FOX  C2/ARX/SASH3/CAV3/MIR199A  1/IGF1/ZFPM2/GATA6/MIR204/  MIR199B/MIR200B/MIR208A |  |

| 0.648367857 12423 |  | tags=41%,  list=22%,  signal=32% |  | SPACA3/FGR/SYK/SIRPB1/C2/A  BCA7/AHSG/C3/DYSF/HCK/SPH  K1/RAB31/SYT11/TULP1/CD300  LF/DOCK2/NCKAP1L/CLEC7A/A  LOX15/CAMK1D/CD300A/IL2RG  /IL1B/ITGA2/IL15RA/IL2RB/PTPR  C/FPR2/IL15/PRKCG/C4B/C4A/  STAP1/TNF/APOA2/SIRPG/IFNG  /MBL2 | |
| --- | --- | --- | --- | --- | --- |
| 0.648367857 13703 |  | tags=35%,  list=24%,  signal=27% |  | TGFB1/FLT1/FABP3/KIT/LYN/PD  GFRB/FGR/TNFAIP8L3/SOCS3/PI  K3R6/KLF4/APOC2/GNB3/CD19/  PIK3R5/PIK3R3/MIR30C1/AGAP2  /CCL19/EPHA8/SOCS2/NOD2/C  CKBR/CCL21/HTR2A/CCR7/FPR2  /FLT3/HTR2B/SLA2/HTR2C/MIR  138-2 |  |

| 0.648367857 10037 |  | tags=40%,  list=18%,  signal=33% |  | WNT5A/ADGRB3/LRRC24/DKK1/  THBS2/SLITRK5/OXTR/GPC6/SY  NDIG1/MDGA1/SLITRK2/ADGRB2  /LHFPL4/GPC4/LRFN5/NTN1/EPH  B2/NTNG2/NTRK1/LRRTM1/SIX4  /NTRK3/LRRN3/AMIGO2/LRTM1/  SLIT1/PTPRD/NRXN1/ADGRL3/C  UX2/EPHA7/SLITRK4/LRRTM2/SL  ITRK1/GRIN1/GRID2/ASIC2/SLIT  RK6/CBLN2/LRTM2/SLITRK3/LRR  TM3/GHSR |
| --- | --- | --- | --- | --- |

| 0.648367857 12002 |  | tags=54%,  list=21%,  signal=42% |  | CXCL12/GPR75/CX3CR1/FOXC1  /CCL18/CCL14/CCL20/CCR10/C  CR1/CMKLR1/CCL13/SLIT2/THP  O/CCL7/CCL8/CCRL2/SLIT3/CC  L3/CCL19/CCL23/CCL11/CCL5/  CCL3L3/CCL16/CCR9/CCL21/CC  L25/CCL24/CCR7/ACKR1/CCL26  /CCR2/CCL4/CCR5/CXCL10/CX  CL11/CCR3/CXCR3/XCL1/CXCL  9/CCR4/CXCR6/CCL22/XCL2/R  EG1A/CCR6/XCR1/CXCL13/CXC  R5/CCL1/CCR8 |
| --- | --- | --- | --- | --- |

| 0.648367857 14198 |  | tags=36%,  list=25%,  signal=27% |  | SLC2A5/AKAP6/ATP1B1/TGFB3/  STAC2/KCNB1/ANK2/CACNB2/C  ACNA1C/STBD1/COL6A3/ANXA  1/KCND3/CACNA2D1/CACNG1/  DYSF/DES/AQP1/CACNB1/NOS1  AP/AHNAK2/SCN1B/CASQ1/AT  P1A2/CDH2/STAC/PGM5/VCAM  1/SLC8A1/CAV3/AQP4/KCNJ11  /SLC27A6/ADRA1A/CACNG4/K  CNJ3/NOS1/SGCG/RYR2/ANK1/  CACNA1S/CACNG6/CACNG7/SC  N1A/OPRM1/SCN2A/SLC8A3/FG  F6 |
| --- | --- | --- | --- | --- |

| 0.648367857 10854 |  | tags=30%,  list=19%,  signal=25% |  | UNC13A/SLC18A1/SLC6A9/SH3  GL2/DRD2/RAB27B/SLC18A2/OT  OF/SLC17A7/SYT11/RAB3B/TME  M163/AMPH/PRRT2/LAMP5/SYT  1/SYT5/SYT4/GABRA2/SYT6/O  PRD1/SYT9/SYT2/RAB3C/DRD3/  SLC32A1/SVOP/SV2C/UNC13C/  SLC17A8/SLC17A6/OR56A5/CPL  X3 |
| --- | --- | --- | --- | --- |
