# Supplementary material for: The Prognostic Value and Immunological Role of STEAP1 in Pan-Cancer: A Result of Data-Based Analysis
Source: Oxid Med Cell Longev. 2022 Mar 11;2022:8297011. doi: 10.1155/2022/8297011 (PMC8933652; doi:10.1155/2022/8297011)
Supplement: Supplementary 4 — Table S4: TMB for 33 cancer types and STEAP1 expression. [file 8297011.f4.doc]

| CancerType | cor | pValue | sig |
| --- | --- | --- | --- |
| ACC | 0.096977 | 0.395202 |  |
| BLCA | -0.05976 | 0.228433 |  |
| BRCA | -0.16563 | 2.01E-07 | *** |
| CESC | -0.07795 | 0.188712 |  |
| CHOL | -0.06969 | 0.686307 |  |
| COAD | 0.059403 | 0.238233 |  |
| DLBC | 0.014699 | 0.93139 |  |
| ESCA | -0.01888 | 0.812639 |  |
| GBM | -0.10929 | 0.186101 |  |
| HNSC | 0.121197 | 0.007116 | ** |
| KICH | 0.445571 | 0.0002 | *** |
| KIRC | 0.08656 | 0.115438 |  |
| KIRP | 0.053097 | 0.377812 |  |
| LAML | 0.058062 | 0.651266 |  |
| LGG | 0.154379 | 0.000525 | *** |
| LIHC | 0.110654 | 0.036107 | * |
| LUAD | 0.064073 | 0.151313 |  |
| LUSC | -0.05622 | 0.215034 |  |
| MESO | -0.12742 | 0.26311 |  |
| OV | -0.0004 | 0.99477 |  |
| PAAD | 0.234409 | 0.003767 | ** |
| PCPG | -0.06605 | 0.382385 |  |
| PRAD | 0.160629 | 0.0004 | *** |
| READ | 0.014486 | 0.869054 |  |
| SARC | -0.03253 | 0.619777 |  |
| SKCM | 0.04791 | 0.302574 |  |
| STAD | 0.015528 | 0.766553 |  |
| TGCT | 0.041995 | 0.615994 |  |
| THCA | -0.02029 | 0.65681 |  |
| THYM | 0.518253 | 2.17E-09 | *** |
| UCEC | 0.130582 | 0.00272 | ** |
| UCS | 0.183892 | 0.174882 |  |
| UVM | -0.02993 | 0.79213 |  |
