# Supplementary material for: The Prognostic Value and Immunological Role of STEAP1 in Pan-Cancer: A Result of Data-Based Analysis
Source: Oxid Med Cell Longev. 2022 Mar 11;2022:8297011. doi: 10.1155/2022/8297011 (PMC8933652; doi:10.1155/2022/8297011)
Supplement: Supplementary 5 — Table S5: MSI for 33 cancer types and STEAP1 expression. [file 8297011.f5.doc]

| CancerType | cor | pValue | sig |
| --- | --- | --- | --- |
| ACC | 0.031915 | 0.780082 |  |
| BLCA | -0.08067 | 0.103695 |  |
| BRCA | -0.03829 | 0.219339 |  |
| CESC | -0.11442 | 0.04696 | * |
| CHOL | -0.04788 | 0.781025 |  |
| COAD | 0.129962 | 0.007165 | ** |
| DLBC | -0.04896 | 0.741042 |  |
| ESCA | -0.03282 | 0.680309 |  |
| GBM | 0.104763 | 0.200484 |  |
| HNSC | 0.071691 | 0.110788 |  |
| KICH | 0.186515 | 0.136845 |  |
| KIRC | 0.115624 | 0.034391 | * |
| KIRP | -0.01342 | 0.821508 |  |
| LAML | 0.023523 | 0.848994 |  |
| LGG | 0.011451 | 0.796826 |  |
| LIHC | 0.029441 | 0.572933 |  |
| LUAD | -0.06295 | 0.155346 |  |
| LUSC | -0.00966 | 0.830603 |  |
| MESO | 0.056687 | 0.612963 |  |
| OV | -0.03227 | 0.596184 |  |
| PAAD | 0.085261 | 0.261921 |  |
| PCPG | -0.0557 | 0.460263 |  |
| PRAD | 0.093754 | 0.03705 | * |
| READ | 0.103316 | 0.205284 |  |
| SARC | 0.070794 | 0.261915 |  |
| SKCM | -0.04454 | 0.336357 |  |
| STAD | -0.07451 | 0.150404 |  |
| TGCT | 0.064332 | 0.434144 |  |
| THCA | 0.109151 | 0.015534 | * |
| THYM | 0.098257 | 0.289804 |  |
| UCEC | 0.015422 | 0.72116 |  |
| UCS | 0.061212 | 0.65404 |  |
| UVM | -0.08059 | 0.477334 |  |
