# Supplementary material for: The Prognostic Value and Immunological Role of STEAP1 in Pan-Cancer: A Result of Data-Based Analysis
Source: Oxid Med Cell Longev. 2022 Mar 11;2022:8297011. doi: 10.1155/2022/8297011 (PMC8933652; doi:10.1155/2022/8297011)
Supplement: Supplementary 6 — Table S6: associations between drug sensitivity and STEAP1 expression. [file 8297011.f6.doc]

| Gene | Drug | cor | pvalue |
| --- | --- | --- | --- |
| STEAP1 | TAK-733 | 0.484552 | 8.74E-05 |
| STEAP1 | PD-0325901 | 0.470255 | 0.00015 |
| STEAP2 | BMS-754807 | 0.469896 | 0.000152 |
| STEAP1 | Trametinib | 0.46727 | 0.000167 |
| STEAP1 | BMS-777607 | 0.45899 | 0.000225 |
| STEAP1 | RO-5126766 | 0.457411 | 0.000239 |
| STEAP1 | RO-4987655 | 0.454787 | 0.000262 |
| STEAP1 | Pimasertib | 0.45159 | 0.000293 |
| STEAP2 | Kahalide F | 0.44957 | 0.000314 |
| STEAP1 | AZD-0364 | 0.441951 | 0.000407 |
| STEAP1 | ARRY-162 | 0.440441 | 0.000429 |
| STEAP1 | Cobimetinib (isomer 1) | 0.436481 | 0.000489 |
| STEAP1 | SCH-772984 | 0.426193 | 0.000685 |
| STEAP1 | ARRY-704 | 0.421389 | 0.000799 |
| STEAP1 | PD 184352 | 0.411156 | 0.001101 |
| STEAP2 | CCT-271850 | 0.407972 | 0.001213 |
| STEAP1 | sitravatinib | 0.407017 | 0.001249 |
| STEAP1 | Selumetinib | 0.396181 | 0.001727 |
| STEAP2 | TPX-0005 | 0.393389 | 0.001874 |
| STEAP1 | ulixertinib | 0.391967 | 0.001953 |
| STEAP1 | LXH-254 | 0.3871 | 0.002247 |
| STEAP1 | LY-3214996 | 0.386694 | 0.002273 |
| STEAP2 | Hydrastinine HCl | -0.3857 | 0.002339 |
| STEAP1 | BMS-754807 | 0.385631 | 0.002343 |
| STEAP1 | EXEL-2280 | 0.383125 | 0.002516 |
| STEAP2 | PYRAZOLOACRIDINE | 0.382621 | 0.002552 |
| STEAP2 | sitravatinib | 0.3809 | 0.002678 |
| STEAP1 | VS-4718 | 0.370914 | 0.003529 |
| STEAP2 | Carboplatin | -0.36847 | 0.00377 |
| STEAP2 | LY-2835219 | 0.365119 | 0.004125 |
| STEAP2 | dimethylfasudil | 0.365107 | 0.004126 |
| STEAP1 | Refametinib | 0.361831 | 0.004501 |
| STEAP1 | CC-90003 | 0.361504 | 0.00454 |
| STEAP2 | EXEL-2280 | 0.360237 | 0.004695 |
| STEAP1 | LEE-011 | 0.359205 | 0.004823 |
| STEAP2 | S-63845 | -0.3546 | 0.005437 |
| STEAP1 | AZ-628 | 0.350414 | 0.006055 |
| STEAP2 | BMS-777607 | 0.344838 | 0.006971 |
| STEAP1 | Kahalide F | 0.343735 | 0.007166 |
| STEAP1 | CEP-40783 | 0.342203 | 0.007445 |
| STEAP2 | CG-806 | 0.340991 | 0.007672 |
| STEAP2 | JNJ-38877605 | 0.340912 | 0.007687 |
| STEAP2 | PF-04217903 | 0.338121 | 0.008235 |
| STEAP2 | Foretinib | 0.337534 | 0.008354 |
| STEAP2 | CEP-40783 | 0.337345 | 0.008393 |
| STEAP2 | ENMD-2076 | 0.335218 | 0.00884 |
| STEAP1 | PF-562271 | 0.334561 | 0.008982 |
| STEAP1 | KHK-Indazole | 0.333925 | 0.009121 |
| STEAP2 | BOS-172722 | 0.33273 | 0.009389 |
| STEAP2 | ADW-742 | 0.330972 | 0.009794 |
| STEAP2 | SAR-20347 | 0.330159 | 0.009987 |
| STEAP1 | Foretinib | 0.328987 | 0.01027 |
| STEAP2 | AS-703569 | 0.320645 | 0.012497 |
| STEAP2 | LY-2874455 | 0.32019 | 0.012629 |
| STEAP2 | LEE-011 | 0.319614 | 0.012799 |
| STEAP2 | JNJ-3887618 | 0.319176 | 0.012929 |
| STEAP1 | CCT-271850 | 0.318344 | 0.01318 |
| STEAP2 | CFI-402257 | 0.318178 | 0.01323 |
| STEAP2 | KHK-Indazole | 0.318015 | 0.01328 |
| STEAP1 | Cabozantinib | 0.317799 | 0.013346 |
| STEAP1 | ADW-742 | 0.317461 | 0.01345 |
| STEAP2 | Arsenic trioxide | -0.31655 | 0.013735 |
| STEAP1 | LY-2801653 | 0.314944 | 0.014248 |
| STEAP1 | AMD-070 | 0.31427 | 0.014468 |
| STEAP2 | Cisplatin | -0.31248 | 0.015069 |
| STEAP2 | BAY-1143572 | 0.310679 | 0.015692 |
| STEAP1 | TAE-226 | 0.308417 | 0.016507 |
| STEAP1 | PF-04217903 | 0.30643 | 0.017254 |
| STEAP1 | PF-03758309 | 0.304852 | 0.017866 |
| STEAP2 | BLU-667 | 0.302382 | 0.018863 |
| STEAP2 | Volitinib | 0.300346 | 0.019719 |
| STEAP2 | Ribavirin | 0.299517 | 0.020078 |
| STEAP2 | ON-123300 | 0.299227 | 0.020204 |
| STEAP2 | Cyclophosphamide | -0.29867 | 0.020447 |
| STEAP2 | Linsitinib | 0.29853 | 0.020511 |
| STEAP2 | TAE-226 | 0.298051 | 0.020724 |
| STEAP1 | XK-469 | -0.29774 | 0.020865 |
| STEAP1 | CG-806 | 0.296764 | 0.021307 |
| STEAP1 | Danusertib | 0.295317 | 0.021978 |
| STEAP1 | SEL-120 | 0.29516 | 0.022052 |
| STEAP2 | Sonidegib | 0.294147 | 0.022534 |
| STEAP1 | Altiratinib | 0.292672 | 0.023252 |
| STEAP2 | SOMCL-12-81 | 0.292507 | 0.023333 |
| STEAP2 | KW-2449 | 0.291865 | 0.023652 |
| STEAP1 | MLN-2480 | 0.291604 | 0.023783 |
| STEAP1 | Simvastatin | 0.28987 | 0.024668 |
| STEAP2 | GSK-1904529A | 0.289608 | 0.024804 |
| STEAP1 | Chlorambucil | -0.28758 | 0.025877 |
| STEAP1 | KW-2449 | 0.286619 | 0.026402 |
| STEAP1 | Tandutinib | 0.286603 | 0.026411 |
| STEAP1 | MLN-0905 | 0.286308 | 0.026573 |
| STEAP1 | AMG-51 | 0.285654 | 0.026936 |
| STEAP2 | XK-469 | -0.28329 | 0.02828 |
| STEAP1 | AEW-541 | 0.282863 | 0.028532 |
| STEAP2 | SGX-523 | 0.280954 | 0.029669 |
| STEAP1 | CFI-402257 | 0.280941 | 0.029677 |
| STEAP2 | Palbociclib | 0.278303 | 0.03131 |
| STEAP2 | Pipobroman | -0.27774 | 0.031668 |
| STEAP2 | AZD-5991 | -0.27652 | 0.032458 |
| STEAP2 | Rabusertib | -0.27637 | 0.032553 |
| STEAP1 | LY-3009120 | 0.276342 | 0.032572 |
| STEAP2 | S-64315 | -0.27599 | 0.032802 |
| STEAP2 | TAK-659 (isomer 1) | 0.274877 | 0.033543 |
| STEAP1 | ENMD-2076 | 0.274418 | 0.033851 |
| STEAP1 | Elliptinium Acetate | -0.27405 | 0.034104 |
| STEAP1 | 5-Fluoro deoxy uridine 10mer | -0.27162 | 0.035786 |
| STEAP1 | SGX-523 | 0.268792 | 0.037834 |
| STEAP1 | TAK-632 | 0.268443 | 0.038094 |
| STEAP1 | Everolimus | -0.26801 | 0.038416 |
| STEAP2 | R-547 | 0.267315 | 0.038942 |
| STEAP1 | RG-7602 | -0.2656 | 0.040265 |
| STEAP2 | Chlorambucil | -0.26555 | 0.040302 |
| STEAP2 | TAK-931 | 0.265524 | 0.040322 |
| STEAP2 | AT-9283 | 0.265409 | 0.040412 |
| STEAP1 | Rebimastat | 0.2648 | 0.04089 |
| STEAP2 | FGF-401 | 0.264791 | 0.040898 |
| STEAP2 | CCT-128930 | 0.26366 | 0.041799 |
| STEAP1 | JNJ-38877605 | 0.263588 | 0.041857 |
| STEAP2 | Simvastatin | 0.263481 | 0.041943 |
| STEAP1 | CEP-28122 | 0.263187 | 0.042182 |
| STEAP1 | Rocilinostat | 0.262998 | 0.042335 |
| STEAP1 | Pracinostat | 0.26248 | 0.042758 |
| STEAP1 | Temsirolimus | -0.26192 | 0.043217 |
| STEAP1 | BMS-536924 | 0.261707 | 0.043396 |
| STEAP1 | Encorafenib | 0.260393 | 0.044497 |
| STEAP1 | GDC-0994 | 0.258555 | 0.046077 |
| STEAP2 | Nilotinib | -0.25835 | 0.04626 |
| STEAP1 | Cyclophosphamide | -0.25827 | 0.046325 |
| STEAP1 | EPZ-020411 | 0.257712 | 0.046817 |
| STEAP1 | UNC-0638 | 0.257042 | 0.047412 |
| STEAP2 | Teglarinad | 0.256799 | 0.047629 |
| STEAP1 | Carboplatin | -0.25514 | 0.049136 |
| STEAP1 | Melphalan | -0.25507 | 0.049195 |
| STEAP1 | Triethylenemelamine | -0.25494 | 0.049318 |
