# Supplementary material for: The Prognostic Value and Immunological Role of STEAP1 in Pan-Cancer: A Result of Data-Based Analysis
Source: Oxid Med Cell Longev. 2022 Mar 11;2022:8297011. doi: 10.1155/2022/8297011 (PMC8933652; doi:10.1155/2022/8297011)
Supplement: Supplementary 7 — Figure S1: the differences of total protein expression of STEAP1 between normal and tumor samples in BRCA, COAD, LUAD, OV, and UCEC, respectively (a-e). [file 8297011.f7.pdf]

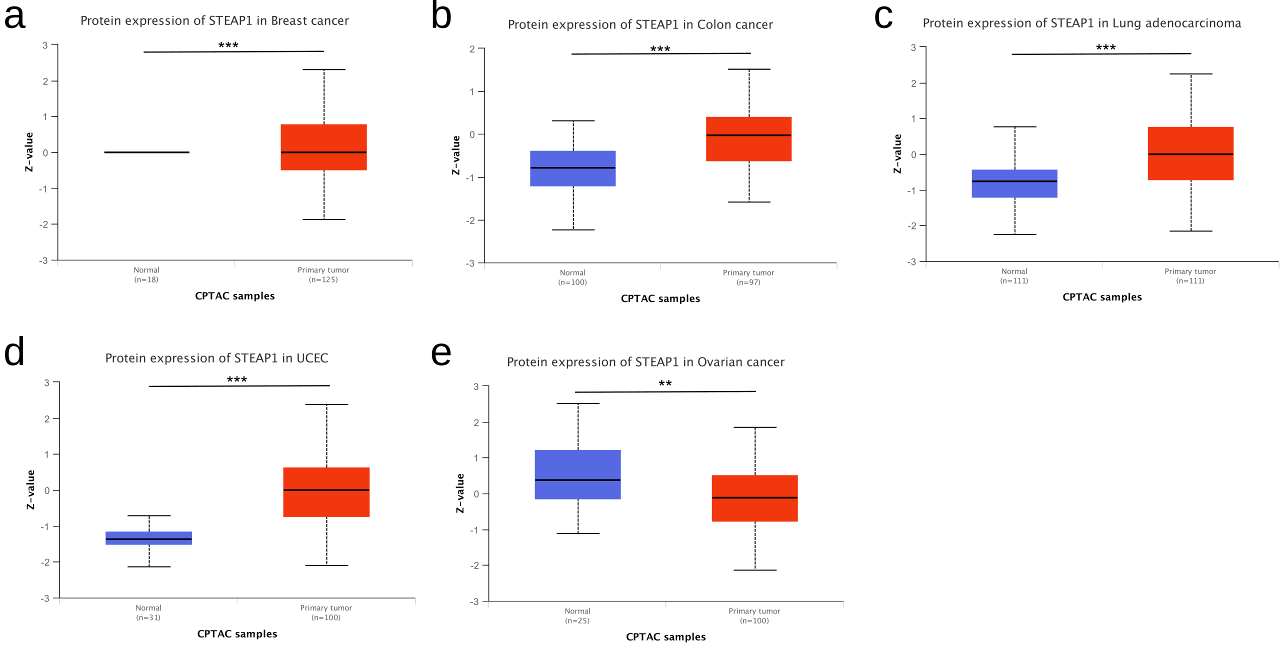
a
Protein expression of STEAP1 in Breast cancer
***
Z−value
3
2
1
0
-1
-2
-3
Normal
(n=18)
Primary tumor
(n=125)
CPTAC samples
b
Protein expression of STEAP1 in Colon cancer
***
Z−value
2
1
0
-1
-2
-3
Normal
(n=100)
Primary tumor
(n=97)
CPTAC samples
c
Protein expression of STEAP1 in Lung adenocarcinoma
***
Z−value
3
2
1
0
-1
-2
-3
Normal
(n=111)
Primary tumor
(n=111)
CPTAC samples
d
Protein expression of STEAP1 in UCEC
***
Z−value
3
2
1
0
-1
-2
-3
Normal
(n=31)
Primary tumor
(n=100)
CPTAC samples
e
Protein expression of STEAP1 in Ovarian cancer
**
Z−value
3
2
1
0
-1
-2
-3
Normal
(n=25)
Primary tumor
(n=100)
CPTAC samples
