# Supplementary material for: The Prognostic Value and Immunological Role of STEAP1 in Pan-Cancer: A Result of Data-Based Analysis
Source: Oxid Med Cell Longev. 2022 Mar 11;2022:8297011. doi: 10.1155/2022/8297011 (PMC8933652; doi:10.1155/2022/8297011)
Supplement: Supplementary 8 — Figure S2: correlations of STEAP1 expression and the methylation of its promoter in BRCA, COAD, LUSC, PRAD, SKCM, and UCEC, respectively (a–f). The red dotted lines represented the fitted lines of correlation tests. [file 8297011.f8.pdf]

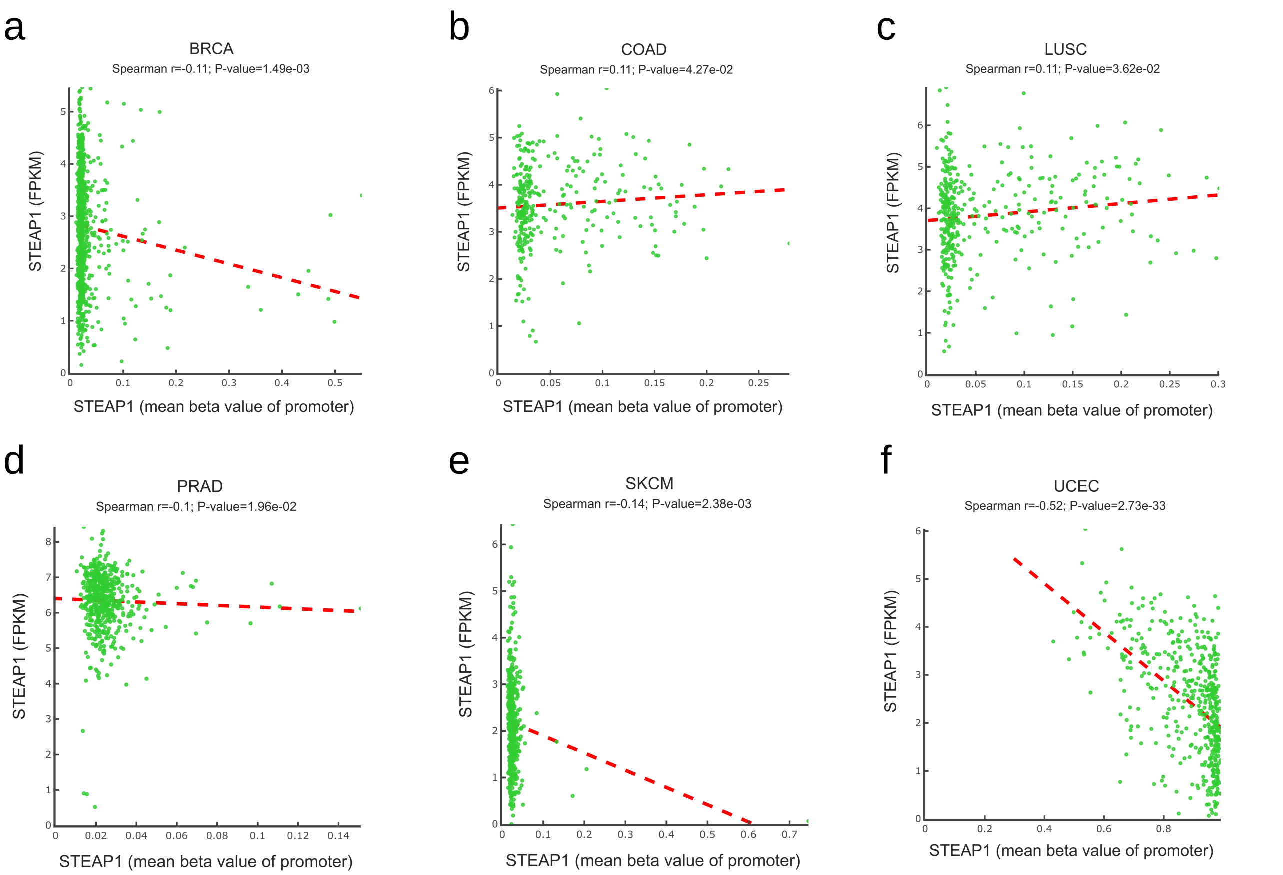

a
BRCA
Spearman r=-0.11; P-value=1.49e-03
STEAP1 (FPKM)
STEAP1 (mean beta value of promoter)
b
COAD
Spearman r=0.11; P-value=4.27e-02
STEAP1 (FPKM)
STEAP1 (mean beta value of promoter)
c
LUSC
Spearman r=0.11; P-value=3.62e-02
STEAP1 (FPKM)
STEAP1 (mean beta value of promoter)
d
PRAD
Spearman r=-0.1; P-value=1.96e-02
STEAP1 (FPKM)
STEAP1 (mean beta value of promoter)
e
SKCM
Spearman r=-0.14; P-value=2.38e-03
STEAP1 (FPKM)
STEAP1 (mean beta value of promoter)
f
UCEC
Spearman r=-0.52; P-value=2.73e-33
STEAP1 (FPKM)
STEAP1 (mean beta value of promoter)
