# Supplementary figures and images for: The Prognostic Value and Immunological Role of STEAP1 in Pan-Cancer: A Result of Data-Based Analysis
Source: Oxid Med Cell Longev. 2022 Mar 11;2022:8297011. doi: 10.1155/2022/8297011 (PMC8933652; doi:10.1155/2022/8297011)

**a**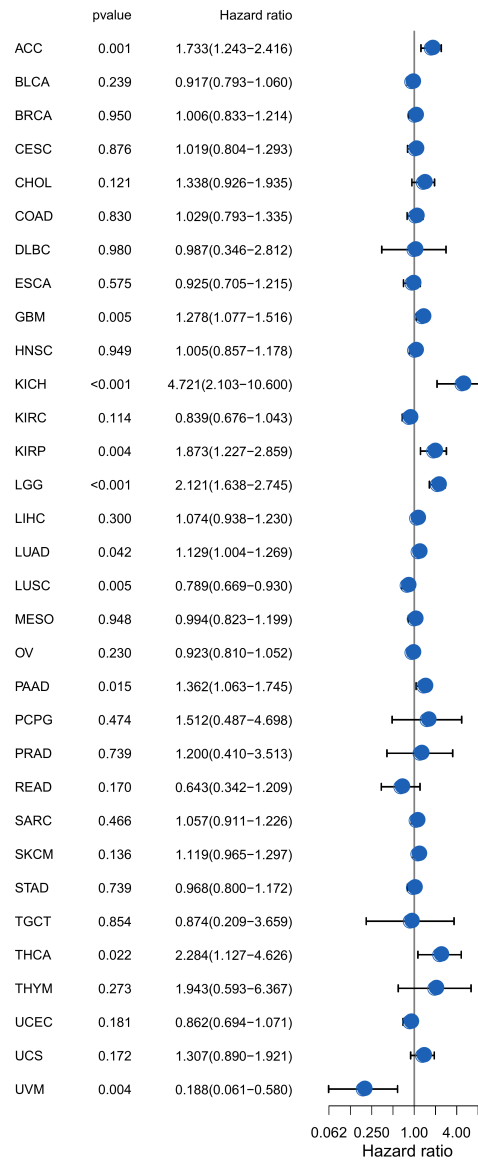**b**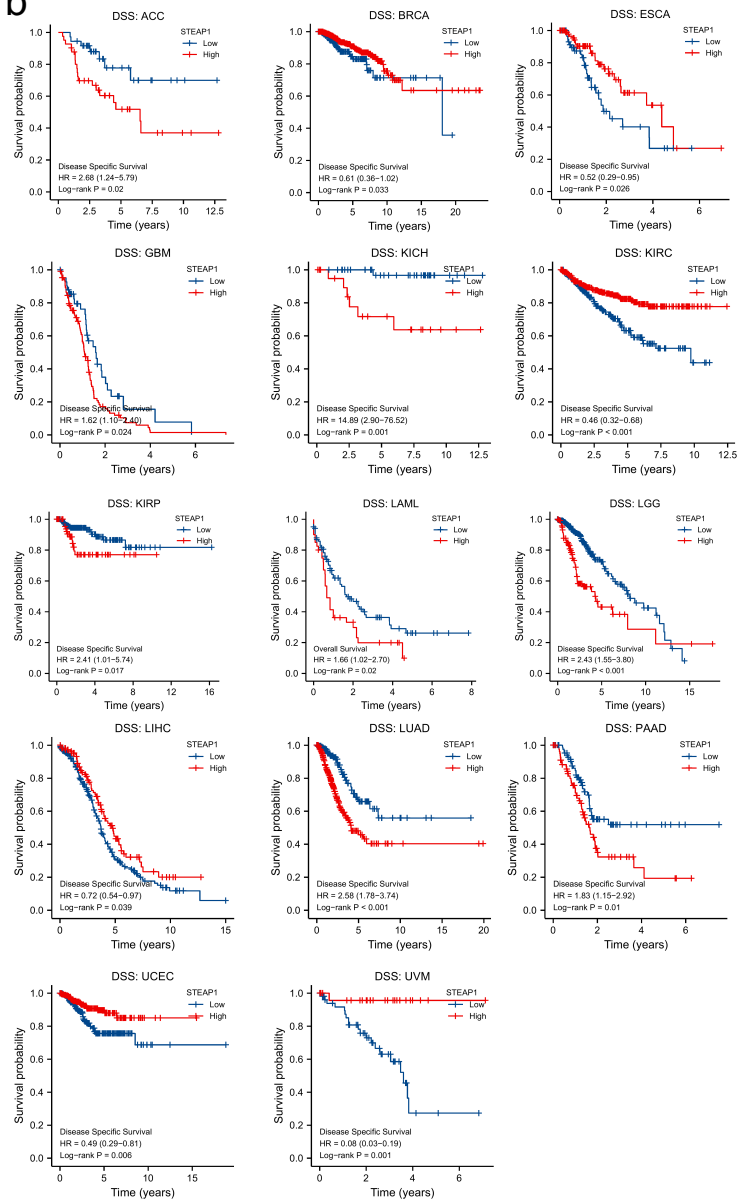

Supplement: Supplementary 9 — Figure S3: (a) a univariate Cox proportional hazard regression of DSS with STEAP1 expression was illustrated by a forest plot. (b) KM survival analysis of DSS between high- and low-expression groups of STEAP1. [file 8297011.f9.pdf]

a

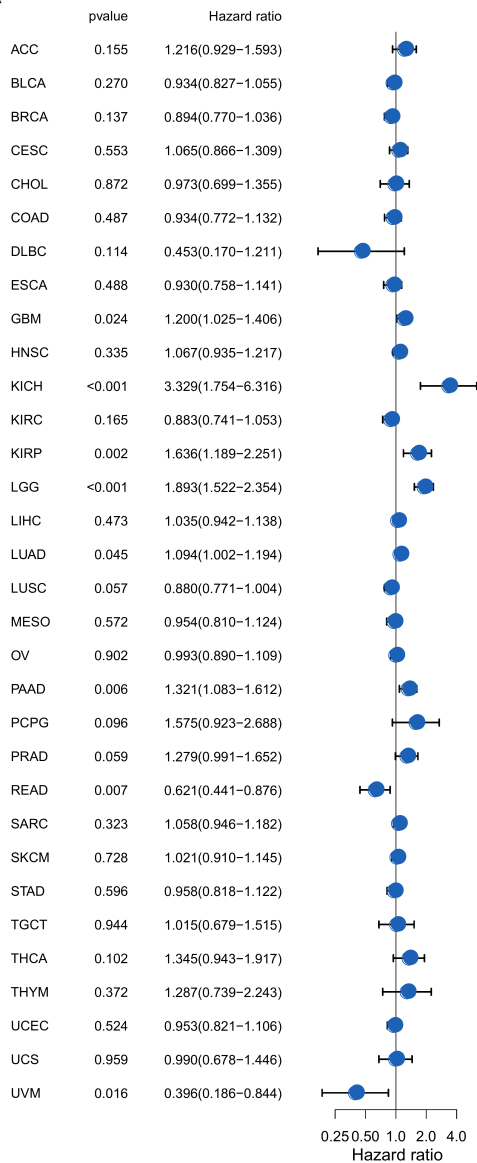

b

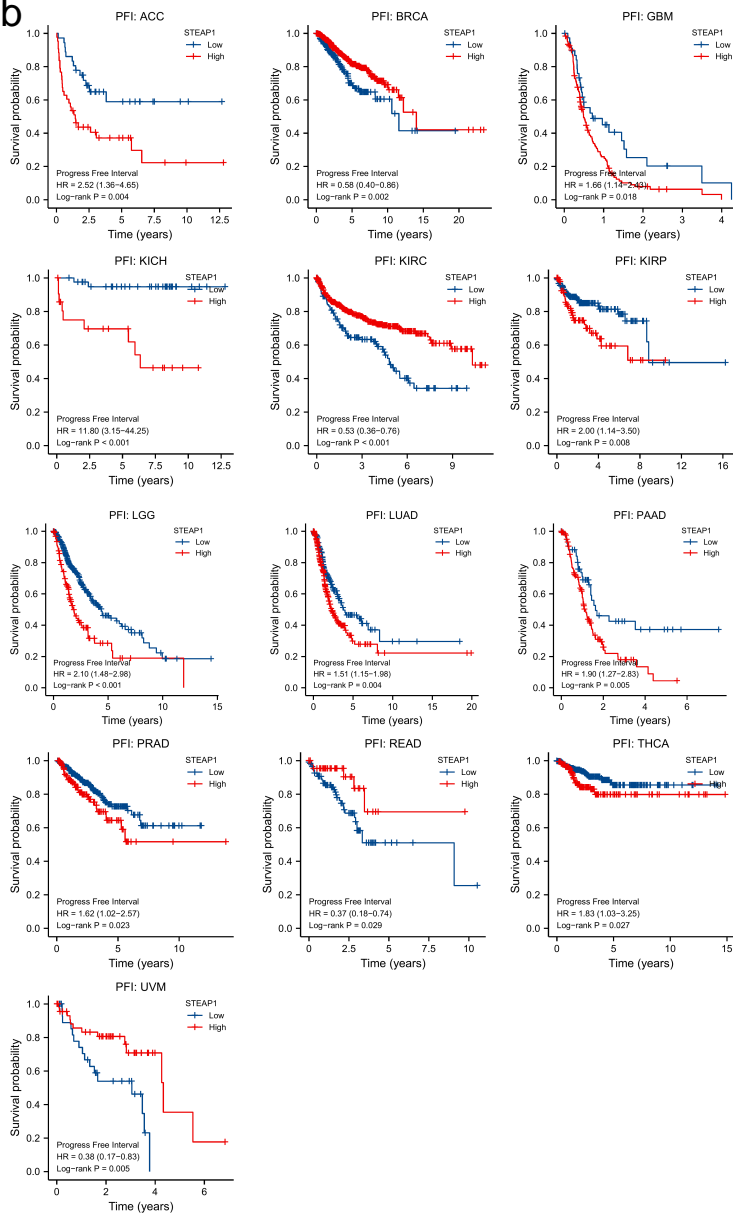

Supplement: Supplementary 10 — Figure S4: (a) a univariate Cox proportional hazard regression of PFI with STEAP1 expression was illustrated by a forest plot. (b) KM survival analysis of PFI between high- and low-expression groups of STEAP1. [file 8297011.f10.pdf]

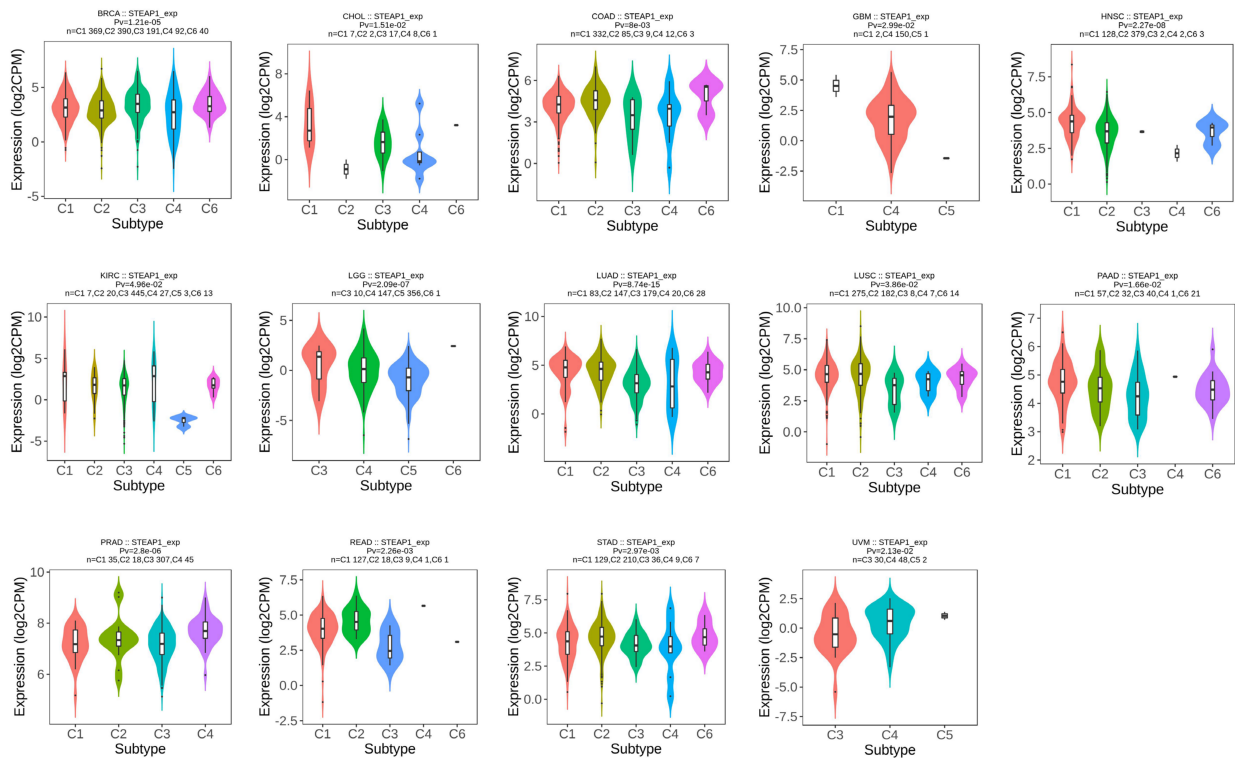

Supplement: Supplementary 11 — Figure S5: the differences of STEAP1 expression among distinctive immunophenotypes in multiple cancers (C1: wound healing; C2: IFN-gamma dominant; C3: inflammatory; C4: lymphocyte depleted; C5: immunologically quiet; C6: TGF-β dominant). [file 8297011.f11.pdf]

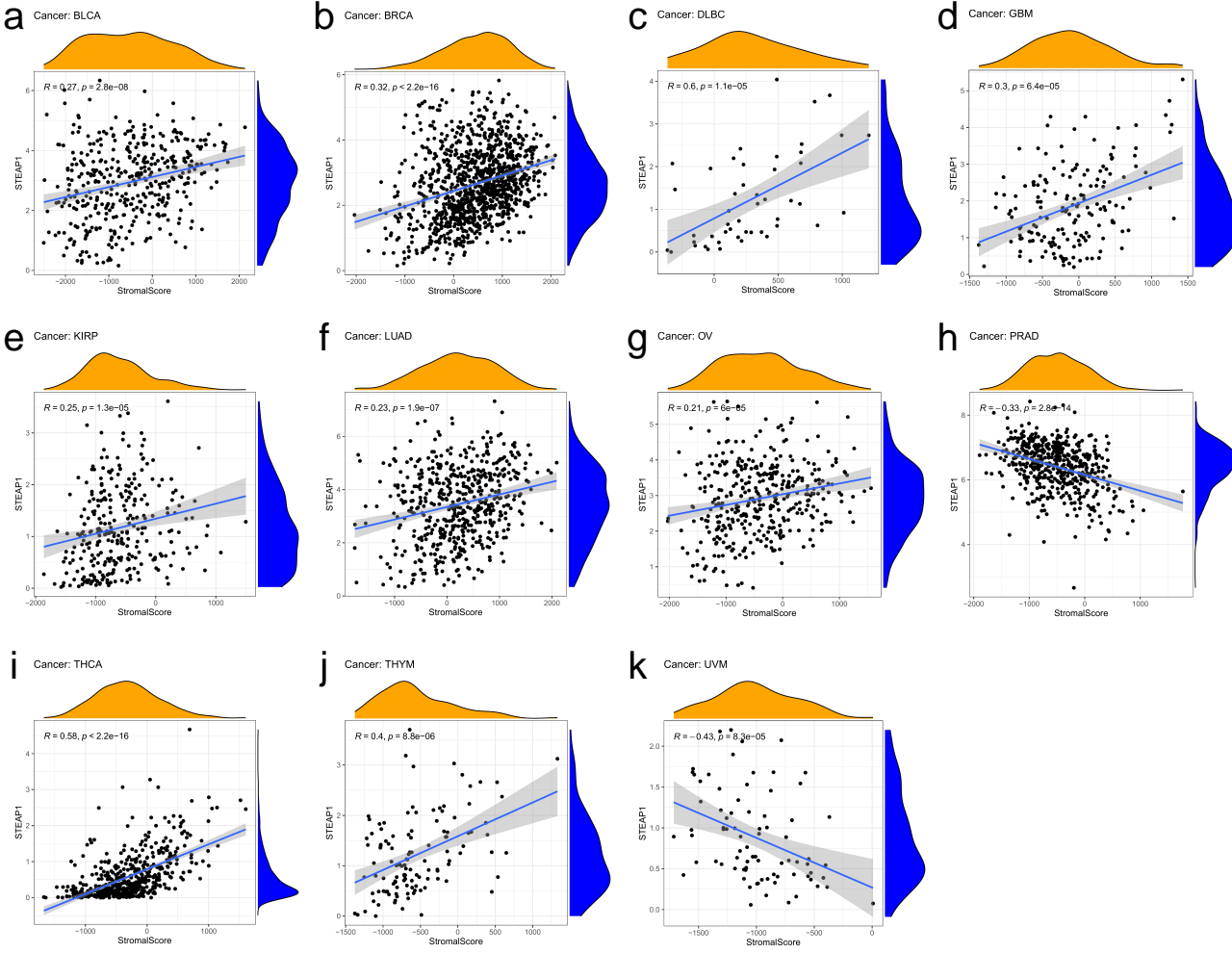

Supplement: Supplementary 12 — Figure S6: significant correlations of STEAP1 expression and stromal score were examined by ESTIMATE algorithm. [file 8297011.f12.pdf]
